# Phosphonomethyl (Thio)Phosphonamidite Reagents for the Construction of Phosphonylmethyl Phosphono(Di)Thioate Mono‐ and Diesters

**DOI:** 10.1002/chem.71563

**Published:** 2026-08-21

**Authors:** Pascal Balić, Roy Steneker, Gijsbert A. van der Marel, Jeroen D. C. Codée, Dmitri V. Filippov

**Affiliations:** ^1^ Leiden Institute of Chemistry Leiden University Leiden The Netherlands

**Keywords:** biomimetics, nucleosides, phosphorylation, pyrophosphate

## Abstract

A repertoire of phosphonomethyl (thio)phosphonamidite reagents has been developed to enable the controlled synthesis of phosphonylmethyl phosphono(di)thioate analogues of intrinsically labile pyrophosphates. Selective amidophosphonitylation of an alcohol or thiol of choice with a phosphonomethyl phosphonbisamidite allows access to four (thio)phosphonamidite reagents bearing three orthogonal protecting groups that can be chemoselectively addressed. These reagents were isolated in homogeneous form in yields up to 95% and applied in a sequence involving azole‐mediated phosphonamidite coupling with an alcohol and successive in situ sulfurization of the P^III^‐P^V^ intermediate, which affords protected phosphonylmethyl phosphono(di)thioate tetraesters. The coupling and sulfurization steps were monitored with ^31^P‐NMR, revealing the nature of the key intermediates and corroborating the high efficiency of the coupling and sulfurization reactions. Immediate global deprotection or subsequent site‐selective deprotection of the terminal phosphonate followed by a phosphonodiester condensation furnishes unprecedented phosphonylmethyl phosphono(di)thioate mono‐ and nonsymmetrical diesters, respectively. Using this method, we have assembled four nucleoside diphosphate analogs as well as a set of eight stable CDP‐glycerol and CDP‐ribitol mimics, all in multimilligram amounts.

## Graphical abstract

A set of new phosphonomethyl (thio)phosphonamidite reagents is introduced to generate unprecedented pyrophosphate mimetics. The versatile P(III)‐P(V) reagents allow for the construction of various pyrophosphate isosteres, and the applicability of the chemistry is illustrated in the assembly of a diverse set of stabilized CDP‐glycerol and CDP‐ribitol analogues.

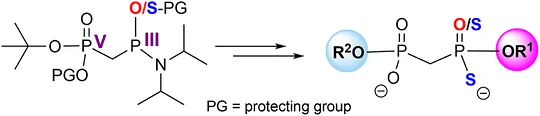

## 1 Introduction

Pyrophosphates (Figure 1) are characteristic structural elements that are found in a multitude of biological molecules that are central to life [1, 2, 3]. They are found in many of the co‐factors (e.g., NAD/NADH, FAD/FADH_2_, thiamine pyrophosphate, Coenzyme A) that play a role in virtually all biosynthesis pathways and are crucial components of the building blocks that are used to assemble nucleic acids (from nucleoside triphosphates), terpenes (constructed from prenyl pyrophosphates), and polysaccharides and glycoconjugates (requiring sugar nucleoside diphosphates), where they serve as privileged leaving groups [4, 5, 6]. Their high reactivity, however, also renders these molecules sensitive to chemical and enzymatic hydrolysis. Stabilized pyrophosphate mimics have attracted significant attention for studying the interaction of these molecules and their target enzymes at the (sub)molecular level [7, 8, 9, 10]. Methylene bisphosphonates (MBPs, see Figure 1) have been introduced as non‐hydrolysable pyrophosphate isosteres and stand out for their close structural homology with pyrophosphates and their supreme stability [11, 12, 13]. They have found wide application in the generation of biomimetic compounds, used for structural studies as well as for the development of biosynthesis inhibitors and probes to unravel the mode of action of pyrophosphate‐processing enzymes [14, 15]. To enable the assembly of nonsymmetric MBPs, in which both sides of the MBP moiety are functionalized, we have previously introduced reagent **1** that features both a P(V) and a P(III) center [16]. The high reactivity of the phosphonamidite moiety allows for the effective reaction of a variety of alcohols, and after oxidation of the P(III) center, a fully protected MBP‐tetraester is formed. Selective removal of the *tert*‐butyl group then sets the stage for the second condensation event, for which different agents can be used.

**FIGURE 1:**
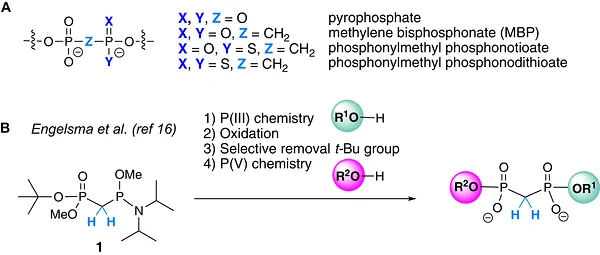
(A) Generic structures of pyrophosphates, methylene bisphosphonates (MBPs), phosphonylmethyl phosphonothioates, and phosphonylmethyl phosphonodithioates; (B) P(V)‐P(III) MBP reagent **1** introduced for the synthesis of nonsymmetric MBPs.

The P(III) center not only endows reagent **1** with exceptional reactivity, it also opens up possibilities for diversification chemistry on this side, for example, through sulfurization chemistry, which would lead to unprecedented phosphonomethyl phosphonothioates. The substitution of an oxygen atom in phosphate mono‐ or diesters has been widely explored. Phosphorothioates, in which one of the non‐bridging oxygens has been replaced by a sulfur atom, have attracted much attention because of their enhanced stability against nucleases and improved cellular uptake [17, 18]. The replacement of oxygen for sulfur atoms in pyrophosphate and triphosphate moieties has also been described. For example, replacing the central O = P‐O^−^ with a O = P‐S^−^ moiety in a triphosphate mRNA cap structure has been shown to confer beneficial properties, with the modified cap showing higher affinity for its designated binding partner, the eukaryotic translation initiation factor 4E (eIF4E) [19]. This increased binding affinity has been traced back to the more localized negative charge on the sulfur atom. At the same time, the higher polarizability of the sulfur atom has also been shown to favor association with softer cations, such as Zn^2+^ and Mn^2+^, used by many enzymes for coordinating pyrophosphates [20, 21, 22]. These beneficial effects, sometimes referred to with the colloquial term “thio‐effect” [23], have provided the incentive for the development of reagents **3–6** (Scheme 1). These reagents enable the flexible introduction of phosphonomethyl phosphonothioate and phosphonodithioate moieties in pyrophosphate isosteres via mild activation and sulfurization chemistries. We demonstrate the versatility of P(III)‐P(V) reagents **3–6** in generating a set of nucleotide bisphosphonate analogues and a library of cytidine diphosphate glycerol (CDP‐Gro) and ribitol (CDP‐Rbo) analogues. CDP‐Gro and CDP‐Rbo are central building blocks for the biosynthesis of bacterial polysaccharides and teichoic acids, in which the nucleoside diphosphates are used to build polymers of repeating alditol phosphate moieties. Wall teichoic acids (WTAs) contribute profoundly to the virulence of Gram‐positive bacteria, such as the multidrug‐resistant pathogen *Staphylococcus aureus* and inhibition of the enzymes involved in the biosynthesis of WTAs is being explored as a promising avenue for the discovery of new antibiotics [24].

**SCHEME 1:**
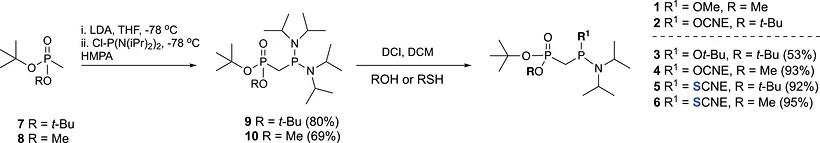
Generation of the toolbox of MBP reagents **1–6**.

## 2 Results and Discussion

### 2.1 Reagent Synthesis

To generate terminal MBPs (phosphonomethyl phosphonothioate and dithioate monoesters) and nonsymmetric MBPs (phosphonomethyl phosphonothioate and dithioate diesters), we set out to expand the toolbox of phosphonodiester‐phosphonamidite P(V)‐P(III) reagents (Scheme 1). We previously reported reagents **1** and **2** and now introduce compounds **3–6**. Reagents **3** and **5** carry two *tert*‐butyl esters at the P(V) center and are therefore ideally suited for the construction of terminal MBPs, whereas the P(V) methyl esters **4** and **6** were designed as tools for the construction of nonsymmetric MBP diesters. Reagents **5** and **6** are the thio‐analogues of **2** and **4**, respectively, and are introduced as reagents for the construction of phosphonodithioate MBPs.

In line with the generation of reagent **2**, we accessed reagents **3–6** from bisamidites **9** and **10**, which in turn were generated by lithiation of the methylphosphonates **7** [25] or **8** [26], respectively, and reaction with bis(di‐*iso*‐propylamino)chlorophosphine (Scheme 1). Initially, these reactions were conducted using THF as a solvent and while the formation of **9** proceeded uneventfully (as reported previously), the formation of **10** was thwarted by the formation of methyl bis(di‐*iso*‐propylamino)phosphoramidite (MeOP(N(*i*Pr)_2_)_2_; δ_P_ 132.3 ppm) and phosphonometyl methylphosphinate (See Figure S1 for ^31^P NMR monitoring of the reaction), through a phospha‐Claisen self‐condensation (Figure S1A,C) [27]. To suppress this side‐reaction, we found that hexamethylphosphoramide (HMPA) could be used as a co‐solvent to solvate the lithium counterion and increase the nucleophilicity of the methylene anion while preventing self‐condensation because of Coulombic repulsion between the more naked anions(Figure S1B,D). This way, bisamidite **10** was formed in 69% yield. Bisamidites **9** and **10** are versatile intermediates, as they can be used to install a variety of alcohols or thiols to deliver a set of reagents featuring different protecting group patterns [28, 29, 30, 31, 32]. Thus, amidophosphonitylation of *tert*‐butanol using **9** under the agency of 0.6 eq. 4,5‐dicyanoimidazole (DCI) delivered **3** in 53% yield and a similar reaction of **10** with 2‐cyanoethanol gave **4** (93%). For the synthesis of phosphonomethyl phosphonodithioates we decided to target reagents **5** and **6** carrying a 2‐cyanoethyl group to mask the thioate moiety. The 2‐cyanoethyl group is one of the most popular phosphate protecting groups and its thiol counterpart has previously been used for the construction of phosphorodithioates [33]. Amidophosphonitylation of freshly prepared 2‐cyanoethylmercaptan [34] proceeded uneventfully, as corroborated by ^31^P‐NMR (Figures S2 and S3). After purification over high‐grade ultra‐pure silica gel to avoid oxidation, dismutation, and Arbuzov‐type rearrangements [35], reagents **5** and **6** were obtained in excellent yield and purity.

### 2.2 Synthesis of Phosphonylmethyl Phosphonothioate and Phosphonodithioate Monoesters

With the set of MBP reagents in hand, we set out to explore their use in the generation of a set of MBP monoesters as analogues adenosine, cytidine and guanosine diphosphates (**13a**, **13b**, **16,** and **19**, Scheme 2). We started with the synthesis of ADP‐mimics **13a** and **13b** (Scheme 2, top). Toward this end, we treated protected adenosine **11** [36] with phosphonomethyl phosphonamidite reagent **2** under the aegis of a slight excess of DCI, which furnished the intermediate phosphonate phosphonite tetraester (not shown in Scheme 2), in near quantitative yield, within 30 min, as revealed by ^31^P NMR spectroscopy (Figure 2B). For the in situ sulfurization of the formed P^V^‐P^III^ intermediate, we first explored 3*H*‐1,2‐benzodithiol‐3‐one [37], which gave the fully protected phosphonylmethyl phosphonothioate tetraester **12a** in 75% yield. ^31^P NMR analysis of the reaction revealed signals of byproducts (δ_P_ 66.05, *J*_PP_ 15.9 Hz; 65.95, *J*_PP_ 14.6 Hz; 65.24, *J*_PP_ 13.9 Hz) that correspond with the chemical shift of charged phosphonothioate moieties, indicating partial hydrolysis or deprotection of the phosphonothioate. To prevent the formation of these side products, the use of Beaucage sulfurizing reagent [38] was explored. By virtue of its higher reactivity, this cleanly furnished the desired phosphonylmethyl phosphonothioate (Figure 2C), and adenosine tetraester **12a** was isolated in 92% yield. A two‐step deprotection sequence was used to complete the synthesis of adenosine phosphonylmethyl phosphonothioate **13a**: acidolysis of the *tert*‐butyl moieties under the agency of HCl/HFIP afforded the bisphosphonic diesters quantitatively, and subsequent deacylation and concomitant β‐elimination of the cyanoethyl group were effected by overnight treatment with aqueous ammonia. Extractive work‐up, size‐exclusion chromatography, and lyophilization then yielded terminal adenosine phosphonylmethyl phosphonothioate **13a** in 49% yield as an *R*_P_/*S*_P_ diastereomeric mixture. In similar vein, cytidine phosphonylmethyl phosphonothioate **16** was generated from cytidine nucleoside **14** with the aid of reagent **2** in 66% overall yield (Scheme 2, middle).

**SCHEME 2:**
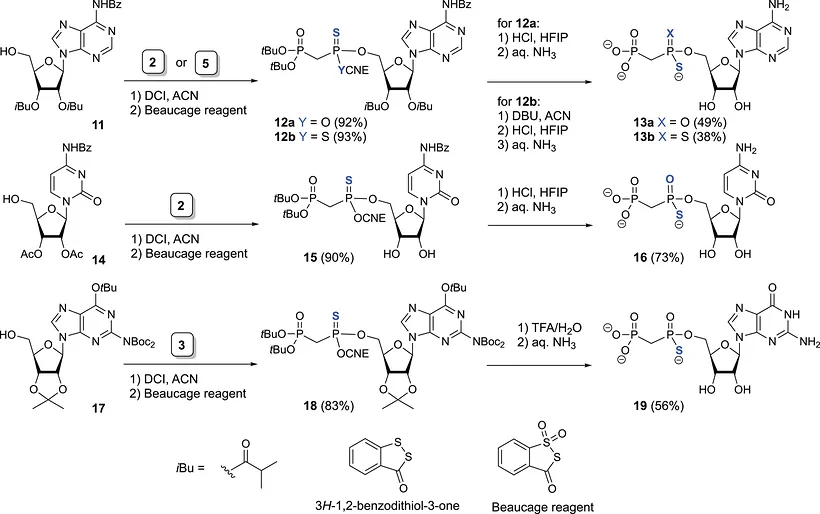
Synthesis of phosphonylmethyl phosphonothioate and phosphonodithioate nucleotides.

**FIGURE 2:**
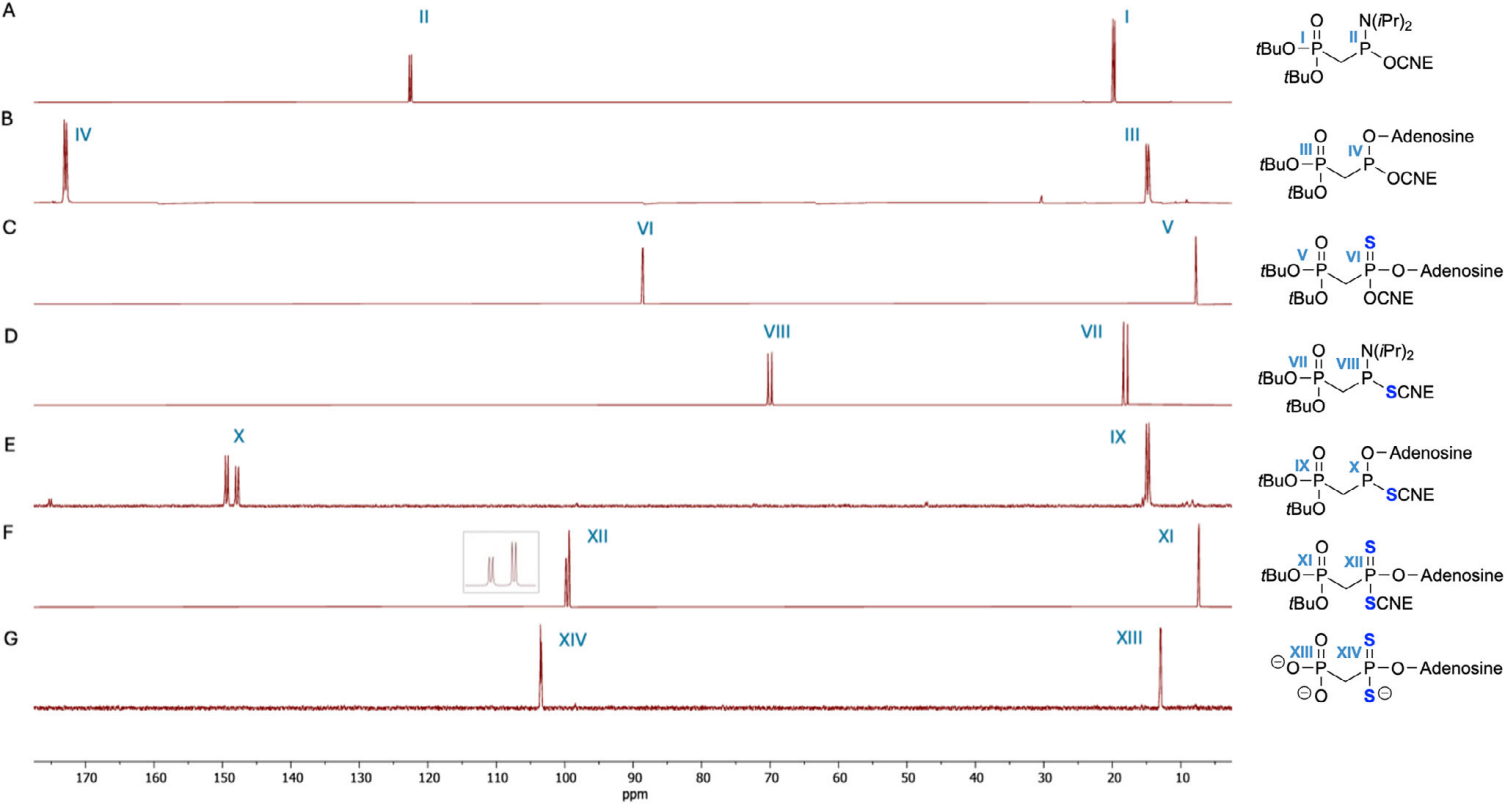
Condensation and sulfurization reactions of reagents **2** and **5** followed by ^31^P NMR spectroscopy. (A) Reagent **2**; (B) Condensation of **2** with adenosine **11**; (C) Sulfurization to give **12a**; (D) Reagent **5**; (E) Condensation of **5** with adenosine **11**; (F) Sulfurization to give **12b**. The P(= S)S diastereoisomers are clearly resolved; (G) Deprotection of **12b** to give **13b** with two non‐chiral P‐atoms.

A more direct approach to the synthesis of terminal methylenebisphosphonate analogues would entail the use of reagent **3**, which enables a one‐step deprotection event at the end of the synthesis. The use of this reagent was explored in the synthesis of GDP analogue **19** (Scheme 2, bottom). Thus, guanosine building block **17**, featuring protecting groups that can all be removed under acidic conditions [39], was used in an azole‐mediated coupling with reagent **3** and subsequent in situ sulfurization under the agency of Beaucage reagent, affording protected tetraester **18** in 83% yield. Global deprotection by overnight treatment with aqueous trifluoroacetic acid provided **19** after size‐exclusion chromatography.

Having established the chemistry for the generation of phosphonylmethyl phosphonothioate monoesters, we next set out to explore the generation of achiral dithioates. To this end, we paired adenosine building block **11** with reagent **5**, the sulfur counterpart of reagent **2** (Scheme 2, top), and followed the reaction by ^31^P NMR (Figure 2). Under the agency of a small excess of DCI, adenosine **11** and reagent **5** (^31^P NMR, Figure 2D) were coupled to give a near‐quantitative conversion to the diastereomeric P^V^‐P^III^ phosphonate‐thiophosphonite intermediate within 10 min (Figure 2E). Notably, it has previously been reported that thioamidite reagents are harder to activate with tetrazole than their phosphoramidite analogues, leading to ∼300‐fold lower coupling rates [33, 40]. The apt reactivity of reagent **5** indicates that the methylene substituent effectively negates the disarming effect of the thio‐substituent. Sulfurization of the P^V^‐P^III^ intermediate with Beaucage sulfur‐transfer reagent gave smooth conversion into dithioate tetraester **12b**, which was isolated in 93% as a mixture of *R*_P_/S_P_ diastereomers (Figure 2F). Next, deprotection conditions for dithioate tetraester **12b** were sought. Initial deblocking conditions consisted of HCl/HFIP‐assisted acidolysis of the *tert*‐butyl moieties and successive removal of the cyanoethyl and acyl protecting groups by overnight treatment with ammonium hydroxide. This, however, led to a complex mixture with monothioate contaminants. It has previously been reported that the deprotection of mercaptocyanoethyl protecting groups with ammonium hydroxide can give high levels of monothioate side products [41]. Jones et al. demonstrated that thioates and dithioates are accessible from thiocyanoethylphosphates when treated with DBU to effect β‐elimination [42, 43]. Therefore, alternative deprotection conditions were explored and we subjected **12b** to treatment with excess DBU, after the acidolysis of the *tert*‐butyl esters. Unfortunately, partial degradation was still observed. We therefore reversed the deprotection steps and first removed the cyanoethyl group through DBU‐mediated β‐elimination, which led to the clean and quantitative conversion into the achiral dithioate (δ_P_ 103.5, *J*_PP_ 15.3 Hz) within an hour. Ensuing acidolysis using 3.5 eq. HCl in HFIP then liberated the terminal phosphonic acid and deacylation via ammonolysis treatment gave adenosine phosphonylmethyl phosphonodithioate **13b** (Figure 2G) after gel filtration and lyophilization, as a homogeneous white powder in 38% yield.

### 2.3 Synthesis of Nonsymmetric Phosphonylmethyl Phosphonothioate and Phosphonodithioate Diesters

With the reagents and conditions established for the assembly of the phosphonylmethyl phosphonothioate and phosphonodithioate monoesters, we next set out to explore the use of the P(III)‐P(V) reagents in the construction of nonsymmetric diesters. Therefore, we targeted a library of CDP‐Gro and CDP‐Rbo analogues as depicted in Scheme 3. Reagent **4** was explored first, in combination with different glycerol and ribitol building blocks. Thus, phosphonitylation of cytidine nucleoside **14** [44] with phosphonamidite **4** and consecutive sulfurization of the P^III^ center afforded phosphonylmethyl phosphonothioate tetraester **20a** in 90% yield (Scheme 3, top). The synthesis of **20a** was monitored by ^31^P NMR (Figure S4A–C) and showed clean conversions in both steps. Subsequently, the fully protected tetraester was quantitatively converted into phosphonic acid **21a** with HCl/HFIP to set the stage for the phosphonodiester condensation. We previously successfully used Wada's reagent, 3‐nitro‐1,2,4‐triazol‐1‐yl‐tris(pyrrolidin‐1‐yl)phosphonium hexafluorophosphate [45] (PyNTP), for the condensation of methylenephosphonic [16] and difluoromethylene phosphonic acids [46], and therefore resorted to the use of this reagent to unite phosphonic acid **21** and the protected glycerol and ribitol building blocks. Gratifyingly, commercially available (*S*)‐ or (*R*)‐solketal **22** and **23** were combined with **21a** to provide bisphosphonate tetraesters **25a** and **26a** in excellent yields (Scheme 3, middle‐left and middle‐center). Hereafter, demethylation with 20 equivalents of thiophenolate in the presence of triethylamine gave the corresponding phosphonates. It was observed that the cyanoethyl groups were also partially removed (∼ 30%). Subsequently, acetal hydrolysis was realized by treatment with AcOH/H_2_O at 40°C, whereafter the remaining base‐labile protecting groups (i.e., acyl and remaining cyanoethyl) were removed using aqueous ammonia. Extractive workup and purification by gel filtration and ion‐exchange chromatography afforded phosphonylmethyl phosphonothioate CDP‐Gro analogues **28a** and **29a** in multi‐mg quantities.

**SCHEME 3:**
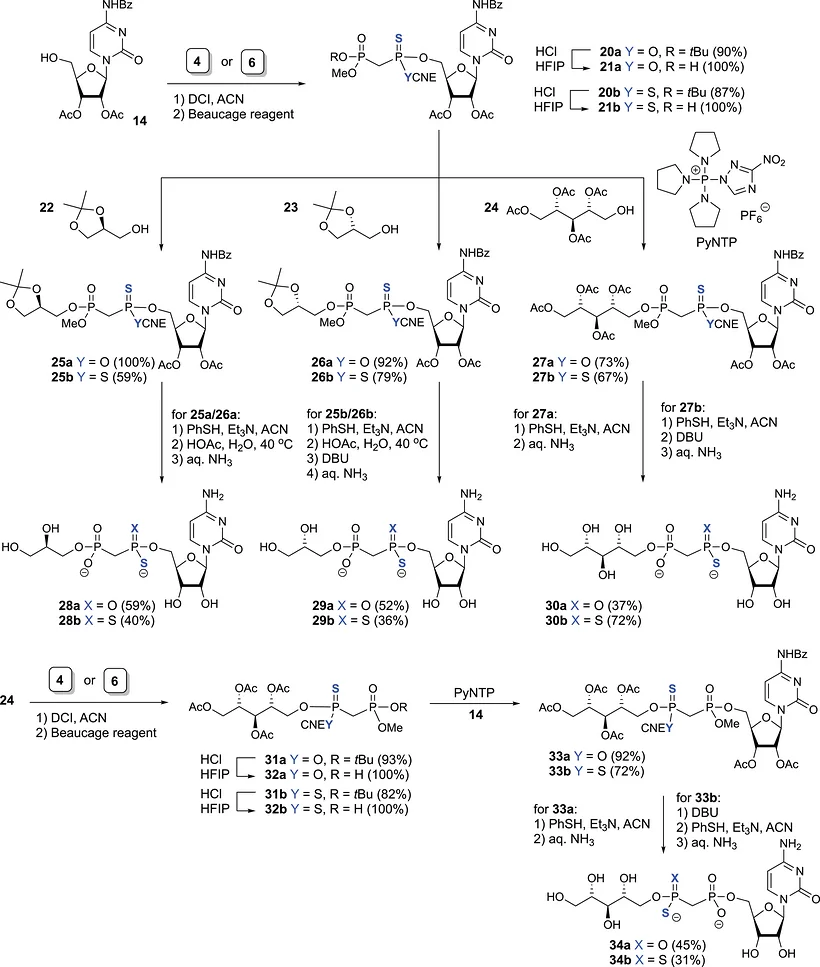
Assembly of phosphonylmethyl phosphono(di)thioate CDP‐glycerol and CDP‐ribitol analogues.

Next, ribitol **24** was subjected to the same sequence of reactions to generate CDP‐Rbo analogue **30a** (Scheme 3, middle‐right). Thus, ribitol **24** was condensed with phosphonic acid **21a** under the agency of Wada's reagent to afford protected tetraester **27a**. Removal of the protecting groups was accomplished through demethylation with thiophenolate in MeCN/TEA, followed by ammonolysis. Purification by size‐exclusion and ion‐exchange chromatography afforded homogeneous CDP‐ribitol analogue **30a** in good overall yield and purity.

With the phosphonothioate targets assembled, we tended to the generation of dithioates **28b**, **29b,** and **30b**. Hereto, phosphonthioamidite **6** was condensed with cytidine **14** (Scheme 3, top) to afford the intermediate P^V^‐P^III^ phosphonate‐phosphonothioite tetraester within 30 min. Subsequent in situ sulfurization afforded tetraester **20b** in 87% yield after aqueous work‐up and flash column chromatography (see Figure S4D–F for ^31^P NMR monitoring of the synthesis of **20b**). The remaining 13% was a complex mixture of monothionated and other by‐products. Formation of these side products can be accounted for by the high leaving group capacity of the thio moieties on the tervalent phosphorus center, as also reported by Bjergårde and Dahl [33], who demonstrated that their reactivity is on par with NR_2_ groups under azole catalysis. This indicates that it is necessary to sulfurize the P^V^‐P^III^ intermediate as quickly as possible. Chemoselective deblocking of the terminal phosphonate by removal of the *tert*‐butyl moiety with HCl/HFIP at ambient temperature again proceeded smoothly, quantitatively affording triester **21b**. Using Wada's reagent, the phosphonic acid was coupled to (*R*)‐ or (*S*)‐solketal (**22** or **23**) or tetra‐acetyl ribitol **24** to give dithioate tetraesters **25b**, **26b,** and **27b** in overall good yields (Scheme 3, middle). Deprotection of **25b/26b** was effected by first removing the methyl esters using thiophenolate. Notably, no cleavage of the cyanoethyl groups was observed during these transformations. The isopropylidene ketals were next cleaved by treatment with AcOH/H_2_O after which excess DBU was used to induce the β‐elimination of the cyanoethyl group. After full deblocking of the phosphonodithioate core (as gauged by ^31^P NMR spectroscopy) ammonolysis of the acetyl and benzoyl groups delivered CDP‐(*R*)‐ and (*S*) ‐glycerol dithioate isosteres **28b** and **29b**, respectively. CDP‐ribitol analogue **27b** was deprotected by demethylation, decyanoethylation and ammonolysis to provide dithioate **30b** in excellent yield and purity after gel filtration.

Finally, having synthesized the first six CDP‐glycerol and ribitol mimetics, attention was directed to generate CDP‐ribitol mimetics, bearing the phosphono(di)thioate moiety on the opposite terminus, that is, on the side of ribitol, to illustrate the flexibility of the developed reagents (Scheme 3, bottom) To this end ribitol **24** was condensed with reagent **4** or **6** to give, after sulfurization, **31a** and **31b**. Acidolytic removal of the *tert*‐butyl group and consecutive phosphonodiester condensation with cytidine nucleoside **14** afforded tetraesters **33a** and **33b** in 92% and 86% yield, respectively (Scheme 3, bottom‐right). Thioate **33a** was deprotected by unmasking the methylester and subsequent global deacylation to afford CDP‐ribitol analogue **34a**. Dithioate **33b** was unmasked by first removing the cyanoethyl group, followed by demethylation. This required a large excess of thiophenolate and a prolonged reaction time (12 h), probably due to Coulombic repulsion and the diminished leaving‐group capacity of the anionic dithioate. Ammonolysis then delivered the final target compound **34b**.

## 3 Conclusion

In conclusion, we have introduced a set of new methylene‐bridged P(III)‐P(V) reagents and chemistries for the generation of phosphonylmethyl phosphono(di)thioates, representing unprecedented pyrophosphate mimetics. The chemistry used to prepare the reagents is flexible and allows the installation of various protecting groups on both the P(III) and P(V) sides. The high reactivity of the phosphonamidite moiety will enable the condensation of a broad variety of alcohols and thiols. It is expected that the reagents introduced here are suitable for the conception of an even wider variety of pyrophosphate mimetics, and we project that selenophosphonate moieties and boranophosphonates [47] are within reach, thereby further broadening the chemical space of pyrophosphate mimetics. The drawback of the reported method is insufficient reactivity of the phosphonylphosphonate triesters in the condensation with secondary alcohols. This limitation will make the preparation of compounds in which two carbons are connected by a bisphosphonate diester bridge challenging and will need to be addressed in future studies. In addition, the synthesis of chirally pure phosphonothioates cannot be achieved with our P(III)‐P(V) methodology at the present stage of its development. Nevertheless, our method allows creating mimetics of biomolecules, as we demonstrated here for the analogs of nucleoside diphosphates, CDP‐glycerol and CDP‐ribitol. We believe that the chemistry described here can also be harnessed to generate tunable ligands for catalysis or reagents for organocatalysis.

## Conflicts of Interest

The authors declare no conflict of interest.

## Supporting Information

The detailed experimental procedures and spectroscopic characterization of the products.

The authors have cited additional references within the Supporting Information [16, 26, 34, 36, 39, 45, 48].

## Supporting information




**Supporting File**: chem71563‐sup‐0001‐SuppMat.pdf.

## Data Availability

The data that support the experimental results of this study are available in the supplementary material of this article.
