# Supplementary material for: Phosphonomethyl (Thio)Phosphonamidite Reagents for the Construction of Phosphonylmethyl Phosphono(Di)Thioate Mono‐ and Diesters
Source: Chemistry. 2026 Aug 21;32(42):e71563. doi: 10.1002/chem.71563 (PMC13755163; doi:10.1002/chem.71563)
Supplement: Supplementary file 1 [file CHEM-32-e71563-s001.pdf]

Supporting Information

Phosphonomethyl (Thio)Phosphonamidite Reagents for the Construction of Phosphonylmethyl Phosphono(di)thioate Mono- and Diesters

Pascal Balić, Roy Steneker, Gijsbert A. van der Marel, Jeroen D. C. Codée,* Dmitri V. Filippov*

Leiden Institute of Chemistry, Leiden University, Einsteinweg 55, 2333 CC Leiden, The Netherlands

*Email: jcodee@chem.leidenuniv.nl; filippov@lic.leidenuniv.nl

Table of Contents

General experimental procedures 2

Preparation of Phosphonomethyl Phosphonamidite and Thiophosphonamidite Reagents 3

Synthesis of Phosphonylmethyl Phosphono(di)thioate Monoesters 5

Synthesis of 1,2,3,4-tetra-O-acetyl-D-ribitol **24** 8

Synthesis of guanosine derivative **17** with acid-sensitive protections 9

Synthesis of Non-Symmetric Phosphonylmethyl Phosphono(di)thioate Diesters 10

Synthesis of phosphonomethyl phosphonbisamidite **10** Monitored by ^{31}P NMR 18

Synthesis of Phosphonylmethyl Thiophosphonamidites **5** and **6** by Amidophosphonitylation of HSCNE with Phosphonbisamidites **9** and **10** Monitored by ^{31}P NMR 19

P(III)-Coupling and Sulfurization of (Thio)Phosphonamidites **4** and **6** with Cytidine Derivative **14** Monitored by ^{31}P NMR 20

NMR Spectra of Phosphonomethyl (Thio)phosphonamidite Reagents 21

NMR Spectra of Phosphonylmethyl Phosphono(di)thioate Monoesters and Precursors 44

NMR Spectra for Ribitol Building Blocks 80

NMR Spectra for Guanosine Building Blocks 87

NMR Spectra of Non-Symmetric Phosphonylmethyl Phosphono(di)thioate Diesters and Precursors 91

NMR Spectra of Non-Symmetric Phosphonylmethyl β-Phosphono(di)thioate Diesters and Precursors 152

General experimental procedures

All reagents and solvents were of reagent grade from Sigma Aldrich, Acros Organics, Chem-Lab, Bio-Connect, Biosynth or Alfa Aesar and used as received unless stated otherwise. Except for bis(diisopropylamino)chlorophosphine (≥95.0% from Sigma Aldrich), which was purified prior to use by precipitating diisopropylamine hydrochloride out in *n*-hexane, 2-cyanoethanol was vacuum distilled and stored over 3Å molecular sieves, 3-mercaptopropionitrile was freshly prepared from di-(2-cyanoethyl) disulfide by reductive cleavage with Zn/HCl, as described in the literature[34], and stored at -20 °C for no longer than 2 weeks. 1,8-Diazabicyclo[5.4.0]undec-7-ene was dried by storing over 3 Å molecular sieves. Potassium *tert*-butoxide (vacuum sublimed grade 99.99%), dimethyl methylphosphonate (≥98%), 4,5-dicyanoimidazole (99.0%) and sulfur-transfer reagent 3H-1,2-benzodithiol-3-one (97%) were purchased from Sigma-Aldrich and Beaucage reagent (98%) from Alfa Aesar. Wada's phosphonium reagent was prepared as described in the literature.[45]

All reactions were performed under an argon or nitrogen atmosphere using anhydrous solvents at ambient temperature unless otherwise stated. In particular, for all reactions involving P^{III} chemistry glassware was flame-dried under high vacuum, backfilled with argon and the reactions performed under an argon atmosphere using anhydrous solvents. All nucleosides, phosphonamidites, thiophosphonamidites and bisphosphonate triesters used in P^{V} phosphonodiester condensations were rendered anhydrous by azeotropically distilling thrice with dry acetonitrile before being used for reactions. Solvents needed for anhydrous conditions were prepared one day prior to use by storing over vacuum flame-dried molecular sieves under a nitrogen atmosphere with the exception of diisopropylamine, diisopropylethylamine, triethylamine, pyridine and 2,6-lutidine, which were distilled over KOH and stored on KOH. Solvents were dried as follows: chloroform, dichloromethane, diethyl ether, tetrahydrofuran and toluene were dried over 4 Å molecular sieves, acetonitrile, methanol and hexamethylphosphoramide over 3 Å molecular sieves. *Tert*-Butanol was dried by liquifying at 25 °C and storing over 4 Å molecular sieves. Additionally, anhydrous solvents were rendered free of oxygen by purging nitrogen gas through for 30 minutes. Solvents for azeotroping were always anhydrous. Solvents for work-up and column chromatography were used as received. Phosphate buffer was prepared by dissolving 81.9 g/L Na_2HPO_4 and 66 g/L of NaH_2PO_4 in water. Methylene bisphosphonate compounds were dried using sodium sulphate. Unless stated otherwise, all solvents were removed *in vacuo* by rotary evaporation at temperatures not exceeding 50 °C. Column chromatography was performed on silica gel 60 Å (40-63 µm, Macherey-Nagel), with the exception of phosphonamidite and methylenebisphosphonate compounds, which were purified on high-purity grade silica (Sigma-Aldrich, Davisil Grade, 633). The high-purity silica gel can be regenerated after use by flushing with four column volumes of methanol and two column volumes of diethyl ether, followed by drying under high vacuum at 100 °C.

Reaction progression for amidophosphonitylations, phosphonitylations, phosphonodiester condensations and deblocking of phosphonates was monitored by virtue of ^{31}P NMR and, unless stated otherwise, exclusively recorded on a Bruker WM-300 spectrometer (^{31}P 122 MHz) by charging an NMR tube with an aliquot of the reaction mixture and fitting with an airtight acetone-d_6 capillary required for locking. Otherwise, reactions were monitored by TLC-analysis using Macherey-Nagel aluminium sheets (silica gel 60 F254) with detection by UV-absorption (254/366 nm) or by one of following treatments: (a) spraying with $(NH_4)_6Mo_7O_{24}{\bullet}4H_2O$ (25 g/L) and $(NH_4)_4Ce(SO_4)_4{\bullet}2H_2O$ (10 g/L) in 10% aqueous sulfuric acid or (b) spraying with a solution of $KMnO_4$ (5 g/L) in 4% aqueous K_2CO_3 followed by charring at approximately 250 °C. TLC sheets for P^{III} compounds were neutralized prior to use with triethylamine. LC-MS analysis was performed on an LCQ Adventage Max (Thermo Finnigan) ion-trap spectrometer (ESI+) coupled to a Surveyor HPLC system (Thermo Finnigan) equipped with a C18 column (Gemini, 4.6 mm x 50 mm, 5 µm particle size, Phenomenex) in combination with buffers A: H2O, B: ACN and C: 1% aq. TFA.

Purification by size-exclusion chromatography (SEC) was performed *via* isocratic elution (1 mL/min) with an aqueous 0.15 NH_4OAc solution and 10% acetonitrile buffer system over an HW-40-S resin (16 x 600 mm) from TOYOPEARL. Purification by Ion Exchange Chromatography was done on an ÄKTA pure system equipped with a Source-15Q column (16 x 100 mm, 4 mL/min). Purified me-NDP analogues were rendered free of NH_4OAc by azeotroping thrice with acetonitrile/water (v/v; 1/1) and lyophilized.

NMR spectra were recorded using a Bruker WM-300 ($^{1}H/^{13}C/^{31}P$ 300 MHz/75 MHz/122 MHz), Bruker AV-400 ($^{1}H/^{13}C/^{31}P$ 400 MHz/101 MHz/162 MHz) or a Bruker AV-500 spectrometer ($^{1}H/^{13}C/^{31}P$ 500 MHz/125 MHz/202MHz). Chemical shifts (δ) are given in ppm relative to TMS for $CDCl_3$ as an internal standard or the residual solvent signal for $CDCl_3$ and D_2O. Chemical shifts for ^{31}P spectra are indirectly referenced to H_3PO_4 (0.00 ppm) according to the IUPAC method. $CDCl_3$ utilized for the characterization of phosphonamidite-containing compounds was neutralized prior to use by filtering over aluminum oxide (Type WB-5: Basic). Coupling constants (J) are given in Hz. ^{13}C spectra are proton-decoupled and acquired with an attached proton test (APT). ^{31}P spectra are proton-decoupled unless otherwise stated, in which case ^{31}P denotes proton-coupled and $^{31}P\{^{1}H\}$ proton-decoupled spectra, respectively. Structural elucidations are based on 2D-NMR COSY, HSQC, NOESY and HMBC experiments. The ratio of P-diastereomeric mixtures was determined by integrating representative signals in ^{1}H and ^{31}P NMR spectra.

High-resolution mass spectra (HRMS) were recorded by direct injection of 2 µL of a 1 µM solution in water/acetonitrile (v/v; 1/1 and 0.1% formic acid) on a LTQ Orbitrap (Thermo Finnigan) mass spectrometer equipped with an electrospray ion source in positive mode (source voltage 3.5kV, sheath gas flow 10, capillary temperature 250 °C) with resolution R = 6000 at m/z = 400 (mass range m/z 150-4000) and dioctylpthalate (m/z = 391.28428) as a 'lock mass'. HRMS samples were prepared in either acetonitrile, Milli-Q® or dioxane with an approximate concentration of 1 mM. HRMS samples for P^{III} compounds were prepared freshly in anhydrous acetonitrile.

Preparation of Phosphonomethyl Phosphonamidite and Thiophosphonamidite Reagents

Di-*tert*-butyl ((bis(diisopropylamino)phosphanyl)methyl)phosphonate[16] **(9)** – To a cooled solution (-78 °C) of diisopropylamine (1.47 mL, 10.5 mmol, 2.1 eq.) in tetrahydrofuran (5 mL) under argon was added *n*-butyllithium (2.5 M in hexanes, 4.2 mL, 10.5 mmol, 2.1 eq.). After stirring for 30 minutes, di-*tert*-butyl methylphosphonate **7**[48] (1.04 g, 5.0 mmol, 1.0 eq.) in tetrahydrofuran (5 mL), dried prior by azeotroping twice with dioxane, was added dropwise (± 10 minutes) to the flask and stirring was continued for 15 minutes. Lastly, bis(diisopropylamino)chlorophosphine (1.6 g, 6.0 mmol, 1.2 eq.), purified as described in the general section, was added dropwise as a solution in *n*-hexane/THF (1/1; v/v, 10 mL, total volume 20 mL, 0.25 M) over the duration of 10 minutes through a syringe fitted with a silica filter and a layer of freshly activated 4 Å molecular sieves (1 - 2 cm) to the pale phosphonolithiate solution formed from **20** as described above. Cooling was removed, and the reaction was stirred for 4 hours while gradually warming to ambient temperature. The resulting turbid mixture was poured into sat. aq. $NaHCO_3$, extracted with EtOAc and the organic layer dried over Na_2SO_4, filtered and concentrated *in vacuo*. Purification of the crude yellow oil by silica gel column chromatography (5 → 10 → 15 → 20% EtOAc in pentane with 2% Et_3N) afforded title phosphonbisamidite **9** as a colourless oil that crystallized into a white solid (1.76 g, 4.01 mmol, 80%). Spectral data was in concordance with that of literary precedence. **^{1}H NMR** (500 MHz, $CDCl_3$) δ 3.38 (dhept, 4H, $^3J_{HP}$ = 11.0, J_{HH} = 6.7 Hz, CH ^{i}Pr), 2.12 (dd, 2H, $^2J_{HP}$ = 20.7, 1.6 Hz, P-CH_2-P), 1.52 (s, 18H, CH_3 ^{t}Bu), 1.19 (d, 12H, J = 6.7 Hz, CH_3 ^{i}Pr) 1.17 (d, 12H, J = 6.7 Hz, CH_3 ^{i}Pr); **^{13}C NMR** (126 MHz, $CDCl_3$) δ 81.3 (d, $^2J_{CP}$ = 8.5 Hz, C_q ^{t}Bu), 47.1 (d, $^2J_{CP}$ = 11.9 Hz, CH ^{i}Pr), 31.7 (dd, $^1J_{CP}$ = 138.7, 30.7 Hz, P-C_α-P), 30.6 (d, $^3J_{CP}$ = 4.0 Hz, Me ^{t}Bu), 24.3 (d, $^3J_{CP}$ = 6.2 Hz, Me ^{i}Pr), 24.0 (d, $^3J_{CP}$ = 8.4 Hz, Me ^{i}Pr); **^{31}P{^{1}H} NMR** (202 MHz, $CDCl_3$) δ 40.68 (d, J = 88.9 Hz, P^{III}), 22.68 (d, J = 88.8 Hz, P^V); **^{31}P NMR** (202 MHz, $CDCl_3$) δ 40.68 (dp, J_{PP} = 88.9, $^3J_{PH}$ = 10.9 Hz), 22.68 (dt, J_{PP} = 88.8, $^2J_{PH}$ = 20.7 Hz); **HRMS** (ESI) m/z: $[M+H+H_2O]^+$ calcd for $C_{21}H_{51}N_2O_4P_2$ 457.3319, found 457.3313.

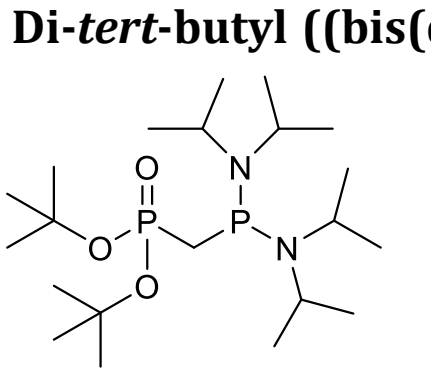

(*R*P/*S*P)-*Tert*-butylmethyl((bis(diisopropylamino)phosphanyl)methyl)phosphonate (10) - A 50 mL desiccated round-bottom flask was desiccated repeatedly by flame-drying under high vacuum, backfilled with argon and charged with tetrahydrofuran (5.0 mL), diisopropylamine (1.47 mL, 10.5 mmol, 2.1 eq.) and hexamethylphosphoramide (1.83 mL, 10.5 mmol, 2.1 eq.). The solution was cooled to -78 °C, whereupon *n*-butyllithium (2.5 M in hexanes, 4.2 mL, 10.5 mmol, 2.1 eq.) was added (± 5 min) to the flask. After 30 minutes, a solution of *tert*-butylmethyl methylphosphonate **8**[26] (831 mg, 5.0 mmol, 1.0 eq.) in anhydrous tetrahydrofuran (5 mL), rendered dry prior by azeotroping thrice with anhydrous dioxane, was slowly added (± 5 min) and stirring continued for 30 minutes, affording a colourless phosphonolithiate solution. Hereto, bis(diisopropylamino)chlorophosphine (1.6 g, 6.0 mmol, 1.2 eq.), purified prior by precipitating diisopropylamine hydrochloride out in *n*-hexane (5 mL), was added dropwise over the duration of 10 minutes as a solution in *n*-hexane/THF (1/1; v/v, 10 mL, total volume 20 mL, 0.25 M) through a syringe fitted with a silica filter and a layer of freshly activated 4 Å molecular sieves (1 - 2 cm). Stirring at -78 °C was maintained for 30 minutes, the mixture was quenched by the addition of EtOH (1 mL), and poured into a separatory funnel containing sat. aq. $NaHCO_3$ and extracted with EtOAc. The organic layer was dried over Na_2SO_4, filtered and concentrated *in vacuo* to yield a crude pale oil. Purification by silica gel column chromatography (5 → 10 → 15 → 20% EtOAc in pentane with 2% Et_3N) furnished title phosphonbisamidite **10** as a colourless oil that slowly crystallized into a white solid (1.37 g, 3.45 mmol, 69%). **^{1}H NMR** (300 MHz, $CDCl_3$) δ 3.71 (d, 3H, $^3J_{HP}$ = 11.3 Hz, P-OCH_3), 3.48 – 3.29 (br. dhept, 4H, $^3J_{HP}$ = 11.4, J_{HH} = 6.6 Hz, CH ^{i}Pr), 2.30 (ddd, 1H, $^2J_{HP}$ = 20.8, 2.8, J_{gem} = 14.7 Hz, P-CH*<u>H'</u>*-P), 2.11 (ddd, 1H, $^2J_{HP}$ = 21.4, 2.4, J_{gem} = 14.7 Hz, P-C*<u>H</u>*H'-P), 1.52 (s, 9H, CH_3 ^{t}Bu), 1.20 (d, 12H, J = 6.6 Hz, CH_3 ^{i}Pr), 1.16, 1.15 (2 x d, 12H, J = 6.7 Hz, CH_3 ^{i}Pr); **^{13}C NMR** (126 MHz, $CDCl_3$) δ 81.6 (d, $^2J_{CP}$ = 8.8 Hz, C_q ^{t}Bu), 51.6 (dd, $^2J_{CP}$ = 6.6, $^4J_{CP}$ = 3.1 Hz, P-OMe), 46.95 (d, $^2J_{CP}$ = 11.8 Hz, CH ^{i}Pr), 46.91 (d, $^2J_{CP}$ = 11.9 Hz, CH ^{i}Pr), 30.3 (d, $^3J_{CP}$ = 4.0 Hz, Me ^{t}Bu), 28.6 (dd, $^1J_{CP}$ = 136.4, 31.3 Hz, P-C_α-P), 24.02 (d, $^3J_{CP}$ = 6.5 Hz, Me ^{i}Pr), 23.99 (d, $^3J_{CP}$ = 6.2 Hz, Me ^{i}Pr), 23.8, 23.73, 23.71, 23.65 (app. $^3J_{CP}$ = 8.3, 7.9 Hz, Me ^{i}Pr); **^{31}P{^{1}H} NMR** (202 MHz, $CDCl_3$) δ 38.97 (d, J = 83.9 Hz, P^{III}), 27.74 (d, J = 83.9 Hz, P^V); **^{31}P NMR** (202 MHz, $CDCl_3$) δ 38.95 (dp, J_{PP} = 83.7, $^3J_{PH}$ = 11.5 Hz), 27.74 (dtq, J_{PP} = 83.7, $^2J_{PH}$ = 21.3, $^3J_{PH}$ = 10.8 Hz); **HRMS** (ESI) m/z: $[M+H+H_2O]^+$ calcd for $C_{18}H_{45}N_2O_4P_2$ 415.2849, found 415.2846.

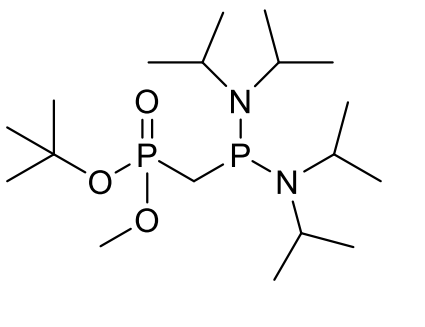

Di-*tert*-butyl (*R*P/*S*P)-(((2-cyanoethoxy)(diisopropylamino)phosphanyl)methyl) phosphonate (2) - To a dry solution of phosphonbisamidite **9** (740 mg, 1.69 mmol) and 2-cyanoethanol (115.2 µL, 1.69 mmol, 1.0 eq.) in anhydrous dichloromethane (11 mL, 0.15 M) was added 4,5-dicyanoimidazole (119 mg, 1.01 mmol, 0.6 eq.). After 30 minutes the volatiles were removed *in vacuo* and the crude yellow residue was chromatographed (20 → 30 → 40 → 50% EtOAc in pentane with 1% Et_3N) to furnish title phosphonamidite **2** as a colourless oil (609 mg, 1.49 mmol, 88%). NMR spectra were identical with those published previously. **^{1}H NMR** (500 MHz, $CDCl_3$) δ 3.89 – 3.77 (m, 2H, OCH_2 CNE), 3.55 – 3.44 (br. dhept, 2H, app. $^2J_{HP}$ = 9.5, J_{HH} = 6.7 Hz, CH ^{i}Pr), 2.70 – 2.59 (m, 2H, CH_2CN), 2.30 (ddd, 1H, $^2J_{HP}$ = 19.0, 1.3, J_{gem} = 14.5, P-C*<u>H</u>*H'-P), 1.89 (ddd, 1H, $^2J_{HP}$ = 19.0, 1.2, J_{gem} = 14.5 Hz, P-CH*<u>H'</u>*-P), 1.52 (s, 9H, CH_3 ^{t}Bu), 1.51 (s, 9H, CH_3 ^{t}Bu), 1.21 (d, 6H, J = 6.8 Hz, CH_3 ^{i}Pr), 1.14 (d, 6H, J = 6.9 Hz, CH_3 ^{i}Pr); **^{13}C NMR** (126 MHz, $CDCl_3$) δ 117.7 (C_q CNE), 81.68 (d, $^2J_{CP}$ = 8.4 Hz, C_q ^{t}Bu), 81.64 (d, $^2J_{CP}$ = 8.4 Hz, C_q ^{t}Bu), 61.6 (d, $^2J_{CP}$ = 24.9 Hz, OCH_2 CNE), 44.9, 44.8(CH ^{i}Pr), 35.4 (dd, $^1J_{CP}$ = 138.8, 31.1 Hz, P-C_α-P), 30.3 (d, $^3J_{CP}$ = 3.9 Hz, Me ^{t}Bu), 24.08, 24.05 (Me ^{i}Pr), 20.2 (d, $^3J_{CP}$ = 8.5 Hz, CH_2CN); **^{31}P NMR (**202 MHz, $CDCl_3$) δ 122.53 (d, J = 60.2 Hz, P^{III}), 19.77 (d, J = 60.3 Hz, P^V); **^{31}P{^{1}H} NMR** (202 MHz, $CDCl_3$) δ 122.53 (dp, J_{PP} = 60.2, J_{PH} = 9.2 Hz), 19.77 (dt, J_{PP} = 60.3, $^2J_{PH}$ = 19.6 Hz); **HRMS** (ESI) m/z: $[M+H+H_2O]^+$ calcd for $C_{18}H_{41}N_2O_5P_2$ 427.2485, found 427.2482.

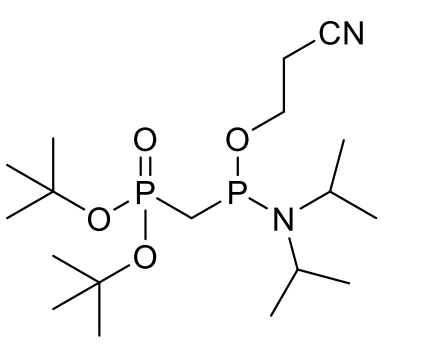

Di-*tert*-butyl (*R*P/*S*P)-(((*tert*-butoxy)diisopropylamino)phosphanyl)methyl)phosphonate (3) – A dry solution of phosphondiamidite **9** (439 mg, 1.0 mmol, 1.0 eq.) in dichloromethane (5 mL, 0.2 M) was prepared, whereto *tert*-butanol (95.0 µL, 1.0 mmol, 1.0 eq) and 4,5-dicyanoimidazole (71 mg, 0.6 mmol, 0.6 eq.) were added consecutively. After 2 hours the solvent was removed *in vacuo*. Purification by silica gel column chromatography (0 → 2.5 → 5 → 10 → 15 → 20 → 30% EtOAc in pentane with 1% Et_3N) afforded phosphonamidite **3** as a pale colourless oil (218 mg, 0.53 mmol, 53%). **^{1}H NMR** (500 MHz, $CDCl_3$) δ 3.55 – 3.33 (br. dhept, 2H, $^2J_{HP}$ = 9.8, J_{HH} = 6.7 Hz, CH ^{i}Pr), 2.12 (ddd, 1H, $^2J_{HP}$ = 19.2, 2.9, J_{gem} = 14.5 Hz, P-CH*H'*-P), 1.82 (ddd, 1H, $^2J_{HP}$ = 19.8, 4.1, J_{gem} = 14.4 Hz, P-C*H*H'-P), 1.46 (2 x s, 18H, CH_3 ^{t}Bu), 1.26 (s, 9H, CH_3 ^{t}Bu), 1.13 (d, 6H, J = 6.7 Hz, CH_3 ^{i}Pr), 1.07 (d, 6H, J = 6.8 Hz, CH_3 ^{i}Pr); **^{13}C NMR** (126 MHz, $CDCl_3$) δ 81.4 (d, $^2J_{CP}$ = 8.6 Hz, C_q ^{t}Bu), 81.3 (d, $^2J_{CP}$ = 8.1 Hz, C_q ^{t}Bu), 75.3 (d, $^2J_{CP}$ = 13.4 Hz, C_q ^{t}Bu), 44.8, 44.7 (CH ^{i}Pr), 37.8 (dd, $^1J_{CP}$ = 136.9, 35.3 Hz, P-C_α-P), 30.7, 30.64, 30.63, 30.60 (Me ^{t}Bu), 30.5 (d, $^3J_{CP}$ = 4.3 Hz, Me ^{t}Bu), 23.6, 23.5 (Me ^{i}Pr); **^{31}P{^{1}H} NMR** (202 MHz, $CDCl_3$) δ 103.20 (d, J = 53.5 Hz, P^{III}), 21.33 (d, J = 53.4 Hz, P^V); **^{31}P NMR** (202 MHz, $CDCl_3$) δ 103.20 (br. dt, J_{PP} = 53.8 Hz, $^2J_{PH}$ = 10.0 Hz), 21.33 (dt, J_{PP} = 53.8, $^2J_{HP}$ = 19.4 Hz); **HRMS** (ESI) m/z: $[M+H+H_2O]^+$ calcd for $C_{19}H_{46}NO_5P_2$ 430.2846, found 430.2855.

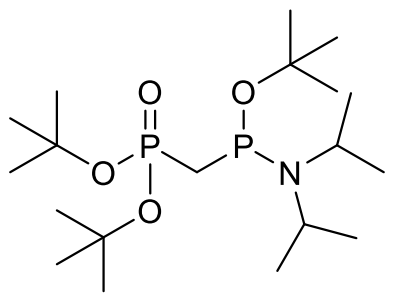

(*R*P/*S*P)-*Tert*-butylmethyl-(*R*P/*S*P)-(((2-cyanoethoxy)diisopropylamino)phosphanyl)methyl) phosphonate (4) – To a dry solution of phosphonbisamidite **10** (397 mg, 1.0 mmol, 1.0 eq.) and 3-hydroxypropionitrile (68.4 µL, 1.0 mmol, 1.0 eq) in anhydrous dichloromethane (6.7 mL, 0.15 M) was added a catalytic amount of 4,5-dicyanoimidazole (701 mg, 0.6 mmol, 0.6 eq.). After 30 minutes the volatiles were evaporated *in vacuo* to afford a crude colourless residue. Purification *via* silica gel column chromatography (20 → 30 → 40 → 50% EtOAc in pentane with 1% Et_3N) furnished phosphonamidite **10** as a colourless oil (5:4 mixture of P-diastereomers, 342 mg, 0.933 mmol, 93%). *Resonances and J-couplings marked with an asterisk* correspond to the minor P-diastereomer*. **^{1}H NMR** (300 MHz, $CDCl_3$) δ 3.90 – 3.76 (m, 2H, OCH_2 CNE), 3.73*, 3.71 (2 x d, 3H, $^3J_{HP}$ = 11.3, 11.3* Hz, P-OCH_3), 3.51 (br. dhept, 2H, $^2J_{HP}$ = 10.3, J_{HH} = 6.6 Hz, CH ^{i}Pr), 2.74 – 2.56 (m, 2H, CH_2CN), 2.40*, 2.36 (2 x ddd, 1H, $^2J_{HP}$ = 19.4*, 19.7, 3.0*, 2.7, J_{gem} = 14.6*, 14.5 Hz, P-CH*H'*), 1.95*, 1.91 (2 x ddd, 1H, $^2J_{HP}$ = 20.3*, 20.7, 3.2*, 2.7, J_{gem} 14.7*, 14.5 Hz, P-C*H*H'-P), 1.52 (s, 9H, CH_3 ^{t}Bu), 1.21 (d, 6H, J = 6.7 Hz, CH_3 ^{i}Pr), 1.14, 1.14* (2 x d, 6H, J = 6.8, J^* = 6.8 Hz, CH_3 ^{i}Pr); **^{13}C NMR** (75 MHz, $CDCl_3$) δ 117.7 (C_q CNE), 82.27*, 82.25 (2 x d, J^* = 8.5, $^2J_{CP}$ = 8.6 Hz, C_q ^{t}Bu), 61.62*, 61.56 (2 x d, J^* = 25.0, $^2J_{CP}$ = 24.8 Hz, OCH_2 CNE), 51.67*, 51.64 (2 x app. d, $^2J_{CP}$ = 6.4, 6.4* Hz, P-OMe), 44.78, 44.64 (CH ^{i}Pr), 32.1*, 31.9 (2 x dd, $^1J_{CP}$ = 136.7*, 31.0*, 136.8, 30.9 Hz, P-C_α-P), 30.11 (d, $^3J_{CP}$ = 4.0 Hz, Me ^{t}Bu), 23.95, 23.85 (br. signals, Me ^{i}Pr), 20.00, 19.97* (2 x d, $^3J_{CP}$ = 8.6, 8.4* Hz, CH_2CN); **^{31}P{^{1}H} NMR** (122 MHz, $CDCl_3$) δ 120.27 (d, J = 58.2 Hz), 25.36* (d, J = 58.5 Hz), 25.04 (d, J = 58.1 Hz); **^{31}P NMR** (122 MHz, $CDCl_3$) δ 120.27 (dp, J_{PP} = 58.3, $^2J_{PH}$ = 9.9 Hz), 25.15 (dtq, J_{PP} = 57.5, $^2J_{PH}$ = 20.2, $^3J_{PH}$ = 10.4 Hz); **HRMS** (ESI) m/z: $[M+H+H_2O]^+$ calcd for $C_{15}H_{35}N_2O_5P_2$ 385.2016, found 385.2013.

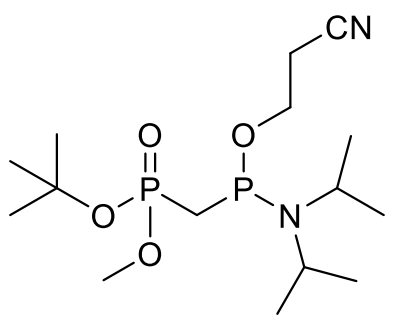

Di-*tert*-butyl (*R*P/*S*P)-(((2-cyanoethyl)thio)(diisopropylamino)phosphanyl)methyl)phosphonate (5) – To a dry solution of phosphonbisamidite **9** (439 mg, 1.0 mmol, 1.0 eq.) and 3-mercaptopropionitrile (81.5 µL, 1.0 mmol, 1.0 eq.) in anhydrous dichloromethane (6.7 mL, 0.15 M) was added 4,5-dicyanoimidazole (71 mg, 0.6 mmol, 0.6 eq.). Reaction progression was monitored with ^{31}P NMR, which indicated full conversion after 30 minutes and the volatiles were removed under reduced pressure to afford a crude colourless oil. Purification by flash column chromatography (0 → 2.5 → 5 → 10 → 15 → 20% EtOAc in pentane with 1% TEA) afforded title thiophosphonamidite **5** as a malodorous colourless oil (392 mg, 0.92 mmol, 92%). **^{1}H NMR** (500 MHz, $CDCl_3$) δ 3.51 – 3.36 (br. dhept, 2H, $^2J_{HP}$ = 10.2, J_{HH} = 6.5 Hz, CH_3 ^{i}Pr), 2.93 – 2.67 (m, 4H, SCNE), 2.36 (ddd, 1H, $^2J_{HP}$ = 18.8, 2.2, J_{gem} = 14.9, P-C*H*H'-P), 2.20 (dd, $^2J_{HP}$ = 19.5, J_{gem} = 14.9 Hz, P-CH*H'*-P), 1.52 (s, 18H, CH_3 ^{t}Bu), 1.19 (d, 6H, J = 6.6 Hz, CH_3 ^{i}Pr), 1.17 (d, 6H, J = 6.7 Hz, CH_3 ^{i}Pr); **^{13}C NMR** (126 MHz, $CDCl_3$) δ 118.4 (C_q SCNE), 82.3 (d, $^2J_{CP}$ = 8.6 Hz, C_q ^{t}Bu), 82.1 (d, $^2J_{CP}$ = 8.3 Hz, C_q ^{t}Bu), 47.9 (*signal verified with HSQC,* CH ^{i}Pr), 34.3 (dd, $^1J_{CP}$ = 139.1, 32.9 Hz, P-C_α-P), 30.4 (d, $^3J_{CP}$ = 4.1 Hz, Me ^{t}Bu), 30.3 (d, $^3J_{CP}$ = 4.0 Hz, Me ^{t}Bu), 27.1 (d, $^2J_{CP}$ = 25.7 Hz, SCH_2 CNE), 23.9 (br. s, Me ^{i}Pr), 19.9 (d, $^3J_{CP}$ = 6.2 Hz, CH_2 CNE); **^{31}P{^{1}H} NMR** (202 MHz, $CDCl_3$) δ 70.03 (d, J = 69.8 Hz, P^{III}), 17.78 (d, J = 69.7 Hz, P^V); **^{31}P NMR**(202 MHz, $CDCl_3$) δ 70.03 (dp, J_{PP} = 69.9, $^2J_{PH}$ = 10.2 Hz), 18.12 (dt, J_{PP} = 69.8, $^2J_{PH}$ = 19.2 Hz); **HRMS** (ESI) m/z: $[M+H+H_2O]^+$ calcd for $C_{18}H_{41}N_2O_4P_2S$ 443.2257, found 443.2270.

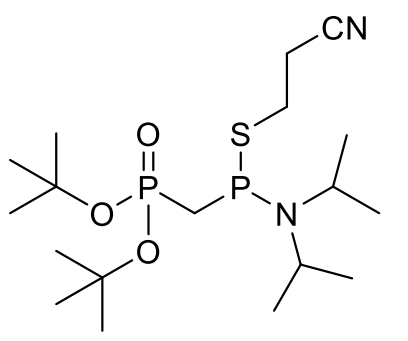

(*R*P/*S*P)-*Tert*-butylmethyl-(*R*P/*S*P)-(((2-cyanoethyl)thio)(diisopropylamino)phosphanyl)methyl)phosphonate (6) – To a dry solution of phosphonbisamidite **10** (397 mg, 1.0 mmol, 1.0 eq.) and 3-mercaptopropionitrile (81.4 µL, 1.0 mmol, 1.0 eq.) in anhydrous dichloromethane (6.7 mL, 0.15M) was added 4,5-dicyanoimidazole (71 mg, 0.6 mmol, 0.6 eq.). After 30 minutes the volatiles were removed *in vacuo* to furnish a crude colourless oil. Purification by silica gel column chromatography (10 → 15 → 20 → 25 → 30 → 40% EtOAc in pentane with 1% Et_3N) afforded a diastereomeric mixture thiophosphonamidite **6** as a malodorous colourless oil (10:7 mixture of P-diastereomers, 362 mg, 0.95 mmol, 95%). *Resonances marked with an asterisk * correspond to the minor P-diastereomer.* **^{1}H NMR** (400 MHz, $CDCl_3$) δ 3.72 (d, 3H, $^3J_{HP}$ = 11.3 Hz, P-OCH_3), 3.52 – 3.35 (m, 2H, CH ^{i}Pr), 2.96 – 2.83 (m, 2H, SCH_2), 2.83 – 2.67 (m, 2H, CH_2CN), 2.51 – 2.18 (m, 2H, P-CH_2-P), 1.52 (s, 9H, CH_3 ^{t}Bu), 1.19 (d, 6H, J = 6.7 Hz, CH_3 ^{i}Pr), 1.17*, 1.16 (2 x d, 6H, J = 6.8*, 6.9 Hz, CH_3 ^{i}Pr); **^{13}C NMR** (101 MHz, $CDCl_3$) δ 118.14, 118.11* (C_q SCNE), 82.7, 82.5* (2 x d, $^2J_{CP}$ = 8.9, 8.7* Hz, C_q ^{t}Bu), 51.87, 51.81, 51.78, 51.72 (P-OMe), 44.1 (d, $^2J_{CP}$ = 5.6 Hz, CH ^{i}Pr), 31.03, 30.99* (2 x dd, $^1J_{CP}$ = 137.8, 137.5* 32.5, 32.2* Hz, P-C_α-P), 30.08, 30.04* (2 x d, $^3J_{CP}$ = 3.9, 3.9* Hz, Me ^{t}Bu), 26.88, 26.86* (2 x d, $^2J_{CP}$ = 26.8, 26.1* Hz, SCH_2 CNE), 23.6 (br. signal, CH_3 ^{i}Pr) 19.72*, 19.68 (2 x d, J^* = 6.1, $^3J_{CP}$ = 6.3 Hz, CH_2CN); **^{31}P NMR** (162 MHz,

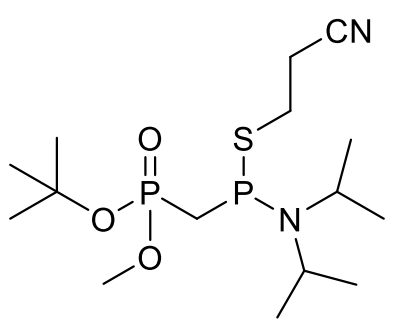

$CDCl_3$) δ 67.98 (d, *J* = 70.6 Hz), 67.94* (d, *J* = 68.9 Hz, P^{III}), 23.82 (d, *J* = 70.5 Hz), 23.50* (d, *J* = 68.8 Hz). **HRMS** (ESI) m/z: $[M+H+H_2O]^+$ calcd for $C_{15}H_{35}N_2O_4P_2S$ 401.1787, found 401.1795.

Synthesis of Phosphonylmethyl Phosphono(di)thioate Monoesters

***N*⁶-benzoyl-2',3'-di-*O*-*iso*-butyryl-di-*tert*-butyl-(((*R*P/*S*P)-cyanoethoxy)thiophosphoryl)methyl) phosphonate adenosine (12a) -** A dry solution of phosphonamidite **2** (41 mg, 0.1 mmol, 1.0 eq.) and adenosine derivative **11**[36] (56 mg, 0.11 mmol, 1.1 eq.) in anhydrous acetonitrile (0.67 mL, 0.15 M) was treated with 4,5-dicyanoimidazole (14 mg, 0.12 mmol, 1.2 eq.) for 30 minutes, followed by the addition of Beaucage reagent (24 mg, 0.12 mmol, 1.2 eq.). The resulting yellow mixture was stirred until complete sulfurization (30 minutes), poured into sat. aq. $NaHCO_3$ and extracted with EtOAc. The organic layer was washed with sat. aq. NaCl, dried over Na_2SO_4, filtered and concentrated *in vacuo*. Purification by silica gel column chromatography (0 → 5 → 10 → 15 → 20 → 30% acetone in dichloromethane) afforded me-ADP-αS tetraester **12a** as a crystalline white solid (5:4 of P-diastereomers, 78 mg, 91.7 µmol, 92%) with inseparable by column chromatography nucleoside **11** (~ 30%). *The nucleoside can be separated by size-exclusion chromatography after subsequent steps. Alternatively, the phosphonamidite can be used in excess. Resonances and J-couplings marked with an asterisk * correspond to the minor P-diastereomer.* **¹H NMR** (500 MHz, $CDCl_3$) δ 9.34 (br. s, 1H, NH), 8.79, 8.79* (2 x s, H-2), 8.50, 8.46* (2 x s, 1H, H-8), 8.02 (app. d, 2H, *J* = 7.6 Hz, *o*-Bz), 7.62 (app. t, *J* = 7.4 Hz, *p*-Bz), 7.52 (app. t, *J* = 7.8 Hz, *m*-Bz), 6.33, 6.29* (2 x d, 1H, *J* = 5.9, 5.8* Hz, H-1'), 5.94*, 5.86 (2 x t, 1H, *J* = 5.7*, 5.8 Hz, H-2'), 5.73 – 5.68 (m, 1H, H-3'), 5.66* (dd, *J* = 5.6, 3.8 Hz, H-3'), 4.57 – 4.39 (m, 3H, H-4', H-5'), 4.38 – 4.25 (m, 2H, OCH_2 CNE), 2.82 – 2.70 (m, 2H, P-CH_2-P), 2.70 – 2.60 (m, 1H, CH *i*Bu), 2.55 (hept, 1H, *J* = 7.0 Hz, CH *i*Bu), 1.52 (s, 18H, CH_3 ᵗBu), 1.26 – 1.20 (m, 6H, CH_3 *i*Bu), 1.15 – 1.05 (m, 6H, CH_3 *i*Bu); **¹³C NMR** (126 MHz, $CDCl_3$) δ 175.59, 175.56, 175.4 (C=O *i*Bu), 165.2 (C=O Bz), 152.7 (C-2), 152.0, 151.9 (C-4), 149.6, 149.5 (C-6), 142.2, 142.1, (C-8), 133.3 (*p*-Bz), 133.1 (*ipso*-Bz), 129.0 (*m*-Bz), 127.9 (*o*-Bz), 123.24, 123.18 (C-5), 116.9, 116.8 (C_q CNE), 86.3*, 86.1 (C-1'), 84.7, 84.6 (C_q ᵗBu), 81.9, 81.6* (2 x d, $^3J_{CP}$ = 8.1, 8.6* Hz, C-4'), 73.3, 73.1* (C-2'), 70.5*, 70.4 (C-3'), 65.7, 65.3* (2 x d, $^2J_{CP}$ = 5.6, 6.2* Hz, C-5'), 61.6*, 61.5 (2 x d, $^2J_{CP}$ = 5.2* 5.4 Hz, OCH_2 CNE), 38.4 – 36.7 (m, *verified with HSQC*, P-C_α-P), 33.9, 33.6 (CH *i*Bu), 30.4, 30.3 (Me ᵗBu), 19.68, 19.62* (2 x d, $^3J_{CP}$ = 6.5, 6.8* Hz, CH_2CN), 19.0, 18.9, 18.82, 18.77 (Me *i*Bu), 18.65 (d, $^3J_{CP}$ = 4.0 Hz, Me *i*Bu); **³¹P NMR** (202 MHz, $CDCl_3$) δ 88.61* (d, *J* = 6.8 Hz), 88.55 (d, *J* = 6.2 Hz, PS), 7.77 (d, *J* = 6.2 Hz), 7.67* (d, *J* = 6.7 Hz, PO); **HRMS** ESI (m/z): $[M+H]^+$ calcd for $C_{37}H_{53}N_6O_{11}P_2S$ 851.2963, found 851.2965.

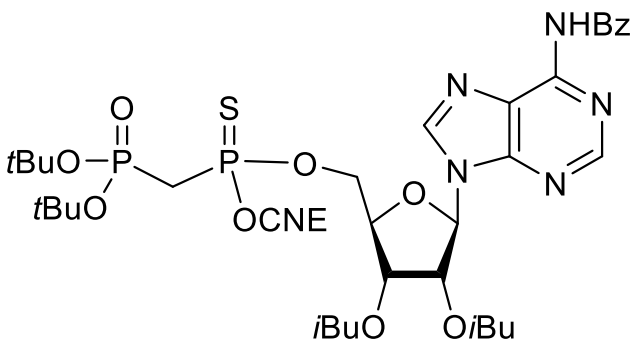

***N*⁶-benzoyl-2',3'-di-*O*-*iso*-butyryl-di-*tert*-butyl-(((*R*P/*S*P)-3-mercaptopropionitrile)thiophosphoryl)methyl)phosphonate adenosine (12b) –** Thiophosphonamidite **5** (85 mg, 0.2 mmol, 1.0 eq.) and adenosine derivative **11**[36] (113 mg, 0.22 mmol, 1.1 eq.) were dried by azeotroping thrice with acetonitrile and dissolved in anhydrous acetonitrile (1 mL, 0.2 M). Hereto was added 4,5-dicyanoimidazole (35 mg, 0.3 mmol, 1.5 eq.) followed by treatment with Beaucage reagent (48.1 mg, 0.24 mmol, 1.2 eq.) after 30 minutes. The pale yellow solution stirred for 20 minutes, diluted with EtOAc, washed successively with sat. aq. $NaHCO_3$ and sat. aq. NaCl and the resulting organic layer was dried over Na_2SO_4, filtered and concentrated *in vacuo*. Purification by silica gel column chromatography (0 → 2.5 → 5 → 10 → 15 → 20% acetone in dichloromethane) afforded a white crystalline foam of title me-ADP-αS_2 tetraester **12b** as an inconsequential mixture of diastereomers (5:4 mixture of P-diastereomers,161 mg, 0.186 mmol, 93%) with residual (< 15%), inseparable by column chromatography nucleoside **11**. *Purification by size-exclusion chromatography or using excess thiophosphonamidite may mitigate the nucleoside impurity*. *Resonances and J-couplings marked with an asterisk * correspond to the minor P-diastereomer.* **¹H NMR** (500 MHz, $CDCl_3$) δ 9.48 (br. s, 1H, 6-NH), 8.80 (s, 1H, H-2), 8.64*, 8.58 (2 x s, 1H, H-8), 8.02 (app. d. 2H, *J* = 7.8 Hz, *o*-Bz), 7.50 (app. tt, 1H, *J* = 7.4, 1.8, 1.0 Hz, *p*-Bz), 7.50 (app. tt, 2H, *J* = 7.5, 1.6 Hz, *m*-Bz), 6.30 (d, 1H, *J* = 6.1 Hz, H-1'), 6.02*, 5.94 (2 x t, 1H, *J* = 6.1*, 5.8 Hz, H-2'), 5.72*, 5.68 (2 x dd, 1H, *J* = 5.9*, 5.6, 3.9*, 3.9 Hz, H-3'), 4.55 – 4.42 (m, 2H, H-4', H-5_A'), 4.41 – 4.34* (m, 1H, H-4'*, H-5_B'), 4.29* (app. ddd, *J* = 11.1, 8.5, 2.6 Hz, 1H, H-5''), 3.32 – 3.15 (m, 2H, SCH_2 CNE), 3.07 – 2.92 (m, 2H, P-CH_2-P), 2.78 (app. t, 2H, *J* = 7.0 Hz, CH_2CN), 2.69 – 2.61 (m, 1H, *J* = 7.0 Hz, CH *i*Bu), 2.54 (hept, 1H, *J* = 7.0 Hz, CH *i*Bu), 1.54 – 1.51 (m, 18H, CH_3 ᵗBu), 1.25 – 1.21 (m, 6H, CH_3 *i*Bu), 1.14, 1.13, 1.11, 1.10 (4 x d, 6H, *J* = 7.0, 7.0, 7.0, 6.9 Hz, CH_3 *i*Bu); **¹³C NMR** (126 MHz, $CDCl_3$) δ 175.73, 175.65*, 175.32, 175.27* (C=O *i*Bu), 165.1 (C=O Bz), 152.7 (C-2), 151.9*, 151.8 (C-6), 149.7 (C-4), 142.4*, 142.2 (C-8), 133.5 (C_q *ipso*-Bz), 132.8 (*p*-Bz), 128.8 (*m*-Bz), 127.9 (*o*-Bz), 123.3 (C-5), 117.80, 117.76* (C_q CNE), 85.93, 85.55* (C-1'), 84.61 ($^2J_{CP}$ = 8.7 Hz), 84.57 ($^2J_{CP}$ = 8.6 Hz, C_q ᵗBu), 84.32, 84.25* (2 x d, $^2J_{CP}$ = 8.6, 8.5* Hz, C_q ᵗBu), 81.6, 81.5* (2 x d, $^3J_{CP}$ = 9.6, 9.5* Hz, C-4'), 73.1, 72.8* (C-2'), 70.5 (C-3'), 64.4, 64.0* (2 x d, $^2J_{CP}$ = 6.6, 6.5* Hz, C-5'), 42.85*, 42.83 (2 x dd, $^1J_{CP}$ = 138.6*, 138.9, 77.1*, 76.5 Hz, P-C_α-P), 33.8 (CH *i*Bu), 33.5 (CH *i*Bu), 30.42, 30.37, 30.34, 30.32, 30.29 (Me ᵗBu), 29.8*, 29.7 (2 x d, $^2J_{CP}$ = 3.1* , 3.1, SCH_2 CNE), 19.69, 19.66 (2 x d, $^3J_{CP}$ = 3.3, 3.6 Hz, CH_2CN), 18.82, 18.78, 18.73, 18.65, 18.6 (Me *i*Bu); **³¹P NMR** (202 MHz, $CDCl_3$) δ 99.59* (d, *J* = 12.4 Hz), 99.18 (d, *J* = 12.4 Hz, PS_2), 7.32 (d, *J* = 12.4 Hz), 7.28* (d, *J* = 12.4 Hz, PO); **HRMS** (ESI) m/z: $[M+H]^+$ calcd for $C_{38}H_{53}N_6O_{10}P_2S_2$ 867.2734, found 867.2732.

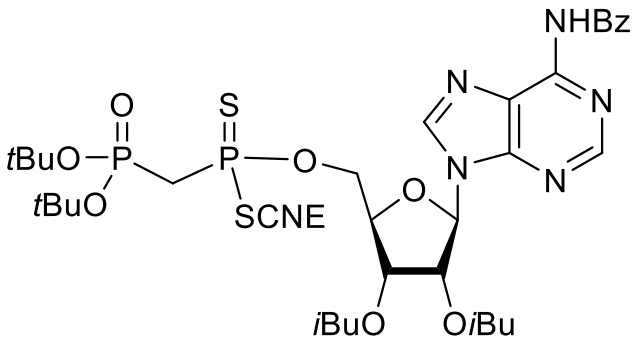

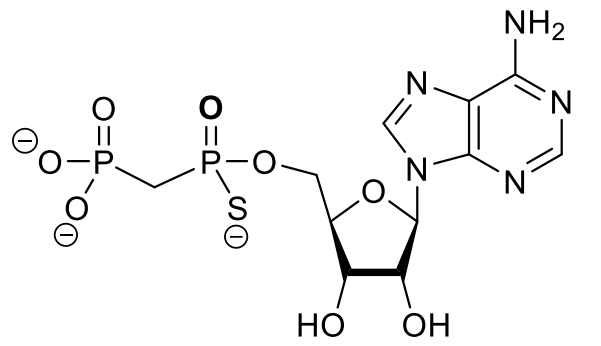

me-ADP-αS (13a) – Me-ADP-αS tetraester **12a** (230 mg, 0.27 mmol, 1.0 eq.) was dissolved in hexafluoroisopropanol (0.64 mL, 0.42 M) and treated with a concentrated aqueous hydrochloric acid in dioxane solution (1/1; v/v; 6 M, 54 µL, 0.32 mmol, 1.2 eq.) for 1.5 hour. The volatiles were removed *in vacuo* and the residue coevaporated with dioxane and acetonitrile. The bisphosphonic acid was redissolved in ammonium hydroxide (30 wt. %, 1.35 mL, 0.2 M) and stirred overnight, followed by evaporation of ammonia. The residue was taken up in H_2O, acidified to pH 4 with AcOH and washed with three portions of Et_2O. The aqueous layer was concentrated *in vacuo* and the crude desalted by size-exclusion chromatography (HW40-column, 0.15 M aqueous NH_4OAc). The material thus obtained was concentrated, coevaporated with MeCN/H_2O (1/1; v/v, 3 x) to remove NH_4OAc and subsequently lyophilized to furnish title me-ADP-αS **12a**, a white powder, as its triethylamine and ammonium salt (5:3 of P-diastereomers, 62.9 mg, 132.4 µmol, 49%, Et_3NH^+- and NH_4^+-form). No efforts were undertaken to resolve the PS-diastereomers. *Resonances and J-couplings marked with an asterisk * correspond to the minor P-diastereomer.* **^{1}H NMR** (400 MHz, D_2O) δ 8.50, 8.48* (2 x s, 1H, H-8), 8.09 (s, 1H, H-2), 6.01 (br. d, 1H, *J* = 5.0 Hz, H-1'), 4.72 – 4.68 (m, 1H, H-2', *verified via 1D NOESY presat, COSY, HSQC and HMBC*), 4.49 (t, 1H, *J* = 4.7 Hz, H-3'), 4.33 (br. s, 1H, H-4'), 4.23 – 4.10 (m, 2H, H-5'), 2.46 (dd, 2H, $^2J_{HP}$ = 19.4, 17.1 Hz, P-CH_2-P); **^{13}C NMR** (101 MHz, D_2O) δ 155.2 (C-6), 152.4 (C-2), 148.8 (C-4), 140.1 (C-8), 118.5 (C-5), 87.1 (C-1'), 83.8*, 83.7 (2 x d, $^3J_{CP}$ = 8.9*, 8.9 Hz, C-4'), 74.3 (C-2'), 70.37*, 70.34 (C-3'), 63.6*, 63.4 (2 x d, $^2J_{CP}$ = 6.1*, 6.1 Hz, C-5'), 37.2 (dd, $^1J_{CP}$ = 121, 92.7 Hz), 37.1 (dd, $^1J_{CP}$ = 122.5, 91.0 Hz, P-C_α-P); **^{31}P{^{1}H} NMR** (162 MHz, D_2O) δ 70.56 (d, *J* = 11.1 Hz), 70.42* (d, *J* = 10.8 Hz, PS), 13.80* (d, *J* = 10.6 Hz), δ 13.78 (d, *J* = 11.0 Hz, PO); **^{31}P NMR** (162 MHz, D_2O) δ 70.76 – 70.21 (m) 13.80 (td, $^2J_{PH}$ = 19.1, J_{PP} = 10.8 Hz), 13.78 (td, $^2J_{PH}$ = 19.2, J_{PP} = 11.1 Hz); **HRMS** (ESI) m/z: [M+H]$^+$ calcd for $C_{11}H_{18}N_5O_8P_2S$ 442.0346, found 442.0359.

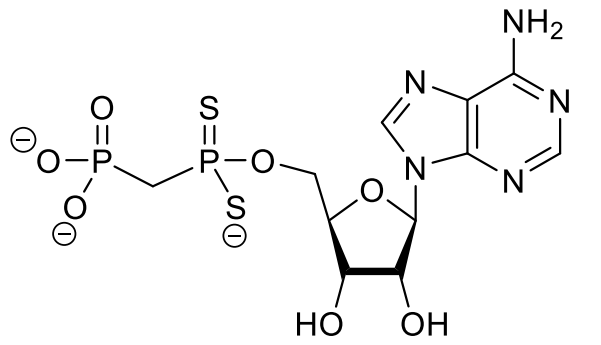

me-ADP-αS$_2$ (13b) – To a solution of me-ADP-αS$_2$ tetraester **12b** (70 mg, 80.7 µmol, 1.0 eq.), rendered anhydrous by evaporation of added acetonitrile (3 x), in dry acetonitrile (0.8 mL, 0.1 M) was added DBU (16.7 µL, 113 µmol, 1.4 eq.), and after 60 minutes ^{31}P NMR confirmed quantitative β-elimination. Subsequently, the volatiles were removed under reduced pressure, the residue redissolved in hexafluoroisopropanol (0.8 mL, 0.1 M) and treated with 6 M aq. HCl in dioxane (1/1; v/v, 45.3 µL, 272 µmol, 3.4 eq.) for 2.5 hours and concentrated *in vacuo*. The crude was coevaporated thrice with toluene and once with acetonitrile and the resulting bisphosphonic acid was taken up in aqueous ammonia (30 wt. %, 0.8 mL, 0.1 M), stirred overnight at ambient temperature and excess ammonia was removed *in vacuo*. The crude was partitioned between H_2O and Et_2O and the aqueous layer isolated, concentrated and the material thus obtained purified by size-exclusion chromatography (HW-40, 0.15 M NH_4OAc). Pooling of fractions, concentrating *in vacuo* and subsequent lyophilization furnished title me-ADP-αS$_2$ **13b** as a white powder (NH_4^+-form, 14.1 mg, 30.8 µmol, 38%). **^{1}H NMR** (400 MHz, D_2O) δ 8.58 (s, 1H, H-8), 8.11 (s, 1H, H-2), 6.04 (d, 1H, *J* = 5.4 Hz, H-1'), 4.75 (app. t, 1H, *J* = 5.3 Hz, H-2', *verified with NOESY presat*), 4.54 (dd, 1H, *J* = 5.0, 4.1 Hz, H-3'), 4.37 (dtd, 1H, *J* = 3.9, 3.0, 1.5 Hz, H-4'), 4.21 (app. dd, 2H, *J* = 8.1, 3.0 Hz, H-5'), 2.80 (dd, 2H, $^2J_{HP}$ = 18.4, 15.7 Hz, P-CH_2-P); **^{13}C NMR** (101 MHz, D_2O) δ 155.3 (C-6), 152.5 (C-2), 148.8 (C-4), 140.2 (C-8), 118.5 (C-5), 87.1 (C-1'), 83.68 (d, $^3J_{CP}$ = 9.8 Hz, C-4'), 74.3 (C-2'), 70.5 (C-3'), 62.93 (d, $^2J_{CP}$ = 6.5 Hz, C-5'), 44.35 (dd, $^1J_{CP}$ = 120.2, 67.9 Hz, P-C_α-P); **^{31}P{^{1}H} NMR** (162 MHz, D_2O) δ 107.67 (d, *J* = 12.6 Hz, PS_2), 12.80 (d, *J* = 12.7 Hz, PO_2); **^{31}P NMR** (162 MHz, D_2O) δ 107.66 (br. s), 12.80 (td, $^2J_{PH}$ = 18.6, J_{PP} = 12.5 Hz); **HRMS** (ESI) m/z: [M+H]$^+$ calcd for $C_{11}H_{18}N_5O_7P_2S_2$ 458.0117, found 458.0128.

***N*^{4}-benzoyl-2',3'-di-*O*-acetyl-di-*tert*-butyl-(((*R*$_P$/*S*$_P$)-cyanoethoxy)thiophosphonyl)methyl)phosphonate cytidine (15)** - A dry solution of phosphonamidite **2** (163 mg, 0.4 mmol, 1.0 eq.) and cytidine derivative **14** (181 mg, 0.42 mmol, 1.05 eq.) in acetonitrile (2.7 mL, 0.15 M) was treated with 4,5-dicyanoimidazole (57 mg, 0.48 mmol, 1.2 eq.) for 15 minutes. Subsequently, sulfurization was effected through the addition of Beaucage reagent (96 mg, 0.48 mmol, 1.2 eq.) and was complete within 30 minutes as gauged by ^{31}P NMR. The yellow mixture was poured into sat. aq. $NaHCO_3$, extracted with EtOAc and the organic layer was washed with sat. aq. NaCl, dried over Na_2SO_4, filtered and concentrated to dryness. The crude colourless oil was chromatographed (0 → 5 → 10 → 15 → 20 → 30% acetone in DCM) to furnish title me-CDP-αS tetraester **15** as a crystalline white solid (5:4 of P-diastereomers, 277 mg, 0.36 mmol, 90%). *Resonances and J-couplings marked with an asterisk * correspond to the minor P-diastereomer.* **^{1}H NMR** (400 MHz, $CDCl_3$) δ 8.94 (br. s, 1H, NH), 8.22, 8.17* (2 x d, 1H, *J* = 7.6, 7.7* Hz, H-6), 7.89 (app. d, 2H, *J* = 7.7 Hz, *o*-Bz), 7.62 – 7.54 (m, 2H, H-5, *p*-Bz), 7.48 (AA'<u>BB'</u>C as app. tt, 2H, *J* = 7.4, 1.6, 1.2 Hz, *m*-Bz), 6.23, 6.18* (d, 1H, *J* = 4.3, 4.8* Hz, H-1'), 5.45* (dd, *J* = 5.6, 4.7 Hz, H-2'), 5.41 – 5.35 (m, 2H, H-2', H-3'), 4.53 – 4.43 (m, 1H, H-5', H-5$_A$''), 4.43 – 4.27 (m, 4H, H-4', H-5$_B$'', OCH_2 CNE), 2.88 – 2.67 (m, 4H, P-CH_2-P, CH_2CN), 2.08, 2.08* (2 x s, 6H, CH_3 Ac), 1.53, 1.52, 1.52*, 1.51* (4 x s, 18H, CH_3 ^{t}Bu); **^{13}C NMR** (101 MHz, $CDCl_3$) δ 169.7, 169.6* (C=O Ac), 169.52, 169.50* (C=O Ac), 162.7 (C-4), 154.9 (C-2, *detected with HMBC*), 144.8 (C-6), 133.19 (*p*-Bz), 133.15 (*ipso*-Bz), 129.0 (*m*-Bz), 127.7 (*o*-Bz), 117.0 (C_q CNE), 116.9* (C_q CNE), 97.5 (C-5, *detected with HSQC*), 88.6*, 88.1 (C-1'), 84.16, 84.15, 84.13, 84.07 (C_q ^{t}Bu), 80.9, 80.6* (2 x d, $^3J_{CP}$ = 8.1, 8.1* Hz, C-4'), 73.7, 73.6* (C-2'), 69.8*, 69.7 (C-3'), 65.1, 64.6* (2 x d, $^2J_{CP}$ = 5.6, 6.0* Hz, C-5'), 61.9*, 61.6 (2 x d, $^2J_{CP}$ = 5.0*, 5.0 Hz, OCH_2 CNE), 37.99, 37.71* (2 x dd, $^1J_{CP}$ = 140.4, 139.9*, 104.2, 104.0* Hz, P-C_α-P), 30.39, 30.37, 30.35, 30.33, 30.30 (Me ^{t}Bu), 20.55, 20.54* (Me Ac), 20.5 (Me Ac), 19.7*, 19.6 (2 x d, $^3J_{CP}$ = 7.2*, 7.7 Hz, CH_2CN); **^{31}P NMR** (162 MHz, $CDCl_3$) δ 89.28 (d, *J* = 7.0 Hz), 89.22* (d, *J* = 8.2 Hz, PS), 7.74 (d, *J* = 7.1 Hz), 7.71* (d, *J* = 8.1 Hz, PO); **HRMS** (ESI) m/z: [M+H]$^+$ calcd for $C_{32}H_{45}N_4O_{12}P_2S$ 771.2224, found 771.2221.

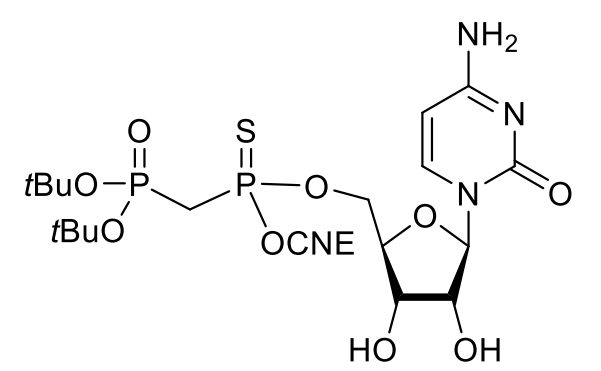

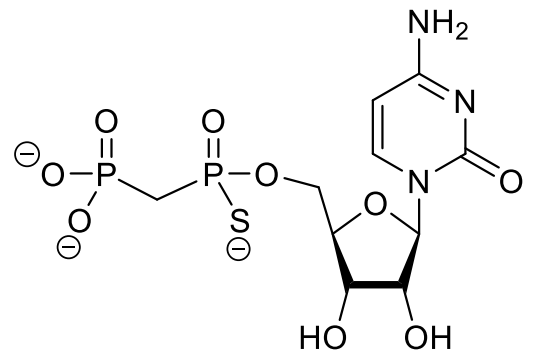

me-CDP-αS (16) – To a solution of me-CDP-αS tetraester **15** (178 mg, 0.23 mmol, 1.0 eq.) in hexafluoroisopropanol (0.55 mL, 0.42 M) was added a mixture of concentrated hydrochloric acid and dioxane (1/1; v/v; 6 M, 46.2 µL, 0.28 mmol, 1.2 eq.). After 90 minutes the volatiles were removed under reduced pressure. Residual hydrochloric acid and hexafluoroisopropanol were removed by azeotroping thrice with dioxane and once with acetonitrile, yielding a white solid. The crude was taken up in aqueous ammonia (30 wt. %, 1.16 mL, 0.2 M), stirred overnight and excess ammonia was purged *in vacuo*. The material thus obtained was directly purified by size-exclusion chromatography (HW40-column, 0.15 M aqueous NH_4OAc), coevaporated thrice with MeCN/H_2O (1/1; v/v, 3 x) and lyophilized to give the trisammonium salt of me-CD-αS **16** as a white powder (1:1 of P-diastereomers, 76.1 mg, 168.6 µmol, 73%, NH_4+-form). No efforts were made to resolve the P-diastereomers. *Resonances and J-couplings marked with an asterisk * correspond to one of the P-diastereomers.* **^{1}H NMR** (400 MHz, D_2O) δ 7.93, 7.91* (2 x d, 1H, *J* = 7.3, 7.1* Hz, H-6), 6.01 (d, 1H, *J* = 7.6 Hz, H-5), 5.85 (d, 1H, *J* = 3.8 Hz, H-1'), 4.29 – 4.02 (m, 5H, H-2', H-3', H-4', H-5'), 2.43 (dd, 2H, $^{2}J_{HP}$ = 19.0, 17.4 Hz, P-CH_2-P); **^{13}C NMR** (101 MHz, D_2O) δ 165.7 (C-4), 157.2 (C-2), 141.9, 141.8* (C-6), 96.3 (C-5), 89.5 (C-1), 82.57, 82.54* (2 x d, $^{3}J_{CP}$ = 8.5, 8.8* Hz, C-4'), 74.13, 74.11* (C-2'), 69.3 (C-3'), 63.0, 62.9* (2 x d, $^{2}J_{CP}$ = 5.8, 6.3* Hz, C-5'), 37.15, 37.11* (2 x dd, $^{1}J_{CP}$ = 121.4, 121.6*, 92.0, 91.8* Hz, P-C_α-P); **^{31}P{^{1}H} NMR** (162 MHz, D_2O) δ 70.39 (d, *J* = 10.4 Hz, PS), 13.58 (d, *J* = 10.2 Hz, PO); **^{31}P NMR** (162 MHz, D_2O) δ 70.61 – 70.16 (m), 13.58 (td, $^{2}J_{PH}$ = 19.3 Hz, J_{PP} = 10.1 Hz); **HRMS** (ESI) m/z: [M+H]$^+$ calcd for $C_{10}H_{18}N_3O_9P_2S$ 418.0234, found 418.0247.

***N*^{2}-di-*tert*-butyloxycarbonyl-*O*^{6}-*tert*-butyl-2',3',-*O*-isopropylidene-di-*tert*-butyl(((*R*$_P$/*S*$_P$)-*tert*-butyloxy)thiophosphoryl)methyl)phosphonate guanosine (18) –** Tri-*tert*-butylphosphonamidite **3** (92 mg, 0.22 mmol, 1.0 eq.) and guanosine derivative **17** (135 mg, 0.23 mmol, 1.05 eq.) were rendered dry by coevaporating thrice with acetonitrile and then dissolved in anhydrous acetonitrile (1.5 mL, 0.15 M). The turbid mixture was vigorously stirred until homogeneous and treated with 4,5-dicyanoimidazole (55 mg, 0.47 mmol, 2.0 eq.) for 30 minutes, followed by Beaucage reagent (67 mg, 0.34 mmol, 1.5 eq.) for an additional 30 minutes. The mixture was diluted with EtOAc (10 mL), successively washed with H_2O, sat. aq. $NaHCO_3$ and sat. aq. NaCl, dried over Na_2SO_4, filtered and concentrated *in vacuo*. Purification by silica gel column chromatography (0 → 5 → 10 → 15 → 20 → 25 → 30% acetone in dichloromethane) afforded title me-GDP-αS tetraester **18** as a crystalline white foam (1:1 mixture of P-diastereomers, 171 mg, 0.185 mmol, 83%) with an inseparable by column chromatography residual nucleoside **17** (< 20%). *The nucleoside is separable by size-exclusion chromatography after subsequent steps. Alternatively, the phosphonamidite can be used in excess. The condensation proceeds equally well under the agency of 1.5 eq. of activator. Resonances and J-couplings marked with an asterisk * correspond to one of the P-diastereomers.* **^{1}H NMR** (400 MHz, $CDCl_3$) δ 8.21, 8.15* (2 x s, 1H, H-8), 6.15 (d, 1H, *J* = 2.6 Hz, H-1'), 5.32, 5.28* (2 x dd, 1H, *J* = 6.3, 6.3*, 2.7, 2.5* Hz, H-2'), 5.11, 5.06* (2 x dd, 1H, *J* = 6.4, 6.3*, 3.4, 3.4* Hz, H-3'), 4.49 – 4.43 (m, 1H, H-4'), 4.38 – 4.27 (m 1H, H-5$_A$'), 4.27 – 4.17 (m, 1H, H-5$_B$'), 2.66 – 2.53 (m, 2H, P-CH_2-P), 1.70 (s, 9H, CH_3 *O*^{6}-^{t}Bu), 1.59 (s, 3H, CH_3 CMe_2), 1.52, 1.48 (2 x s, 9H, CH_3 P(O^{t}Bu), 1.50, 1.49 (2 x s, 18H, CH_3 P(O^{t}Bu)), 1.45 (s, 9H, CH_3 *N*^{2}-Boc_2), 1.44, 1.44 (2 x s, 9H, CH_3 *N*^{2}-Boc_2), 1.35 (s, 3H, CH_3 CMe_2); **^{13}C NMR** (101 MHz, $CDCl_3$) δ 161.1 (C-6), 151.94, 151.86* (C-4), 151.10, 151.05* (C-2), 150.7 (C=O Boc), 141.5, 141.3* (C-8), 121.2 (C-5), 114.65, 114.61* (C_q CMe_2), 90.0, 89.8* (C-1'), 85.70 (*signal detected with HMBC*, C_q P(O ^{t}Bu)), 85.5, 85.3* (2 x d, $^{3}J_{CP}$ = 9.3, 9.2 Hz, C-4'), 84.46, 84.32* (C-2'), 84.16, 84.12* (C_q *O*^{6}-^{t}Bu), 83.5 (C_q ^{t}Bu), 83.15, 83.13 (C_q Boc), 81.44, 81.35* (C-3'), 66.4, 66.3* (d, $^{2}J_{CP}$ = 5.9, 6.0 Hz, C-5'), 41.41 – 38.54 (m, P-C_α-P, *signal detected with HSQC*), 30.6, 30.53, 30.49, 30.44, 30.40, 30.36, 30.3 (Me ^{t}Bu) , 28.5, 28.4 (Me *O*^{6}-^{t}Bu), 28.1 (Me Boc), 27.32, 27.29* (Me CMe_2), 25.5, 25.4* (Me CMe_2)**;** **^{31}P NMR** (162 MHz, $CDCl_3$) δ 76.95 (d, *J* = 14.1 Hz), 76.64* (d, *J* = 14.0 Hz, PS), 9.19 (d, *J* = 14.0 Hz), 8.93* (d, *J* = 14.1 Hz, PO); **HRMS** (ESI) m/z: [M+H]$^+$ calcd for $C_{40}H_{70}N_5O_{13}P_2S$ 922.4161, found 922.4163.

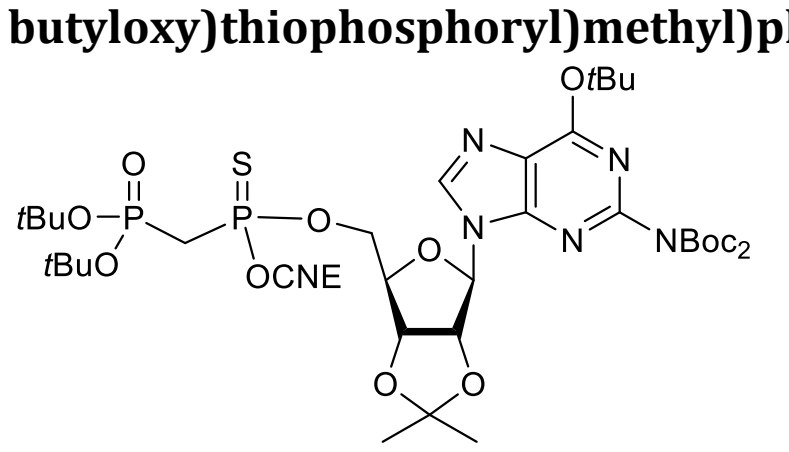

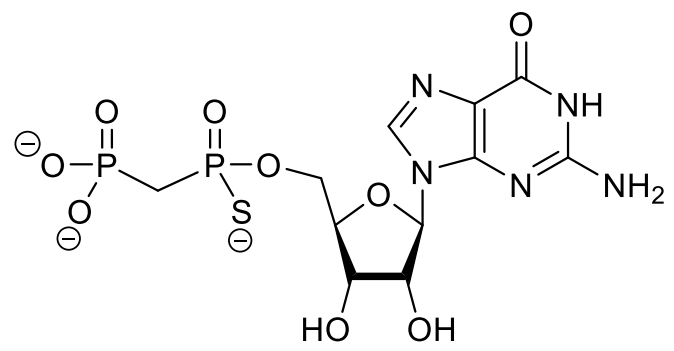

me-GDP-αS (19)– Me-GDP-αS tetraester **18** (44 mg, 47.8 µmol, 1.0 eq.) was cooled to 0 °C in an ice bath and a freshly prepared cold 50% aqueous trifluoroacetic acid (0.96 mL, 0.05 M) was added. This temperature was maintained for 2 hours whereafter the cooling was removed and the cloudy mixture was stirred overnight while gradually warming to ambient temperature. After LCMS indicated complete acidolysis, the now homogenous solution was concentrated to dryness. Excess trifluoroacetic acid was removed by coevaporating with three portions of toluene and the resultant oil was redissolved in aqueous ammonia (30 wt.%, 1 mL) and concentrated *in vacuo* to give the crude trisammonium salt. Purification by size-exclusion chromatography and lyophilization afforded title me-GDP-αS **19** as a white powder (1:1 of P-diastereomers, NH_4^+-form, 13.6 mg, 26.7 µmol, 56%). No efforts were undertaken to resolve the PS-diastereomers. *Resonances and J-couplings marked with an asterisk * correspond to one of the P-diastereomers.* **^{1}H NMR** (400 MHz, D_2O) δ 8.39, 8.36* (2 x s, 1H, H-8), 5.88 (d, 1H, *J* = 5.1 Hz, H-1'), 4.71, 4.70* (2 x app. t, 1H, *J* = 4.8, 4.7* Hz, H-2'), 4.50 (app. t, 1H, *J* = 4.7 Hz, H-3'), 4.32 (app. q, 1H, *J* = 3.8 Hz, H-4'), 4.27 – 4.12 (m, 2H, H-5'), 2.49 (dd, 2H, $^{2}J_{HP}$ = 19.2, 17.2 Hz, P-CH_2-P); **^{13}C NMR** (101 MHz, D_2O) δ 158.03, 157.98 (C-6), 154.2 (C-2), 151.11, 151.09* (C-4), 137.4 (C-8), 114.5, 114.4* (C-5), 87.6 (C-1'), 83.70, 83.67* (2 x d, $^{3}J_{CP}$ = 8.8, 8.7* Hz, C-4'), 73.93, 73.90* (C-2'), 70.13, 70.08* (C-3'), 63.41, 63.28* (2 x d, $^{2}J_{CP}$ = 6.1, 6.0* Hz, C-5'), 37.03, 36.95* (2 x dd, $^{1}J_{CP}$ = 121.9, 121.5* 92.3, 91.7* Hz, P-C_α-P); **^{31}P NMR** (162 MHz, D_2O) δ 70.46 (d, *J* = 11.8 Hz), 70.33* (d, *J* = 11.5 Hz, PS), 14.09 (d, *J* = 11.7 Hz, PO); **^{31}P{^{1}H} NMR** (162 MHz, D_2O) δ 70.61 – 70.10 (m), 14.09 (td, $^{2}J_{PH}$ = 19.2, J_{PP} 11.7 Hz); **^{31}P{^{1}H} NMR** (202 MHz, D_2O) δ 70.43 (d, *J* = 11.7 Hz), 70.29

(d, J = 11.6 Hz, PS), 14.00 (d, J = 11.7 Hz, PO); **^{31}P NMR** (202 MHz, D_2O) δ 70.61 – 70.10 (m), 14.00 (td, $^2J_{PH}$ = 19.3, J_{PP} 11.4 Hz); **HRMS** (ESI) m/z: $[M+H]^+$ calcd for $C_{11}H_{18}N_5O_9P_2S$ 458.0295, found 458.0308.

Synthesis of 1,2,3,4-*tetra-O*-acetyl-D-ribitol 24

Scheme S1. Synthesis of 1,2,3,4-*tetra-O*-acetyl- D-ribitol

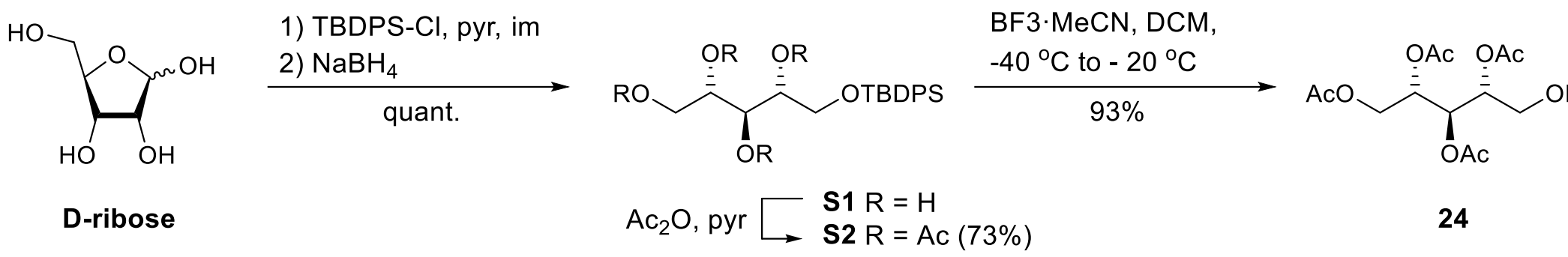

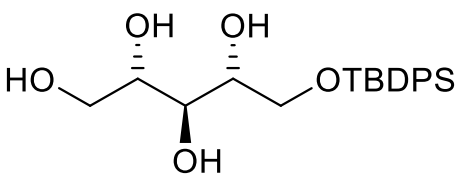

5-*O*-*tert*-butyldiphenylsilyl-D-ribitol (S1) – Commercially available D-ribose (1.5 g, 10 mmol) was coevaporated thrice with anhydrous pyridine and suspended in anhydrous pyridine (50 mL, 0.2 M), whereto imidazole (1.36 g, 20.0 mmol, 2.0 eq.) and *tert*-butyldiphenylsilyl chloride (2.9 mL, 11.0 mmol, 1.1 eq.) where added consecutively. The pale solution was stirred for 16 hours, cooled in an ice bath and sodium borohydride (3.0 g, 80.0 mmol, 8.0 eq.) in ice-cold methanol (2.0 mL) was added in succession. After cessation of effervescence, cooling was removed and stirring continued for 3 hours while warming to ambient temperature. Hereafter, the turbid mixture was ice-cooled, carefully quenched by the dropwise addition of acetic acid until foaming ceased and diluted with MeOH (20 mL). To the now clear solution was added Celite® and the volatiles were evaporated *in vacuo*. The crude was dry loaded and chromatographed (0 → 1 → 2 → 3 → 5% MeOH in DCM) to afford title tetraol **S1** as a colourless oil (3.9 g, 10.0 mmol, quant.). The material was carried on without further purification. **^{1}H NMR** (500 MHz, $CDCl_3$) δ 7.66 – 7.60 (m, 4H, *o*-Ph TBDPS), 7.40 – 7.29 (m, 6H, *m*-, *p*-Ph TBDPS), 3.99 (br. s, 3H, 3 x OH), 3.87 – 3.66 (m, 7H, H-1, H-1', H-2, H-3, H-4, H-5, H-5'), 1.03 (s, 9H, CH_3 ^{t}Bu TBDPS); **^{13}C NMR** (126 MHz, $CDCl_3$) δ 135.6 (*o*-Ph), 132.80 (*ipso*-Ph), 132.78 (*ipso*-Ph), 130.0 (*p*-Ph), 128.0 (*m*-Ph), 73.1, 73.9, 72.3 (C-2, C-3, C-4), 65.7 (C-1), 63.2 (C-5), 27.0 (Me ^{t}Bu), 19.3 (C_q ^{t}Bu); **HRMS** (ESI) m/z: $[M+Na]^+$ calcd for $C_{21}H_{30}O_5SiNa$ 413.1755, found 413.1761.

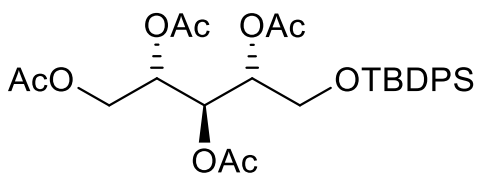

1,2,3,4-*tetra-O*-acetyl-5-*O*-*tert*-butyldiphenylsilyl-D-ribitol (S2) – Tetraol **S1** (1.95 g, 5.0 mmol, 1.0 eq.) was dissolved in acetic anhydride/pyridine (2/1; v/v, 50 mL, 0.2 M), stirred for 5 hours and the volatiles removed under reduced pressure. The resulting residue was partitioned between EtOAc and sat. aq. $NaHCO_3$, the layers were separated and the aqueous washing was extracted twice more with EtOAc. The organic extracts were combined, dried over $MgSO_4$, filtered and concentrated *in vacuo*. Purification by flash chromatography (0 → 5 → 10 → 15 → 20% EtOAc in pentane) afforded tetraacetate **S2** as a viscous colourless oil (2.04 g, 3.65 mmol, 73% over 3 steps). **^{1}H NMR** (500 MHz, $CDCl_3$) δ 7.67 – 7.62 (m, 4H, *o*-Ph TBDPS), 7.45 – 7.36 (m, 6H, *m*-, *p*-Ph TBDPS), 5.43 (app. t, 1H, J = 5.3 Hz, H-3), 5.34 (ddd, 1H, J = 6.5, 5.1, 3.2 Hz, H-2), 5.21 (td, 1H, J = 5.7, 3.6 Hz, H-4), 4.34 (dd, 1H, J_{ABX} = 12.2, 3.2 Hz, H-1_A), 4.15 (dd, 1H, J_{BAX} = 12.2, 6.7 Hz, H-1_B), 3.80 (dd, 1H, J_{ABX} = 11.4, 3.6 Hz, H-5_A), 3.73 (dd, 1H, J_{BAX} = 11.4, 5.8 Hz, H-5_B), 2.06 (s, 3H, CH_3 Ac), 2.03 (s, 3H, CH_3 Ac), 2.02 (s, 3H, CH_3 Ac), 1.97 (s, 3H, CH_3 Ac), 1.03 (s, 9H, CH_3 ^{t}Bu TBDPS); **^{13}C NMR** (126 MHz, $CDCl_3$) δ 170.7 (C=O Ac), 170.0 (C=O Ac), 169.9 (C=O Ac), 169.5 (C=O Ac), 135.73 (*o*-Ph), 135.67 (*o*-Ph), 133.1 (*ipso*-Ph), 133.0 (*ipso*-Ph), 133.03 (*p*-Ph), 129.98 (*p*-Ph), 127.9 (*m*-Ph), 72.3 (C-4), 69.9 (C-2), 69.6 (C-3), 62.2 (C-5), 62.1 (C-1), 26.7 (Me ^{t}Bu), 21.0 (Me Ac), 20.9 (Me Ac), 20.8 (Me Ac), 19.3 (C_q ^{t}Bu); **HRMS** (ESI) m/z: $[M+H+NH_3]^+$ calcd for $C_{29}H_{42}NO_9Si$ 576.2623, found 576.2626.

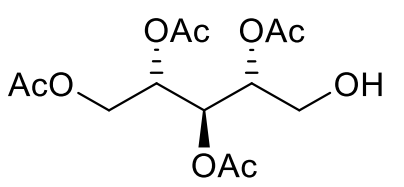

1,2,3,4-*tetra-O*-acetyl-D-ribitol (24) – A solution of silylated **S2** (168 mg, 0.3 mmol, 1.0 eq.) in anhydrous dichloromethane (6.0 mL, 0.05 M) was cooled to -40 °C. Hereto, ice-cold boron trifluoride acetonitrile complex (15.2–16.8% BF_3·MeCN, 0.3 mL) was added dropwise, and the reaction was maintained at 0 °C for 1 hour. Hereafter, the mixture was allowed to gradually warm to -20 °C over the duration of 2 hours, whereupon TLC indicated complete conversion into a single lower migrating spot. The solution was poured into a separatory funnel containing phosphate buffer, extracted thrice with DCM, and the resulting organic extracts were combined, dried over Na_2SO_4, filtered, and evaporated to dryness. The crude was purified by silica gel flash column chromatography (0 → 5 → 10 → 20 → 30% acetone/DCM). Pooling of product containing fractions and concentrating under reduced pressure afforded alcohol **24** as a colourless oil (86 mg, 0.27 mmol, 90%). **^{1}H NMR** (300 MHz, $CDCl_3$) δ 5.38 – 5.28 (m, 2H, H-2, H-3), 5.09 (td, 1H, J= 5.5, 3.7 Hz, H-4), 4.38 (dd, 1H, J_{ABX} = 12.2, 2.9 Hz, H-1_A), 4.16 (dd, 1H, J_{BAX} = 12.1, 6.4 Hz, H-1_B), 3.82 (dd, 1H, J_{ABX} = 12.4, 3.7 Hz, H-5_A), 3.67 (dd, 1H, J_{BAX} = 12.4, 5.6 Hz, H-5_B), 3.04 (br. s, 1H, 5-OH), 2.13 (s, 3H, CH_3 Ac), 2.10 (s, 3H, CH_3 Ac), 2.09 (s, 3H, CH_3 Ac), 2.05 (s, 3H, CH_3 Ac); **^{13}C NMR** (75 MHz, $CDCl_3$) δ 170.6, 170.3, 170.1, 169.8 (C=O Ac), 72.4 (C-4), 69.7, 69.6 (C-2, C-3), 61.8 (C-1), 60.6 (C-5), 20.8, 20.7, 20.57, 20.55 (Me Ac); **HRMS** (ESI) m/z: $[M+Na]^+$ calcd for $C_{13}H_{20}O_9Na$ 343.1000, found 343.1002.

Synthesis guanosine derivative 17 with acid-sensitive protections

Scheme S2. Synthesis of acid-labile guanosine derivative **17** (based on a reported procedure[39])

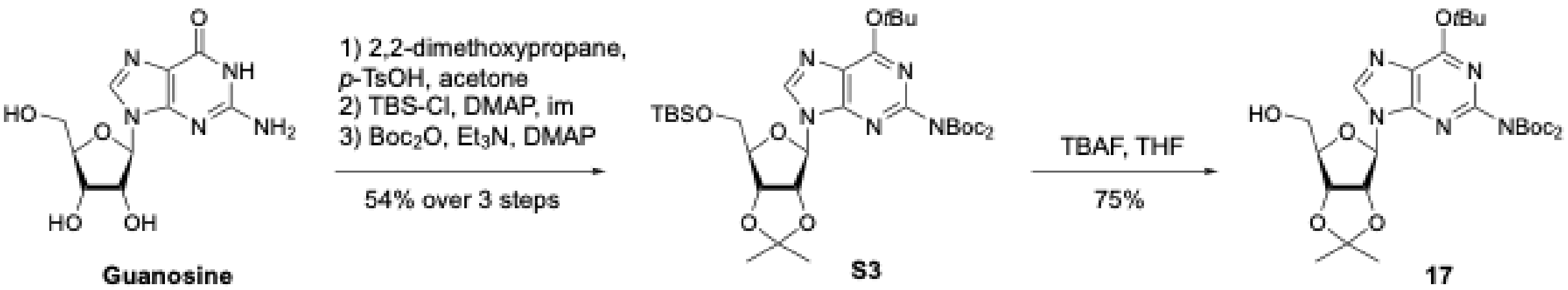

***N*²-di-*tert*-butoxycarbonyl-*O*⁶-*tert*-butyl-2',3'-*O*-isopropylidene-5'-O-*tert*-butyldi methylsilyl guanosine (S3) –** Guanosine (14.1 g, 50 mmol) was suspended in acetone (500 mL, 0.1 M), 2,2-dimethoxypropane (73.4 mL, 600 mmol, 12 eq.) and *p*-toluenesulfonic acid (8.6 g, 50 mmol, 1 eq.) were added and the reaction mixture was stirred overnight at room temperature under a nitrogen atmosphere. After full conversion was observed by TLC analysis, the reaction was quenched by the addition of Et_3N (pH 7-8) and concentrated to dryness, followed by repeated co-evaporation with DMF (3x) to remove residual methanol. The crude 2',3'-*O*-isopropylidene guanosine was subsequently dissolved in DMF (500 mL, 0.1 M) and brought under a nitrogen atmosphere. Then, imidazole (8.5 g, 125 mmol, 2.5 eq.), DMAP (1.2 g, 10 mmol, 0.2 eq.), and lastly *tert*-butyldimethylsilyl chloride (15.1 g, 100 mmol, 2 eq.) were added and the reaction mixture was stirred overnight at room temperature. After full conversion was established by TLC, the reaction was quenched by the addition of MeOH (10 mL). After transferring the mixture to a separatory funnel containing 0.01 M aq. HCl, the organic and aqueous layers were separated and the aqueous layer was extracted with EtOAc (3x). The pooled organic layers were washed with sat. aq. $NaHCO_3$ and brine, dried over $MgSO_4$, filtered, and concentrated *in vacuo.* The crude material was dissolved in anhydrous DCM (100 mL, 0.1 M) and cooled on ice. Triethylamine (48 mL, 350 mmol, 7 eq.), DMAP (3.0 g, 25 mmol, 0.5 eq.), and Boc_2O (52.5 g, 250 mmol, 5.0 eq.) were added on ice and the ice bath was immediately removed. After overnight stirring at room temperature, TLC(-MS) analysis showed full conversion to the O^6-*t*Bu product and the mixture was quenched with the addition of MeOH (20 mL) and diluted with sat. aq. $NaHCO_3$. The aqueous layer was extracted three more times with EtOAc, after which the combined organic layers were washed with sat. aq. $NaHCO_3$ and brine, dried over $MgSO_4$, filtered and concentrated under reduced pressure. Purification by silica gel column chromatography of the crude product (40→50→60→70% Et_2O in pentane) afforded title compound **S3** as a crystalline white foam (18.6 g, 26.8 mmol, 54% over 3 steps). **¹H NMR** (500 MHz, $CDCl_3$) δ 8.11 (s, 1H, H-8), 6.15 (d, *J* = 2.6 Hz, 1H, H-1'), 5.12 (dd, *J* = 6.2, 2.6 Hz, 1H, H-2'), 4.91 (dd, *J* = 6.1, 3.0 Hz, 1H, H-3'), 4.34 (app. td, *J* = 4.2, 3.0 Hz, 1H, H-4'), 3.84 (dd, *J* = 11.3, 3.9 Hz, 1H, H-5_A'), 3.75 (dd, *J* = 11.3, 4.6 Hz, 1H, H-5_B'), 1.70 (s, 9H, O^6-*t*Bu), 1.59 (s, 3H, CH_3 CMe_2), 1.43 (2 x s, 18H, N^2-Boc_2), 1.35 (s, 3H, CH_3 CMe_2), 0.87 (s, 9H, CH_3 ^{t}Bu TBS), 0.03 (s, 6H, CH_3 TBS); **¹³C NMR** (126 MHz, $CDCl_3$): δ 161.1 (C-6), 151.8 (C-4), 151.3 (C-2), 151.0 (C=O Boc), 141.1 (C-8), 121.3 (C-5), 114.3 (C_q CMe_2), 90.6 (C-1'), 87.3 (C-4'), 85.0 (C-2'), 84.0 (C_q O^6-^{t}Bu), 82.9 (C_q Boc), 81.3 (C-3'), 63.7 (C-5'), 28.5 (Me O^6-^{t}Bu), 28.0 (Me Boc), 27.4 (Me CMe_2), 26.1 (Me ^{t}Bu), 25.5 (Me CMe_2), 18.5 (C_q ^{t}Bu), –5.2, –5.3 (Me TBS); **HRMS** (ESI) m/z: $[M+Na]^+$ calcd for $C_{33}H_{55}N_5O_9SiNa$ 694.3842, found 694.3842.

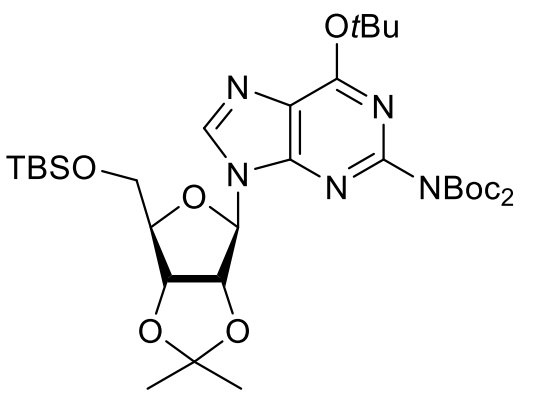

***N*²-di-*tert*-butoxycarbonyl-*O*⁶-*tert*-butyl-2',3'-*O*-isopropylidene guanosine (17) –** Guanosine derivative **S3** (18.6 g, 26.8 mmol) was dissolved in anhydrous THF (270 mL, 0.1 M) and brought under a nitrogen atmosphere, followed by the addition of TBAF (1M in THF; 40.2 mL, 40.2 mmol, 1.5 eq.) stirring for 1 hour at room temperature. After TLC analysis showed full conversion, the reaction mixture was concentrated *in vacuo.* The crude material was then re-dissolved in DCM and poured in a separatory funnel containing sat. aq. $NaHCO_3$ and brine (1:1; v/v). The organic and aqueous layers were separated and the aqueous layer was extracted thrice more with DCM. The organic layers were combined and washed once with brine, dried over $MgSO_4$, filtered, and concentrated to dryness. Flash column chromatography of the crude material (SiO_2, 50→60→70→80% EtOAc in pentane) afforded title compound **17** as a crystalline off-white foam (11.7 g, 20 mmol, 75%). **¹H NMR** (500 MHz, $CDCl_3$) δ 7.95 (s, 1H, H-8), 5.88 (d, *J* = 4.3 Hz, 1H, H-1'), 5.23 (dd, *J* = 5.9, 4.4 Hz, 1H, H-2'), 5.08 (dd, *J* = 5.9, 1.7 Hz, 1H, H-3'), 4.79 (dd, *J* = 10.4, 3.1 Hz, 1H, 5'-OH), 4.45 (app. q, *J* = 2.3 Hz, 1H, H-4'), 3.84 (app. dt, *J* = 12.6, 2.6 Hz, 1H, H-5_A'), 3.71 (ddd, *J* = 12.7, 10.3, 2.8 Hz, 1H, H-5_B'), 1.70 (s, 9H, O^6-*t*Bu), 1.62 (s, 3H, CH_3 CMe_2), 1.44 (2 x s, 18H, N^2-Boc_2), 1.36 (s, 3H, CH_3 CMe_2); **¹³C NMR** (126 MHz, $CDCl_3$): δ 161.6 (C-6), 151.1 (C-4), 151.1 (C-2), 150.8 (C=O Boc), 142.3 (C-8), 122.5 (C-5), 114.1 (C_q CMe_2), 93.5 (C-1'), 86.5 (C-4'), 84.7 (C_q O^6-^{t}Bu), 83.3 (C_q Boc), 83.2 (C-2'), 81.7 (C-3'), 63.2 (C-5'), 28.4 (Me O^6-^{t}Bu), 28.0 (Me Boc), 27.7, 25.3 (Me CMe_2); **HRMS** (ESI) m/z: $[M+Na]^+$ calcd for $C_{27}H_{41}N_5O_9Na$, 602.2796 found 602.2797

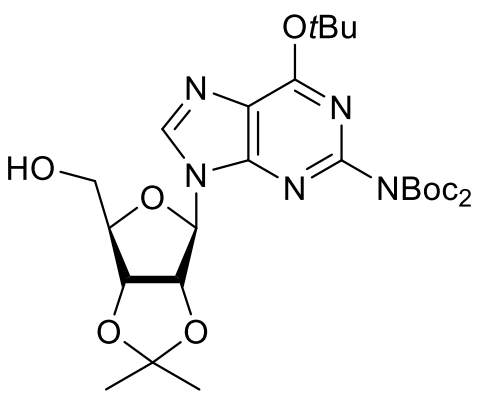

Synthesis of Non-Symmetric Phosphonylmethyl Phosphono(di)thioate Diesters

N^4-benzoyl-2',3'-di-*O*-acetyl-(*R*$_P$/*S*$_P$)-*tert*-butylmethyl-(((*R*$_P$/*S*$_P$)-(cyanoethoxy)phosphorothioyl)methyl)phosphonate cytidine (20a) – A dry solution of phosphonamidite **4** (275 mg, 0.75 mmol, 1.0 eq.) and cytidine derivative **14** (340 mg, 0.79 mmol, 1.05 eq.) in anhydrous acetonitrile (3.6 mL, 0.15 M) was stirred until homogenous, whereupon 4,5-dicyanoimidazole (106 mg, 0.9 mmol, 1.2 eq.) was added. After 30 minutes the solution was treated with Beaucage reagent (180 mg, 0.9 mmol, 1.2 eq.) for an additional 20 minutes, diluted with EtOAc, successively washed with H_2O, sat. aq. $NaHCO_3$, dried over Na_2SO_4, filtered and evaporated to dryness. Purification by silica gel column chromatography (0 → 5 → 10 → 15 → 20 → 25 → 30 → 40% acetone in dichloromethane) furnished title me-CDP-αS tetraester **20a** as a brittle, crystalline, white foam (mixture of 4 P-diastereomers, 494 mg, 0.68 mmol, 90%). No efforts were undertaken to resolve the four P-diastereomers. **^{1}H NMR** (400 MHz, $CDCl_3$) δ 8.87 (br. s, 1H, NH), 8.24, 8.22, 8.18, 8.15 (4 x d, 1H, *J* = 7.6, 7.6, 7.6, 7.6 Hz, H-6), 7.89 (app. d, 2H, *J* = 7.7 Hz, *o*-Bz), 7.62 – 7.55 (m, H-5, *p*-Bz), 7.49 (app. t, 2H, *J* = 7.4 Hz, *m*-Bz), 6.25, 6.23, 6.22, 6.20 (4 x d, 1H, *J* = 4.5, 4.2, 4.0, 4.2 Hz, H-1'), 5.48 – 5.36 (m, 2H, H-2', H-3'), 4.55 – 4.28 (m, 5H, H-4', H-5', OCH_2 CNE), 3.81, 3.79, 3.79, 3.78 (4 x d, 3H, $^3J_{HP}$ = 11.7, 11.7, 11.6, 11.9 Hz, P-OCH_3), 2.88 – 2.71 (m, 4H, P-CH_2-P, CH_2CNE), 2.08 (s, 6H, CH_3 Ac), 1.53, 1.53, 1.52, 1.51 (4 x s, 9H, CH_3 ^{t}Bu); **^{13}C NMR** (101 MHz, $CDCl_3$) δ 169.43, 169.40, 169.34, 169.27, 169.24 (C=O Ac), 166.8 (C=O Bz), 162.9 (C-4), 154.9 (C-2), 144.9, 144.8, 144.6 (C-6), 132.9 (*p*-Bz), 132.8 (*ipso*-Bz), 128.6 (*m*-Bz), 127.7 (*o*-Bz), 116.93, 116.90, 116.86 (C_q CNE), 97.5 (C-5), 88.7, 88.5, 88.4, 88.3 (C-1'), 84.79, 84.70, 84.68 84.65, 84.60, 84.56 (app. $^2J_{CP}$ = 9, 8 Hz, C_q ^{t}Bu), 80.47, 80.43, 80.27 (3 x d, $^3J_{CP}$ = 8.7, 7.9, 7.9 Hz, C-4'), 73.46, 73.43, 73.38, 73.34 (C-2'), 69.36, 69.32, 69.22, 69.11 (C-3'), 65.0, 64.9, 64.6, 64.5 (4 x d, $^2J_{CP}$ = 5.5, 4.8, 5.5, 5.2 Hz, C-5'), 61.8, 61.7, 61.5 (3 x d, $^2J_{CP}$ = 4.8, 5.0, 5.0 Hz, OCH_2 CNE), 53.10, 53.07, 53.03, 52.95 (P-OMe), 34.8 (dd, $^1J_{CP}$ = 139.5, 106.3 Hz, P-$C_α$-P), 34.6, 34.5 (2 x dd, app. $^1J_{CP}$ = 138, 106 Hz, P-$C_α$-P), 30.12, 30.08, 30.05 (Me ^{t}Bu), 20.33 (Me Ac), 20.29 (Me Ac), 19.45, 19.39 (2 x d, $^3J_{CP}$ = 8.7, 9.1 Hz, CH_2CN); **^{31}P NMR** (162 MHz, $CDCl_3$) δ 88.16, 88.14, 88.09, 88.08, 87.97, 87.95, 87.93, 87.92 (PS), 14.14, 14.13, 14.06, 14.04, 14.03, 14.01, 13.92, 13.90 (PO); **HRMS** (ESI) m/z: $[M+H]^+$ calcd for $C_{29}H_{39}N_4O_{12}P_2S$ 729.1755, found 729.1752.

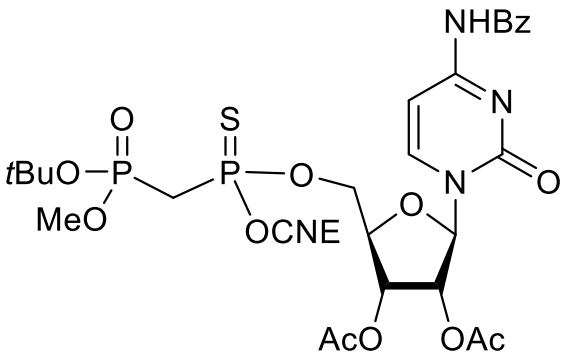

N^4-benzoyl-2',3'-di-*O*-acetyl-(*R*$_P$/*S*$_P$)-*tert*-butylmethyl-(((*R*$_P$/*S*$_P$)(mercaptopropionitrile)dithiophosphoryl)methyl)phosphonate cytidine (20b) - Thiophosphonamidite **6** (162 mg, 0.42 mmol, 1.0 eq.) and cytidine nucleoside **14** (192 mg, 0.45 mmol, 1.05 eq.) were rendered dry by azeotroping thrice with anhydrous acetonitrile, taken up in anhydrous acetonitrile (0.28 mL, 0.15 M) and stirred until all dissolved and the mixture appeared clear and homogenous. Hereto, 4,5-dicyanoimidazole (60 mg, 0.51 mmol, 1.2 eq.) was added and, after 30 minutes, sulfurization was effected by the addition of Beaucage reagent (102 mg, 0.51 mmol, 1.2 eq.). After stirring for an additional 30 minutes the pale yellow mixture was diluted with EtOAc, successively washed with sat. aq. $NaHCO_3$ and sat. aq. NaCl. The resulting organic layer was dried over Na_2SO_4, filtered and concentrated *in vacuo*. Purification of the yellowish residue by silica gel column chromatography (0 → 2.5 → 5 → 10 → 15 → 20% acetone in dichloromethane) furnished title me-CDP-αS_2 tetraester **20b** as a crystalline white solid (mixture of 4 P-diastereomers, 274 mg, 0.37 mmol, 87%) with residual inseparable by column chromatography nucleoside **14**. *The nucleoside is inconsequential and separable via size-exclusion chromatography or the thiophosphonamidite can be used in excess.* No efforts were made to separate the four diastereomers. **^{1}H NMR** (400 MHz, $CDCl_3$) δ 9.22 (br. s, 1H, NH), 8.33, 8.29, 8.21 (3 x d, 1H, *J* = 7.6, 7.5, 7.6 Hz, H-6), 7.93 (app. d, 2H, *J* = 7.7 Hz, *o*-Bz), 7.66 – 7.55 (m, 2H, H-5, *p*-Bz), 7.53 – 7.46 (m, 2H, *m*-Bz), 6.25, 6.23, 6.21 (3 x d, *J* = 5.2, 5.0, 5.0 Hz, H-1'), 5.61 (t, *J* = 5.3 Hz, H-2'), 5.53 – 5.44 (m, 2H, H-2', H-3'), 5.41, 5.41 (2 x t, 1H, *J* = 5.5, 5.6 Hz, H-3'), 4.52 – 4.43 (m, 1H H-5$_A$'), 4.43 – 4.36 (m, 1H, H-4'), 4.37 – 4.25 (m, 1H, H-5$_B$'), 3.82, 3.81, 3.81, 3.80 (4 x d, 3H, $^3J_{HP}$ = 11.6, 11.6, 11.6, 11.6 Hz, P-OCH_3), 3.38 – 3.22 (m, 2H, SCH_2 CNE) 3.17 – 3.03 (m, 2H, P-CH_2-P), 2.88 – 2.80 (m, 2H, CH_2CN), 2.11 – 2.08 (m, 6H, CH_3 Ac), 1.56, 1.55, 1.54 (3 x s, 9H, CH_3 ^{t}Bu); **^{13}C NMR** (101 MHz, $CDCl_3$) δ 169.64, 169.61, 169.59, 169.57, 169.53, 169.47, 169.40 (C=O Ac), 162.9 (C-4), 155.1 (C-2), 145.8, 145.6, 145.3, 145.2 (C-6), 133.1 (*p*-Bz), 133.0 (*ipso*-Bz), 128.9 (*m*-Bz), 127.8 (*o*-Bz), 117.82, 117.78, 117.75 (C_q SCNE), 97.7 (C-5), 88.5, 88.4, 88.2 (C-1'), 85.4, 85.3, 85.23, 85.22 (4 x d, $^2J_{CP}$ = 9.0, 8.7, 8.8, 8.7 Hz, C_q ^{t}Bu), 80.61, 80.58, 80.4 (3 x d, $^3J_{CP}$ = 9.9, 9.9, 10.0 Hz, C-4'), 73.53, 73.51, 73.4, 73.3 (C-2'), 69.71, 69.66, 69.6 (C-3'), 63.94, 63.85, 63.63, 63.58 (4 x d, $^2J_{CP}$ = 6.9, 7.4, 4.9, 5.6 Hz, C-5'), 53.20, 53.17 (2 x d, $^2J_{CP}$ = 6.7, 6.5 Hz, P-OMe), 40.31 (dd, $^1J_{CP}$ = 138.6, 78.8 Hz, P-$C_α$-P) , 40.26 (dd, $^1J_{CP}$ = 137.6, 78.5 Hz, P-$C_α$-P), 40.21 (dd, $^1J_{CP}$ = 138.1, 79.0 Hz, P-$C_α$-P), 30.35, 30.32, 30.28 (Me ^{t}Bu), 29.98, 29.94, 29.91, 29.8 (4 x d, $^2J_{CP}$ = 3.3, 3.7 3.5, 3.4 Hz, SCH_2 CNE), 20.51 (Me Ac), 20.45 (Me Ac), 19.8, 19.7 (2 x d, $^3J_{CP}$ = 2.7, 2.8 Hz, CH_2CN); **^{31}P NMR** (162 MHz, $CDCl_3$) δ 98.24, 98.17, 98.09, 98.05, 97.92, 97.89, 97.86, 97.82 (PS2), 13.11, 13.04, 13.00, 12.98, 12.97 (PO). **HRMS** (ESI) m/z: $[M+H]^+$ calcd for $C_{29}H_{39}N_4O_{11}P_2S_2$ 745.1527, found 745.1526.

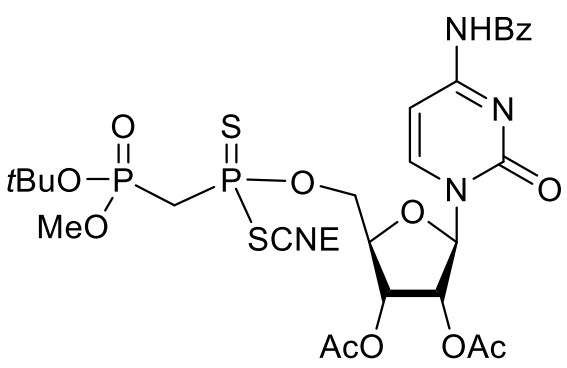

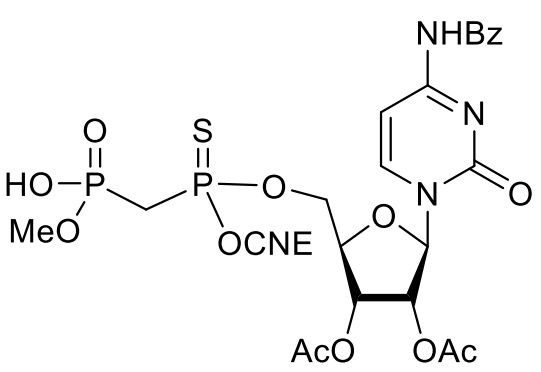

me-CDP-αS triester (21a) – To a solution of cytidine tetraester **20a** (117 mg, 0.16 mmol, 1.0 eq.) in hexafluoroisopropanol (1.25 mL, 0.13 M) was added a 6 M hydrochloric acid in dioxane solution (1/1; v/v; aq. HCl/dioxane, 40.1 μL, 0.24 mmol, 1.5 eq.). After complete acidolytic removal of the *tert*-butyl group (0.5 - 1 hour), the mixture was evaporated to dryness. Successive coevaporation of the crude with toluene (3 x), chloroform (3 x) and acetonitrile (1 x) afforded triester **21a** as a crystalline white foam (108 mg, 0.16 mmol,

quant.). The material was carried on without further purification. **^{31}P NMR** (162 MHz, acetone-d_6 capillary) δ 88.14 (PS), 20.19, 19.79 (PO).

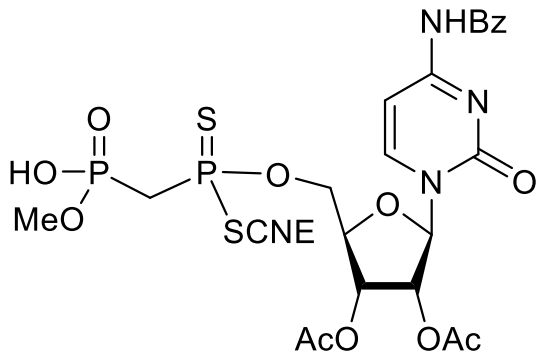

me-CDP-αS$_2$ triester (21b) – me-CDP-αS$_2$ tetraester **20b** (56 mg, 75.0 µmol, 1.0 eq.) was dissolved in hexafluoroisopropanol (0.75 mL, 0.1 M) and treated with 6 M aq. HCl in dioxane (1/1; v/v, 15 µL, 90 µmol, 1.2 eq.). The solution was stirred for 1 hour and concentrated to dryness. The crude was coevaporated thrice with toluene, thrice with chloroform and once with anhydrous acetonitrile to afford me-CDP-αS$_2$ phosphonic acid triester **21b** as a crystalline white foam (52 mg, 75.0 µmol, quant.). The material was carried on without further purification. *Resonances marked with an asterisk * correspond to the minor P-diastereomer.* **^{31}P NMR** (121 MHz, acetone-d_6 capillary) δ 97.42 (d, J = 12.6 Hz), 97.05 (d, J = 12.5 Hz, PS$_2$), 16.73 (br. s, PO).

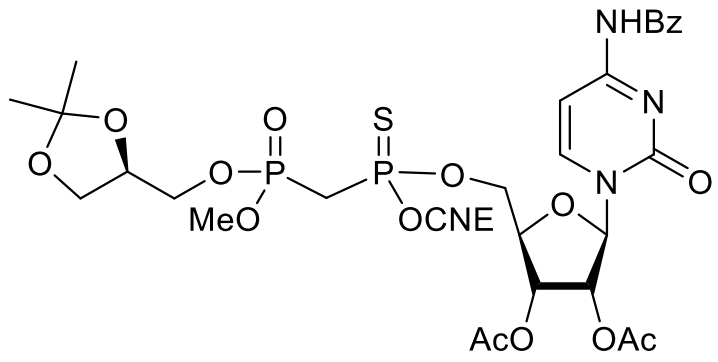

***sn*-3 me-CDP-αS-Gro tetraester (25a) –** To a dry solution of bisphosphonic triester **21a** (138 mg, 0.205 mmol, 1.0 eq.) in anhydrous acetonitrile (1.0 mL, 0.2 M) was added triethylamine, (28.6 µL, 0.205 mmol, 1.0 eq.), 2,6-lutidine (237.8 µL, 2.05 mmol, 10.0 eq.), (*S)*-solketal **22** (38.1 µL, 0.308 mmol, 1.5 eq.) and Wada's reagent (307 mg, 0.614 mmol, 3.0 eq.) consecutively. After completion (2 hours), the mixture was quenched with sodium acetate in methanol (1 M, 1 mL), stirred for 10 minutes and poured into a separatory funnel containing 1 M aq. HCl. The aqueous phase was extracted thrice with DCM and the pooled organic layers were dried over Na_2SO_4, filtered and concentrated *in vacuo*. Purification by silica gel column chromatography (0 → 5 → 10 → 15 → 20 → 25 → 30 → 35 → 40 → 45 → 50% acetone in DCM) afforded title *sn*-3 me-CDP-αS-Gro tetraester **25a** as a crystalline white solid (mixture of four P-diastereomers, 106 mg, 0.135 mmol, 84%). No efforts were made to resolve the four diastereomers, as one of the phosphonates loses chirality in the subsequent demethylation step. **^{1}H NMR** (500 MHz, $CDCl_3$) δ 9.25 (br. s, 1H, NH), 8.23, 8.23, 8.20, 8.16 (4 x d, 1H, J = 7.5, 7.6, 7.6, 7.6 Hz, H-6), 7.94 (app. d, 2H, J = 7.7 Hz, *o*-Bz), 7.62 – 7.56 (m, 2, H-5', *p*-Bz), 7.50 (app. t, 2H, J = 7.7 Hz, *m*-Bz), 6.23 – 6.17 (m, 1H, J = 4.5 Hz, H-1'), 5.53 – 5.44 (m, 2H, H-2', H-3'), 5.42 (t, J = 5.4 Hz, H-3'), 4.58 – 4.30 (m, 6H, H-4', H-5', H-2'', OCH_2 CNE), 4.20 – 4.04 (m, 3H, H-1$_A$'', H-3''), 3.86, 3.85, 3.84 (3 x d, $^3J_{HP}$ = 11.4, 11.5, 11.5 Hz, P-OCH_3), 3.84 – 3.78 (m, 1H, H-1$_B$''), 3.06 – 2.91 (m, 2H, P-CH_2-P), 2.89 – 2.76 (m, 2H, CH_2CN), 2.10 (s, 6H, CH_3 Ac), 1.43, 1.42 (2 x s, 3H, CH_3 CMe_2), 1.35*, 1.34 (2 x s, 3H, CH_3' CMe_2); **^{13}C NMR** (126 MHz, $CDCl_3$) δ 169.7, 169.6, 169.50, 169.46 (C=O Ac), 162.9 (C-4), 154.9 (C-2), 145.0 (C-6), 133.1 (*p*-Bz), 133.0 (*ipso*-Bz), 128.9 (*m*-Bz), 127.8 (*o*-Bz), 116.7, 116.94, 116.90, 116.87 (C_q CNE), 109.90, 109.87 (C_q CMe_2), 97.5 (C-5), 88.6, 88.40, 88.37 (C-1'), 80.7, 80.4 (2 x d, $^3J_{CP}$ = 8.3, 9.1 Hz, C-4'), 74.3, 74.24, 74.19 (C-2''), 73.61, 73.59, 73.55, 73.53 (C-2'), 69.57, 69.53, 69.45 (C-3'), 67.02, 67.00, 66.98, 66.95, 66.90, 66.86 (C-3''), 65.82, 65.80 (C-1''), 65.3, 65.2, 64.9 (3 x d, $^2J_{CP}$ = 5.2, 5.4, 5.5 Hz, C-5'), 62.02, 62.00, 61.93, 61.87 (4 x $^2J_{CP}$ = 5.9, 5.1, 4.8, 4.8 Hz, OCH_2 CNE), 53.48, 53.43, 53.37, 53.33, 53.32 (P-OMe), 33.7* (dd, $^1J_{CP}$ = 137.1, 107.5 Hz, P-C$_α$-P), 33.45, 33.41 (2 x dd, app. $^1J_{CP}$ = 138, 108 Hz, P-C$_α$-P), 26.71, 26.67 (Me CMe_2), 25.17 (Me' CMe_2), 20.50 (Me Ac), 20.45 (Me Ac), 19.61, 19.59, 19.51, 19.49 (4 x d, $^3J_{CP}$ = 7.8, 8.3, 8.2, 8.1 Hz, CH_2CN); **^{31}P NMR** (202 MHz, $CDCl_3$) δ 86.91, 86.89, 86.87, 86.85, 86.65, 86.63, 86.60, 86.58 (PS), 19.42, 19.40, 19.38, 19.37, 19.16, 19.15, 19.13 (PO); **HRMS** (ESI) m/z**:** [M+H]$^+$ calcd for $C_{31}H_{41}N_4O_{14}P_2S$ 787.1809, found 787.1780.

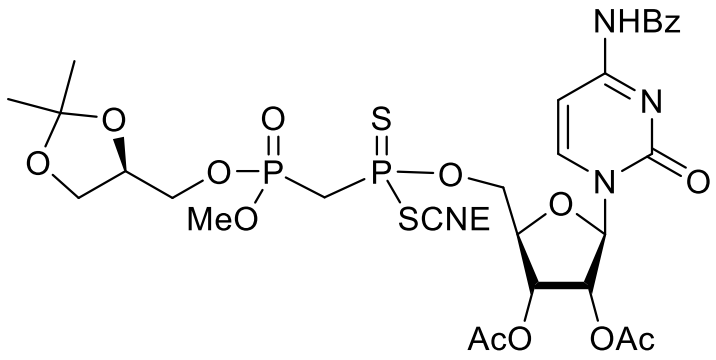

***sn*-3 me-CDP-αS$_2$-Gro tetraester (25b) –** me-CDP-αS$_2$ triester **21b** (70 mg, 0.10 mmol, 1.0 eq.) was desiccated from entrapped water by azeotroping thrice with dry acetonitrile, placed under an argon atmosphere and subsequently dissolved in anhydrous acetonitrile (0.5 mL, 0.2 M). Hereto, triethylamine (14.5 µL, 0.10 mmol, 1.0 eq.), 2,6-lutidine (118.3 µL, 1.0 mmol, 10.0 eq.), *S*-solketal **22** (14.2 µL, 0.123 mmol, 1.2 eq.) and Wada's reagent (PyNTP, 127 mg, 0.26 mmol, 2.5 eq.) were added consecutively. Monitoring of the reaction by ^{31}P NMR spectroscopy indicated complete phosphonodiester condensation after 2 hours. The reaction mixture was quenched by addition of methanolic sodium acetate (1 M, 1 mL), stirred for an additional 10 minutes and poured into 1 M aq. HCl. The aqueous layer was extracted thrice with DCM and the resulting organic layers were combined, dried over Na_2SO_4, filtered and concentrated *in vacuo*. Purification by silica gel column chromatography (0 → 5 → 10 → 15 → 20 → 25 → 30 → 35 → 40 → 45 → 50% acetone in DCM) afforded title *sn*-3 me-CDP-αS$_2$-Gro tetraester **25b** as a crystalline white foam (mixture of 4 P-diastereomers, 48 mg, 59.8 µmol, 59%). The product was isolated as an inconsequential mixture of four P-diastereomers and no efforts were undertaken to resolve them since both phosphonates loose chirality in subsequent deprotection steps. **^{1}H NMR** (500 MHz, $CDCl_3$) δ 9.14 (br. s, 1H, NH), 8.28 – 8.17 (m, 1H, H-6), 7.93 (app. d, 2H, J = 7.7 Hz, *o*-Bz), 7.63 – 7.56 (m, 2H, H-5', *p*-Bz), 7.51 (app. t, 2H, J = 7.5 Hz, *m*-Bz), 6.26 – 6.21 (m, 1H, H-1'), 5.53 – 5.44 (m, 2H, H-2', 3'), 5.43 (t, J = 5.1 Hz, H-3'), 4.47 (app. t, 1H, J = 9.7 Hz, H-5$_A$'), 4.41 – 4.33 (m, 2H, H-4', H-2''), 4.29 (app. td, 1H, J = 9.4, 5.0 Hz, H-5$_B$'), 4.24 – 4.06 (m, 3H, H-1$_A$'', H-3''), 3.89 – 3.78 (m, 4H, H-1$_B$'', P-OCH_3), 3.38 – 3.09 (m, 4H, P-CH_2-P, SCH_2 CNE), 2.83 (t, 2H, J = 6.5 Hz, CH_2CN), 2.11, 2.10 (2 x s, 6H, CH_3 Ac), 1.44, 1.43 (2 x s, 3H, CH_3 CMe_2), 1.35 (s, 3H, CH_3' CMe_2); **^{13}C NMR** (126 MHz, $CDCl_3$) δ 169.8, 169.70, 169.65, 169.60, 169.58 (C=O Ac), 166.6 (C=O Bz, *detected with HMBC*), 162.9 (C-4), 154.9 (C-2, *detected with HMBC*), 145.6 (C-6), 133.3 (*p*-Bz), 133.1 (*ipso*-Bz), 129.0 (*m*-Bz), 127.8 (*o*-Bz), 117.78, 117.75 (C_q CNE), 110.1 (C_q CMe_2), 97.7 (C-5, *verified with HSQC*), 88.3, 88.21, 88.16, (C-1'), 80.7, 80.50, 80.49 (3 x d, $^3J_{CP}$ = 10.0, 9.9, 10.1, C-4'), 74.40, 74.37, 74.35, 74.31, 74.31, 74.28, 74.23 (C-2''), 73.64, 73.46, 73.42 (C-2'), 69.79, 69.74 (C-3'), 67.22, 67.20, 67.08, 67.07 (4 x d, $^2J_{CP}$ = 6.7, 6.7, 6.6, 6.5 Hz, C-3''), 65.93, 65.90 (C-1''), 64.11, 64.09, 64.07, 64.05, 63.81, 63.77 (C-5'), 53.63, 53.59, 53.49, 53.44 (4 x d, $^3J_{CP}$ = 6.2, 5.7, 5.7, 6.2 Hz, P-OMe), 39.2 (dd, $^1J_{CP}$ = 135.4, 79.7, P-C$_α$-P), 30.28, 30.26, 30.23, 30.20 (app. $^2J_{CP}$ = 4 Hz, SCH_2 CN), 26.81, 26.79 (Me CMe_2), 25.28 (Me' CMe_2), 20.61 (Me Ac),

20.55 (Me Ac), 19.8 (CH_2CN); **^{31}P NMR** (202 MHz, $CDCl_3$) δ 97.84, 97.81, 97.80, 97.77, 97.45, 97.42, 97.41, 97.38 (PS_2), 18.98, 18.93, 18.90, 18.85, 18.66, 18.62, 18.56 (PO); **HRMS** (ESI) m/z: $[M+H]^+$ calcd for $C_{31}H_{41}N_4O_{14}P_2S_1$ 787.1810, found 787.1800.

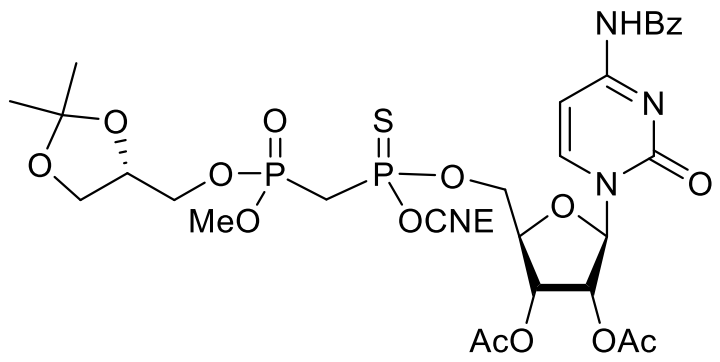

***sn*-1 me-CDP-αS-Gro tetraester (26a) –** Bisphosphonic triester **21a** (144 mg, 0.21 mmol, 1.0 eq.) was coevaporated thrice with anhydrous acetonitrile and dissolved in anhydrous acetonitrile (1.0 mL, 0.21 M), whereto, triethylamine (29.9 µL, 0.21 mmol, 1.0 eq.), 2,6-lutidine (248.4 µL, 2.1 mmol, 10.0 eq.), (*R)*-solketal **23** (39.8 µL, 0.32 mmol, 1.5 eq.) were added. Subsequently, Wada's reagent (320 mg, 0.64 mmol, 3.0 eq.) was added, the mixture was stirred overnight and quenched by the addition of sodium acetate in methanol (1M, 1 mL). After stirring for an additional 10 minutes, the mixture was diluted with 1.0 M aq. HCl , extracted with DCM and the combined organic layers were dried over Na_2SO_4, filtered and concentrated *in vacuo*. Purification by flash column chromatography (0 → 5 → 10 → 15 → 20 → 25 → 30 → 35 → 40 → 45 → 50% acetone in DCM) afforded *sn*-1 me-CDP-αS-Gro tetraester **26a** as a crystalline white foam (mixture of four P-diastereomers, 128 mg, 0.16 mmol, 76%). No efforts were made to resolve the diastereomers since one phosphonate loses chirality in the subsequent demethylation step. **^{1}H NMR** (500 MHz, $CDCl_3$) δ 9.15 (br. s, 1H, NH), 8.23, 8.21, 8.19, 8.16 (4 x d, 1H, *J* = 7.5, 7.6, 7.6, 7.6 Hz, H-6), 7.93 (app. d, 2H, *J* = 8.0 Hz, *o*-Bz), 7.63 – 7.56 (m, 2, H-5', *p*-Bz), 7.51 (app. t, 2H, *J* = 7.7 Hz, *m*-Bz), 6.23 – 6.19 (m, 1H, H-1'), 5.53 – 5.44 (m, 2H, H-2', H-3'), 5.42 (t, *J* = 5.4 Hz, H-3'), 4.57 – 4.31 (m, 6H, H-4', H-5$_A$', H-5$_B$', H-2'', OCH_2 CNE), 4.21 – 4.05 (m, 3H, H-1$_A$'', H-3''), 3.87, 3.85, 3.84, 3.83 (4 x d, 3H, $^3J_{HP}$ = 11.5, 11.7, 11.5, 11.5 Hz, P-OCH_3), 3.82 – 3.79 (m, 1H, H-1$_B$''), 3.05 – 2.89 (m, 2H, P-CH_2-P), 2.88 – 2.75 (m, 2H, CH_2CN), 2.10 (s, 6H, CH_3 Ac), 1.44, 1.43 (2 x s, 3H, CH_3 CMe_2), 1.35, 1.34 (2 x s, 3H, CH_3' CMe_2); **^{13}C NMR** (126 MHz, $CDCl_3$) δ 169.7, 169.63, 169.62, 169.59, 169.58,169.57, 169.5 (C=O Ac), 162.9 (C-4), 155.0 (C-2), 145.1 (C-6), 133.2 (*p*-Bz), 133.0 (*ipso*-Bz), 129.0 (*m*-Bz), 127.8 (*o*-Bz), 116.96, 116.94, 116.90, 116.86 (C_q CNE), 109.9 (C_q CMe_2), 97.4 (C-5, *verified with HSQC and HMBC*), 88.6, 88.4, (C-1'), 80.78, 80.75, 80.53 (3 x d, $^3J_{CP}$ = 8.4, 8.6, 8.5 Hz, C-4'), 74.38, 74.36, 74.34, 74.32, 74.31, 74.28, 74.23 (C-2''), 73.68, 73.65, 73.63, 73.59 (C-2'), 69.63, 69.59, 69.54, 69.51 (C-3'), 67.07, 67.02, 66.98 (3 x d, $^2J_{CP}$ = 6.6, 6.6, 6.4 Hz, C-3''), 65.87, 65.85 (C-1''), 65.4, 65.3 (2 x d, $^2J_{CP}$ = 5.4, 5.6 Hz, C-5'), 64.99, 64.97, 64.94, 64.92 (C-5'), 62.1, 62.00, 61.99, 61.9 (4 x d, $^2J_{CP}$ = 4.9, 4.8, 4.8, 4.8 Hz, OCH_2 CNE), 53.51, 53.42, 53.37 (3 x d, $^3J_{CP}$ = 6.7, 6.6, 6.7 Hz, P-OMe), 33.8 (dd, $^1J_{CP}$ = 138.2, 107.6 Hz, P-C_α-P), 33.6 (dd, $^1J_{CP}$ = 136.9, 107.8 Hz, P-C_α-P), 26.8, 26.7 (Me CMe_2), 25.2 (Me' CMe_2), 20.6 (Me Ac), 20.5 (Me Ac), 19.70, 19.67, 19.64, 19.61, 19.5 (CH_2CN); **^{31}P NMR** (202 MHz, $CDCl_3$) δ 86.94, 86.92, 86.89, 86.88, 86.67, 86.65, 86.56, 86.55 (PS), 19.38, 19.36, 19.19, 19.17, 19.13, 19.12 (PO); **HRMS** (ESI) m/z: $[M+H]^+$ calcd for $C_{31}H_{41}N_4O_{14}P_2S$ 787.1810, found 787.1805.

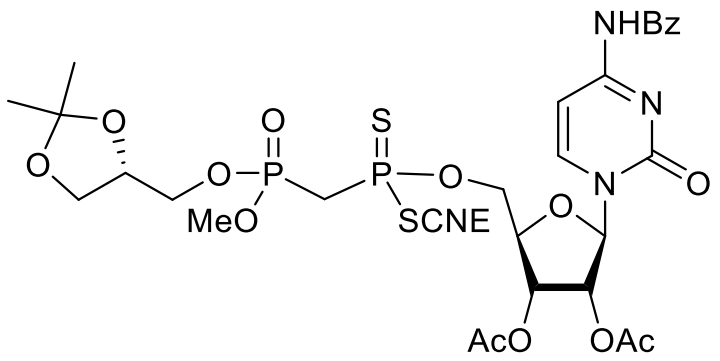

***sn*-1 me-CDP-αS$_2$-Gro tetraester (26b) -** me-CDP-αS$_2$ triester **21b** (85 mg, 0.12 mmol, 1.0 eq.) was desiccated by evaporation of added acetonitrile, placed under an argon atmosphere and dissolved in anhydrous acetonitrile (0.6 mL, 0.2 M). Hereto, triethylamine (17.1 µL, 0.12 mmol, 1.0 eq.), 2,6-lutidine (143.3 µL, 1.2 mmol, 10.0 eq.), (*R*)-solketal **23** (18.4 µL, 0.148 mmol, 1.2 eq.) and Wada's reagent (PyNTP, 154 mg, 0.31 mmol, 2.5 eq.) were added successively. After 2 hours the reaction mixture was quenched by adding 1 M methanolic sodium acetate (1 mL), stirred for an additional 10 minutes and poured into a separatory funnel containing 1 M aq. HCl. The aqueous phase was extracted thrice with DCM and the resulting organic layers were combined, dried over Na_2SO_4, filtered and concentrated under reduced pressure. Purification by silica gel column chromatography (0 → 5 → 10 → 15 → 20 → 25 → 30 → 35 → 40 → 45 → 50% acetone in DCM) furnished title *sn*-1 me-CDP-αS$_2$-Gro tetraester **26b** a a crystalline white foam (mixture of 4 P-diastereomers, 78 mg, 97.2 µmol, 79%). The product was isolated as an inconsequential mixture of four diastereomers. No efforts were undertaken to separate them, since both phosphonates lose chirality in the subsequent deprotection steps. **^{1}H NMR** (500 MHz, $CDCl_3$) δ 9.37 (br. s, 1H, NH), 8.28 – 8.17 (m, 1H, H-6), 7.94 (br. d, 2H, *J* = 7.7 Hz, *o*-Bz), 7.65 – 7.57 (m, 2, H-5', *p*-Bz), 7.51 (app. t, 2H, *J* = 7.6 Hz, *m*-Bz), 6.27 – 6.21 (m, 1H, H-1'), 5.54 – 5.46 (m, 2H, H-2', 3'), 5.43 (app. t, *J* = 5.4 Hz, H-3'), 4.48 (app. t, 1H, app. *J* = 9.7 Hz, H-5$_A$'), 4.41 – 4.34 (m, 2H, H-4', H-2''), 4.34 – 4.26 (m, 1H, H-5$_B$'), 4.23 – 4.05 (m, 3H, H-1$_A$'', H-3''), 3.90 – 3.79 (m, 4H, H-1$_B$'', P-OCH_3), 3.38 – 3.11 (m, 4H, P-CH_2-P, SCH_2 CNE), 2.83 (t, 2H, *J* = 6.8 Hz, CH_2CN), 2.11, 2.10, 2.09 (3 x s, 6H, CH_3 Ac), 1.43 (s, 3H, CH_3 CMe_2), 1.35 (s, 3H, CH_3' CMe_2);**^{13}C NMR** (126 MHz , $CDCl_3$) δ 169.79, 169.68, 169.67, 169.63, 169.60, 169.57, 165,54 (C=O Ac), 166.8 (C=O Bz, *detected with HMBC*) 162.9 (C-4), 154.9 (C-2), 145.6 (C-6), 133.3 (*p*-Bz), 133.0 (*ipso*-Bz), 129.0 (*m*-Bz), 127.8 (*o*-Bz), 117.8 (C_q CNE), 110.1 (C_q CMe_2), 97.6 (C-5, *signal verified with HSQC*), 88.2, 88.1 (C-1'), 80.7, 80.5 (2 x d, $^3J_{CP}$ = 9.9, 10.1 Hz, C-4'), 74.46, 74.39, 74.35, 74.32, 74.27 (C-2''), 73.64, 73.61, 73.46, 73.42 (C-2'), 69.77, 69.74 (C-3'), 67.21, 67.16, 67.08, 67.0 (4 x d, $^2J_{CP}$ = 6.7, 6.7, 6.6, 6.5 Hz, C-3''), 65.91, 65.89 (C-1''), 64.12, 64.08 (2 x d, $^2J_{CP}$ = 5.7, 5.7 Hz, C-5') , 63.79, 63.77, 63.75 (br. signals, C-5'), 53.7, 53.6, 53.5, 53.4 (4 x d, $^2J_{CP}$ = 6.7, 6.7, 7.0, 6.7 Hz, P-OMe), 39.23 (dd, $^1J_{CP}$ = 136.0, 80.0 Hz, P-C_α-P), 30.29, 30.26 30.20 (br. signals, SCH_2 CNE), 26.81, 26.80, 26.77 (Me CMe_2), 25.3 (Me' CMe_2), 20.60 (Me Ac), 20.54 (Me Ac), 19.86, 19.82, 19.80 (br. signals, CH_2CN); **^{31}P NMR** (202 MHz, $CDCl_3$) δ 97.24, 97.20, 96.85, 96.80, 96.75, 96.71 (PS_2), 18.36, 18.32, 18.28, 18.24, 18.13, 18.08, 18.02, 17.98 (PO); **HRMS** (ESI) m/z: $[M+H]^+$ calcd for $C_{31}H_{41}N_4O_{14}P_2S_1$ 787.1810, found 787.1805.

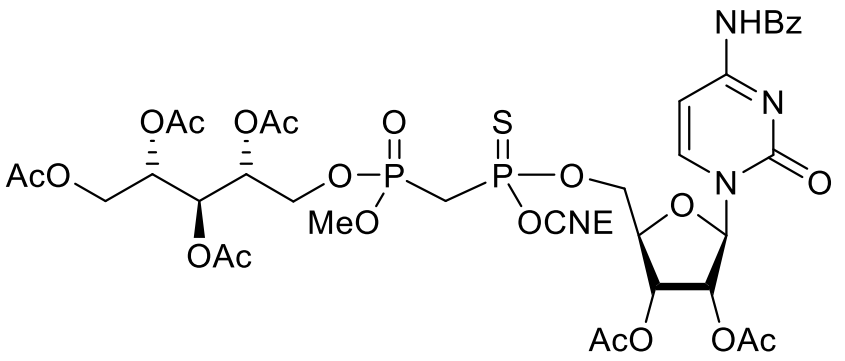

me-CDP-αS-ribitol tetraester (27a) - Cytidine bisphosphonic triester **21a** (119 mg, 0.18 mmol, 1.0 eq.) and tetra-*O*-acetyl D-ribitol **24** (68 mg, 0.21 mmol, 1.2 eq.) were coevaporated thrice with acetonitrile and then dissolved in anhydrous acetonitrile (0.9 mL, 0.2 M). Hereto, triethylamine (24.6 µL, 0.18 mmol, 1.0 eq.), 2,6-lutidine (205.3 µL, 1.8 mmol, 10.0 eq.) and Wada's reagent (221 mg, 0.44 mmol, 2.5 eq.) were added consecutively and reaction progression was monitored using ^{31}P NMR spectroscopy, which indicated complete phosphonodiester condensation after 3 hours. The reaction mixture was quenched by the addition of 1 M methanolic NaOAc (1 mL), stirred for 10 minutes and poured into a separatory funnel containing 1 M aq. HCl. The material obtained by extracting thrice with DCM, drying over Na_2SO_4, filtering and concentrating *in vacuo* was purified by flash column chromatography (0 → 2.5 → 5 → 7.5 → 10 → 15 → 20 → 25 → 30 → 35 → 40 → 50% acetone in DCM) to furnish title me-CDP-αS-ribitol tetraester **27a** as a white crystalline foam (mixture of 4 P-diastereomers, 126 mg, 0.129 mmol, 73%). No effort was made to separate the diastereomers, since one phosphonate loses chirality upon demethylation. **^{1}H NMR** (400 MHz, CDCl3) δ 8.92 (br. s, 1H, NH), 8.25 – 8.10 (m, 1H, H-6), 7.91 (app. d, *J* = 7.4 Hz, 2H, *o*-Bz), 7.64 – 7.53 (m, 2H, H-5, *p*-Bz), 7.52 (app. t, 2H, *J* = 7.6 Hz, *m*-Bz), 6.27 – 6.20 (m, 1H, H-1'), 5.53 – 5.34 (m, 3H, H-2', H-3', H-3''), 5.35 – 5.19 (m, 2H, H-2'', H-4''), 4.58 – 4.01 (m, 9H, H-4', H-5', H-1'', H-5'', OCH_2 CNE),3.87, 3.85, 3.83, 3.81 (4 x d, 3H, $^{3}J_{HP}$ = 11.6, 11.5, 11.4, 11.4 Hz, P-OCH_3), 3.04 – 2.74 (m, 4H, P-CH_2-P, CH_2CNE) 2.16 – 2.02 (m, 18H, CH_3 Ac); **^{13}C NMR** (126 MHz, $CDCl_3$) δ 170.65, 170.63, 170.06, 169.95, 169.93, 169.88, 169.78, 169.63, 169.66, 169.53, (C=O Ac), 166.5 (C=O Bz, *signal detected with HMBC*), 154.5 (C-2, *signal detected with HMBC*), 162.7 (C-4), 145.1 (C-6), 133.3 (*p*-Bz), 130.0 (*m*-Bz), 127.8 (*o*-Bz), 116.94, 116.86, (C_q CNE), 97.5 (C-5, *verified with HSQC*), 88.58, 88.35, 88.26 (C-1') 80.91, 80.83, 80.66, 80.57 (C-4'), 73.77, 73.72, 73.67, 70.14, 70.08, 69.77, 69.71, 69.67, 69.58, 68.99, 68.95, 68.89, 68.76, 68.72, 68.69 (C-2', C-3', C-2'', C-3'' & C-4''), 65.59, 65.52, 65.28, 65.05, 64.30, 64.24, 64.17, 64.11, 62.24, 62.19, 62.13, 62.01, 61.74 (C-5', C-5'', OCH_2 CNE), 53.58, 53.65, 53.76 (P-OMe), 34.8 – 32.5 (m, P-C_α-P, *also detected with HSQC*), 21.01, 20.97, 20.86, 20.78, 20.64, 20.58 (Me Ac), 19.77, 19.69, 19.61 (CH_2CN); **^{31}P NMR** (162 MHz, $CDCl_3$) δ 86.73, 86.72, 86.68, 86.66, 86.62, 86.42, 86.40, 86.32 (PS), 19.72, 19.61, 19.50 (PO); **HRMS** (ESI) m/z: $[M+H]^+$ calcd for $C_{38}H_{49}N_4O_{20}P_2S$ 975.2131, found 975.2122.

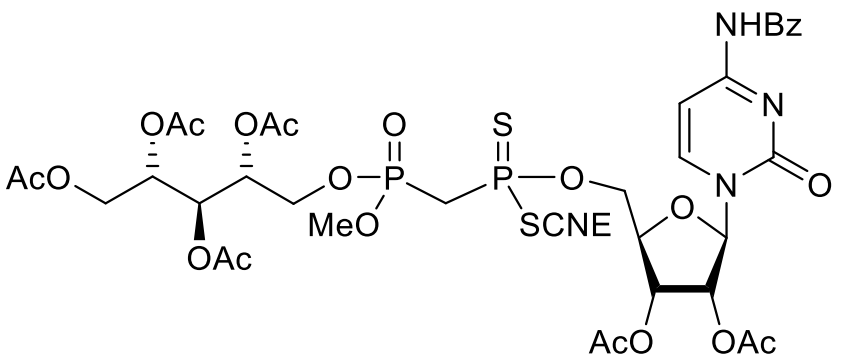

me-CDP-αS$_2$-ribitol tetraester (27b) – me-CDP-αS$_2$ phosphonic acid **21b** (52 mg, 75.0 µmol, 1.0 eq.) and freshly prepared tetra-*O*-acetyl ribitol **24** (29 mg, 90.0 µmol, 1.2 eq.) were coevaporated thrice with and then dissolved in anhydrous acetonitrile (0.4 mL, 0.2 M). Hereto triethylamine (10.4 µL,), 2,6-lutidine (87 µL, 10 eq.) and PyNTP (93.6 mg,) were added consecutively, the mixture stirred for 2 hours and subsequently quenched by the addition of 1 M methanolic sodium acetate (1 mL). After stirring for an additional 10 minutes the mixture was diluted with 1 M aq. HCl, extracted thrice with DCM and the combined organic extracts were dried over Na_2SO_4, filtered and concentrated *in vacuo*. Purification by flash column chromatography (0 → 5 → 10 → 15 → 20 → 30% acetone in DCM) afforded title me-CDP- αS$_2$-ribitol tetraester **27b** as a white crystalline foam (50 mg, 50.5 µmol, 67%). **1H NMR** (500 MHz, $CDCl_3$) δ 9.03 (br. s, 1H, NH), 8.27 – 8.20 (m, 1H, H-6), 7.92 (app. d, 2H, *J* = 7.7 Hz, *o*-Bz), 7.64 – 7.55 (m, 2H, H-5, *p*-Bz), 7.51 (app. t, 2H, *J* = 7.6 Hz, *m*-Bz), 6.25 – 6.18 (m, 1H), 5.56 – 5.35 (m, 2H, H-2', H-3', H-3''), 5.35 – 5.18 (m, 2H, H-2'', H-4''), 4.52 – 4.09 (m, 7H, H-4', H-5', H-1'', H-5''), 3.94 – 3.75 (m, 3H, P-OCH_3), 3.37 – 3.09 (m, 4H, P-CH_2-P, SCH_2 CNE), 2.89 – 2.79 (m, 2H, CH_2CN), 2.15 – 2.06 (m, 18H, CH_3 Ac); **^{13}C NMR** (126 MHz, $CDCl_3$) δ 170.63, 170.61, 170.08, 170.04, 169.93, 169.85, 169.80, 169.77, 169.6, 169.5 (C=O Ac), 166.4 (C=O Bz, *signal detected with HMBC*), 162.8 (C-4), 155.1 (C-2, *signal verified with HSQC*), 145.6 (C-6), 133.30, 133.27 (*p*-Bz), 129.1 (*m*-Bz), 127.8 (*o*-Bz), 117.81, 117.78 (C_q CNE), 97.6 (C-5, *signal verified with HSQC*), 88.53, 88.45, 88.37, 88.33 (C-1'), 80.71, 80.68 (2 x d, $^{3}J_{CP}$ = 9.4, 10.0 Hz, C-4'), 80.54, 80.47 (C-4'), 73.64, 73.59, 73.59, 73.41, 70.10, 69.81, 69.76, 69.70, 69.67, 69.63, 69.58, 69.06, 69.03, 69.00, 68.86, 68.81 (C-2', C-3', C-2'', C-3'', C-4''), 64.24, 64.22, 64.19, 64.13 (C-5', C-5''), 61.8 (C-1''), 53.80, 53.76, 53.71, 53.65, 53.61, 53.59, 53.56, 53.53 (P-OMe), 39.21 (dd, *J* = 135.5, 79.3 Hz, P-C_α-P), 39.16 (dd, *J* = 138.7, 79.1 Hz, P-C_α-P), 30.32, 30.29, 30.25, 30.22, 30.19, 30.16, 30.13 (SCH_2 CNE), 19.8 (br. signal, CH_2CN); **^{31}P NMR** (202 MHz, $CDCl_3$) δ 97.00, 96.96, 96.94, 96.90, 96.87, 96.83, 96.54, 96.52, 96.50, 96.49, 96.45, 96.40, 96.32 (PS$_2$), 18.57, 18.53, 18.50, 18.46, 18.42, 18.38, 18.33 (PO); **HRMS** (ESI) m/z: $[M+H]^+$ calcd for $C_{38}H_{49}N_4O_{19}P_2S_2$ 991.1902, found 991.1897.

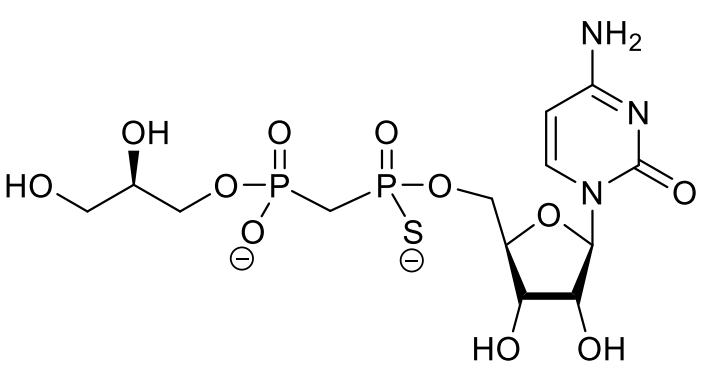

***sn*-3 me-CDP-αS-Gro (28a)–** *sn*-3 me-CDP-αS-Gro tetraester **25a** (118 mg, 150.0 µmol, 1.0 eq.) was treated with PhSH/TEA/MeCN (1/2/2; v/v/v, [1.96], 1.5 mL, 20 eq.), until ^{31}P NMR spectroscopy indicated complete demethylation and partial de-cyanoethylation (2 hours). Subsequently, the volatiles were evaporated *in vacuo*, the residue taken up in H_2O, acidified to pH 4 with acetic acid, washed thrice with Et_2O and concentrated *in vacuo*. The crude was dissolved in AcOH/H_2O (2.3/1; v/v, 2.6 mL, 0.05 M) for 3 hours at 40 °C. After concentrating and azeotroping thrice with toluene, the residue was suspended in ammonium hydroxide (28 wt. % NH_4OH in H_2O, 2.6 mL, 0.05 M), stirred overnight at ambient temperature and the clear homogenous mixture was rendered free of excess ammonia by purging under vacuum. Aqueous extractive work-up as described above, except washing once with DCM, afforded a yellow crystalline solid. Purification by gel filtration (HW40-column, 0.15 M aqueous NH_4OAc) and ion-exchange chromatography afforded after lyophilization *sn*-3 me-CDP-αS-Gro **28a** as a crystalline white solid (1:1 mixture of P-diastereomers, NH_4^+-form, 46.2 mg, 87.9 µmol, 59%). No efforts were undertaken to resolve the PS-diastereomers. *Resonances and J-couplings marked with an asterisk * correspond to one of the P-diastereomers.* **^{1}H NMR**

(400 MHz, D_2O, NH_4^+-form) δ 8.06, 8.03* (2 x, 1H, *J* = 7.5, 7.3* Hz, H-6), 6.09 (d, 1H, *J* = 7.6 Hz, H-5), 5.91 (d, *J* = 3.8 Hz, H-1'), 4.32 4.32* (2 x t, 1H, *J*= 5.3, 5.3* Hz, H-3'), 4.29 (dd, 1H, *J* = 5.3, 3.8 Hz, H-2'), 4.27 – 4.11 (m, 3H, H-4', H-5'), 3.92, 3.92* (2 x app. q, *J* = 6.6, 6.6* Hz, H-3_A''), 3.89 – 3.81 (m, 2H, H-2'', H-3_B''), 3.63 (dd, 1H, J_{ABX} = 11.8, 4.4 Hz, H-1_A''), 3.56 (dd, 1H, J_{BAX} = 11.8, 5.8 Hz, H-1_B''), 2.52, 2.52* (2 x dd, 2H, $^2J_{HP}$ = 19.1, 19* 17.2, 17* Hz, P-CH_2-P); **^{13}C NMR** (101 MHz, D_2O) δ 165.1 (C-4), 156.4 (C-2), 142.3, 142.2* (C-6), 96.3 (C-5), 89.5 (C-1'), 82.8 (d, $^3J_{CP}$ = 8.8 Hz, C-4'), 74.3 (C-2'), 70.9 (d, $^3J_{CP}$ = 6.9 Hz, C-2''), 69.3 (C-3'), 65.19, 65.16* (2 x d, $^2J_{CP}$ = 5.3, 5.5* Hz, C-3''), 63.0*, 62.8 (2 x d, $^2J_{CP}$ = 5.7*, 6.1 Hz, C-5'), 62.1, 62.0* (C-1''), 35.88, 35.83* (2 x dd, $^1J_{CP}$ = 125.5, 125.5*, 93.5, 92.9* Hz, P-C_α-P); **^{31}P NMR** (162 MHz, D_2O) δ 69.67 (d, *J* = 12.9 Hz), 69.55* (d, *J* = 12.9 Hz, PS), 16.47 (d, *J* = 12.9 Hz, PO); **HRMS** (ESI) m/z: [M+H]$^+$ calcd for $C_{13}H_{24}N_3O_{11}P_2S$ 492.0601, found 492.0611.

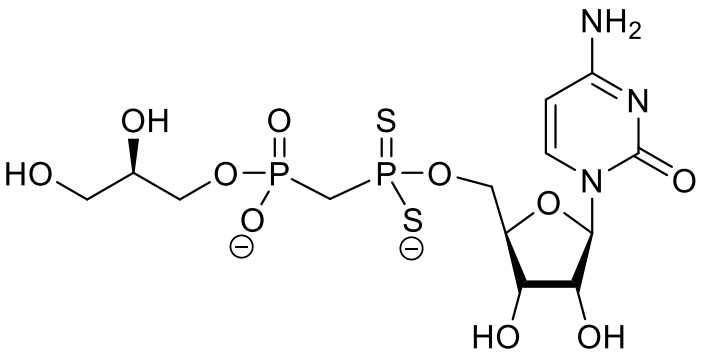

***sn*-3 me-CDP-αS_2 Gro (28b) –** Fully protected *sn*-3 me-CDP-αS_2-Gro tetraester **25b** (45 mg, 56.1 µmol, 1.0 eq.) was dissolved in a mixture of PhSH/MeCN/Et_3N (1/2/2; v/v/v, [1.96], 0.56 mL, 20 eq.) stirred for 3 hours and the volatiles removed *in vacuo*. The residue was taken up in AcOH/H_2O (2.3/1; v/v, 1.1 mL, 0.05 M), heated to 40 °C and stirred at this temperature for 3 hours. Subsequently, the mixture was concentrated to dryness, coevaporated thrice with toluene and thrice with acetonitrile to remove residual acetic acid and moisture, affording a crude colourless oil. The preceding crude was dissolved in anhydrous acetonitrile (0.56 mL, 0.1 M), whereto dry 1,8-Diazabicyclo[5.4.0]undec-7-ene (33.5 µL, 0.224 mmol, 4.0 eq.) was added, followed after 1 hour by the addition of aqueous ammonia (30 wt. %, 0.56 mL, total volume 1.1 mL, 0.05 M). After stirring overnight, excess ammonia was purged *in vacuo* and the crude taken up in H_2O, acidified to pH 4 with acetic acid and washed thrice with Et_2O. The organics were discarded and the aqueous layer was concentrated *in vacuo*. The material thus obtained was purified by size-exclusion chromatography (HW-40, 0.15 M NH_4OAc), product containing fractions were pooled, evaporated, azeotroped thrice with MeCN/H_2O (1/1; v/v) and subsequently lyophilized to furnish title *sn*-3 me-CDP-αS_2 Gro **28b** as an off-white powder (NH_4^+-form, 12.1 mg, 22.3 µmol, 40%). **^{1}H NMR** (400 MHz, D_2O) δ 8.18 (d, 1H, *J* = 7.7 Hz, H-6), 6.15 (d, 1H, *J* = 7.7 Hz, H-5), 5.90 (d, 1H, *J* = 3.8 Hz, H-1'), 4.36 (t, 1H, *J* = 5.2 Hz, H-3'), 4.32 (dd, 1H, *J* = 5.1, 3.9 Hz, H-2'), 4.30 – 4.25* (m, 2H, H-4', H-5'), 4.25 – 4.17 (m, 1H, H-5'), 3.95 (app. q, 1H, *J* = 6.4 Hz, H-3_A'') 3.91 – 3.83 (m, 2H, H-2'', H-3_B'') 3.65 (dd, 1H, J_{ABX} = 11.8, 4.2 Hz, H-1_A''), 3.57 (dd, 1H, J_{BAX} = 11.8, 5.9 Hz, H-1_B''), 2.85 (dd, 2H, $^2J_{HP}$ = 18.1, 16.2 Hz, P-CH_2-P); **^{13}C NMR** (101 MHz, D_2O) δ163.8 (C-4), 154.6 (C-2), 143.0 (C-6), 96.0 (C-5), 89.6 (C-1'), 82.8 (d, $^3J_{CP}$ = 9.8 Hz, C-4'), 74.4 (C-2'), 71.01 (d, $^3J_{CP}$ = 6.9 Hz, C-2''), 69.8 (C-3'), 65.2 (d, $^2J_{CP}$ = 6.0 Hz, C-3''), 62.25 – 62.18 (m, C-5', C-1'', δ_C 62.21 d, $^2J_{CP}$ = 6.9 Hz, C-5', δ_C 62.21, s, C-1''), 43.25 (dd, $^1J_{CP}$ = 125.6, 69.2 Hz, P-C_α-P); **^{31}P NMR** (162 MHz, D_2O) δ 107.08 (d, *J* = 13.5 Hz, PS_2), 15.22 (d, *J* = 13.6 Hz, PO); **HRMS** (ESI) m/z: [M+H]$^+$ calcd for $C_{13}H_{24}N_3O_{10}P_2S_2$ 508.0373, found 508.0384.

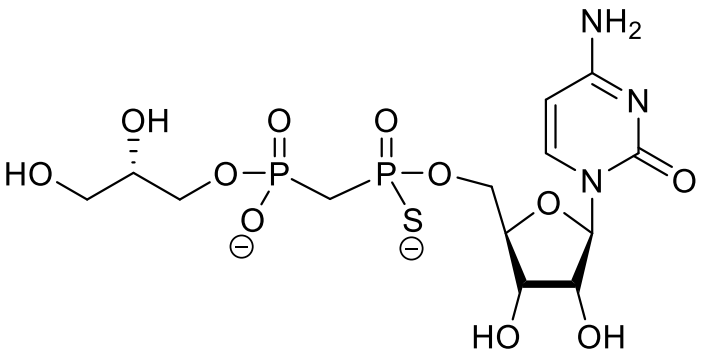

***sn*-1 me-CDP-αS-Gro (29a) -** Fully protected *sn*-1 me-CDP-αS-Gro tetraester **26a** (116 mg, 147.6 µmol, 1.0 eq.) was treated with PhSH/TEA/Dioxane (1/2/2; v/v/v, [1.96], 1.5 mL, 2.95 mmol, 20 eq.) for 4 hours, concentrated and residue partitioned between H_2O and Et_2O. The aqueous layer was acidified to pH 4 with AcOH, washed thrice with Et_2O, isolated and concentrated *in vacuo*. The preceding crude oil was dissolved in AcOH/ 2.3/1; v/v, 1.14 mL, 0.05 M) for 3 hours at 40 °C and the mixture was concentrated and azeotroped with toluene (3 x 1 mL). Finally, overnight ammonolysis with aq. NH_3 (28 wt. %, 1.14 mL, 0.05 M) at ambient temperature, followed by purging of excess ammonia under vacuum and aqueous extractive work-up by dissolving in H_2O, acidifying to pH 4 and washing once with DCM (10 mL) afforded a crude colourless oil. The material thus obtained was purified by size-exclusion chromatography (HW40-column, 0.15 M aqueous NH_4OAc) followed by ion-exchange chromatography and afforded after lyophilization title *sn*-1 me-CDP-αS-Gro **29a** as a white powder (5:4 mixture of P-diastereomers, NH_4^+-form, 40.3 mg, 76.7 µmol, 52%). *Resonances and J-couplings marked with an asterisk * correspond to the minor P-diastereomer.* **^{1}H NMR** (400 MHz, D_2O) 8.08, 8.06* (2 x d, 1H, *J* = 7.7, 7.8* Hz, H-6), 6.10 (d, 1H, *J* = 7.6 Hz, H-5), 5.90 (d, *J* = 3.8 Hz, H-1'), 4.32 (t, 1H, *J* = 5.1 Hz, H-3'), 4.30 (dd, 1H, *J* = 5.1, 3.7 Hz, H-2'), 4.28 – 4.11* (m, 3H, H-4', H-5'), 3.92 (app. q, 1H, *J* = 6.6 Hz, H-3_A''), 3.89 – 3.81* (m, 2H, H-2'', H-3_B''), 3.63 (dd, 1H, J_{ABX} = 11.8, 4.3 Hz, H-1_A''), 3.55 (dd, 1H, J_{BAX} = 11.8, 5.8 Hz, H-1_B''), 2.52, 2.51* (2 x dd, 2H, $^2J_{HP}$ = 19.1, 19* 17.1, 17* Hz, P-CH_2-P); **^{13}C NMR** (101 MHz, D_2O) δ 164.7 (C-4), 155.8 (C-2), 142.5, 142.4* (C-6), 96.2 (C-5), 89.6 (C-1'), 82.81 (d, $^3J_{CP}$ = 8.8 Hz, C-4'), 74.3 (C-2'), 70.95 (d, $^3J_{CP}$ = 6.9 Hz, C-2''), 69.3 (C-3'), 65.24*, 65.15 (2 x d, *J** = 5.3, $^2J_{CP}$ = 5.5 Hz, C-3''), 63.0*, 62.8, (2 x d, *J** = 5.7, $^2J_{CP}$ = 6.1 Hz, C-5'), 62.13, 62.10 (C-1''), 35.87, 35.81* (2 x dd, $^1J_{CP}$ = 125.9, 125.3*, 93.5, 93.2* Hz, P-C_α-P); **^{31}P NMR** (162 MHz, D_2O) δ 68.96 (d, *J* = 12.4 Hz), 68.86* (d, *J* = 12.9 Hz, PS), 15.79* (d, *J* = 12.9 Hz), 15.78 (d, *J* = 12.4 Hz, PO); **HRMS** (ESI) m/z: [M+H]$^+$ calcd for $C_{13}H_{24}N_3O_{11}P_2S$ 492.0601, found 492.0615.

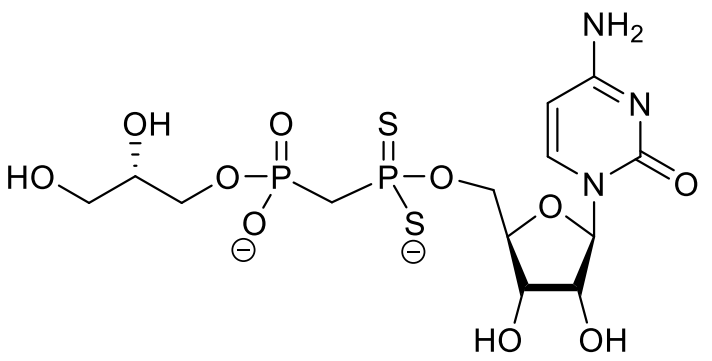

***sn*-1 me-CDP-αS_2 Gro (29b)** – Fully protected *sn*-1 me-CDP-αS_2 Gro tetraester **26b** (70 mg, 87.2 µmol, 1.0 eq.) was treated with PhSH/MeCN/Et_3N (1/2/2; v/v/v, [1.96], 0.87 mL, 20 eq.) for 3 hours and concentrated to dryness. The residue was taken up in AcOH/H_2O (2.3/1; v/v, 1.7 mL, 0.05 M), stirred for 4 hours at 40 °C, concentrated and azeotroped by evaporation of added toluene (3 x) and acetonitrile (3 x) to remove residual acetic acid and water. Next, the crude was dissolved in anhydrous acetonitrile (0.9 mL, 0.1 M), sequentially treated with dried 1,8-Diazabicyclo[5.4.0]undec-7-ene (52.2 µL, 0.349 mmol, 4.0 eq.) for 1 hour, followed by addition of ammonium hydroxice (30 wt. %, 0.87 mL, 1.7 mL, 0.05 M) and the turbid mixture stirred overnight at ambient temperature. The resulting homogenous mixture was rendered free of excess ammonia under reduced pressure, taken up in H_2O, acidified to pH 4 with acetic acid and washed thrice with Et_2O. The aqueous layer was concentrated and purified by gel-filtration

(HW-40, 0.15 M NH_4OAc) to afford after azeotroping with MeCN/H_2O (1/1; v/v, 3 x) and lyophilization title *sn*-1 me-CDP-αS_2 Gro **29b** as an off-white powder (NH_4^+-form, 17.1 mg, 31.6 µmol, 36%). **^{1}H NMR** (400 MHz, D_2O) δ 8.21 (d, 1H, *J* = 7.8 Hz, H-6), 6.16 (d, 1H, *J* = 7.7 Hz, H-5), 5.90 (d, 1H, *J* = 3.7 Hz, H-1'), 4.36 (t, 1H, *J* = 5.2 Hz, H-3'), 4.32 (dd, 1H, *J* = 5.1, 3.7 Hz, H-2'), 4.31 – 4.25 (m, 2H, H-4', H-5'), 4.25 – 4.16 (m, 1H, H-5'), 3.95 (app. q, 1H, *J* = 6.5 Hz, H-3_A'') 3.91 – 3.83 (m, 2H, H-2'', H-3_B'') 3.65 (dd, 1H, J_{ABX} = 11.8, 4.0 Hz, H-1_A''), 3.57 (dd, 1H, J_{BAX} = 11.8, 5.6 Hz, H-1_B''), 2.85 (dd, 2H, $^2J_{HP}$ = 18.4, 15.8 Hz, P-CH_2-P); **^{13}C NMR** (101 MHz, D_2O) δ163.3 (C-4), 153.9 (C-2), 143.2 (C-6), 95.9 (C-5), 89.6 (C-1'), 82.9 (d, $^3J_{CP}$ = 9.8 Hz, C-4'), 74.4 (C-2'), 71.0 (d, $^3J_{CP}$ = 6.9 Hz, C-2''), 69.3 (C-3'), 65.2 (d, $^2J_{CP}$ = 6.0 Hz, C-3''), 62.25 – 62.20 (m, C-5', C-1'', δ_C 62.23 d, $^2J_{CP}$ = 5.6 Hz, C-5', δ_C 62.20 C-1''), 43.3 (dd, $^1J_{CP}$ = 125.2, 69.0 Hz, P-C_α-P); **^{31}P NMR** (162 MHz, D_2O) δ 107.10 (d, 1P, *J* = 13.8 Hz, PS_2), 15.19 (d, 1P, *J* = 13.8 Hz, PO); **HRMS** (ESI) m/z: [M+H]$^+$ calcd for $C_{13}H_{24}N_3O_{10}P_2S_2$ 508.0373, found 508.0384.

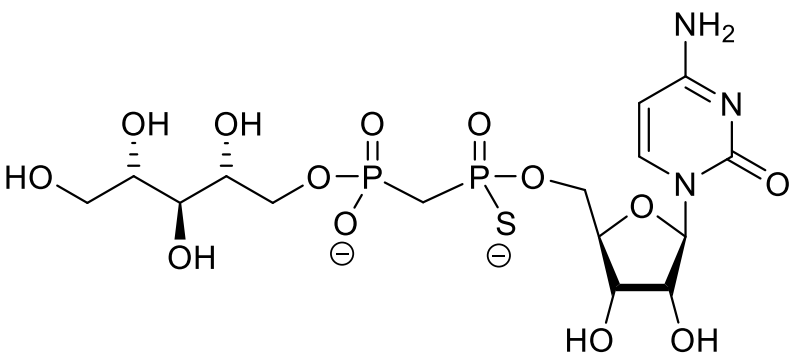

me-CDP-αS-ribitol (30a) – me-CDP-αS-ribitol tetraester **27a** (110 mg, 0.113 mmol, 1.0 eq.) was dissolved in PhSH/TEA/MeCN (1/2/2; v/v/v, 1.1 mL, 20 eq.), stirred at ambient temperature for 2 hours and concentrated *in vacuo*. The crude was taken up in ammonium hydroxide (30 wt. %, 2.3 mL, 0.05 M), stirred overnight, followed by purging of excess ammonia *in vacuo*. After aqueous work-up consisting of diluting with H_2O, acidifying to pH 4, washing thrice with DCM and concentrating the aqueous layer, the residue thus obtained was purified by size-exclusion chromatography (HW-40, 0.15 M NH_4OAC). Product containing fractions were pooled, concentrated and lyophilized to afford title me-CDP-αS-ribitol **30a** as a white powder (5:4 mixture of P-diastereomers, NH_4^+-form, 21.9 mg, 37%). No efforts were made to resolve the PS-diastereomers. *Resonances and J-couplings marked with an asterisk * correspond to the minor P-diastereomer.* **^{1}H NMR** (400 MHz, D_2O) δ 8.18, 8.17* (2 x d, 1H, *J* = 7.9, 7.9* Hz, H-6), 6.17 (d, 1H, *J* = 7.8 Hz, H-5), 5.90 (d, 1H, *J* = 3.4 Hz, H-1), 4.37 – 4.29 (m, 2H, H-2', H-3'), 4.29 – 4.12 (m, 3H, H-4', H-5'), 4.06 (app. ddd, *J* = 10.9, 6.1, 3.0 Hz, H-5_A''), 3.98 (app. dt, 1H, *J* = 10.9, 6.2 Hz, H-5_B''), 3.89 – 3.80 (m, 2H, H-3'', H-4''), 3.79 – 3.69 (m, 2H, CH, H-1_A'', H-2''), 3.60, 3.59* (2 x dd, 1H, J_{BAX} = 11.9, 11.9*, 7.4, 7.3* Hz, H-1_B''), 2.54, 2.53* (2 x app. dd, 2H, $^2J_{HP}$ = 19.1, 19*, 17.2, 17* Hz P-CH_2-P); **^{13}C NMR** (101 MHz, D_2O) δ 162.6 (C-4), 153.1 (C-2), 143.3, 143.2 (C-6), 95.8 (C-5), 89.61, 89.58 (C-1'), 83.0 (d, $^3J_{CP}$ = 8.4 Hz, C-4'), 74.34, 74.31 (C-2'), 72.1, 71.67, 71.65 (C-2'' & C-3''), 71.0 (d, $^3J_{CP}$ = 6.9 Hz, C-4''), 69.2 (C-3'), 65.24, 65.18* (2 x d, $^2J_{CP}$ = 6.4, 6.2* Hz, C-5''), 63.0, 62.7 (2 x d, $^2J_{CP}$ = 5.7, 5.7 Hz, C-5'), 62.31 (C-1''), 35.84, 35.82* (2 x dd, $^1J_{CP}$ = 125.7, 93.7, J^* = 125.4, 93.4 Hz, P-C_α-P); **^{31}P NMR** (162 MHz, D_2O) δ 69.88, 69.82, 69.79, 69.74, 69.71, 69.66, 69.62 69.50 (PS), 16.79, 16.74, 16.72, 16.71, 16.69, 16.66, 16.65, 16.63, 16.62, 16.55 (PO); **HRMS** (ESI) m/z: [M+H]$^+$ calcd for $C_{15}H_{28}N_3O_{13}P_2S$ 552.0813, found 552.0825.

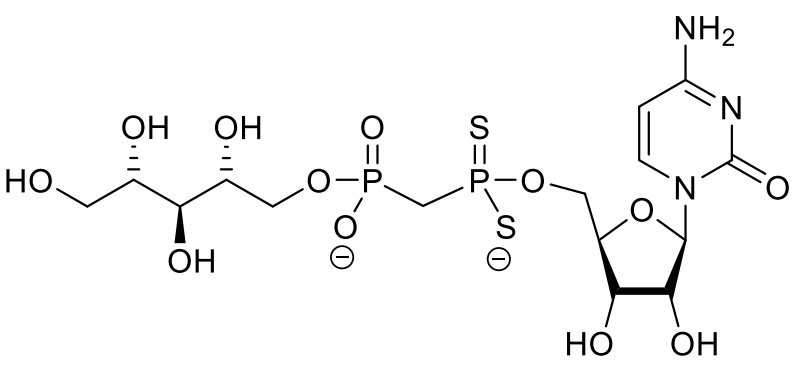

me-CDP-αS_2 ribitol (30b) – Protected me-CDP-αS_2-ribitol tetraester **27b** (44 mg, 44.4 µmol, 1.0 eq.) was dissolved in PhSH/TEA/MeCN (1/2/2; v/v/v, [1.96], 0.44 mL, 88.8 µmol, 20 eq.) and stirred for 2 hours, whereafter ^{31}P NMR analysis inferred complete and selective demethylation. The mixture was concentrated under reduced pressure and dried by coevaporating thrice with acetonitrile to afford a colourless oil. The preceding crude was taken up in anhydrous acetonitrile (0.44 mL, 0.1 M), treated with dry 1,8-diazabicyclo[5.4.0]undec-7-7ene (16.7 µL, 0.111 mmol, 2.5 eq.) for 1 hour followed by the addition of aqueous ammonia (30 wt. %, 1.78 mL, 0.025 M). The amber solution was stirred overnight at ambient temperature and the resulting clear homogeneous solution purged from excess ammonia *in vacuo* to yield an amber oil. The residue was diluted with H_2O, acidified to pH 4 with AcOH and washed thrice with Et_2O. The aqueous layer was isolated, concentrated and the material purified by gel-filtration (HW-40, 0.15 M NH_4OAc) and lyophilized to furnish title me-CDP-αS_2 ribitol **30b** as a white powder (NH_4^+-form, 18.1 mg, 31.9 µmol, 72%). **^{1}H NMR** (400 MHz, D_2O) δ 8.18 (d, 1H, *J* = 7.7 Hz, H-6), 6.14 (d, 1H, *J* = 7.7 Hz, H-5), 5.90 (d, 1H, *J* = 3.7 Hz, H-1'), 4.39 (app. t, 1H, *J* = 5.2 Hz, H-3'), 4.34 (dd, 1H, *J* = 5.0, 3.9 Hz, H-2'), 4.29 – 4.25* (m, 2H, H-4', H-5'), 4.24 – 4.16* (m, 1H, H-5'), 4.09 (app. ddd, 1H, *J* = 9.1, 6.2, 2.8 Hz, H-5_A''), 4.03 – 3.96* (m, 1H, H-5_B'') 3.88 (app. dt, 1H, *J* = 6.1, 3.4 Hz, H-4''), 3.82 (app. td, 1H, *J* = 7.2, 2.9 Hz, H-2''), 3.77 – 3.71 (m, 2H, H-3'', H-1_A''), 3.59 (dd, 1H, J_{ABX} = 11.9, 7.3 Hz, H-1_B''), 2.86 (dd, 2H, $^2J_{HP}$ = 18.1, 16.2 Hz, P-CH_2-P); **^{13}C NMR** (101 MHz, D_2O) δ 163.7 (C-4), 154.5 (C-2), 143.0 (C-6), 96.0 C-5), 89.6 (C-1'), 82.79 (d, $^3J_{CP}$ = 9.7 Hz, C-4'), 74.4 (C-2'), 72.1 (C-2''), 71.7 (C-3''), 71.02 (d, $^3J_{CP}$ = 6.7 Hz, C-4''), 69.3 (C-3'), 65.20 (d, $^2J_{CP}$ = 5.6 Hz, C-5''), 62.3 (C-1''), 62.21 (d, $^2J_{CP}$ = 6.5 Hz, C-5'), 43.18 (dd, $^1J_{CP}$ = 125.8, 69.4 Hz, P-C_α-P); **^{31}P NMR** (162 MHz, D_2O) δ 107.07 (d, *J* = 11.6 Hz, PS_2), 15.50 (d, *J* = 11.7 Hz, PO); **HRMS** (ESI) m/z: [M+H]$^+$ calcd for $C_{15}H_{28}N_3O_{12}P_2S_2$ 568.0584, found 568.0586.

1,2,3,4-tetra-*O*-acetyl-5-*tert*-butylmethyl-(((R_P/S_P)-(2-cyanoethoxy)thiophosphoryl)methyl)phosphonate D-ribitol (31a) –

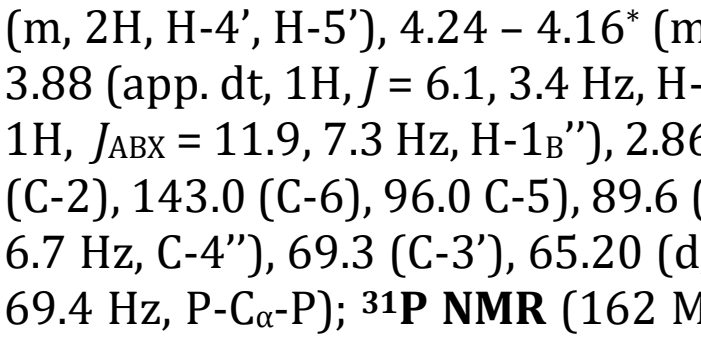

Freshly prepared *tetra-O-*acetyl D-ribitol **24** (100 mg, 0.31 mmol, 1.1 eq.) and phosphonamidite **4** (104 mg, 0.28 mmol, 1.0 eq.) were rendered anhydrous by evaporation of dry acetonitrile (3 x), subsequently dissolved in anhydrous acetonitrile (1.9 mL, 0.15 M) and treated with 4,5-dicyanoimidazole (50 mg, 0.43 mmol, 1.5 eq.). After 30 minutes, sulfurization was effected by treatment with Beaucage reagent (68 mg, 0.34 mmol, 1.2 eq.). The pale yellow solution was stirred for 20 minutes, diluted with EtOAc and successively washed with sat. aq. $NaHCO_3$, sat. aq. NaCl, dried over Na_2SO_4, filtered and concentrated *in vacuo*. Purification by flash column chromatography (0 → 2.5 → 5 → 7.5 → 10 → 15 → 20 → 25 → 30 → 35% acetone in DCM) furnished ribitol bisphosphonate tetraester **31a** as a viscous colourless oil (mixture of 4 P-diastereomers, 155 mg, 0.25 mmol, 88%). No efforts were made to separate the four diastereomers. **^{1}H NMR** (400 MHz, $CDCl_3$) δ 5.38 – 5.33 (m, 1H, H-3), 5.29 – 5.21 (m, 2H, H-2, H-4), 4.46 – 4.06 (m, 6H, H-5, H-5', H-1, H-1', OCH_2 CNE), 3.79, 3.77, 3.76, 3.75 (4 x d, 3H, $^3J_{HP}$ = 11.4, 11.6,

11.4 11.5 Hz, P-OCH_3), 2.87 – 2.65 (m, 4H, P-CH_2-P, CH_2CN), 2.14 – 2.12 (m, 3H, CH_3 Ac), 2.11 – 2.08 (m, 6H, CH_3 Ac), 2.04 (s, 3H, CH_3 Ac), 1.53 – 1.50 (m, 9H, CH_3 ^{t}Bu); **^{13}C NMR** (101 MHz, $CDCl_3$) δ 170.6 169.9, 169.8, 169.5 (C=O Ac), 116.88, 116.86, 116.82, 116.79 (C_q CNE), 84.8 (C_q ^{t}Bu, *detected with HMBC*), 70.1, 70.0, 69.93, 69.89, 69.85, 69.84, 69.79 (C-4), 69.56, 69.55 (C-2), 68.67, 68.65, 68.57, 68.49 (C-3), 64.39 (d, $^2J_{CP}$ = 6.1 Hz, C-5), 64.09, 64.06, 63.98 (app. $^2J_{CP}$ = 4.9, 5.4 Hz), 63.75 (d, $^2J_{CP}$ = 5.6 Hz, C-5), 61.82, 61.77 (2 x d $^2J_{CP}$ = 5.2, 5.4 Hz, OCH_2 CNE), 61.64, 61.58 (C-1), 61.54, 61.48, 61.42, 61.37 (OCH_2 CNE), 53.31, 53.27, 53.25, 53.21, 53.14, 53.08, 53.02 (P-OMe), 36.14 – 33.55 (m, P-C_α-P, *verified with HSQC and HMBC*), 30.33, 30.29 (Me ^{t}Bu), 20.99, 20.96 (Me Ac), 20.92, 20.84, 20.81, 20.79 (Me Ac), 20.69 (Me Ac), 19.66, 19.65, 19.57, 19.50, 19.49 (CH_2CNE); **^{31}P NMR** (162 MHz, $CDCl_3$) δ 87.90, 87.89 (PS), 14.60, 14.59, 14.55, 14.52, 14.49 (PO); **HRMS** (ESI) m/z: $[M+H+NH_3]^+$ calcd for $C_{22}H_{41}N_2O_{13}P_2S$ 635.1799, found 635.1799.

1,2,3,4-tetra-*O*-acetyl-5-*tert*-butylmethyl-(((*R*$_P$/*S*$_P$)-(2-mercaptocyanoethyl)thiophosphoryl)methyl)phoshonate D-ribitol (31b) –

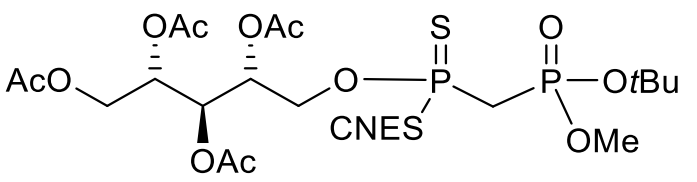

Freshly prepared tetra-*O*-acetyl D-ribitol **24** (32 mg, 0.1 mmol, 1.0 eq.) and thiophosphonamidite **6** (42 mg, 0.11 mmol, 1.1 eq.) were rendered dry by coevaporating thrice with acetonitrile and dissolved in anhydrous acetonitrile (0.7 mL, 0.15 M). The solution was treated with 4,5-dicyanoimidazole (18 mg, 0.15 mmol, 1.5 eq.) for 30 minutes, followed by the addition of Beaucage reagent (24 mg, 0.12 mmol, 1.2 eq.) to effect sulfurization. After stirring for 20 minutes the pale yellow solution was diluted with EtOAc, washed with sat. aq. $NaHCO_3$ and sat. aq. NaCl and the resulting organic layer was dried over Na_2SO_4, filtered and concentrated *in vacuo*. Purification by flash column chromatography (0 → 5 → 10 → 20 → 30 → 40% acetone in DCM) furnished phosphonomethyl phosphonodithioate D-ribitol tetraester **31b** as a pale colourless oil (mixture of 4 P-diastereomers, 52 mg, 82.1 µmol, 82%). No efforts were made to separate the four diastereomers. **^{1}H NMR** (400 MHz, $CDCl_3$) δ 5.37 – 5.30 (m, 1H, H-3), 5.28 – 5.20 (m, 2H, H-2, H-4), 4.38 – 4.16 (m, 2H, H-1, H-5), 4.16 – 3.93 (m, 1H, H-1, H-5'), 3.78, 3.76, 3.74, 3.73 (4 x d, 3H, $^3J_{HP}$ = 11.6, 11.6, 11.6, 11.5 Hz, P-OCH_3), 3.29 – 3.17 (m, 2H, SCH_2 CNE), 3.04 – 2.85 (m, 2H, P-CH_2-P), 2.83 – 2.76 (m, 2H, CH_2CN), 2.11 – 2.09 (m, 3H, CH_3 Ac), 2.09 – 2.06 (m, 6H, CH_3 Ac), 2.02 (br. s, 3H, CH_3 Ac), 1.51 – 1.49 (m, 9H, CH_3 ^{t}Bu); **^{13}C NMR** (101 MHz, $CDCl_3$) δ 170.6, 169.97, 169.95, 169.87, 169.53, 169.50, 169.49 (C=O Ac), 117.95, 117.94, 117.90, 117.88 (C_q CNE), 69.90, 69.87, 69.7 (3 x d, $^3J_{CP}$ = 9.4, 9.5, 9.8 Hz, C-4), 69.62, 69.56 (C-2), 68.99, 68.68, 68.61 (C-3), 62.7, 62.6 (2 x d, $^2J_{CP}$ = 6.3, 6.2 Hz, C-5), 61.75, 61.73 (C-1), 53.36, 53.32, 53.07, 53.05 (4 x d, $^2J_{CP}$ = 6.5, 6.4, 6.4, 6.4 Hz, P-OMe), 40.37 (dd, $^1J_{CP}$ = 142.0, 80.1 Hz, P-C_α-P), 40.15 (dd, $^1J_{CP}$ = 141.7, 80.3 Hz, P-C_α-P), 40.00 (dd, $^1J_{CP}$ = 140.0, 79.7 Hz, P-C_α-P), 39.91 (dd, $^1J_{CP}$ = 140.6, 80.1 Hz, P-C_α-P), 30.44, 30.40 (Me ^{t}Bu), 30.33, 30.32, 30.30, 30.17, 30.12, 30.08 (SCH_2 CNE), 21.03 (Me Ac), 20.98, 20.96, 20.88 (Me Ac), 20.77 (Me Ac), 19.72, 19.70, 19.67, 19.65 (4 x d, $^3J_{CP}$ = 8.4, 8.8, 8.1, 8.1 Hz, CH_2CN); **^{31}P NMR** (162 MHz, $CDCl_3$) δ 98.03, 97.89, 97.73, 97.70, 97.59, 97.56 (PS_2), 13.47, 13.41, 13.36, 13.33, 13.30, 13.27 (PO); **HRMS** (ESI) m/z: $[M+H+NH_3]^+$ calcd for $C_{22}H_{41}N_2O_{12}P_2S_2$ 651.1571 found 651.1577.

1,2,3,4-tetra-*O*-acetyl-5-methyl-(((*R*$_P$/*S*$_P$)-(2-cyanoethoxy)thiophosphoryl)methyl)phosphonate D-ribitol (32a) –

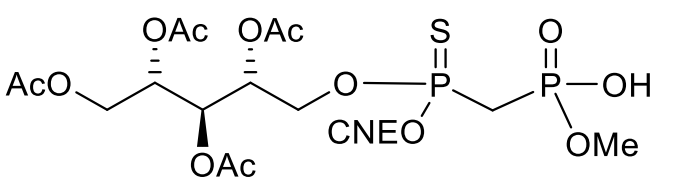

To a solution of ribitol bisphosphonate tetraester **31a** (80 mg, 0.13 mmol, 1.0 eq.) in hexafluoroisopropanol (1 mL, 0.13 M) was added a mixture of concentrated hydrochloric acid with dioxane (1/1; v/v; 6 M, 32.3 µL, 0.195 mmol, 1.5 eq.). ^{31}P NMR affirmed complete acidolytic deblocking after 1 hour and the volatiles were removed *in vacuo*. The crude was azeotroped by evaporation of added portions of toluene (3 x), chloroform (3 x) and acetonitrile (3 x) to remove residual hydrchloric acid and hexafluoroisopropanol and furnished title bisphosphonic acid **32a** as a crystalline white solid that was used without further purification (72 mg, 0.13 mmol, quant.).

1,2,3,4-tetra-*O*-acetyl-5-methyl-(((*R*$_P$/*S*$_P$)-(2-mercaptocyanoethyl)thiophosphoryl)methyl)phosphonate D-ribitol (32b) -

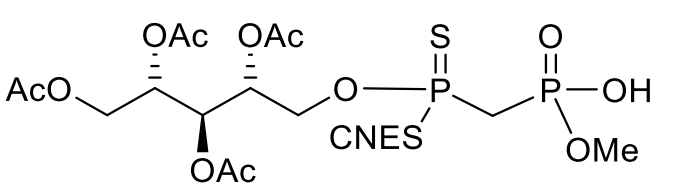

Ribitol phosphonomethyl phosphonodithioate tetraester **31b** (52 mg, 82.1 µmol, 1.0 eq.) was dissolved in hexafluoroisopropanol (0.8 mL, 0.1 M), whereto a mixture of concentrated hydrochloric acid with dioxane (1/1; v/v; 6 M, 16.4 µL, 98.5 µmol, 1.2 eq.) was added. The pale yellow solution was stirred for 1 hour and concentrated *in vacuo*. The crude was coevaporated with toluene (3 x), chloroform (3 x) and acetonitrile (3 x) to furnish title phosphonic acid **32b** as a crystalline white foam (47 mg, 82.1 µmol, quant.). The material was carried on without further purifcation. *Resonances marked with an asterisk * correspond to the minor P-diastereomer.* **^{31}P NMR** (121 MHz, Acetone-capil) δ 96.89, 96.39* (PS_2), 17.36 (PO).

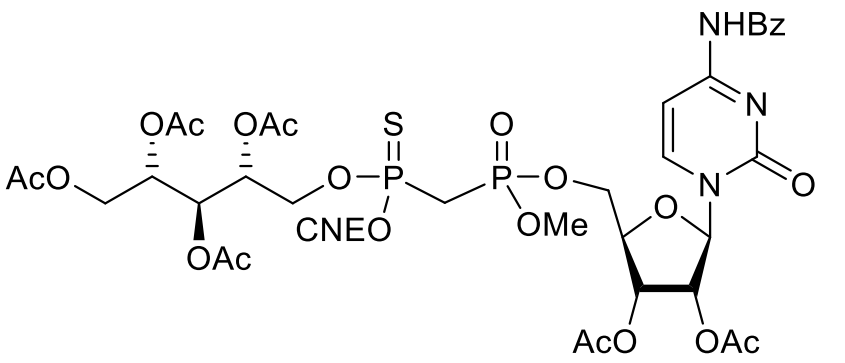

me-CDP-βS-ribitol tetraester (33a) - Ribitol bisphosphonic triester **32a** (72 mg, 0.13 mmol, 1.0 eq.) and cytidine nucleoside **14** (67 mg, 0.16 mmol, 1.2 eq.) were rendered dry by azeotroping thrice with acetonitrile and dissolved in anhydrous acetonitrile (0.65 mL, 0.2 M). Hereto, dry triethylamine (18 µL, 0.13 mmol, 1.0 eq.) and 2,6-lutidine (151 µL, 1.3 mmol, 10.0 eq.) were added, followed by Wada's reagent (194 mg, 0.39 mmol, 3.0 eq.) to effect the phosphonodiester condensation. ^{31}P NMR indicated full conversion within 2 hours, and the reaction mixture was quenched by the addition of 1 M methanolic sodium acetate (1 mL), stirred for an additional 10 minutes, and taken up in 1 M aq. HCl. The aqueous phase was extracted thrice with DCM and the resulting organic layers were combined, dried over Na_2SO_4, filtered and the volatiles removed *in vacuo*. Purification by silica gel column chromatography (0 → 2.5 → 5 → 7.5 → 10 → 15 → 20 → 25 → 30% acetone in DCM) furnished title me-CDP-βS-ribitol tetraester **33a** as a brittle white crystalline solid (mixture of 4 P-diastereomers, 116

mg, 0.12 mmol, 92%). No efforts were undertaken to separate the P-diastereomers since one phosphonate loses chirality upon demethylation. **^{1}H NMR** (400 MHz, CDCl3) δ 8.94 (br. s, 1H, NH), 8.21 – 8.00 (m, 1H, H-6), 7.88 (br. d, *J* = 7.7 Hz, 2H, *o*-Bz), 7.64 – 7.53 (m, 2H, H-5, *p*-Bz), 7.49 (br. t, 2H, *J* = 7.6 Hz, *m*-Bz), 6.23 – 6.15 (m, 1H, H-1'), 5.46 – 5.29 (m, 5H, H-2', H-3', H-3''), 5.28 – 5.19 (m, 2H, H-2'', H-4''), 4.52 – 4.05 (m, 9H, H-4', H-5', H-1'', H-5'', OCH_2 CNE), 3.89 – 3.75 (m, 3H, P-OCH_3), 3.00 – 2.71 (m, 4H, P-CH_2-P, CH_2CNE) 2.13 – 1.98 (m, 18H, CH_3 Ac); **^{13}C NMR** (126 MHz, $CDCl_3$) δ 170.62, 170.59, 170.08, 170.05, 169.94, 169.85, 169.74, 169.67, 169.62, 169.53 (C=O Ac), 166.7 (C=O Bz, *detected with HMBC*), 162.9 (C-4), 154.7 (C-2, *dected with HMBC*), 144.8, 144.4 (C-6, *detected with HSQC*), 133.2 (*p*-Bz), 129.1 (*m*-Bz), 127.8 (*o*-Bz), 117.02, 116.99, 116.98, (C_q CNE), 103.58, 103.53, 103.45 (C-5, *detected with HSQC*), 88.55 (C-1'), 80.97, 80.91, 80.81, 80.78, 80.74 (C-4'), 73.7, 73.72, 69.95, 69.86, 69.65, 69.61, 68.80, 68.75, 68.71, 68.57 (C-2', C-3', C-2'', C-3'' & C-4''), 65.43, 65.36, 65.18, 65.09, 64.78, 64.67, 64.62, 64.44, 64.38, 64.34, 62.13, 62.09, 61.72, 61.65 (C-5', C-5'', OCH_2 CNE), 54.00, 53.94, 53.88, 53.82, 53.80, 53.73, 53.70, 53.63 (P-OMe), 34.60 – 32.35 (m, P-C_α-P, *signal also detected with HSQC*), 21.05, 21.01, 20.91, 20.85, 20.76, 20.62, 20.60, 20.56 (Me Ac), 19.71, 19.64, 19.57 (app. $^{3}J_{CP}$ = 6.8, 7.5 Hz, CH_2CN); **^{31}P NMR** (162 MHz, $CDCl_3$) δ 86.13, 86.09, 86.05, 86.00, 85.98, 85.95, 85.94, 85.92 (PS), 20.70, 20.65, 20.62, 20.57, 20.54, 20.26, 20.23, 20.18, 20.15, 20.08, 19.97 (PO); **HRMS** (ESI) m/z**:** [M+H]$^+$ calcd for $C_{38}H_{49}N_4O_{20}P_2S$ 975.2131, found 975.2122.

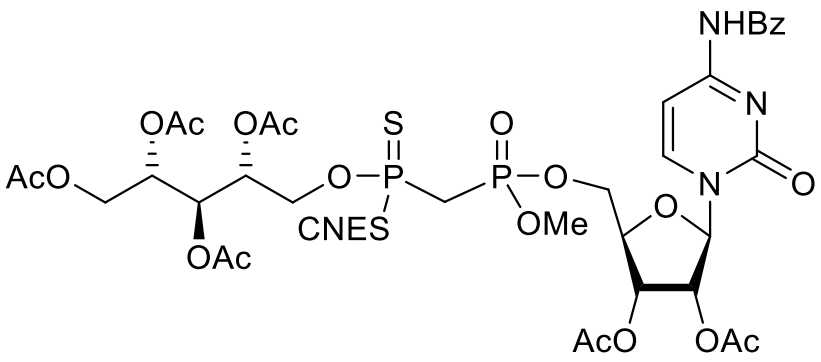

me-CDP-βS_2-ribitol tetraester (**33b**) - D-ribitol phoshonomethyl phosphonodithioate triester **32b** (47 mg, 81.4 µmol, 1.0 eq.) and cytidine nucleoside **14** (42 mg, 97.7 µmol, 1.2 eq.) were dried *in vacuo* with acetonitrile and dissolved in anhydrous acetonitrile (0.4 mL, 0.2 M). Hereto were added triethylamine (11.4 µL, 81.4 µmol, 1.0 eq.), 2,6-lutidine (94.3 µL, 0.81 mmol, 10.0 eq.) and PyNTP (122 mg, 0.24 mmol, 3.0 eq.) in succession. The resulting solution was stirred at ambient temperature for 4 hours, quenched by the addition of 1 M methanolic sodium acetate (1 mL), stirred for an additional 10 minutes and poured into a separatory funnel containing 1 M aq. HCl. The aqueous layer was extracted thrice with DCM and the resulting organic layers were combined, dried over Na_2SO_4, filtered and concentrated *in vacuo*. Purification by flash column chromatography (0 → 5 → 10 → 20 → 30 → 40% acetone in DCM) furnished title me-CDP-βS_2-ribitol tetraester **33b** as an off-white brittle white foam (58 mg, 58.6 µmol, 72%). **^{1}H NMR** (400 MHz, $CDCl_3$) δ 8.80 (br. s, 1H, NH), 8.23 – 8.03 (m, 1H, H-6), 7.91 (app. d, 2H, *J* = 7.4 Hz, *o*-Bz), 7.66 – 7.57 (m, 2H, H-5, *p*-Bz), 7.52 (app. t, 2H, *J* = 7.6 Hz, *m*-Bz), 6.25 – 6.19 (m, 1H, H-1'), 5.50 – 5.36 (m, 3H, H-2', H-3', H-3''), 5.33 – 5.25 (m, 2H, H-2'', H-4''), 4.58 – 4.00 (m, 7H, δ_H 4.14 (app. dd, *J* 12.2, 6.4 Hz), H-4', H-5', H-1'', H-5''), 3.92 – 3.80 (m, 3H, δ_H 3.89, 3.88, 3.87, 3.85 (4 x d, *J* = 11.6 Hz), P-OCH_3), 3.38 – 3.09 (m, 4H, P-CH_2-P, SCH_2 CNE), 2.91 – 2.82 (m, 2H, CH_2CN), 2.18 (s, 6H, CH_3 Ac), 2.15 – 2.08 (m, 12H, CH_3 Ac), 2.04 (s, 3H, CH_3 Ac); **^{13}C NMR** (101 MHz, $CDCl_3$) δ 170.70, 170.09, 169.74, 169.68, 169.60 (C=O Ac), 162.5 (C-4, *detected with HMBC*), 133.4 (*p*-Bz) ,129.2 (*m*-Bz), 127.8 (*o*-Bz), 118.0 (C_q CNE), 88.7 (C-1'), 81.0, 80.94 (C-4'), 73.78, 73.73, 69.78, 69.67, 69.57, 69.05, 68.72, 68.72 (C-2', C-3', C-2'', C-3'', C-4''), 65.38, 65.00, 63.02, 62.84 (C-5', C-5''), 61.8 (C-1''), 54.10, 53.73 (P-OMe), 39.8 – 37.7 (m, P-C_α-P, *detected with HSQC*), 30.60, 30.55, 30.52 (SCH_2 CNE), 21.08, 21.03, 20.92, 20.81, 20.66, 20.61 (Me Ac), 19.80, 19.73, 19.63 (CH_2CN); **^{31}P NMR** (162 MHz, $CDCl_3$) δ 96.53, 96.42, 96.37, 96.32, 96.25 (PS_2), 19.57, 19.53, 19.17, 19.13, 19.11, 19.09 (PO); **HRMS** (ESI) m/z: [M+H]$^+$ calcd for $C_{38}H_{49}N_4O_{19}P_2S_2$ 991.1902, found 991.1901.

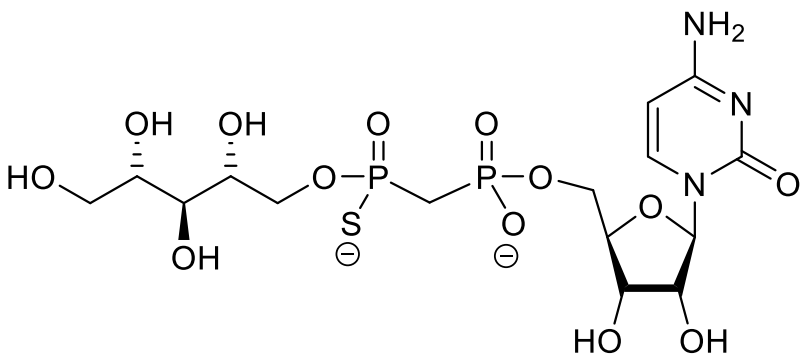

me-CDP-βS ribitol (34a) – Protected me-CDP-βS-ribitol **33a** (105 mg, 0.108 mmol, 1.0 eq.) was dissolved in PhSH/MeCN/TEA (1/2/2; v/v/v; [1.96], 1.1 mL, 20 eq.), stirred for 2 hours until complete demethylation and concentrated to dryness. The residue was suspended in NH_4OH (30 wt. %, 2.2 mL, 0.05 M), stirred overnight at ambient temperature and excess ammonia was evaporated *in vacuo*. The crude was taken up in H_2O, acidified to pH 4 with AcOH, washed thrice with DCM and the aqueous layer concentrated *in vacuo.* The material thus obtained was purified by size-exclusion chromatography (HW-40, 0.15 M NH_4OAc) followed by lyophilization to afford title me-CDP-βS-ribitol **34a** as a white powder (5:4 mixture of P-diastereomers, NH_4^+-form, 28.2 mg, 48.2 µmol, 45%); *Resonances and J-couplings marked with an asterisk * correspond to the minor P-diastereomer.* **^{1}H NMR** (400 MHz, D_2O) δ 8.00*, 8.00 (2 x d, 1H, *J* = 7.5*, 7.6 Hz, H-6), 6.11*, 6.10 (2 x d, 1H, *J* = 7.5*, 7.6 Hz, H-5), 5.93*, 5.93 (2 x d, 1H, *J* = 4.3*, 4.2 Hz, H-1'), 4.34 (app. t, 1H, *J* = 4.9 Hz, H-3'), 4.29 (app. t, *J* = 4.7 Hz, H-2'), 4.24 – 3.98 (m, 5H, H-4', H-5', H-5''), 3.92 – 3.87 (m, 1H, H-4''), 3.86 – 3.81 (m, 1H, H-3''), 3.80 – 3.71 (m, 2H, H-1_A'', H-2''), 3.60*, 3.60 (2 x dd, 1H, J_{BAX} = 11.9*, 12.0, 7.3*, 7.2 Hz, H-1_B''), 2.53, 2.53* (2 x dd, 2H, app. $^{2}J_{HP}$ = 19, 17 Hz, P-CH_2-P); **^{13}C NMR** (101 MHz, D_2O) δ 165.5 (C-4), 156.9 (C-2), 142.0 (C-6), 96.50*, 96.46 (C-5), 89.30, 89.26* (C-1'), 83.03, 82.94* (C-4'), 74.28, 74.24 (C-2'), 72.1 (C-3''), 71.74, 71.71 (C-2''), 70.80 (d, $^{3}J_{CP}$ = 8.0 Hz), 70.65 (d, $^{3}J_{CP}$ = 8.0 Hz, C-4''), 69.39, 69.32 (C-3'), 65.19, 64.99* (2 x d, $^{2}J_{CP}$ = 6.5, 6.5* Hz, C-5'), 63.18, 63.13, 63.08 (C-5''), 62.3 (C-1''), 35.80 (dd, $^{1}J_{CP}$ = 126.1, 96.7 Hz), 35.78 (dd, $^{1}J_{CP}$ = 124.4, 95.6 Hz, P-C_α-P); **^{31}P NMR** (162 MHz, D_2O) δ 68.41* (d, *J* = 14.8 Hz), 68.32 (d, *J* = 13.8 Hz, PS) , 15.81* (d, *J* = 14.3 Hz), 15.79 (d, *J* = 13.4 Hz, PO); **HRMS** (ESI) m/z: [M+H]$^+$ calcd for $C_{15}H_{28}N_3O_{13}P_2S$ 552.0813, found 552.0818.

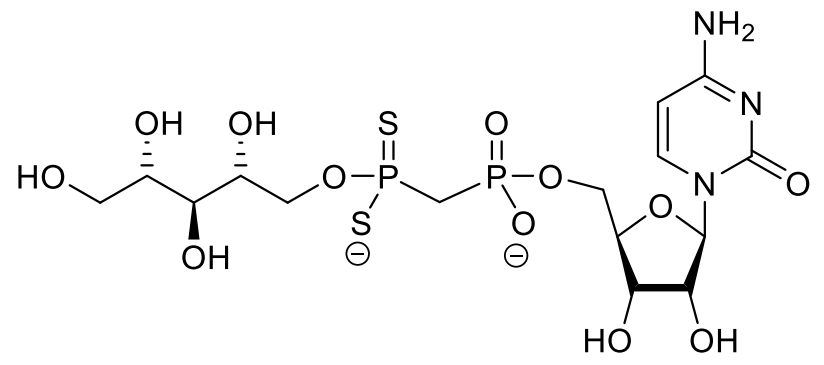

me-CDP-βS$_2$-ribitol (34b) - me-CDP-βS$_2$-ribitol tetraester **33b** 57 mg, 57.5 µmol, 1.0 eq.) was rendered dry by coevaporating with acetonitrile, dissolved in anhydrous acetonitrile (0.58 mL, 0.1 M) and treated with dry 1,8-diazabicyclo[5.4.0]undec-7-7ene (10.5 µL, 70.2 µmol, 1.2 eq.). After 30 minutes ^{31}P NMR inferred quantitative decyanoethylation, the volatiles were removed under reduced pressure and was followed by the addition of PhSH/MeCN/Et$_3$N (1/2/2; v/v/v, [1.96], 0.58 mL, 20 eq.). The resulting orange mixture was stirred for 4 hours, whereupon ^{31}P NMR NMR analysis showed only minor demethylation (~10%). To effectuate demethylation, additional PhSH/MeCN/Et$_3$N (0.58 mL, 20 eq. 40 eq. total) was added. The mixture was stirred overnight at ambient temperature, partially reduced *in vacuo* and resuspended in aqueous ammonia (30 wt. %, 1.6 mL, 0.05 M). After stirring overnight at ambient temperature, excess ammonia was purged *in vacuo*, the residue taken up in H_2O, acidified to pH 4 with AcOH and washed thrice with Et_2O. The aqueous layer was evaporated and the material purified by size-exclusion chromatography (HW-40, 0.15 M NH_4OAc), coevaporated thrice with MeCN/H_2O (1/1; v/v, 3 x 5 mL) and lyophilized to furnish title me-CDP-βS$_2$ ribitol **34b** as an off-white powder (NH_4^+-form, 10.0 mg, 17.6 µmol, 31%). **^{1}H NMR** (400 MHz, D_2O) δ 8.17 (d, 1H, J = 7.8 Hz, H-6), 6.20 (d, 1H, J = 7.8 Hz, H-5), 5.93 (d, 1H, J = 4.6 Hz, H-1'), 4.39 (app. t, 1H, J = 5.0 Hz, H-3'), 4.34 (app. t, 1H, J = 4.8 Hz, H-2'), 4.28 – 4.20 (m, 2H, H-4', H-5$_A$'), 4.20 – 4.11 (m, 2H, H-5$_B$', H-5$_A$''), 4.07 (ddd, 1H, J_{AB} = 10.8, 9.0, 5.8 Hz, H-5$_B$''), 3.93 (app. tdd, 1H, J = 5.8, 3.2, 1.6 Hz, H-4''), 3.85 (app. ddt, 1H, J = 7.2, 5.9, 3.0 Hz, H-3''), 3.79 – 3.72 (m, 2H, H-2'', H-1$_A$''), 3.60 (dd, 1H, J_{BAX} = 11.8, 7.2 Hz, H-1$_B$''), 2.88 (dd, 2H, $^2J_{HP}$ = 18.3, 15.4 Hz, P-CH$_2$-P); **^{13}C NMR** (101 MHz, D_2O) δ 164.8 (C-4), 156.1 (C-2), 142.4 (C-6), 96.45, 96.42 (2 x C-5), 89.2 (C-1'), 83.19 (d, $^3J_{CP}$ = 8.4 Hz, C-4'), 74.3 (C-2'), 72.10, 72.07 (C-2''), 71.86, 71.83 (C-3''), 70.59 (d, $^3J_{CP}$ = 8.8 Hz, C-4''), 69.5 (C-3'), 64.32 (d, $^2J_{CP}$ = 6.9 Hz, C-5''), 63.17 (d, $^2J_{CP}$ = 5.3 Hz, C-5'), 62.3 (C-1''), 43.27 (dd, $^1J_{CP}$ = 123.6, 71.7 Hz, P-C$_\alpha$-P); **^{31}P NMR** (162 MHz, D_2O) δ 105.93 (d, J = 16.7 Hz, PS$_2$), 15.30 (d, J = 16.7 Hz); **HRMS** (ESI) m/z: [M+H]$^+$ calcd for $C_{15}H_{28}N_3O_{12}P_2S_2$ 568.0584, found 568.0588.

Synthesis of phosphonomethyl phosphonbisamidite 10 monitored by ^{31}P NMR

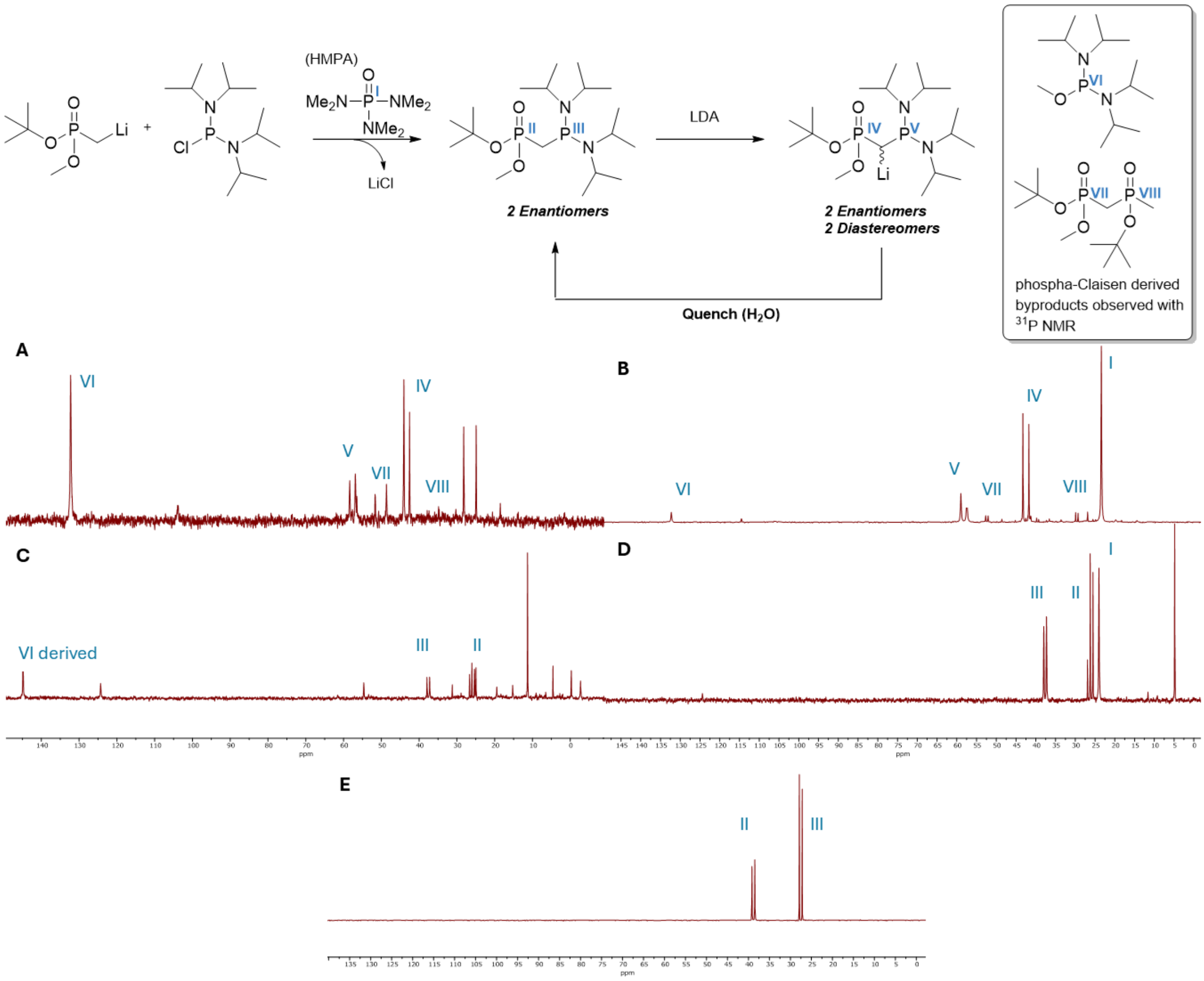

Figure S1. Synthesis of phosphonomethyl phosphonbisamidite **10** with and without HMPA monitored by ^{31}P NMR. **A** and **B:** The reaction after addition of chlorophosphorbisamidite in the absence **(A)** and presence **(B)** of HMPA. The spectra show quenched and lithiated phosphorus species due to excess LDA. The introduced stereocenter as a result of lithiation (peaks IV and V) provides two distinguishable diastereomers. With HMPA as a cosolvent, the formation of the side-products from a phospha-Claisen self-condensation (peaks VI, VII, and VIII) is decreased. **C**

and **D:** The crude mixtures after extractive work-up. Highly polar and water-soluble side-products are largely washed out, and protonation of the lithiated species gives racemic **28** (peaks II and III), simplifying the spectra. **E:** Phosphonbisamdite **10** after column purification using high purity-grade silica gel.

Synthesis of Phosphonomethyl Thiophosphonamidites 5 and 6 by Amidophosphonylation of HSCNE with Phosphonbisamidites 9 and 10 Monitored by ^{31}P NMR

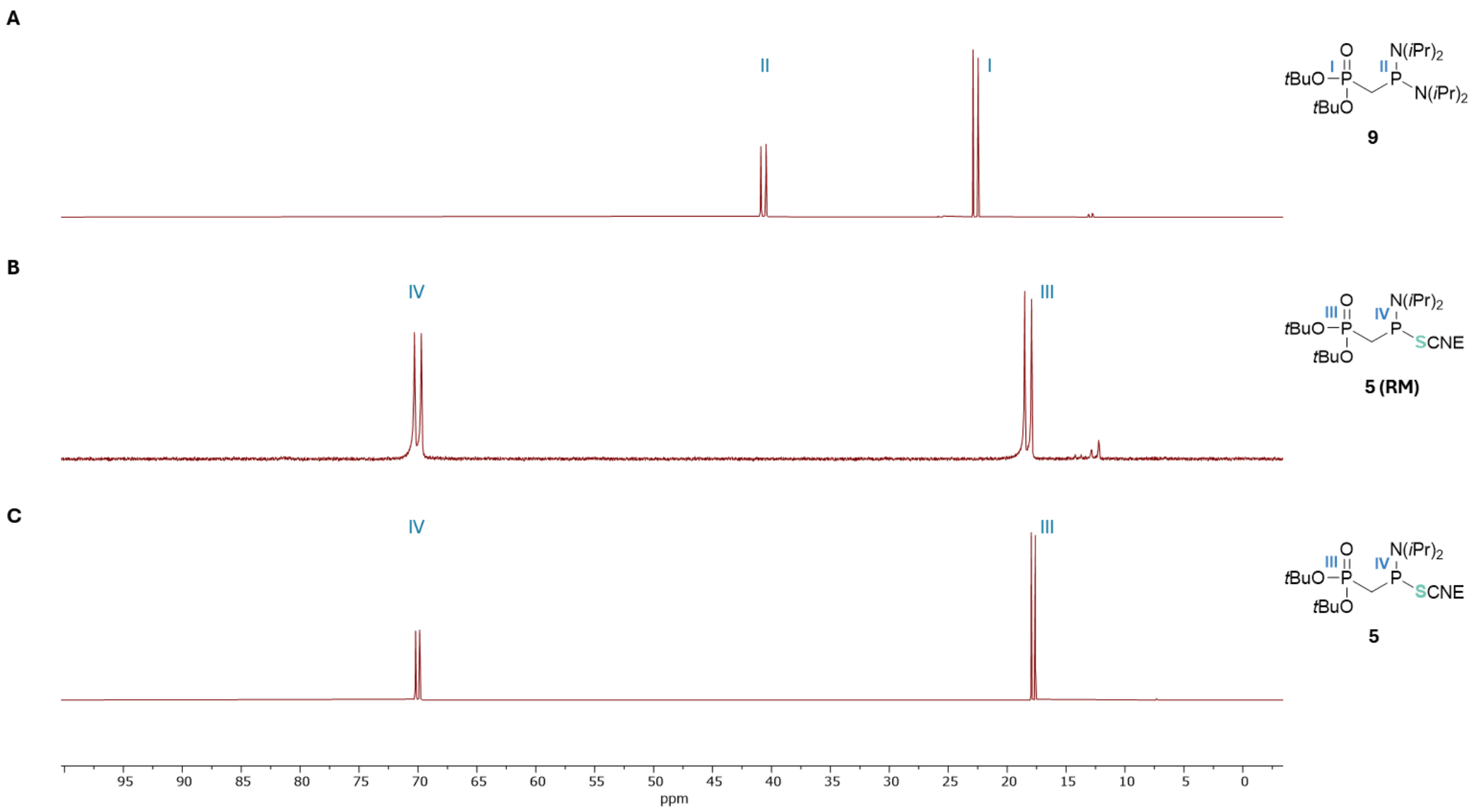

Figure S2. Synthesis of phosphonomethyl thiophosphonamidite **5** monitored by ^{31}P NMR. **A:** Phosphonbisamidite **9**. **B:** Extent of amidophosphonityation of mercaptocyanoethyl with phosphonbisamidite **9** in the presence of catalytic 4,5-dicyanoimidazole as activator after 30 minutes, showing clean and quantitative conversion into thiophosphonamidite **5**. **C:** Phosphonthioamidite **5** after column purification using high-purity silica gel.

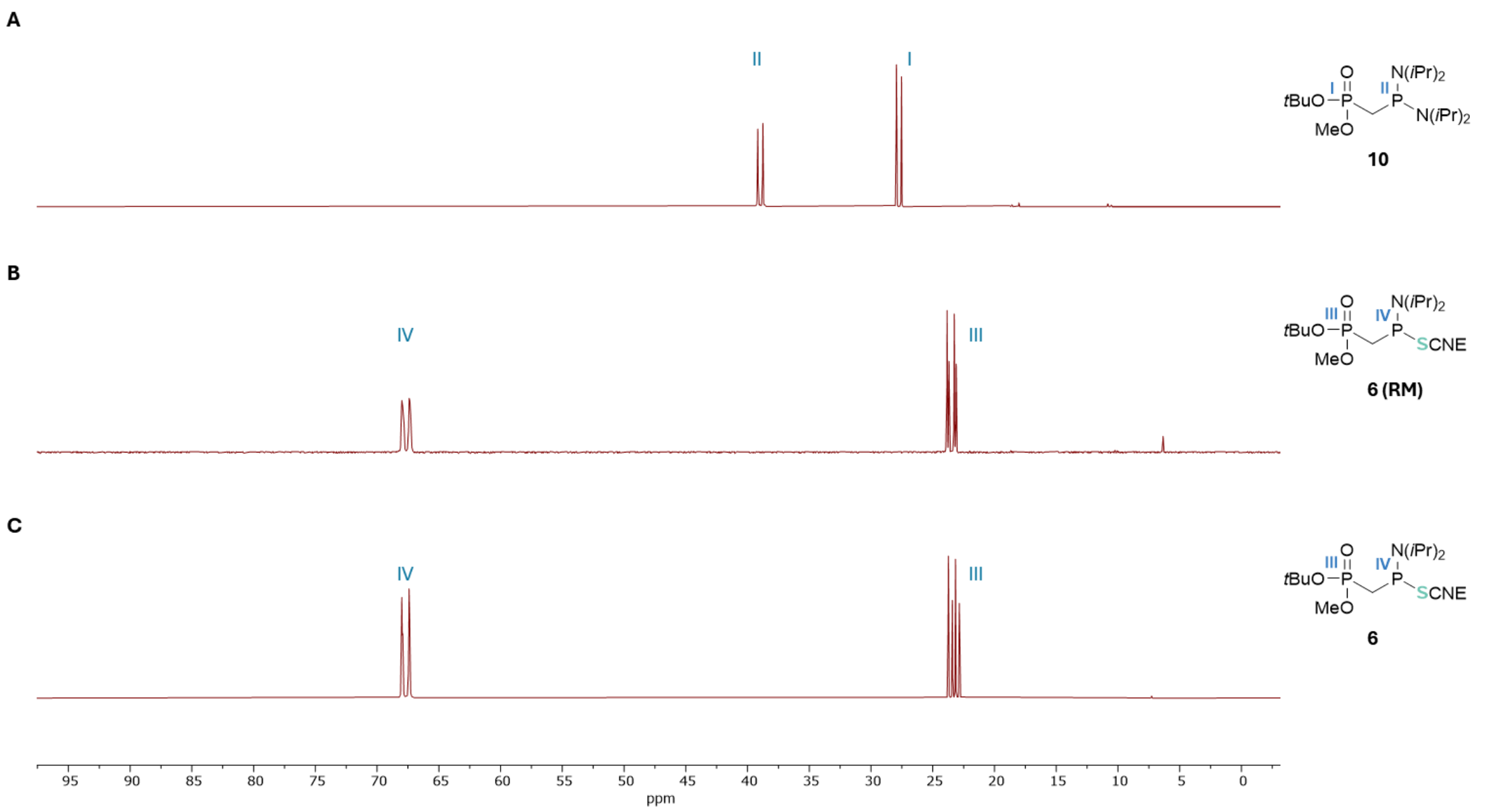

Figure S3. Synthesis of phosphonomethyl thiophosphonamidite **6** monitored by ^{31}P NMR. **A:** Phosphonbisamidite **10**. **B:** Extent of amidophosphonityation of mercaptocyanoethyl with phosphonbisamidite **10** in the presence of catalytic 4,5-dicyanoimidazole as activator after 30 minutes, showing clean and quantitative conversion into

thiophosphonamidite **5**. **C:** Phosphonthioamidite **6** after column purification using high-purity silica gel. The diastereomeric phosphonthioamidite reagent has resolved spectra.

P^{III}-Coupling and Sulfurization of (Thio)Phosphonamidites 4 and 6 with Cytidine Derivative 14 Monitored by ^{31}P NMR

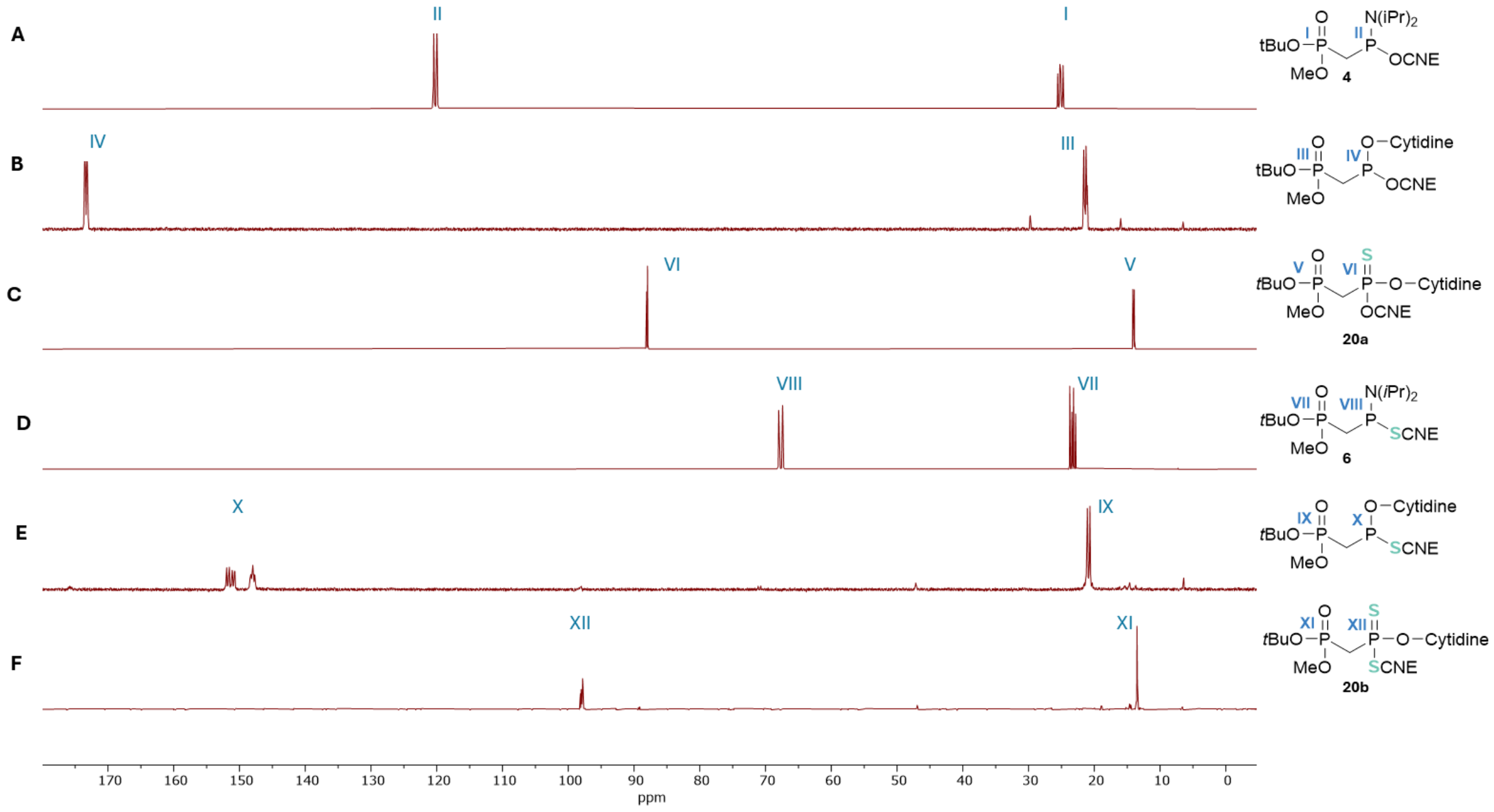

Figure S4. Synthesis of phosphonomethyl phosphono(di)thioate cytidine **20a** and **20b** monitored by ^{31}P NMR spectroscopy. **A** and **D:** (thio)phosphonamidite reagents **4** and **6. B:** Condensation of **4** with cytidine **14**. **C:** Crude phosphonomethyl phosphonothioate cytidine **20a** after sulfurization, showing clean and quantitative conversion. **E:** Condensation of **6** with cytidine **14. F:** Crude phosphonomethyl phosphonodithioate cytidine **20b** after sulfurization. The spectrum shows four partially resolved PS_2 diastereomers and almost quantitative conversion.

NMR Spectra of Phosphonomethyl (Thio)phosphonamidite Reagents

Phosphonomethyl Phosphonbisamidite 9

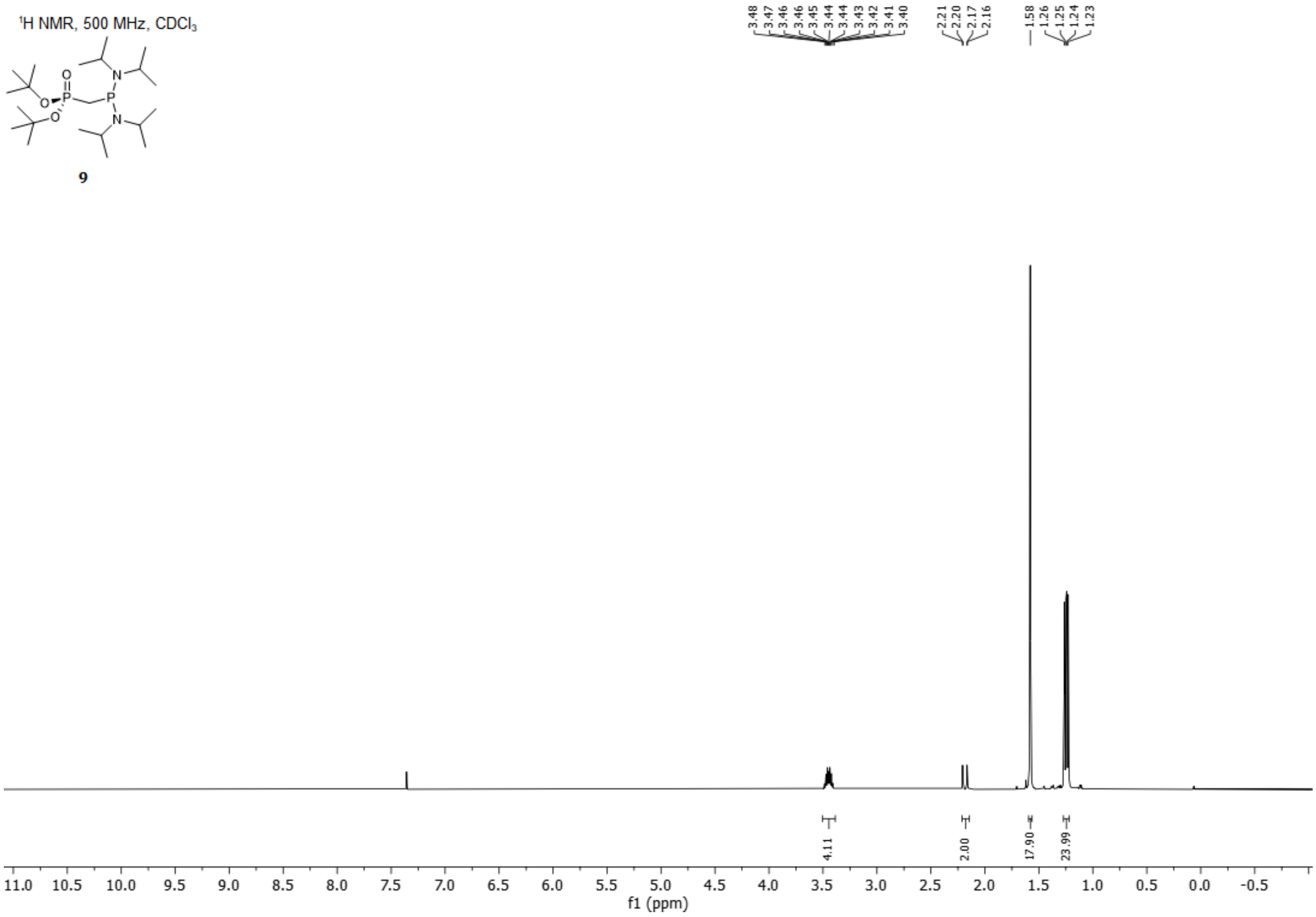

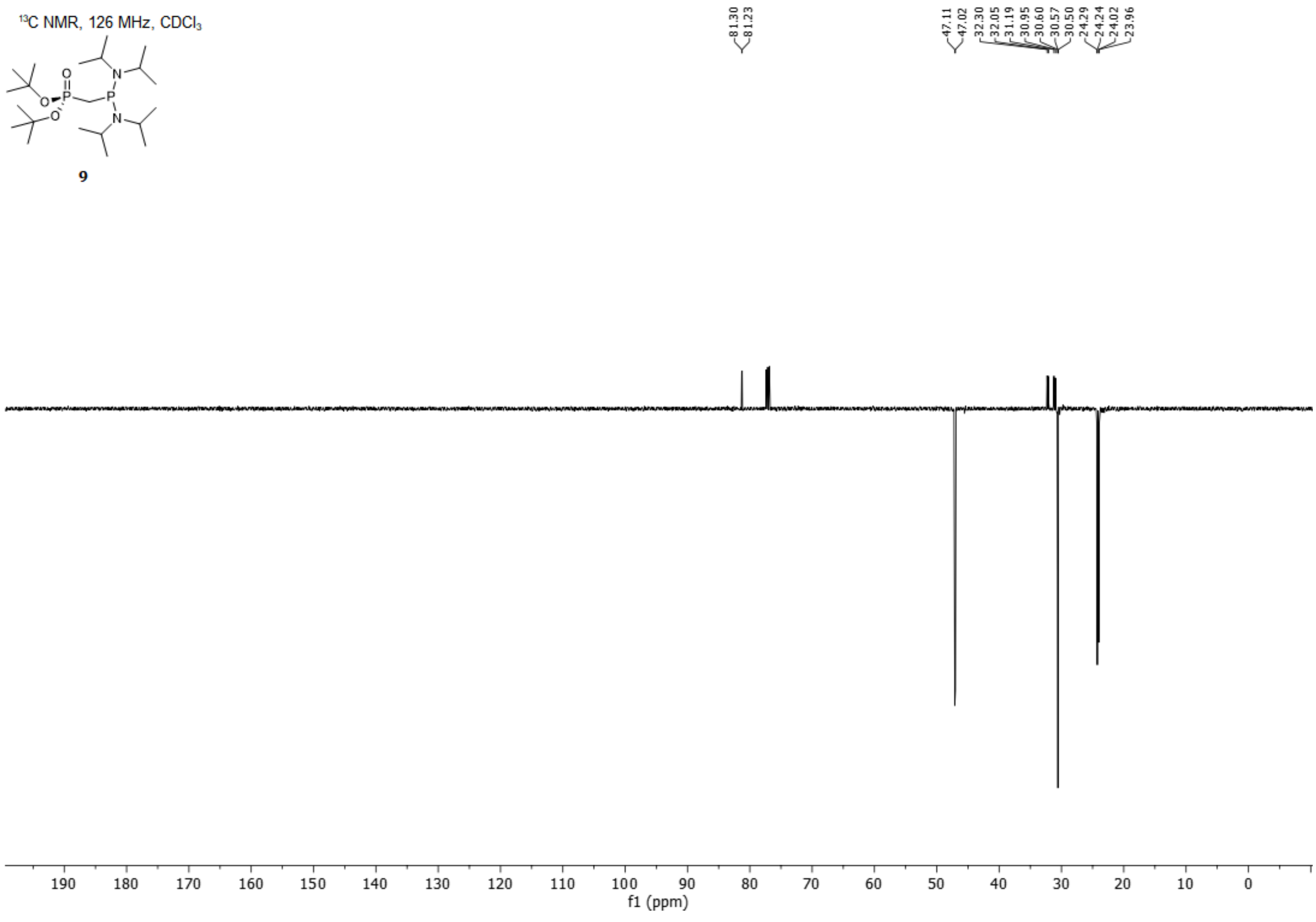

$^{31}P\{^1H\}$ NMR, 202 MHz, $CDCl_3$

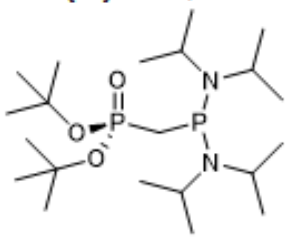

9

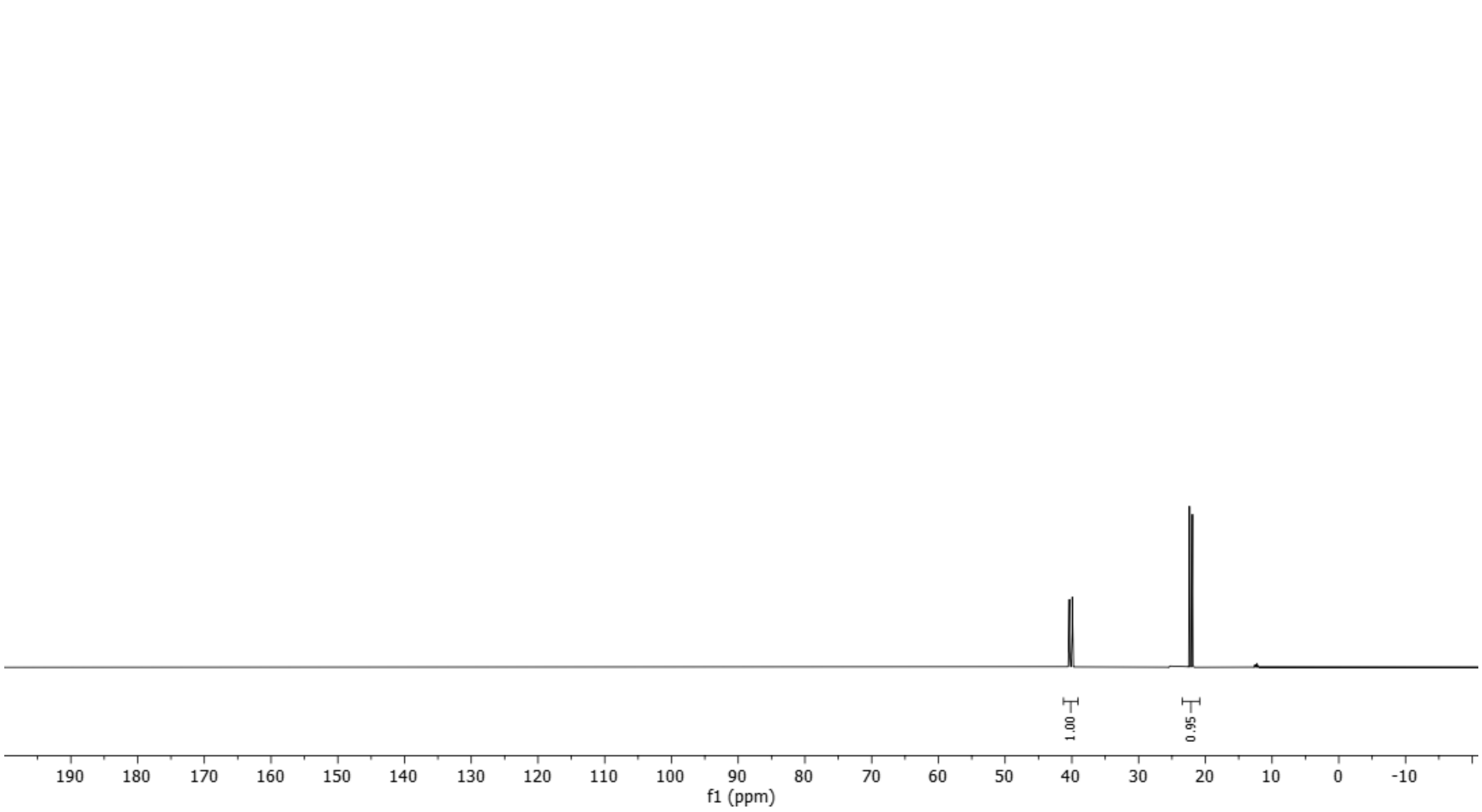

^{31}P NMR, 202 MHz, $CDCl_3$

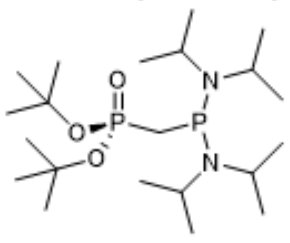

9

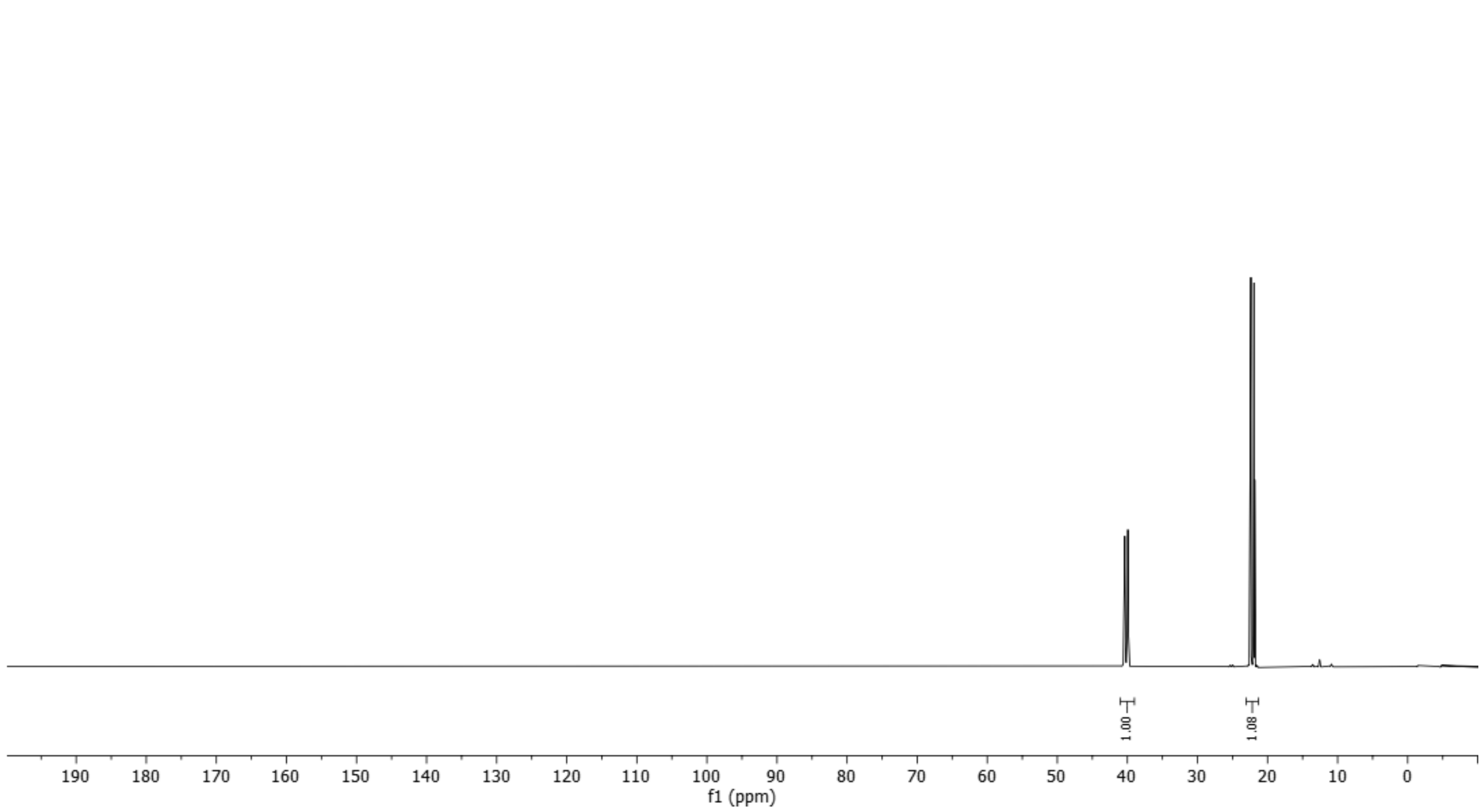

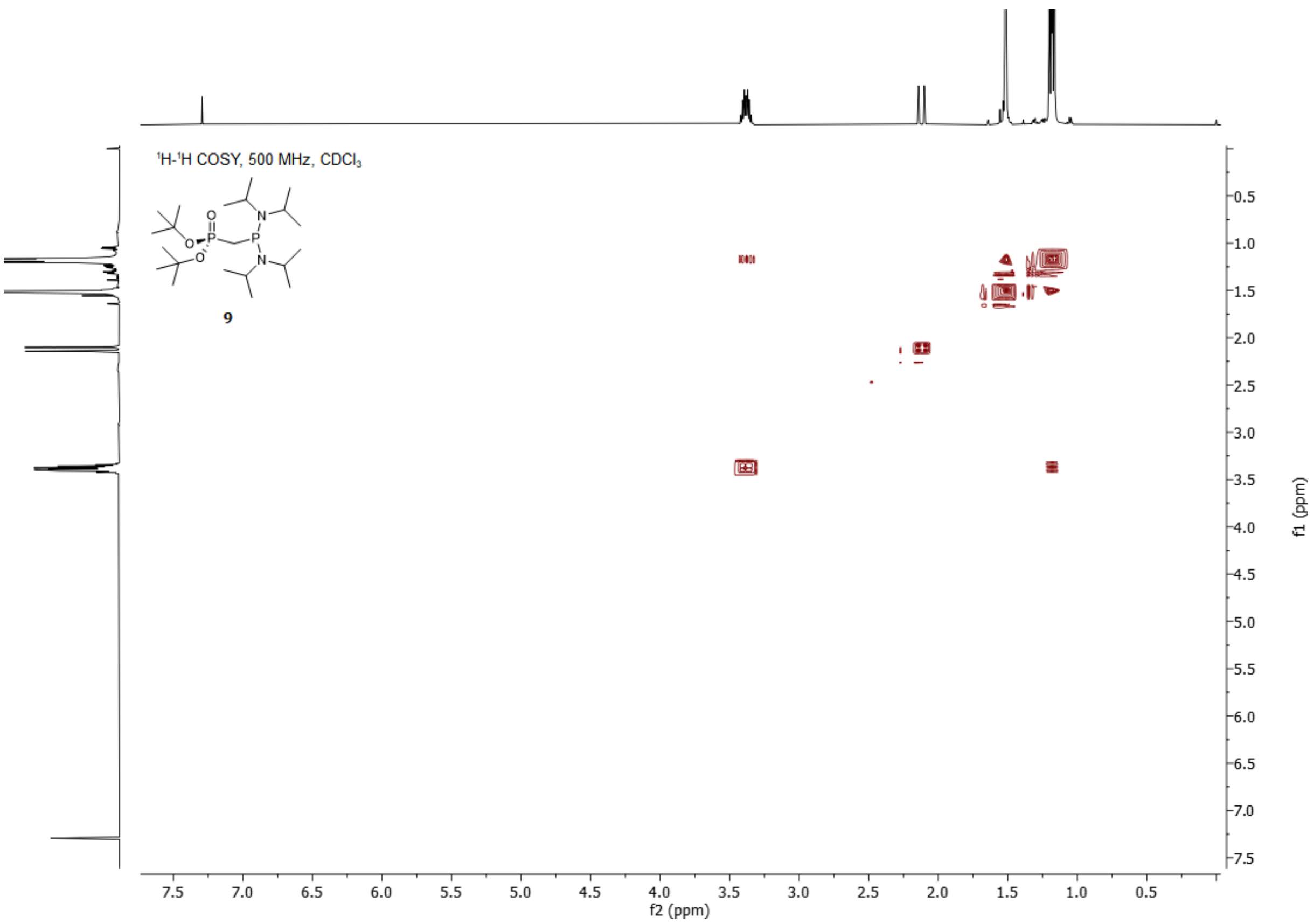

1H-1H COSY, 500 MHz, CDCl3
9
f2 (ppm)
f1 (ppm)

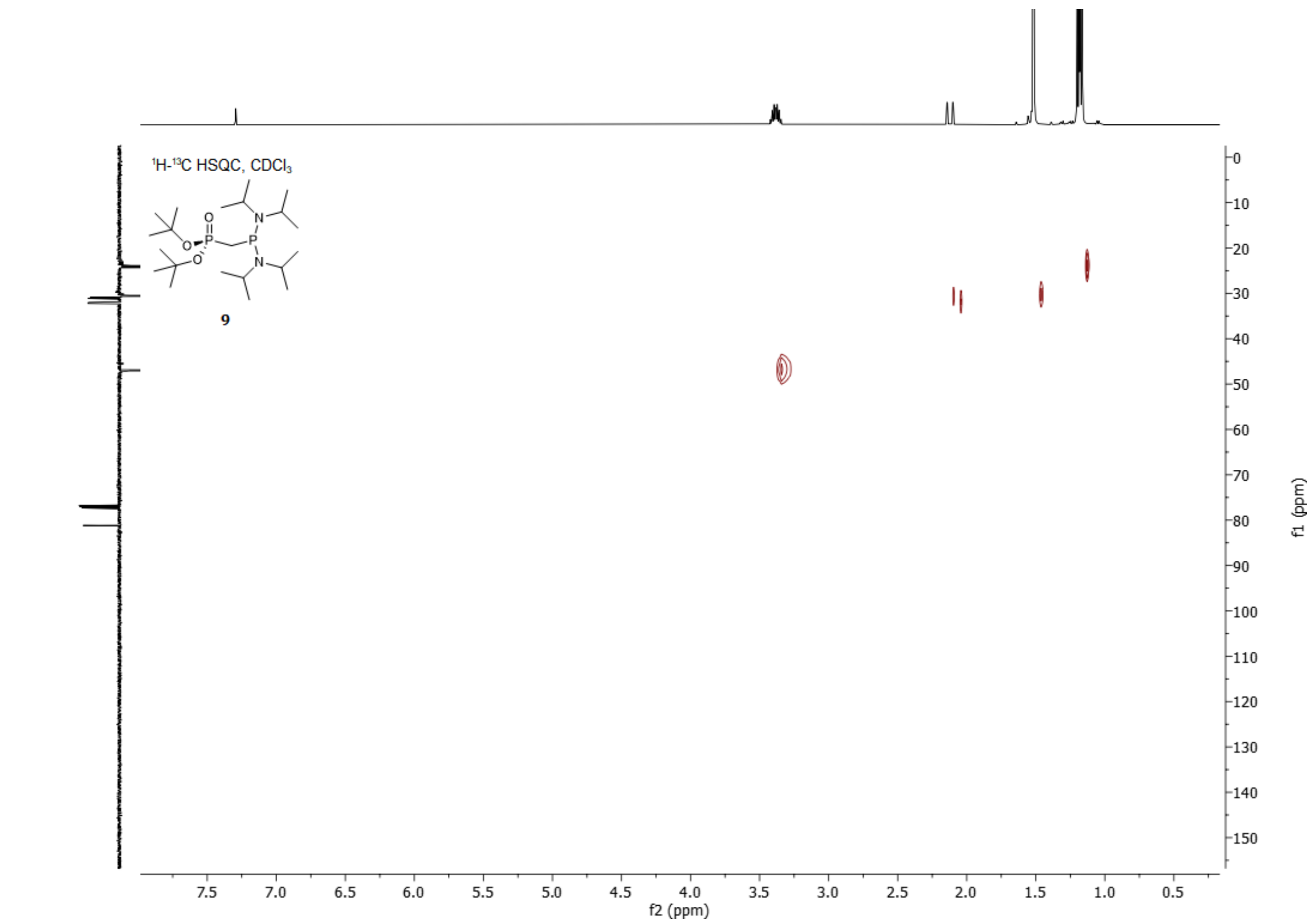

1H-13C HSQC, CDCl3
9
f2 (ppm)
f1 (ppm)

Phosphonomethyl Phosphonbisamidite 10

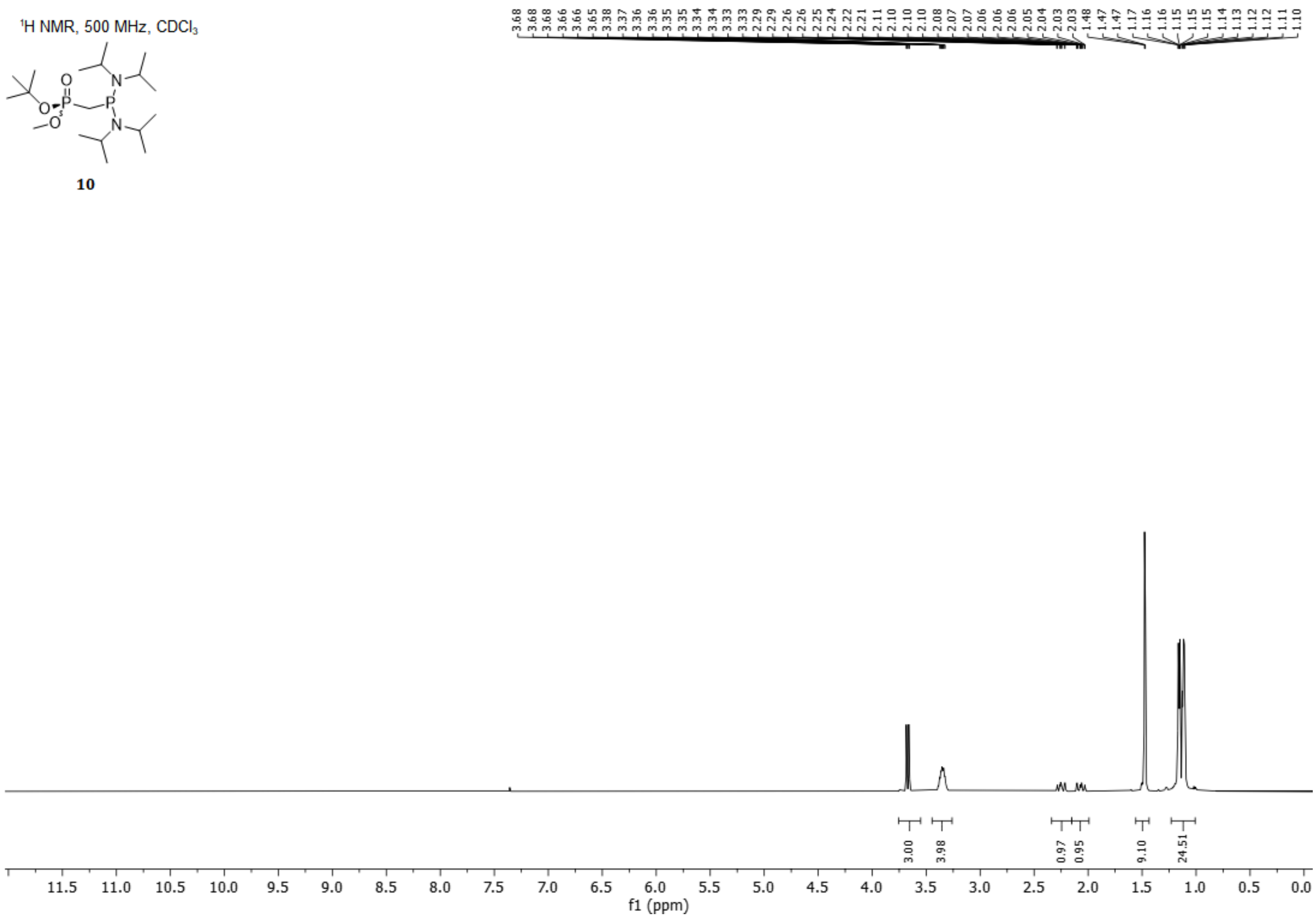

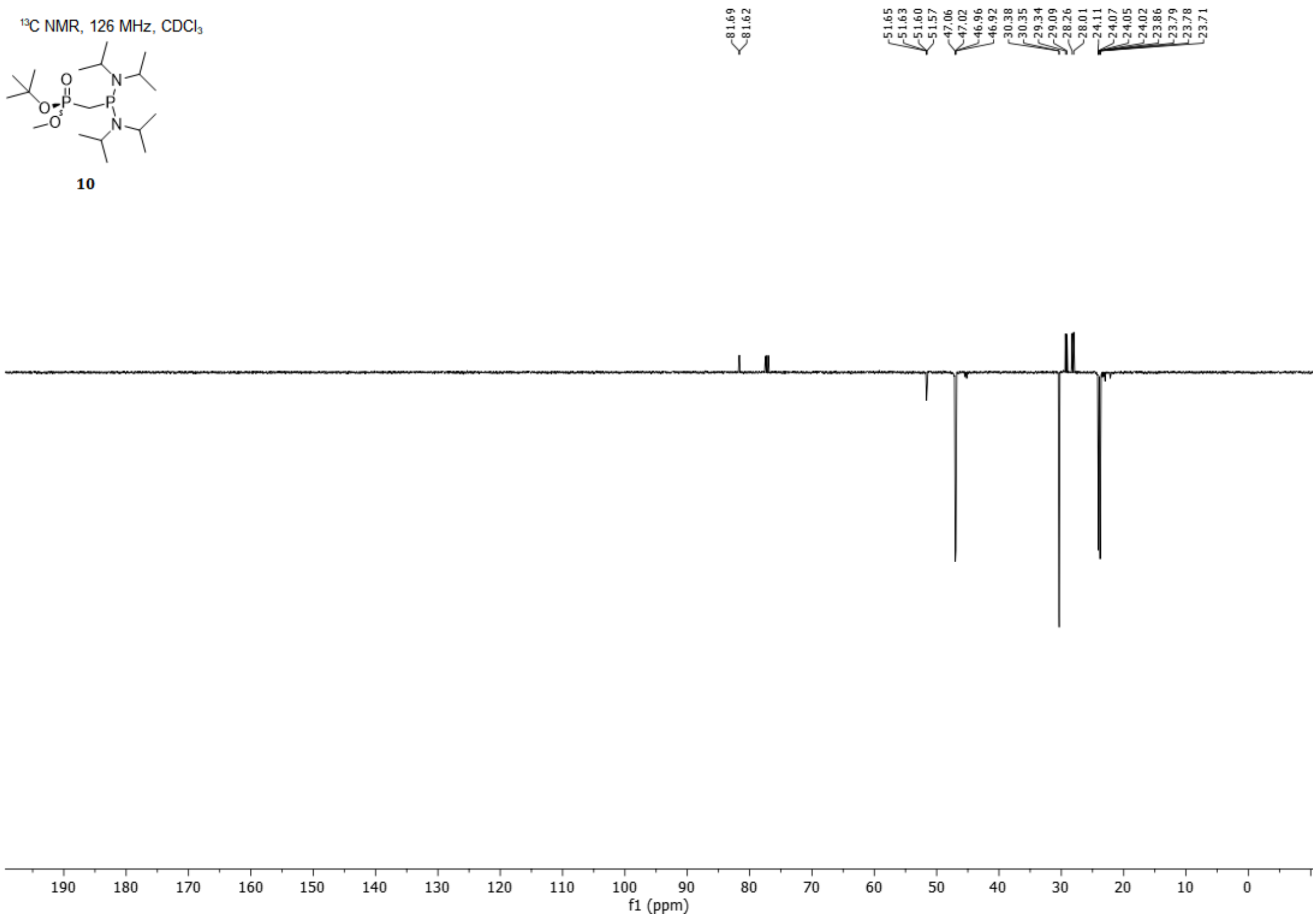

$^{31}P\{^1H\}$ NMR, 202 MHz, $CDCl_3$

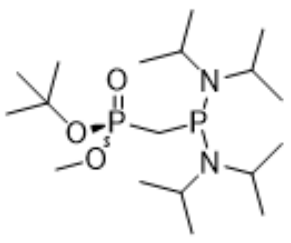

10

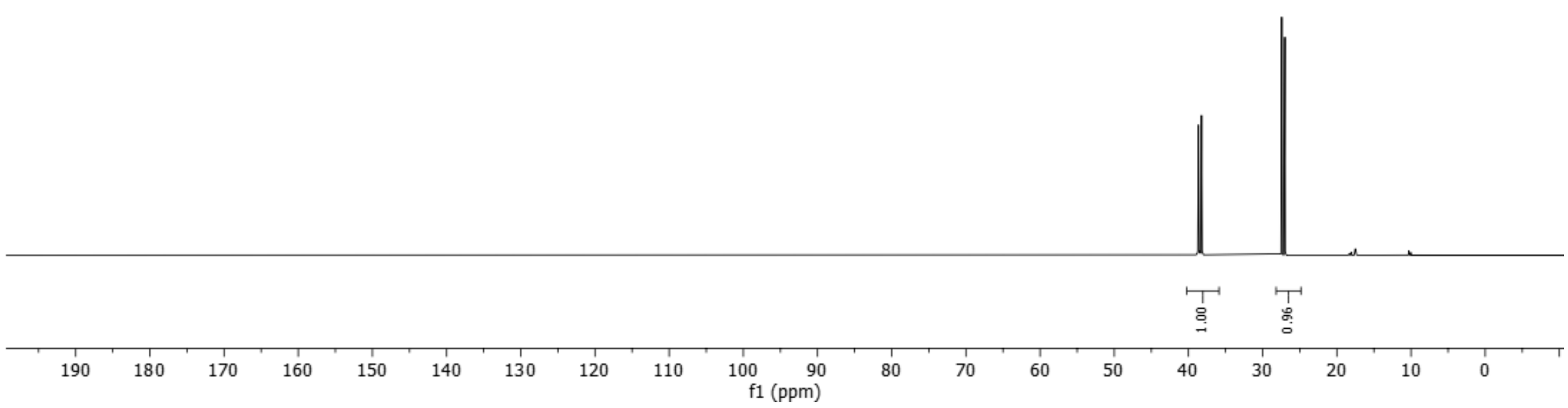

^{31}P NMR, 202 MHz, $CDCl_3$

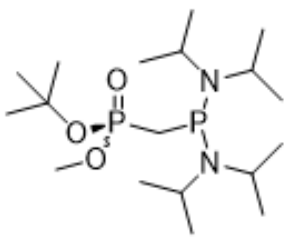

10

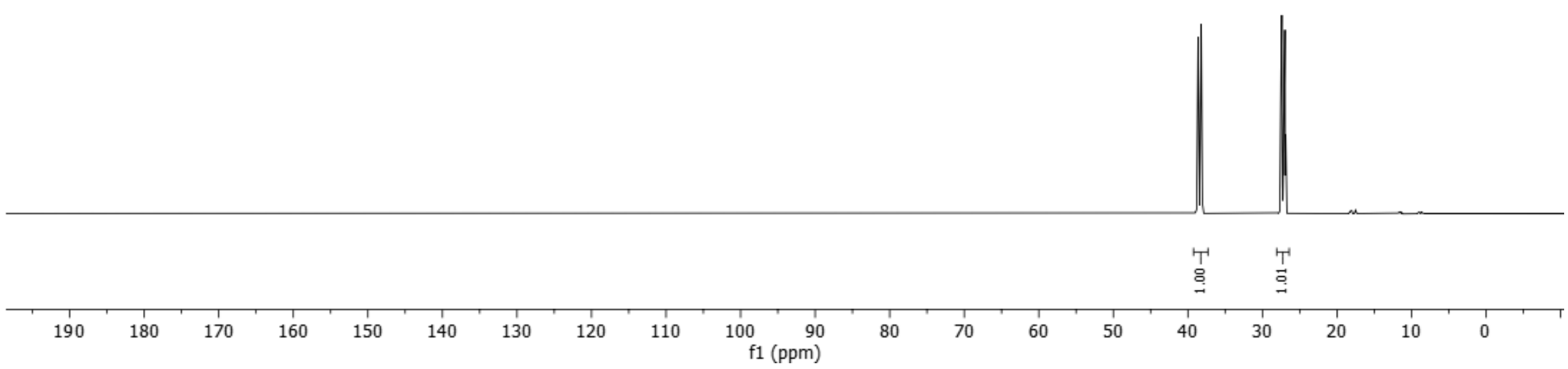

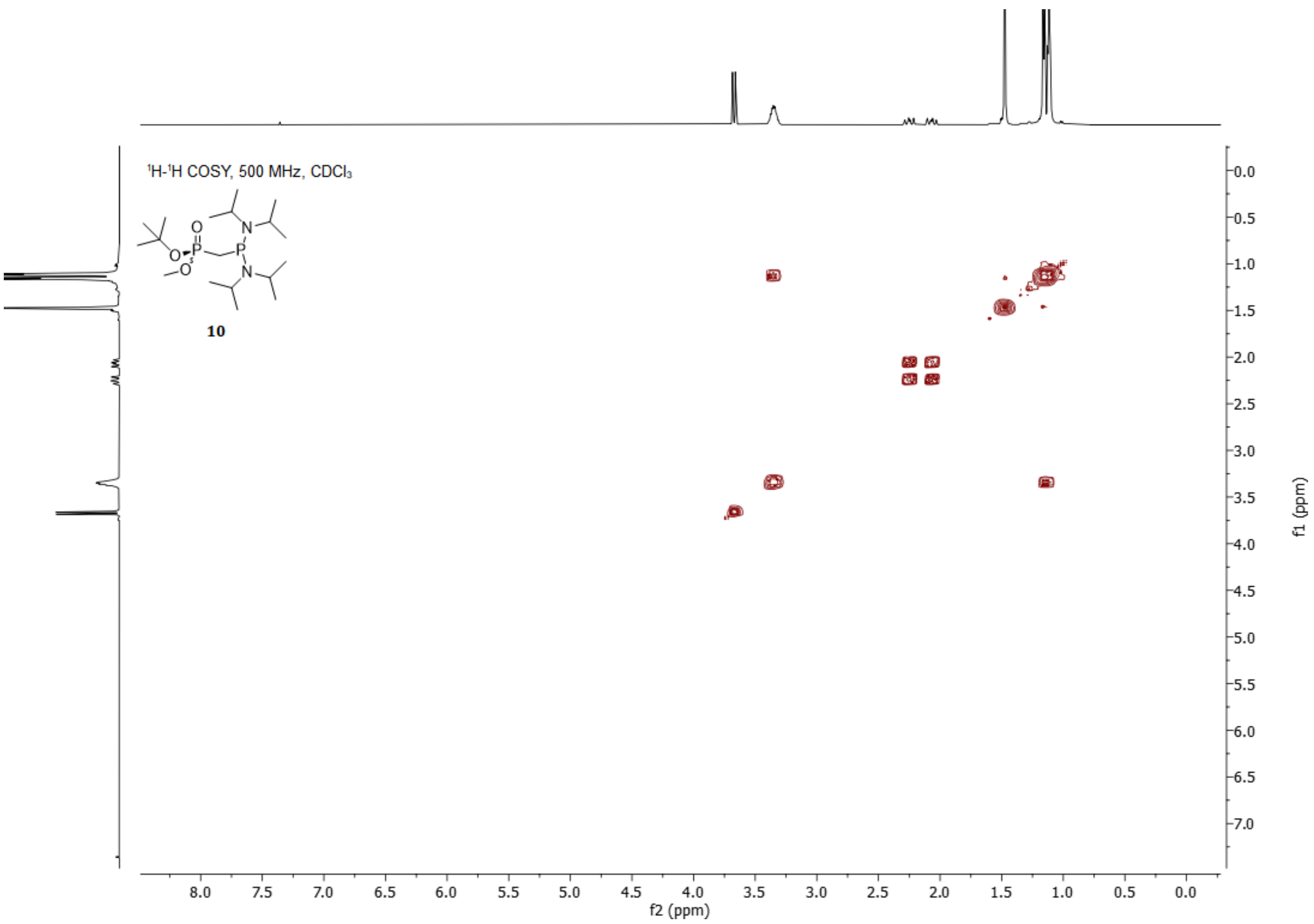
1H-1H COSY, 500 MHz, CDCl3
10
f2 (ppm)
f1 (ppm)

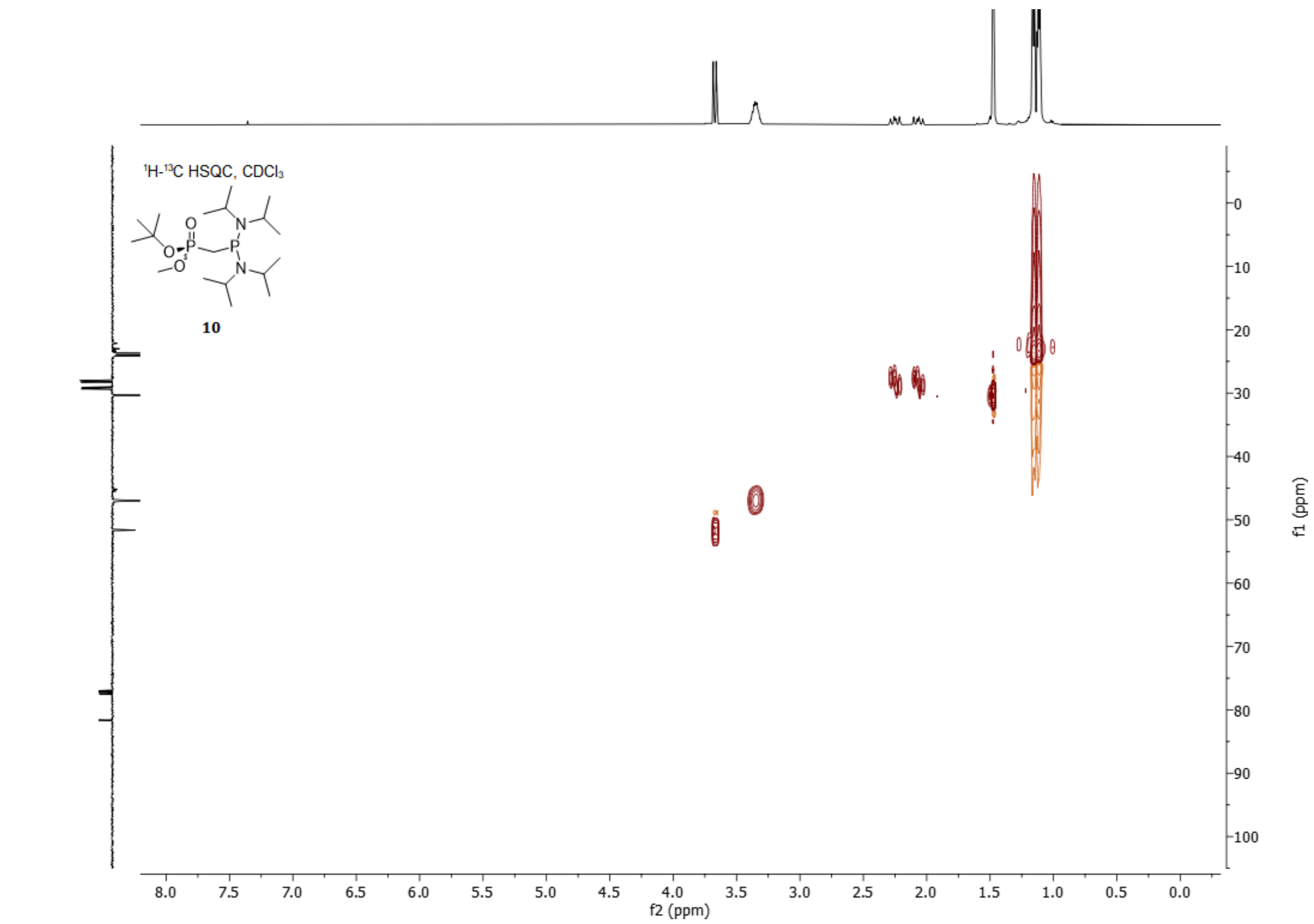
1H-13C HSQC, CDCl3
10
f2 (ppm)
f1 (ppm)

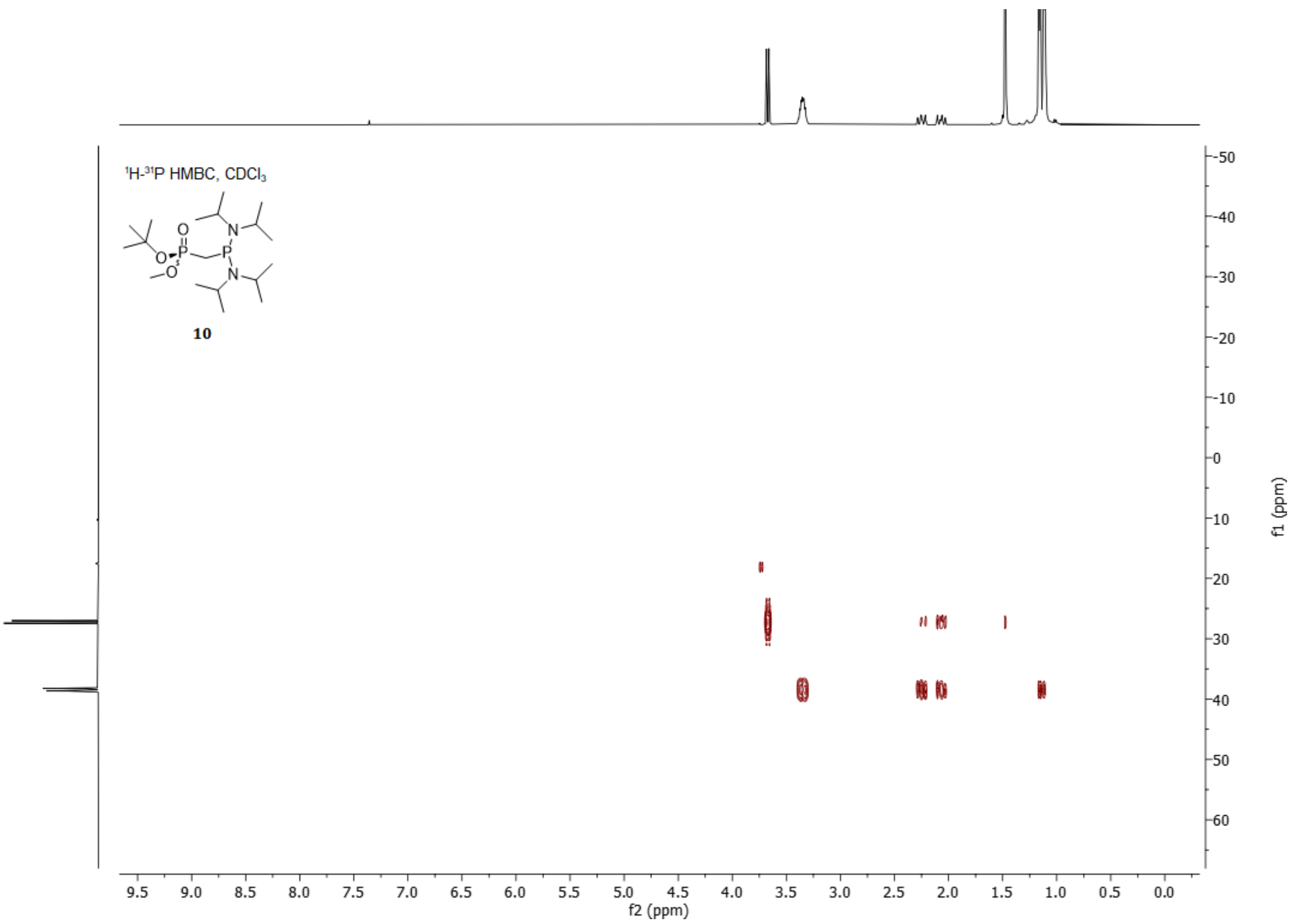

Phosphonomethyl Phosphonamidite 2

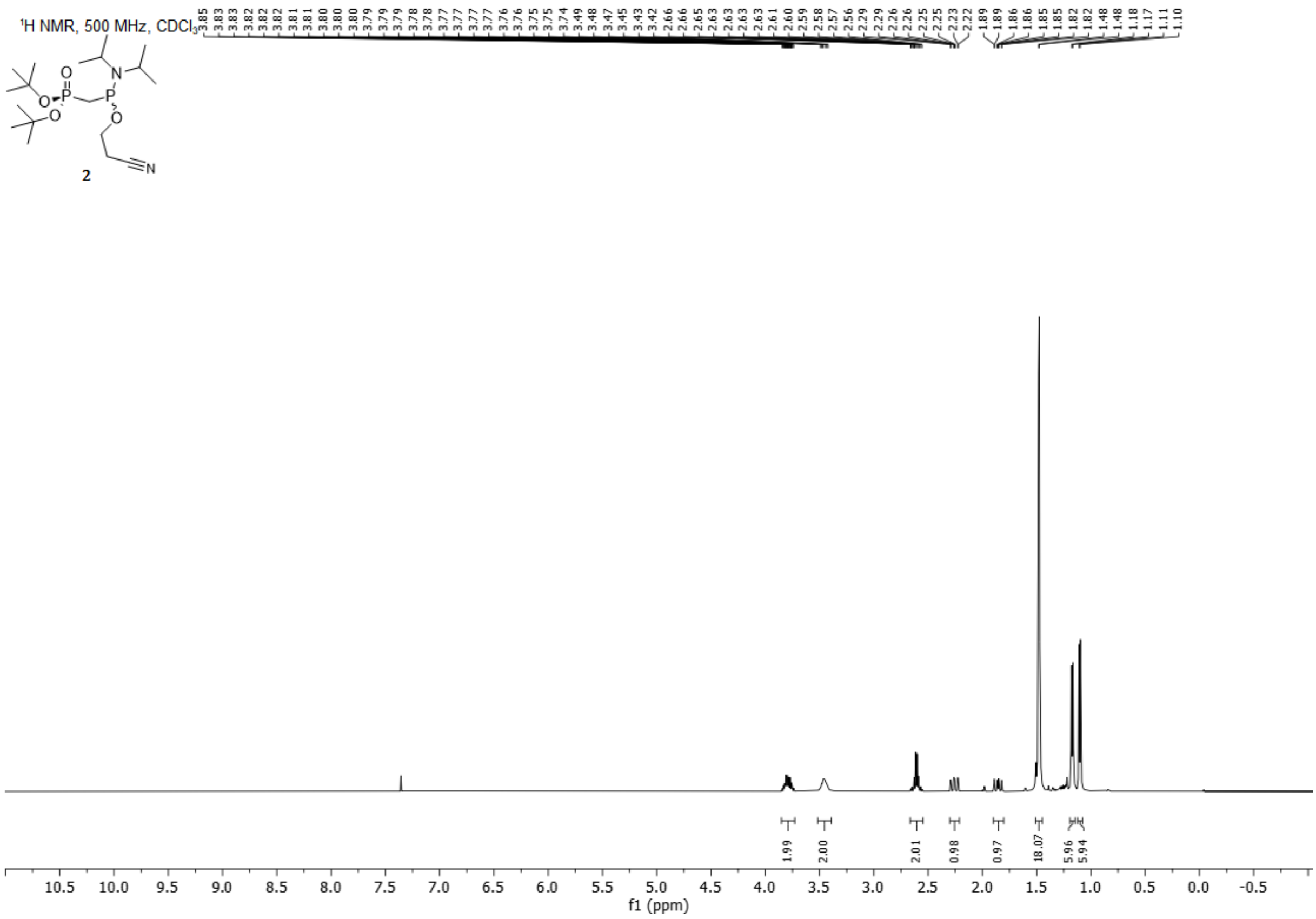

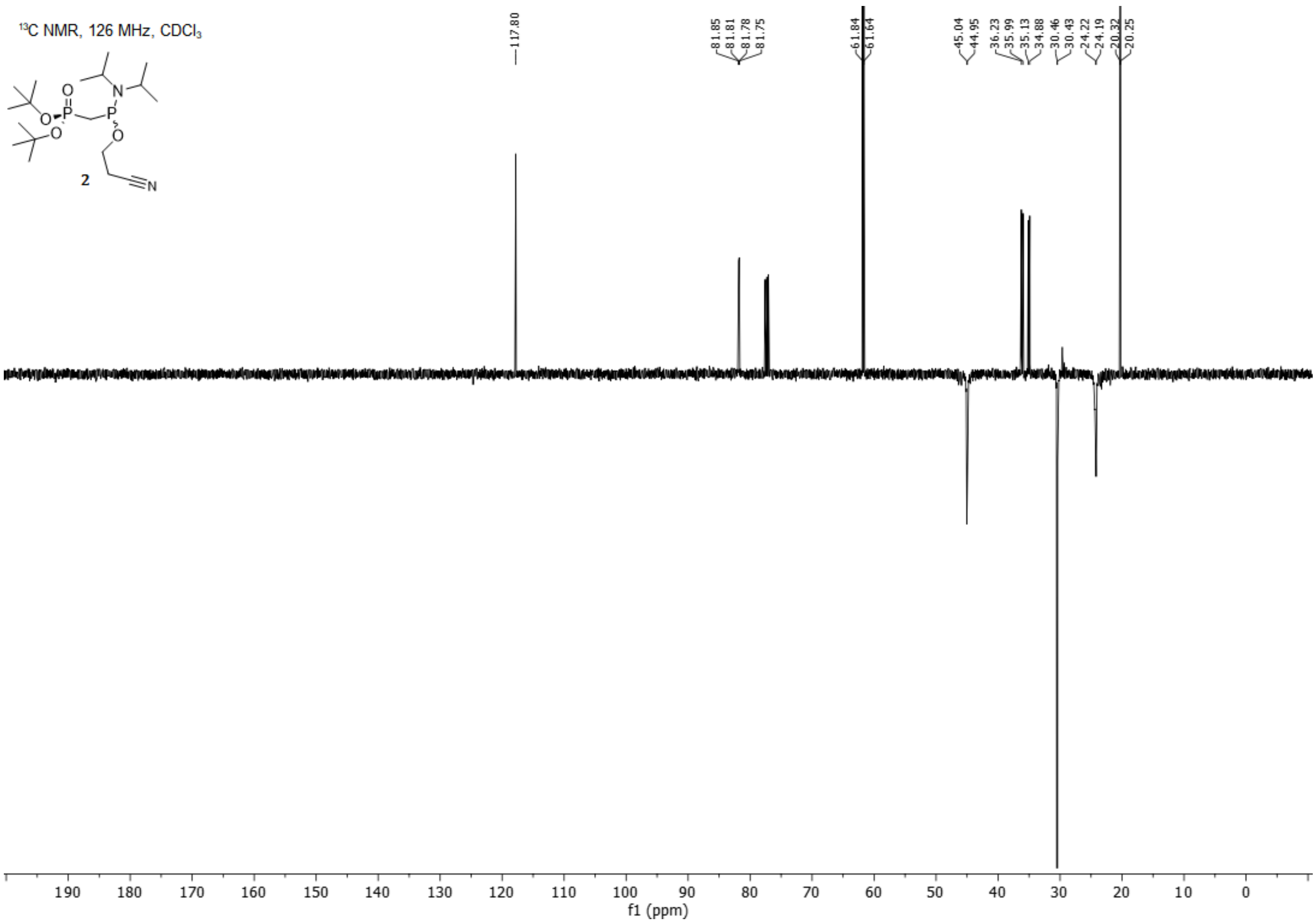

^{13}C NMR, 126 MHz, CDCl$_3$
2
117.80
81.85
81.81
81.78
81.75
61.84
61.64
45.04
44.95
36.23
35.99
35.13
34.88
30.46
30.43
24.22
24.19
20.32
20.25
190 180 170 160 150 140 130 120 110 100 90 80 70 60 50 40 30 20 10 0
f1 (ppm)

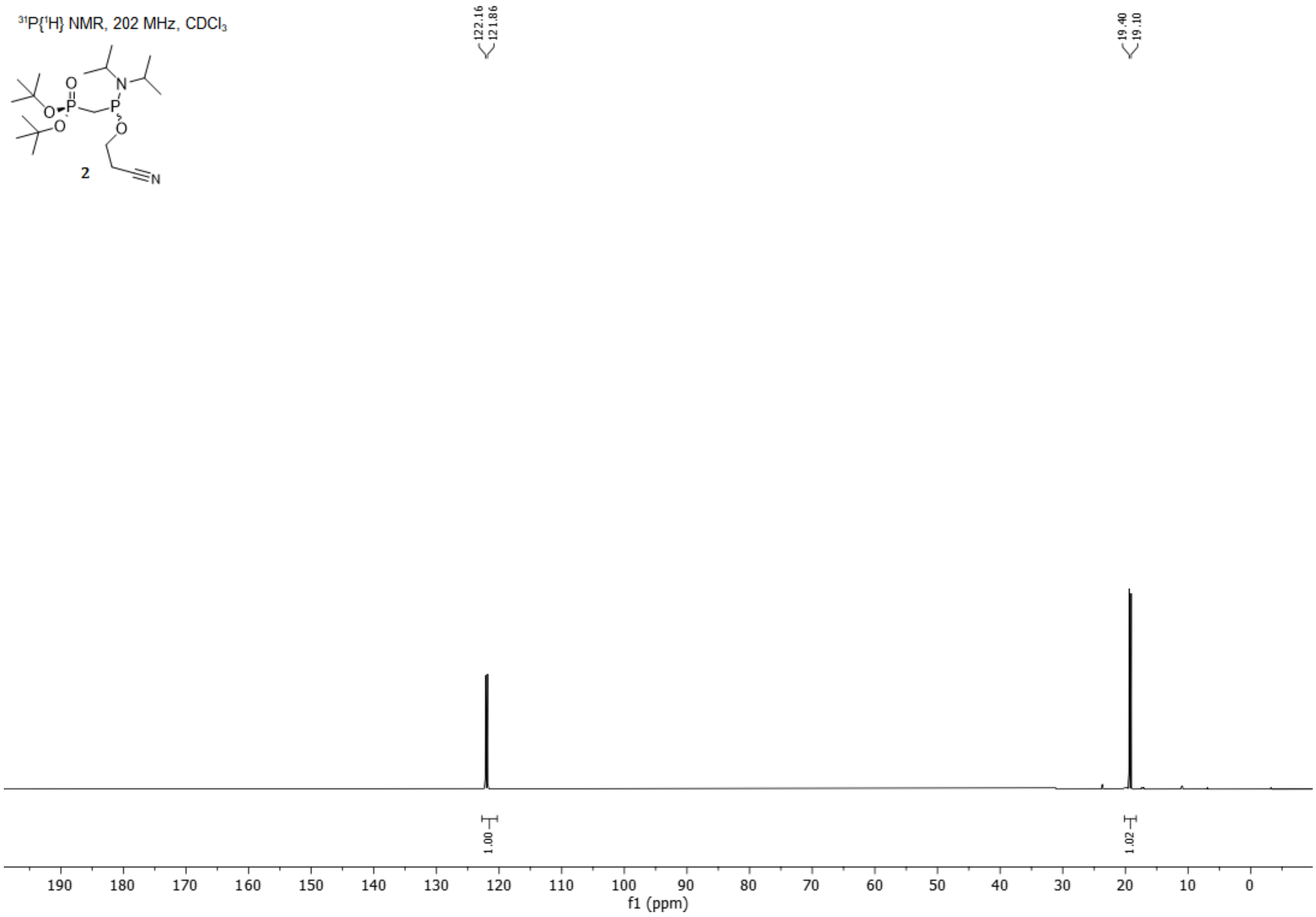

^{31}P{^{1}H} NMR, 202 MHz, CDCl$_3$
2
122.16
121.86
19.40
19.10
1.00
1.02
190 180 170 160 150 140 130 120 110 100 90 80 70 60 50 40 30 20 10 0
f1 (ppm)

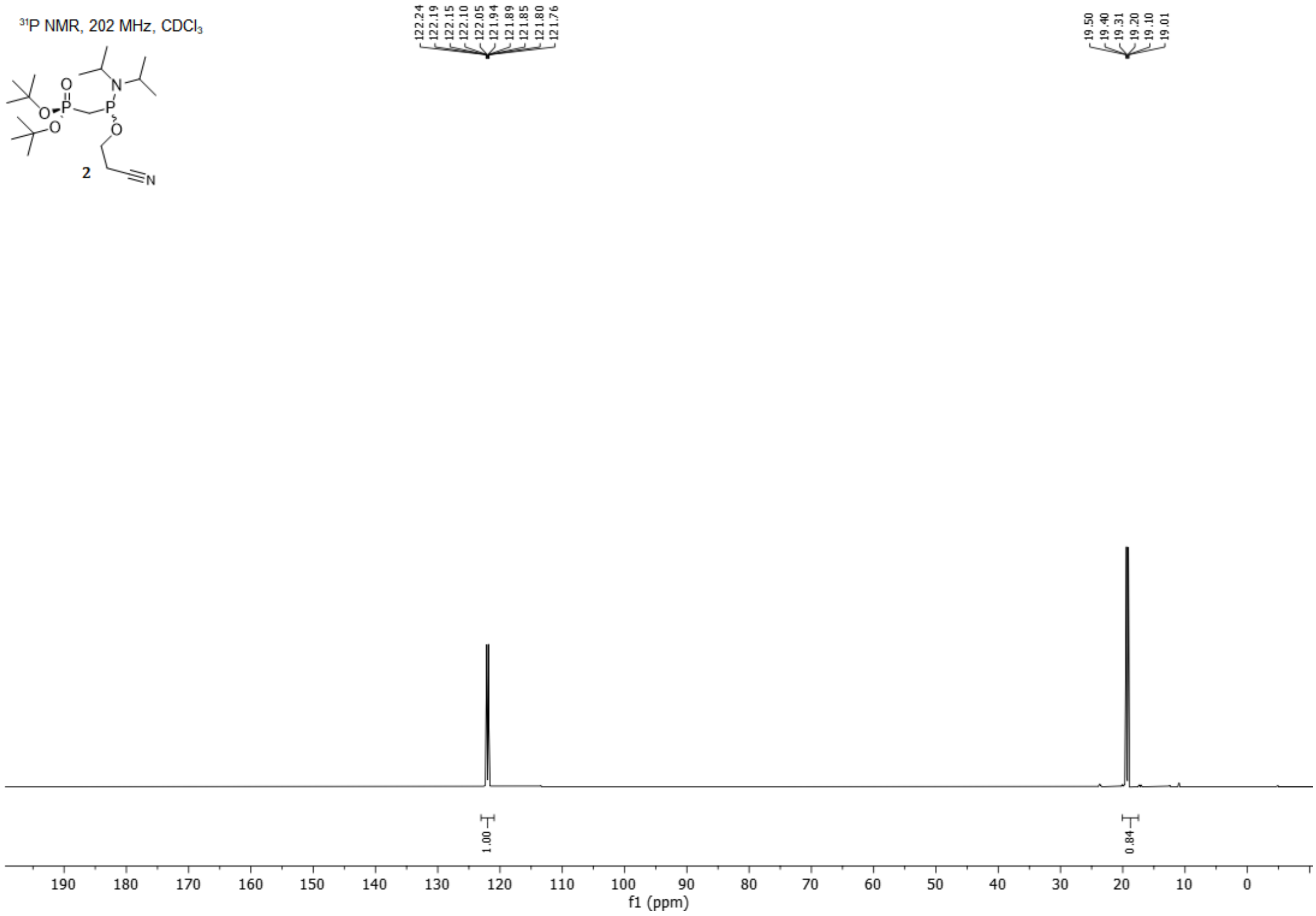

^{31}P NMR, 202 MHz, CDCl$_3$
2
122.24
122.19
122.15
122.10
122.05
121.94
121.89
121.85
121.80
121.76
19.50
19.40
19.31
19.20
19.10
19.01
1.00
0.84
f1 (ppm)

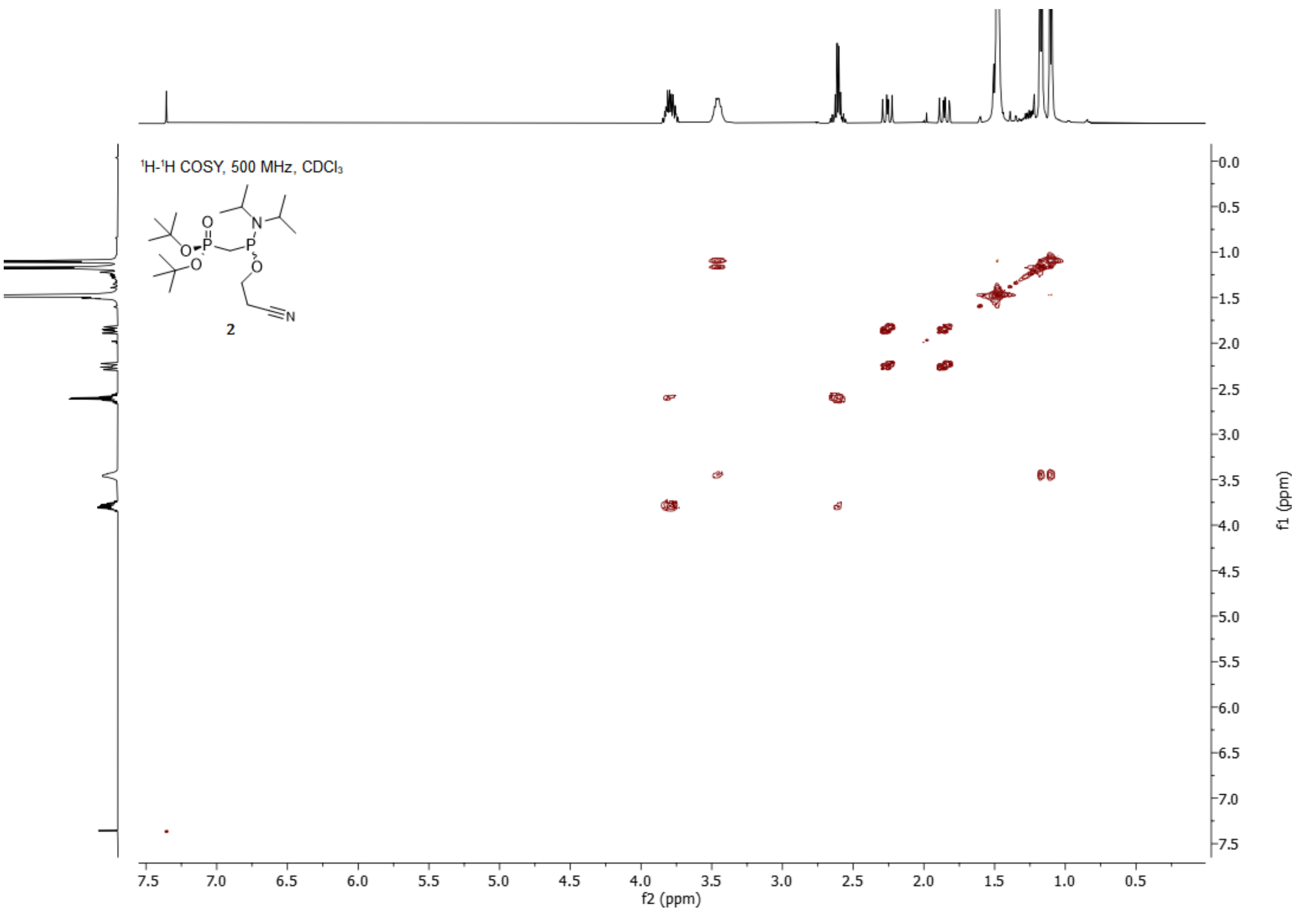

^{1}H-^{1}H COSY, 500 MHz, CDCl$_3$
2
f2 (ppm)
f1 (ppm)

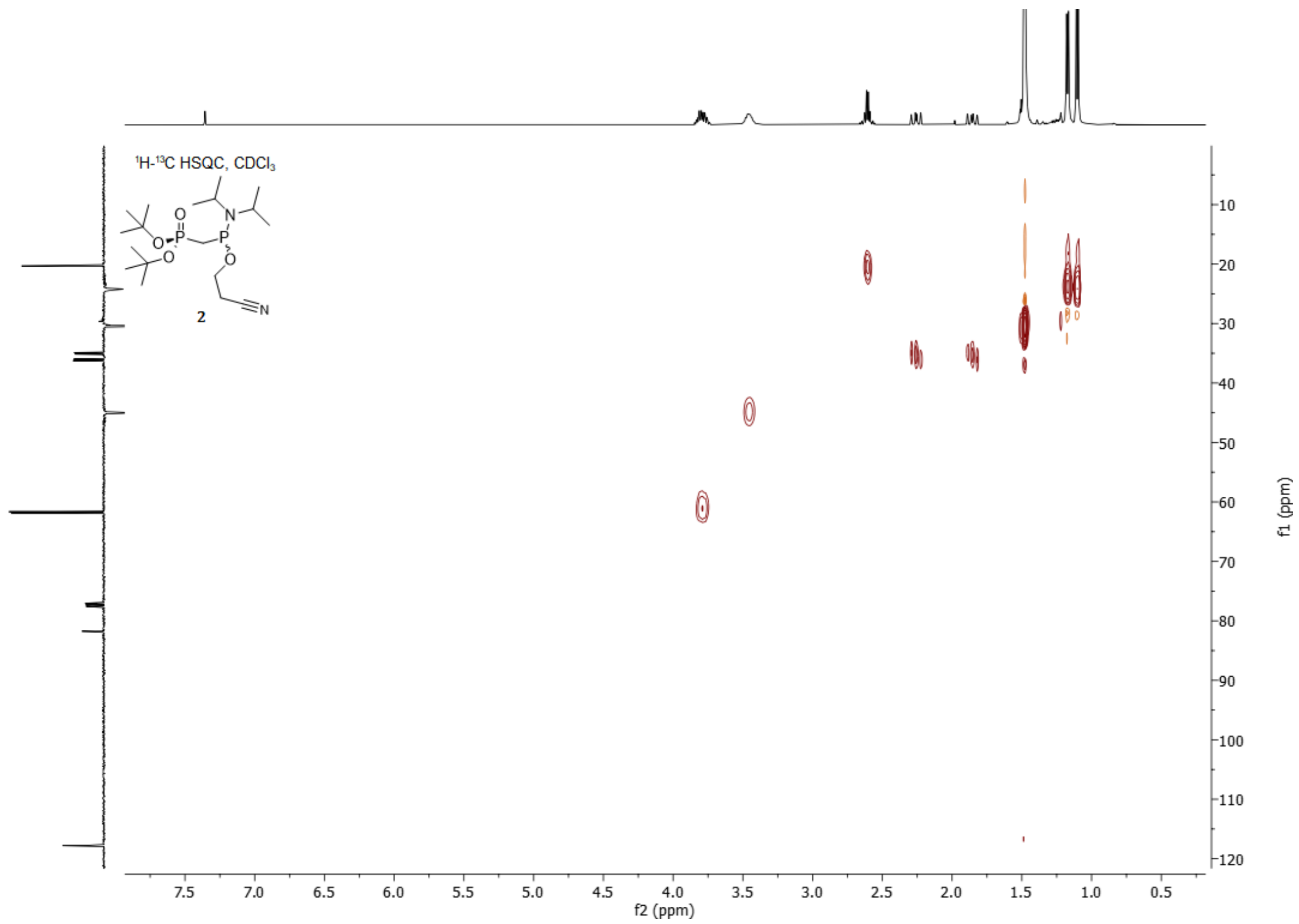

Phosphonomethyl Phosphonamidite 3

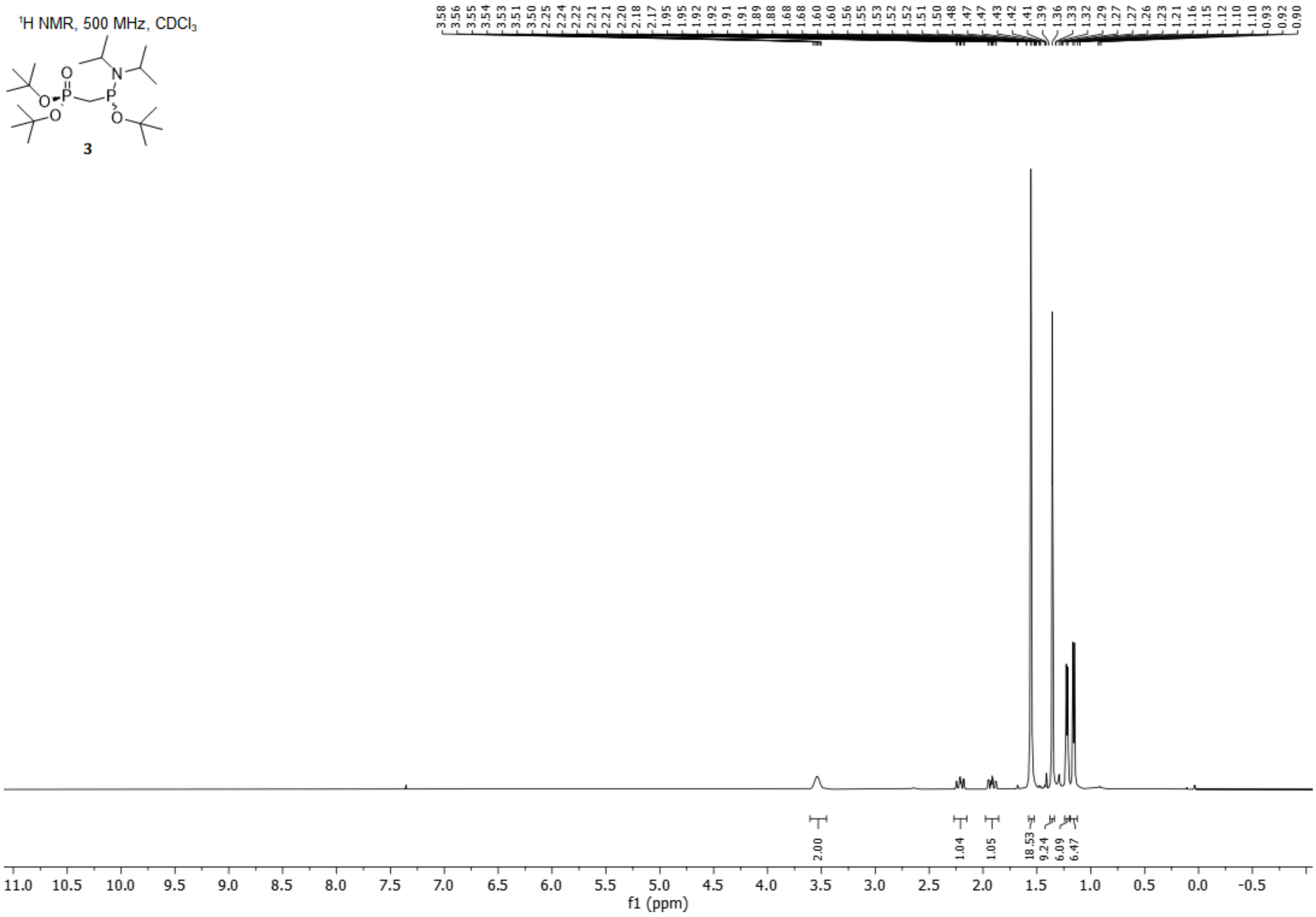

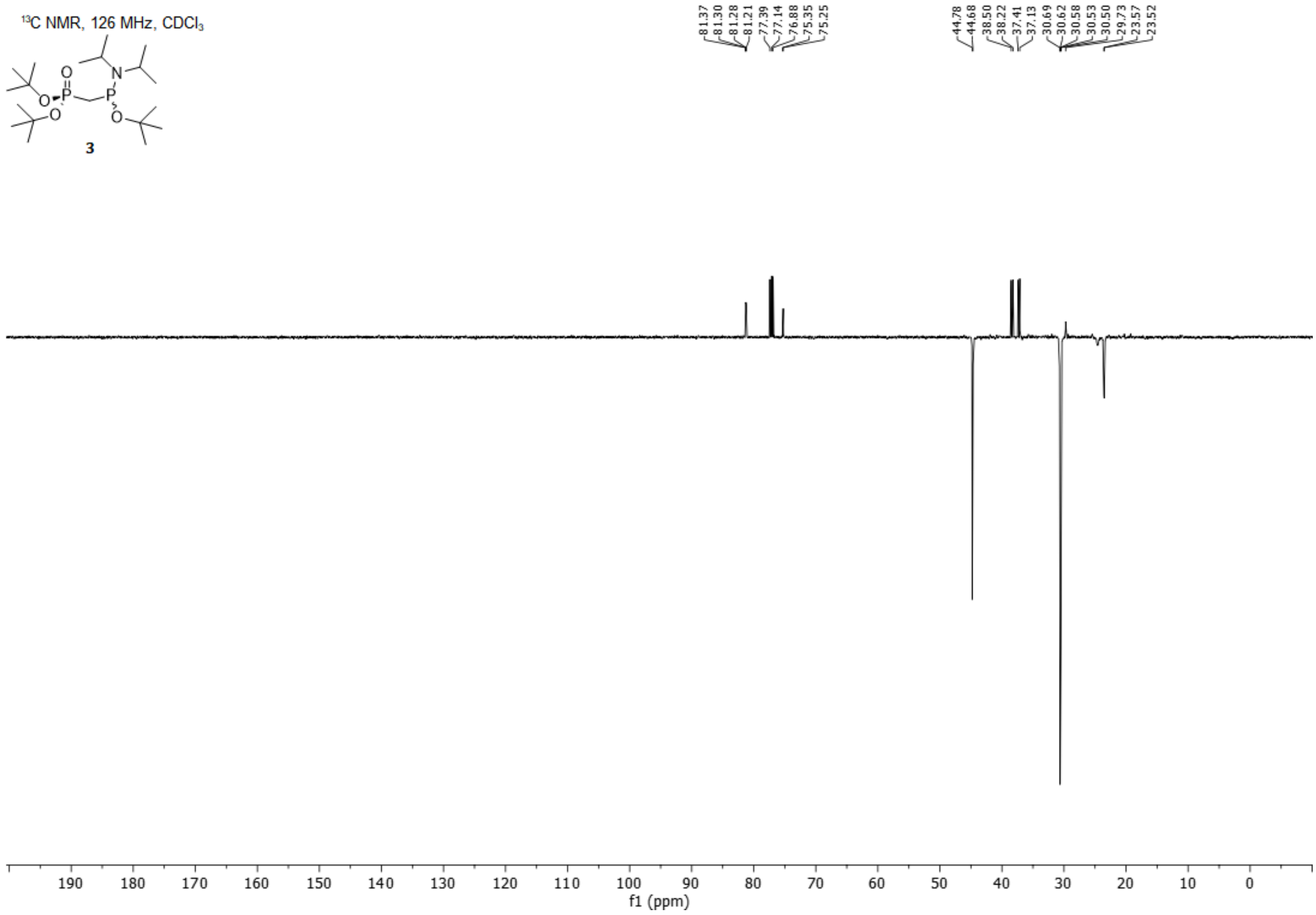

13C NMR, 126 MHz, CDCl3
3
81.37
81.30
81.28
81.21
77.39
77.14
76.88
75.35
75.25
44.78
44.68
38.50
38.22
37.41
37.13
30.69
30.62
30.58
30.53
30.50
29.73
23.57
23.52
190 180 170 160 150 140 130 120 110 100 90 80 70 60 50 40 30 20 10 0
f1 (ppm)

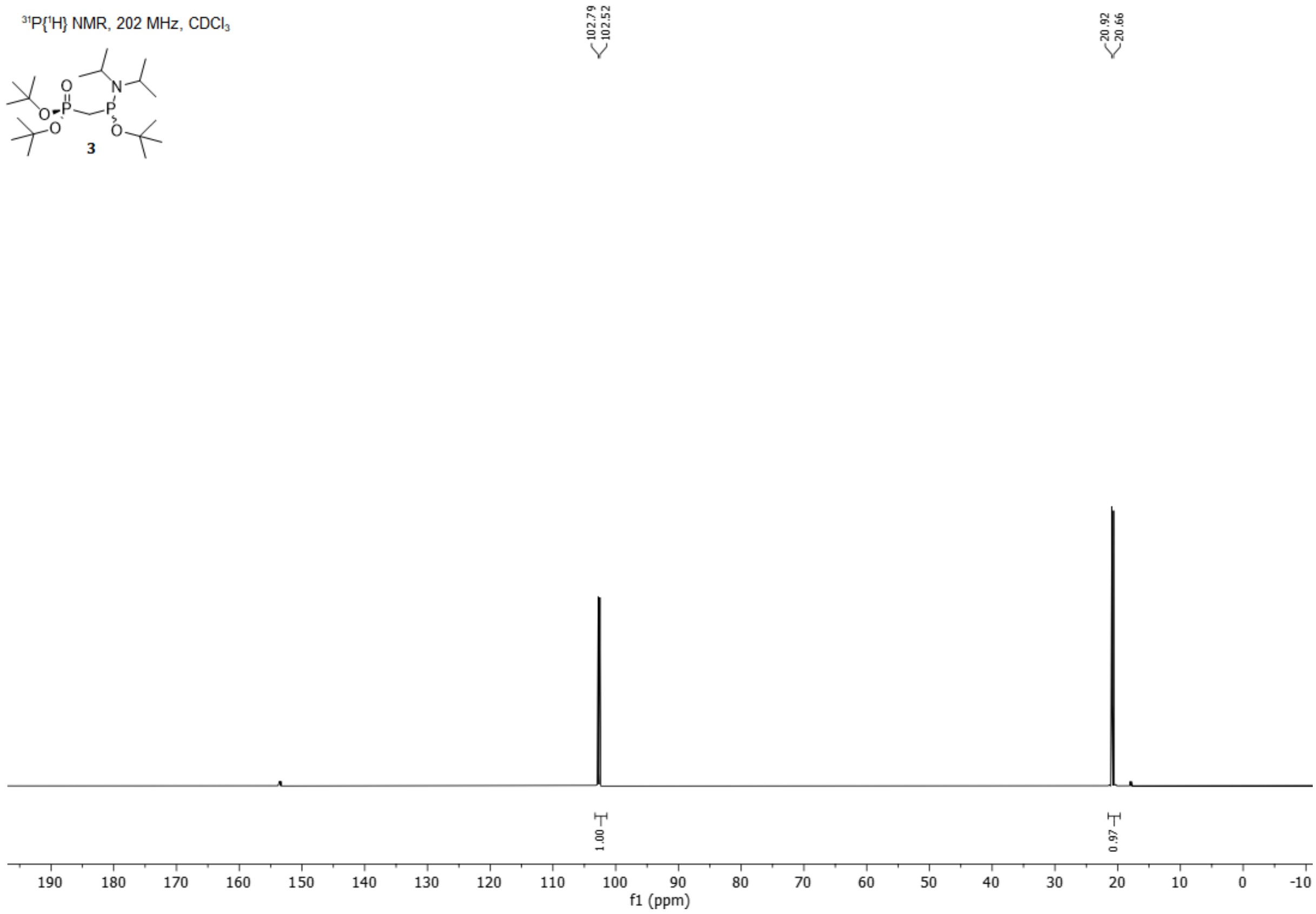

31P{1H} NMR, 202 MHz, CDCl3
3
102.79
102.52
20.92
20.66
1.00
0.97
190 180 170 160 150 140 130 120 110 100 90 80 70 60 50 40 30 20 10 0 -10
f1 (ppm)

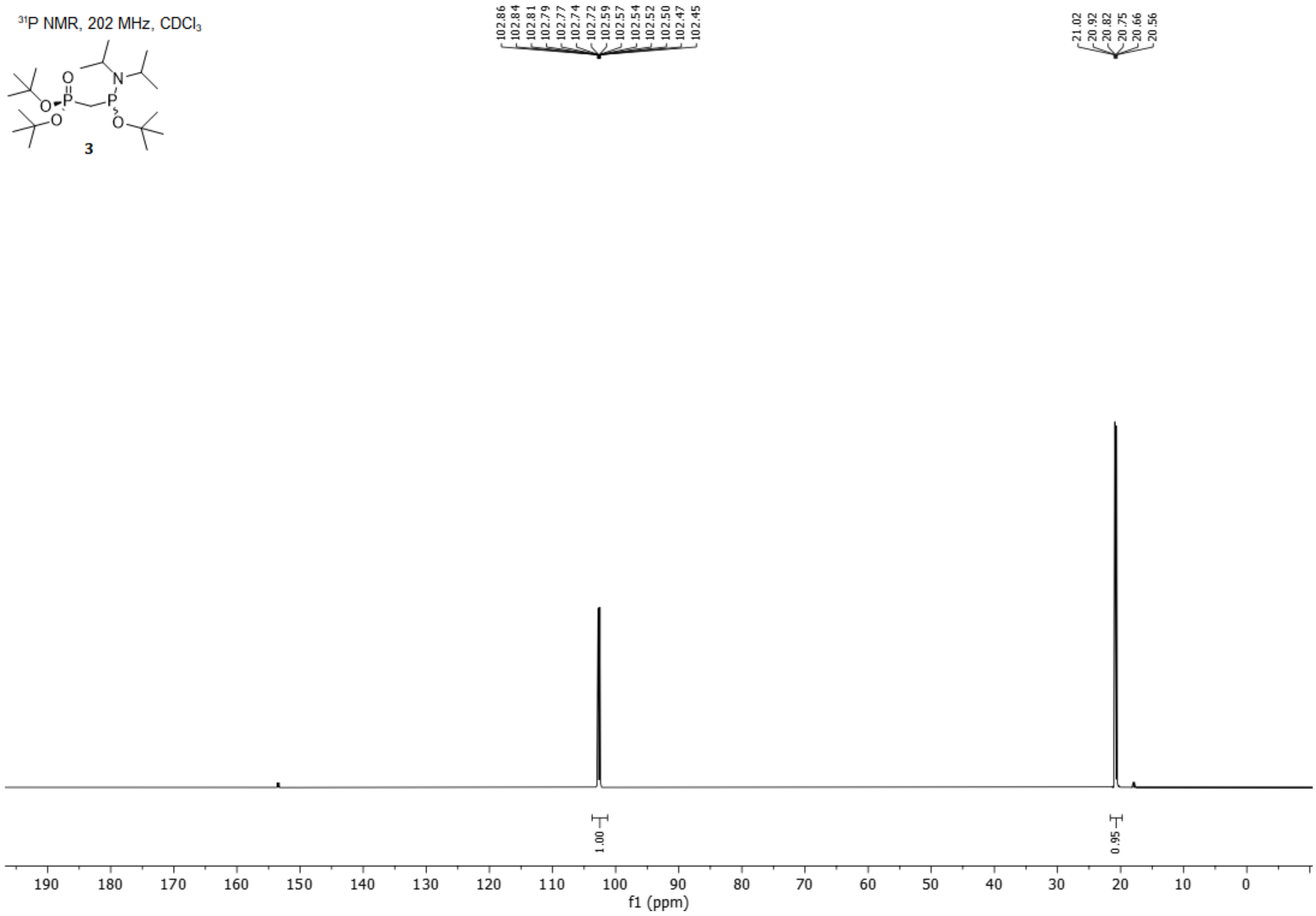

31P NMR, 202 MHz, CDCl3
3
102.86
102.84
102.81
102.79
102.77
102.74
102.72
102.59
102.57
102.54
102.52
102.50
102.47
102.45
21.02
20.92
20.82
20.75
20.66
20.56
1.00
0.95
f1 (ppm)

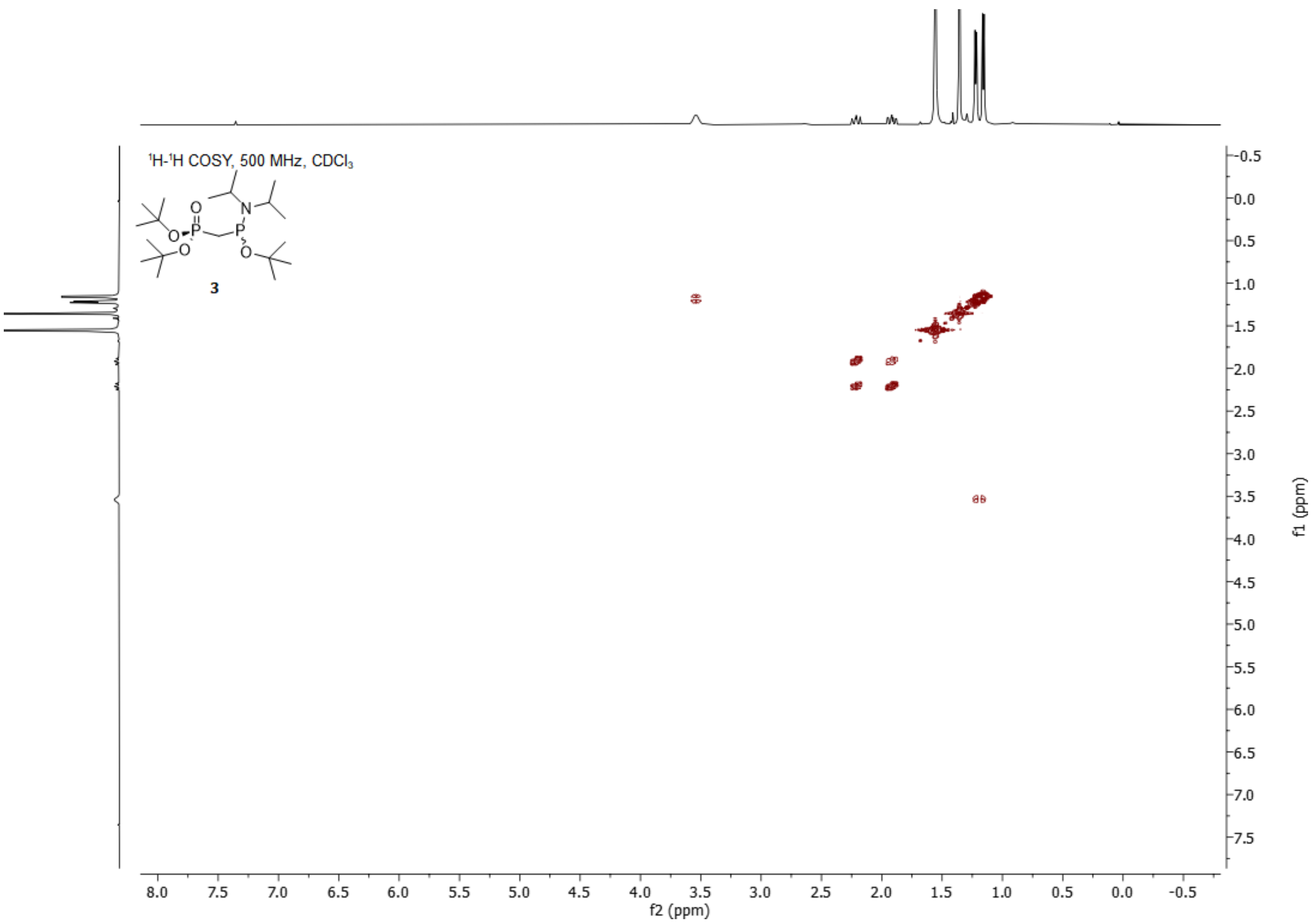

1H-1H COSY, 500 MHz, CDCl3
3
f2 (ppm)
f1 (ppm)

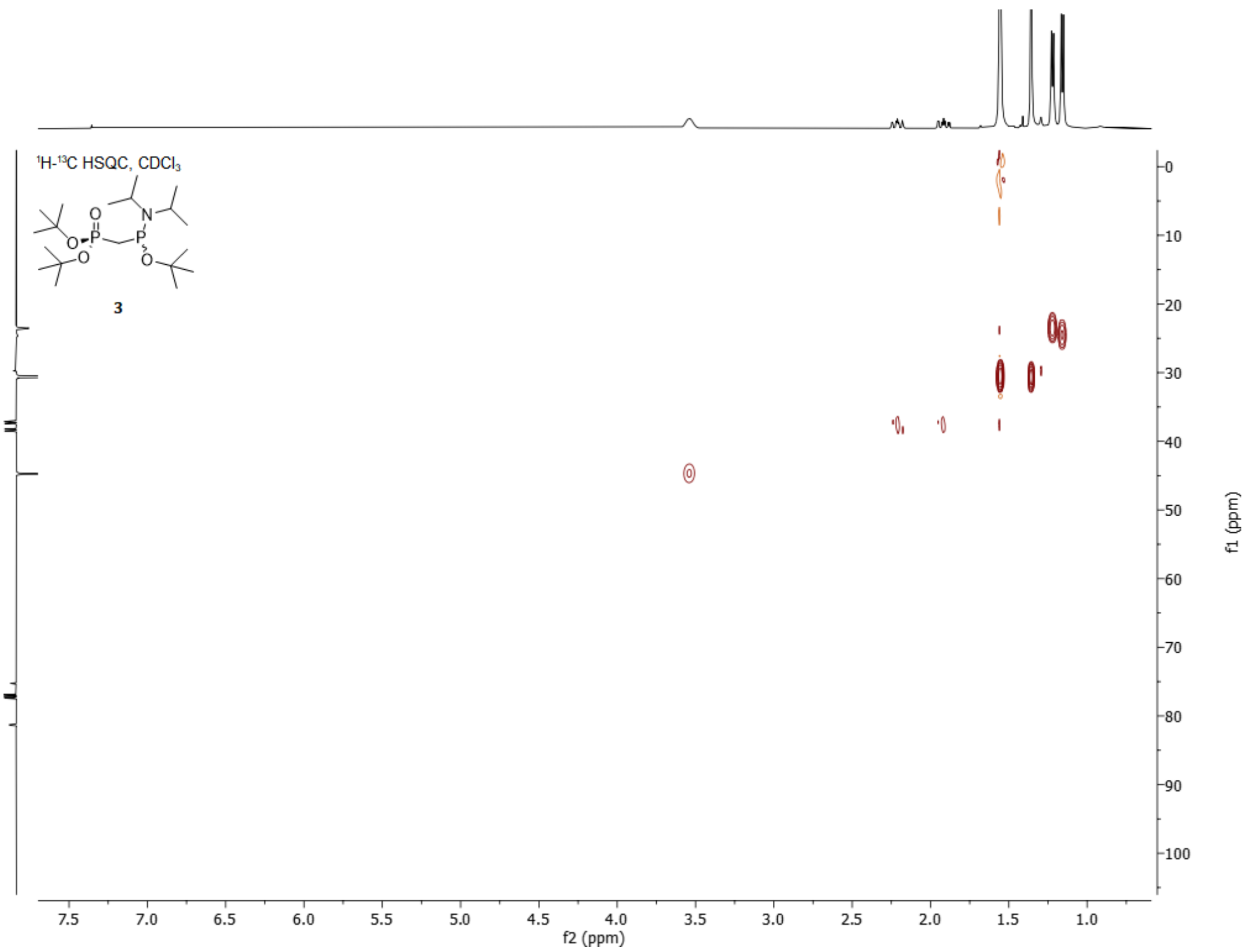

Phosphonomethyl Phosphonamidite 4

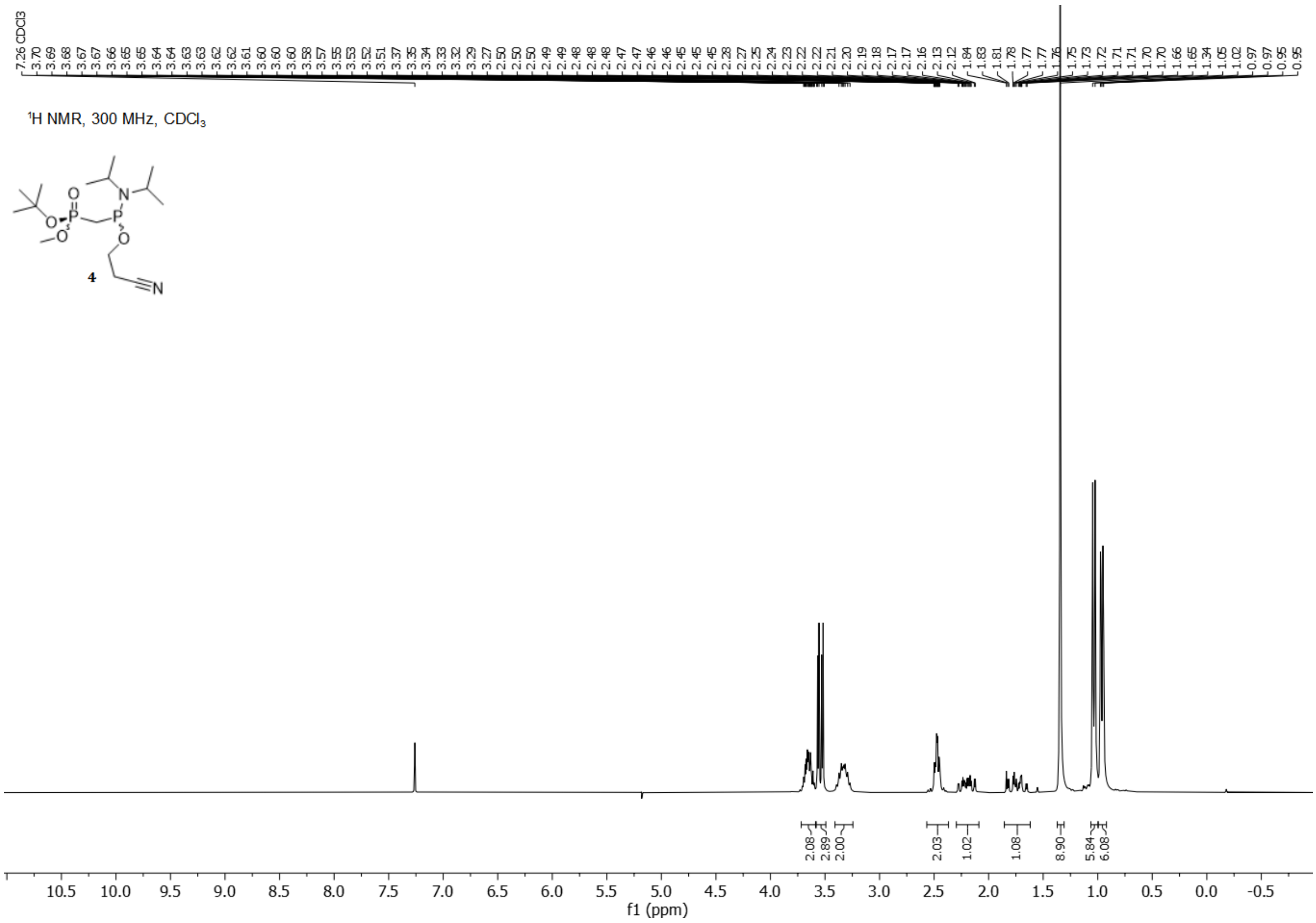

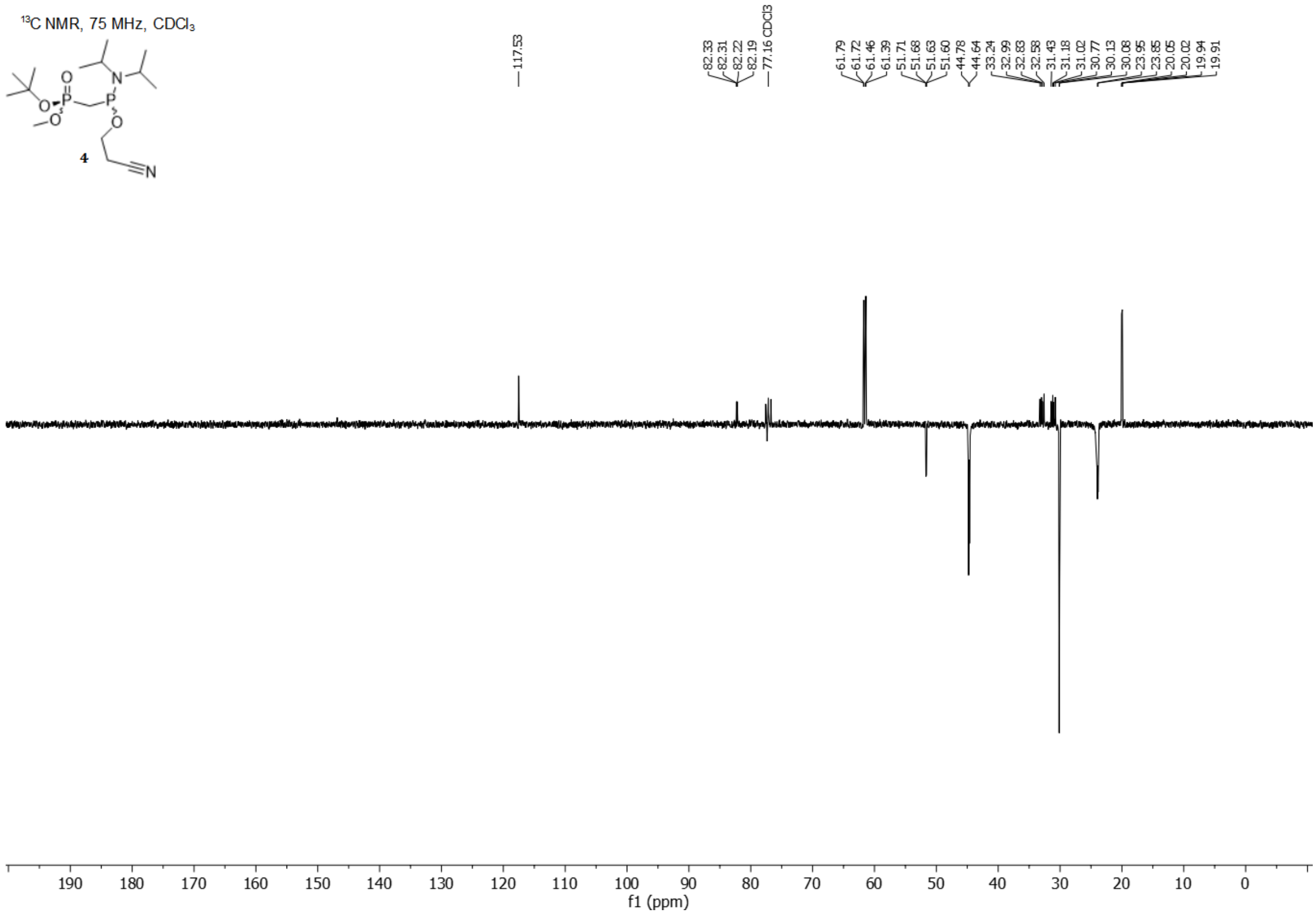

13C NMR, 75 MHz, CDCl3
4
117.53
82.33
82.31
82.22
82.19
77.16 CDCl3
61.79
61.72
61.46
61.39
51.71
51.68
51.63
51.60
44.78
44.64
33.24
32.99
32.83
32.58
31.43
31.18
31.02
30.77
30.13
30.08
23.95
23.85
20.05
20.02
19.94
19.91
f1 (ppm)

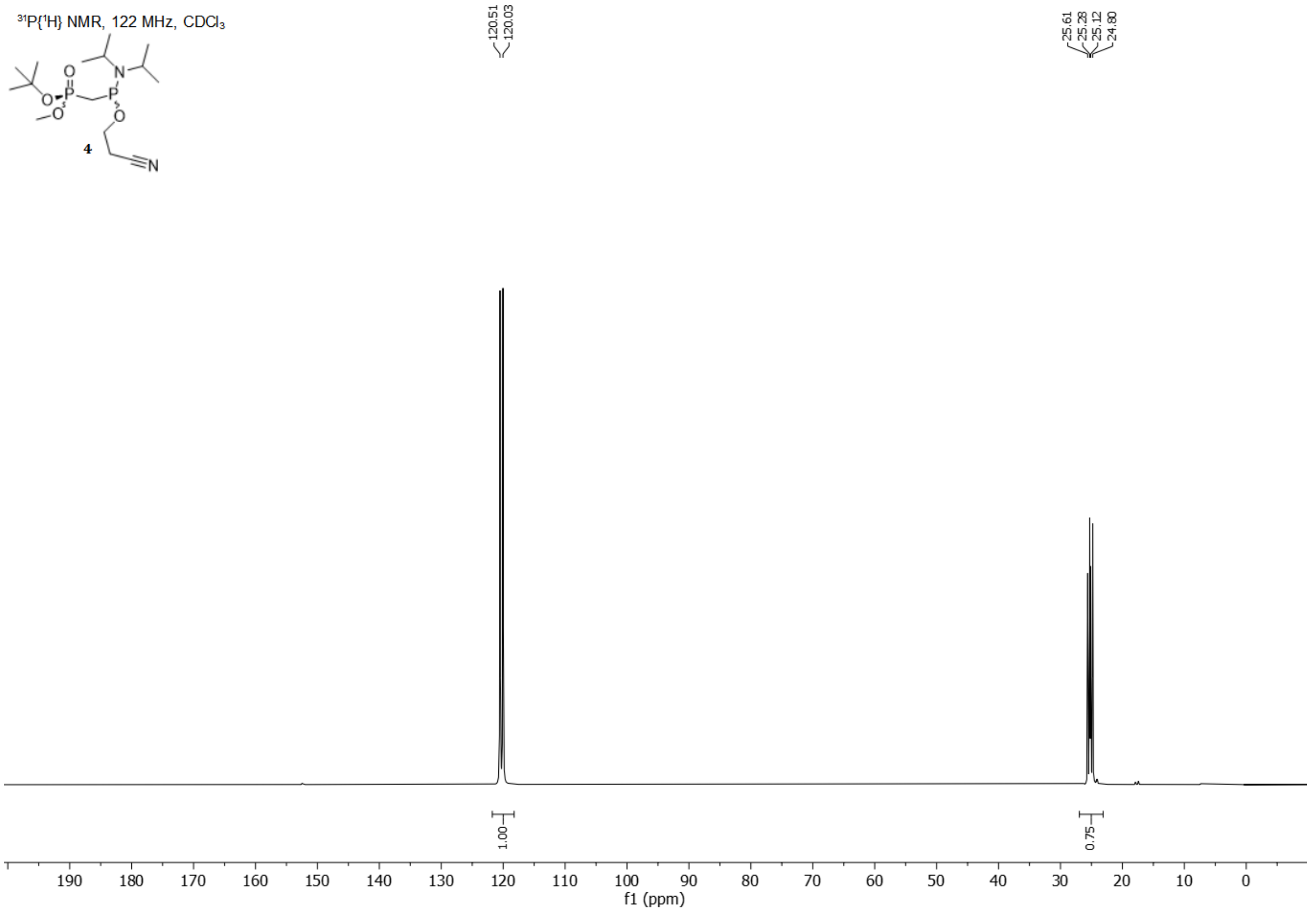

31P{1H} NMR, 122 MHz, CDCl3
4
120.51
120.03
25.61
25.28
25.12
24.80
1.00
0.75
f1 (ppm)

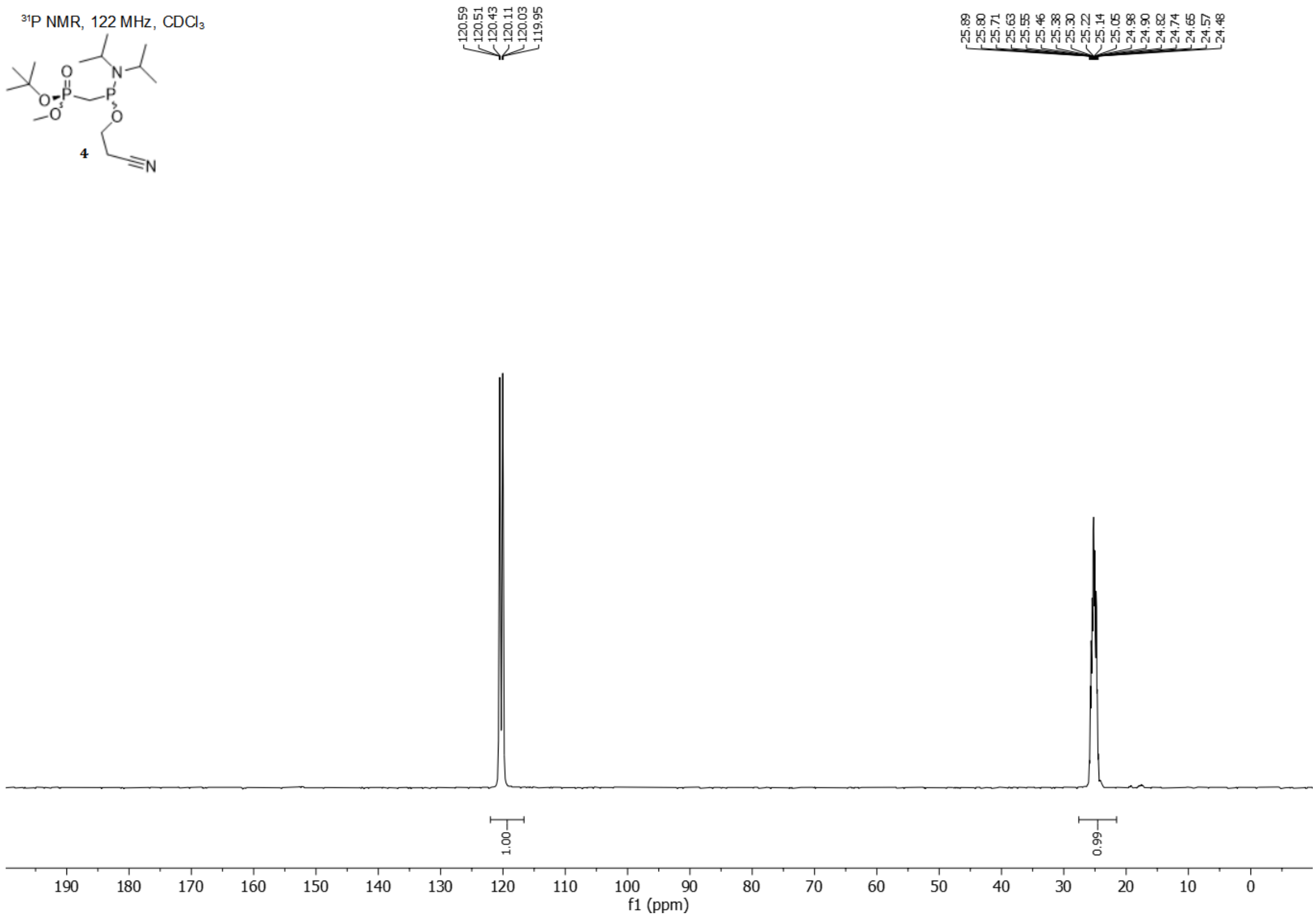
31P NMR, 122 MHz, CDCl3
4
120.59
120.51
120.43
120.11
120.03
119.95
25.89
25.80
25.71
25.63
25.55
25.46
25.38
25.30
25.22
25.14
25.05
24.98
24.90
24.82
24.74
24.65
24.57
24.48
1.00
0.99
f1 (ppm)

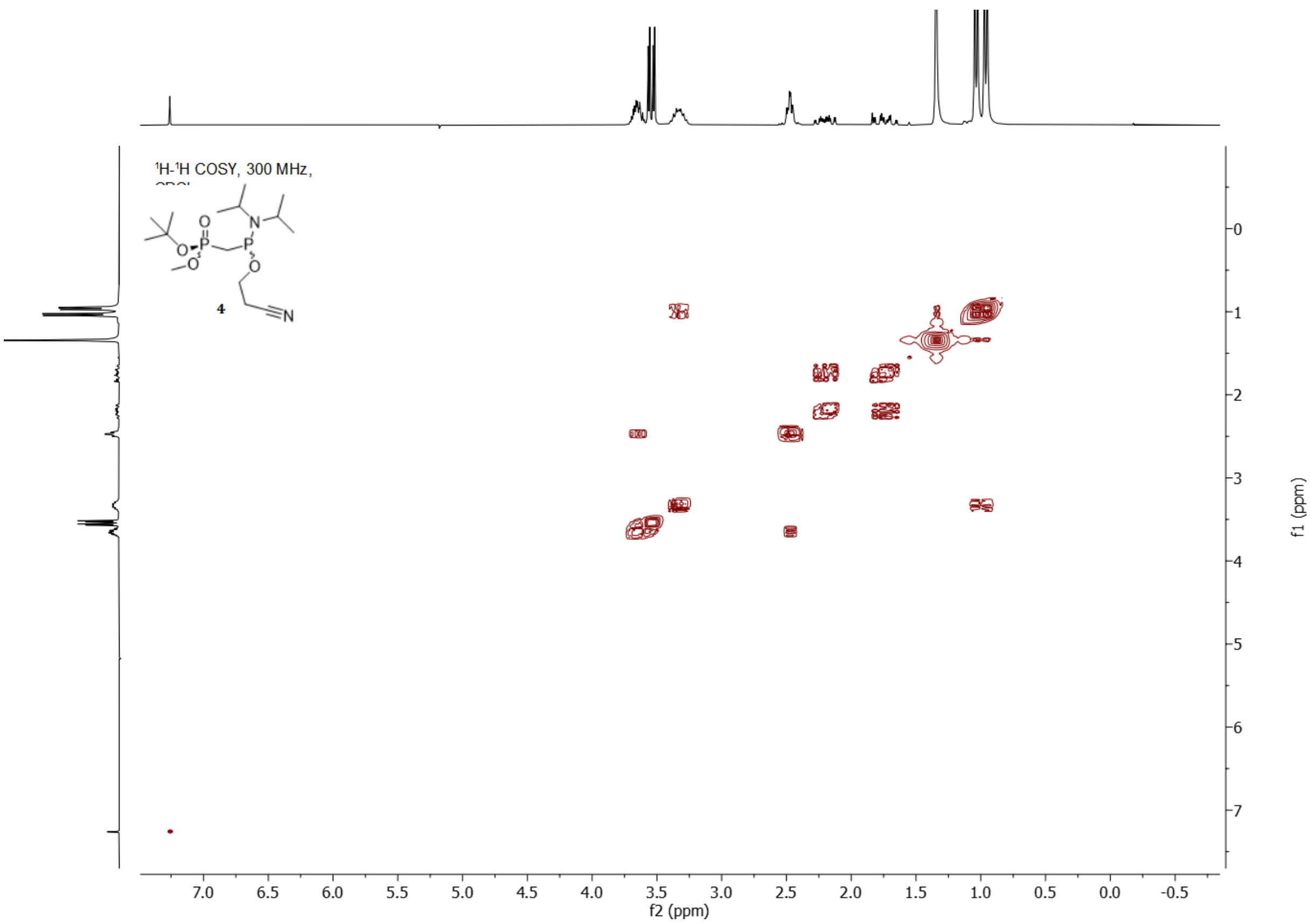
1H-1H COSY, 300 MHz,
4
f2 (ppm)
f1 (ppm)

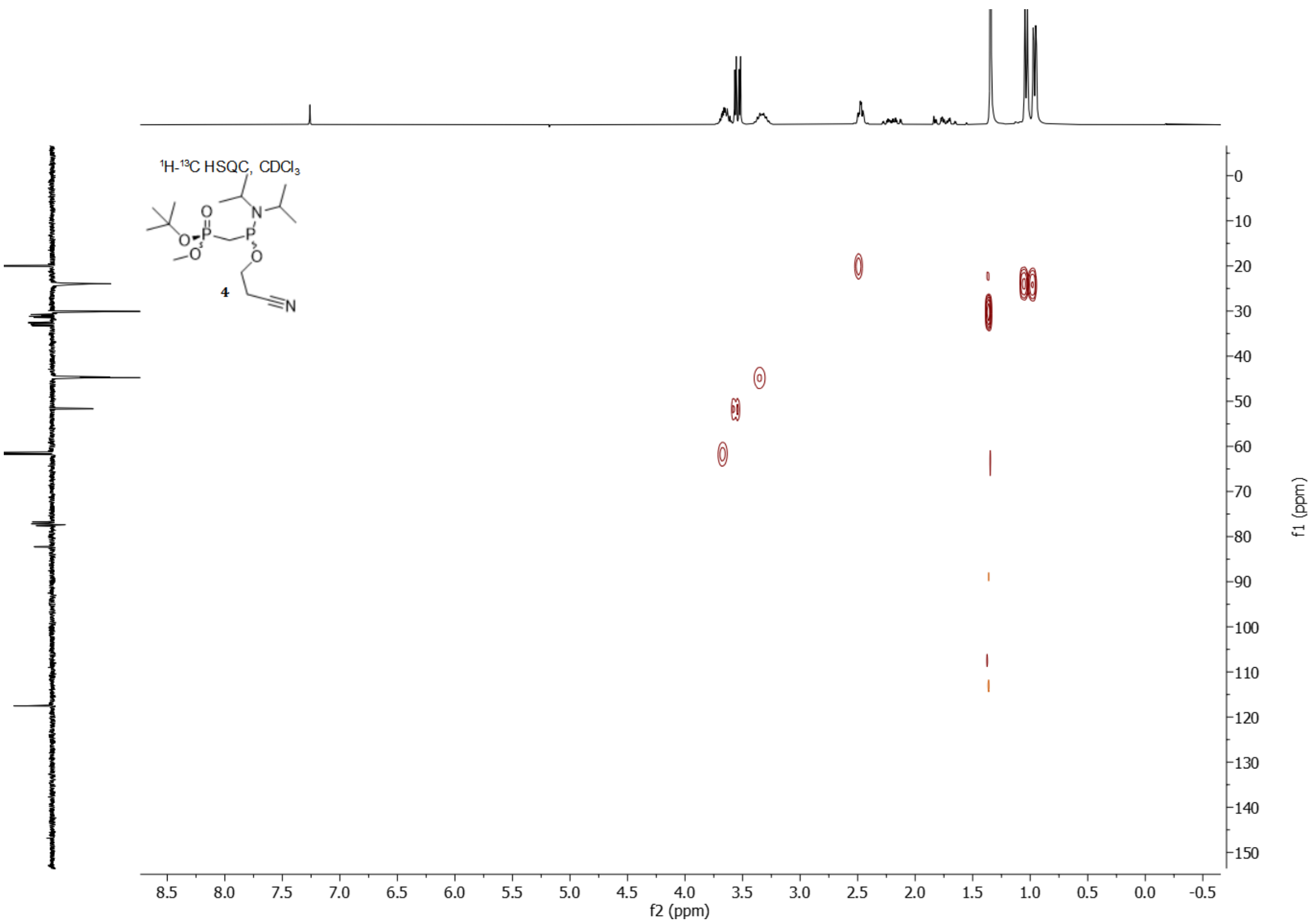

Phosphonomethyl Thiophosphonamidite 5

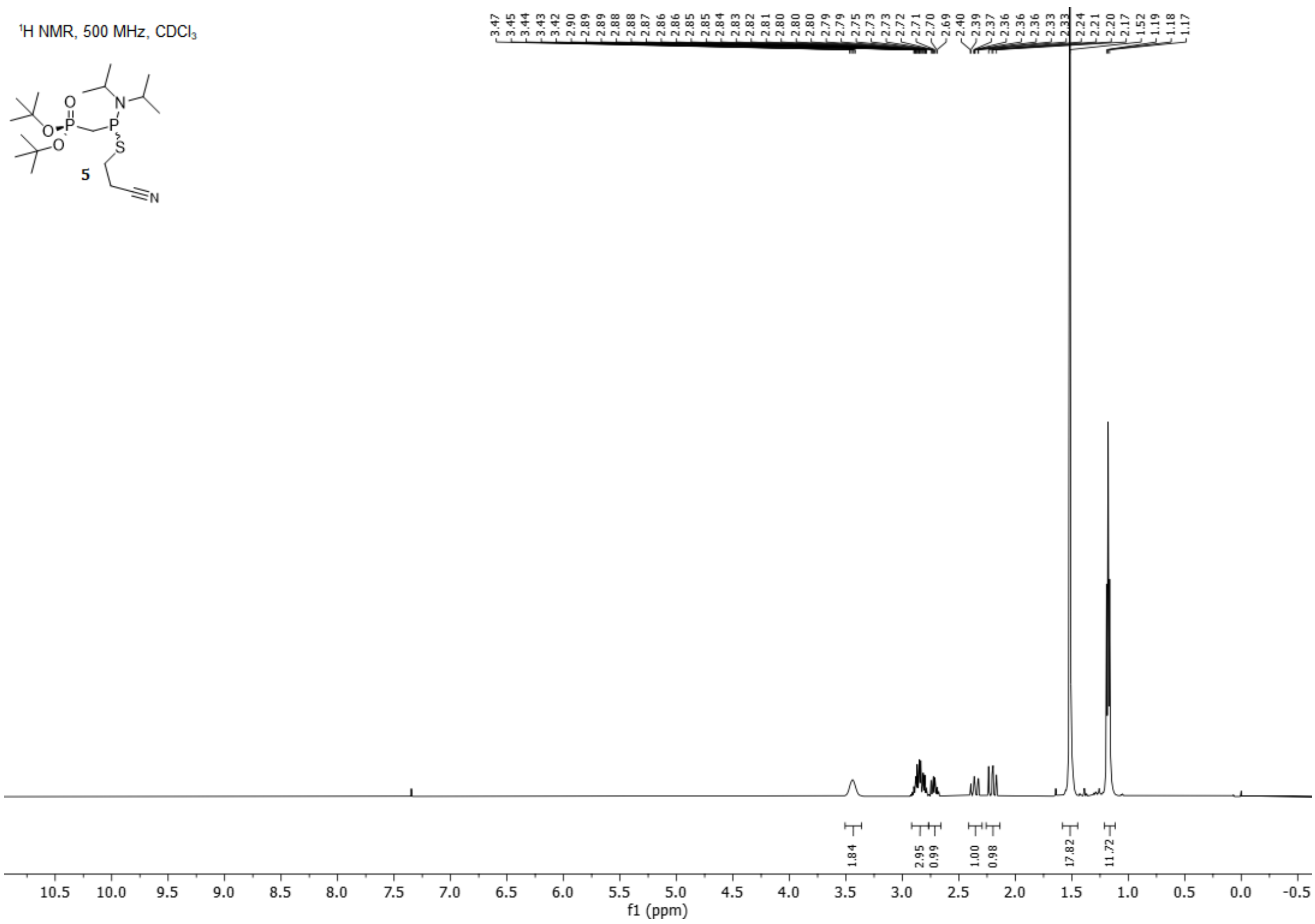

^{1}H NMR, 500 MHz, $CDCl_3$

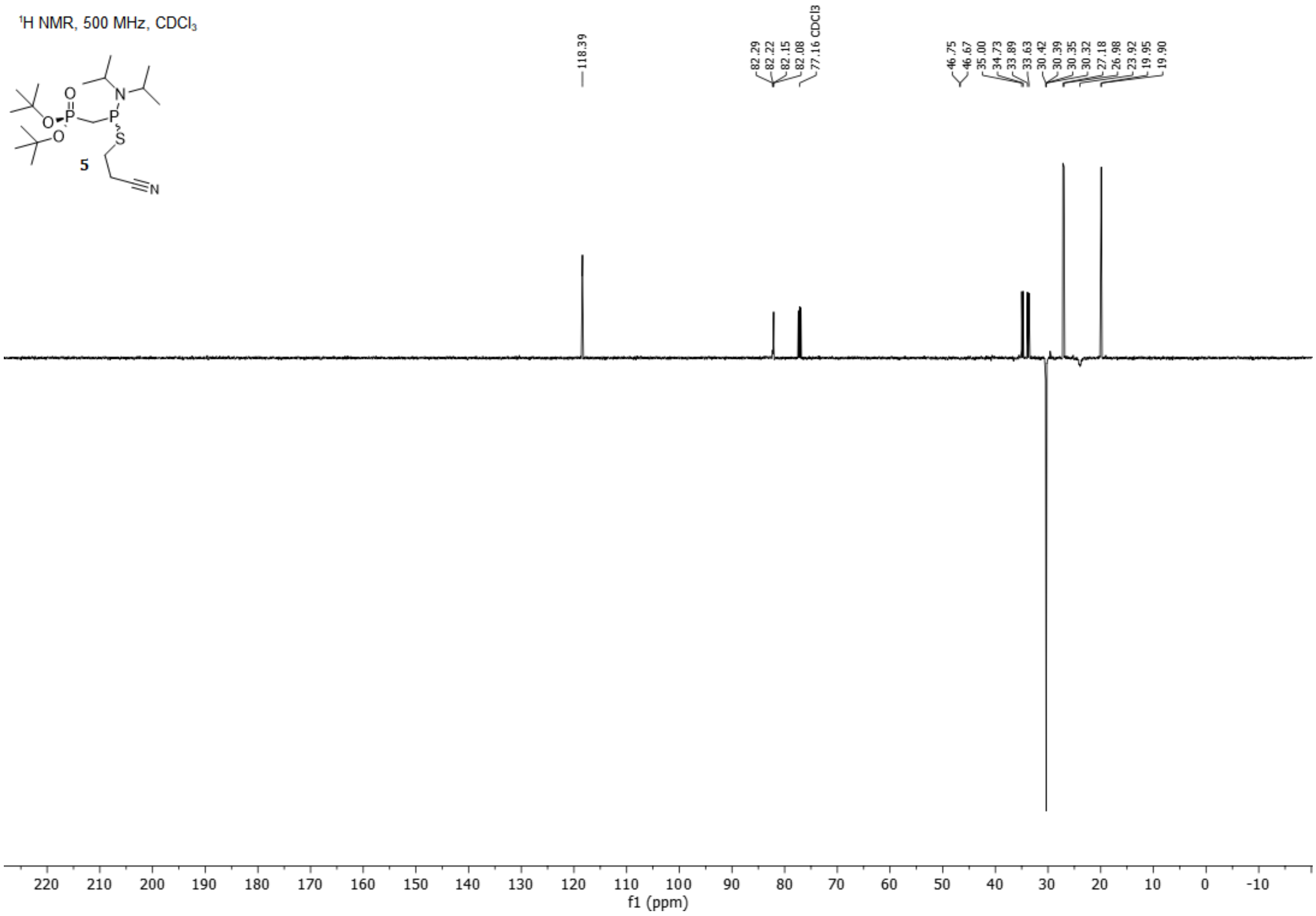

$^{31}P\{^{1}H\}$ NMR, 202 MHz, $CDCl_3$

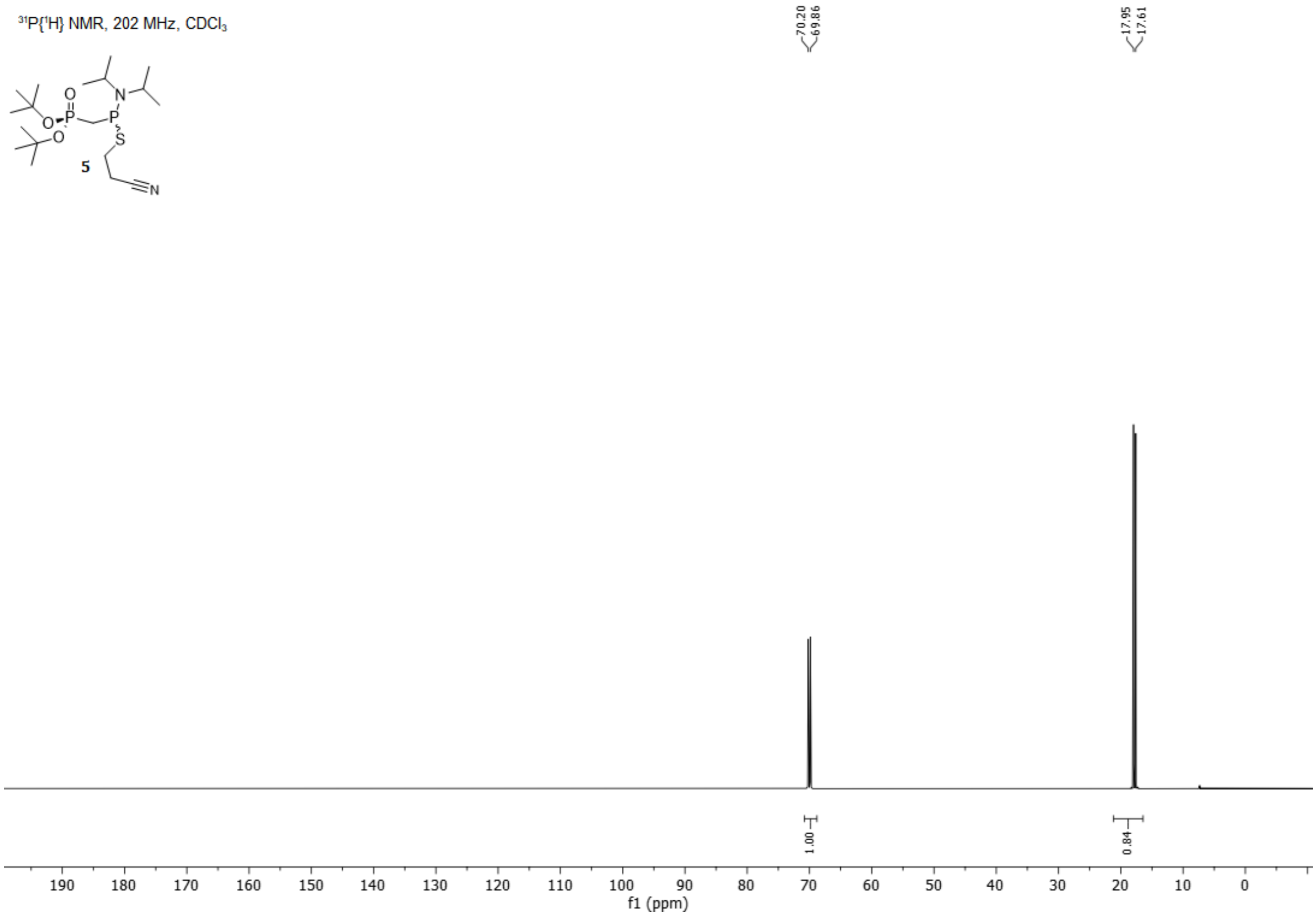

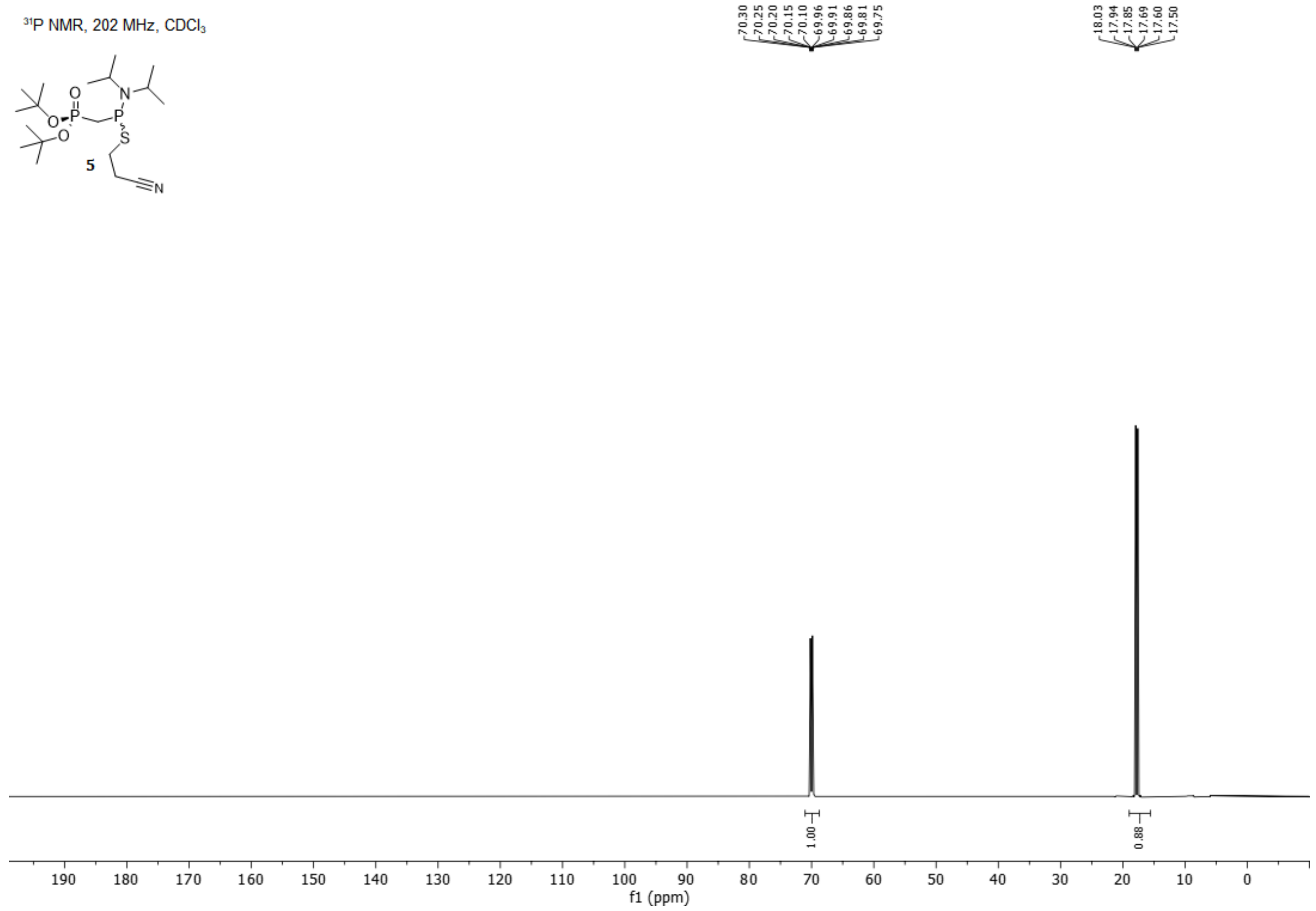

31P NMR, 202 MHz, CDCl3
5
70.30
70.25
70.20
70.15
70.10
69.96
69.91
69.86
69.81
69.75
18.03
17.94
17.85
17.69
17.60
17.50
1.00
0.88
f1 (ppm)

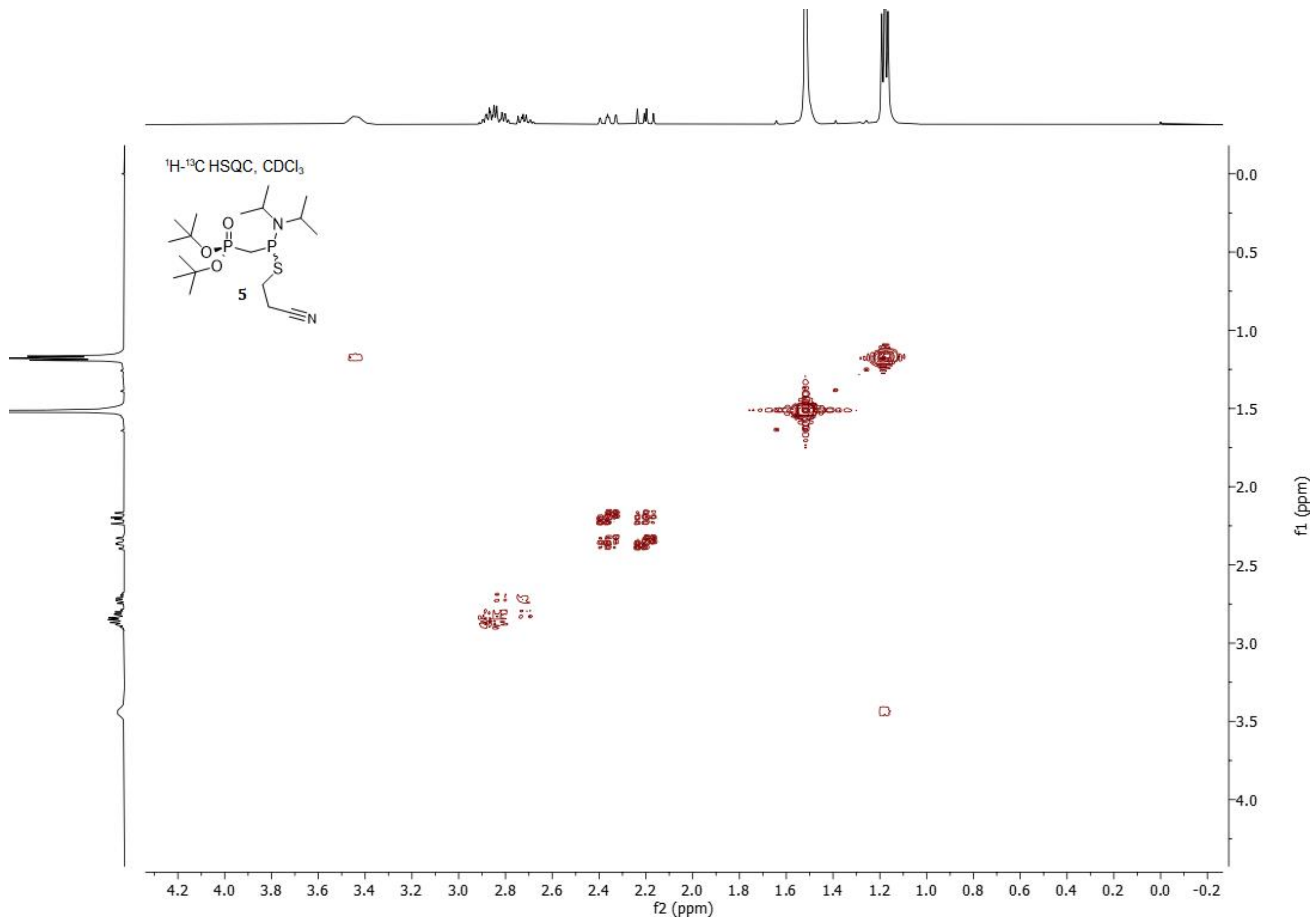

1H-13C HSQC, CDCl3
5
f2 (ppm)
f1 (ppm)

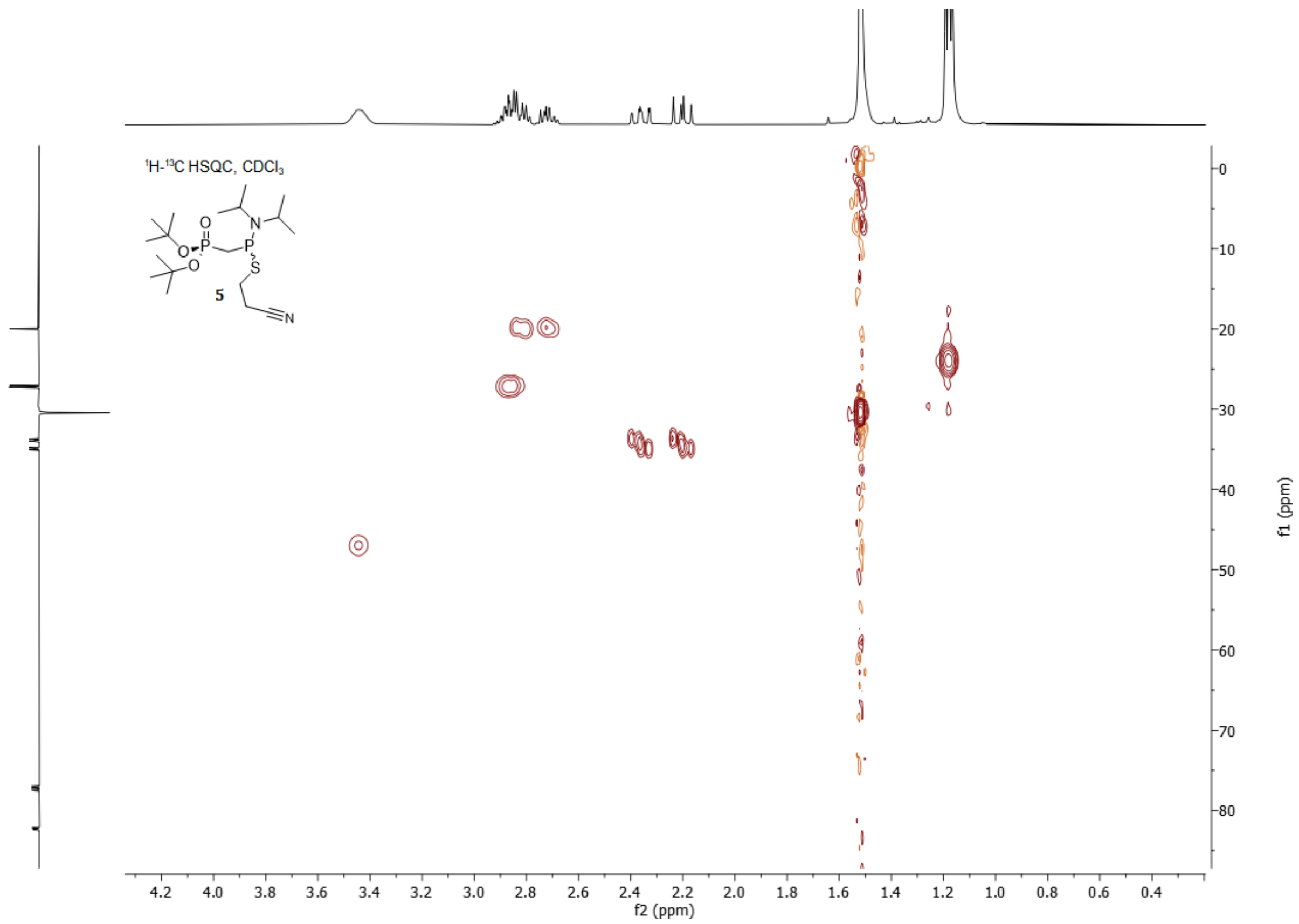

Phosphonomethyl Thiophosphonamidite 6

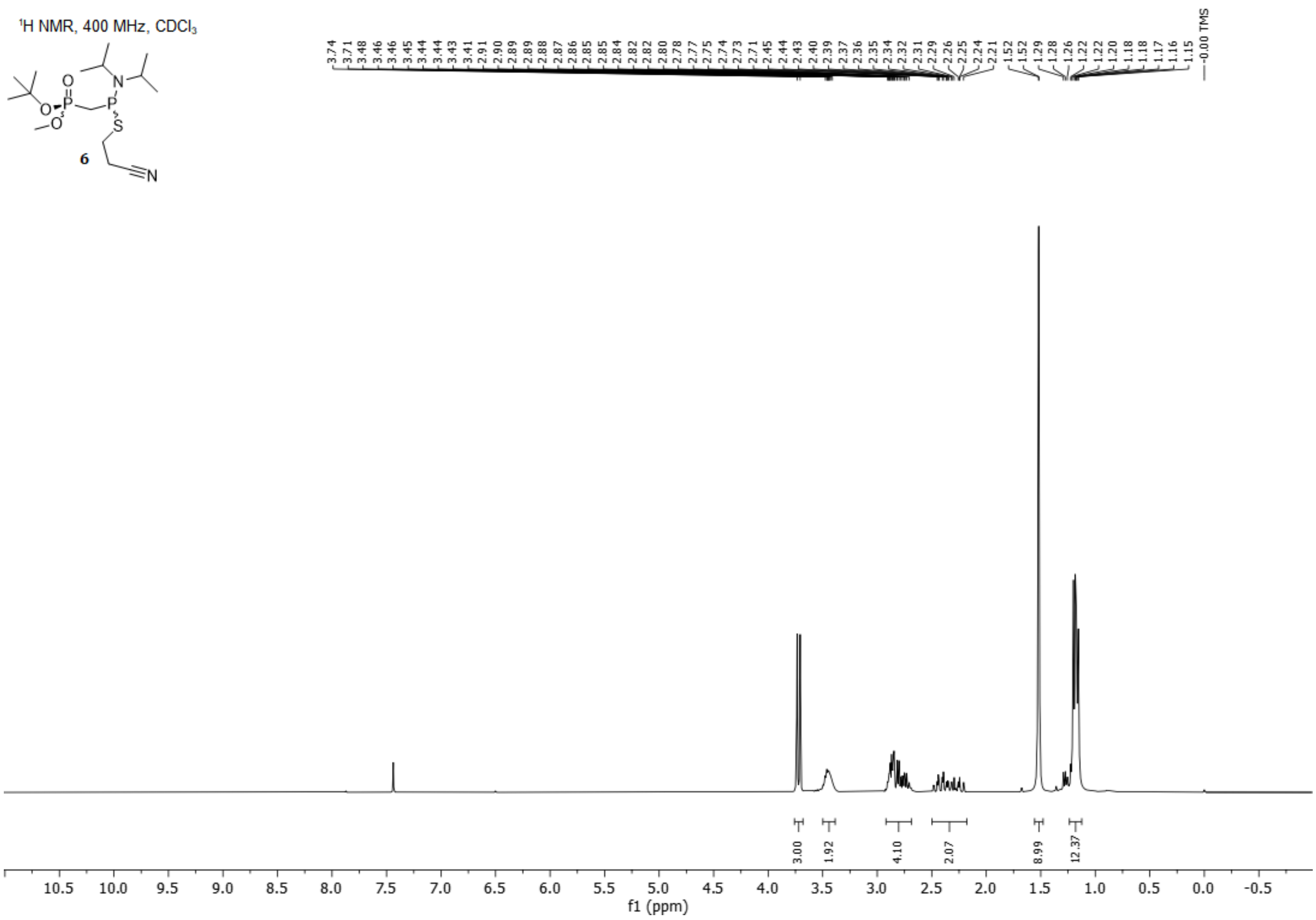

^{13}C NMR, 101 MHz, $CDCl_3$

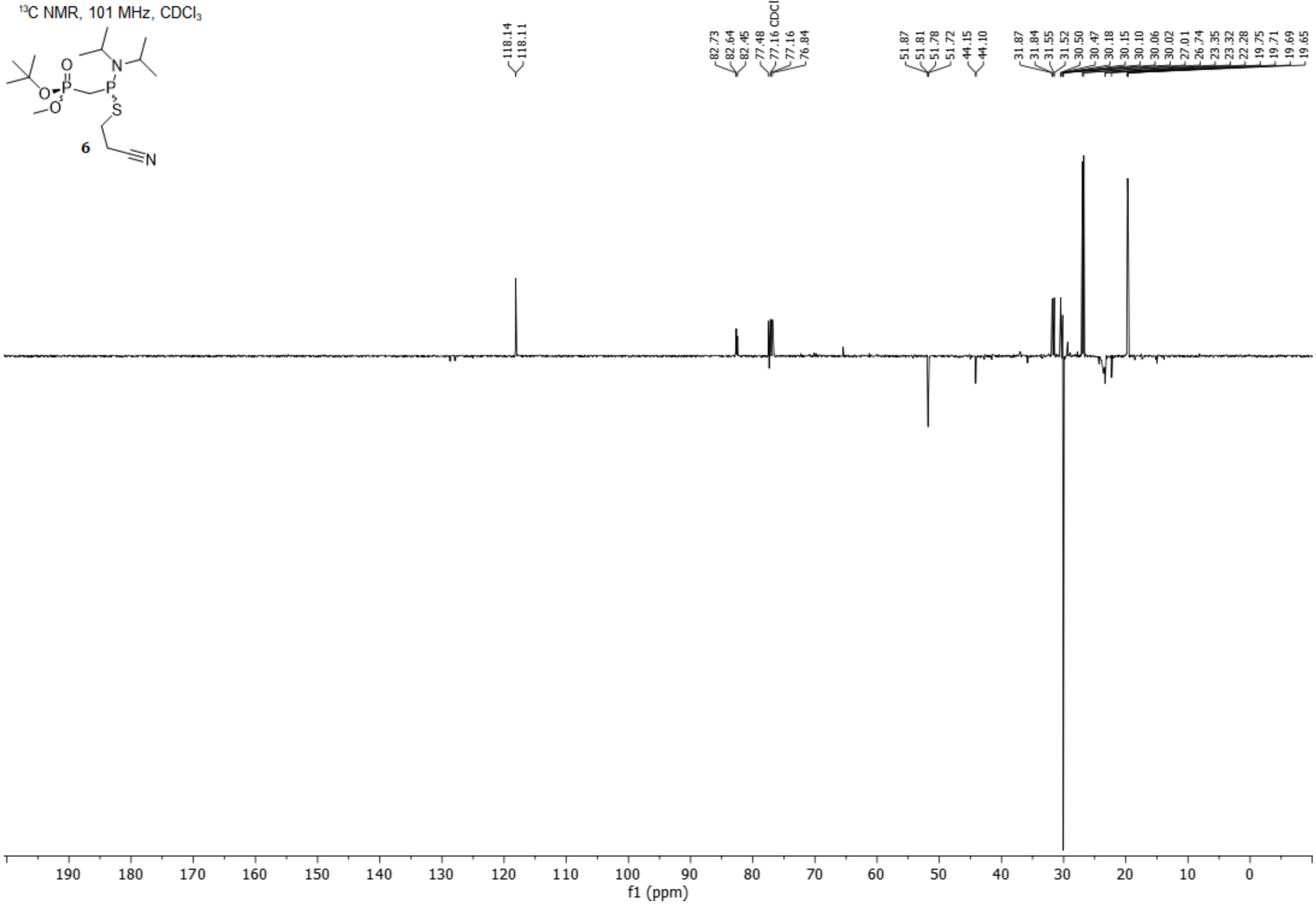

$^{31}P\{^1H\}$ NMR, 122 MHz,

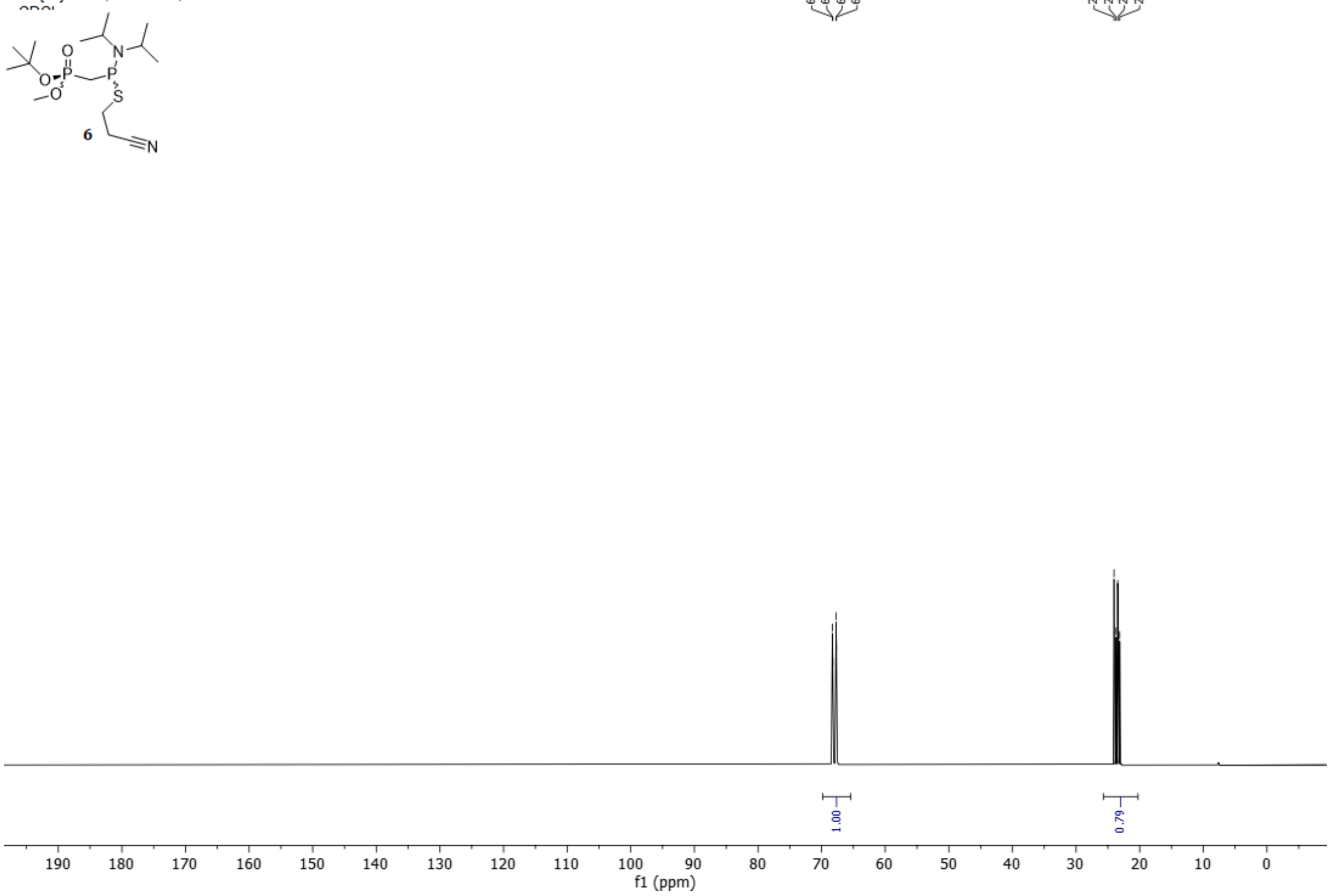

$^{31}P\{^{1}H\}$ NMR, 162 MHz, $CDCl_3$

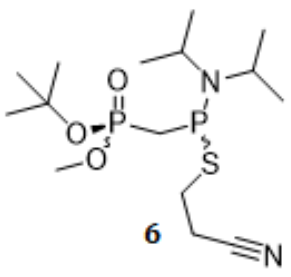

6

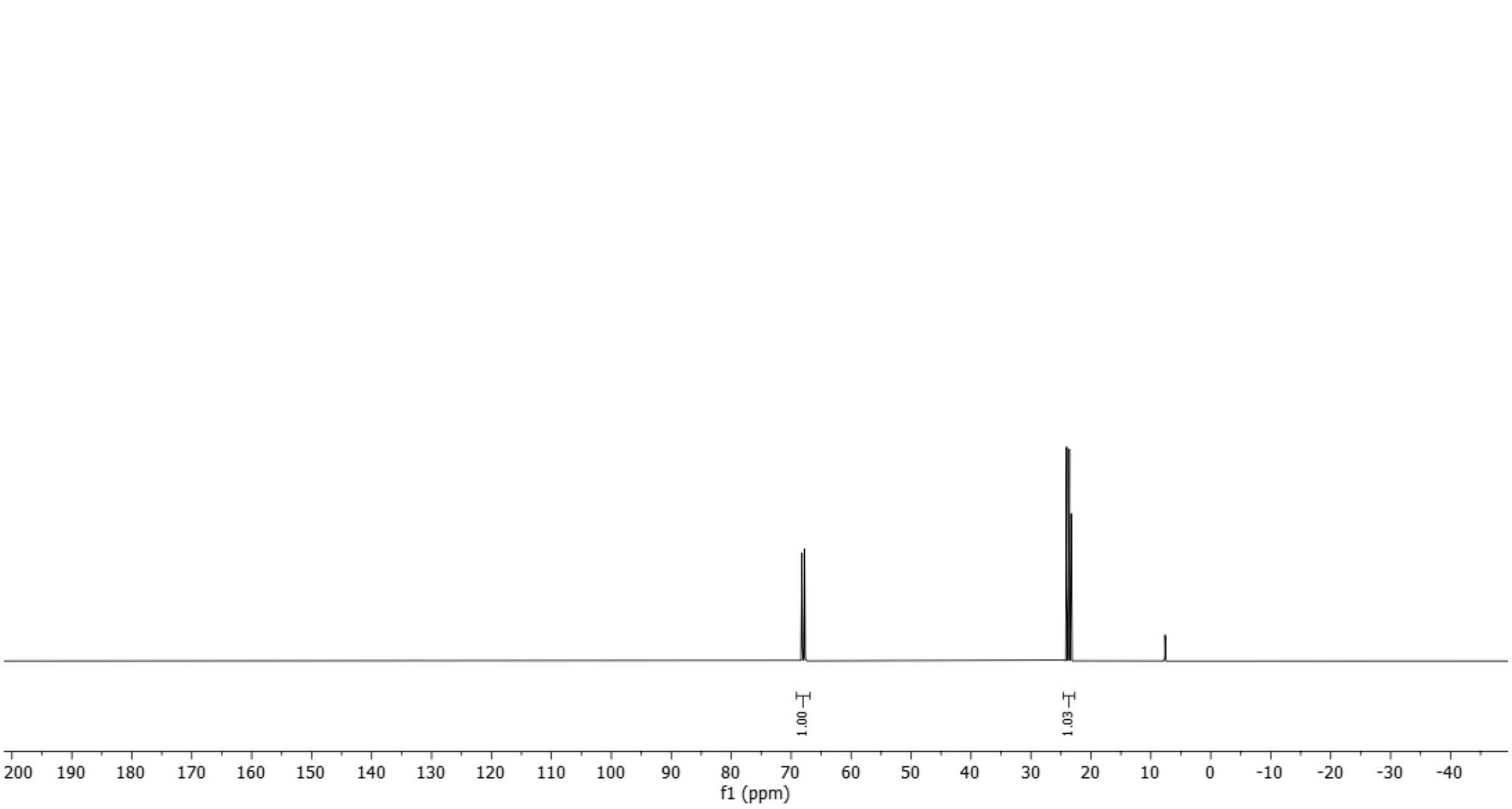

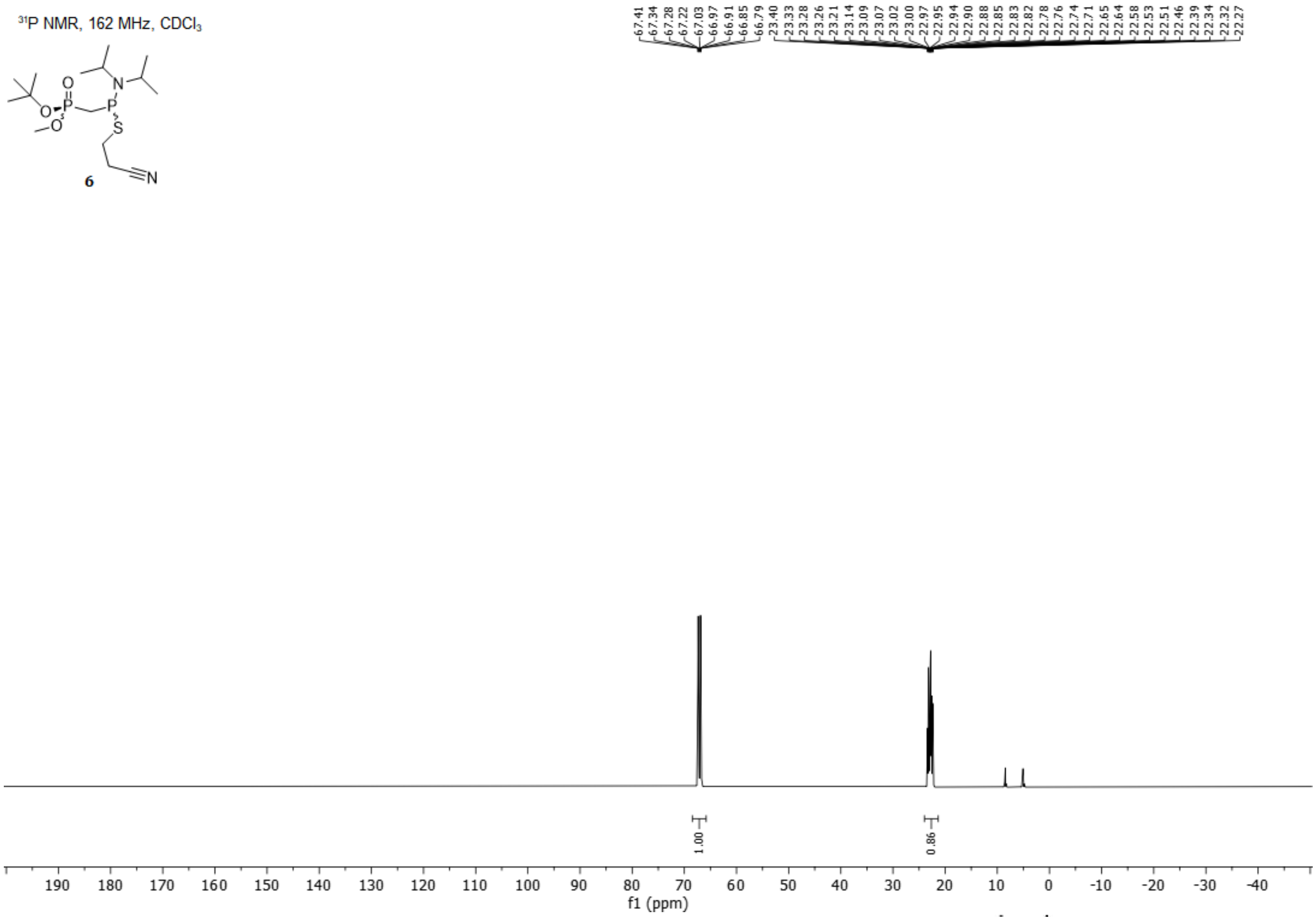

³¹P NMR, 162 MHz, CDCl₃
6
67.41
67.34
67.28
67.22
67.03
66.97
66.91
66.85
66.79
23.40
23.33
23.28
23.26
23.21
23.14
23.09
23.07
23.02
23.00
22.97
22.95
22.94
22.90
22.88
22.85
22.83
22.82
22.78
22.76
22.74
22.71
22.65
22.64
22.58
22.53
22.51
22.46
22.39
22.34
22.32
22.27
1.00
0.86
190 180 170 160 150 140 130 120 110 100 90 80 70 60 50 40 30 20 10 0 -10 -20 -30 -40
f1 (ppm)

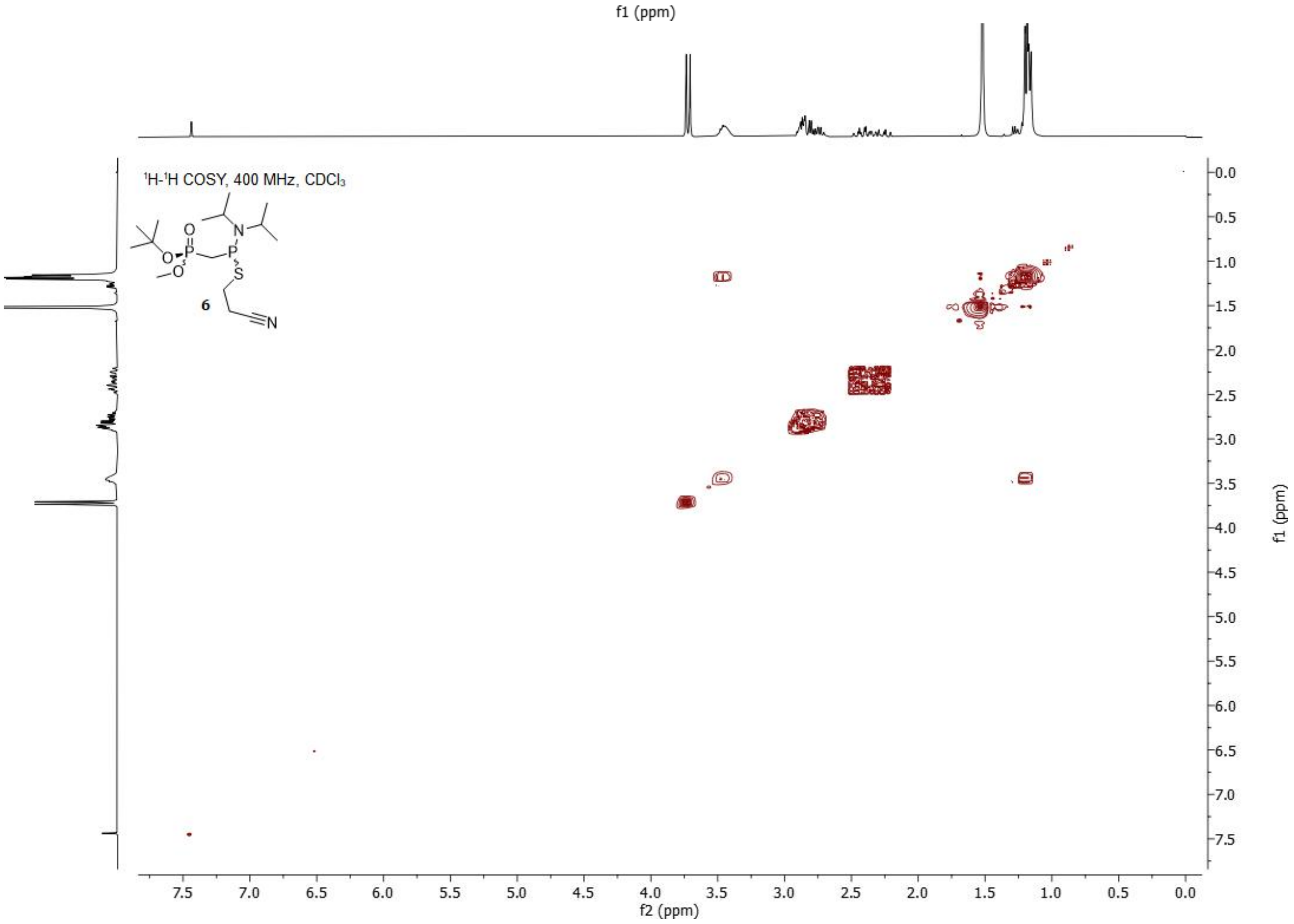

¹H-¹H COSY, 400 MHz, CDCl₃
6
7.5 7.0 6.5 6.0 5.5 5.0 4.5 4.0 3.5 3.0 2.5 2.0 1.5 1.0 0.5 0.0
f2 (ppm)
0.0 0.5 1.0 1.5 2.0 2.5 3.0 3.5 4.0 4.5 5.0 5.5 6.0 6.5 7.0 7.5
f1 (ppm)

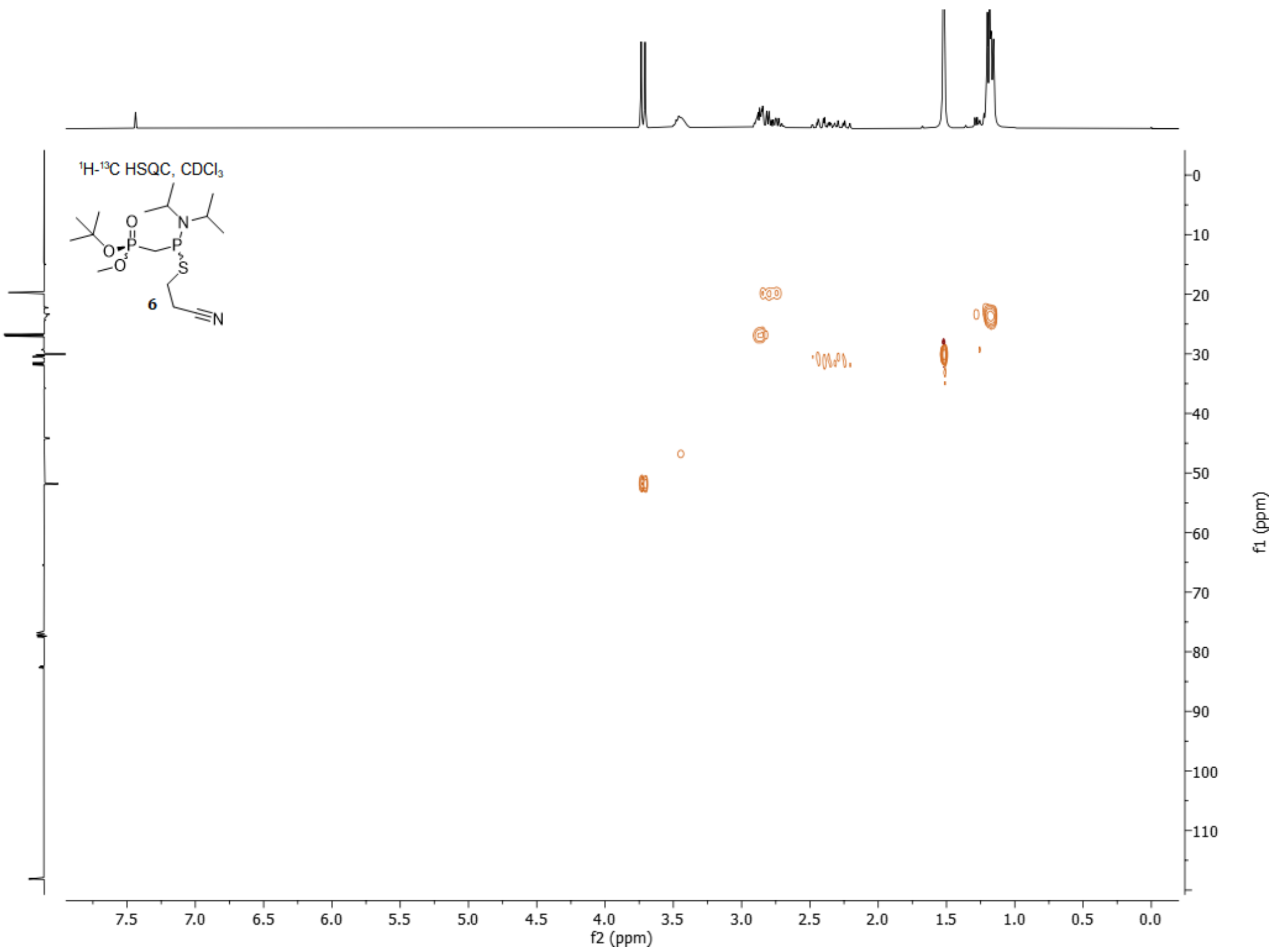

^1H-^{13}C HSQC, $CDCl_3$
6
f2 (ppm)
f1 (ppm)

NMR Spectra of Phosphonylmethyl Phosphono(di)thioate Monoesters and Precursors

me-ADP-αS tetraester 12a

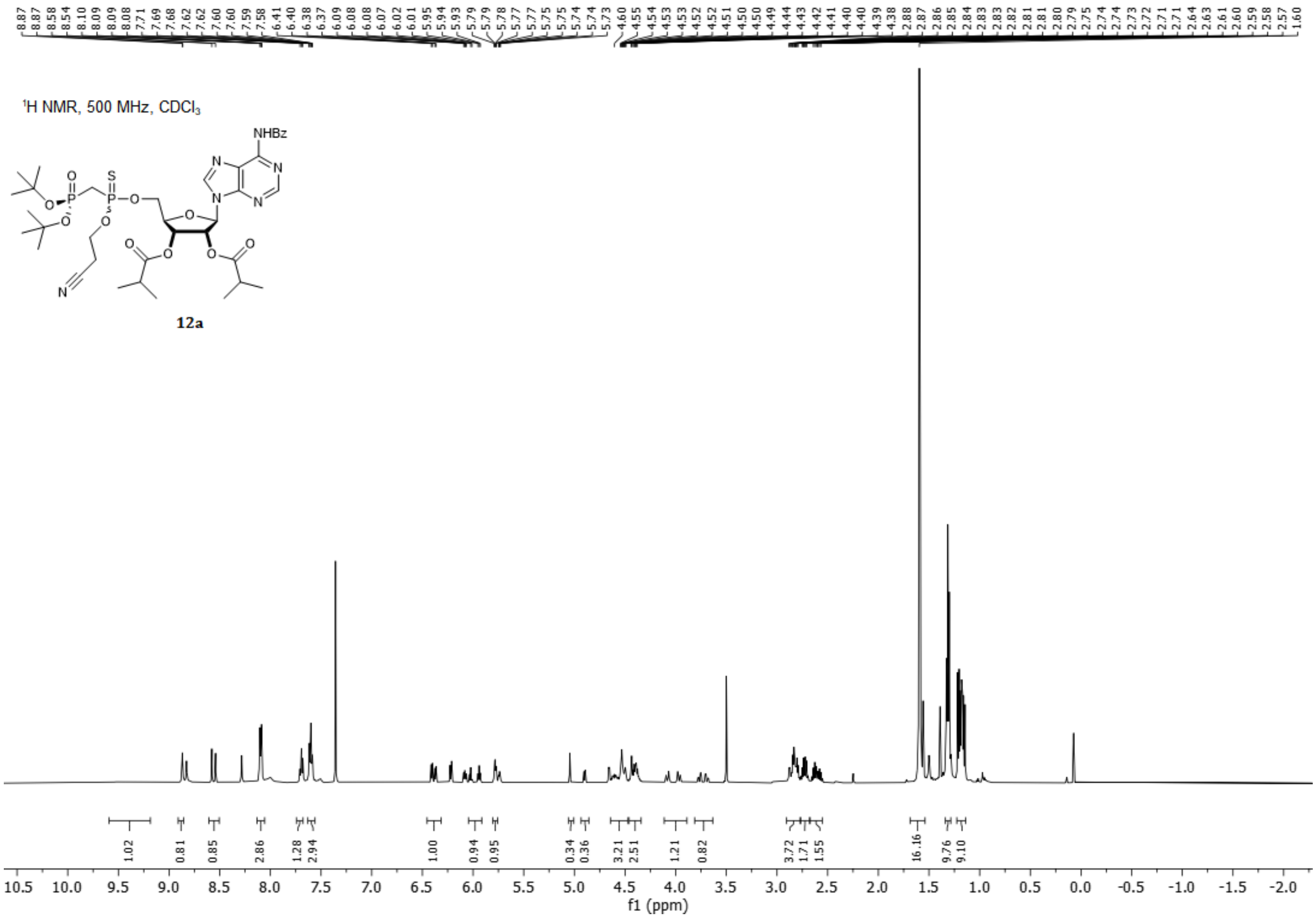

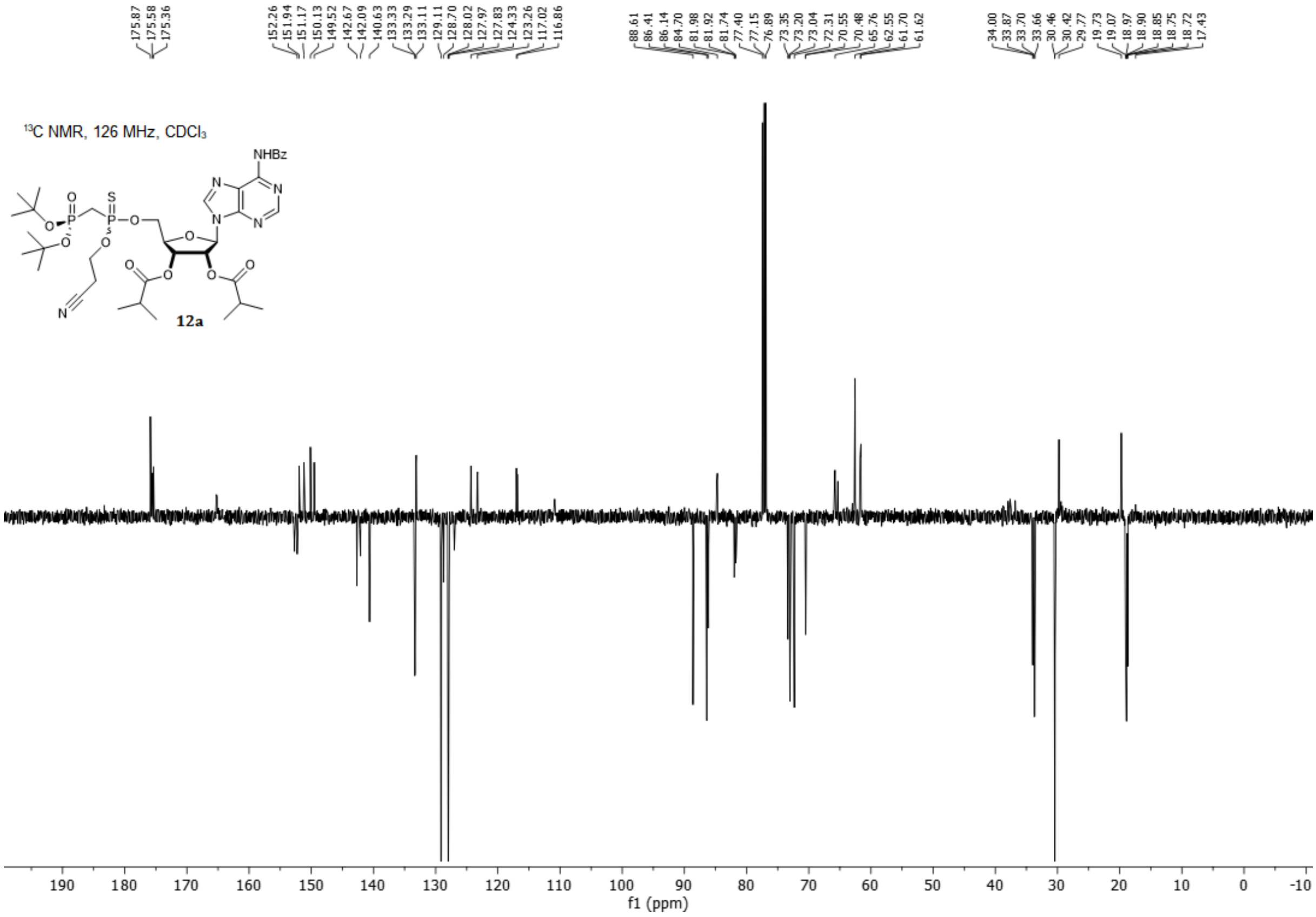

$^{31}P\{^{1}H\}$ NMR, 202 MHz, $CDCl_3$

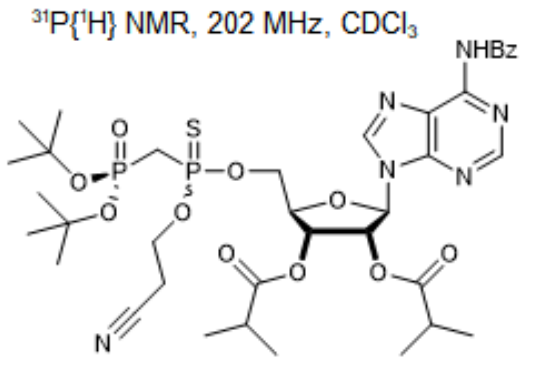

12a

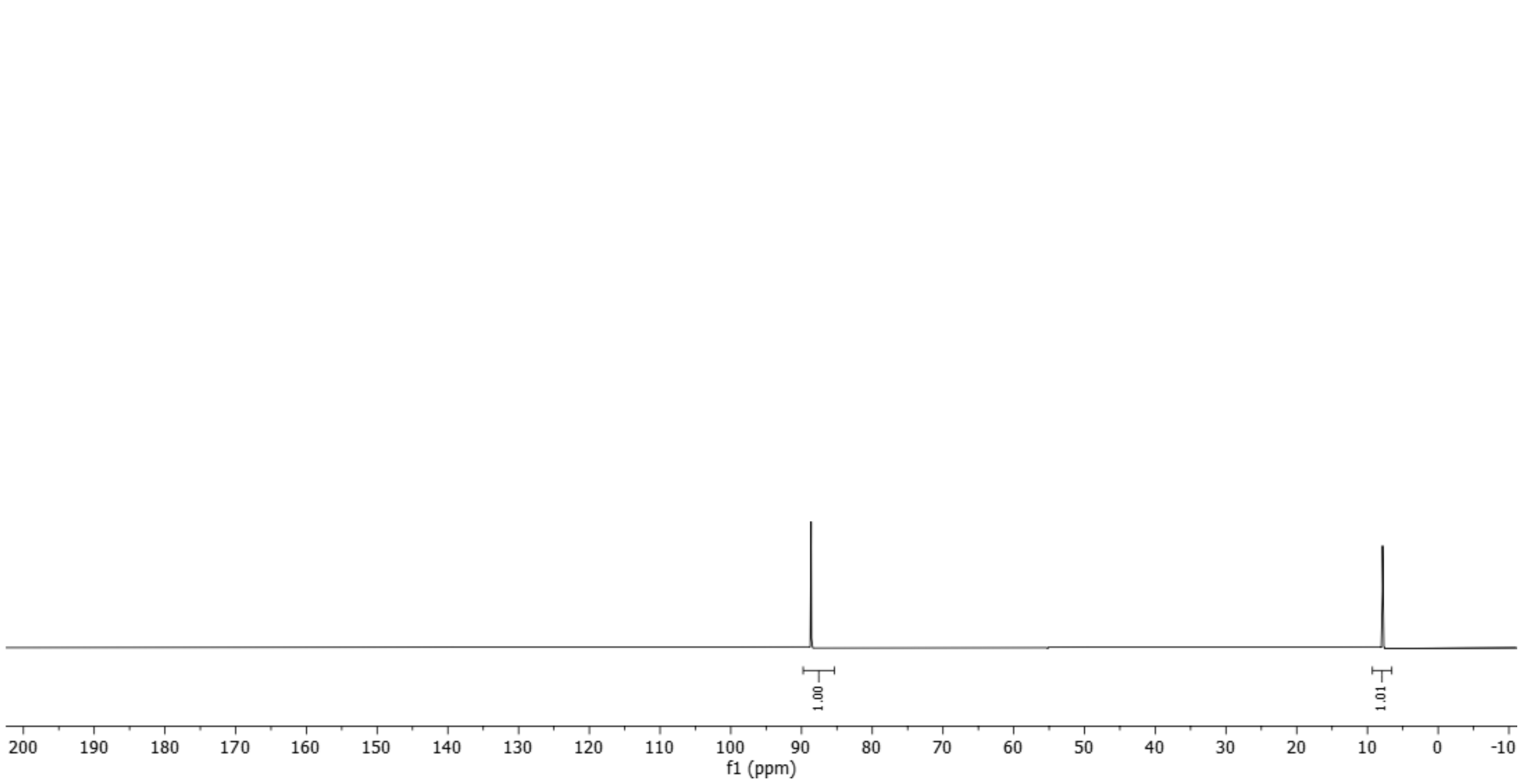

^{31}P NMR, 202 MHz, $CDCl_3$

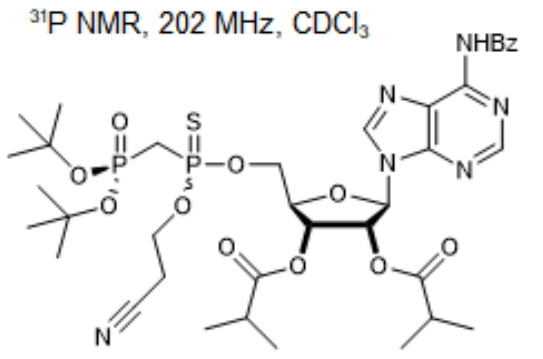

12a

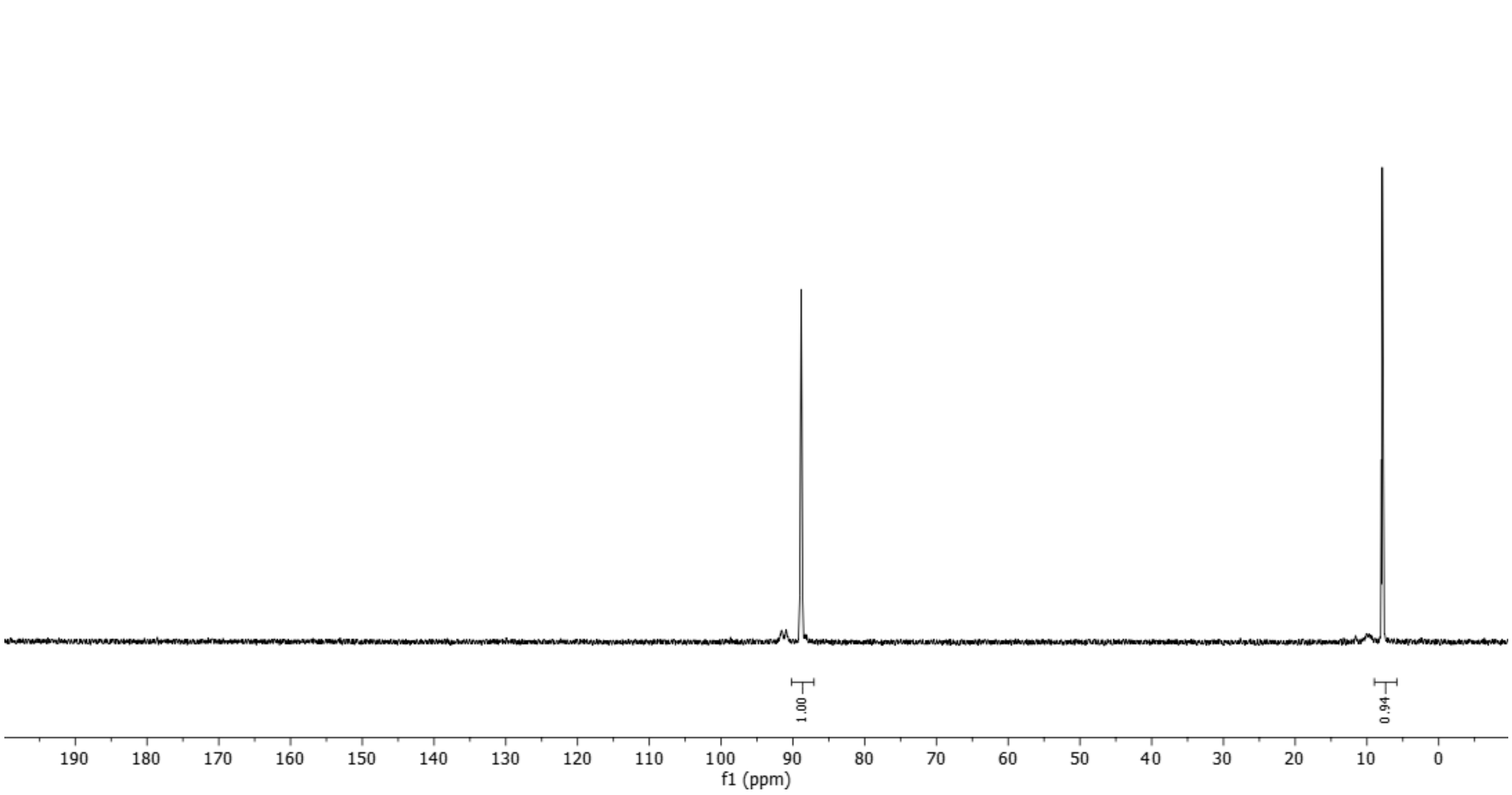

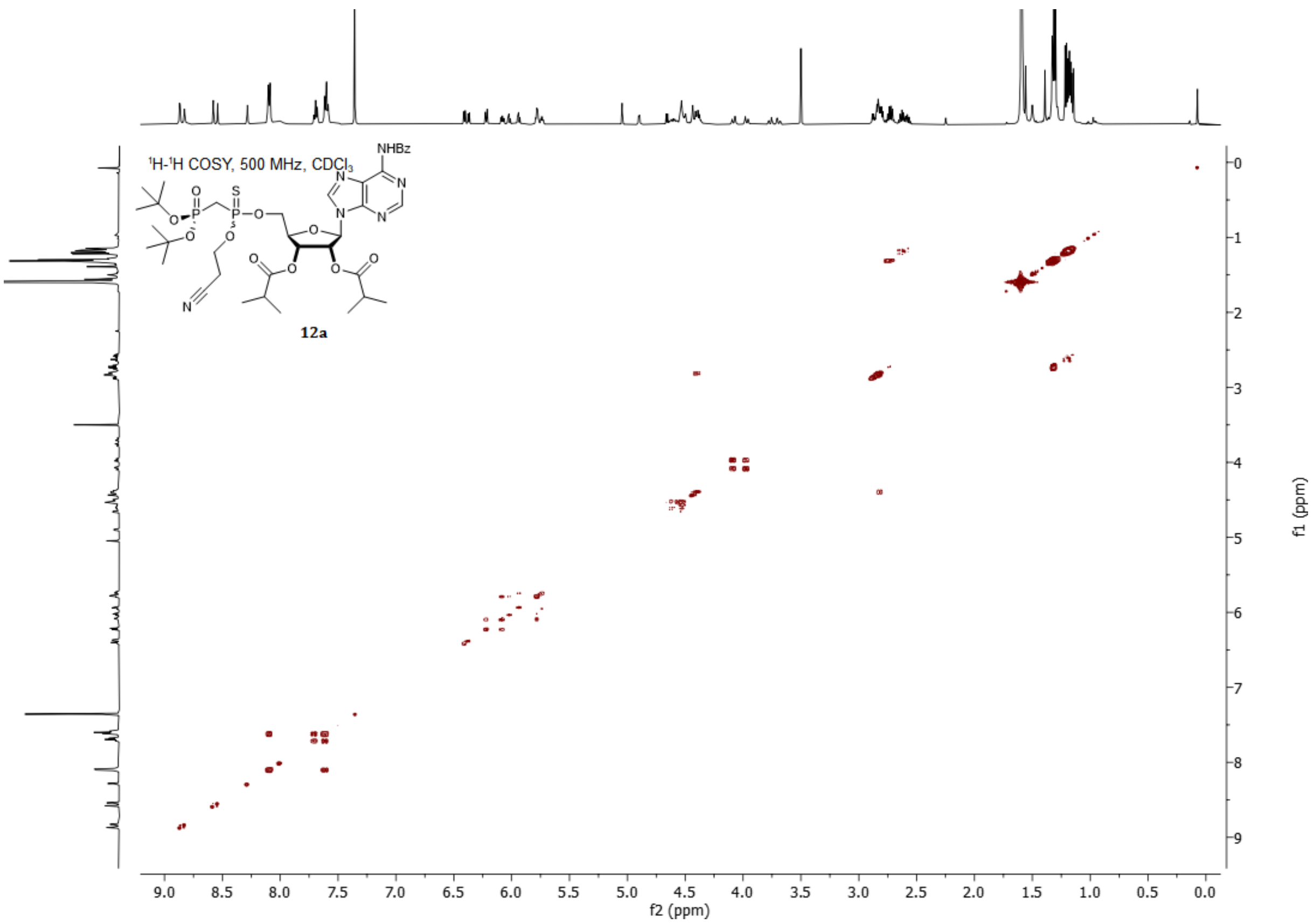

1H-1H COSY, 500 MHz, CDCl3
NHBz
12a
f2 (ppm)
f1 (ppm)

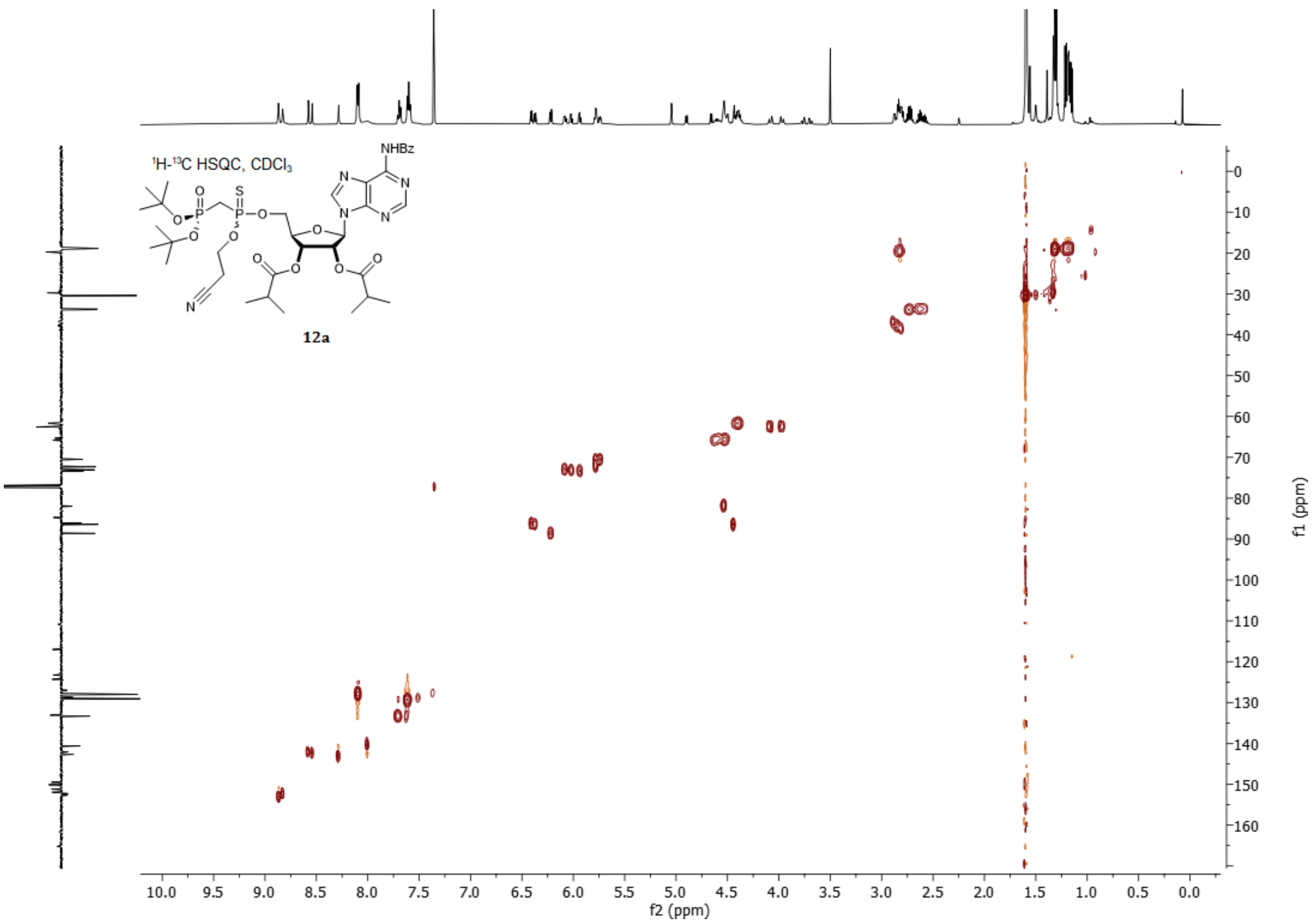

1H-13C HSQC, CDCl3
NHBz
12a
f2 (ppm)
f1 (ppm)

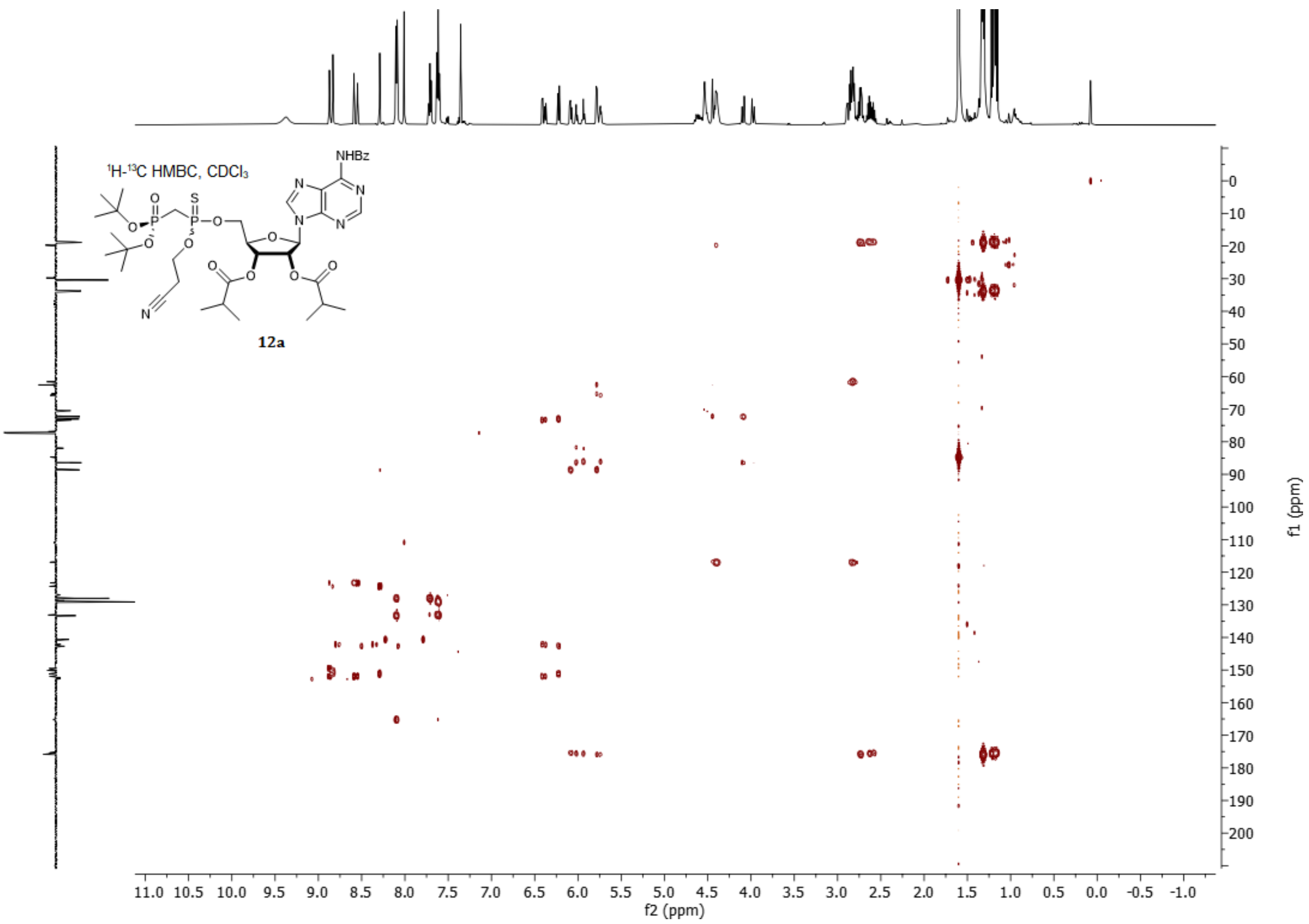

me-ADP-αS$_2$ tetraester 12b

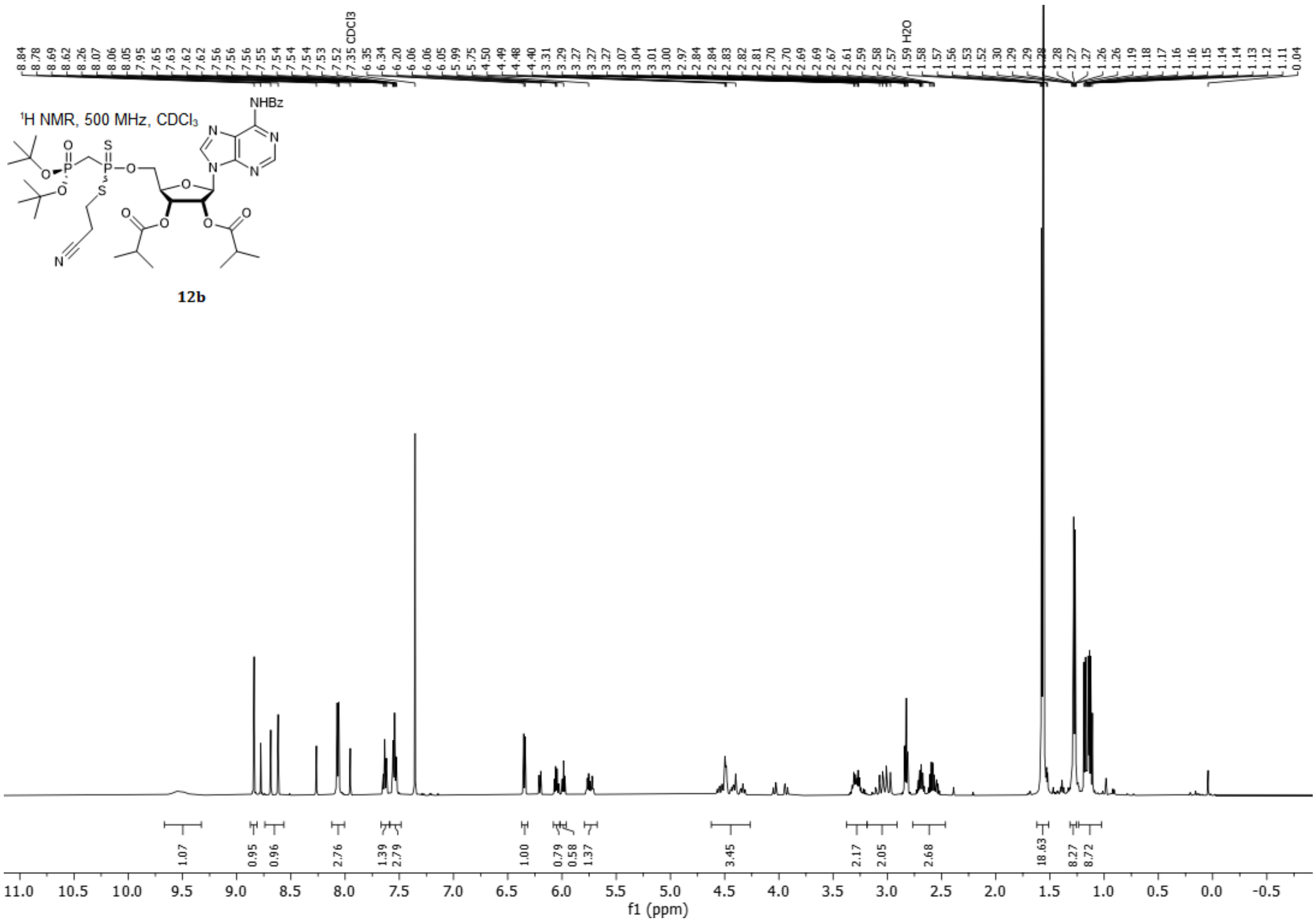
8.84 8.78 8.69 8.62 8.26 8.07 8.06 8.05 7.95 7.65 7.63 7.62 7.62 7.56 7.56 7.56 7.55 7.54 7.54 7.54 7.53 7.52 7.35 CDCl3 6.35 6.34 6.20 6.06 6.06 6.05 5.99 5.75 4.50 4.49 4.48 4.40 3.31 3.29 3.27 3.27 3.27 3.07 3.04 3.01 3.00 2.97 2.84 2.84 2.83 2.82 2.81 2.70 2.70 2.69 2.69 2.67 2.61 2.59 2.58 2.57 1.59 H2O 1.58 1.57 1.56 1.53 1.52 1.30 1.29 1.29 1.28 1.28 1.27 1.27 1.26 1.26 1.19 1.18 1.17 1.16 1.16 1.15 1.14 1.14 1.13 1.12 1.11 0.04
1H NMR, 500 MHz, CDCl3
NHBz
12b
1.07 0.95 0.96 2.76 1.39 2.79 1.00 0.79 0.58 1.37 3.45 2.17 2.05 2.68 18.63 8.27 8.72
11.0 10.5 10.0 9.5 9.0 8.5 8.0 7.5 7.0 6.5 6.0 5.5 5.0 4.5 4.0 3.5 3.0 2.5 2.0 1.5 1.0 0.5 0.0 -0.5
f1 (ppm)

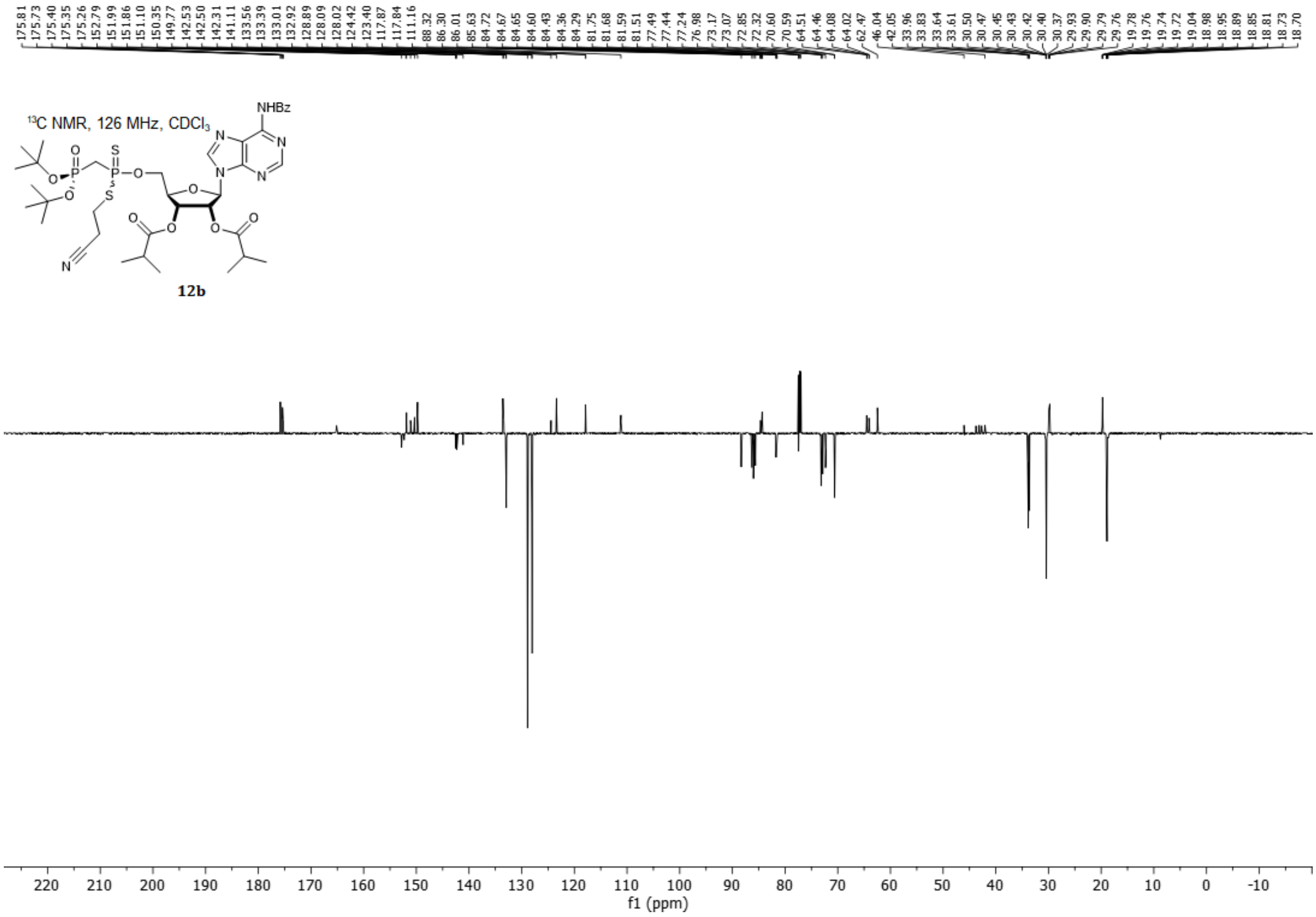
175.81 175.73 175.40 175.35 175.26 152.79 151.99 151.86 151.10 150.35 149.77 142.53 142.50 142.31 141.11 133.56 133.39 133.01 132.92 128.89 128.09 128.02 124.42 123.40 117.87 117.84 111.16 88.32 86.30 86.01 85.63 84.72 84.67 84.65 84.60 84.43 84.36 84.29 81.75 81.68 81.59 81.51 77.49 77.44 77.24 76.98 73.17 73.07 72.85 72.32 70.60 70.59 64.51 64.46 64.08 64.02 62.47 46.04 42.05 33.96 33.83 33.64 33.61 30.50 30.47 30.45 30.43 30.42 30.40 30.37 29.93 29.90 29.79 29.76 19.78 19.76 19.74 19.72 19.04 18.98 18.95 18.89 18.85 18.81 18.73 18.70
13C NMR, 126 MHz, CDCl3
NHBz
12b
220 210 200 190 180 170 160 150 140 130 120 110 100 90 80 70 60 50 40 30 20 10 0 -10
f1 (ppm)

³¹P{¹H} NMR, 202 MHz, $CDCl_3$

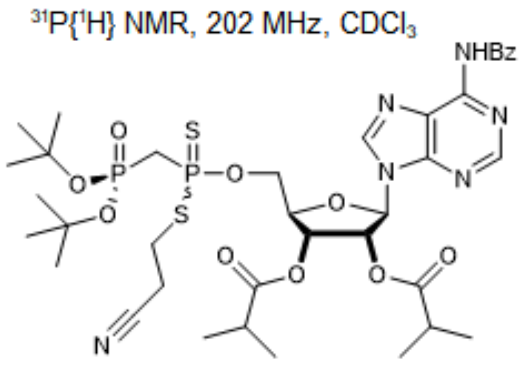

12b

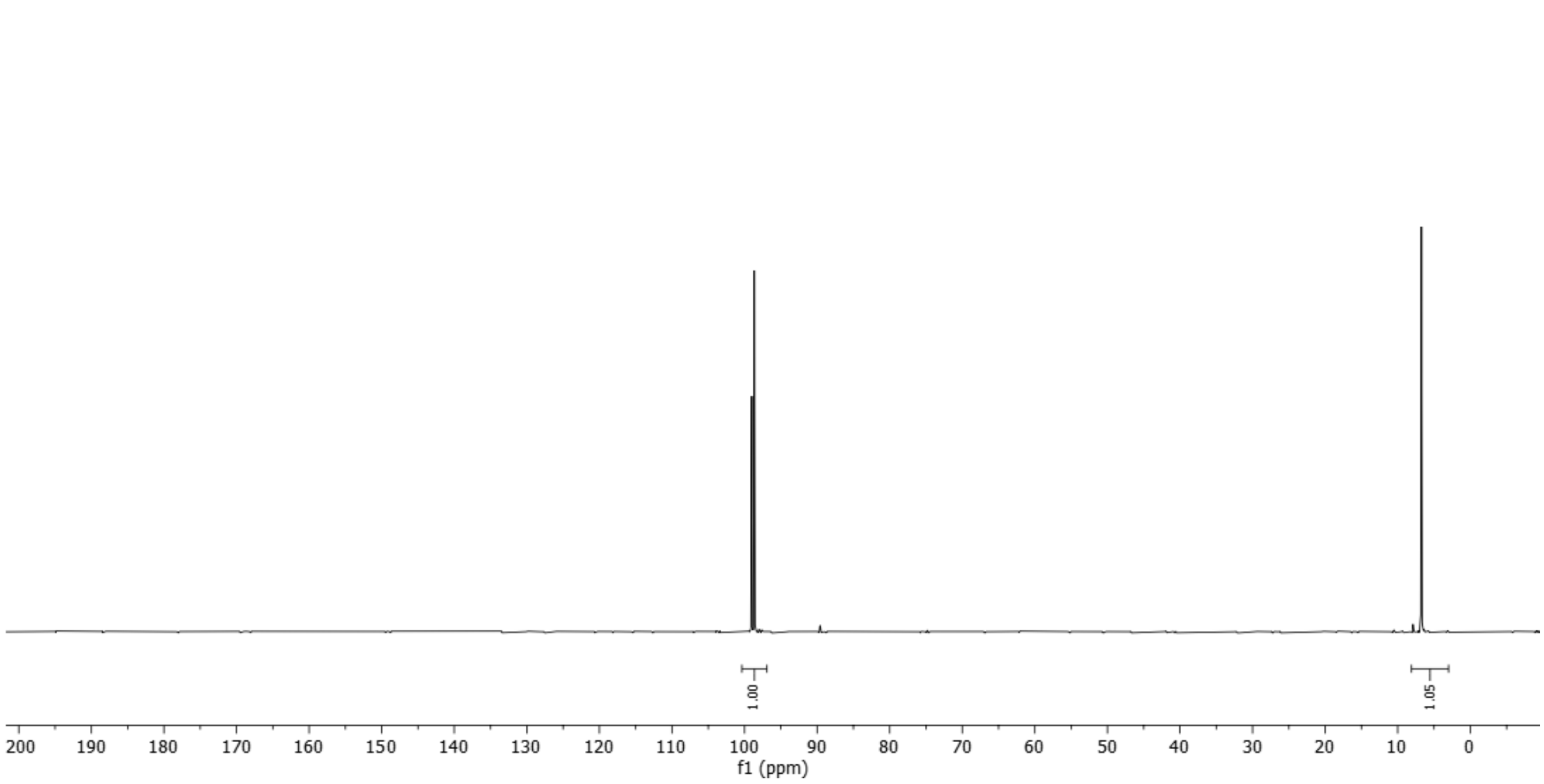

³¹P NMR, 202 MHz, $CDCl_3$

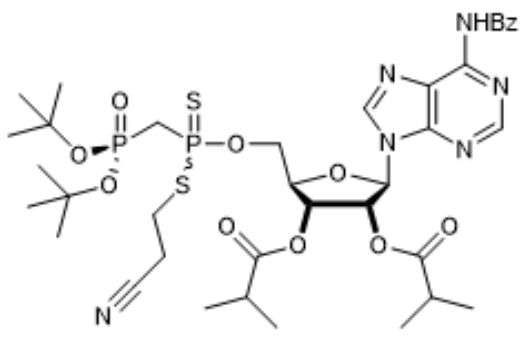

12b

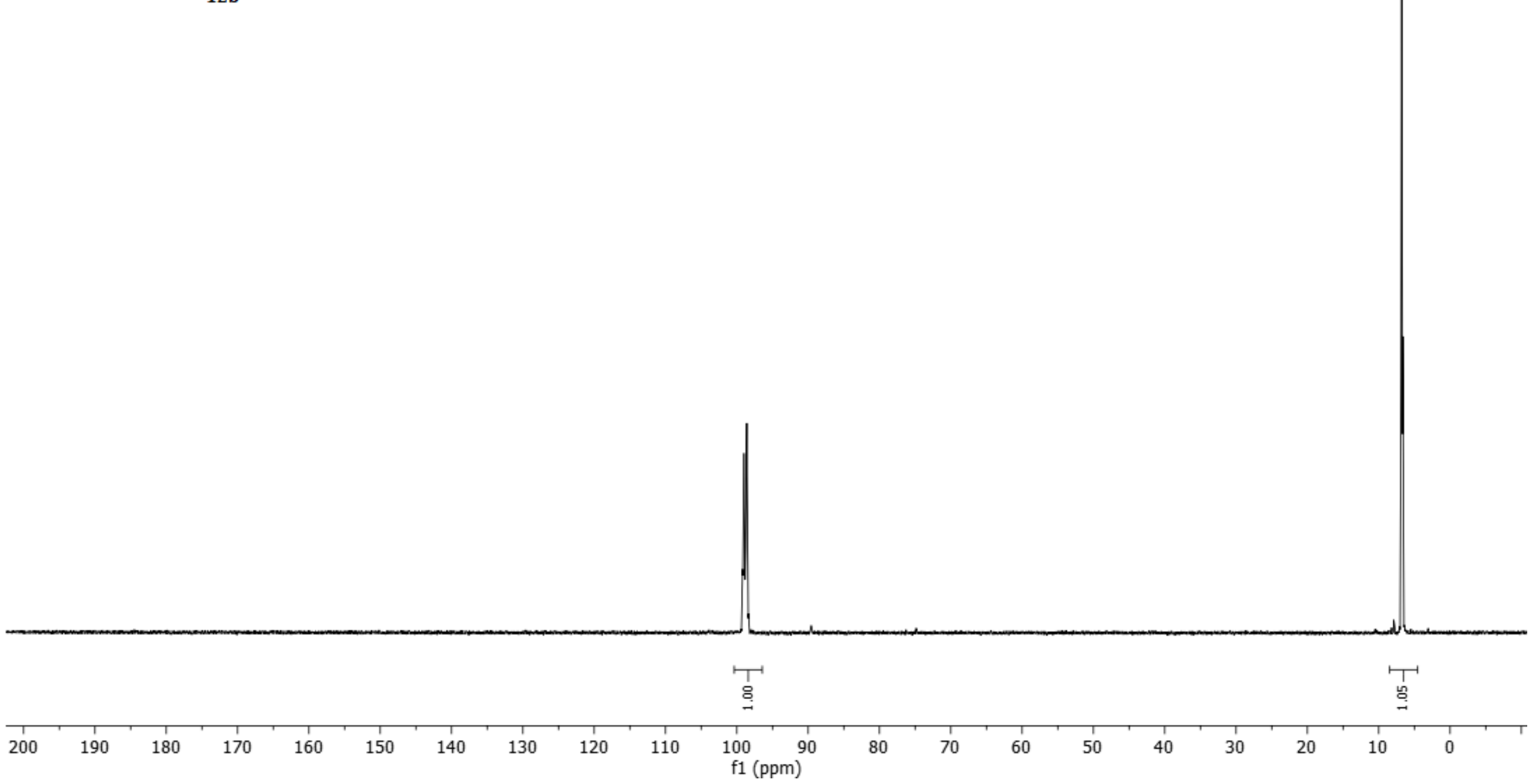

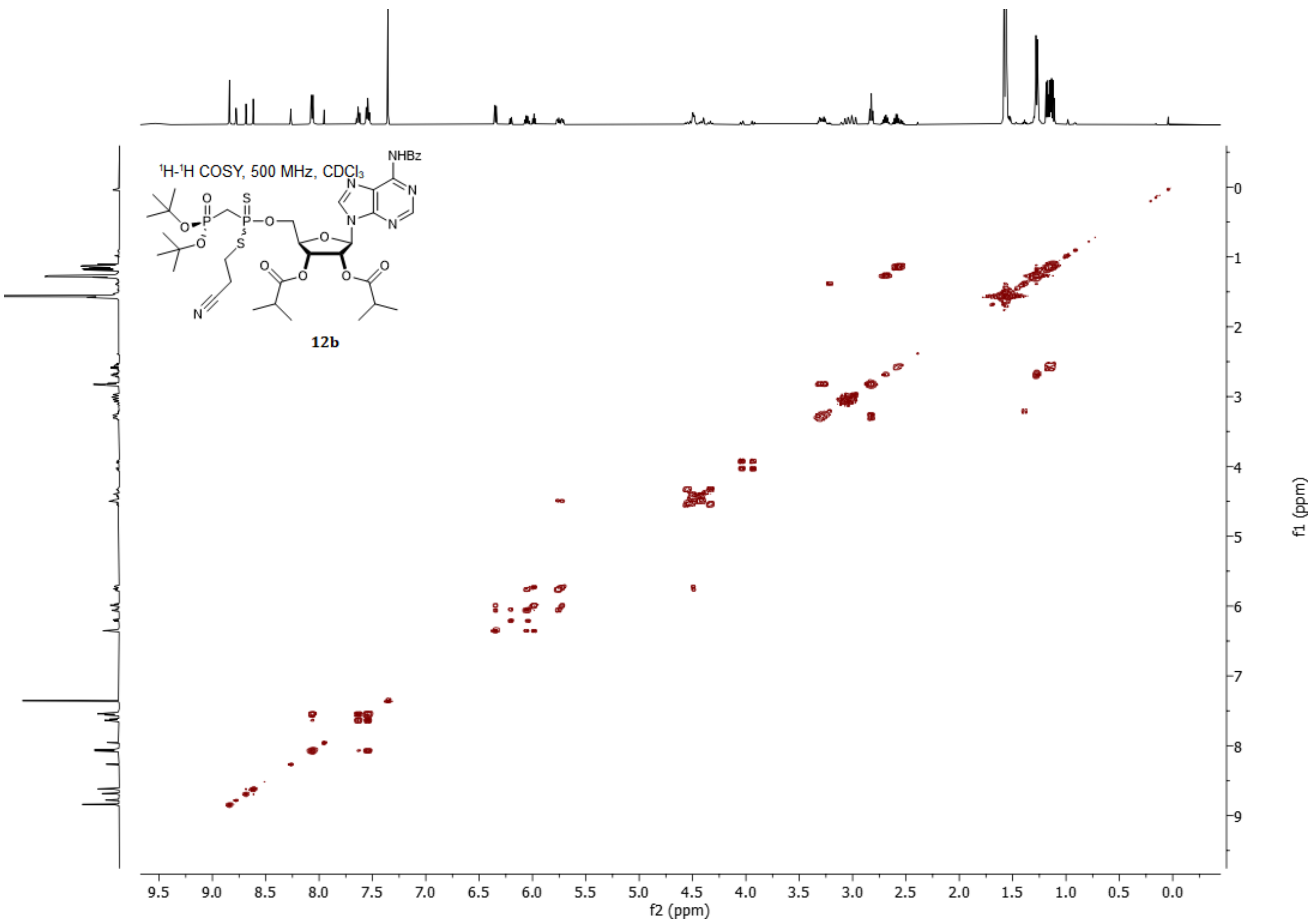

^{1}H-^{1}H COSY, 500 MHz, CDCl$_3$
12b
f2 (ppm)
f1 (ppm)

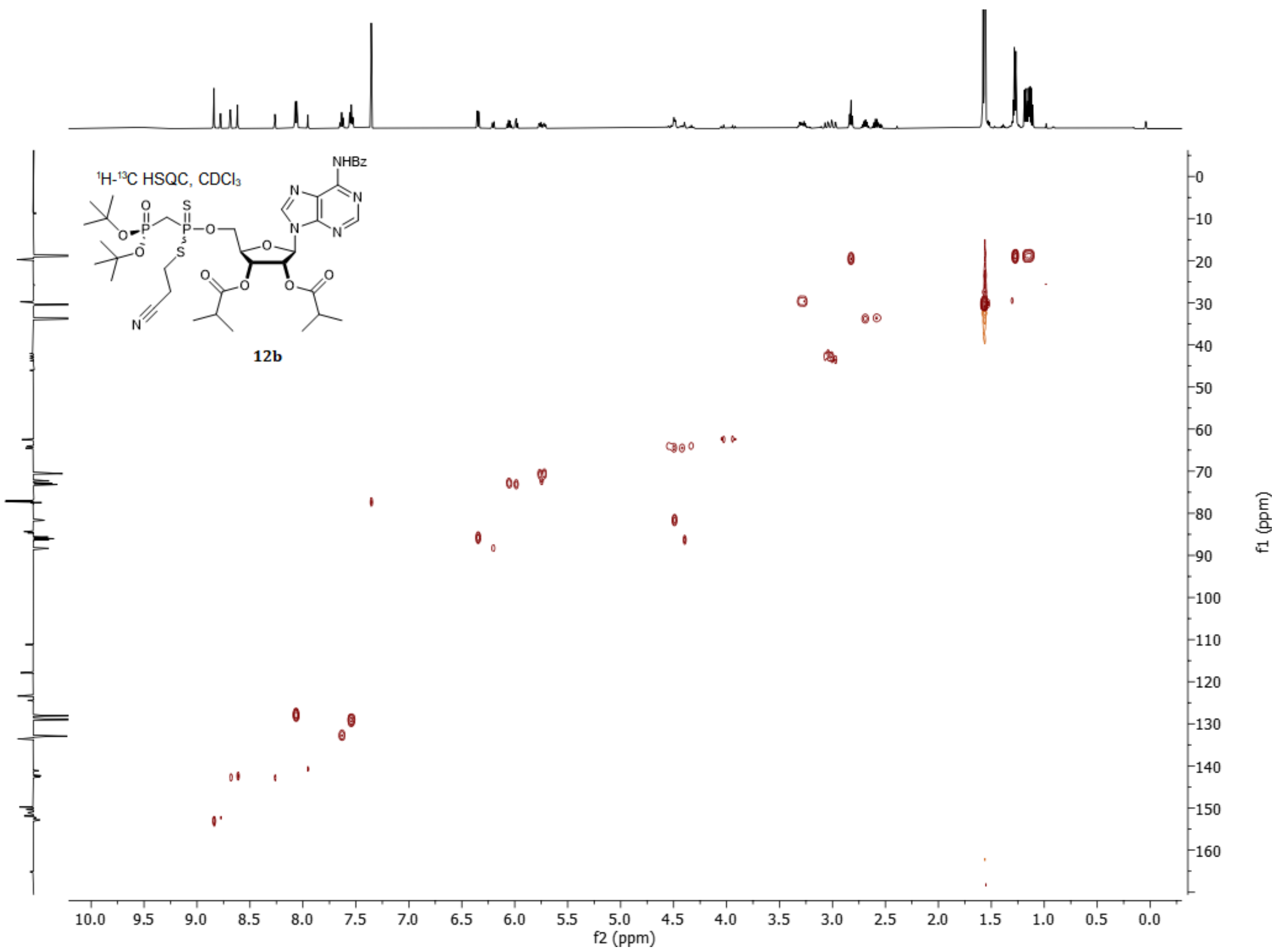

^{1}H-^{13}C HSQC, CDCl$_3$
12b
f2 (ppm)
f1 (ppm)

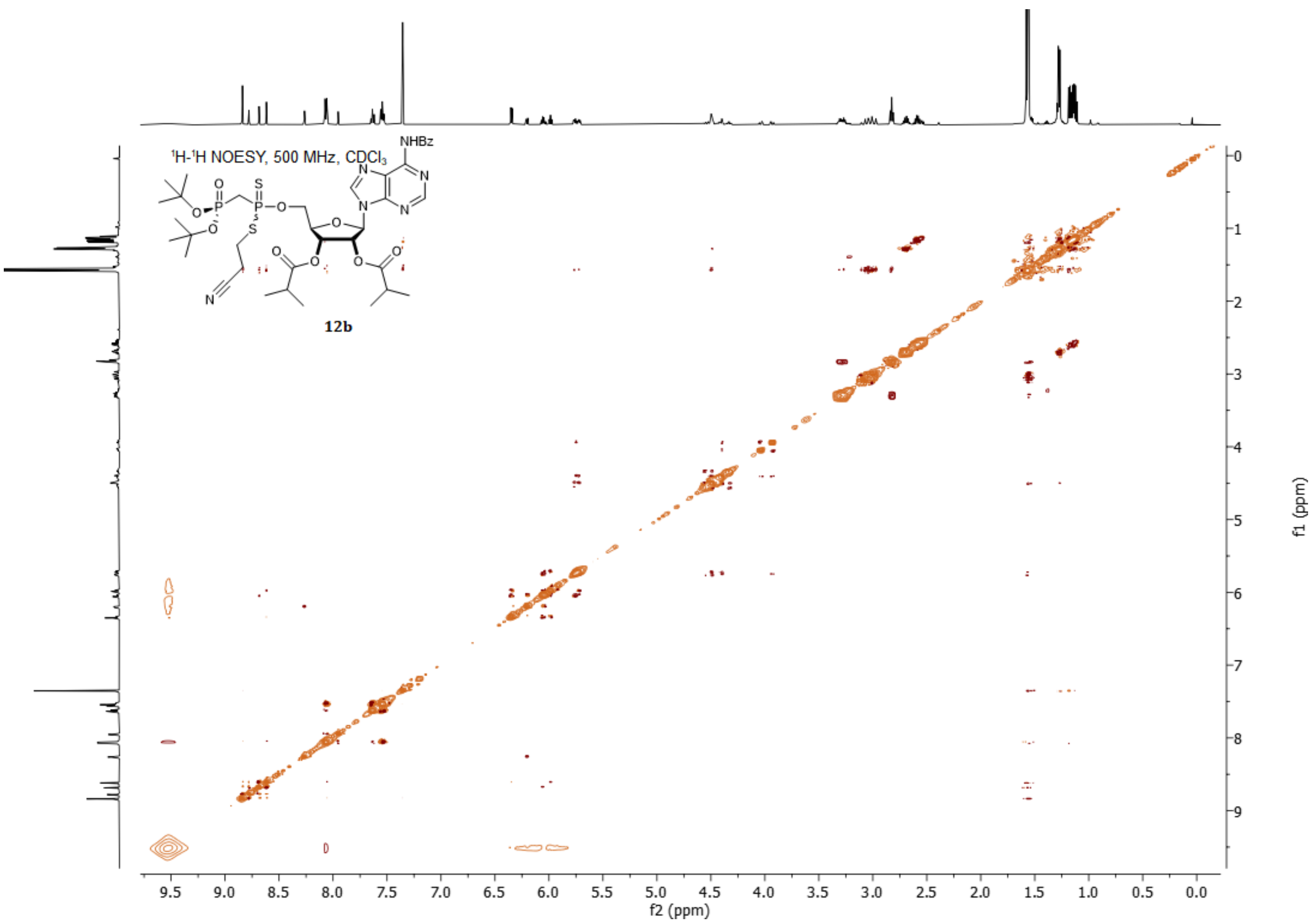

^{1}H-^{1}H NOESY, 500 MHz, CDCl$_3$
NHBz
12b
f2 (ppm)
f1 (ppm)

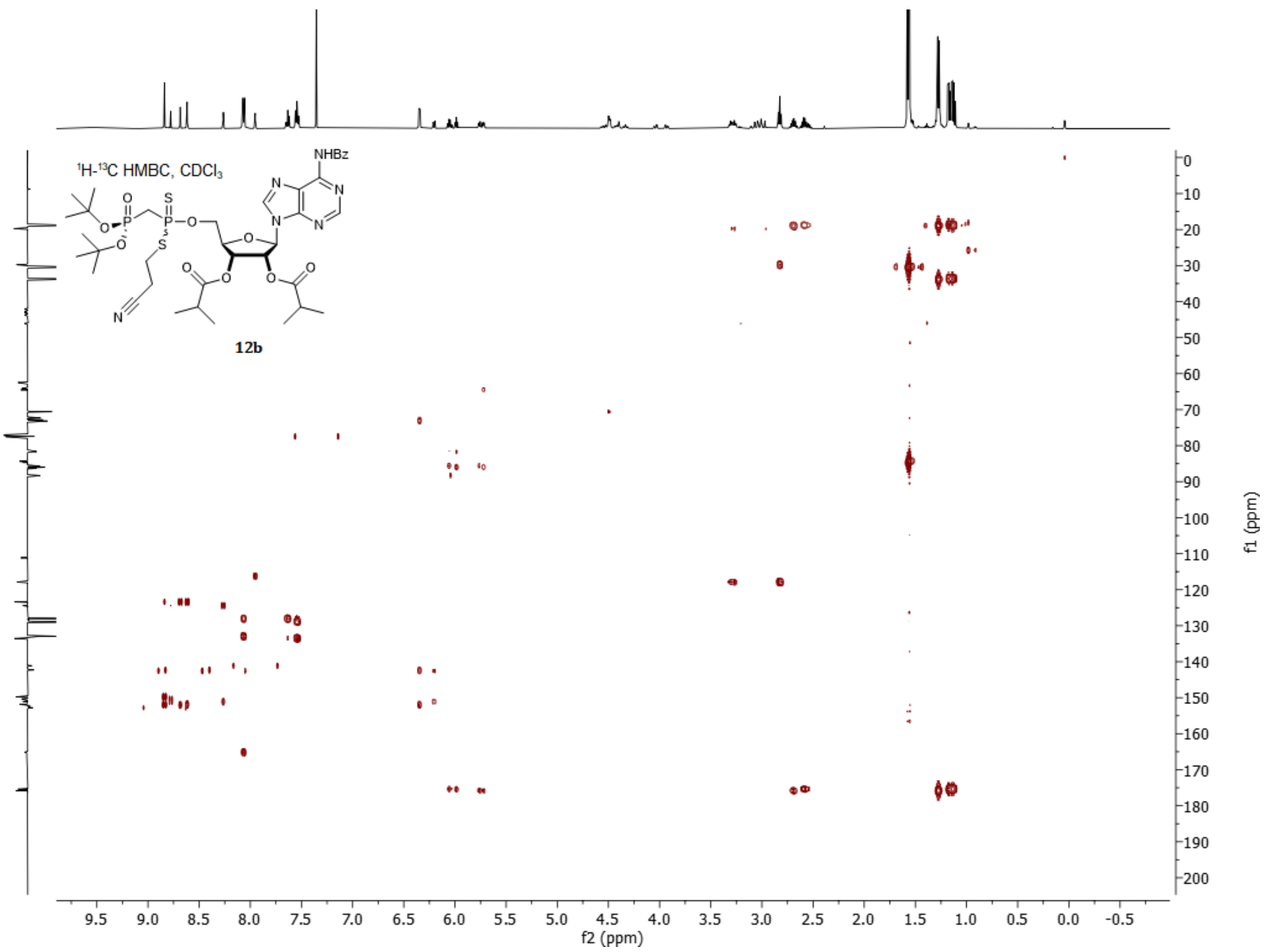

^{1}H-^{13}C HMBC, CDCl$_3$
NHBz
12b
f2 (ppm)
f1 (ppm)

me-ADP-αS 13a

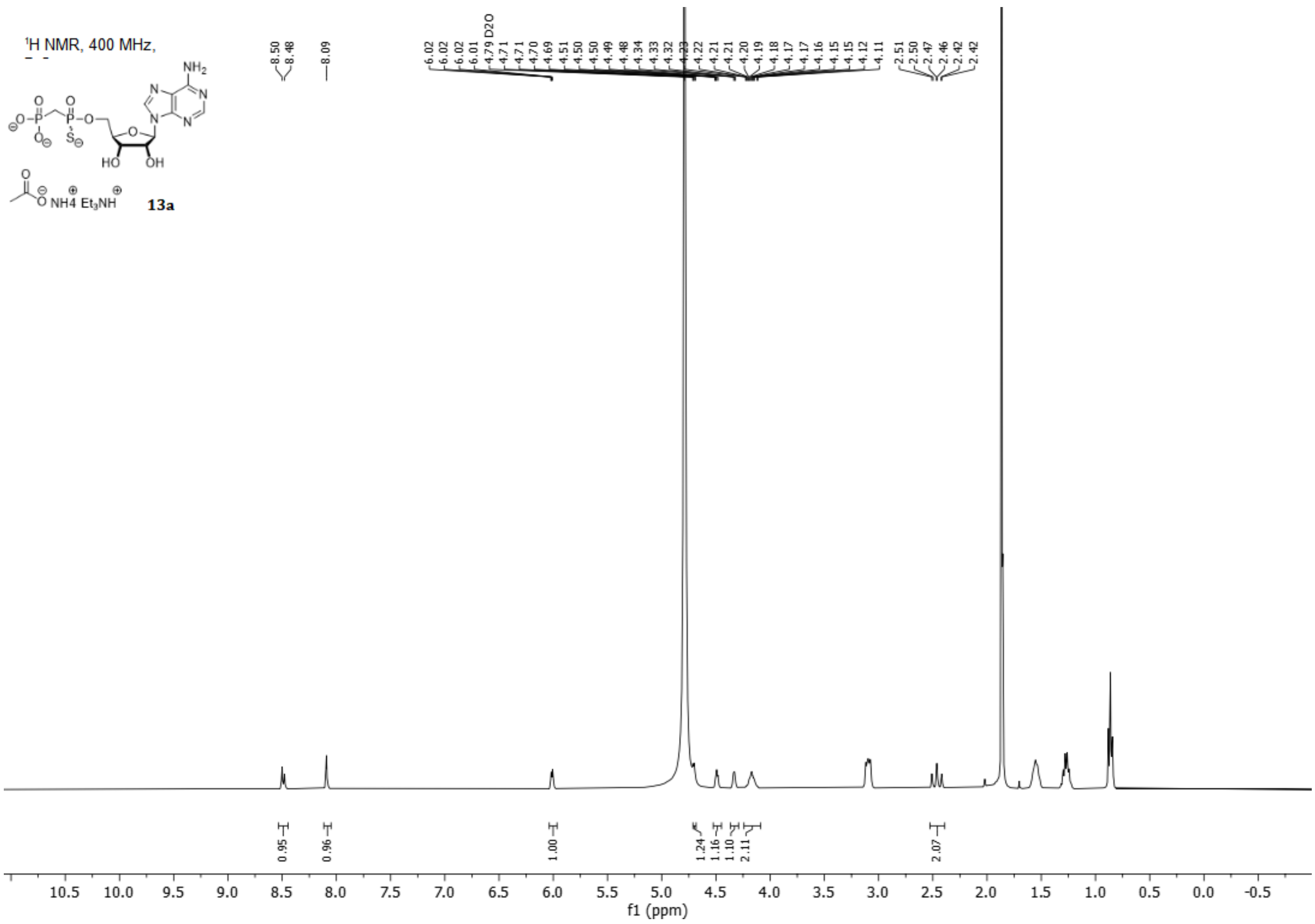

¹H NMR, 400 MHz,
NH2
HO
OH
NH4 Et3NH
13a
4.79 D2O
f1 (ppm)

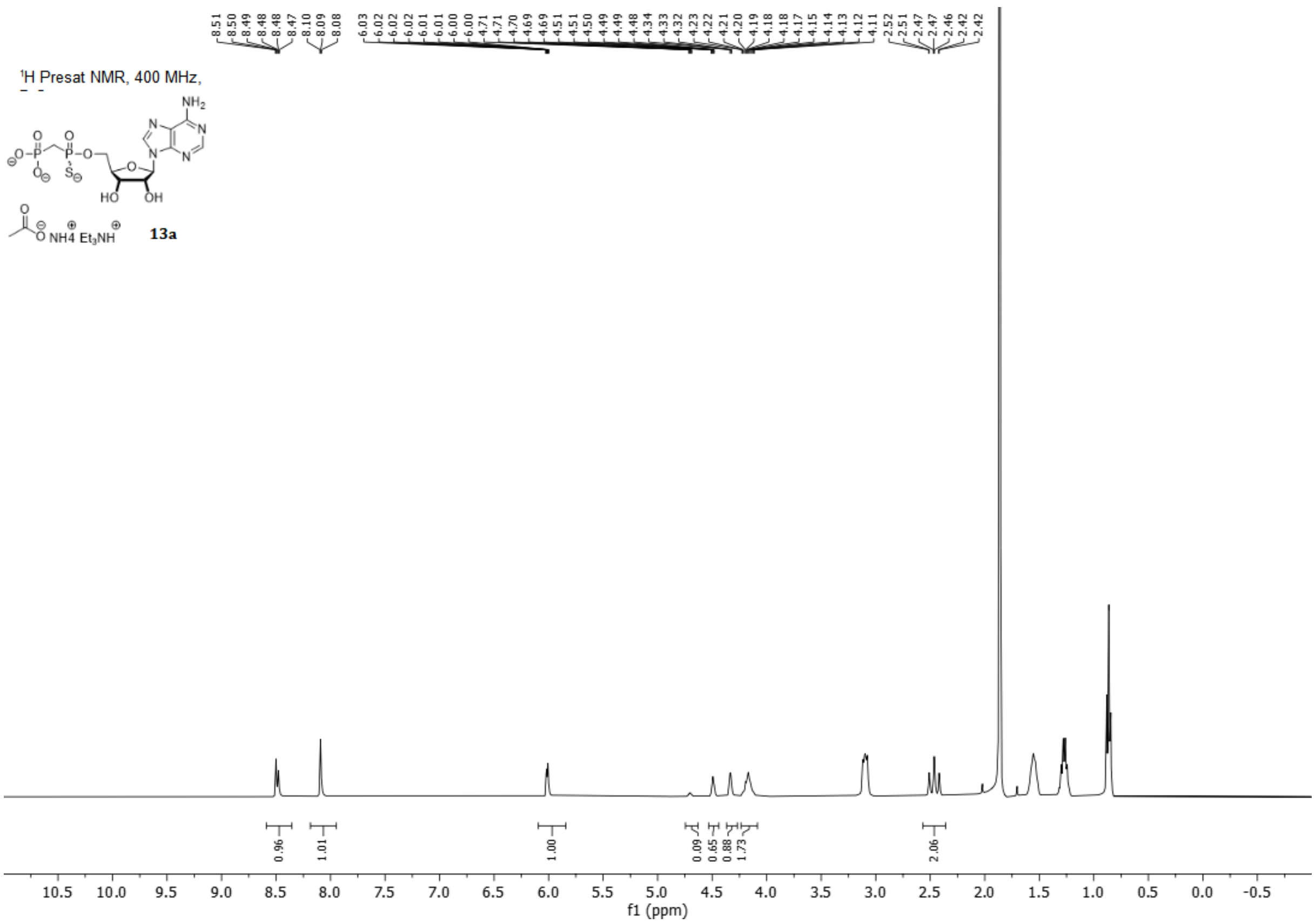

¹H Presat NMR, 400 MHz,
NH2
HO
OH
NH4 Et3NH
13a
f1 (ppm)

^{13}C NMR, 101 MHz,

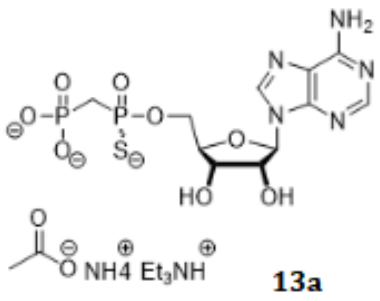

13a

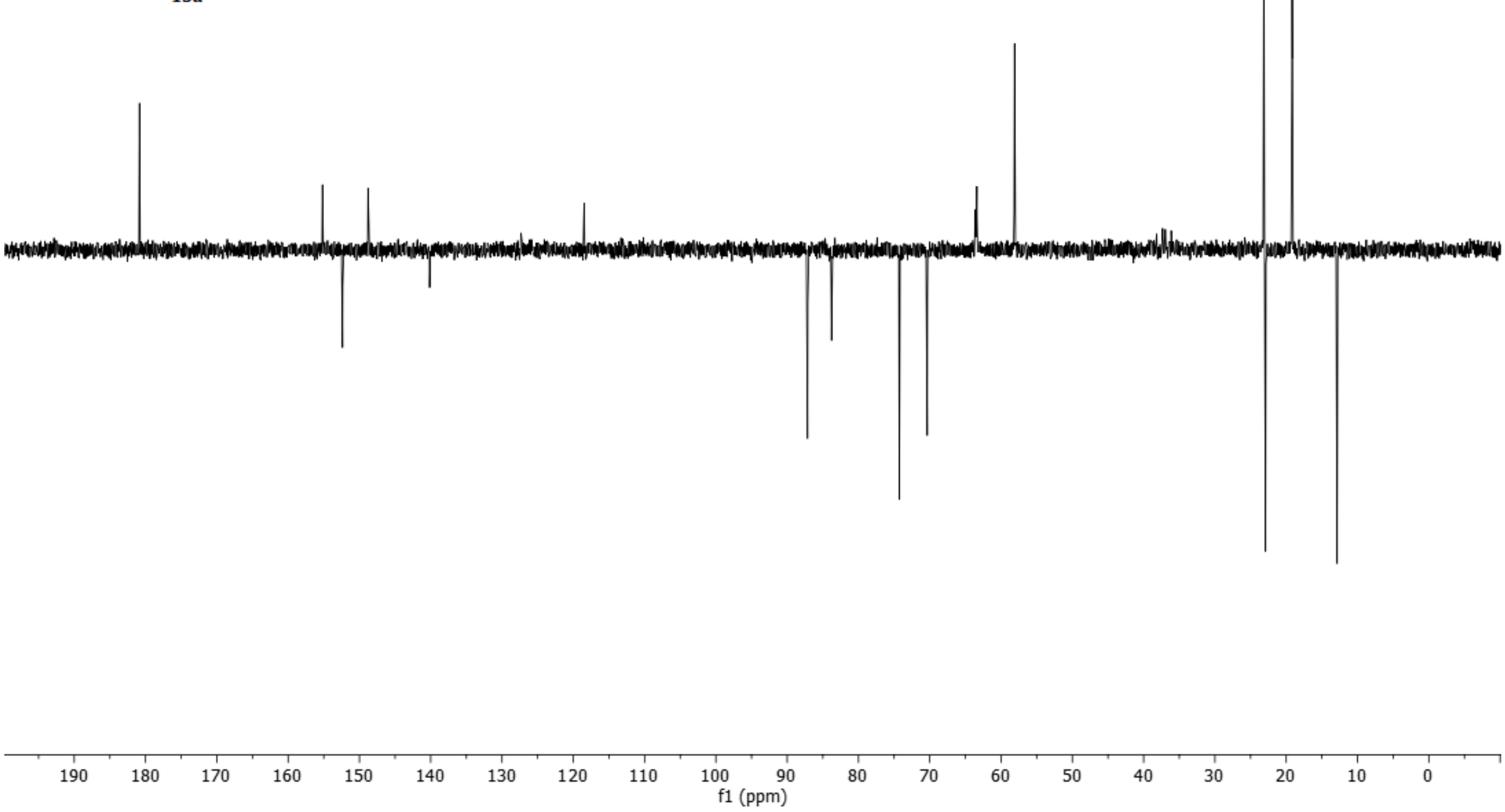

$^{31}P\{^{1}H\}$ NMR, 162 MHz,

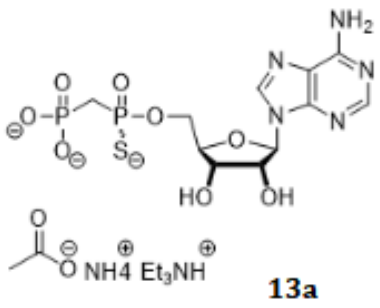

13a

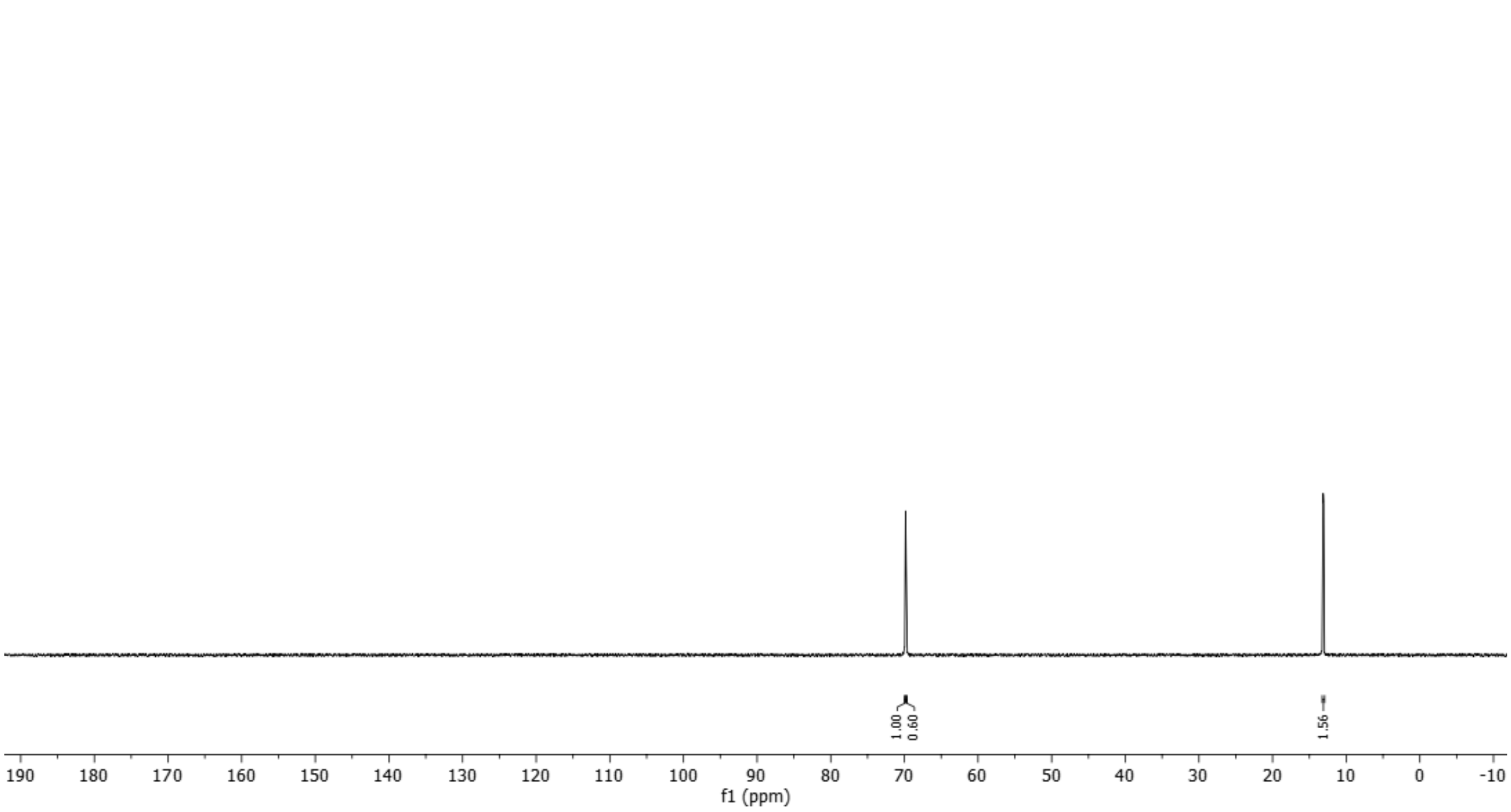

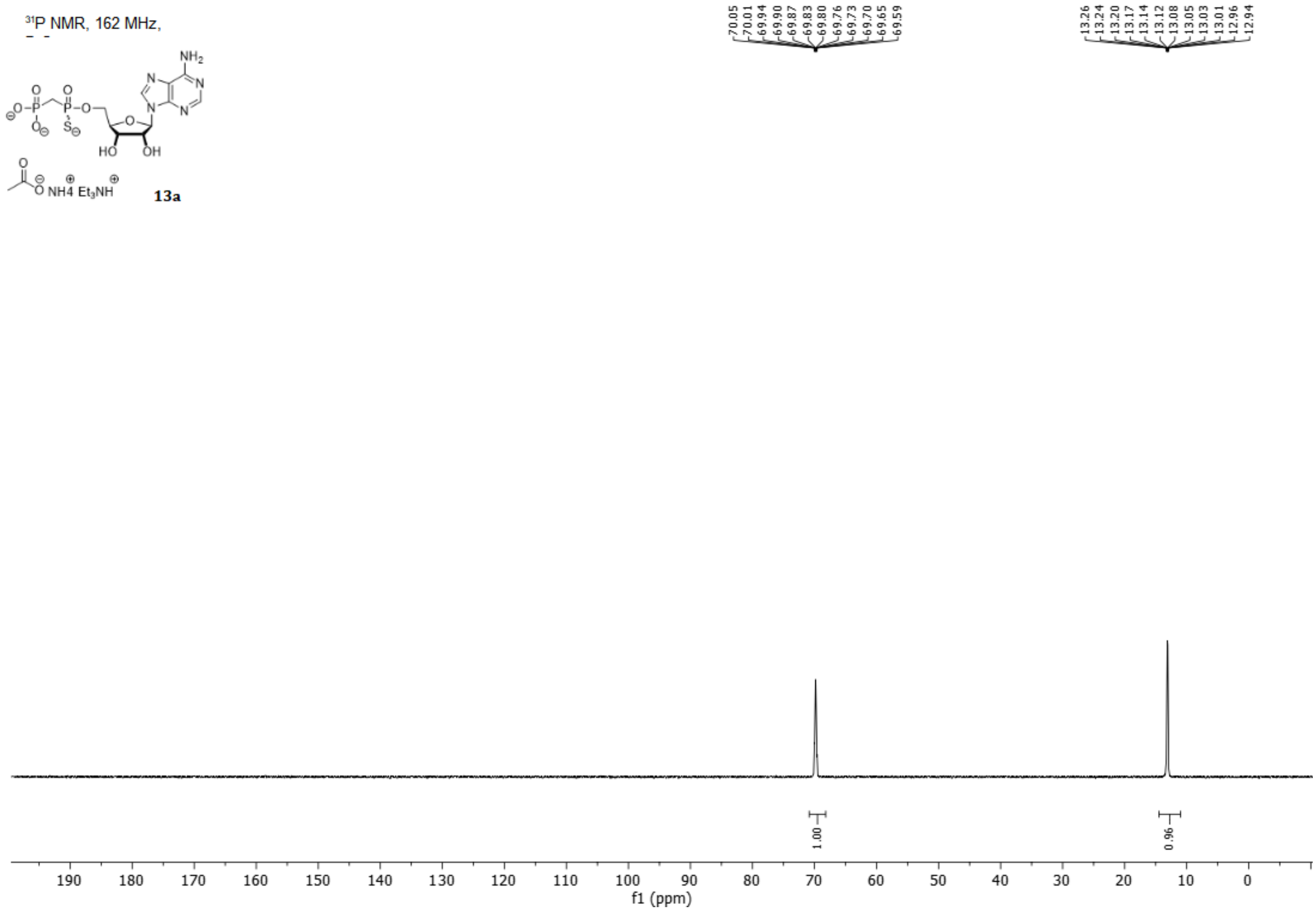

31P NMR, 162 MHz,
13a
70.05 70.01 69.94 69.90 69.87 69.83 69.80 69.76 69.73 69.70 69.65 69.59
13.26 13.24 13.20 13.17 13.14 13.12 13.08 13.05 13.03 13.01 12.96 12.94
1.00
0.96
f1 (ppm)

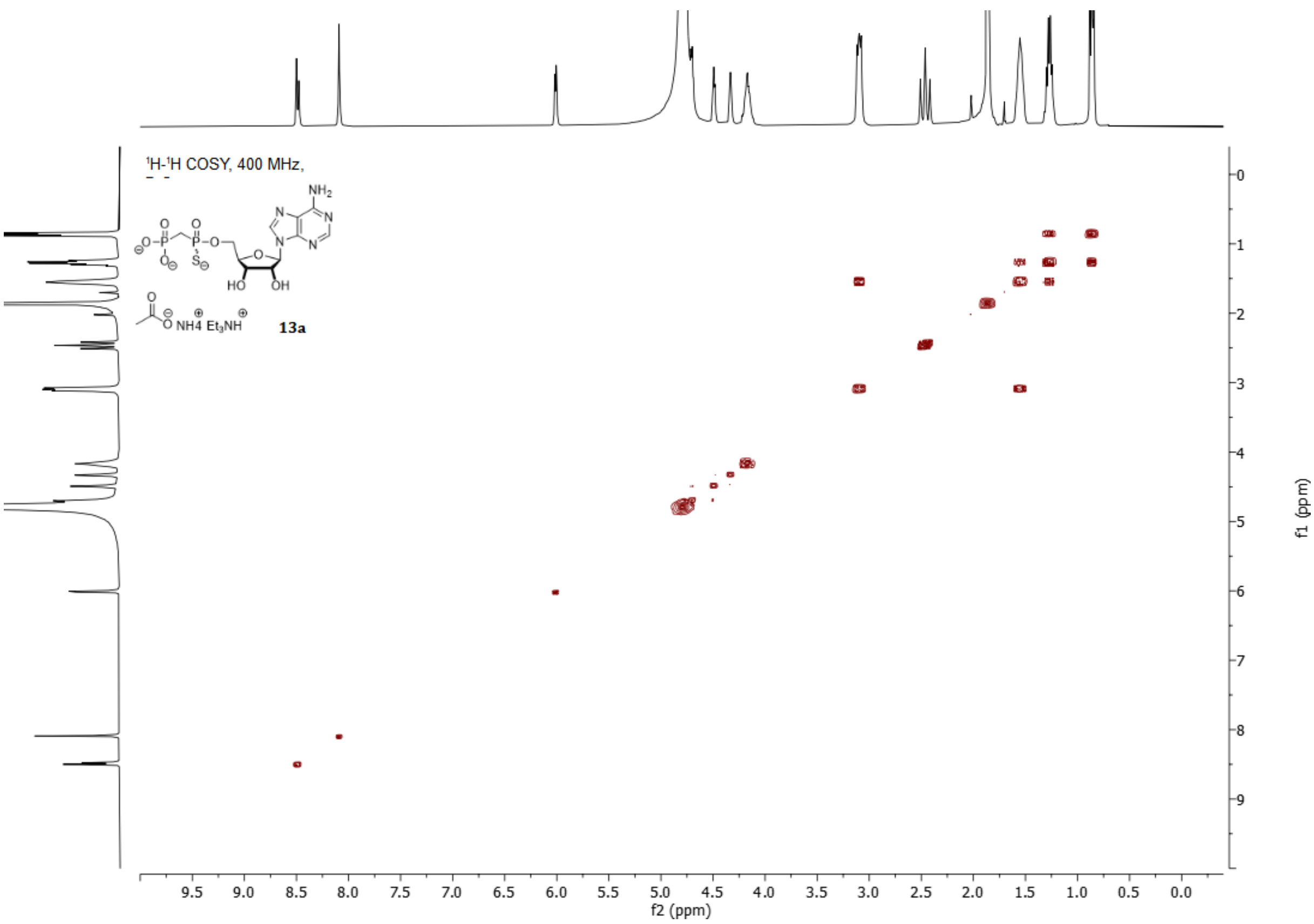

1H-1H COSY, 400 MHz,
13a
f2 (ppm)
f1 (ppm)

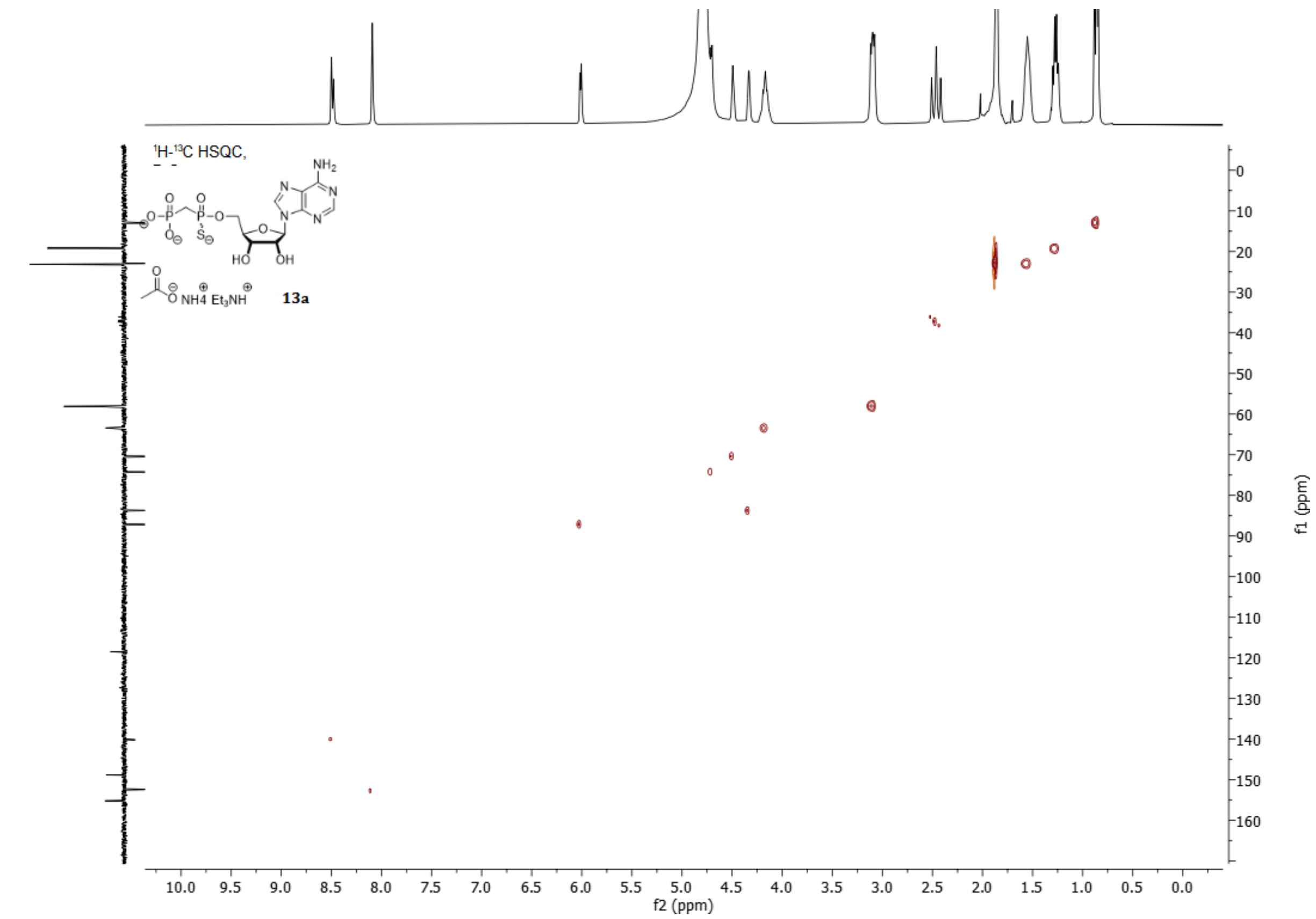

1H-13C HSQC,
NH2
HO
OH
NH4 Et3NH
13a
f2 (ppm)
f1 (ppm)

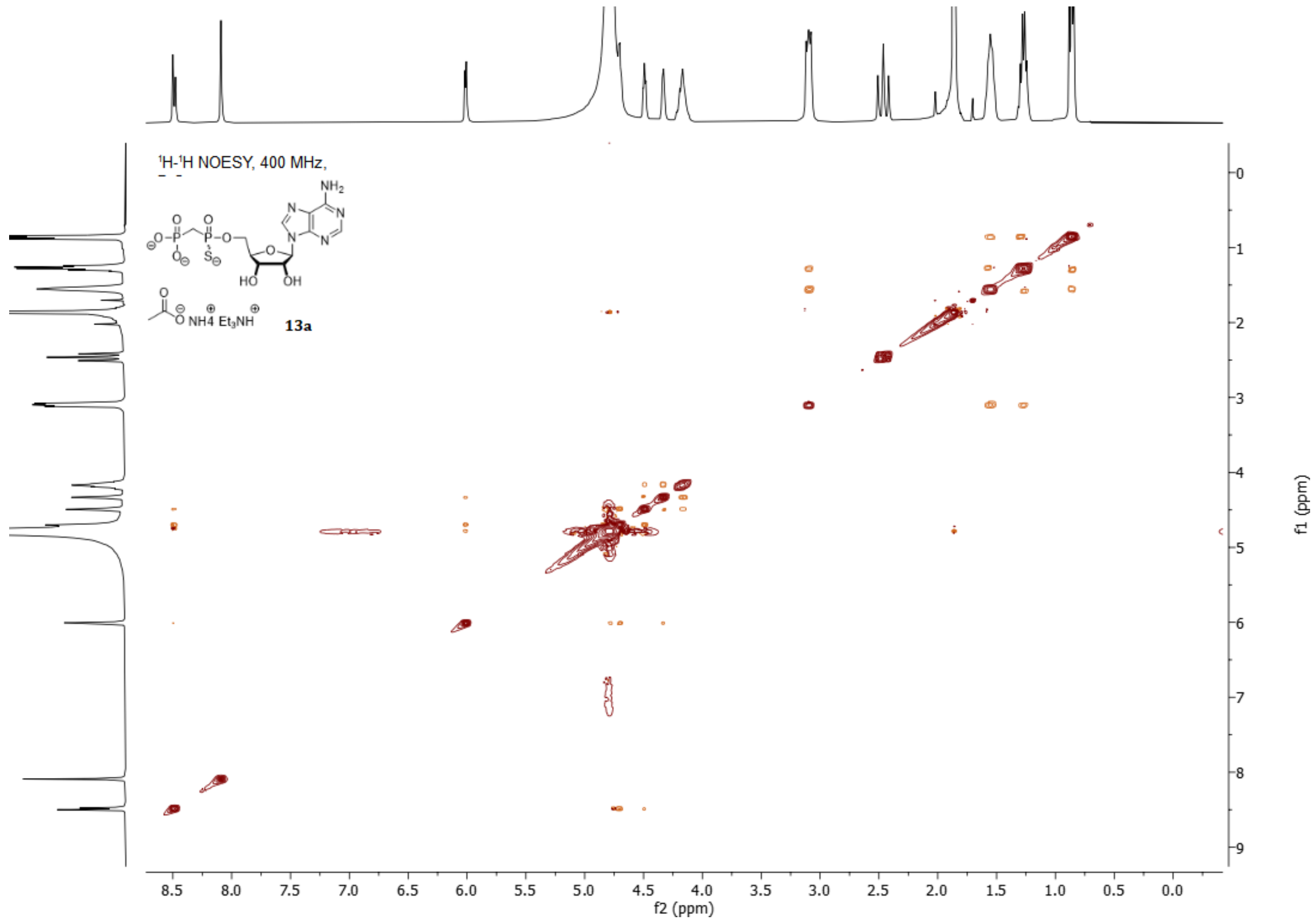

1H-1H NOESY, 400 MHz,
NH2
HO
OH
NH4 Et3NH
13a
f2 (ppm)
f1 (ppm)

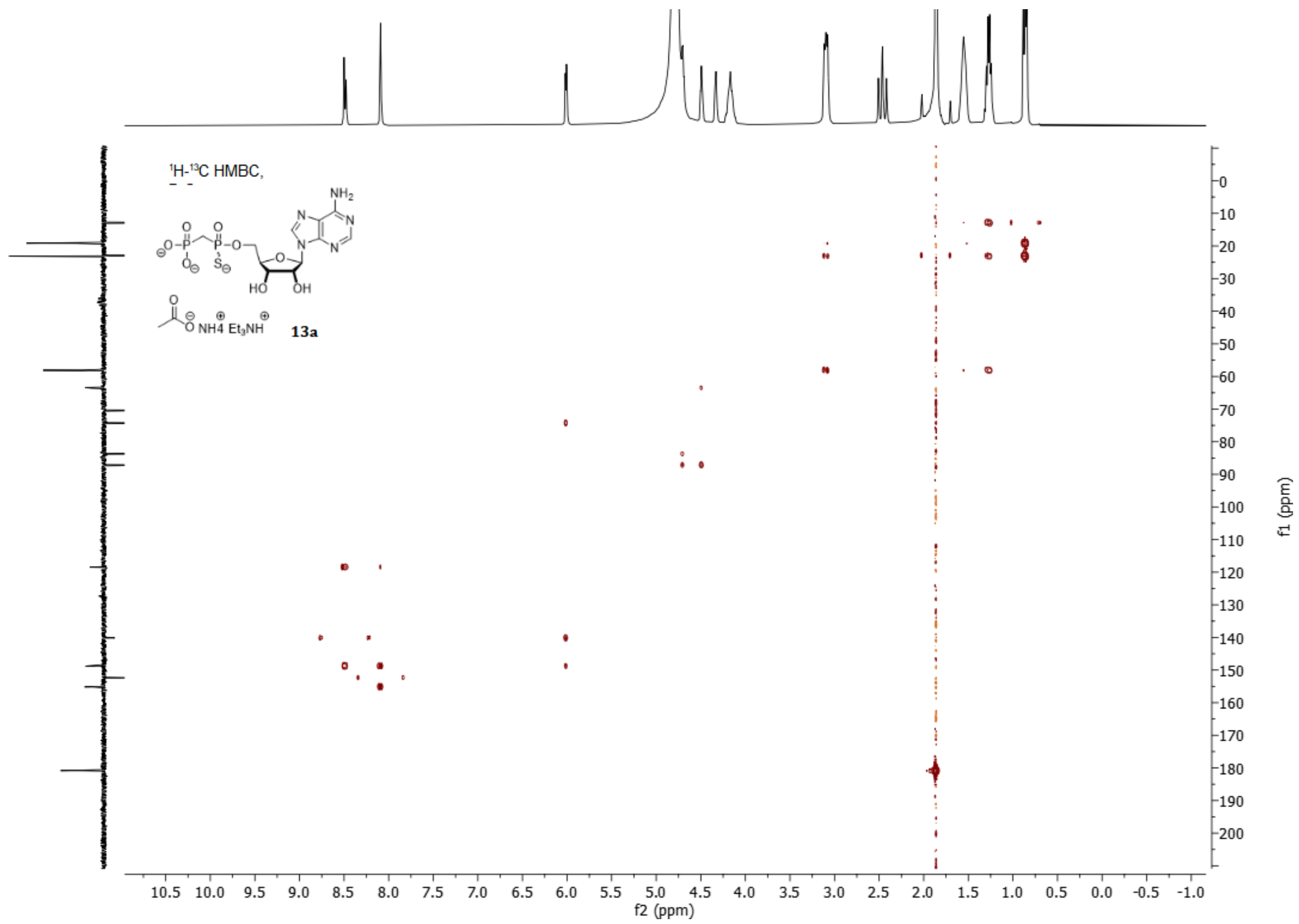
1H-13C HMBC,
13a
f1 (ppm)
f2 (ppm)

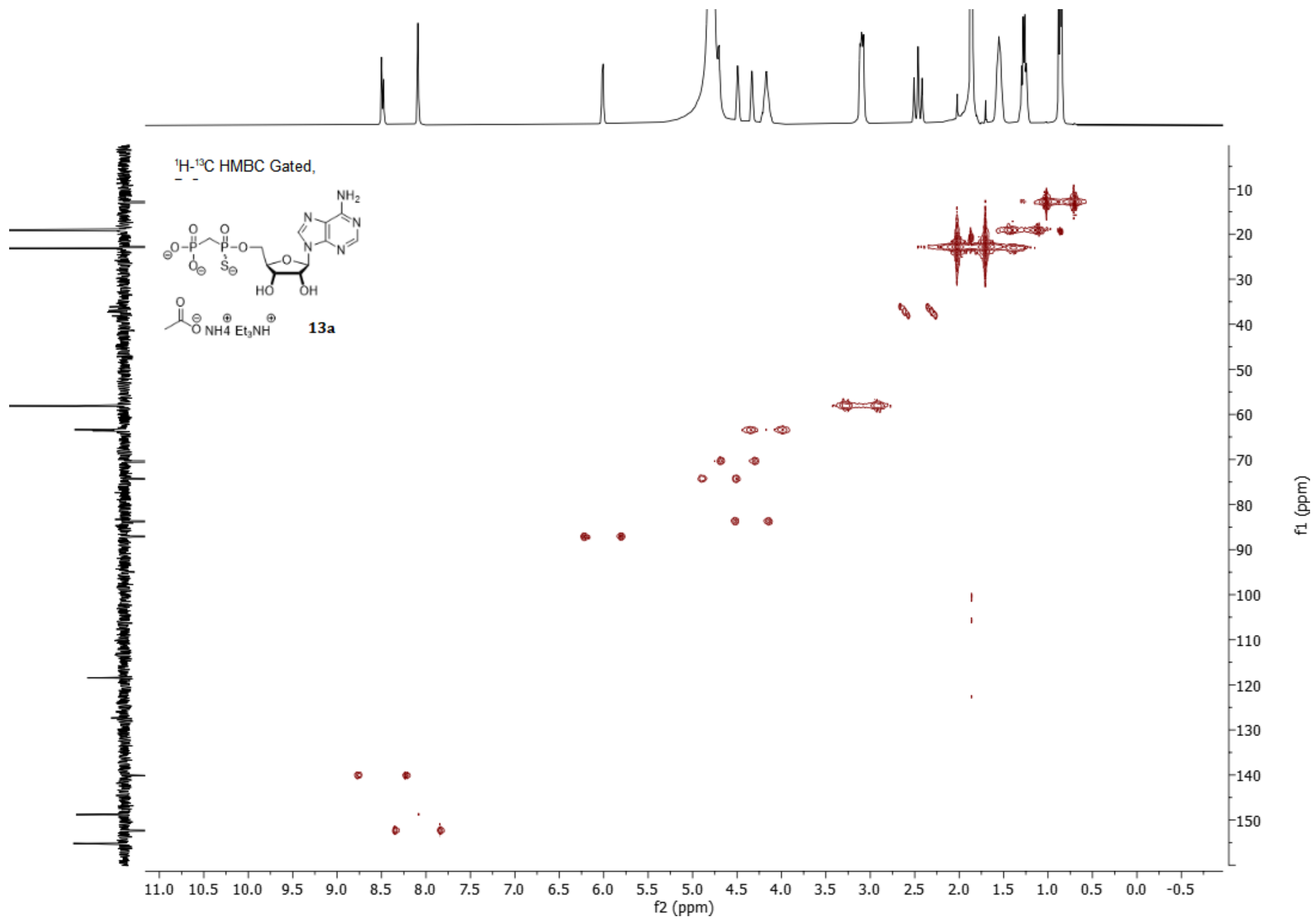
1H-13C HMBC Gated,
13a
f1 (ppm)
f2 (ppm)

me-ADP-αS$_2$

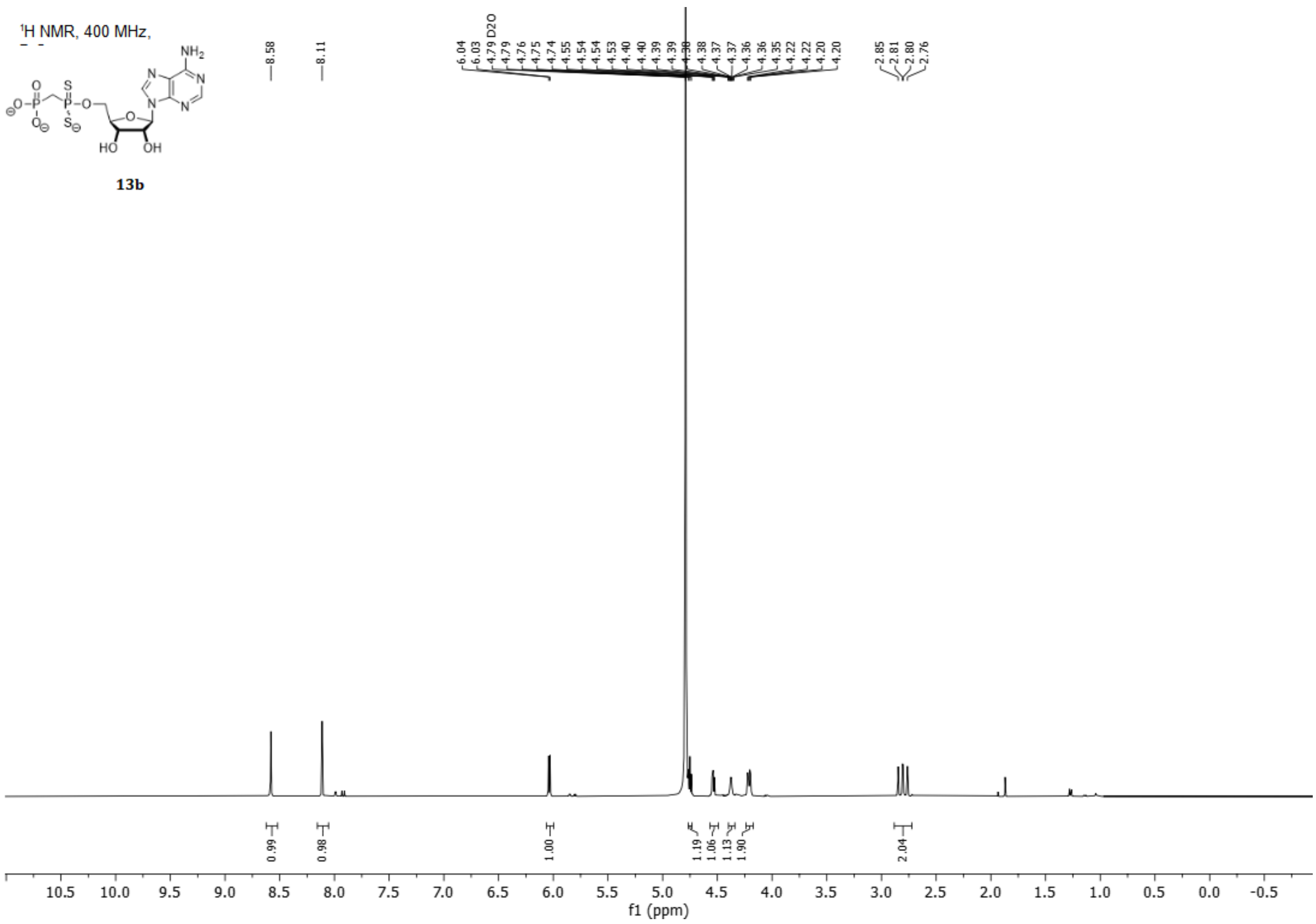

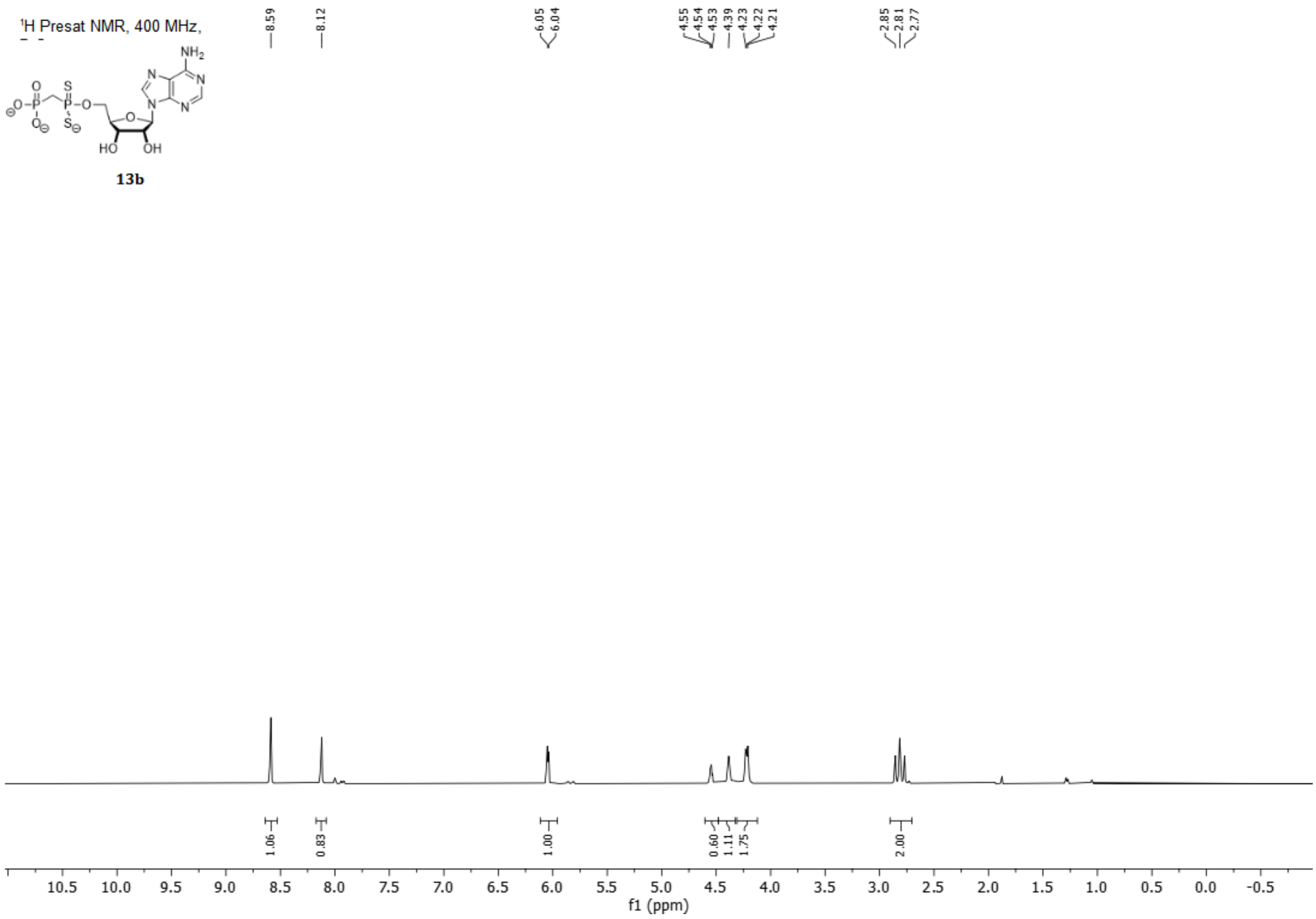

^{13}C NMR, 101 MHz,

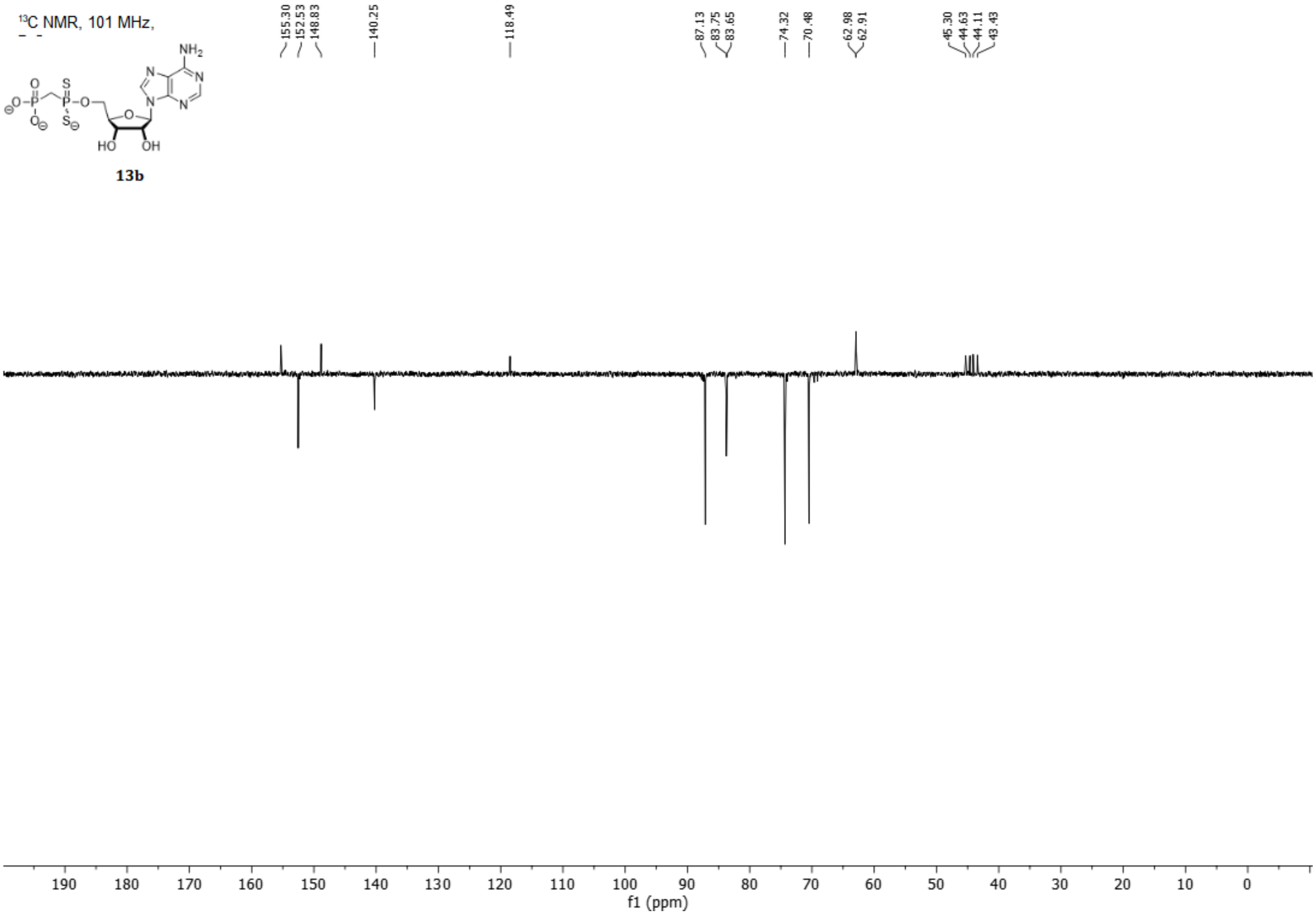

$^{31}P\{^{1}H\}$ NMR, 162 MHz,

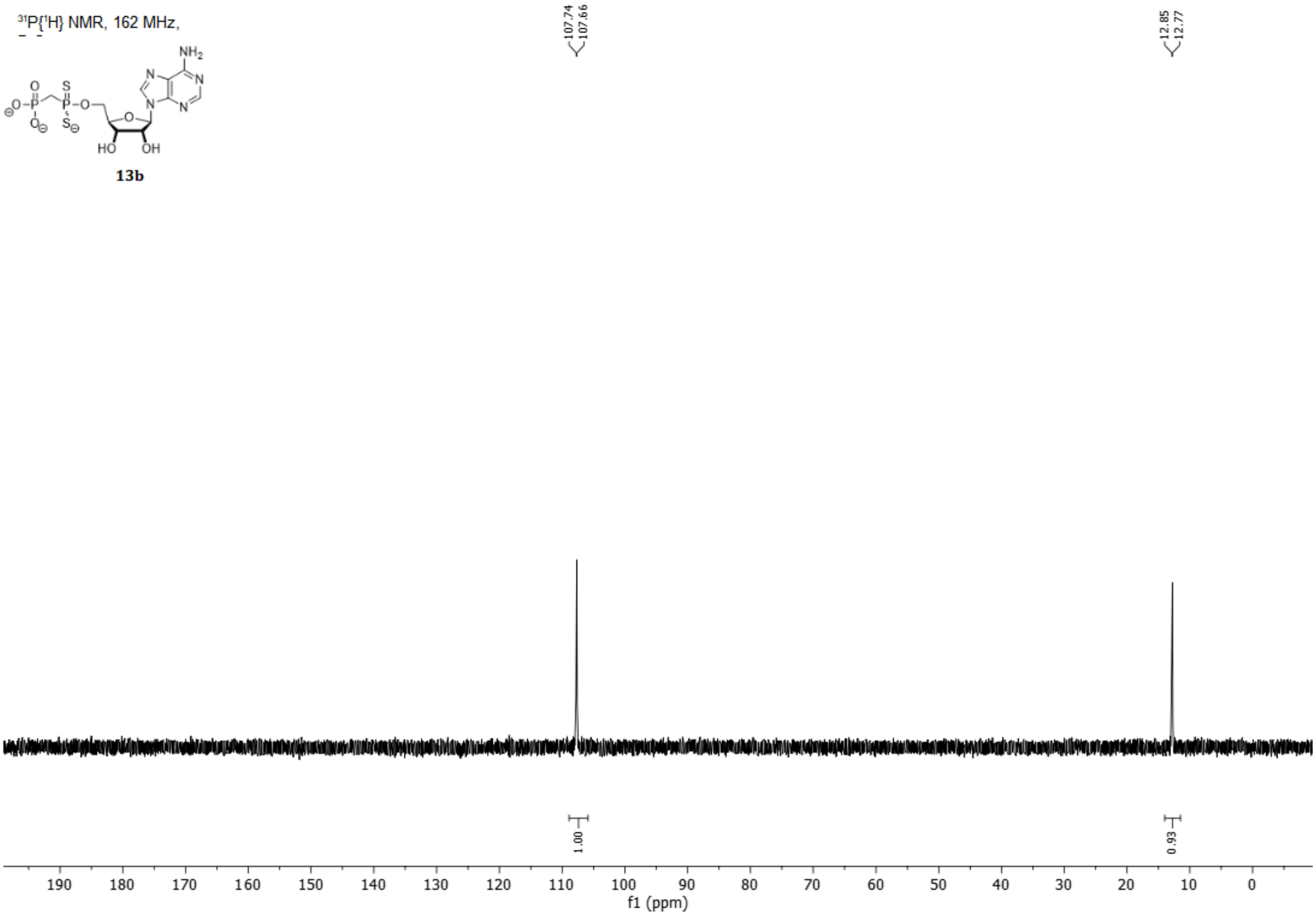

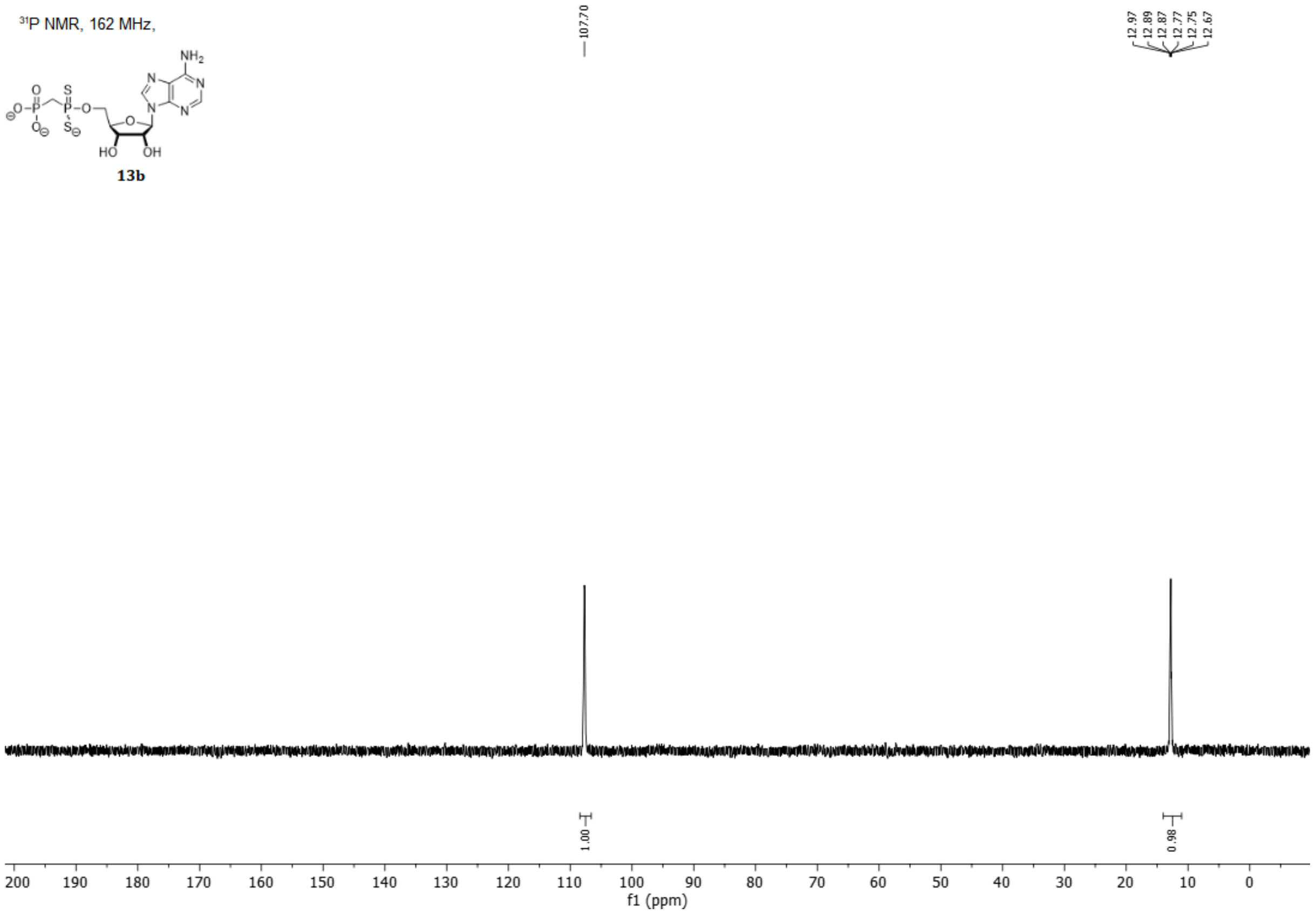

³¹P NMR, 162 MHz,
NH2
13b
107.70
12.97
12.89
12.87
12.77
12.75
12.67
1.00
0.98
f1 (ppm)

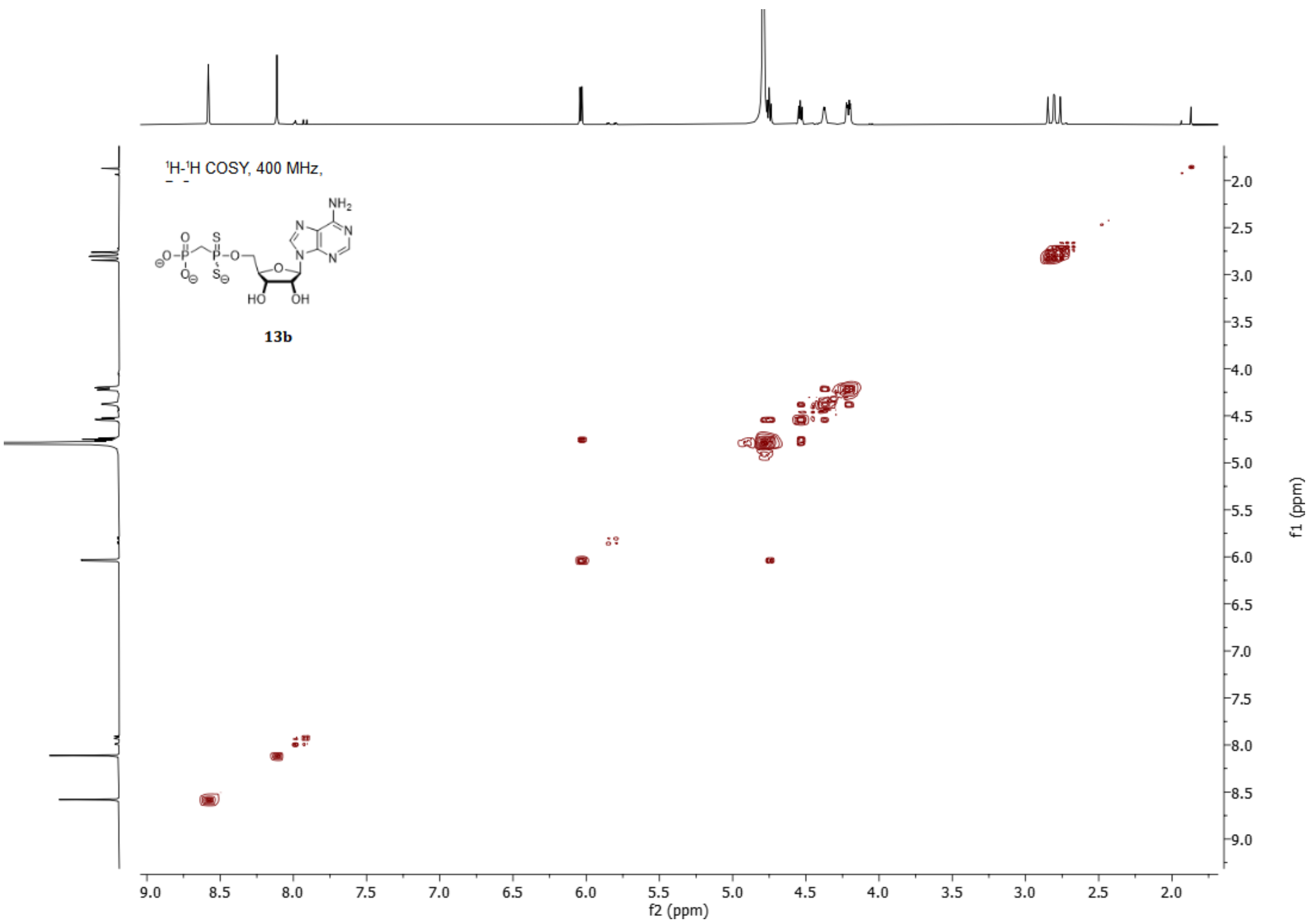

¹H-¹H COSY, 400 MHz,
NH2
13b
f2 (ppm)
f1 (ppm)

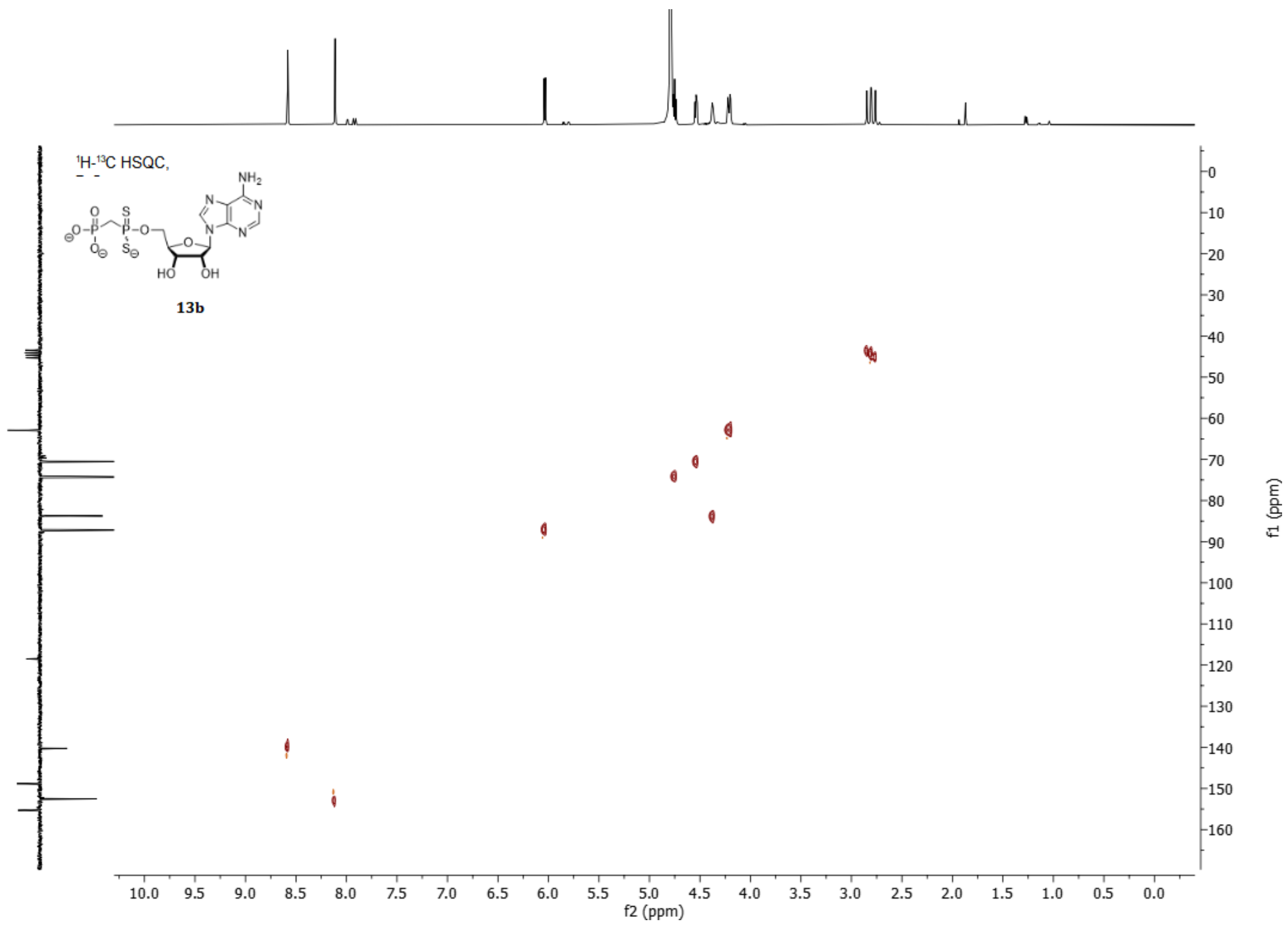

1H-13C HSQC,
NH2
13b
f1 (ppm)
f2 (ppm)

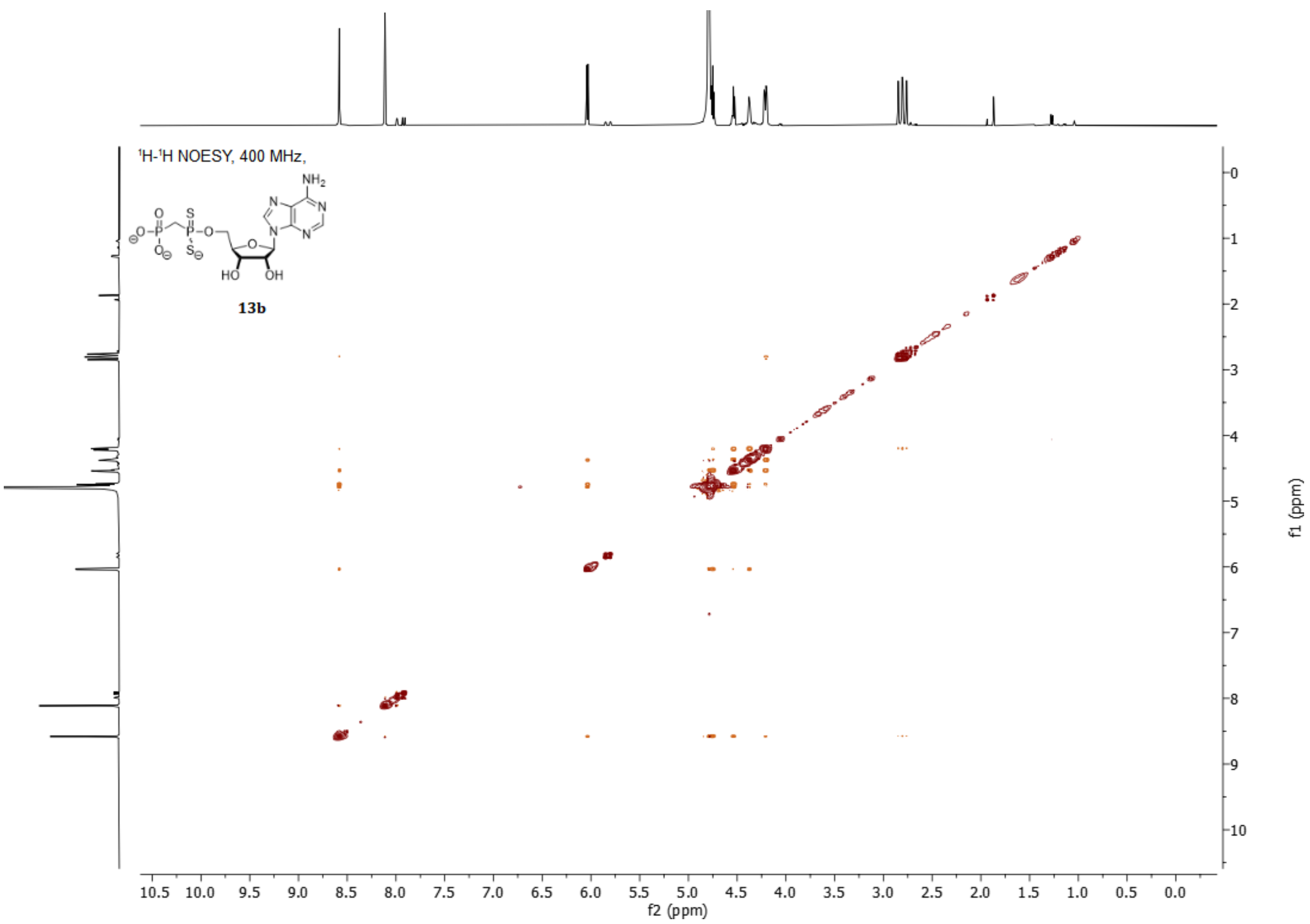

1H-1H NOESY, 400 MHz,
NH2
13b
f1 (ppm)
f2 (ppm)

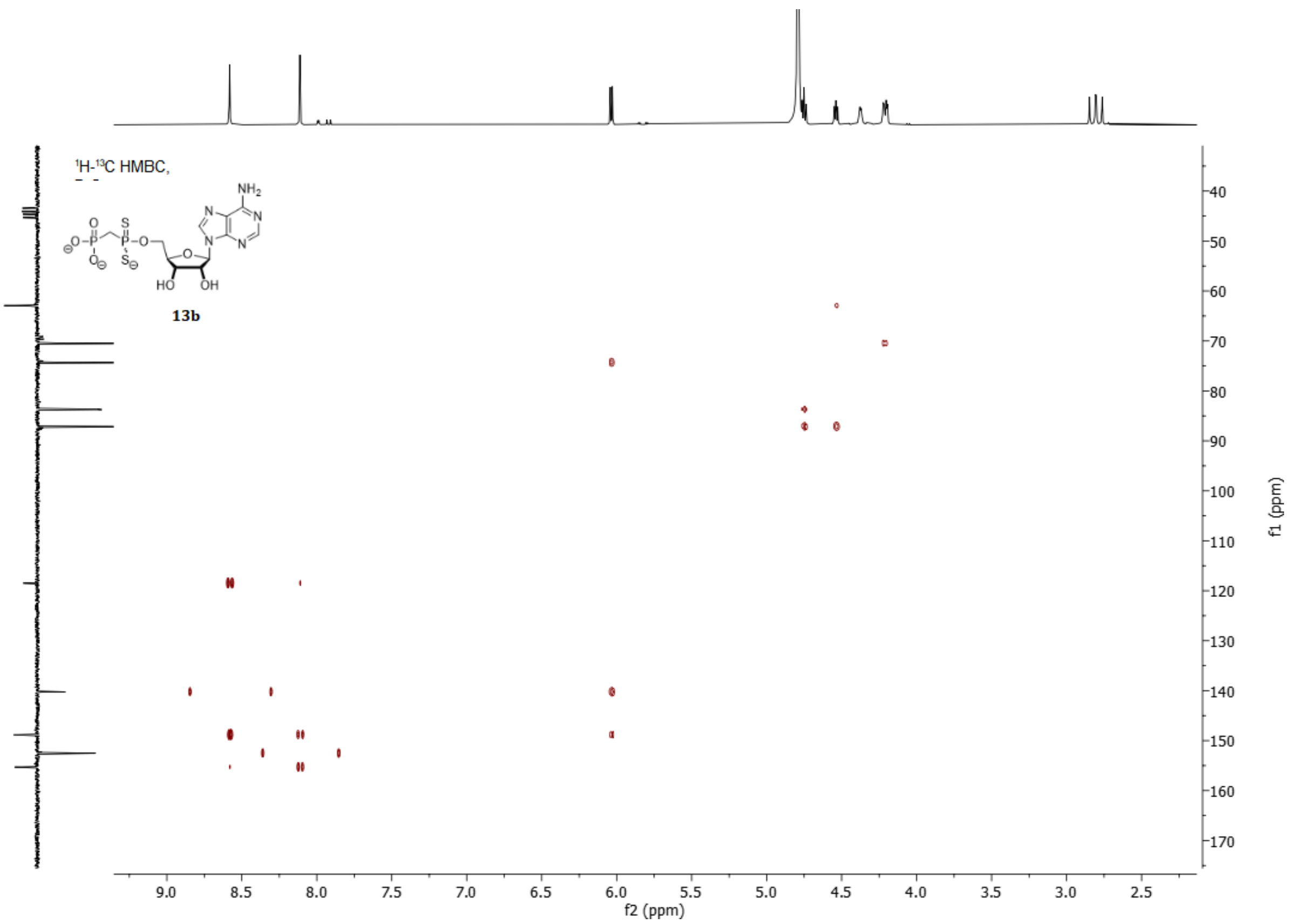

me-CDP-αS tetraester 15

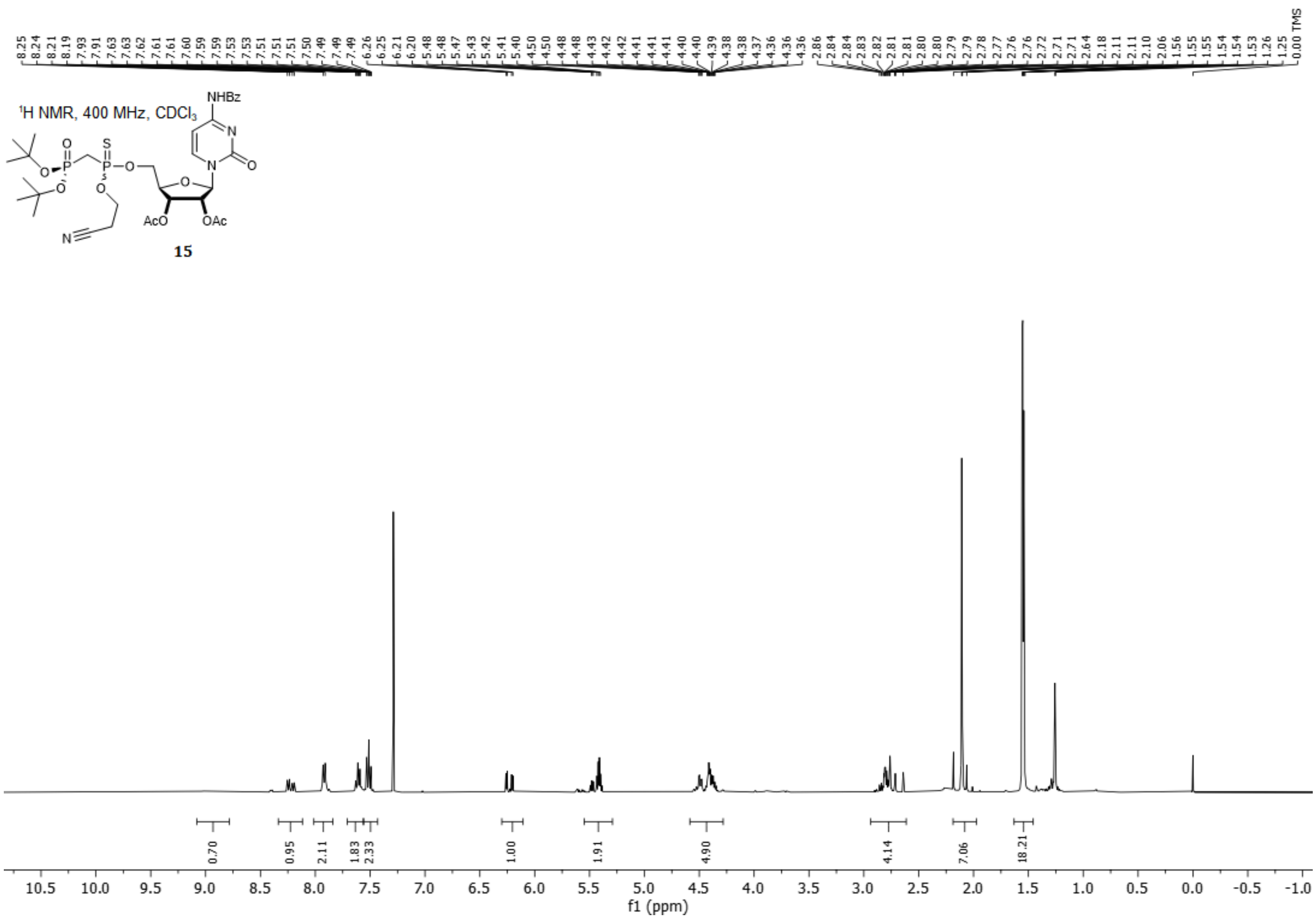

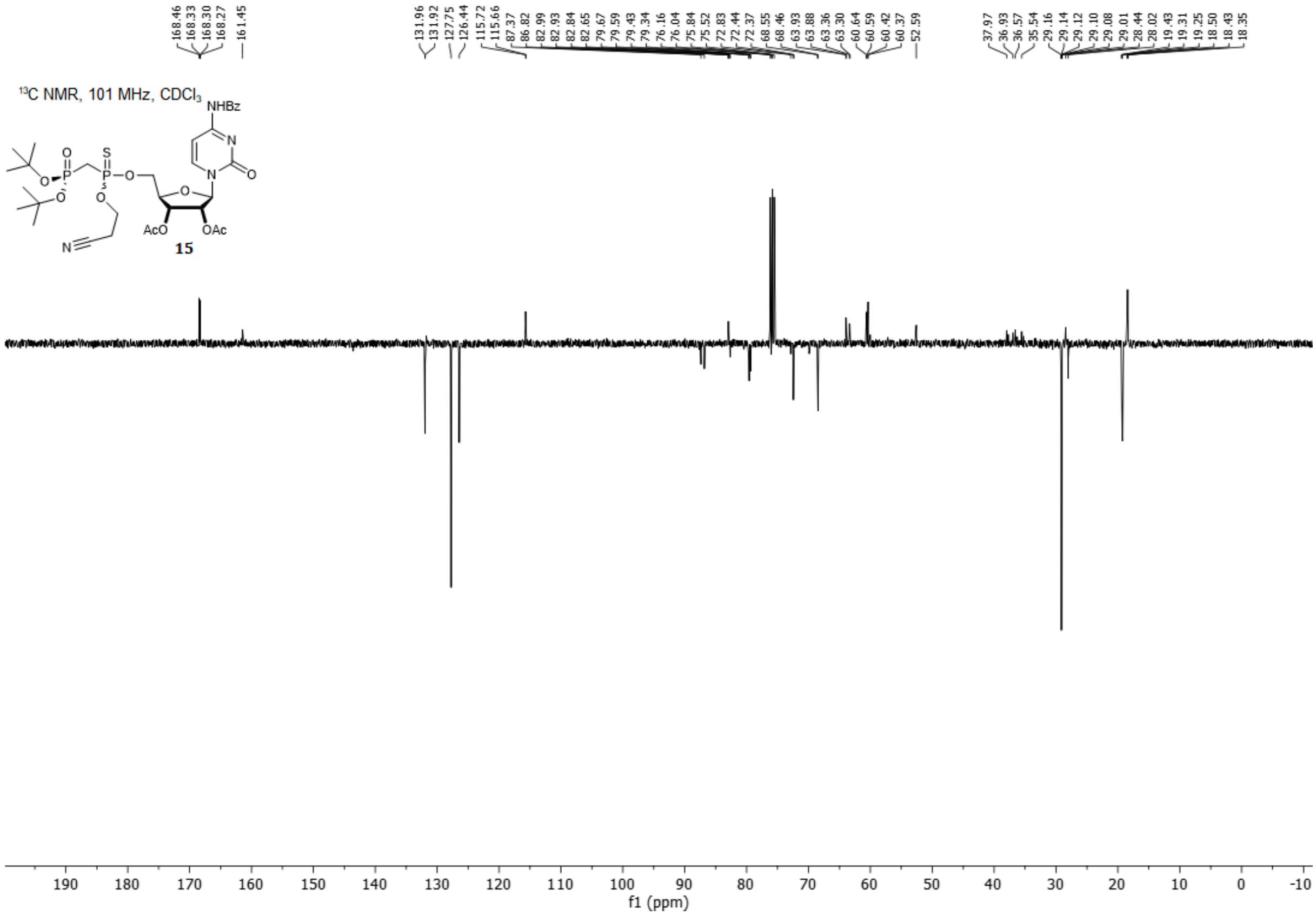
168.46
168.33
168.30
168.27
161.45
131.96
131.92
127.75
126.44
115.72
115.66
87.37
86.82
82.99
82.93
82.84
82.65
79.67
79.59
79.43
79.34
76.16
76.04
75.84
75.52
72.83
72.44
72.37
68.55
68.46
63.93
63.88
63.36
63.30
60.64
60.59
60.42
60.37
52.59
37.97
36.93
36.57
35.54
29.16
29.14
29.12
29.10
29.08
29.01
28.44
28.02
19.43
19.31
19.25
18.50
18.43
18.35
13C NMR, 101 MHz, CDCl3
NHBz
N
O
S
P
O
AcO
OAc
N
15
190 180 170 160 150 140 130 120 110 100 90 80 70 60 50 40 30 20 10 0 -10
f1 (ppm)

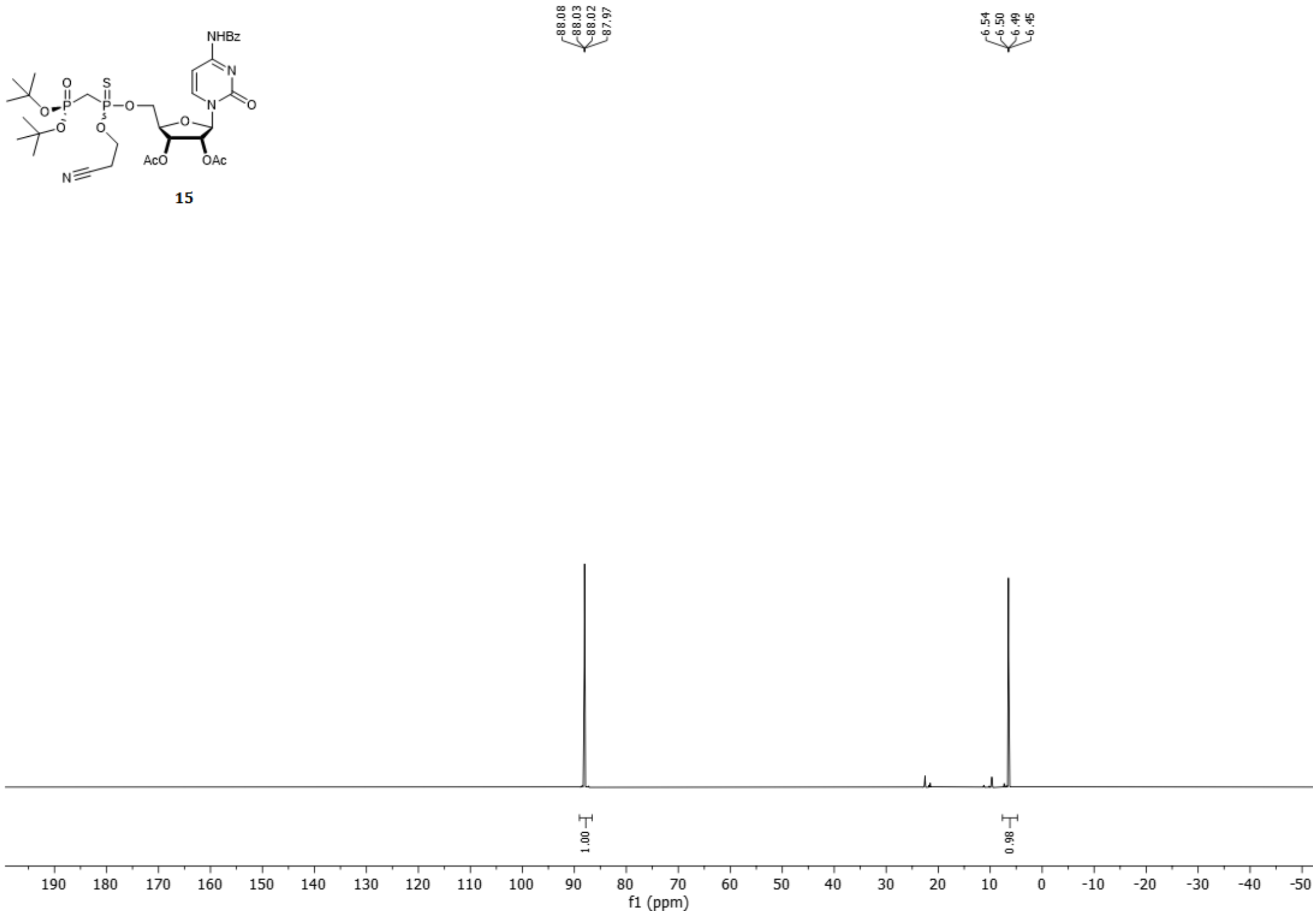
88.08
88.03
88.02
87.97
6.54
6.50
6.49
6.45
NHBz
15
1.00
0.98
190 180 170 160 150 140 130 120 110 100 90 80 70 60 50 40 30 20 10 0 -10 -20 -30 -40 -50
f1 (ppm)

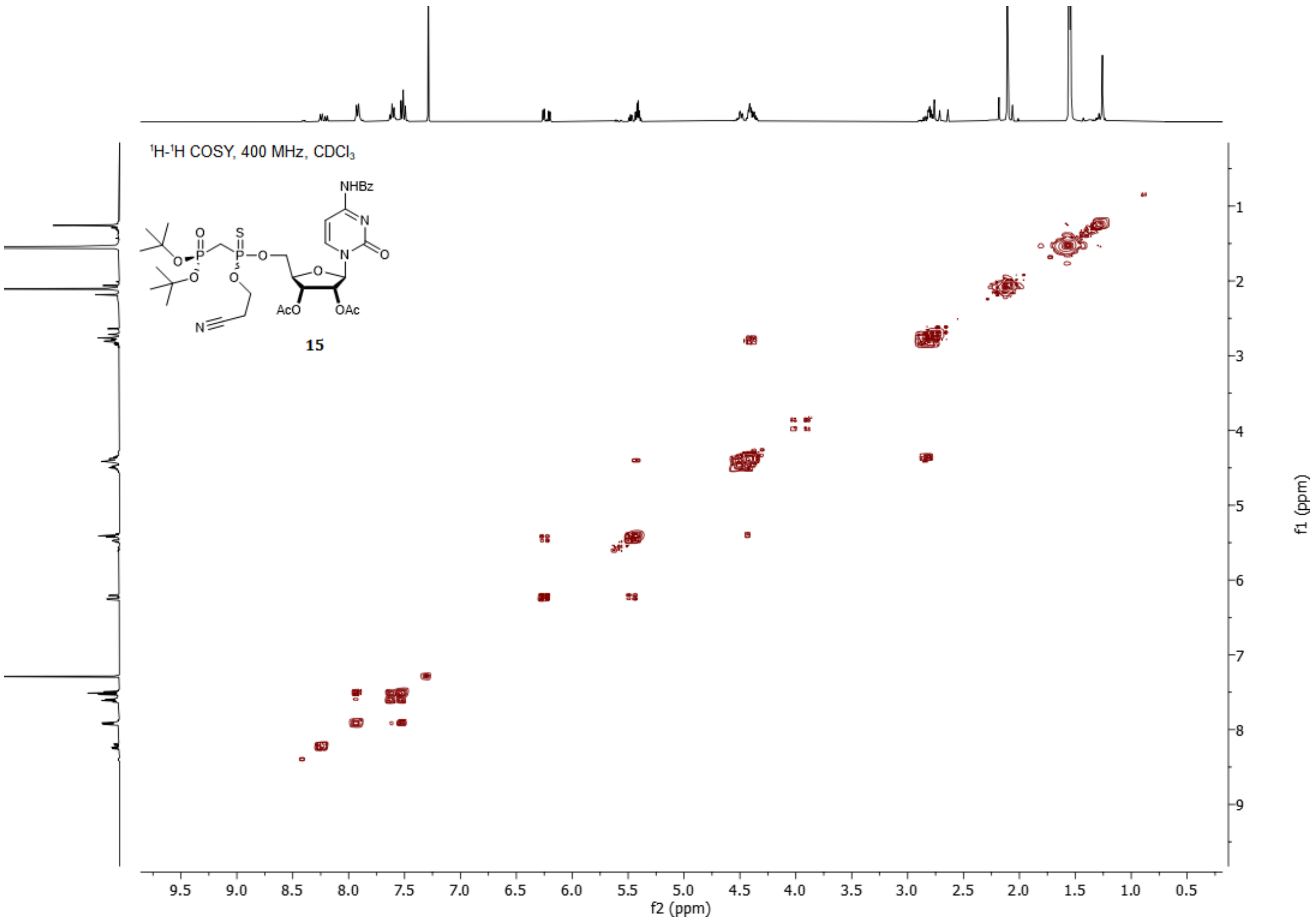
¹H-¹H COSY, 400 MHz, CDCl₃
NHBz
AcO
OAc
15
f2 (ppm)
f1 (ppm)

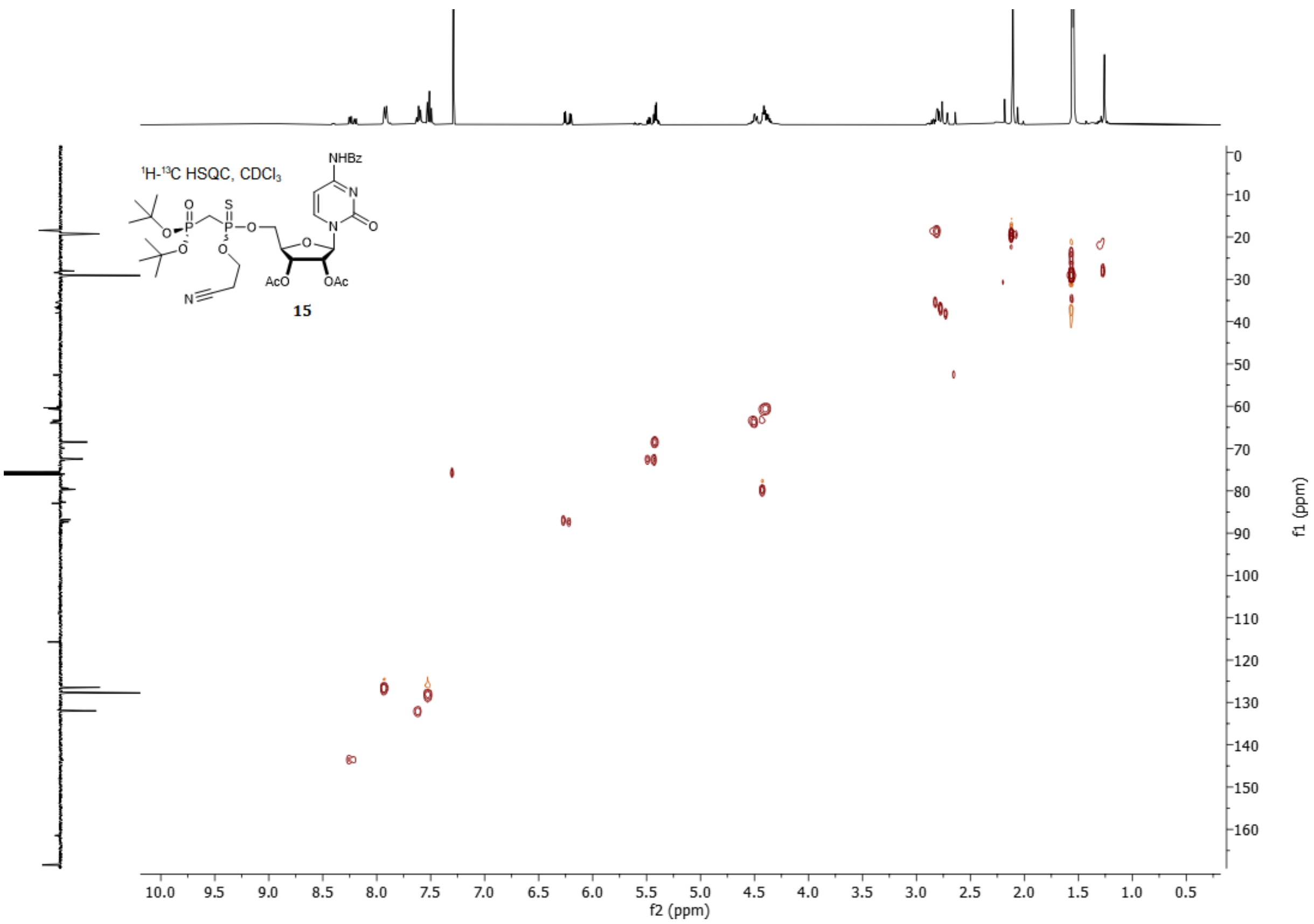
¹H-¹³C HSQC, CDCl₃
NHBz
AcO
OAc
15
f2 (ppm)
f1 (ppm)

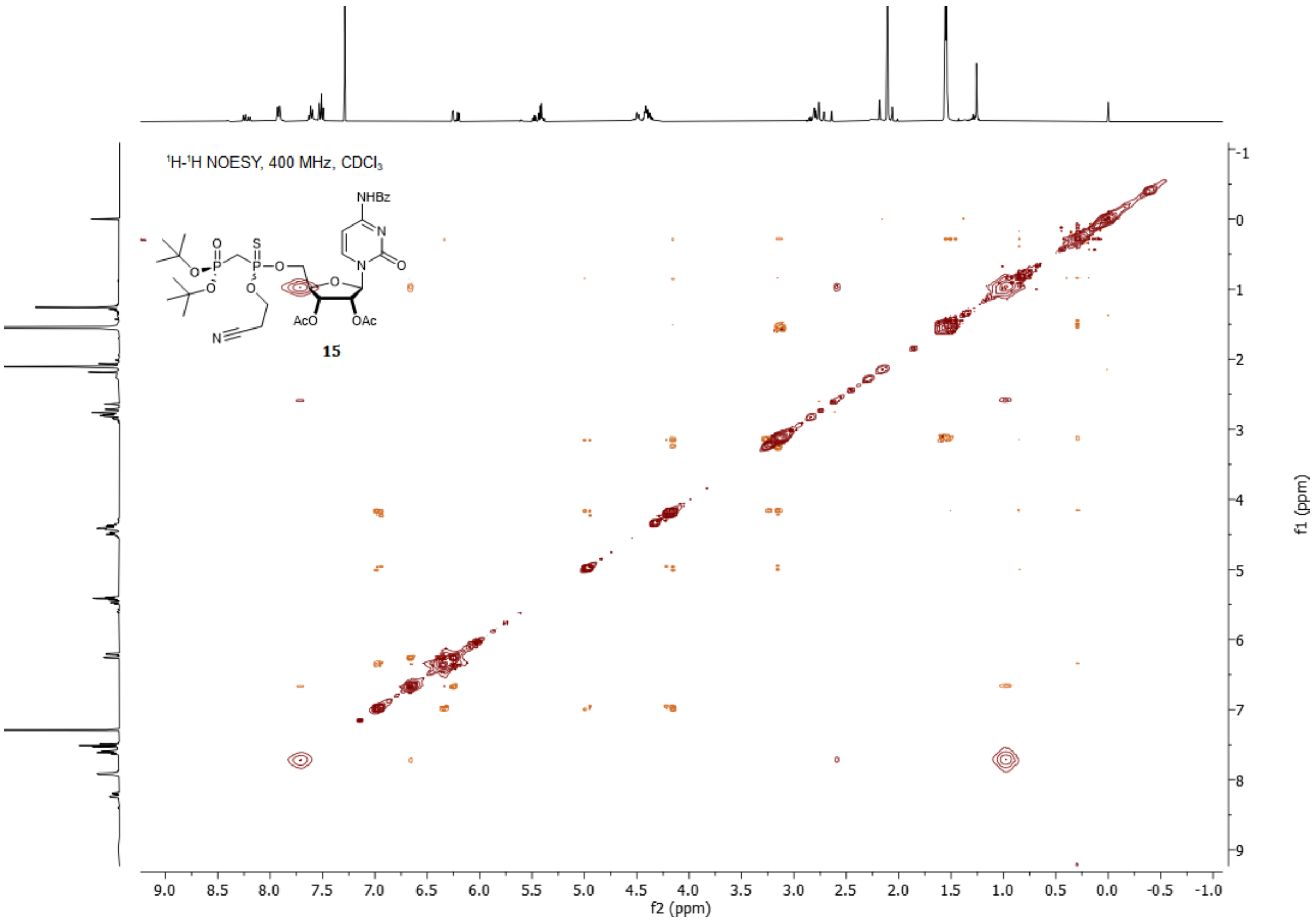

^{1}H-^{1}H NOESY, 400 MHz, CDCl$_3$
NHBz
AcO
OAc
15
f2 (ppm)
f1 (ppm)

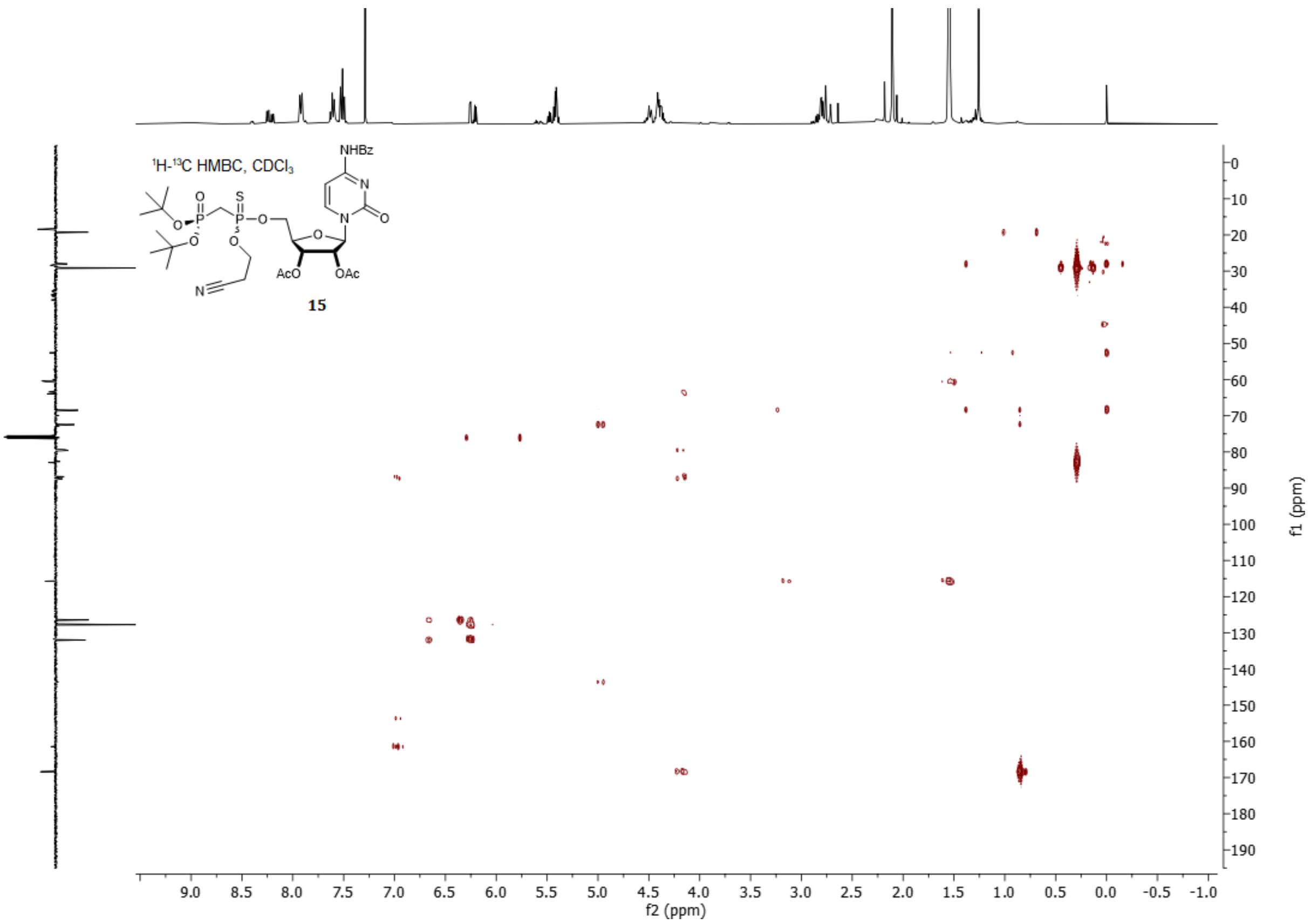

^{1}H-^{13}C HMBC, CDCl$_3$
NHBz
AcO
OAc
15
f2 (ppm)
f1 (ppm)

me-CDP-αS

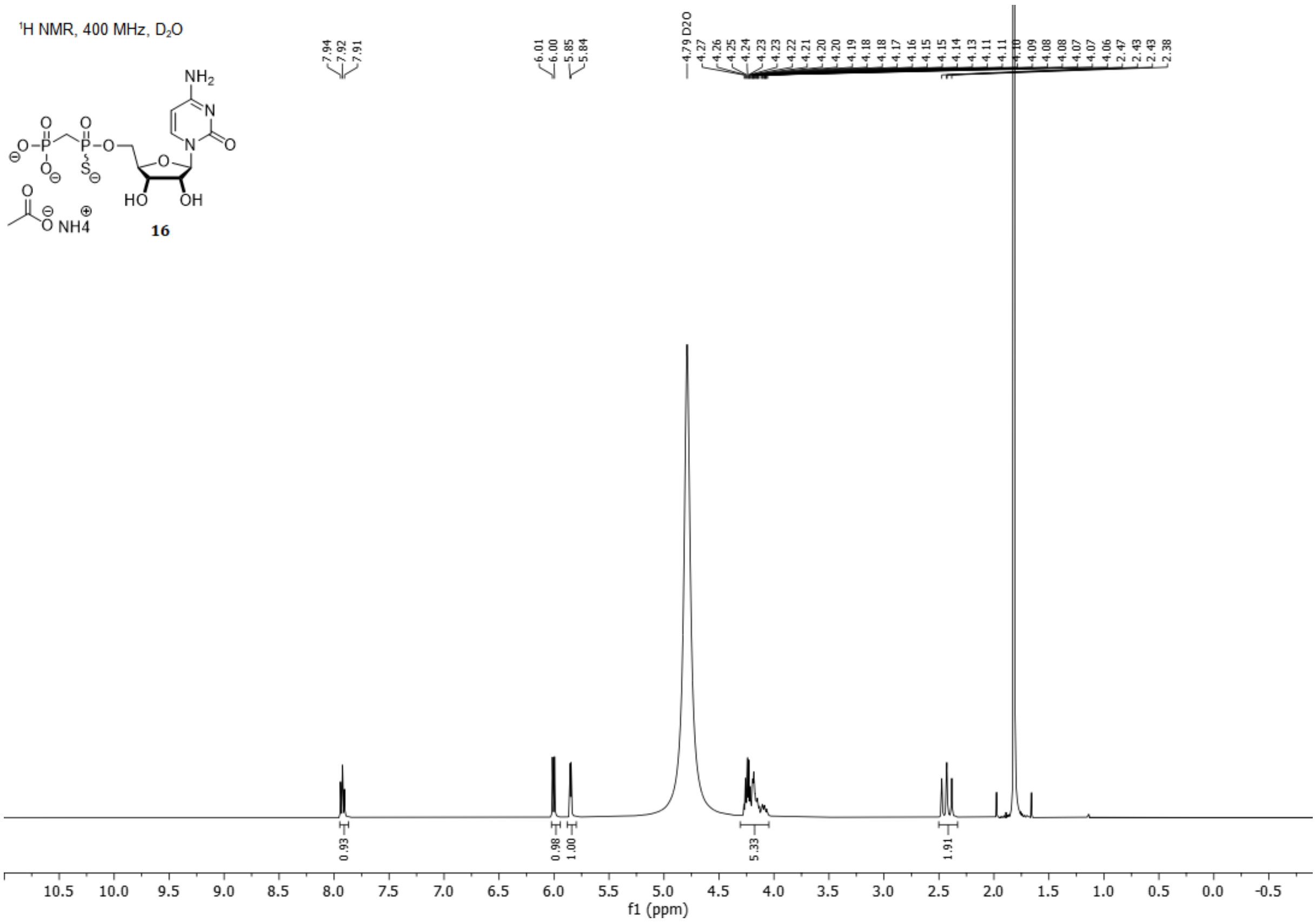

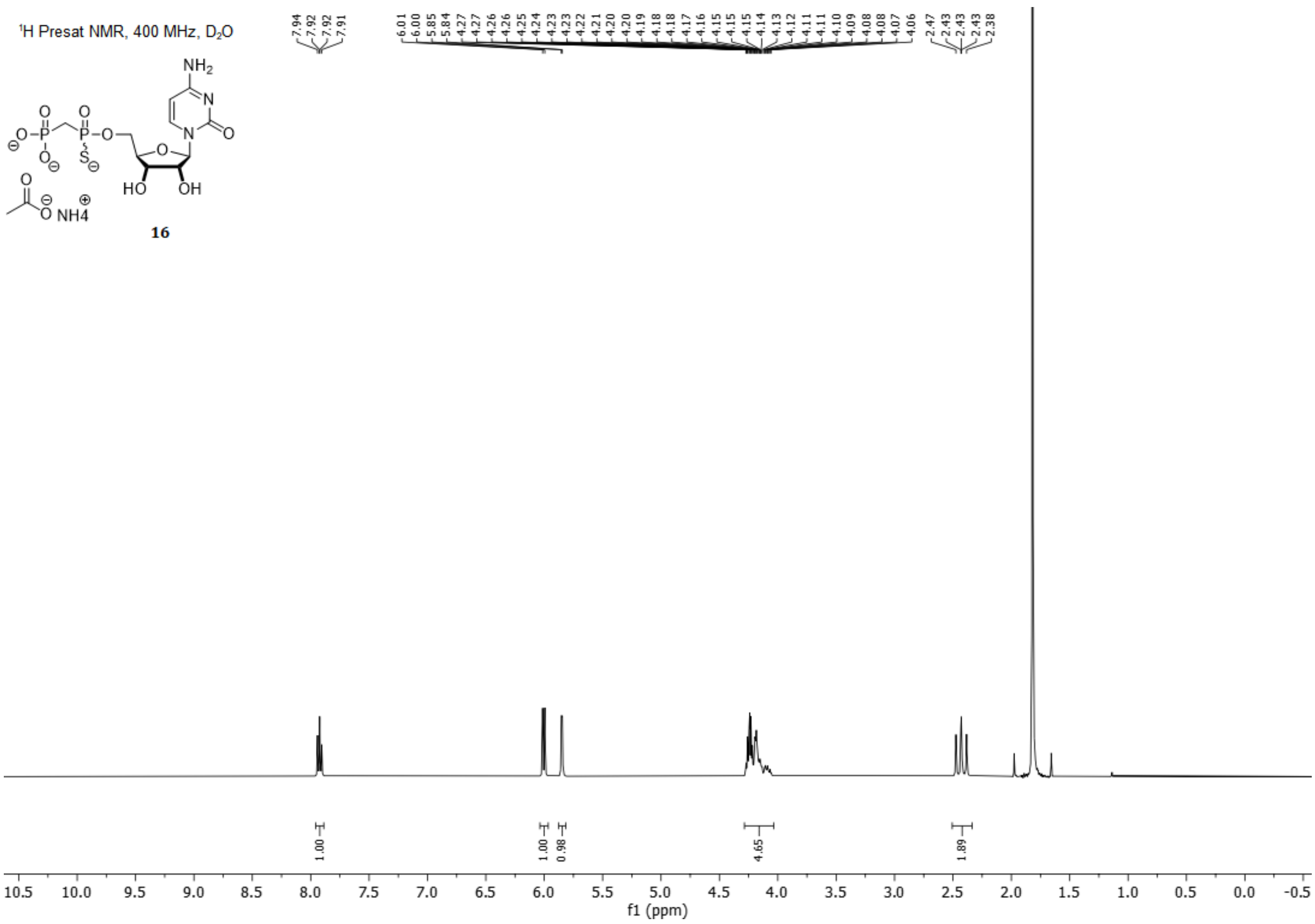

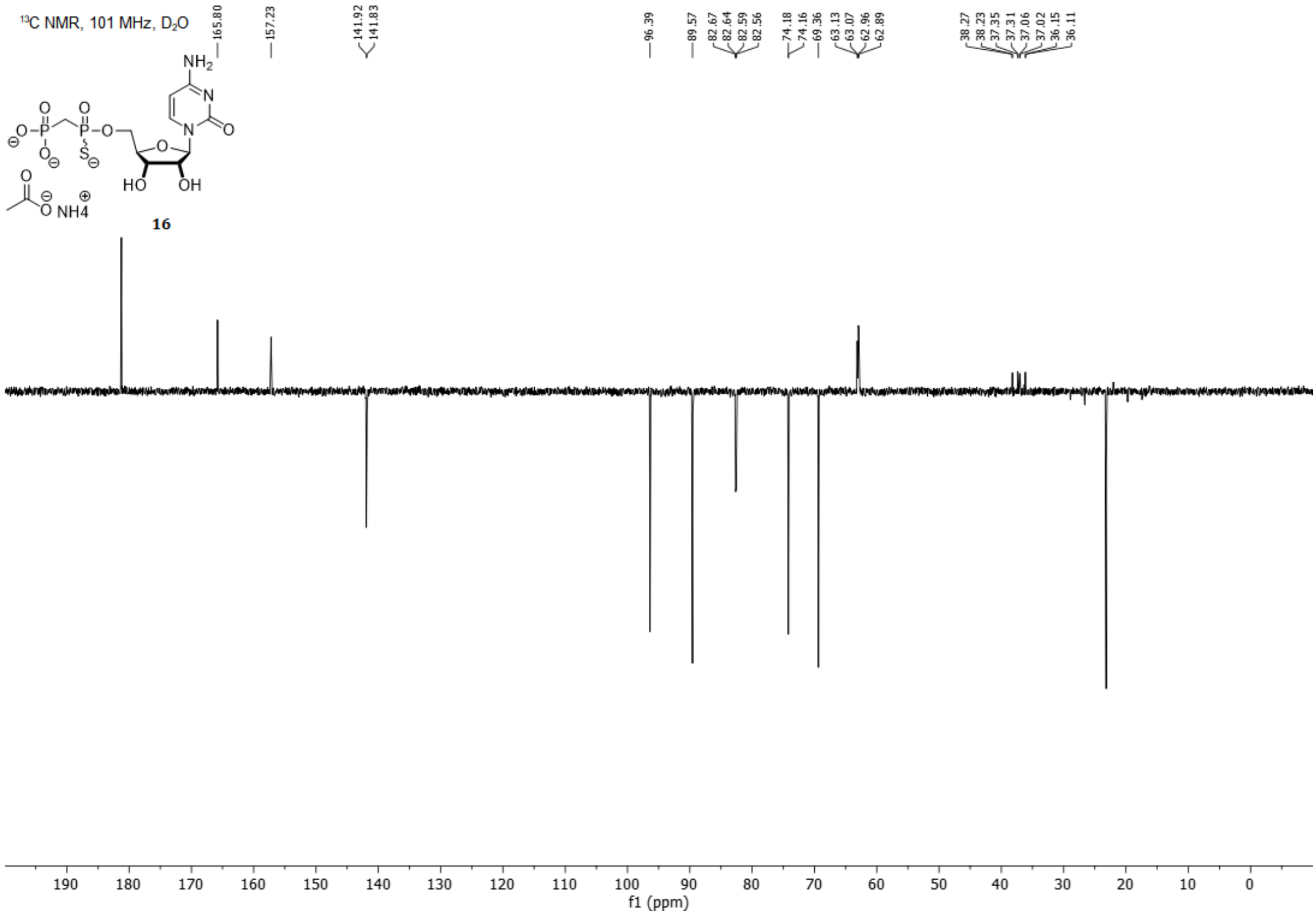
13C NMR, 101 MHz, D2O
165.80
157.23
141.92
141.83
96.39
89.57
82.67
82.64
82.59
82.56
74.18
74.16
69.36
63.13
63.07
62.96
62.89
38.27
38.23
37.35
37.31
37.06
37.02
36.15
36.11
NH2
N
O
S
HO
OH
NH4
16
190 180 170 160 150 140 130 120 110 100 90 80 70 60 50 40 30 20 10 0
f1 (ppm)

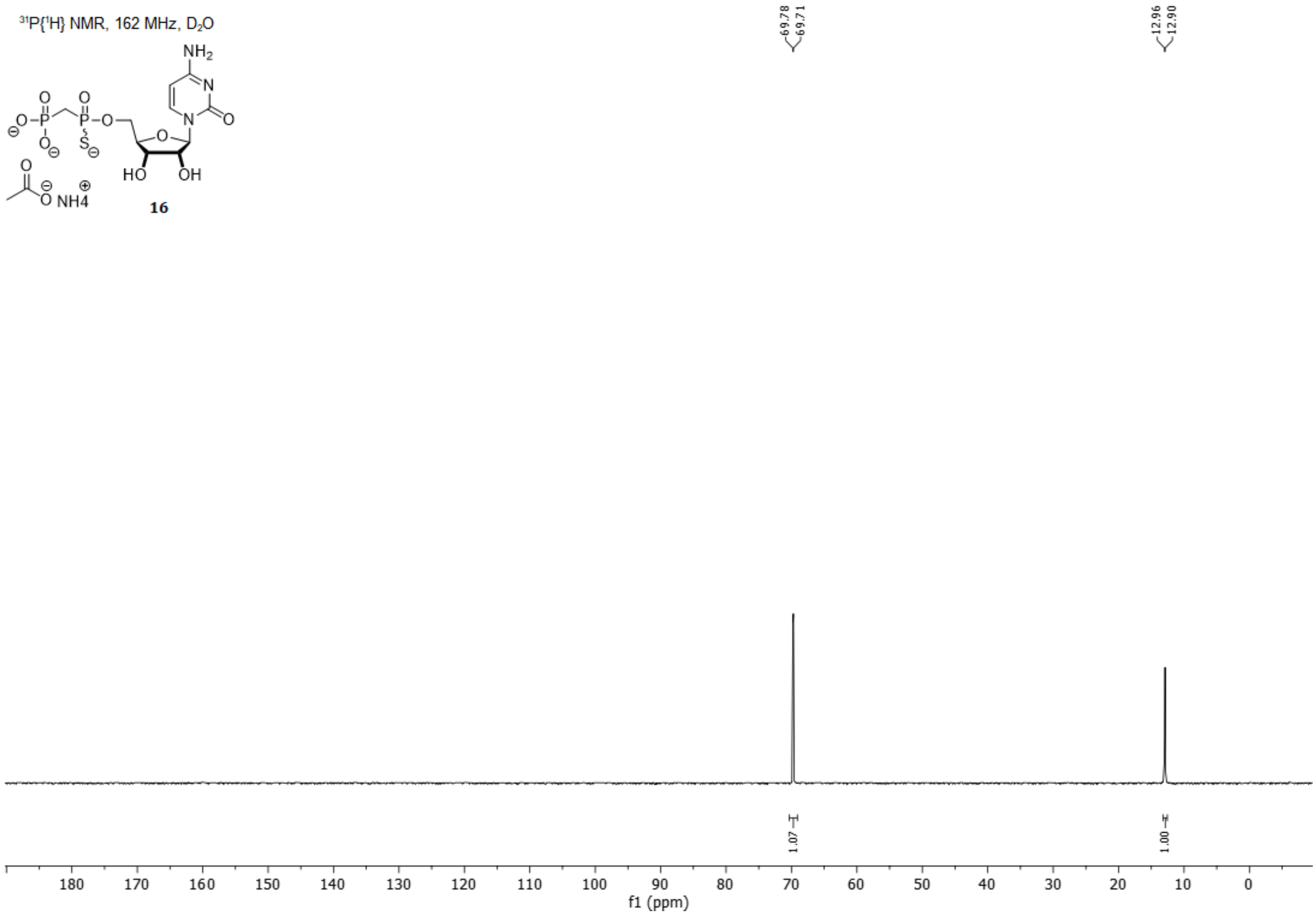
31P{1H} NMR, 162 MHz, D2O
69.78
69.71
12.96
12.90
NH2
N
O
S
HO
OH
NH4
16
1.07
1.00
180 170 160 150 140 130 120 110 100 90 80 70 60 50 40 30 20 10 0
f1 (ppm)

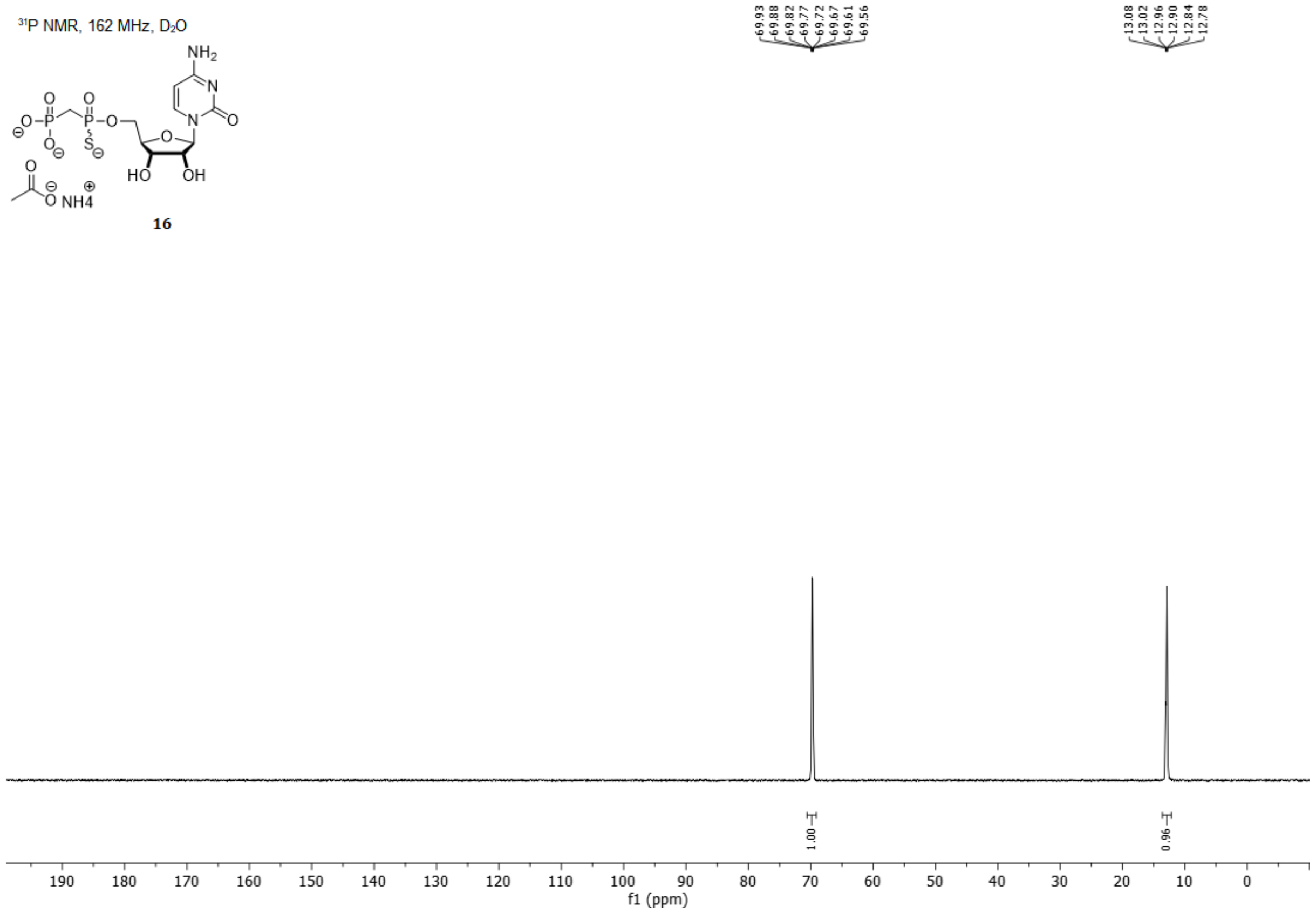
31P NMR, 162 MHz, D2O
NH2
N
O
O
P
P
O
O
S
O
HO
OH
O
O
NH4
69.93
69.88
69.82
69.77
69.72
69.67
69.61
69.56
13.08
13.02
12.96
12.90
12.84
12.78
1.00
0.96
190 180 170 160 150 140 130 120 110 100 90 80 70 60 50 40 30 20 10 0
f1 (ppm)

16

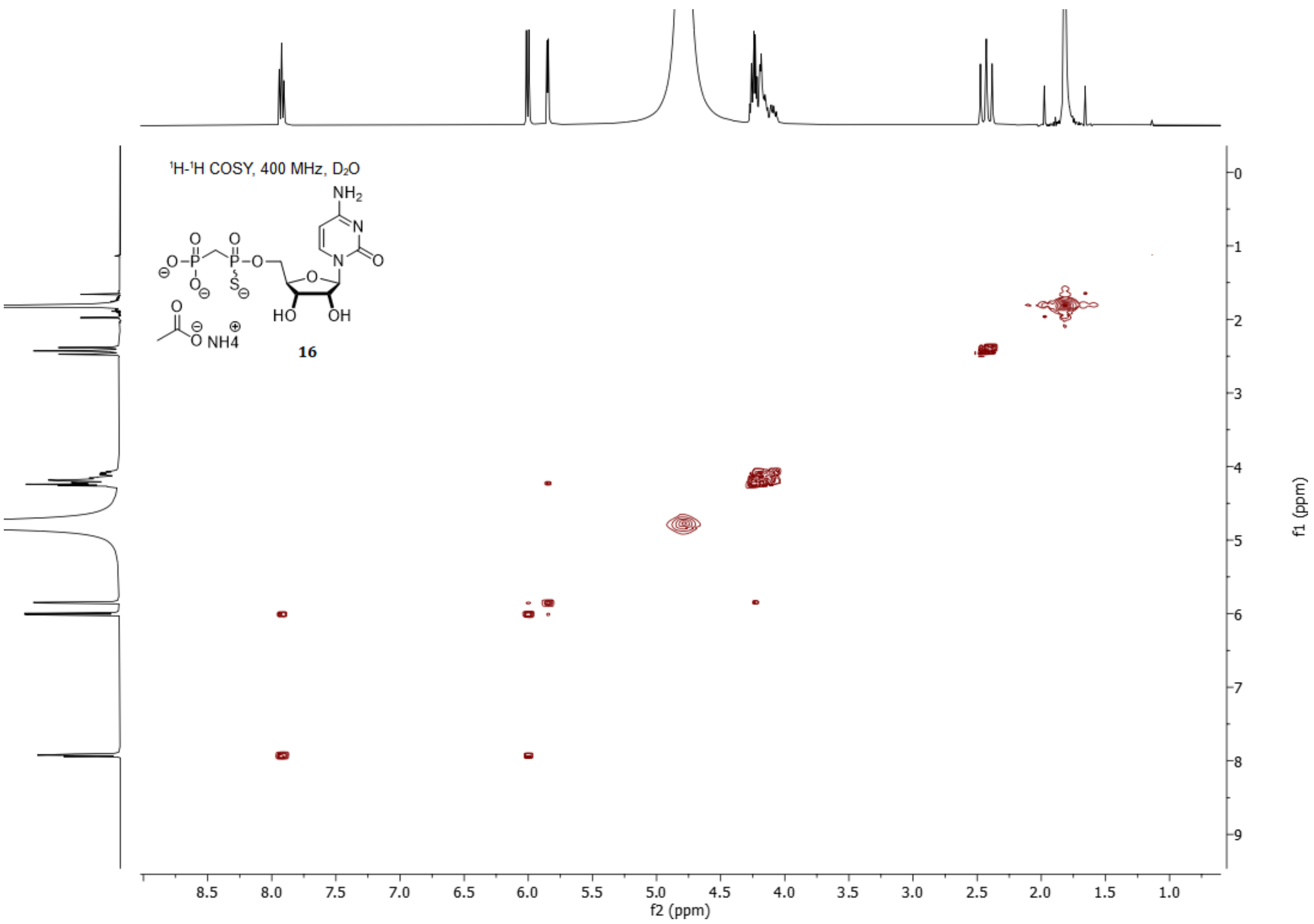
1H-1H COSY, 400 MHz, D2O
NH2
N
O
O
P
P
O
O
S
O
HO
OH
O
O
NH4
8.5 8.0 7.5 7.0 6.5 6.0 5.5 5.0 4.5 4.0 3.5 3.0 2.5 2.0 1.5 1.0
f2 (ppm)
0 1 2 3 4 5 6 7 8 9
f1 (ppm)

16

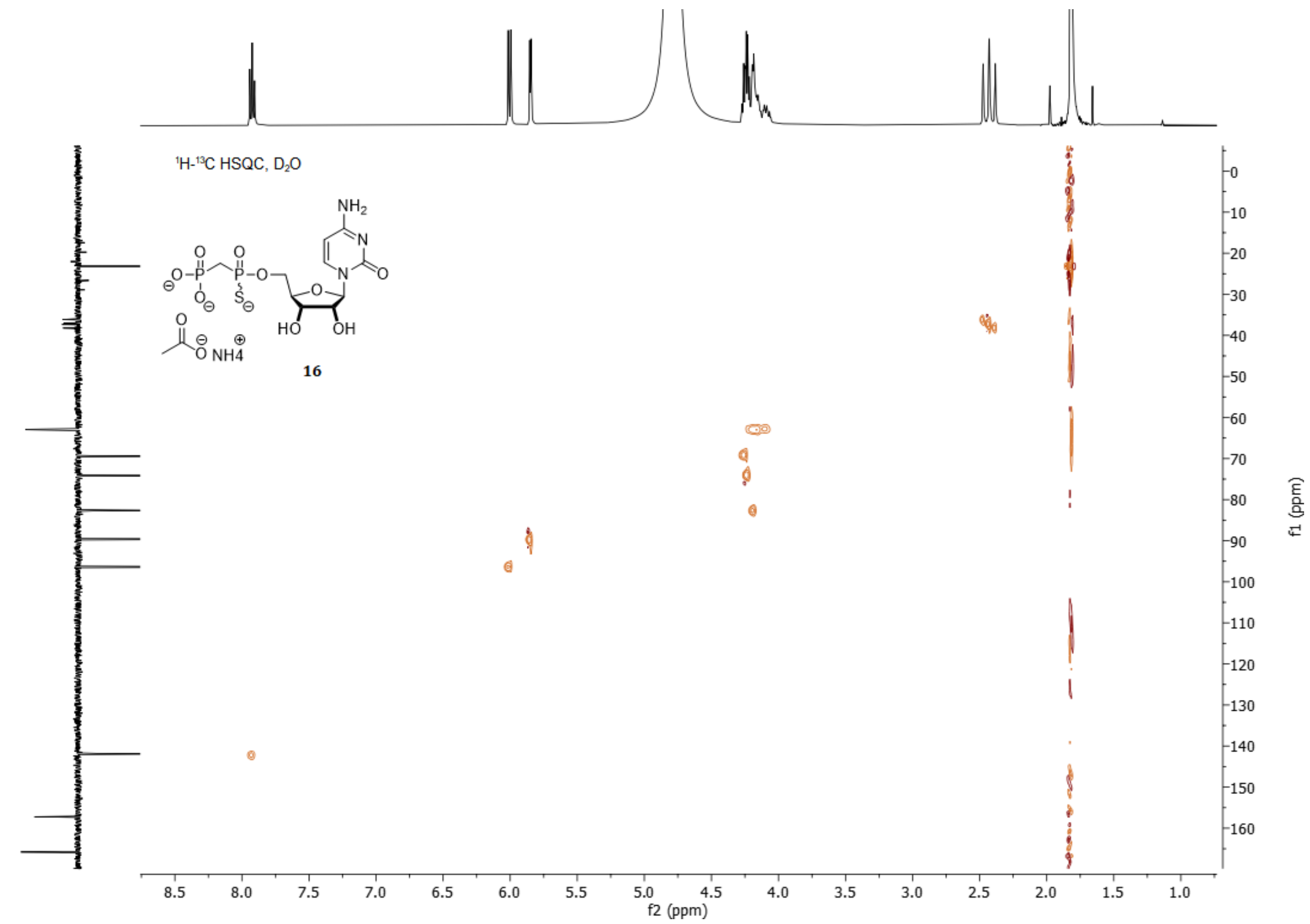

^{1}H-^{13}C HSQC, D_2O
16
f2 (ppm)
f1 (ppm)

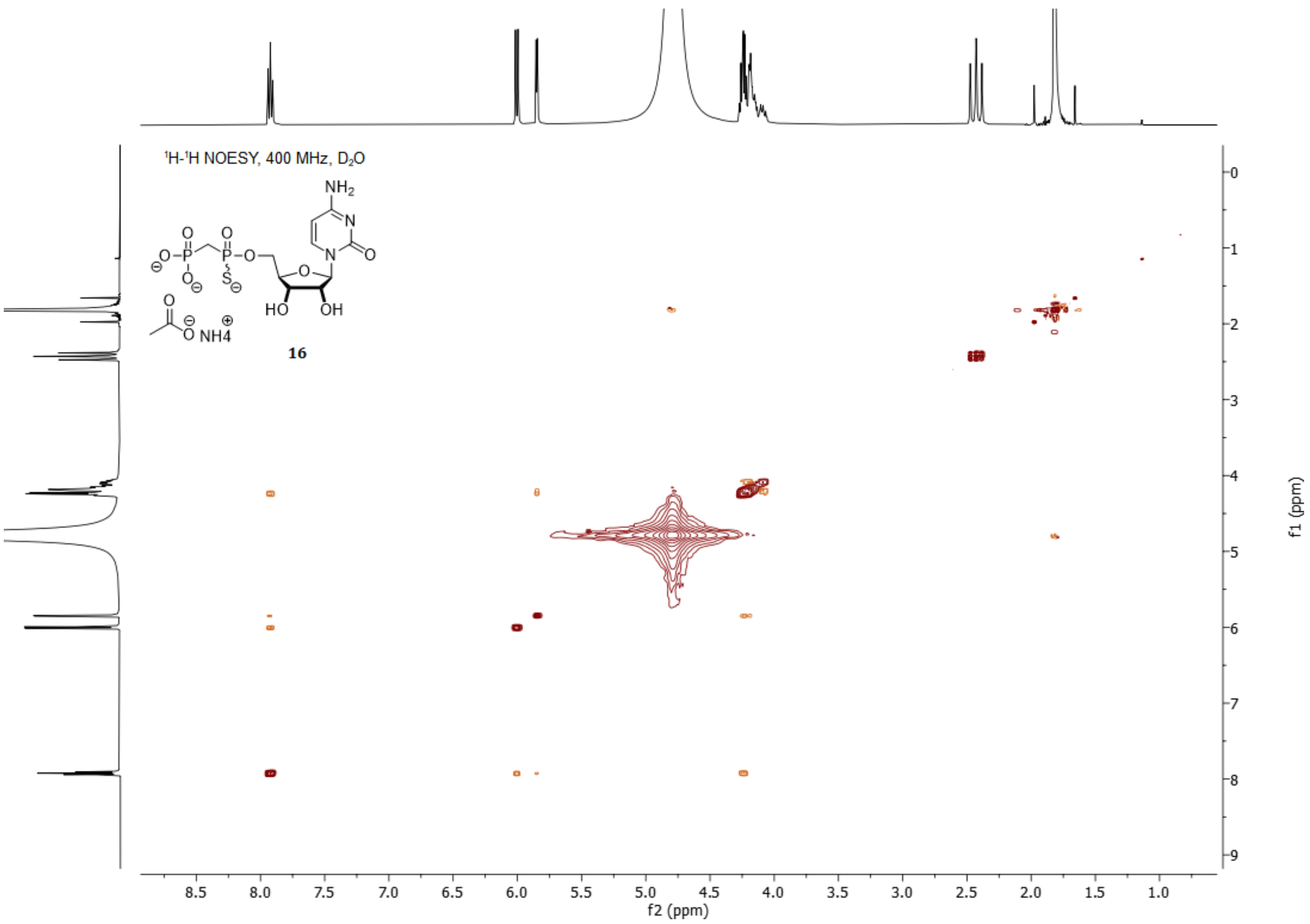

^{1}H-^{1}H NOESY, 400 MHz, D_2O
16
f2 (ppm)
f1 (ppm)

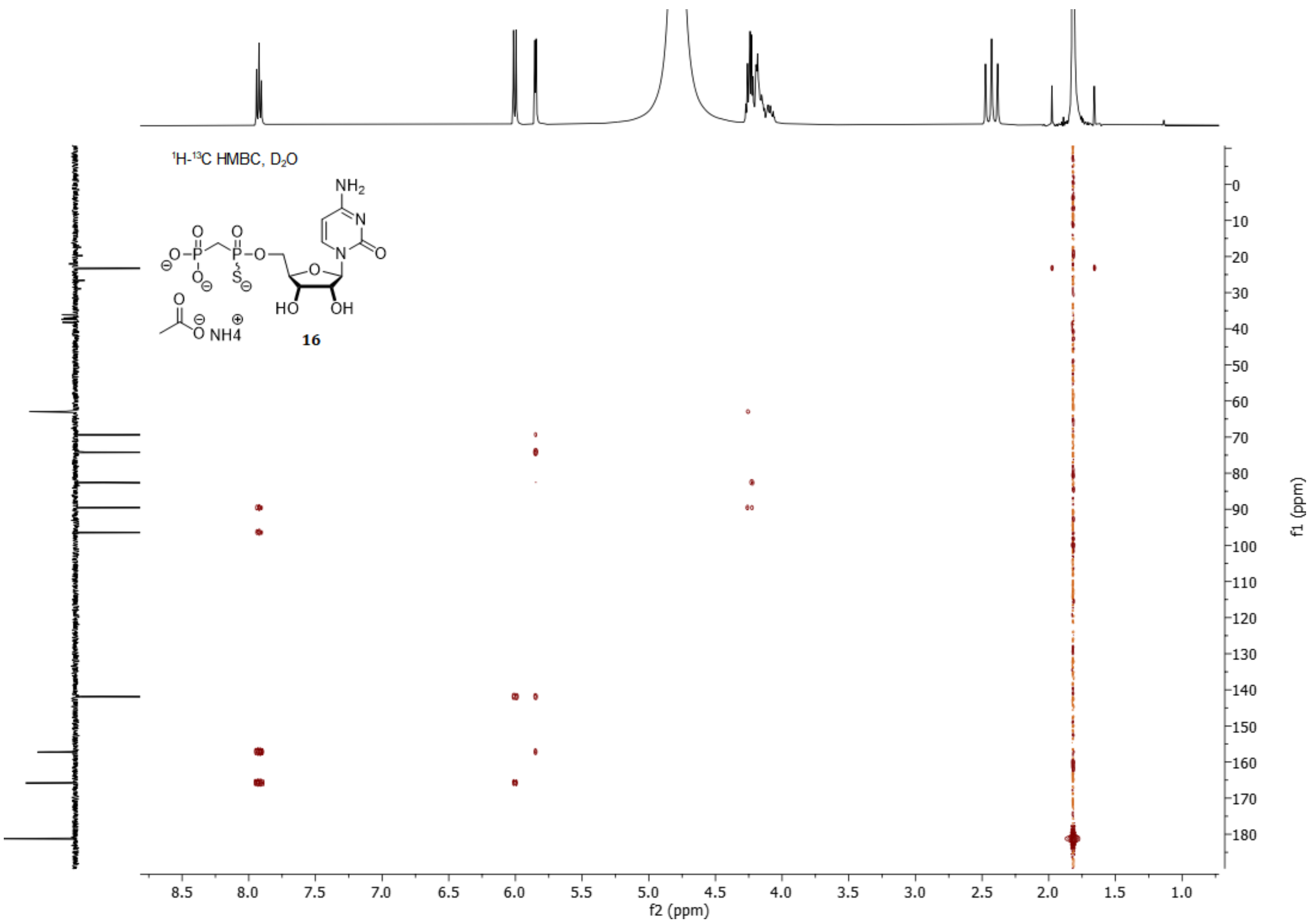

¹H-¹³C HMBC, D₂O
16
f2 (ppm)
f1 (ppm)

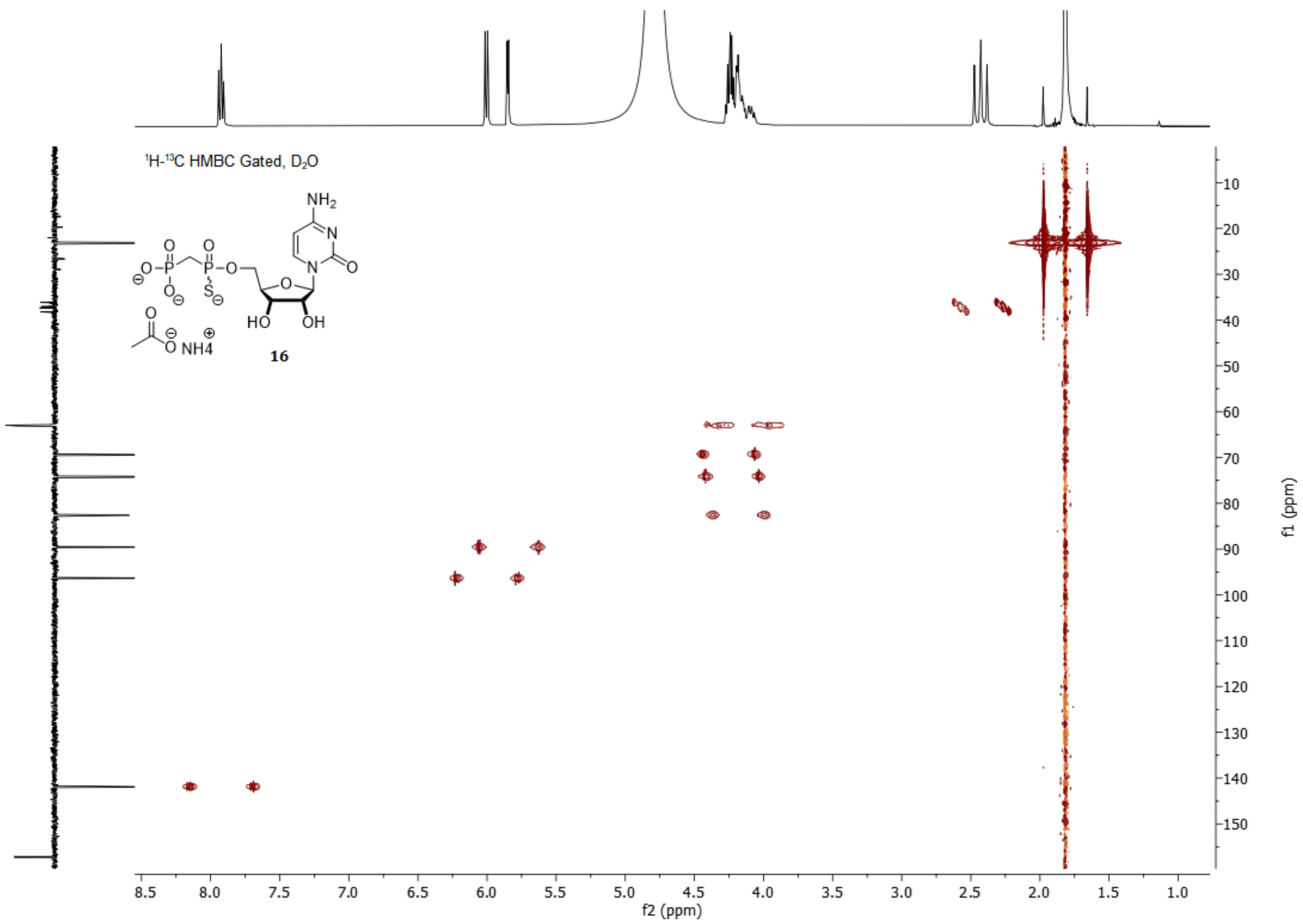

¹H-¹³C HMBC Gated, D₂O
16
f2 (ppm)
f1 (ppm)

me-GDP-αS tetraester 18

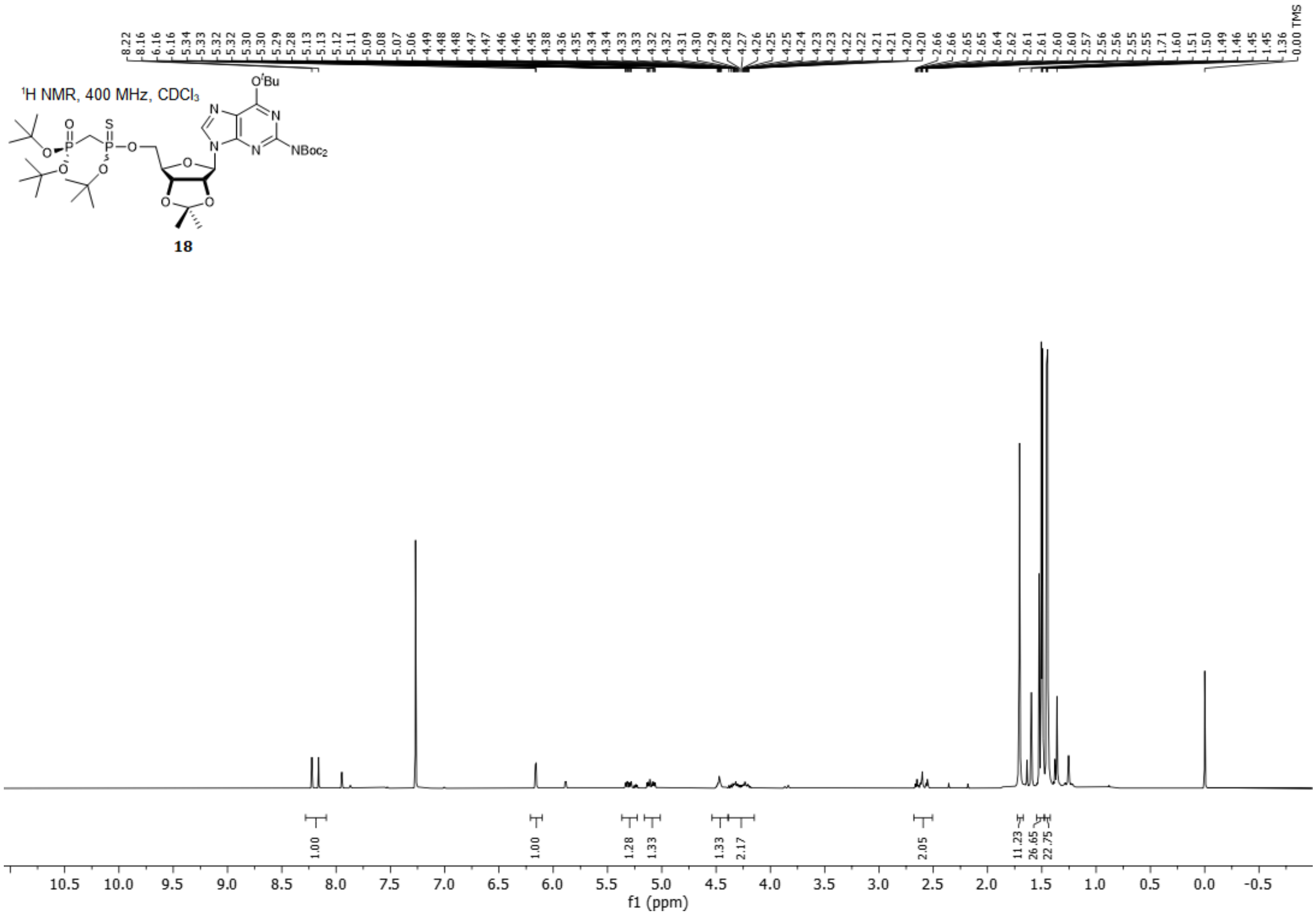

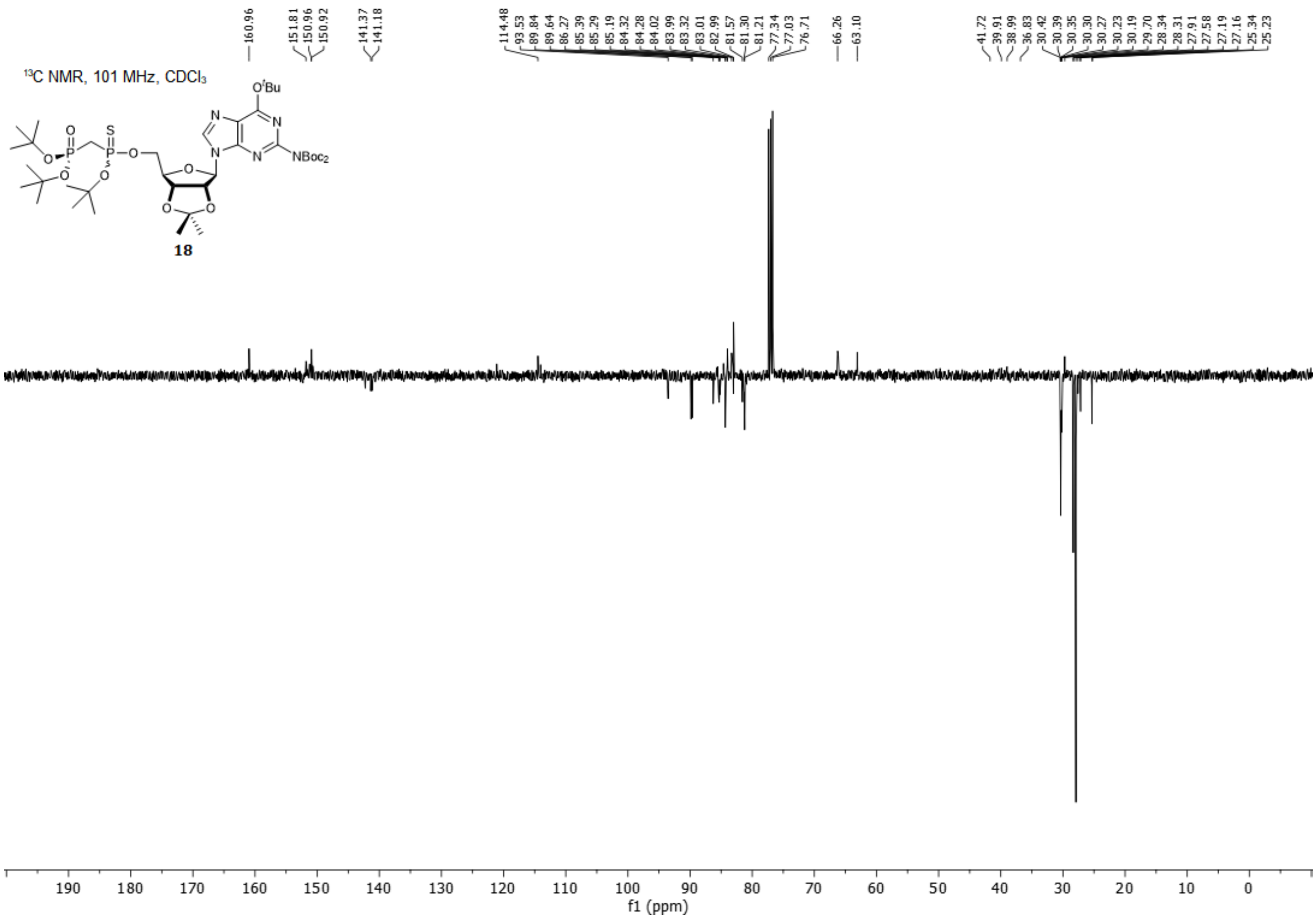

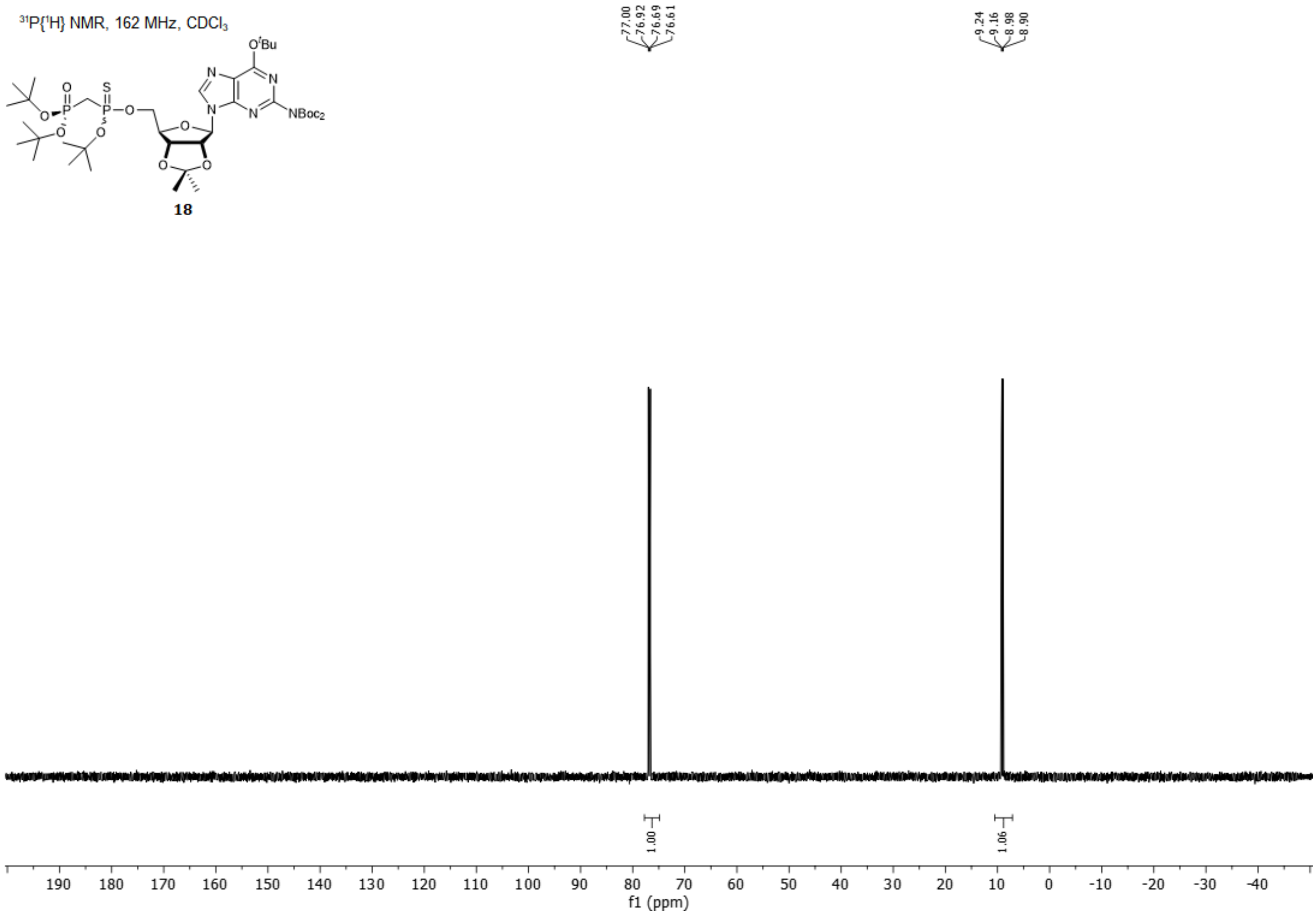

$^{31}P\{^{1}H\}$ NMR, 162 MHz, $CDCl_3$
O^{t}Bu
NBoc$_2$
18
77.00
76.92
76.69
76.61
9.24
9.16
8.98
8.90
1.00
1.06
190 180 170 160 150 140 130 120 110 100 90 80 70 60 50 40 30 20 10 0 -10 -20 -30 -40
f1 (ppm)

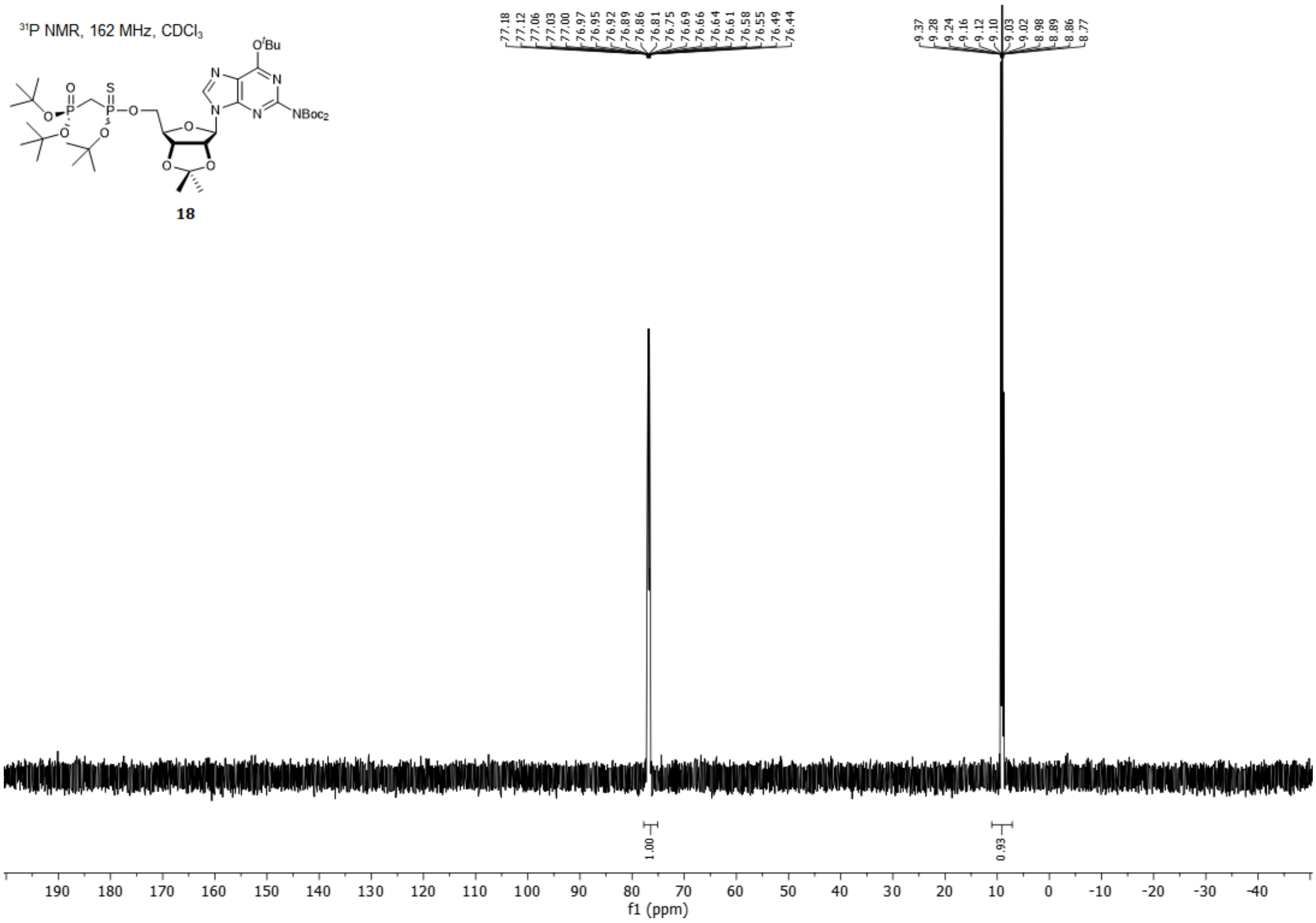

^{31}P NMR, 162 MHz, $CDCl_3$
O^{t}Bu
NBoc$_2$
18
77.18
77.12
77.06
77.03
77.00
76.97
76.95
76.92
76.89
76.86
76.81
76.75
76.69
76.66
76.64
76.61
76.58
76.55
76.49
76.44
9.37
9.28
9.24
9.16
9.12
9.10
9.03
9.02
8.98
8.89
8.86
8.77
1.00
0.93
190 180 170 160 150 140 130 120 110 100 90 80 70 60 50 40 30 20 10 0 -10 -20 -30 -40
f1 (ppm)

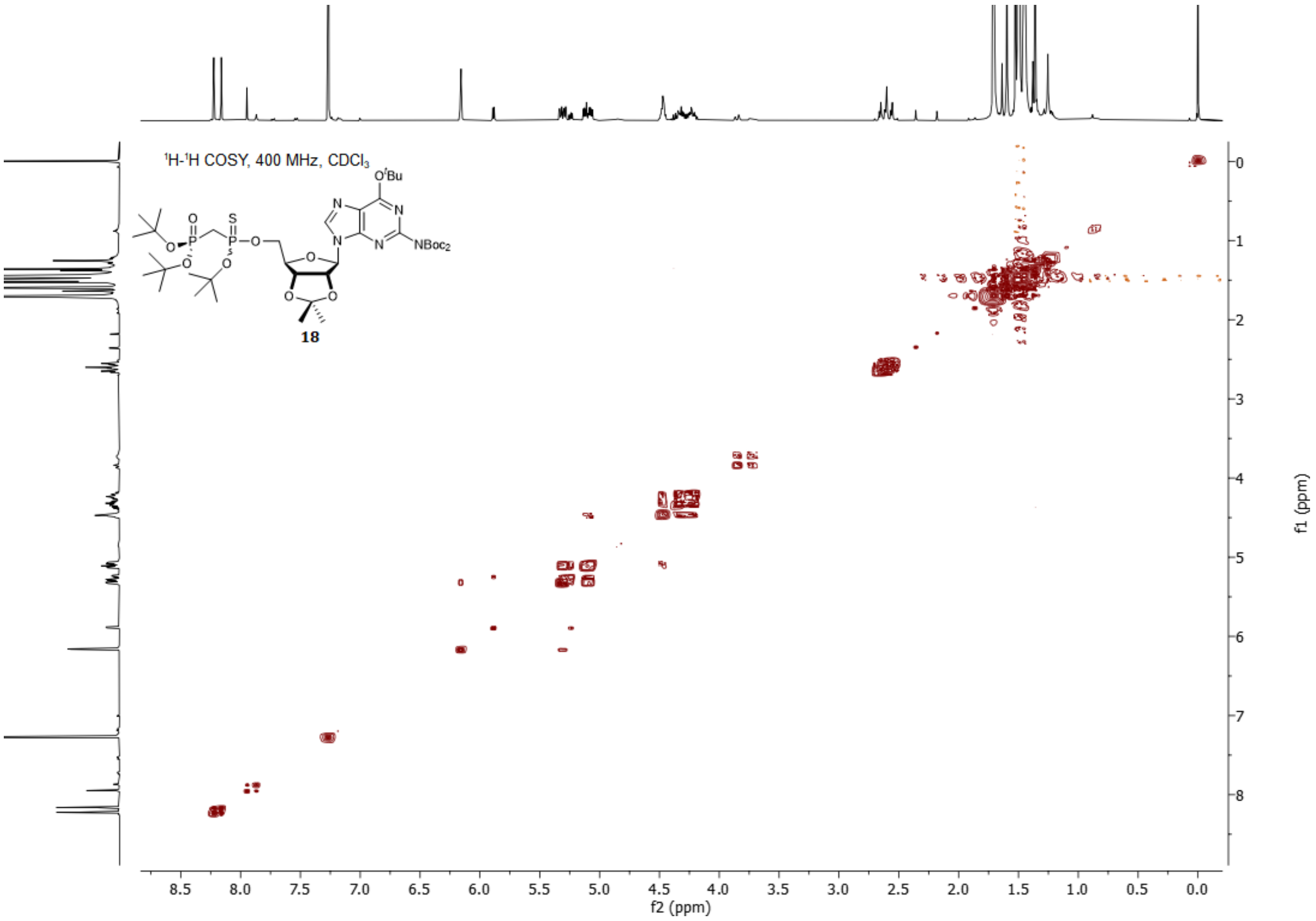

¹H-¹H COSY, 400 MHz, CDCl₃
O^tBu
NBoc₂
18
f2 (ppm)
f1 (ppm)

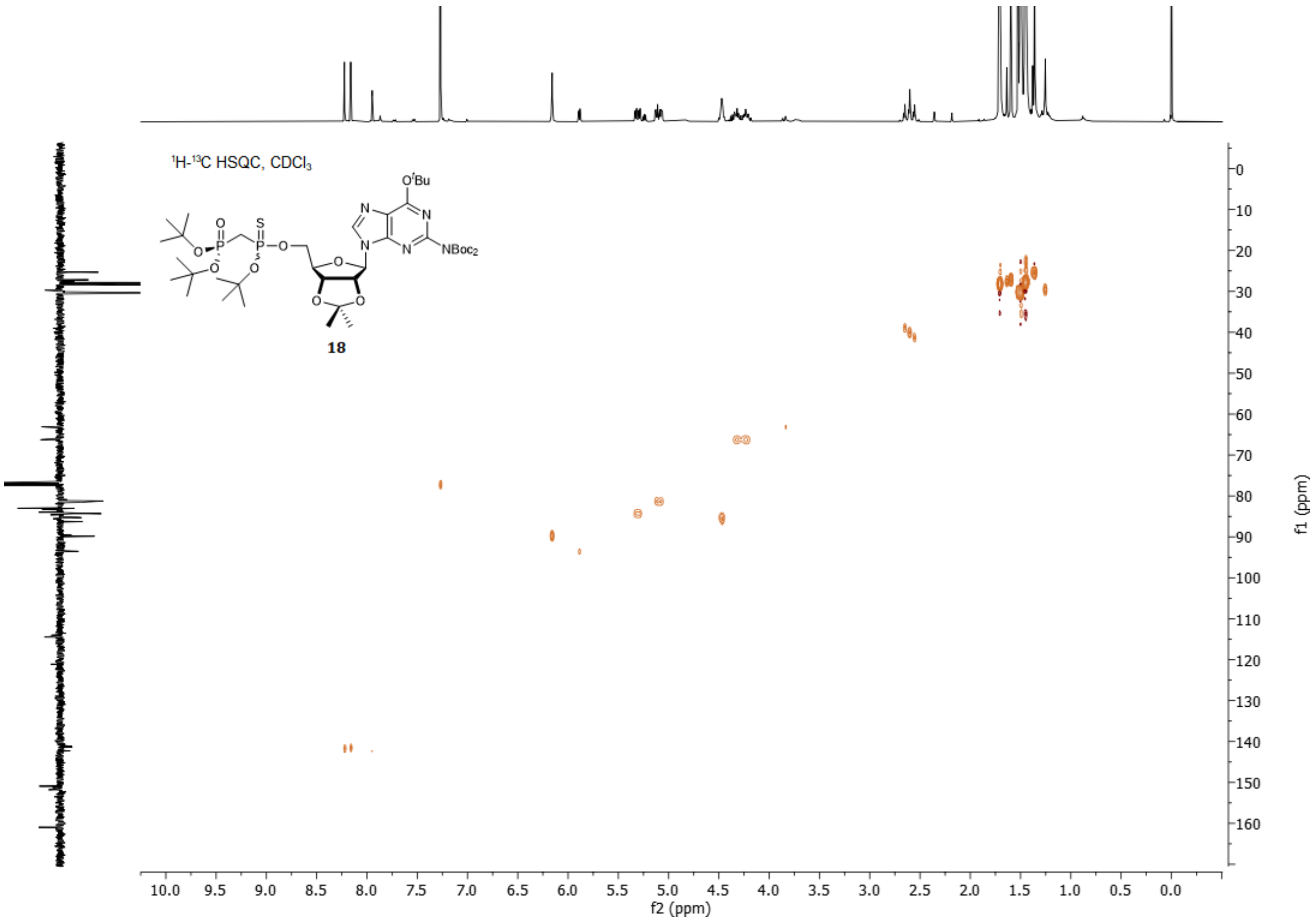

¹H-¹³C HSQC, CDCl₃
O^tBu
NBoc₂
18
f2 (ppm)
f1 (ppm)

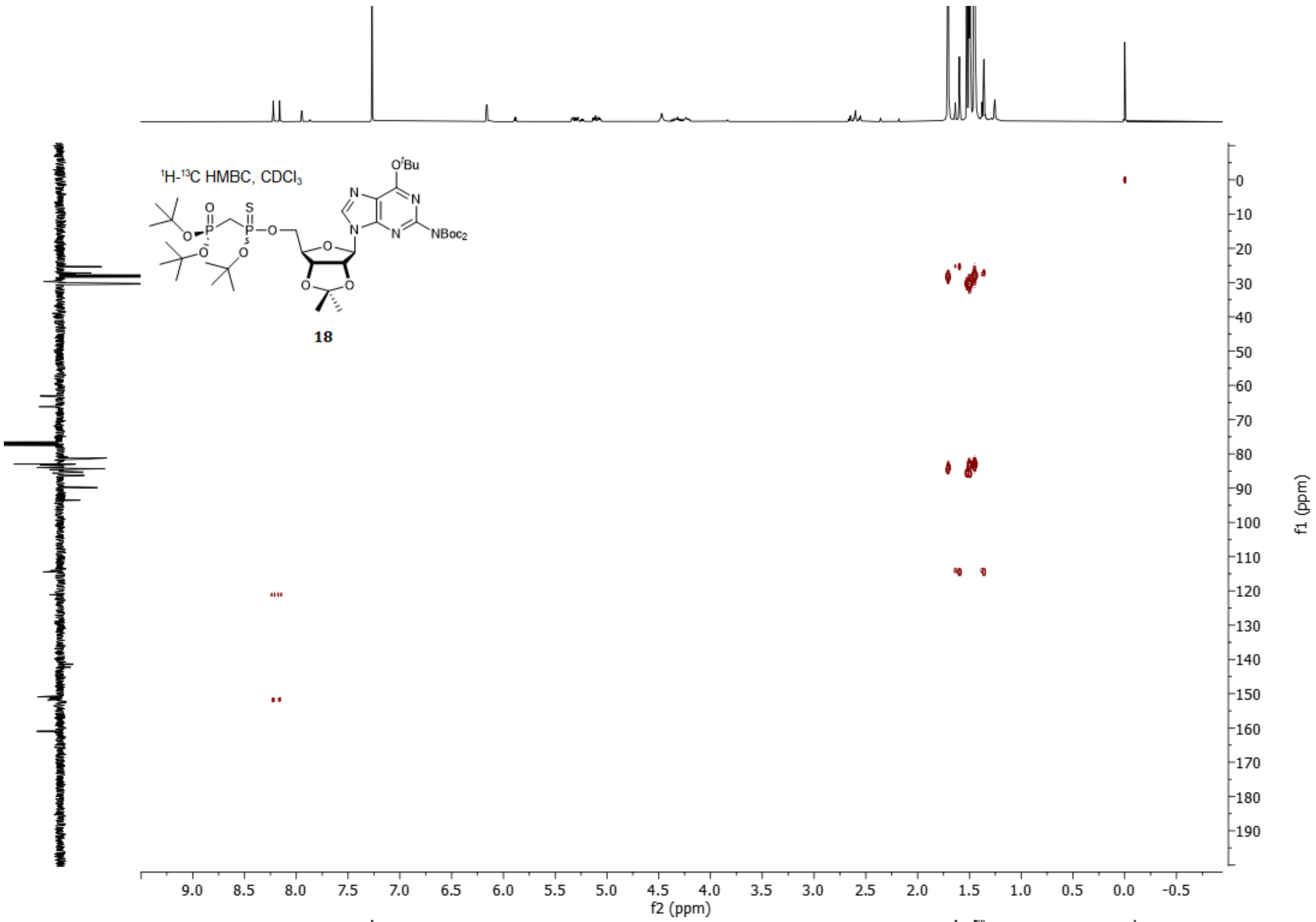
^{1}H-^{13}C HMBC, CDCl$_3$
O^{t}Bu
NBoc$_2$
18
f2 (ppm)
f1 (ppm)

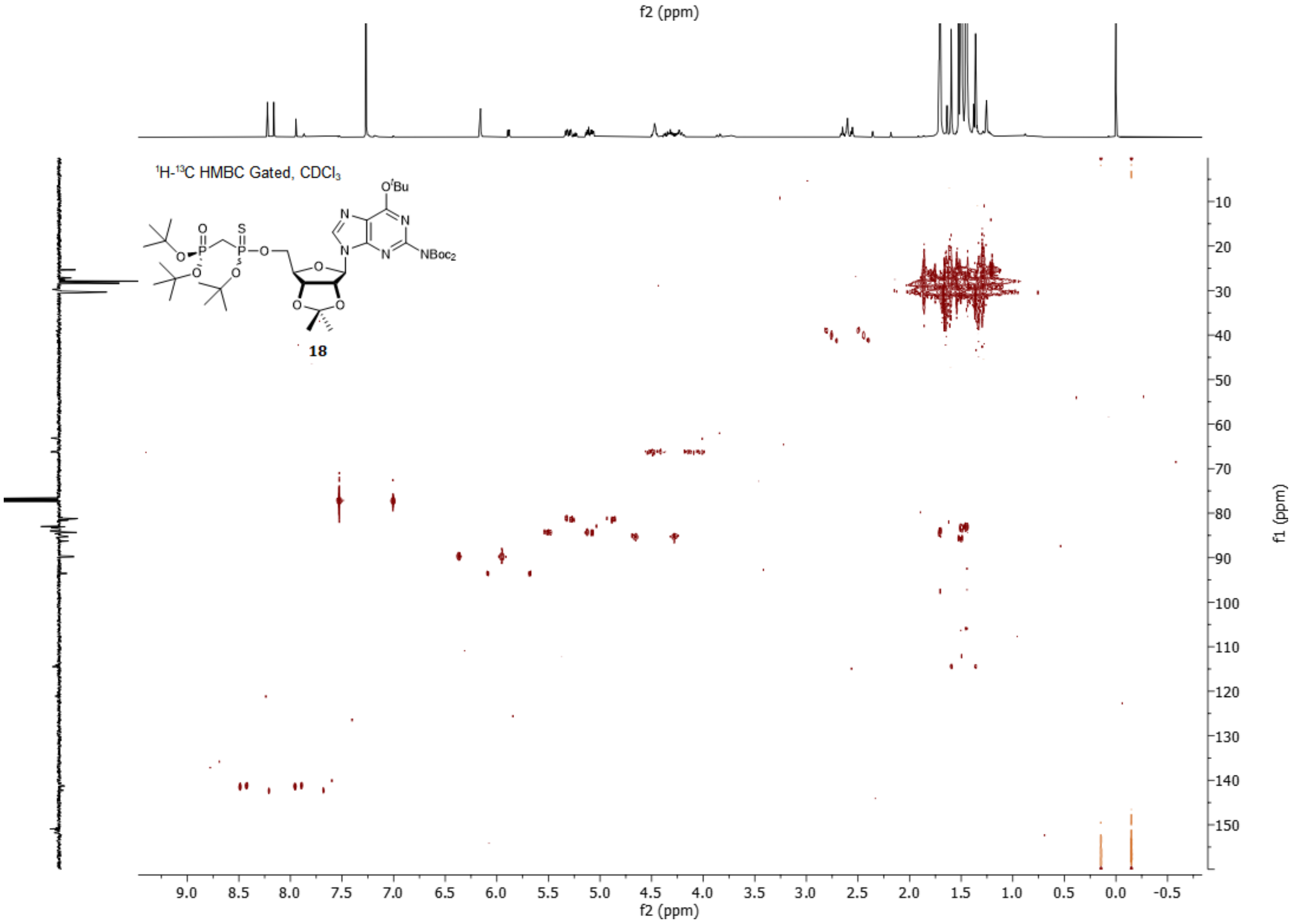
^{1}H-^{13}C HMBC Gated, CDCl$_3$
O^{t}Bu
NBoc$_2$
18
f2 (ppm)
f1 (ppm)

me-GDP-αS 19

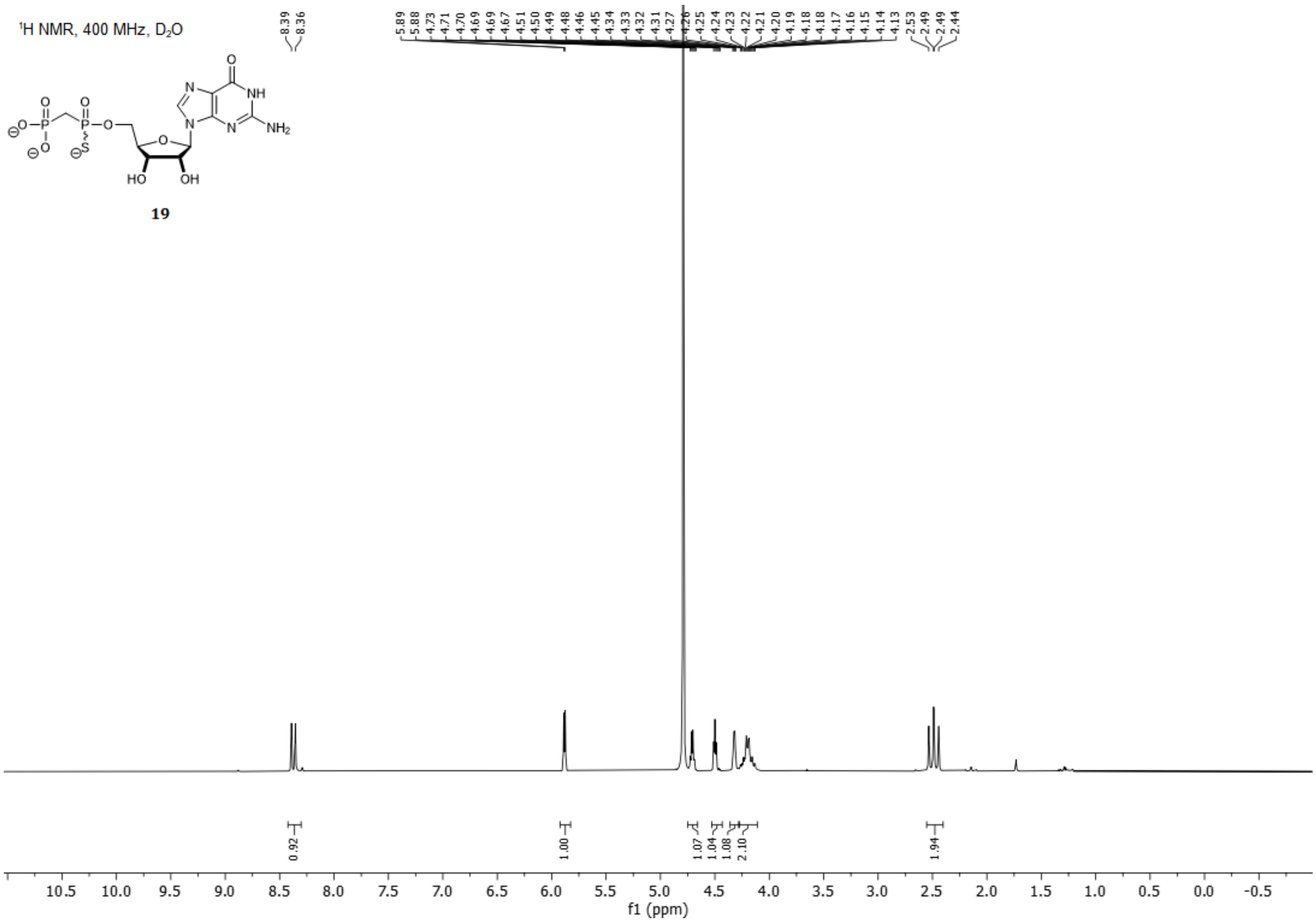

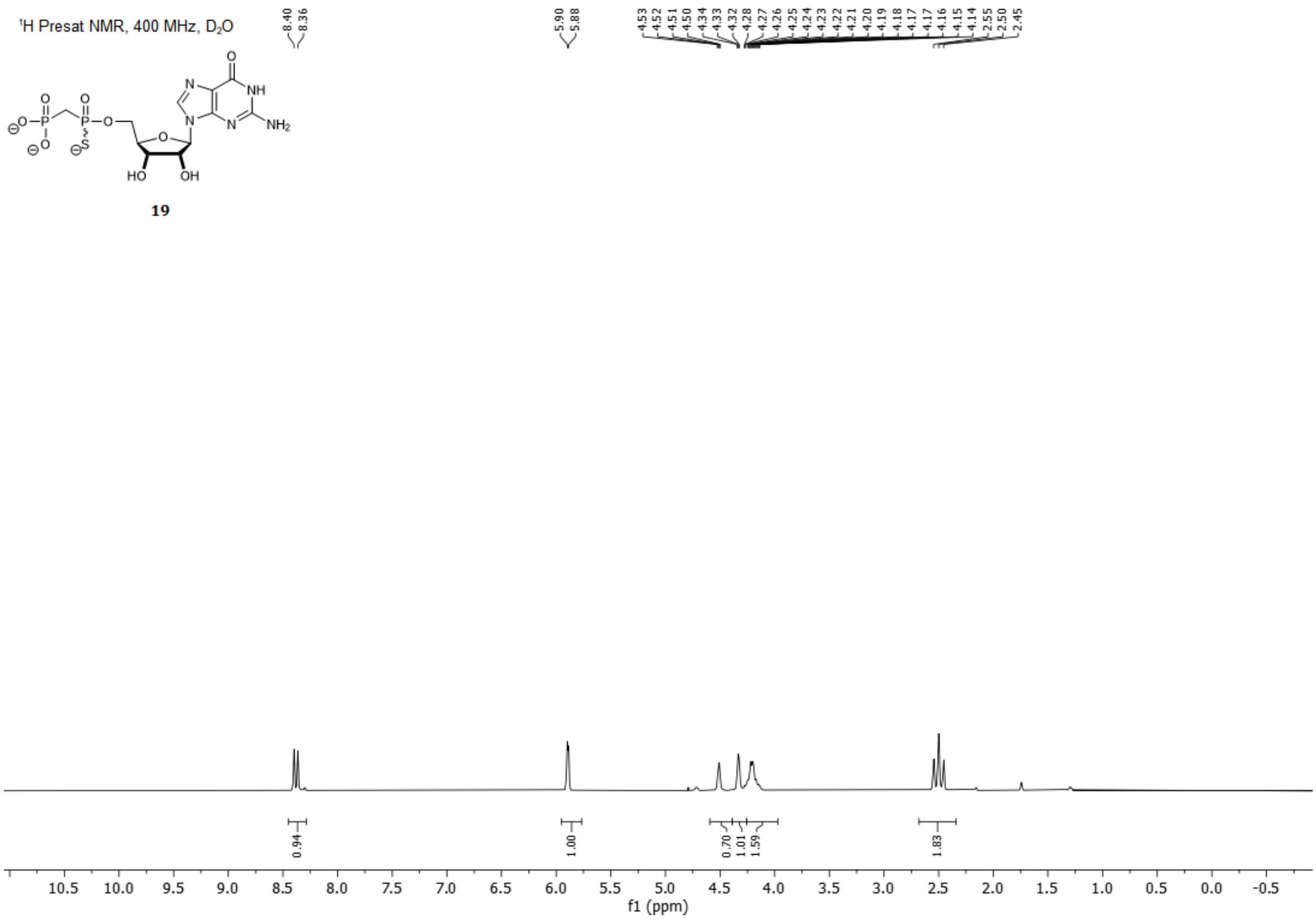

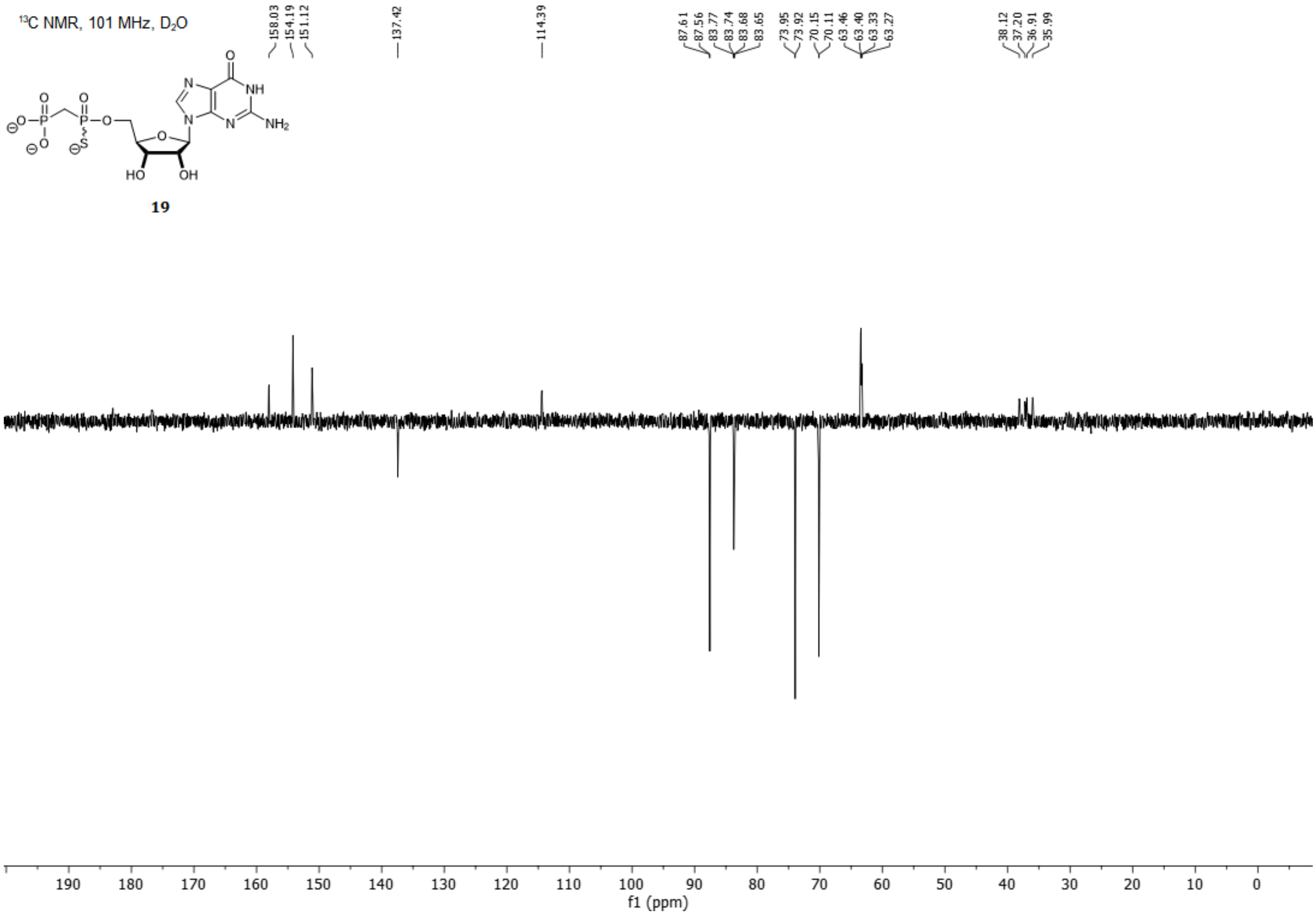
13C NMR, 101 MHz, D2O
19
158.03
154.19
151.12
137.42
114.39
87.61
87.56
83.77
83.74
83.68
83.65
73.95
73.92
70.15
70.11
63.46
63.40
63.33
63.27
38.12
37.20
36.91
35.99
f1 (ppm)

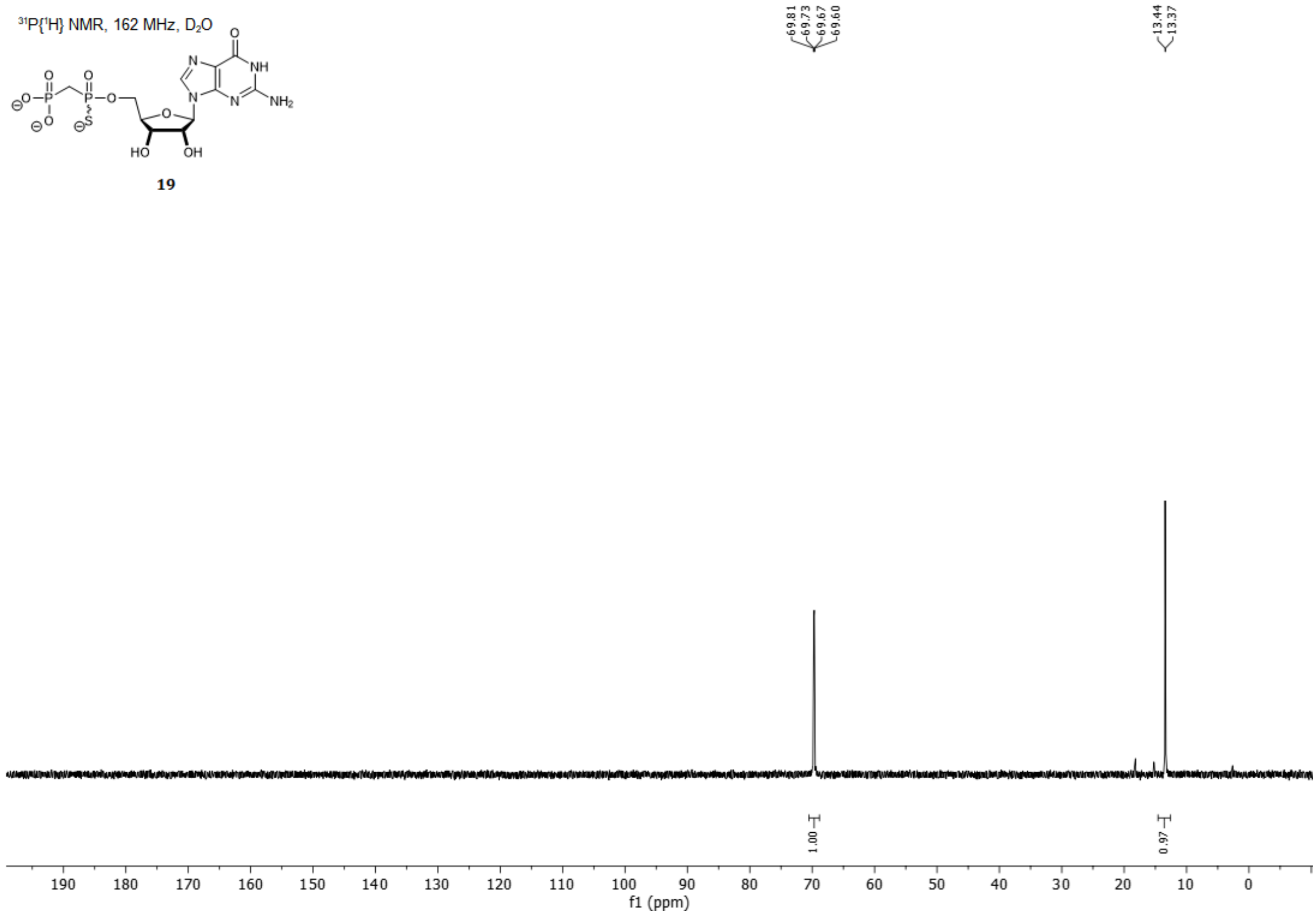
31P{1H} NMR, 162 MHz, D2O
19
69.81
69.73
69.67
69.60
13.44
13.37
1.00
0.97
f1 (ppm)

^{31}P NMR, 202 MHz, D_2O

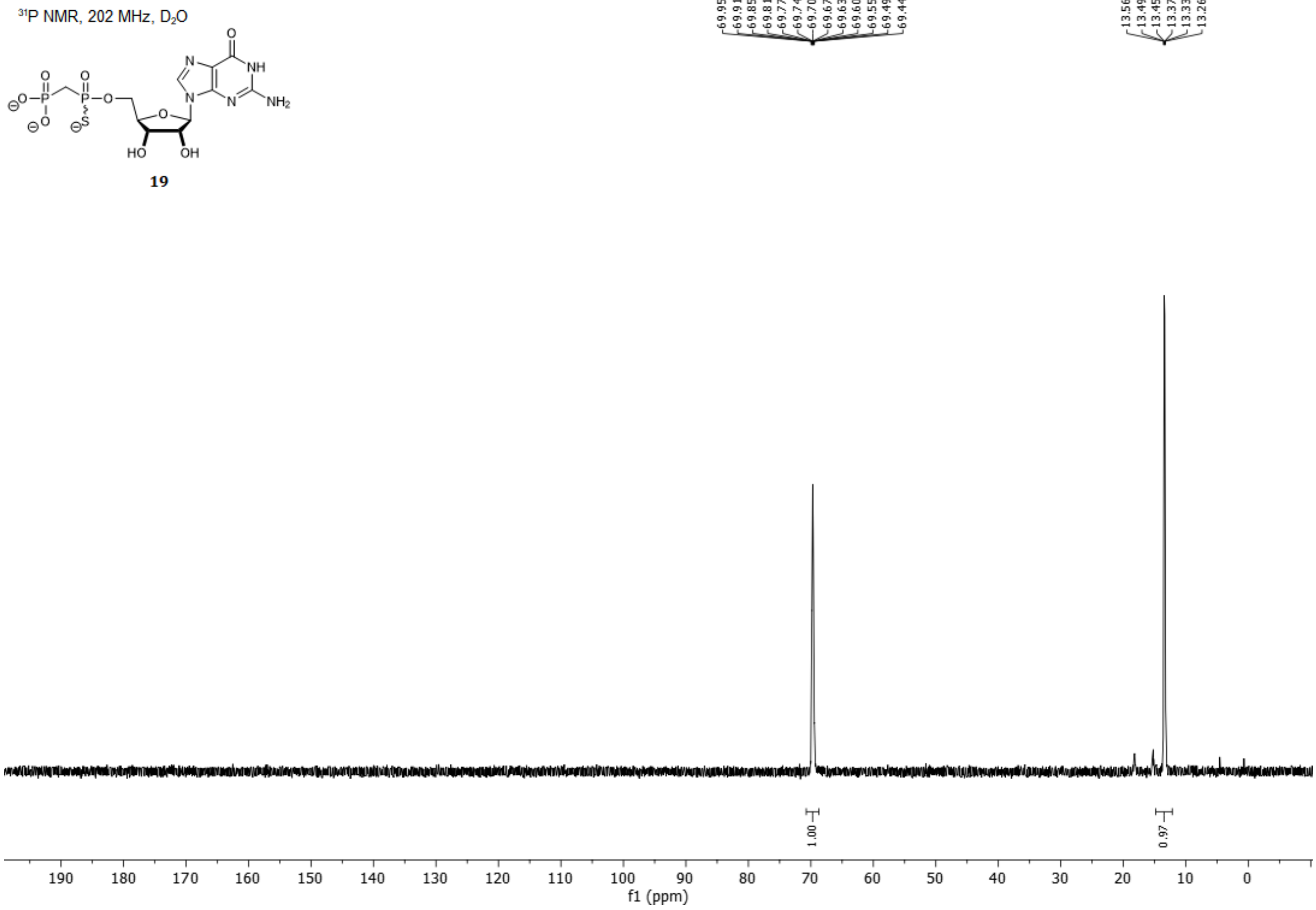

19

^{31}P NMR, 202 MHz, D_2O

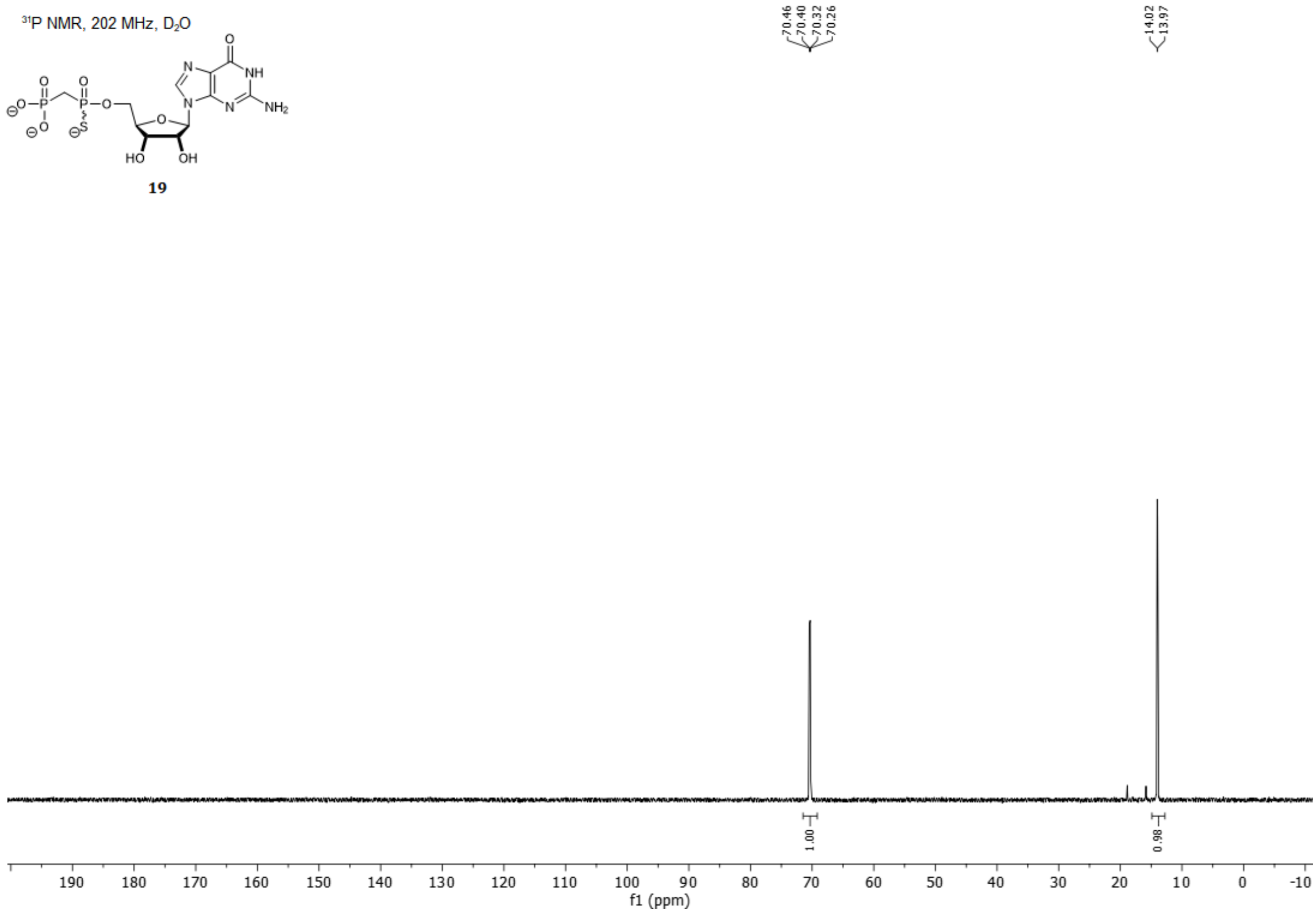

19

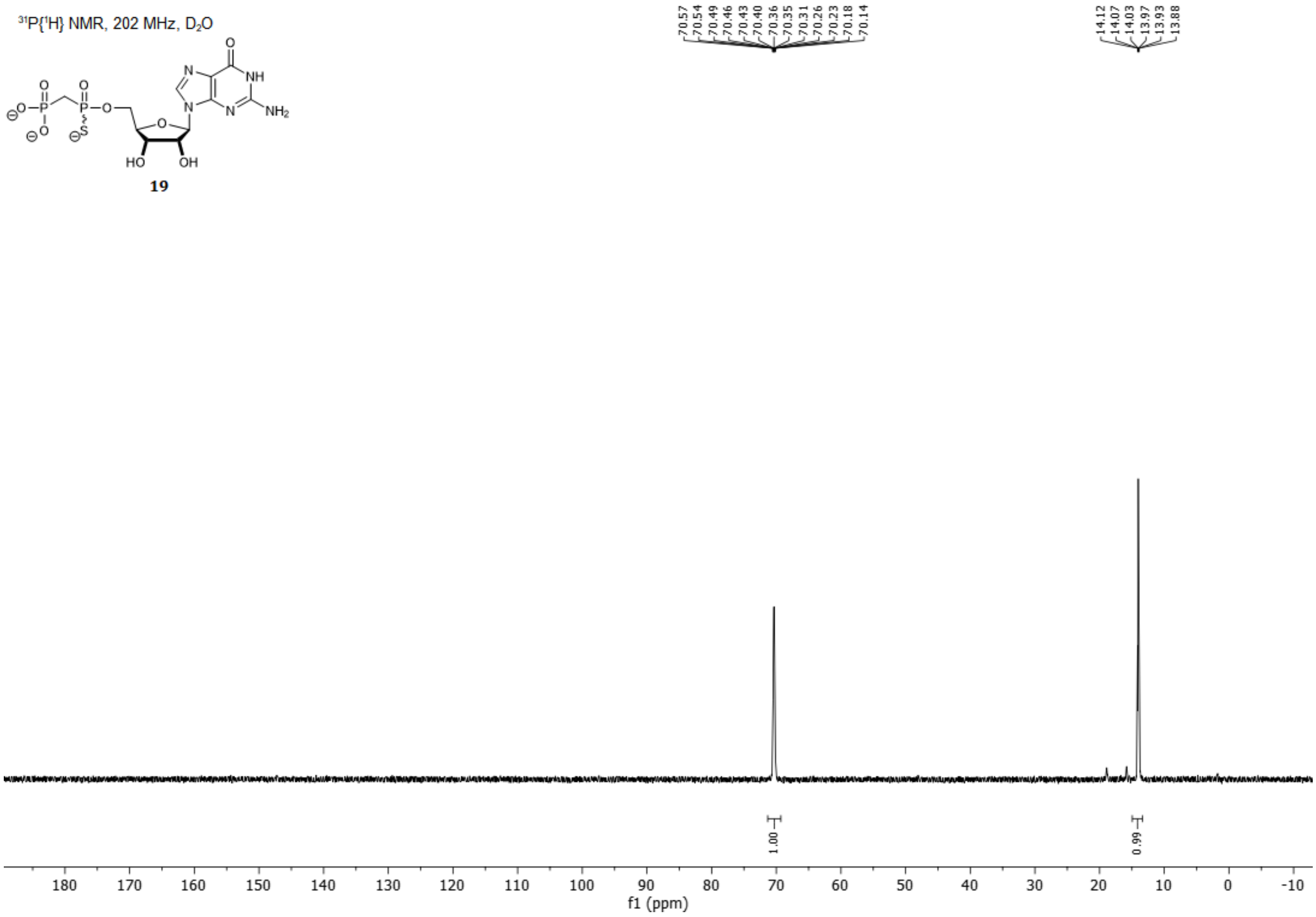

$^{31}P\{^{1}H\}$ NMR, 202 MHz, D_2O
19
70.57 70.54 70.49 70.46 70.43 70.40 70.36 70.35 70.31 70.26 70.23 70.18 70.14
14.12 14.07 14.03 13.97 13.93 13.88
1.00
0.99
f1 (ppm)

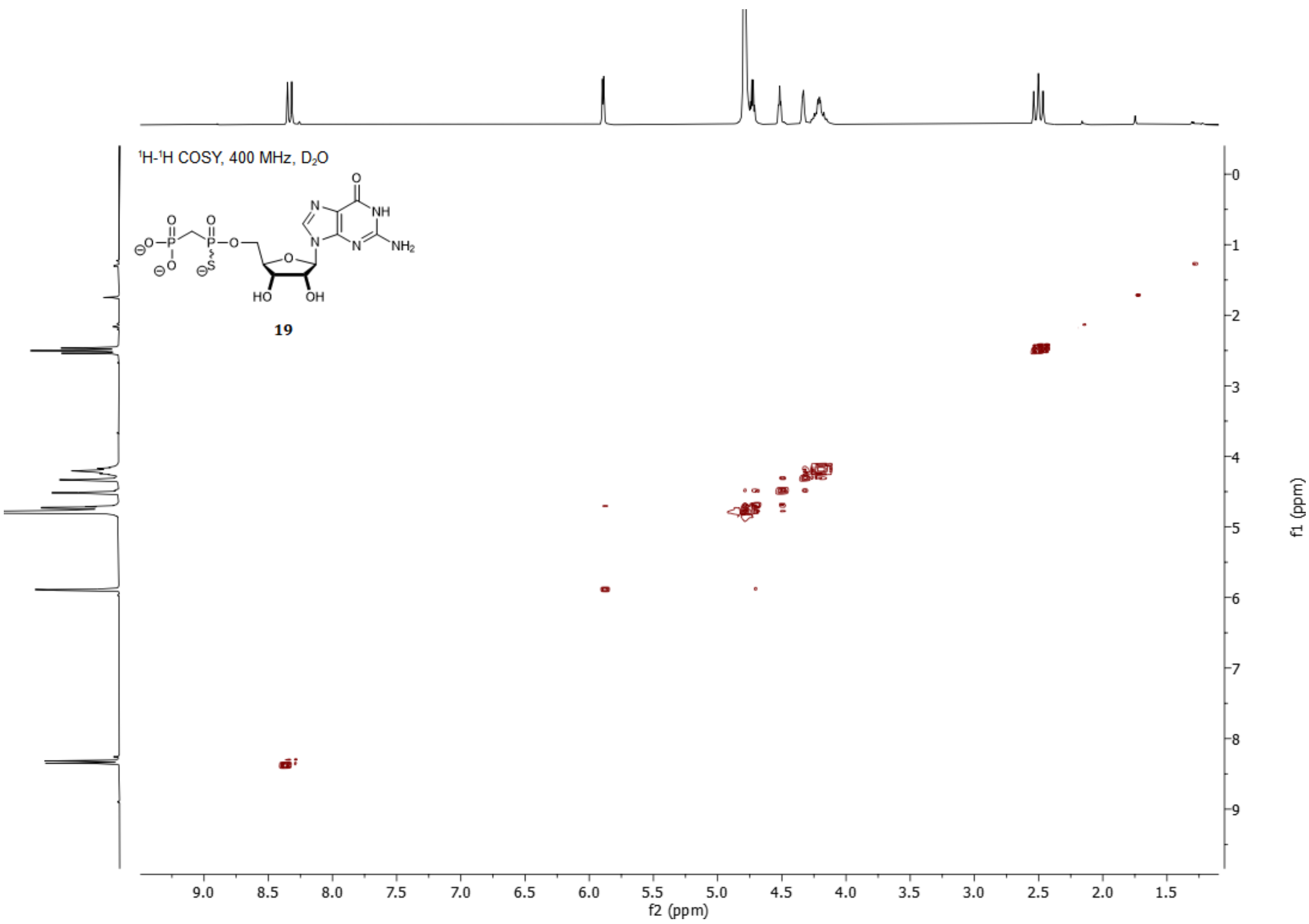

^{1}H-^{1}H COSY, 400 MHz, D_2O
19
f2 (ppm)
f1 (ppm)

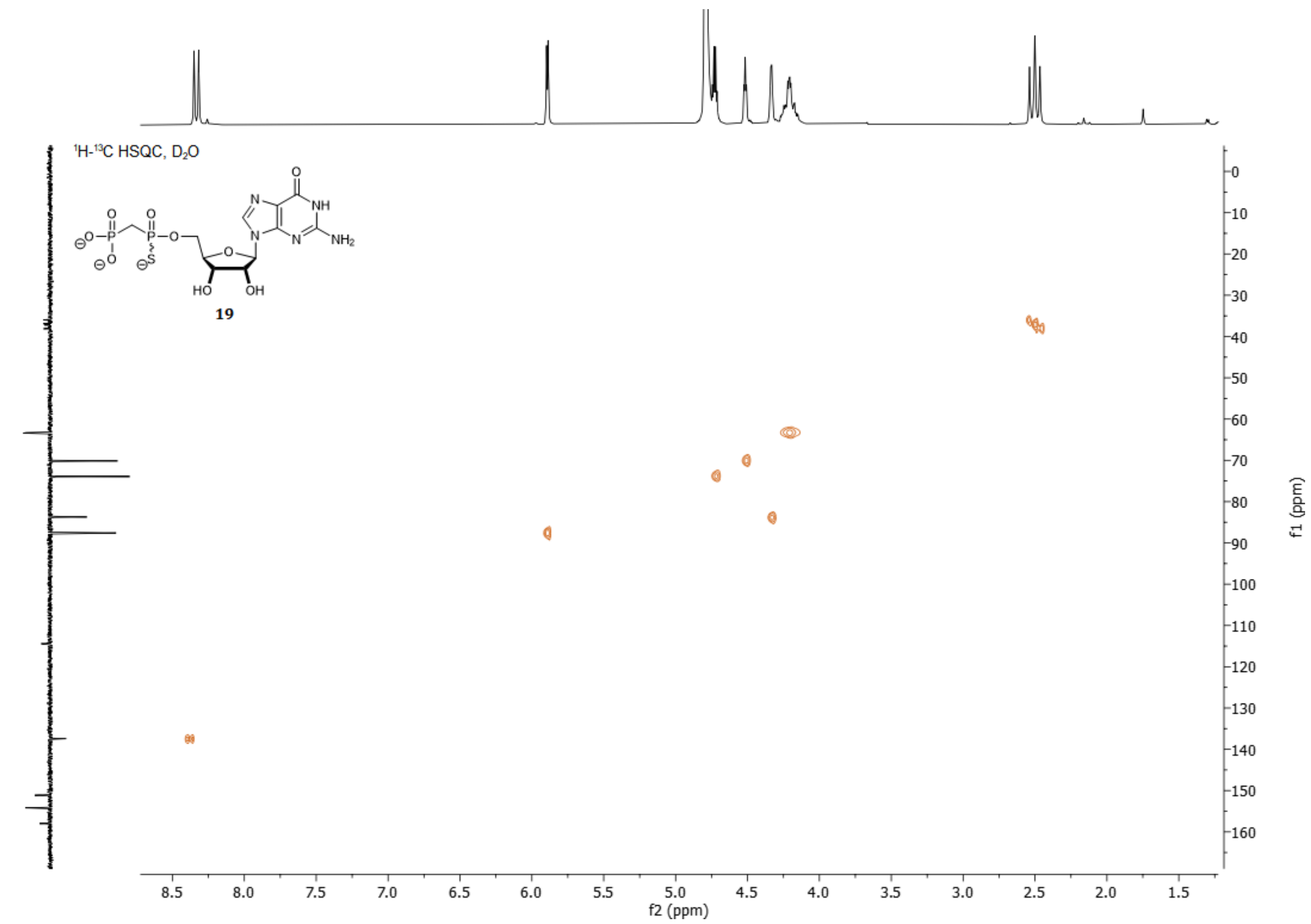
1H-13C HSQC, D2O
19
f2 (ppm)
f1 (ppm)

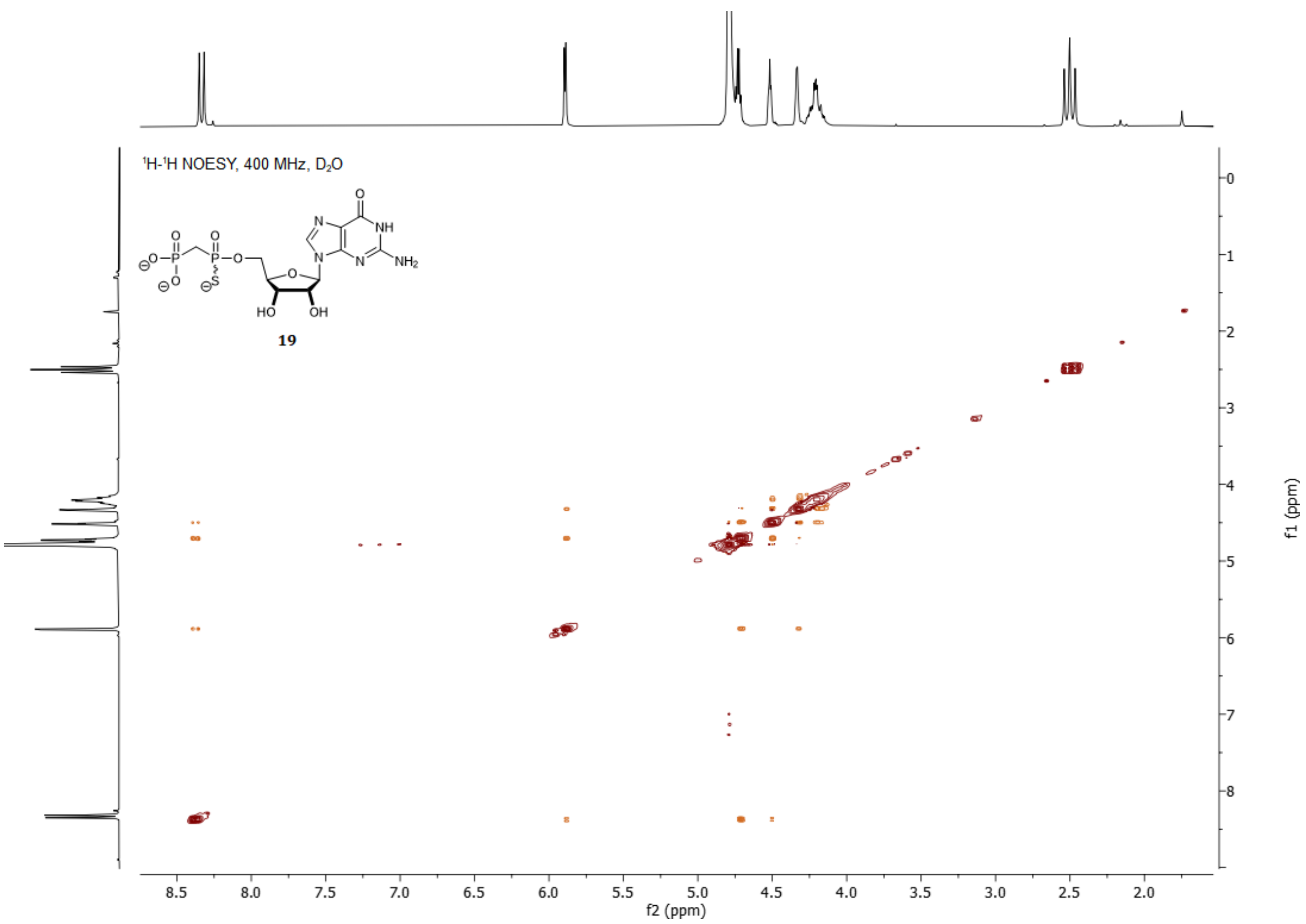
1H-1H NOESY, 400 MHz, D2O
19
f2 (ppm)
f1 (ppm)

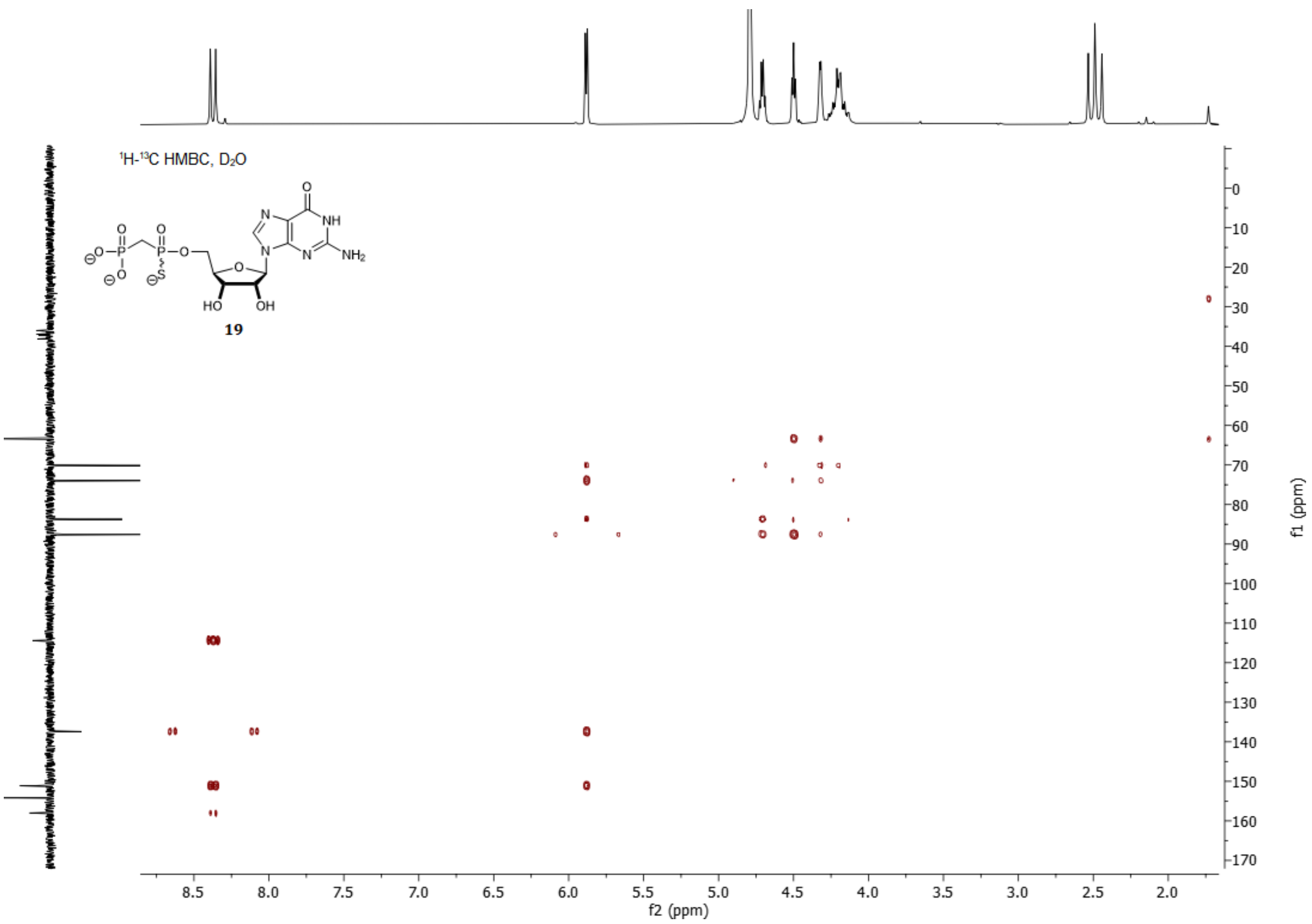

^{1}H-^{13}C HMBC, D$_2$O
19
f2 (ppm)
f1 (ppm)

NMR Spectra for Ribitol Building Blocks

5-*O*-*tert*-butyldiphenylsilyl-D-ribitol S1

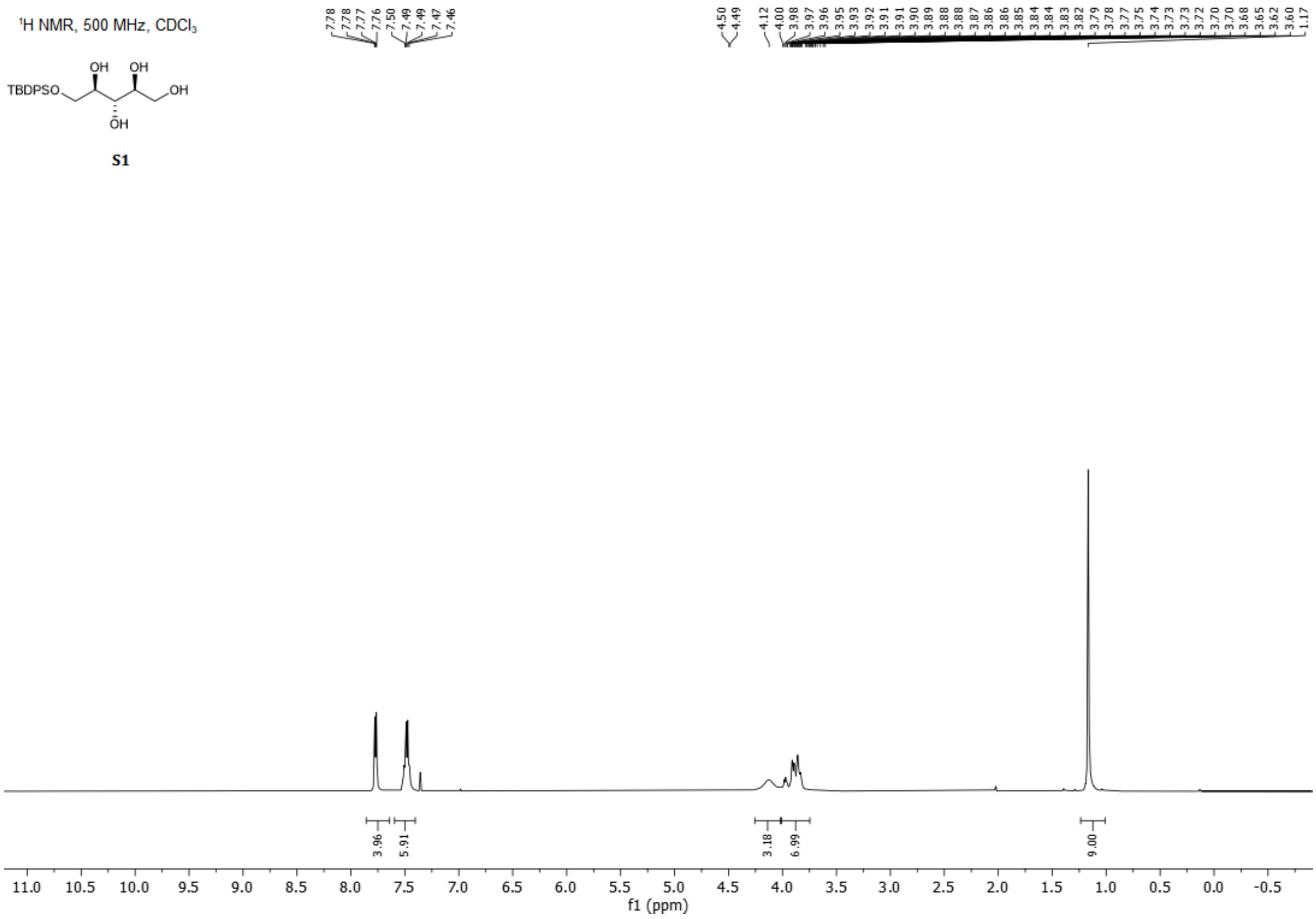

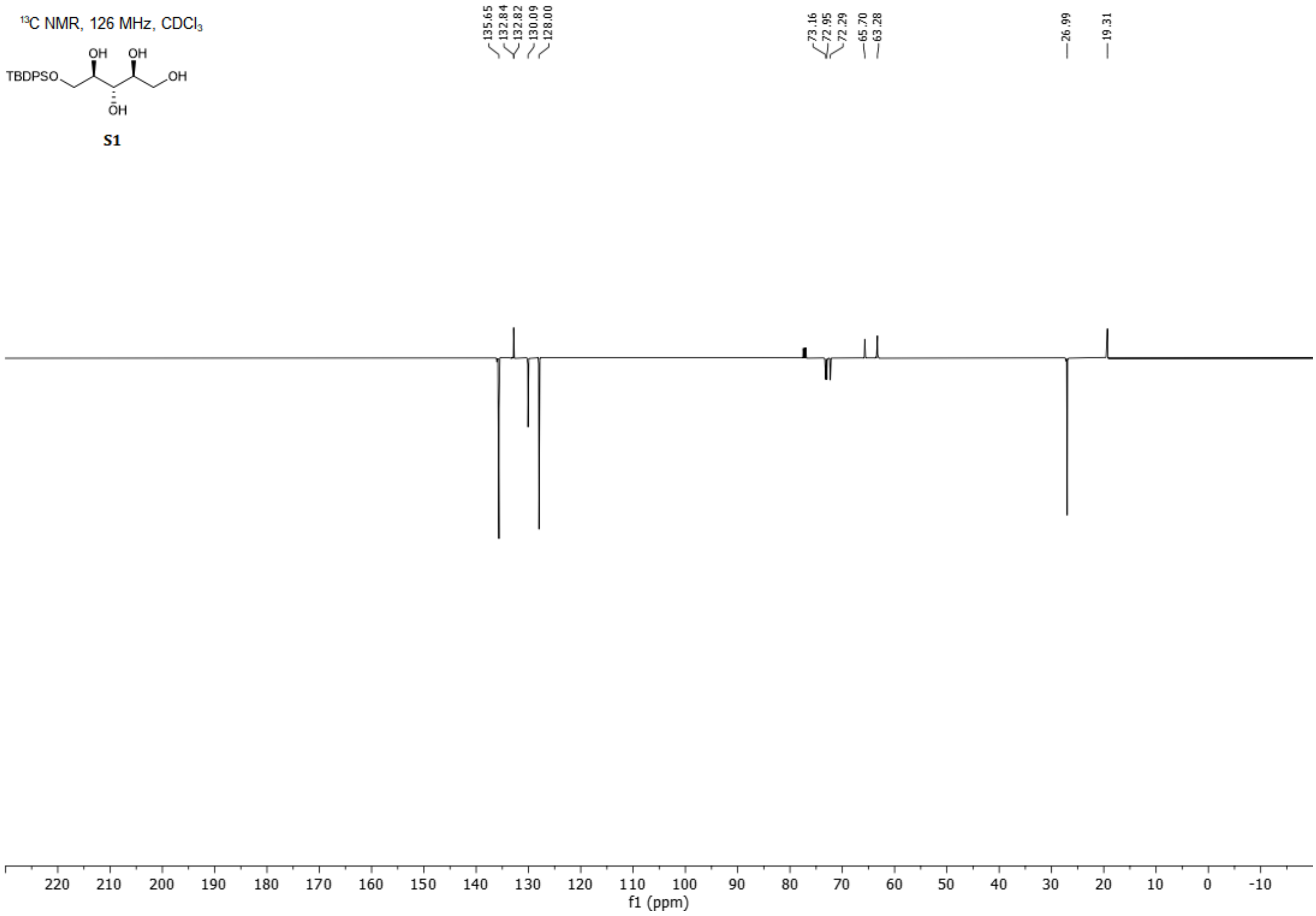

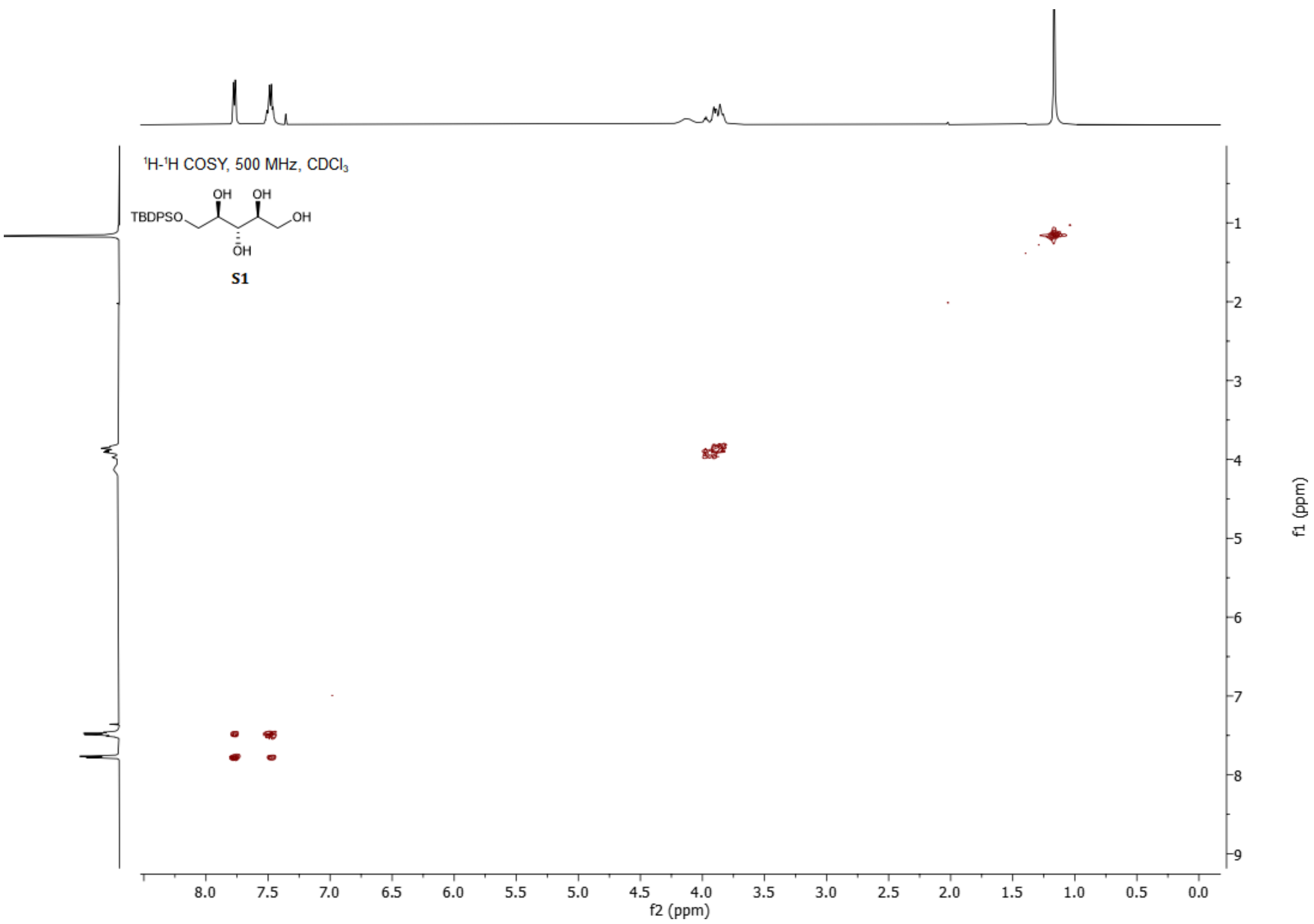

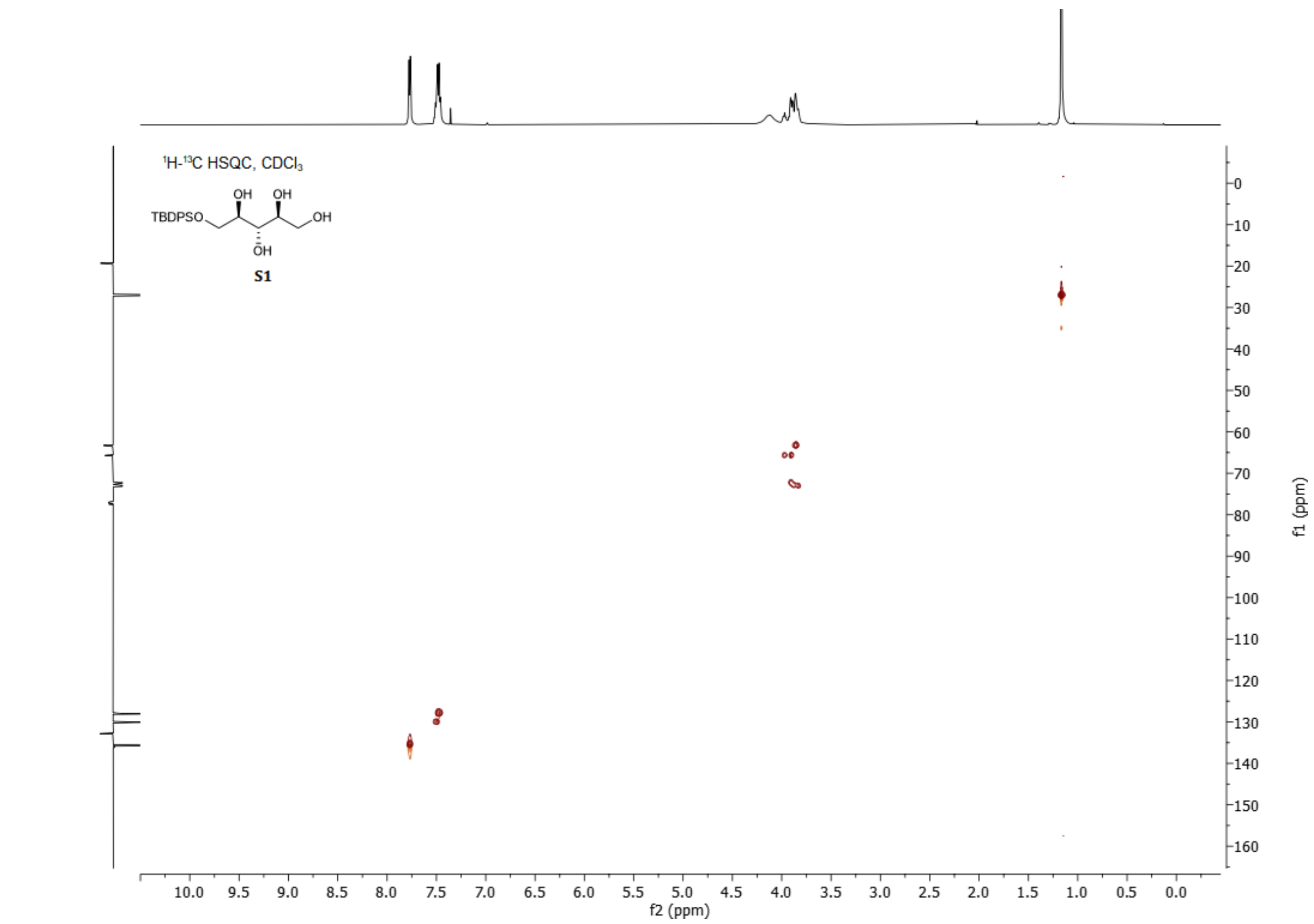

1,2,3,4-*tetra*-*O*-acetyl-5-*O*-*tert*-butyldiphenylsilyl-D-ribitol S2

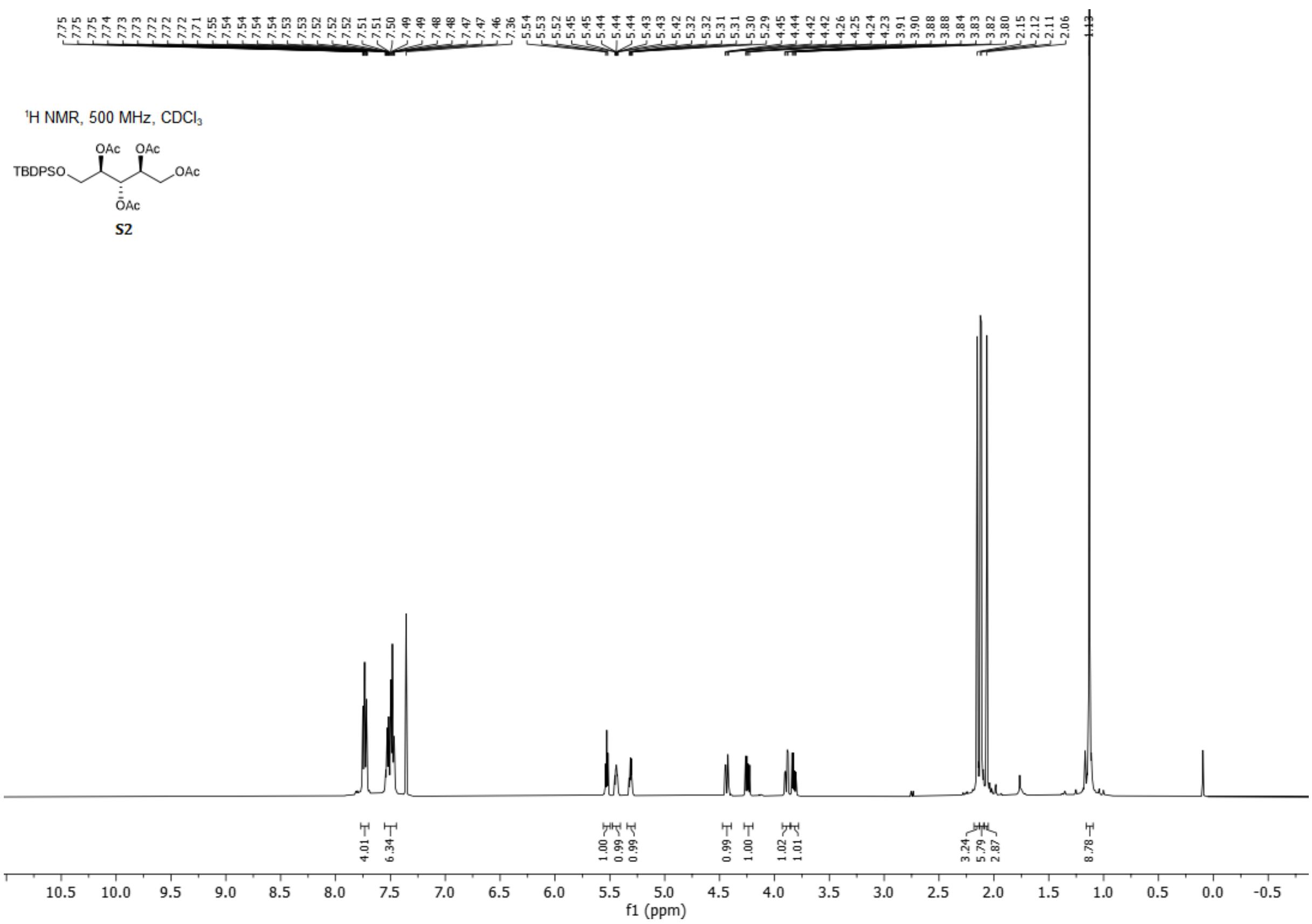
1H NMR, 500 MHz, CDCl3
TBDPSO
OAc
S2
f1 (ppm)

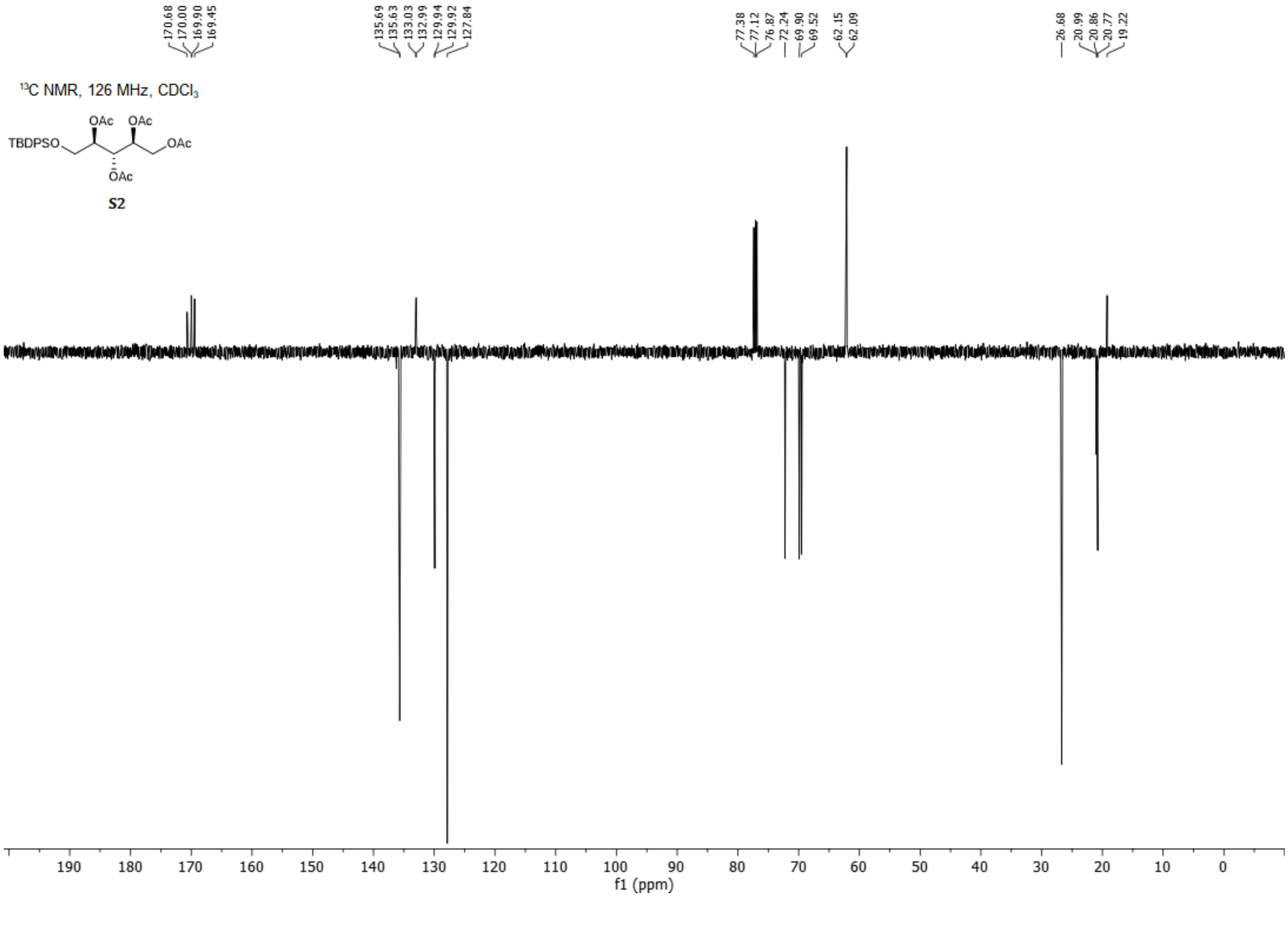
13C NMR, 126 MHz, CDCl3
TBDPSO
OAc
S2
f1 (ppm)

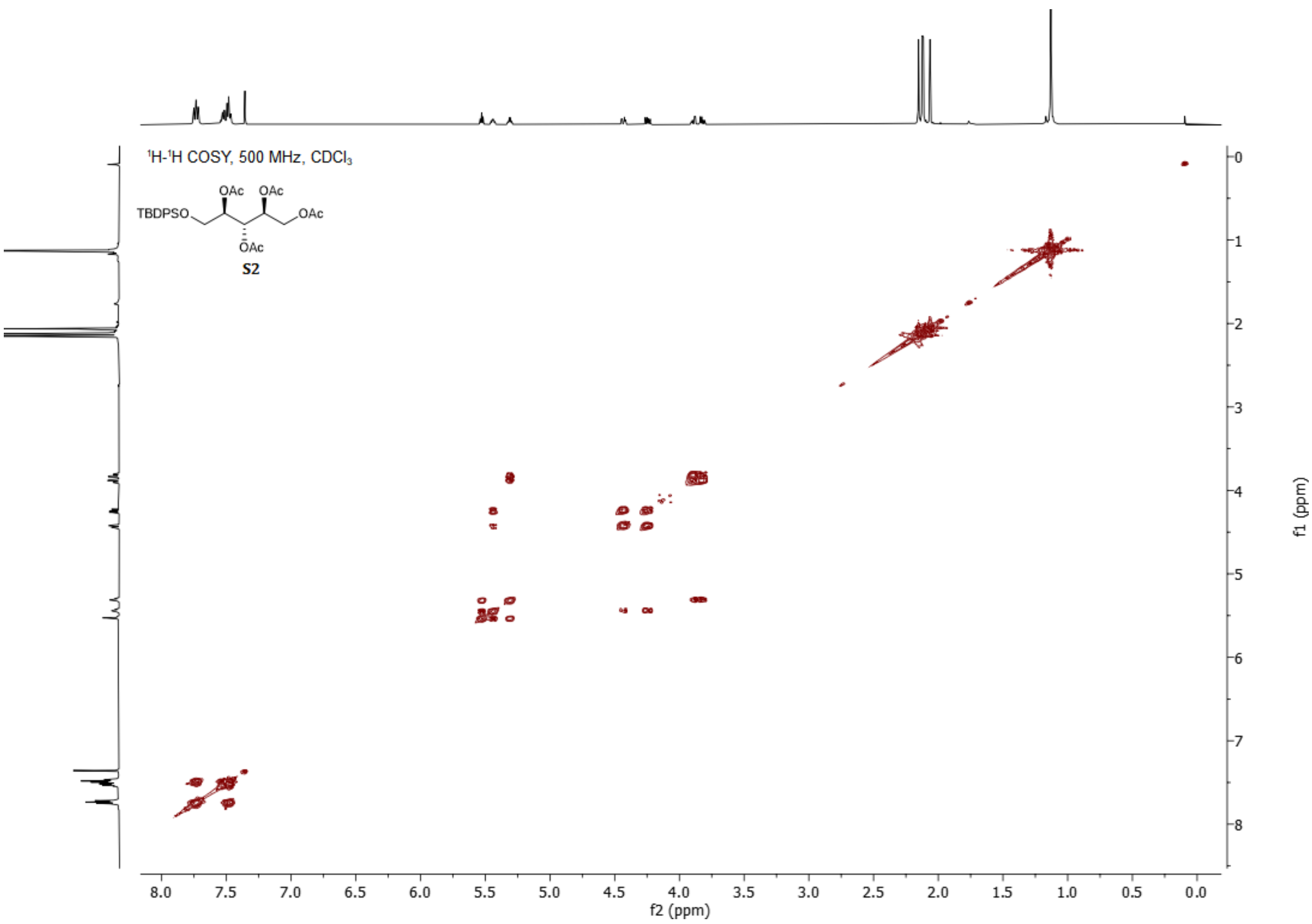

¹H-¹H COSY, 500 MHz, CDCl₃
TBDPSO
OAc
OAc
OAc
OAc
S2
f2 (ppm)
f1 (ppm)

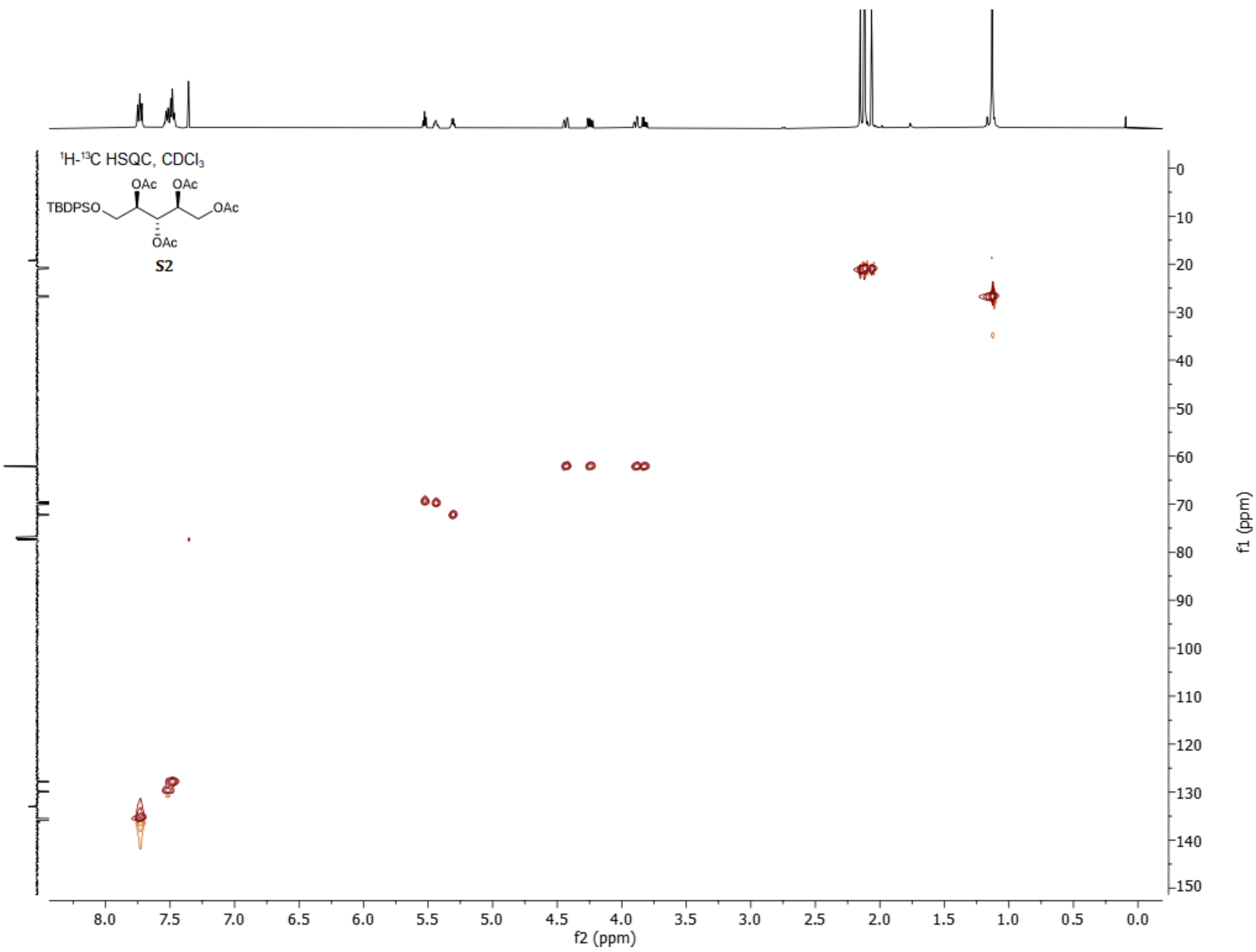

¹H-¹³C HSQC, CDCl₃
TBDPSO
OAc
OAc
OAc
OAc
S2
f2 (ppm)
f1 (ppm)

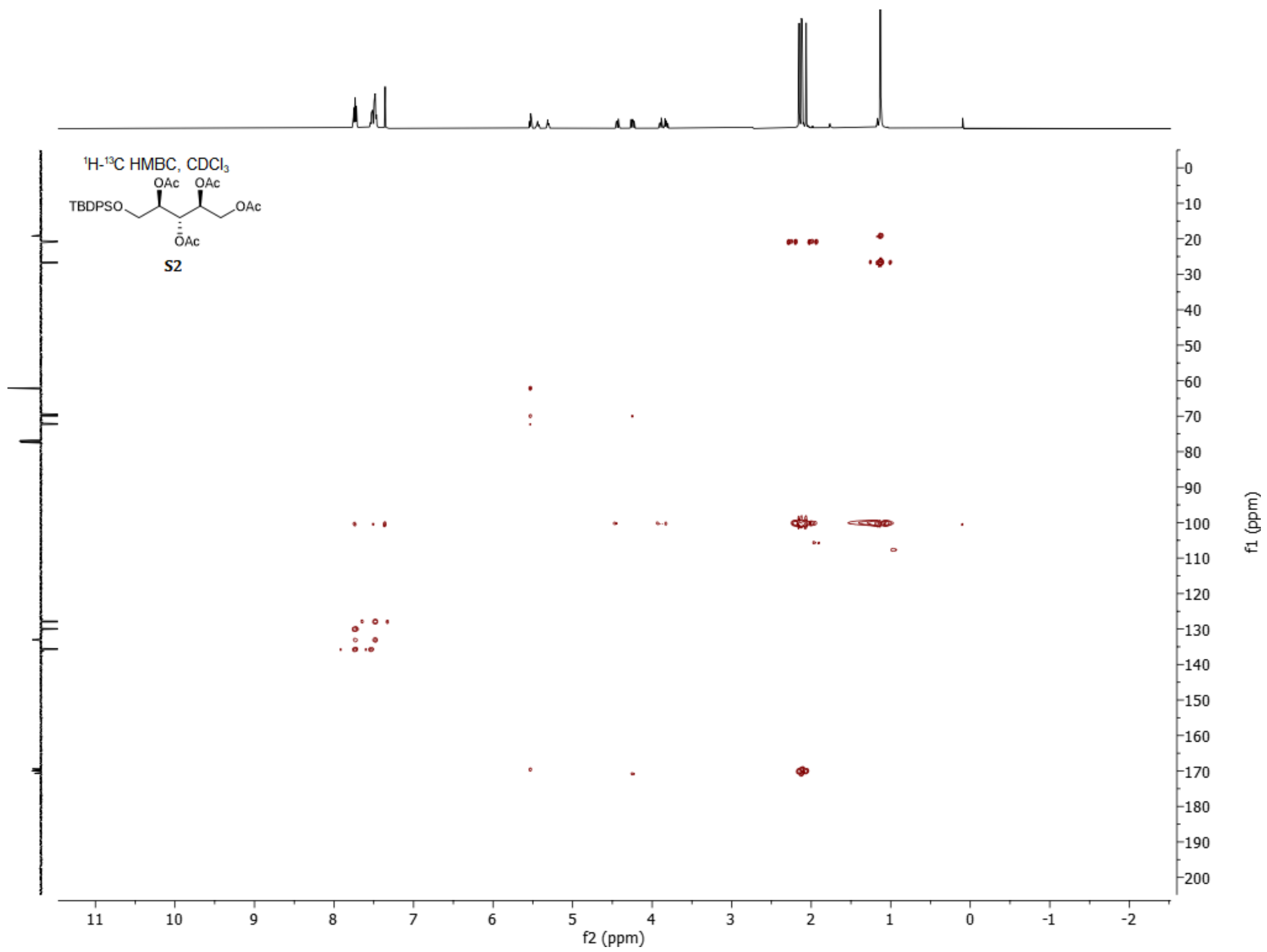

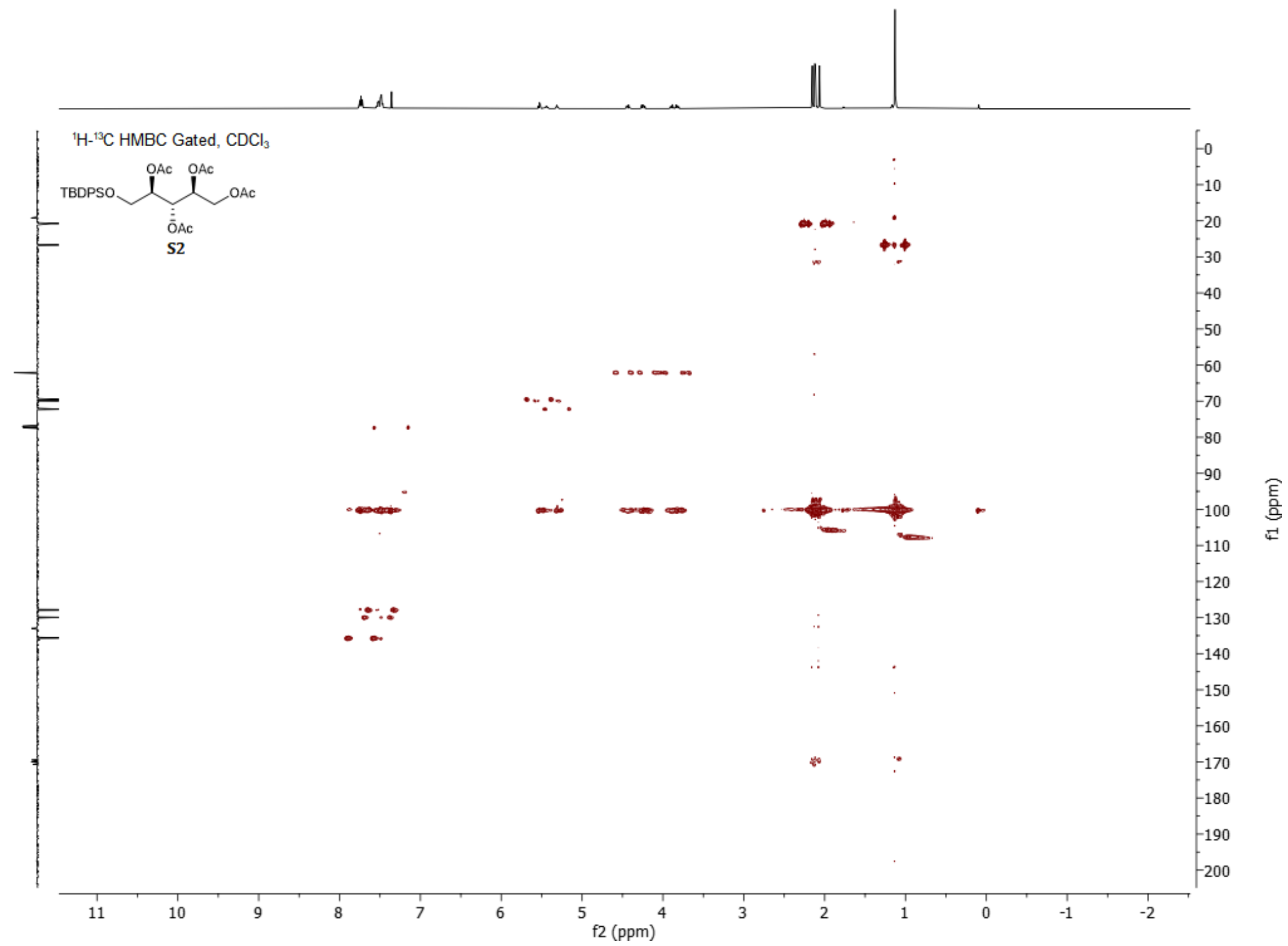

1,2,3,4-*tetra-O*-acetyl-D-ribitol 24

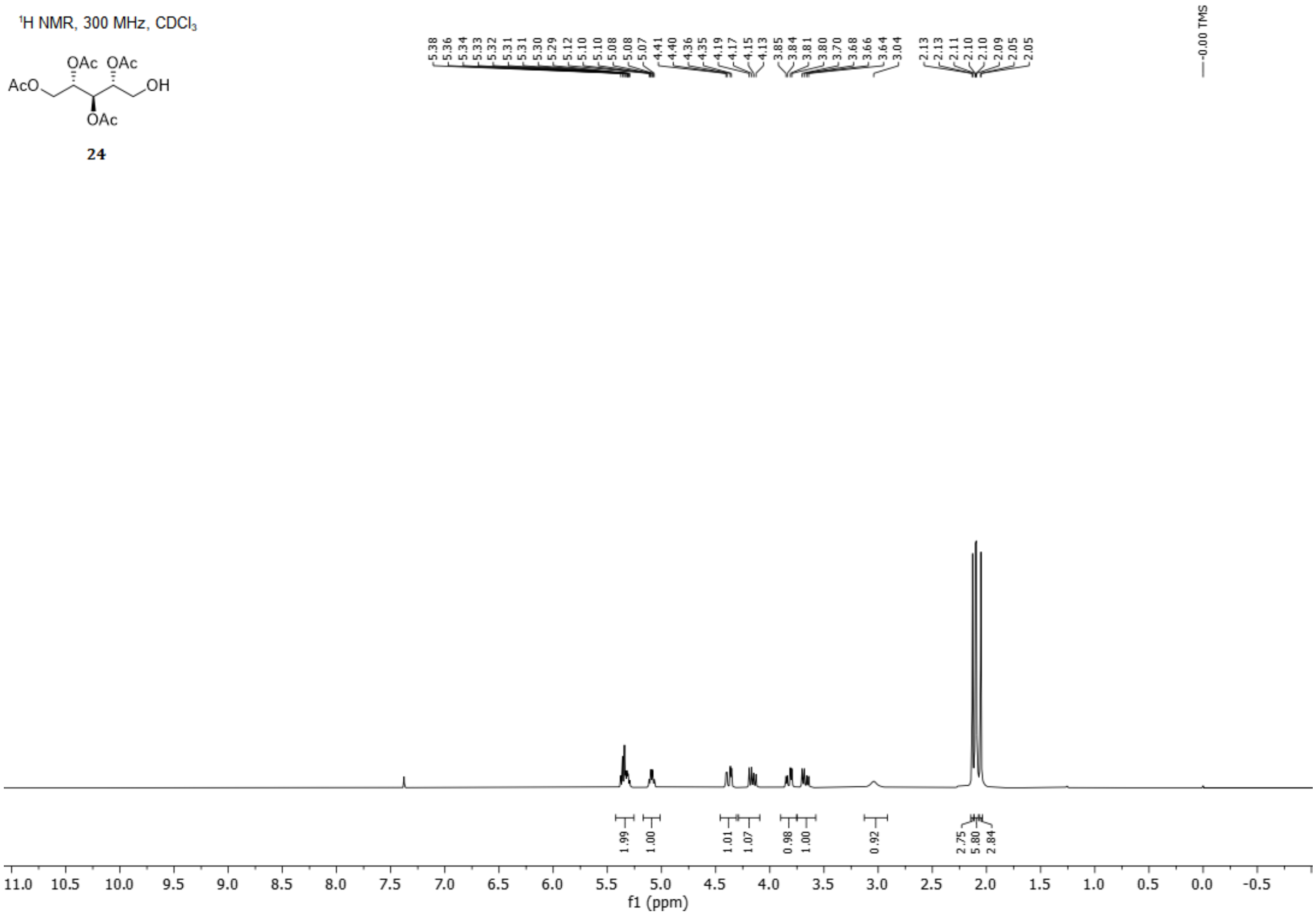

1H NMR, 300 MHz, CDCl3
AcO OAc OAc OH OAc
24
5.38 5.36 5.34 5.33 5.32 5.31 5.31 5.30 5.29 5.12 5.10 5.10 5.08 5.07 4.41 4.40 4.36 4.35 4.19 4.17 4.15 4.13 3.85 3.84 3.81 3.80 3.70 3.68 3.66 3.64 3.04
2.13 2.13 2.11 2.10 2.10 2.09 2.05 2.05
0.00 TMS
1.99 1.00 1.01 1.07 0.98 1.00 0.92 2.75 5.80 2.84
f1 (ppm)

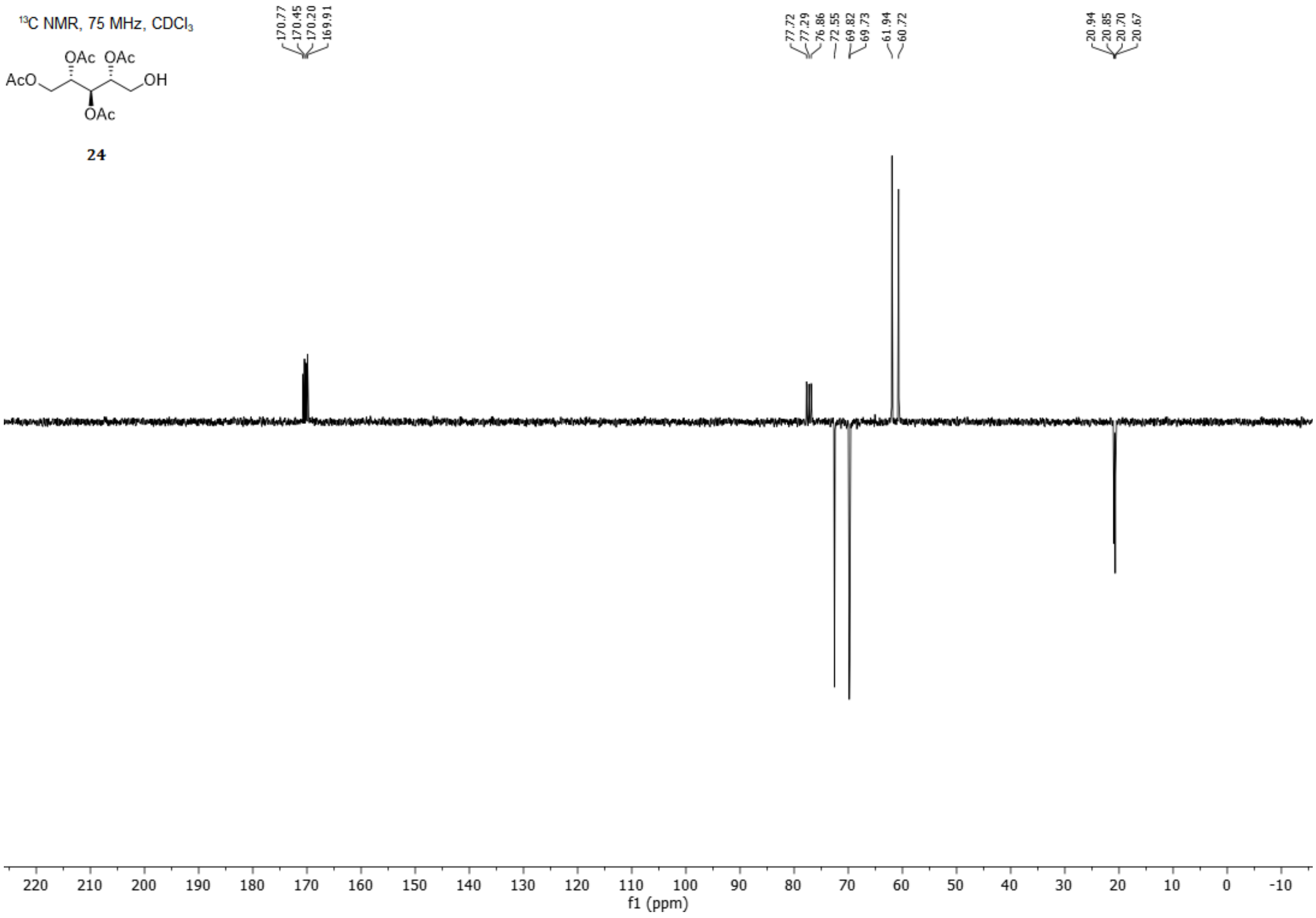

13C NMR, 75 MHz, CDCl3
AcO OAc OAc OH OAc
24
170.77 170.45 170.20 169.91
77.72 77.29 76.86 72.55 69.82 69.73 61.94 60.72
20.94 20.85 20.70 20.67
f1 (ppm)

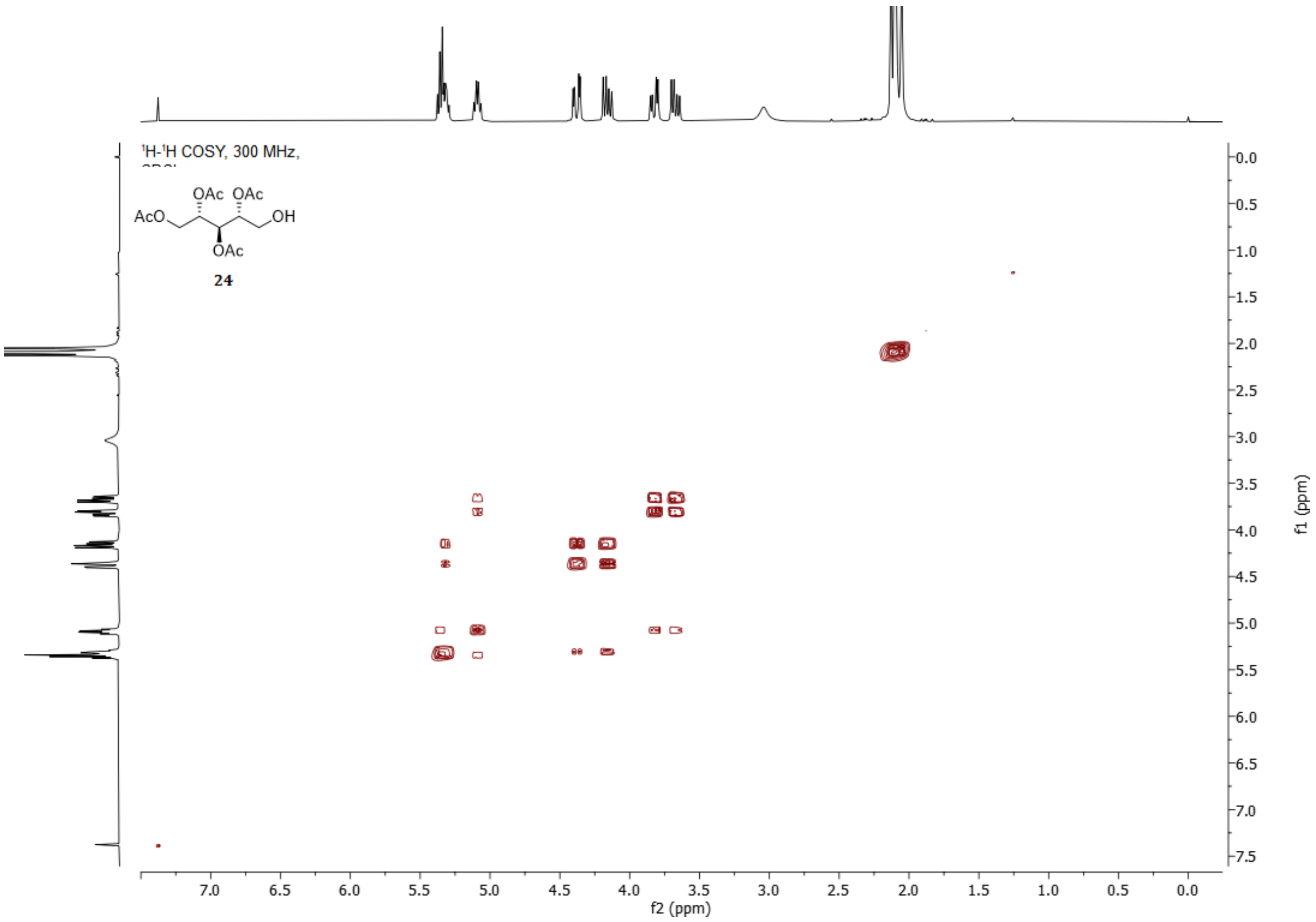
1H-1H COSY, 300 MHz,
AcO OAc OAc OH
OAc
24
f2 (ppm)
f1 (ppm)

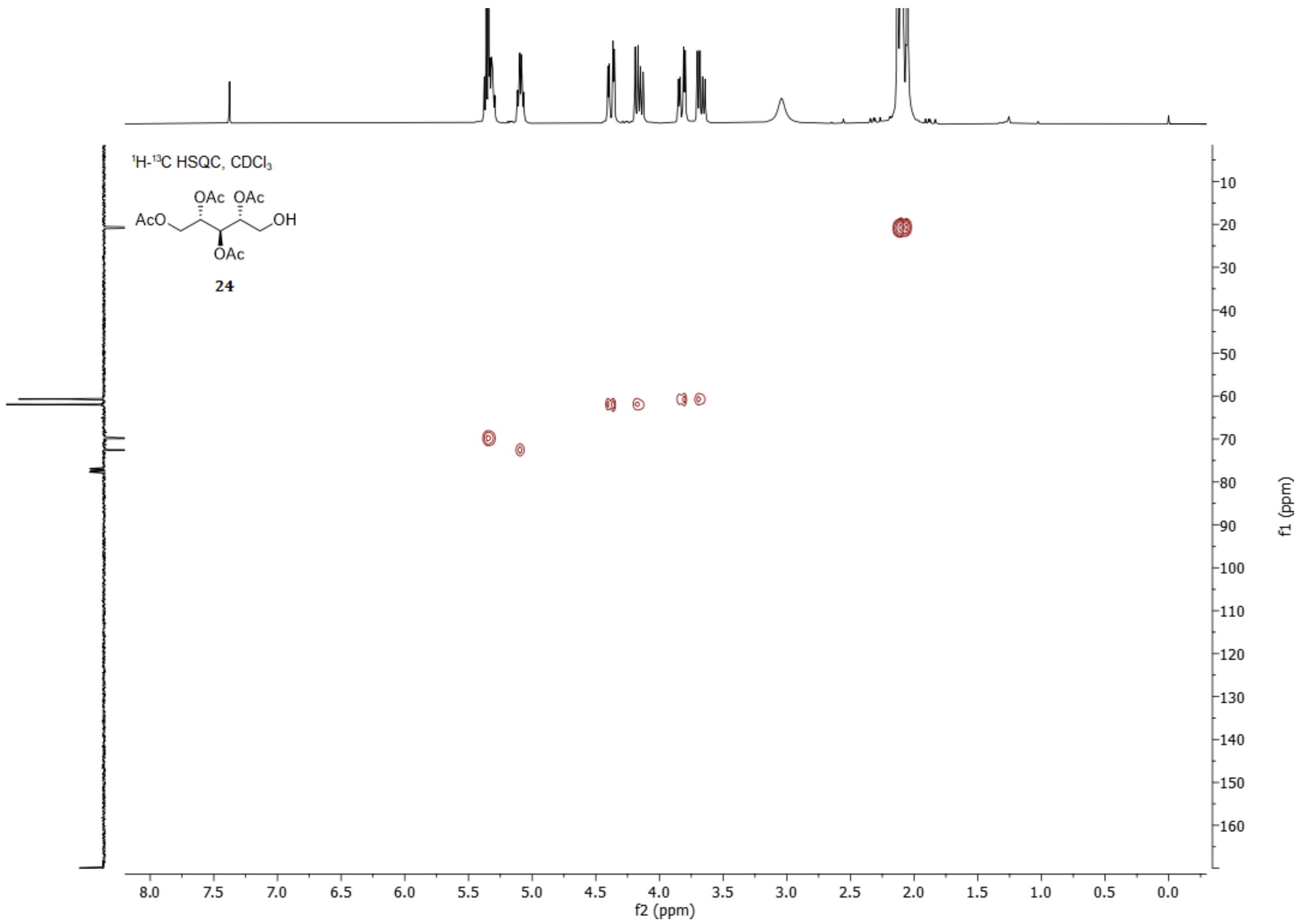
1H-13C HSQC, CDCl3
AcO OAc OAc OH
OAc
24
f2 (ppm)
f1 (ppm)

NMR Spectra for Guanosine Building Blocks

*N*2-*tert*-butoxycarbonyl-*O*6-*tert*-butyl-2',3'-*O*-isopropylidene-5'-O-*tert*-butyldimethylsilyl guanosine S3

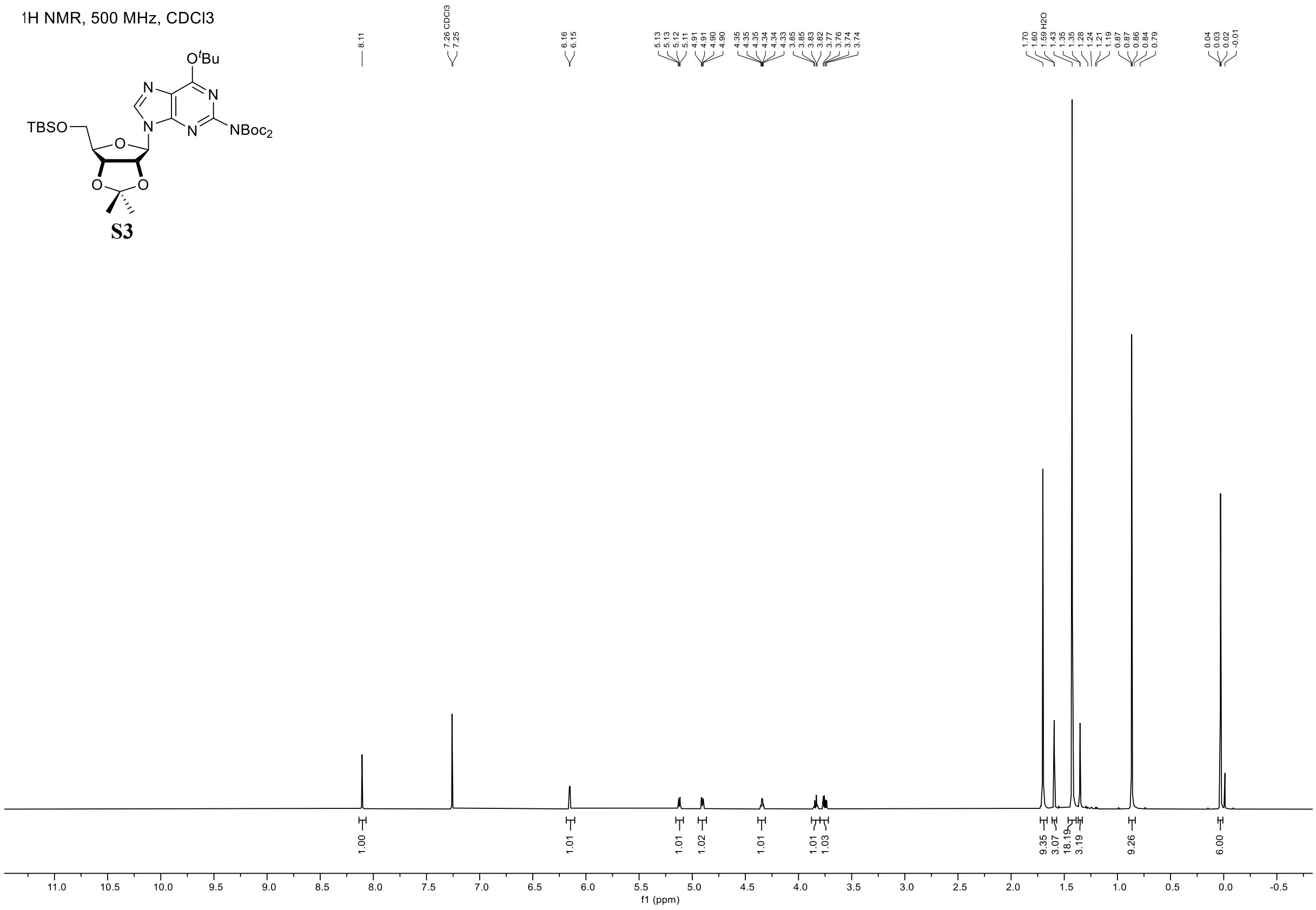

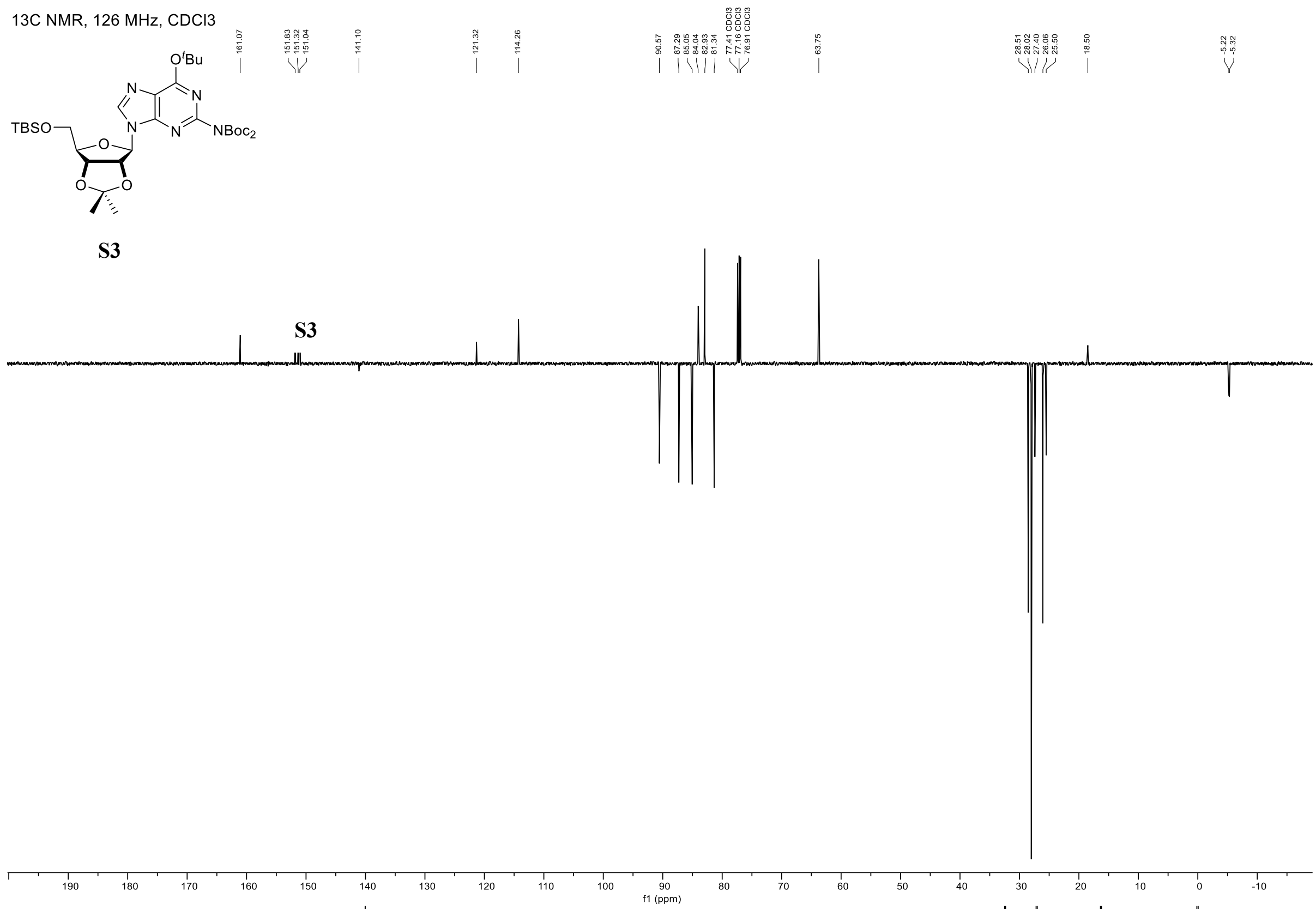
13C NMR, 126 MHz, CDCl3
161.07
151.83
151.32
151.04
141.10
121.32
114.26
90.57
87.29
85.05
84.04
82.93
81.34
77.41 CDCl3
77.16 CDCl3
76.91 CDCl3
63.75
28.51
28.02
27.40
26.06
25.50
18.50
-5.22
-5.32
O^{t}Bu
TBSO
NBoc$_2$
S3
S3
f1 (ppm)

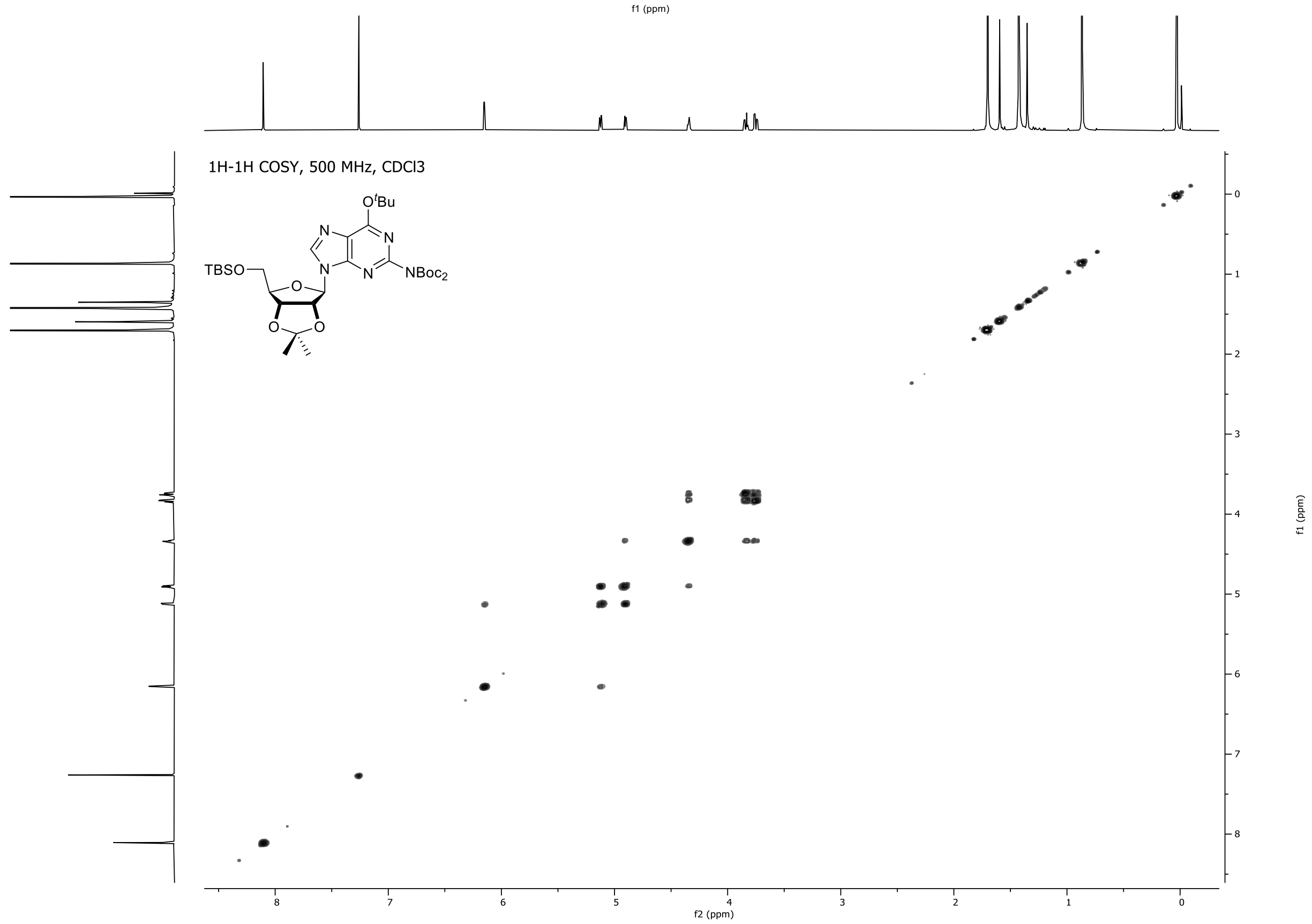
1H-1H COSY, 500 MHz, CDCl3
O^{t}Bu
TBSO
NBoc$_2$
f2 (ppm)
f1 (ppm)

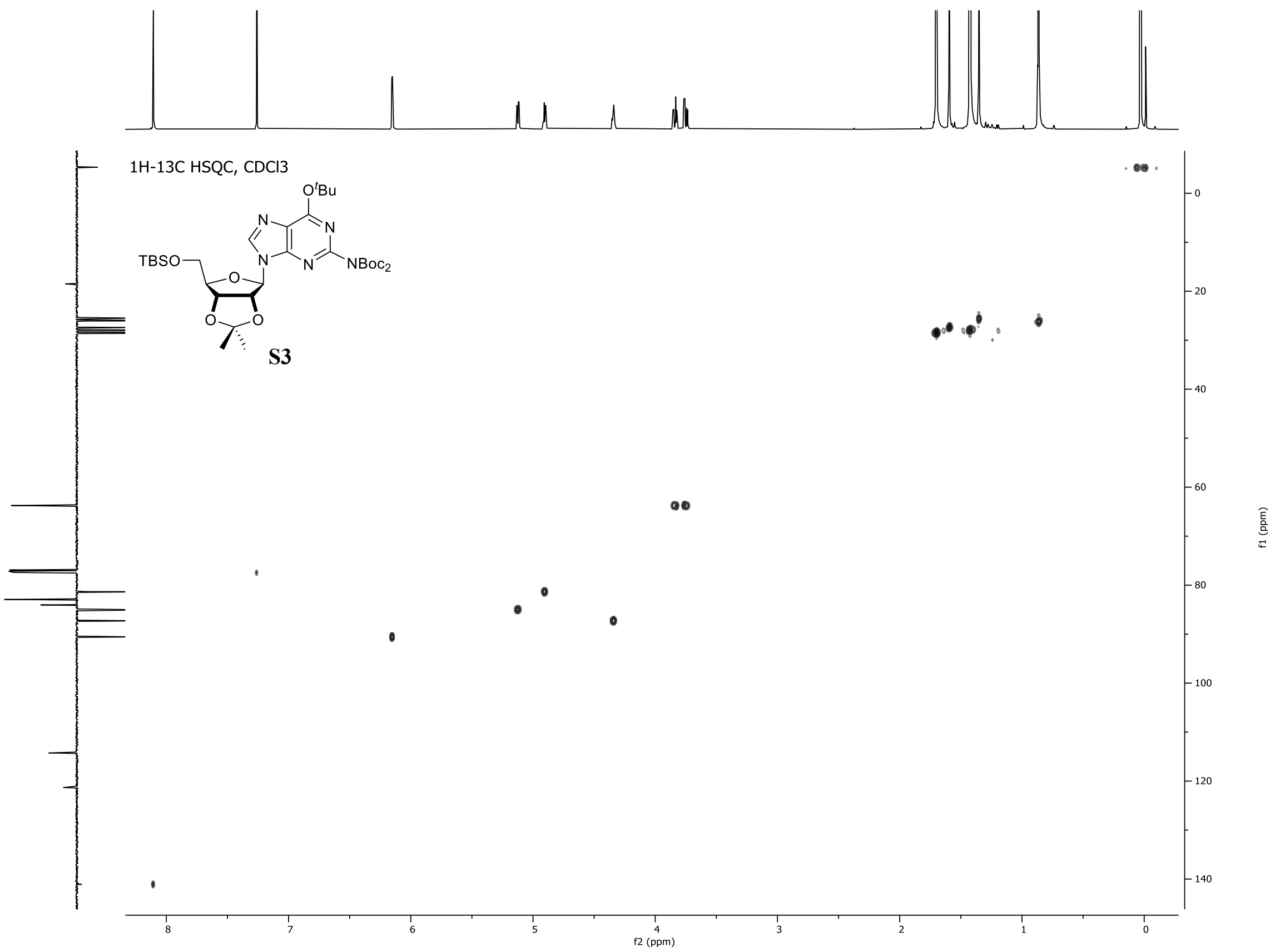

N^2-*tert*-butoxycarbonyl-O^6-*tert*-butyl-2',3'-*O*-isopropylidene guanosine 17

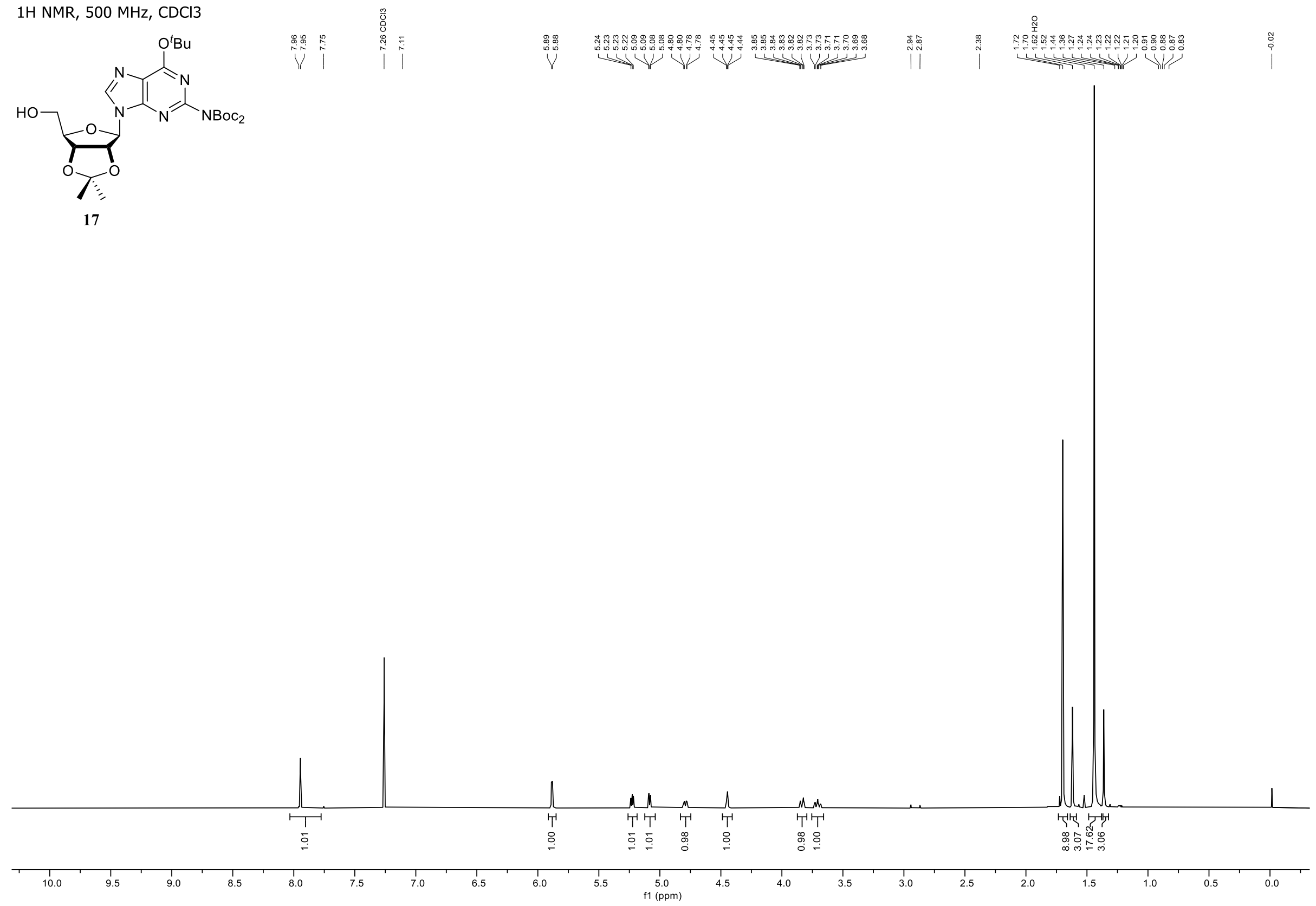

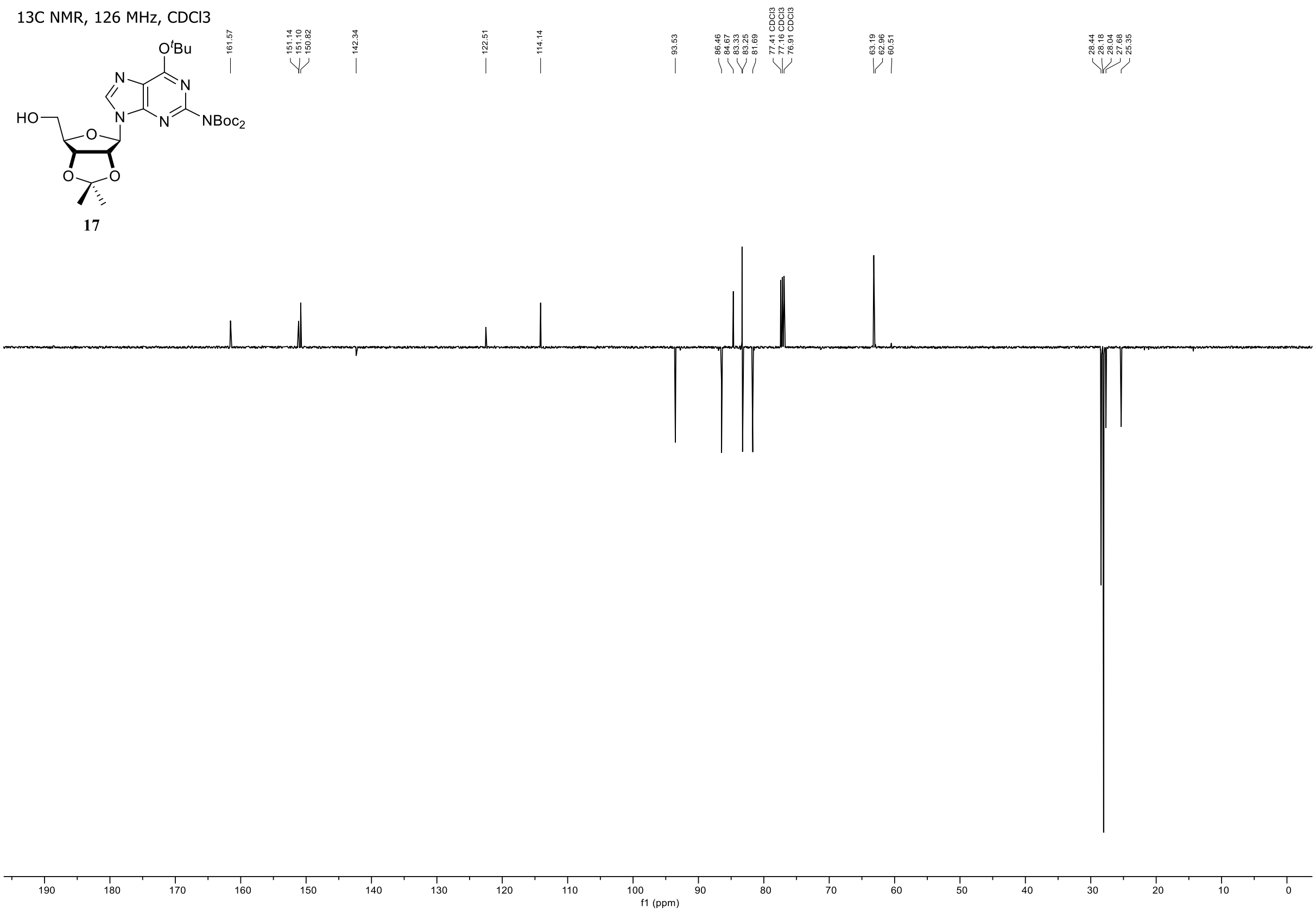
13C NMR, 126 MHz, CDCl3
O^{t}Bu
HO
NBoc$_2$
161.57
151.14
151.10
150.82
142.34
122.51
114.14
93.53
86.46
84.67
83.33
82.25
81.69
77.41 CDCl3
77.16 CDCl3
76.91 CDCl3
63.19
62.96
60.51
28.44
28.18
28.04
27.68
25.35
f1 (ppm)

17

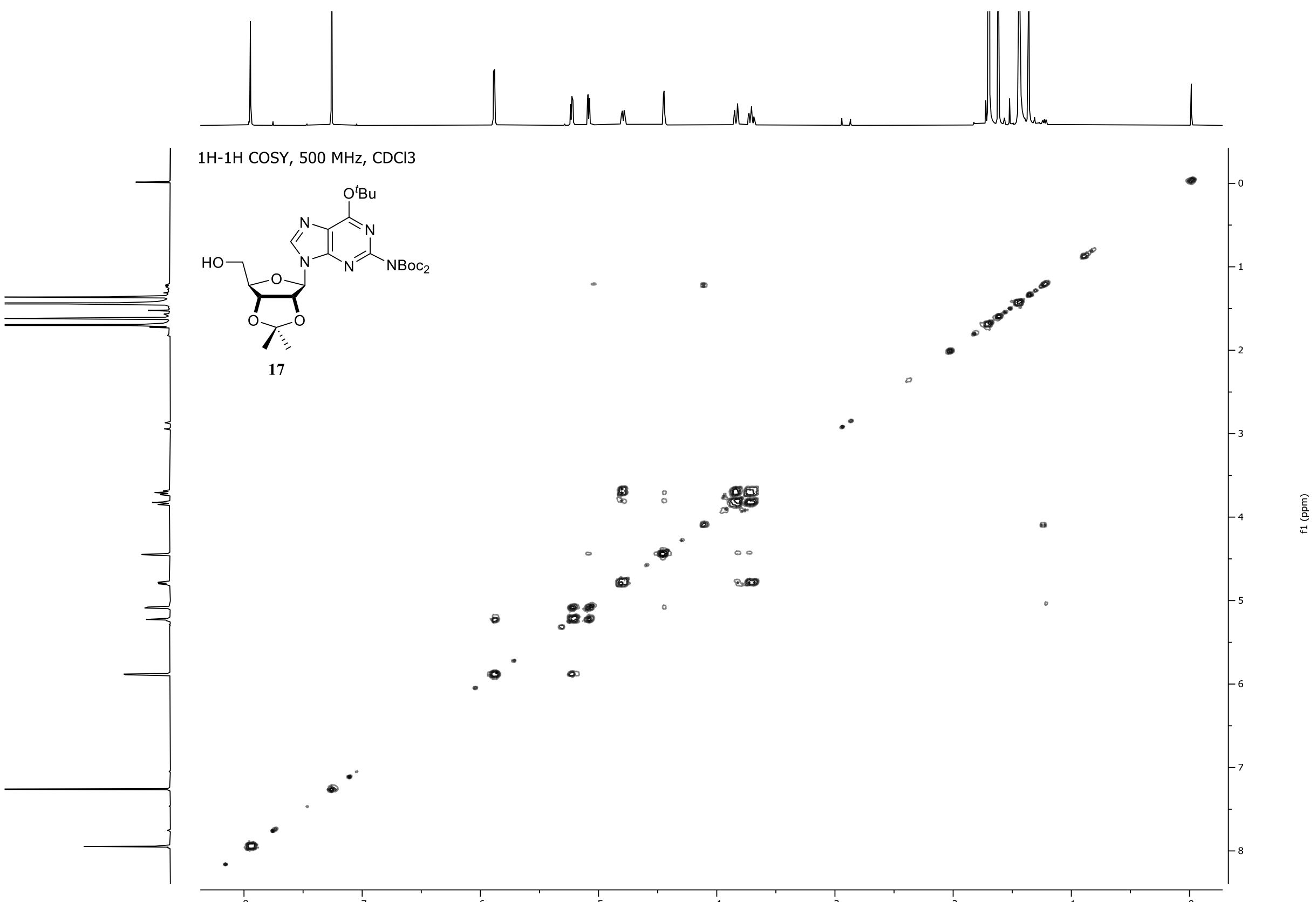
1H-1H COSY, 500 MHz, CDCl3
O^{t}Bu
HO
NBoc$_2$
f2 (ppm)
f1 (ppm)

17

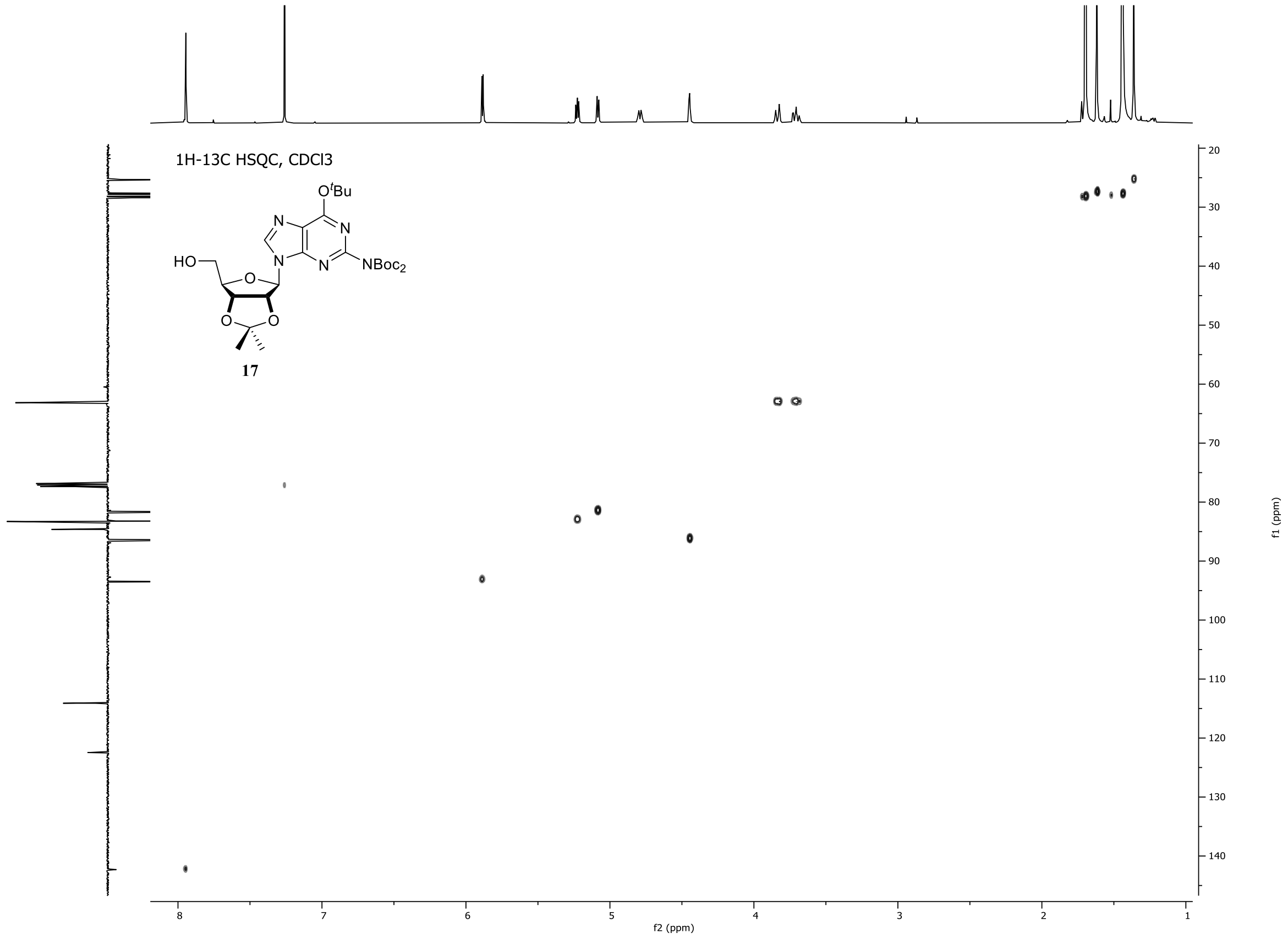

NMR Spectra of Non-Symmetric Phosphonylmethyl Phosphono(di)thioate Diesters and Precursors

me-CDP-αS tetraester 20a

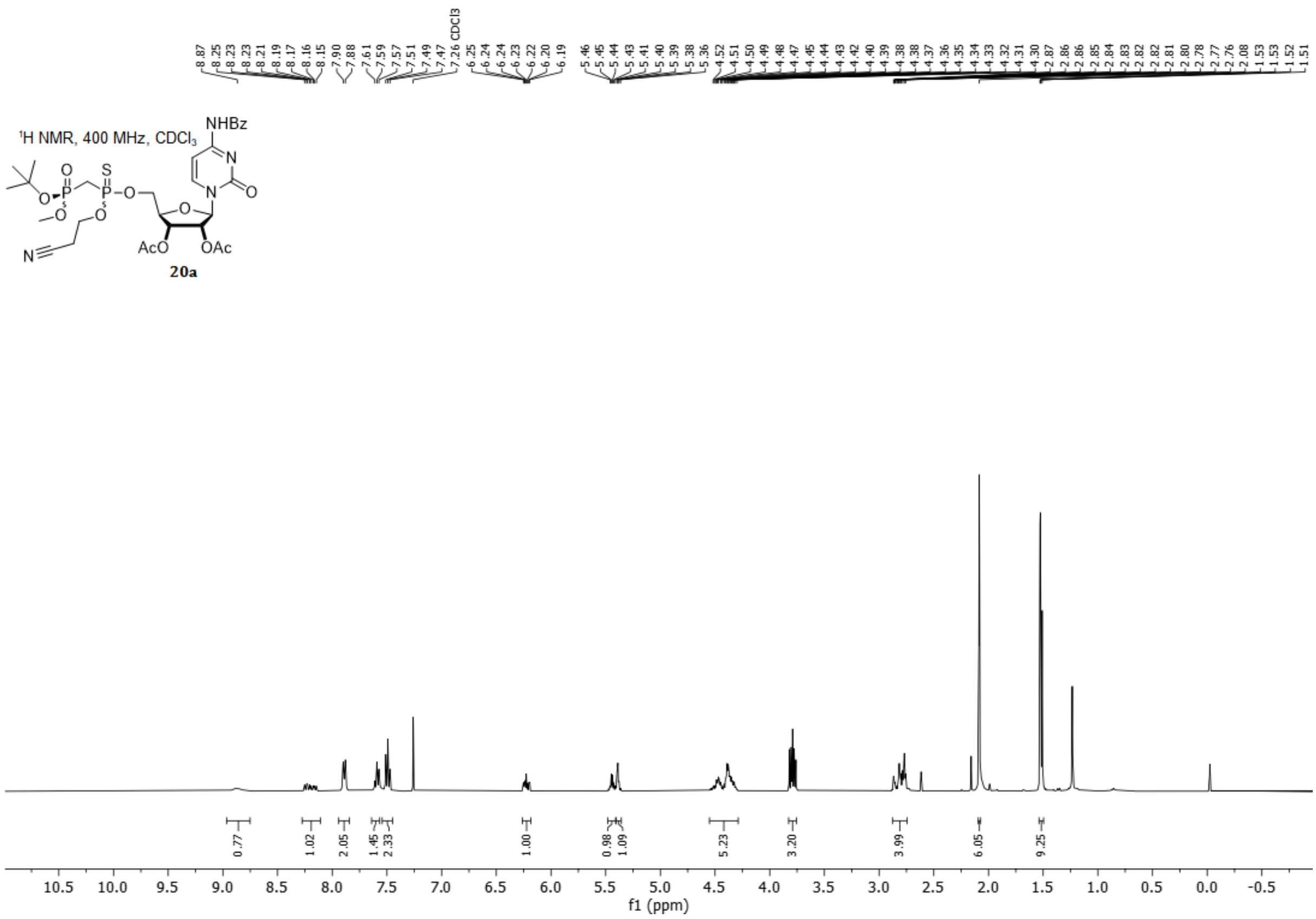

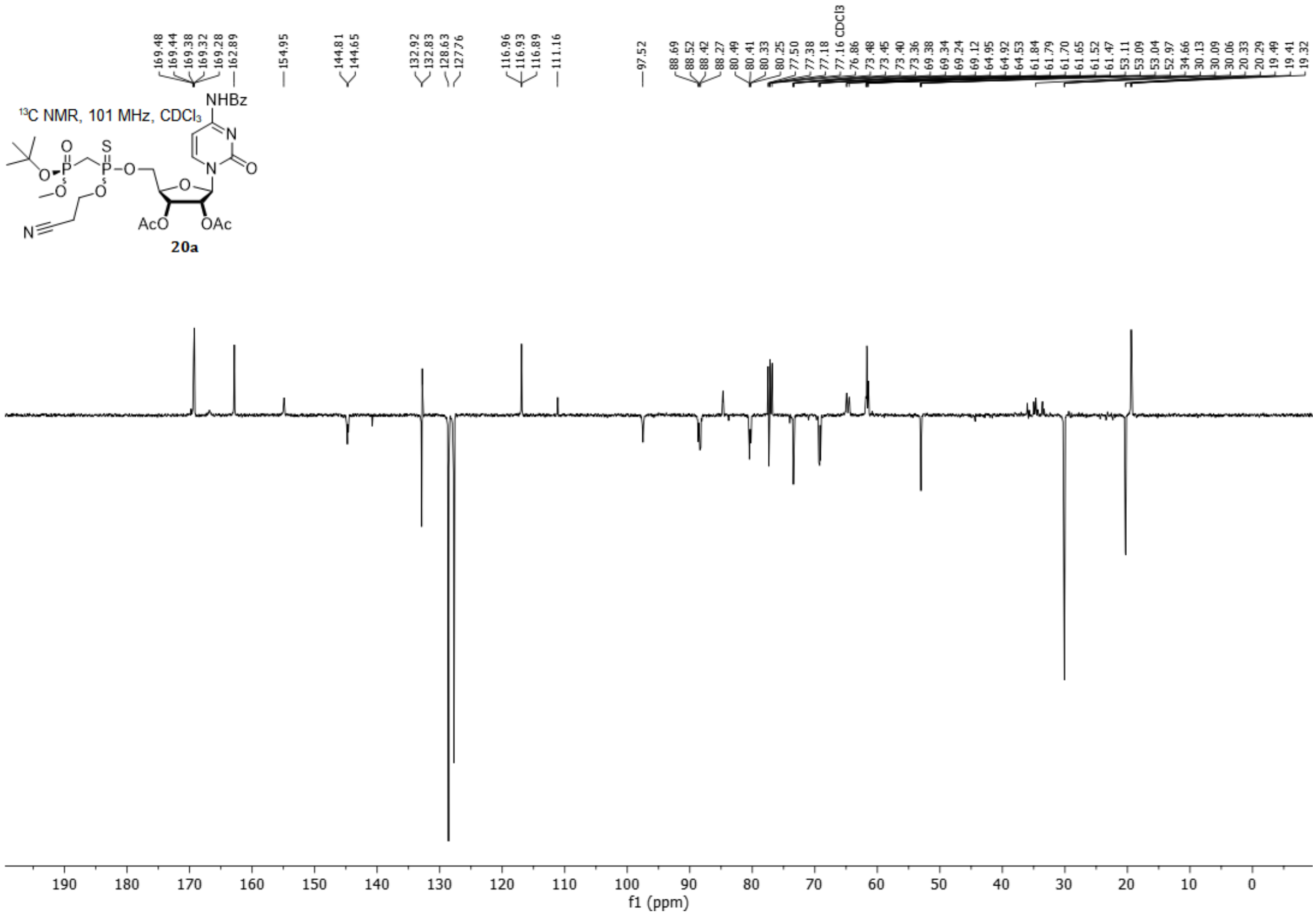
13C NMR, 101 MHz, CDCl3
NHBz
AcO
OAc
20a
169.48
169.44
169.38
169.32
169.28
162.89
154.95
144.81
144.65
132.92
132.83
128.63
127.76
116.96
116.93
116.89
111.16
97.52
88.69
88.52
88.42
88.27
80.49
80.41
80.33
80.25
77.50
77.38
77.18
77.16 CDCl3
76.86
73.48
73.45
73.40
73.36
69.38
69.34
69.24
69.12
64.95
64.92
64.53
61.84
61.79
61.70
61.65
61.52
61.47
53.11
53.09
53.04
52.97
34.66
30.13
30.09
30.06
20.33
20.29
19.49
19.41
19.32
f1 (ppm)

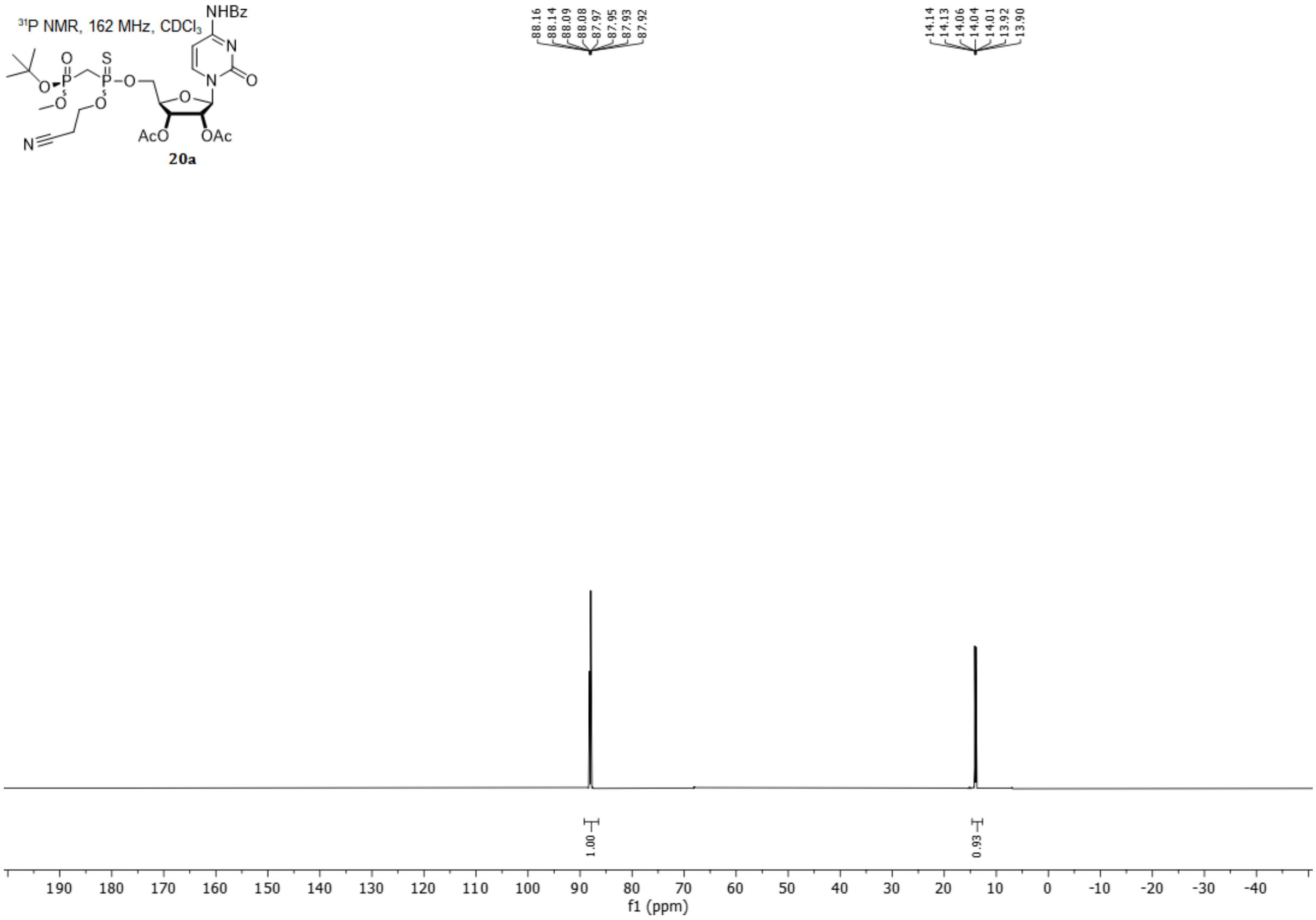
31P NMR, 162 MHz, CDCl3
NHBz
AcO
OAc
20a
88.16
88.14
88.09
88.08
87.97
87.95
87.93
87.92
14.14
14.13
14.06
14.04
14.01
13.92
13.90
1.00
0.93
f1 (ppm)

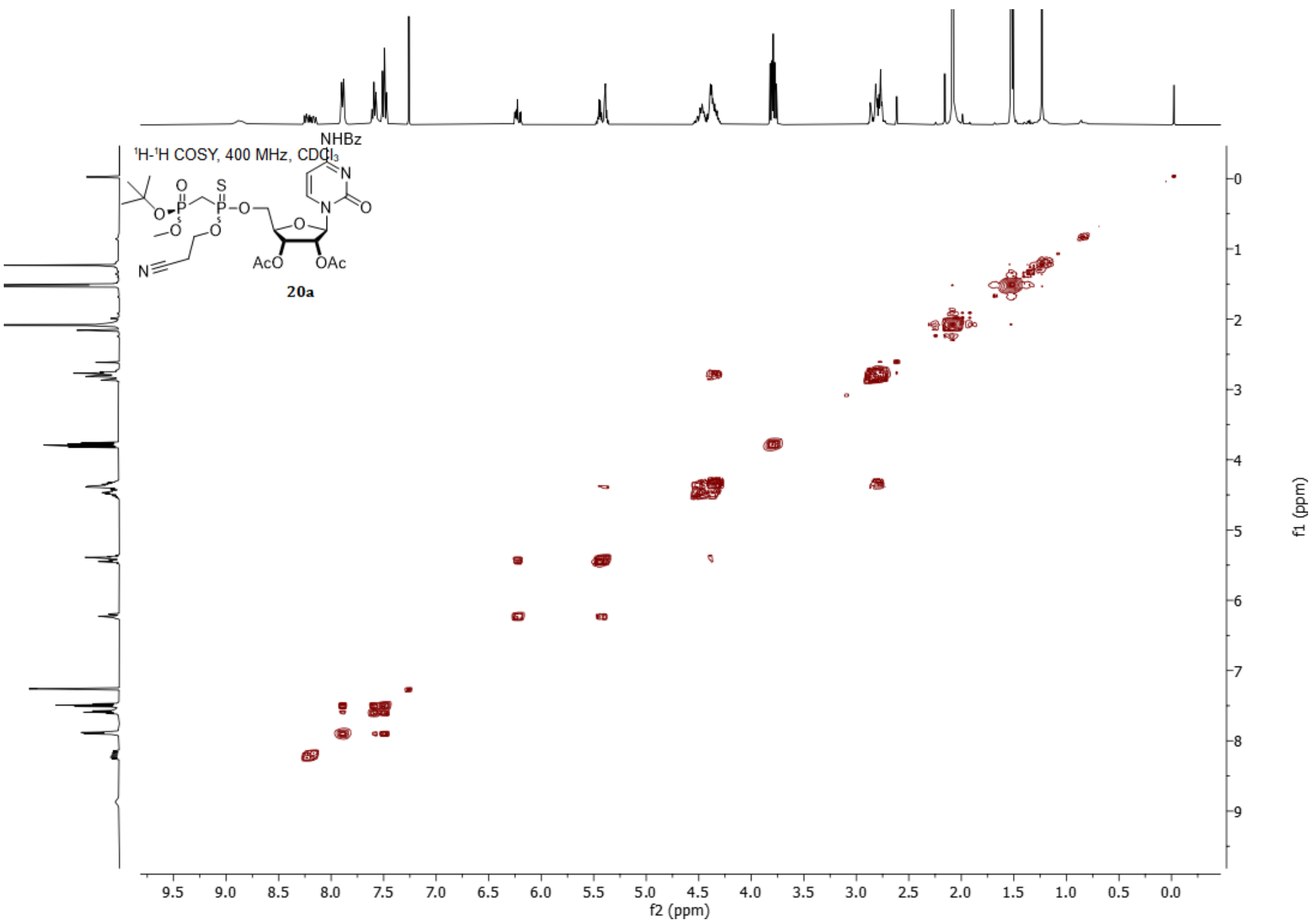
1H-1H COSY, 400 MHz, CDCl3
NHBz
20a
f2 (ppm)
f1 (ppm)

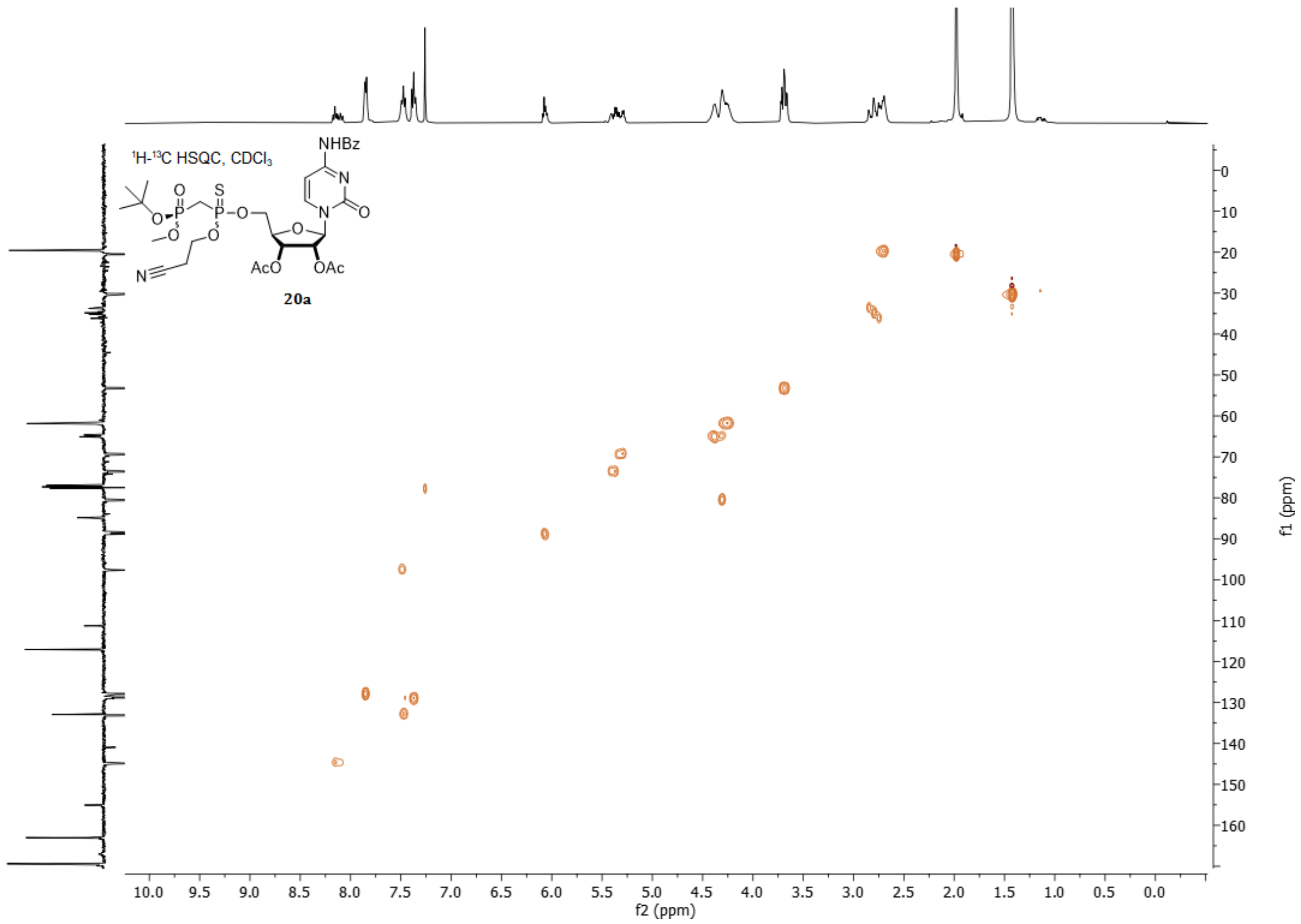
1H-13C HSQC, CDCl3
NHBz
20a
f2 (ppm)
f1 (ppm)

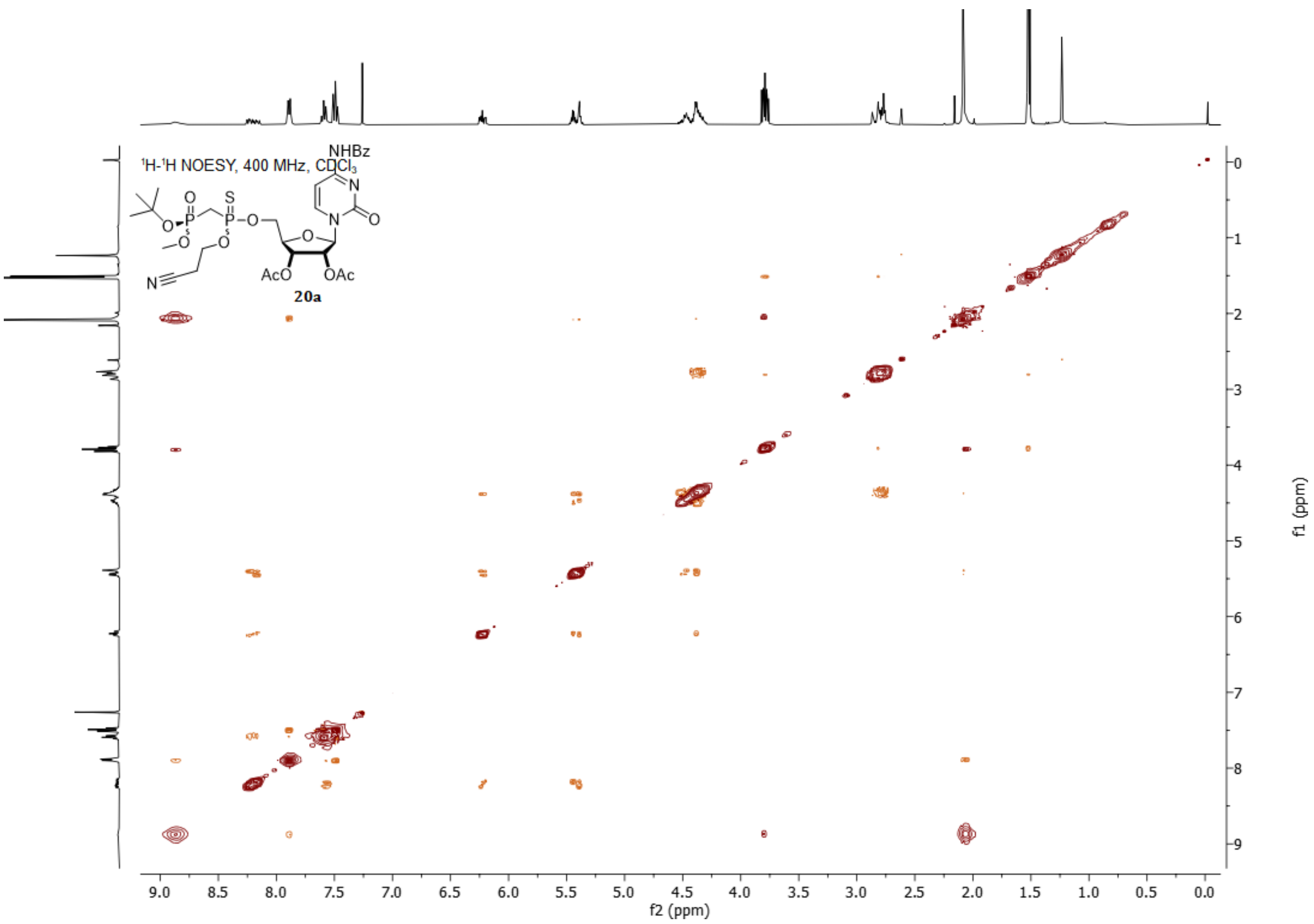

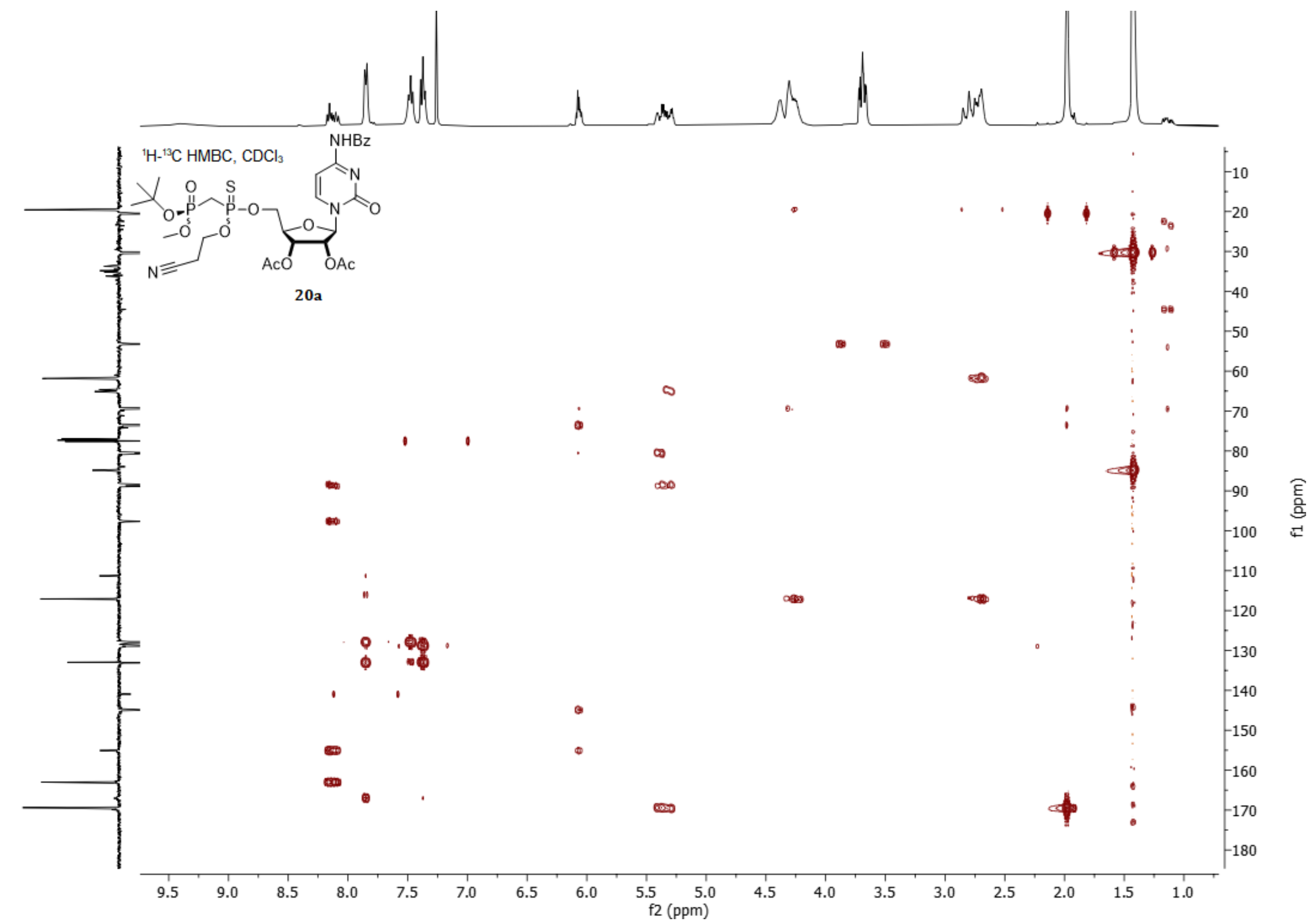

me-CDP-αS$_2$ tetraester 20b

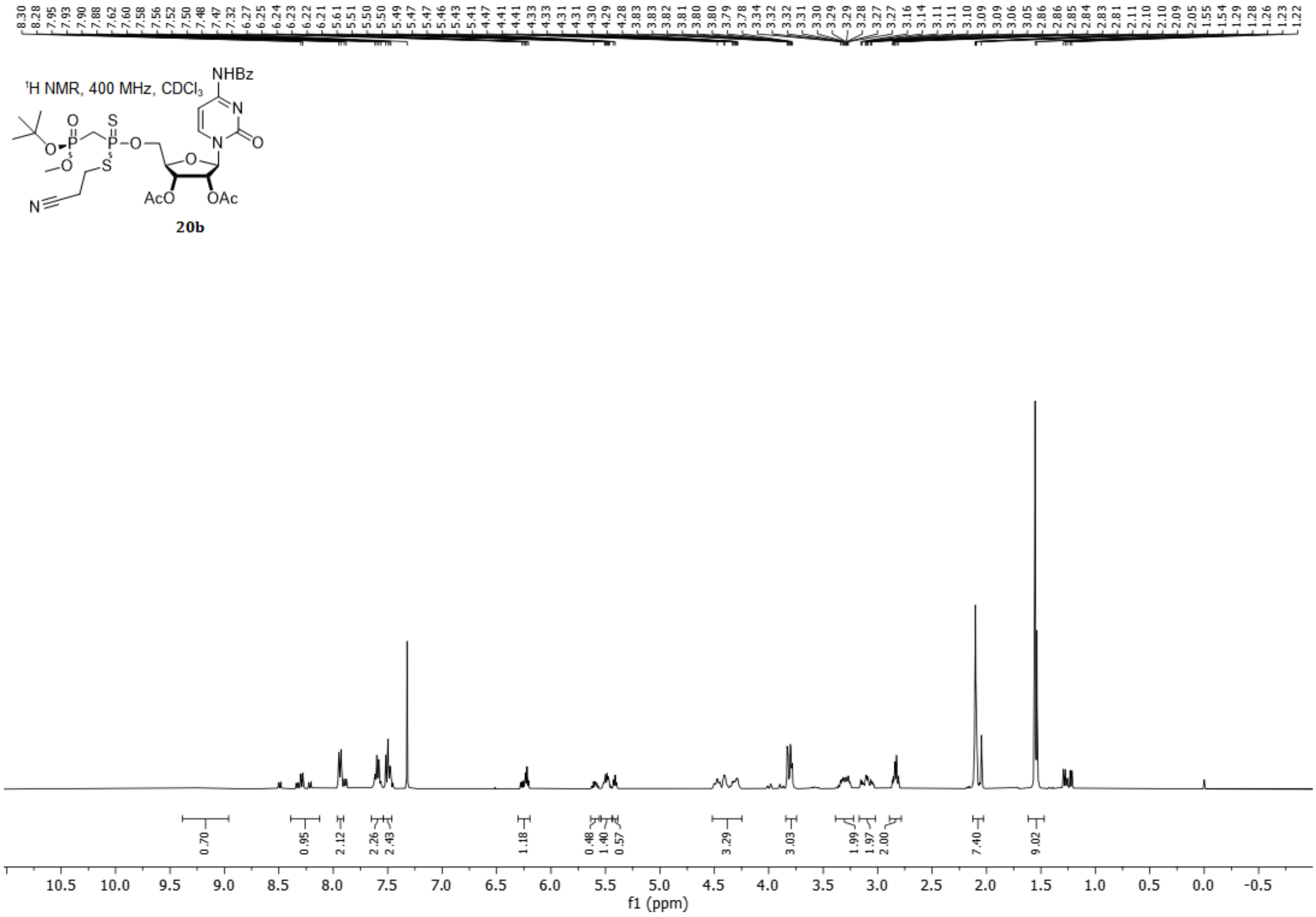
1H NMR, 400 MHz, CDCl3
NHBz
20b
8.30 8.28 7.95 7.93 7.90 7.88 7.62 7.60 7.58 7.56 7.52 7.50 7.48 7.47 7.32 6.27 6.25 6.24 6.23 6.22 6.21 5.61 5.51 5.50 5.50 5.49 5.47 5.47 5.46 5.43 5.41 4.47 4.41 4.41 4.33 4.33 4.31 4.31 4.30 4.29 4.28 3.83 3.83 3.82 3.81 3.80 3.80 3.79 3.78 3.34 3.32 3.32 3.31 3.30 3.29 3.29 3.28 3.27 3.27 3.16 3.14 3.11 3.11 3.10 3.09 3.09 3.06 3.05 2.86 2.86 2.85 2.84 2.83 2.81 2.11 2.10 2.10 2.09 2.05 1.55 1.54 1.29 1.28 1.26 1.23 1.22
0.70 0.95 2.12 2.26 2.43 1.18 0.48 1.40 0.57 3.29 3.03 1.99 1.97 2.00 7.40 9.02
10.5 10.0 9.5 9.0 8.5 8.0 7.5 7.0 6.5 6.0 5.5 5.0 4.5 4.0 3.5 3.0 2.5 2.0 1.5 1.0 0.5 0.0 -0.5
f1 (ppm)

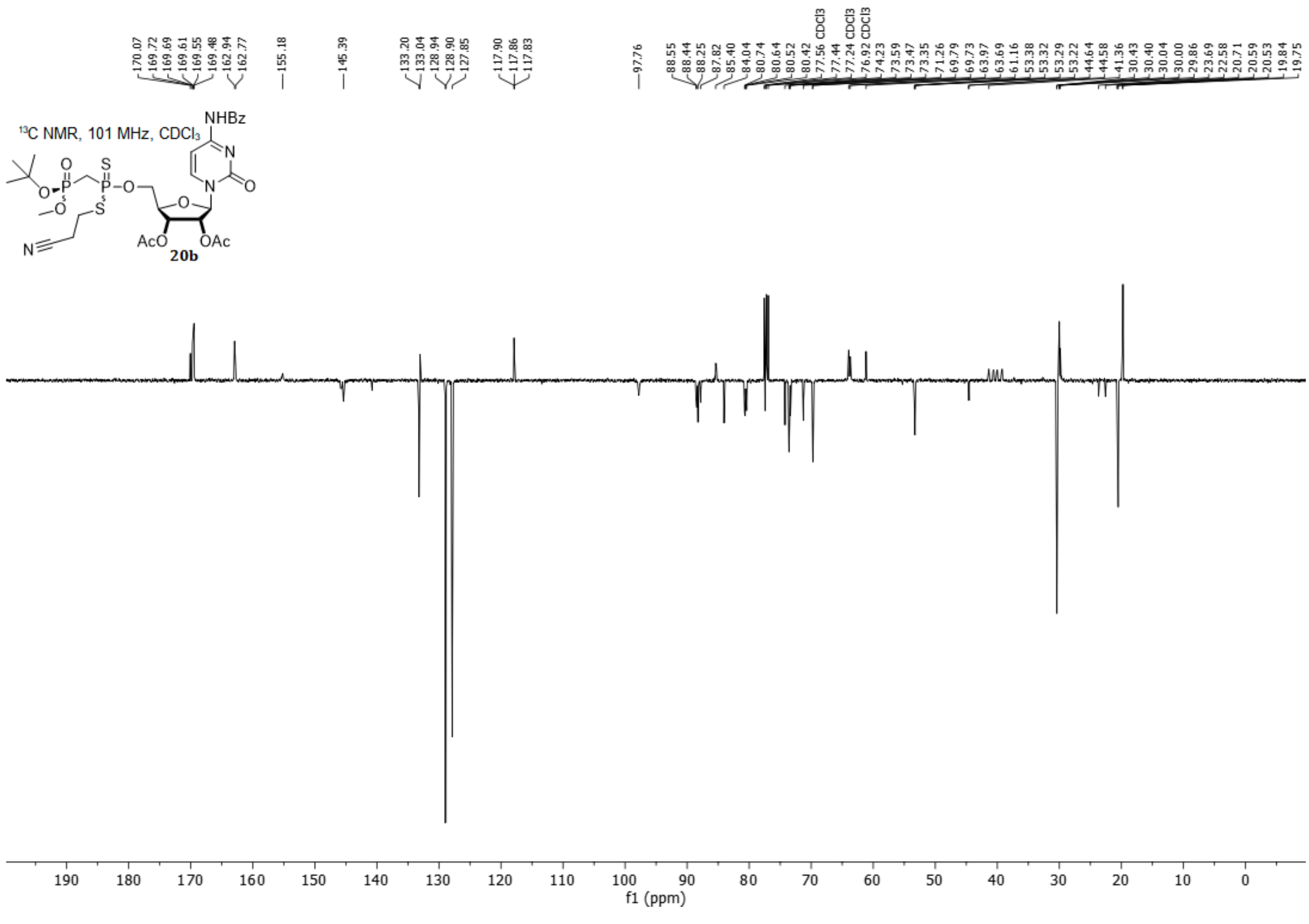
13C NMR, 101 MHz, CDCl3
NHBz
20b
170.07 169.72 169.69 169.61 169.55 169.48 162.94 162.77 155.18 145.39 133.20 133.04 128.94 128.90 127.85 117.90 117.86 117.83 97.76 88.55 88.44 88.25 87.82 85.40 84.04 80.74 80.64 80.52 80.42 77.56 CDCl3 77.44 77.24 CDCl3 76.92 CDCl3 74.23 73.59 73.47 73.35 71.26 69.79 69.73 63.97 63.69 61.16 53.38 53.32 53.29 53.22 44.64 44.58 41.36 30.43 30.40 30.04 30.00 29.86 23.69 22.58 20.71 20.59 20.53 19.84 19.75
190 180 170 160 150 140 130 120 110 100 90 80 70 60 50 40 30 20 10 0
f1 (ppm)

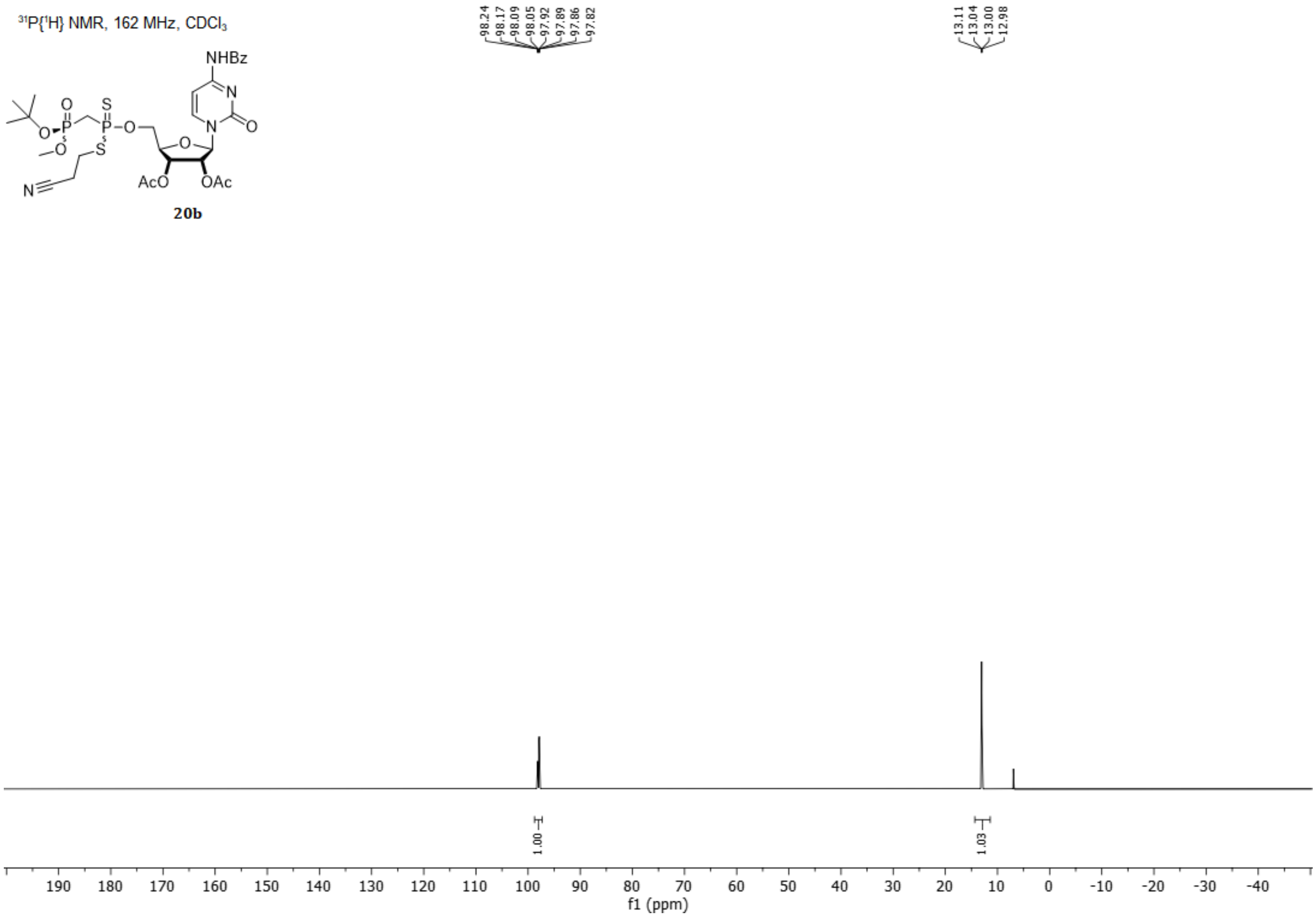
31P{1H} NMR, 162 MHz, CDCl3
NHBz
S
O
O
N
N
O
AcO
OAc
20b
98.24
98.17
98.09
98.05
97.92
97.89
97.86
97.82
13.11
13.04
13.00
12.98
1.00
1.03
f1 (ppm)

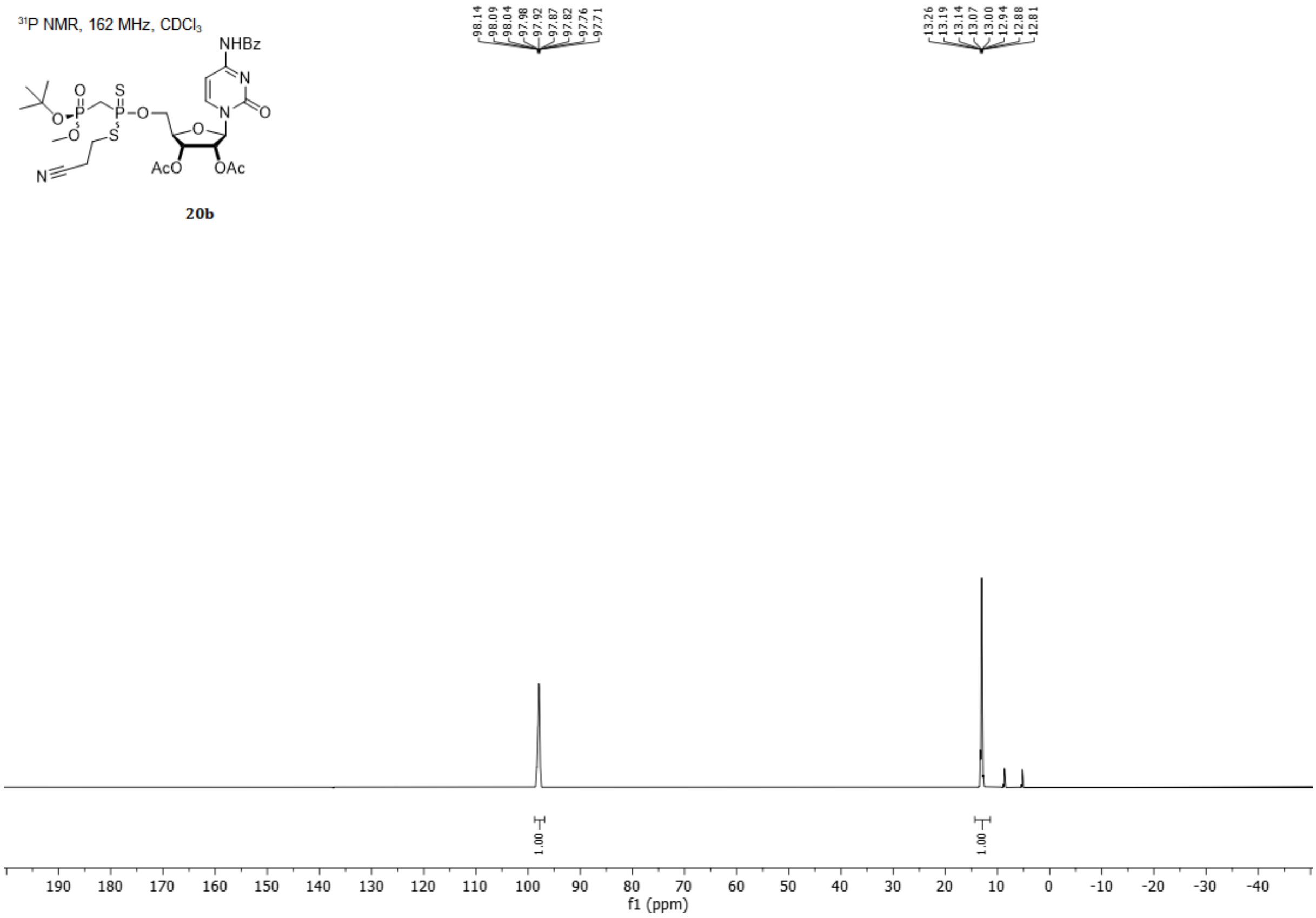
31P NMR, 162 MHz, CDCl3
NHBz
20b
98.14
98.09
98.04
97.98
97.92
97.87
97.82
97.76
97.71
13.26
13.19
13.14
13.07
13.00
12.94
12.88
12.81
1.00
1.00
f1 (ppm)

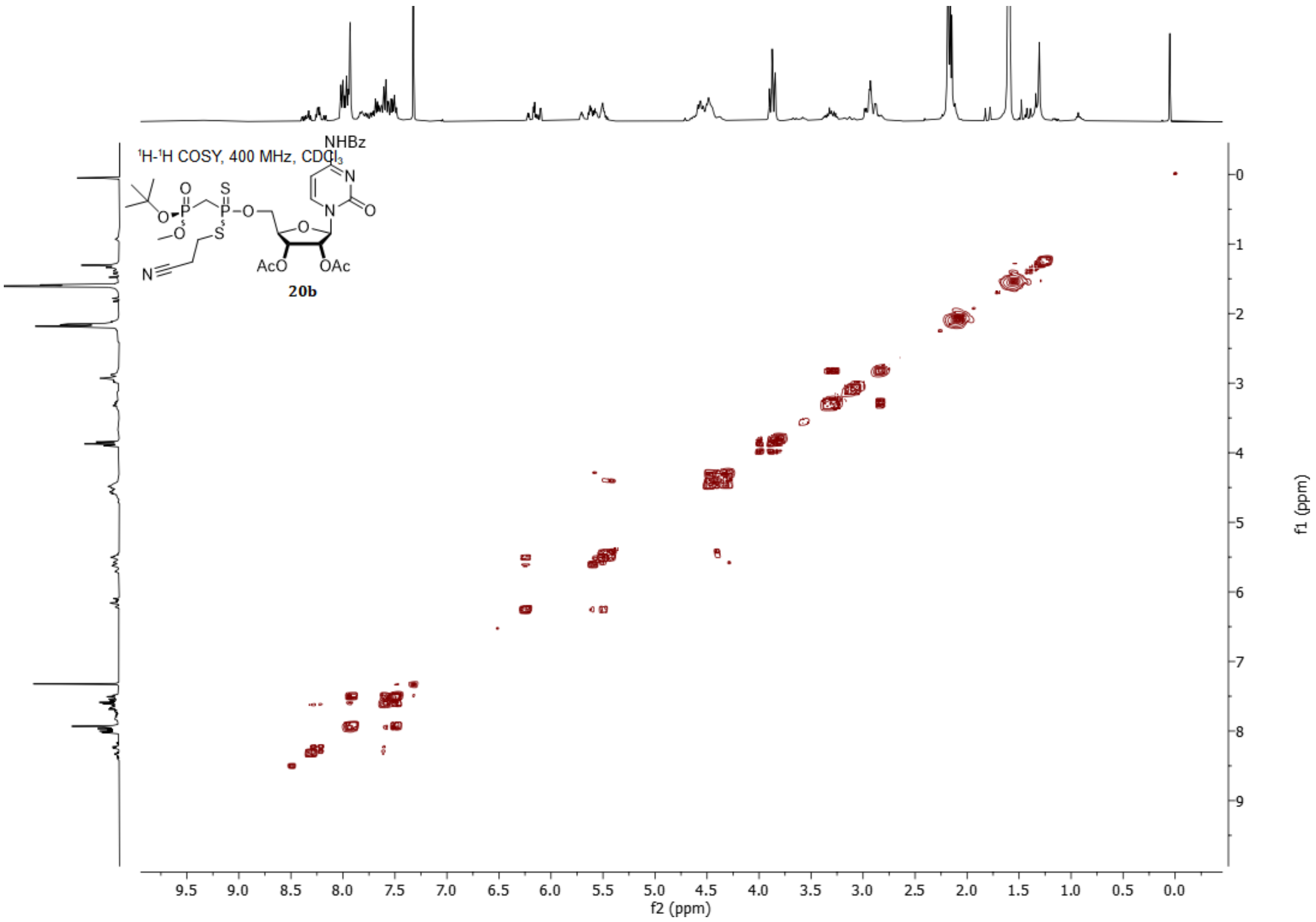

^{1}H-^{1}H COSY, 400 MHz, CDCl$_3$
NHBz
AcO
OAc
20b
f2 (ppm)
f1 (ppm)

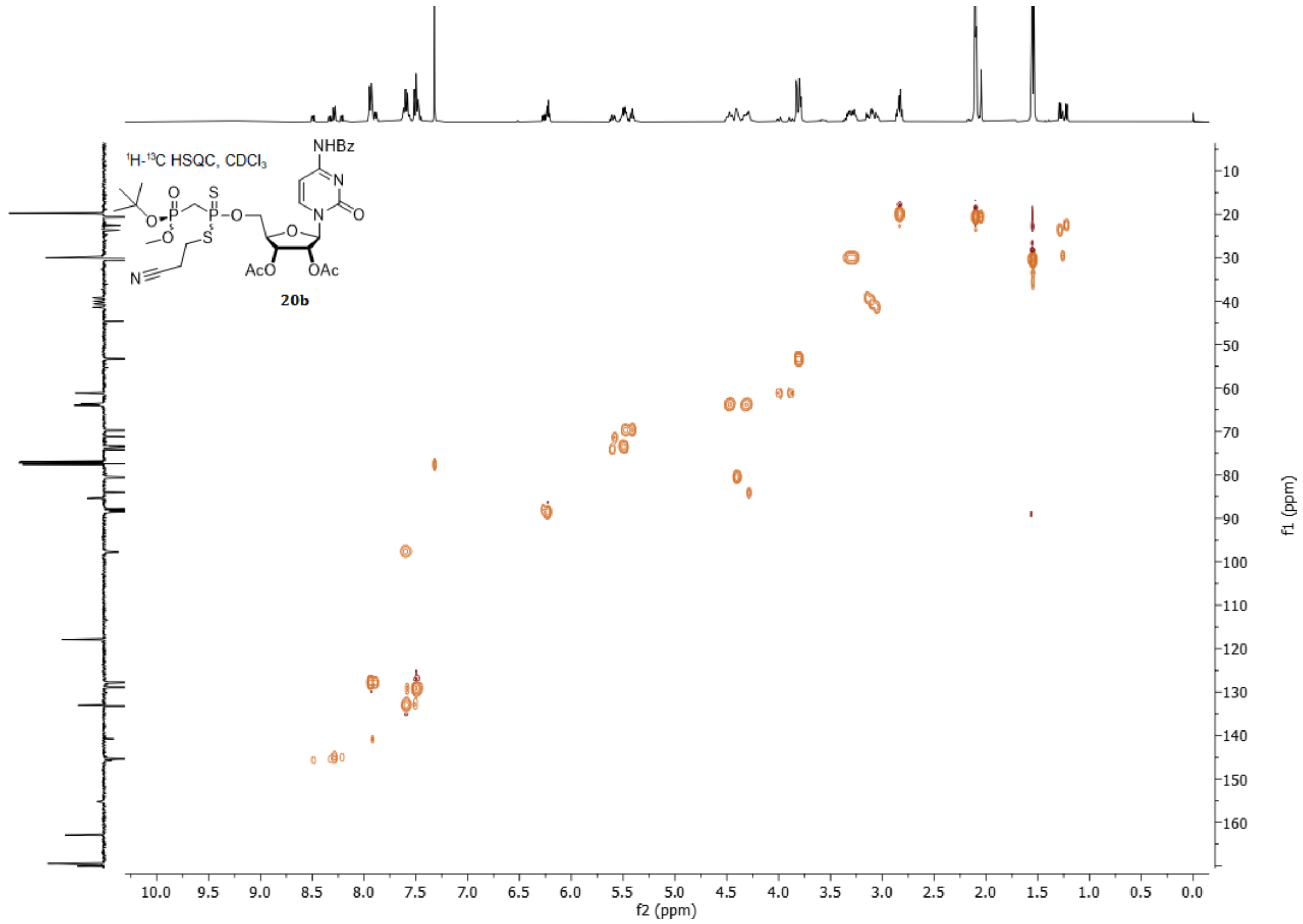

^{1}H-^{13}C HSQC, CDCl$_3$
NHBz
AcO
OAc
20b
f2 (ppm)
f1 (ppm)

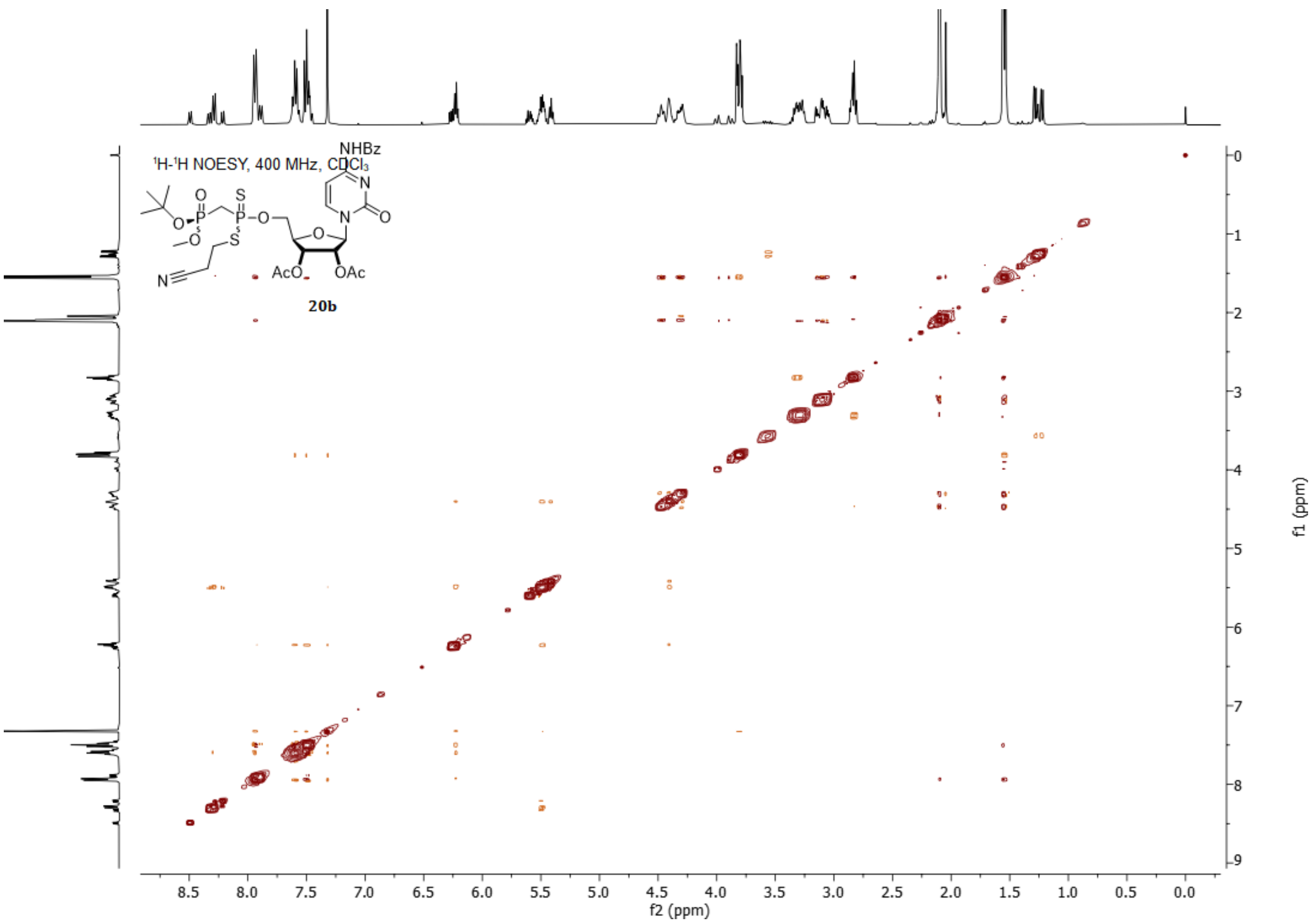

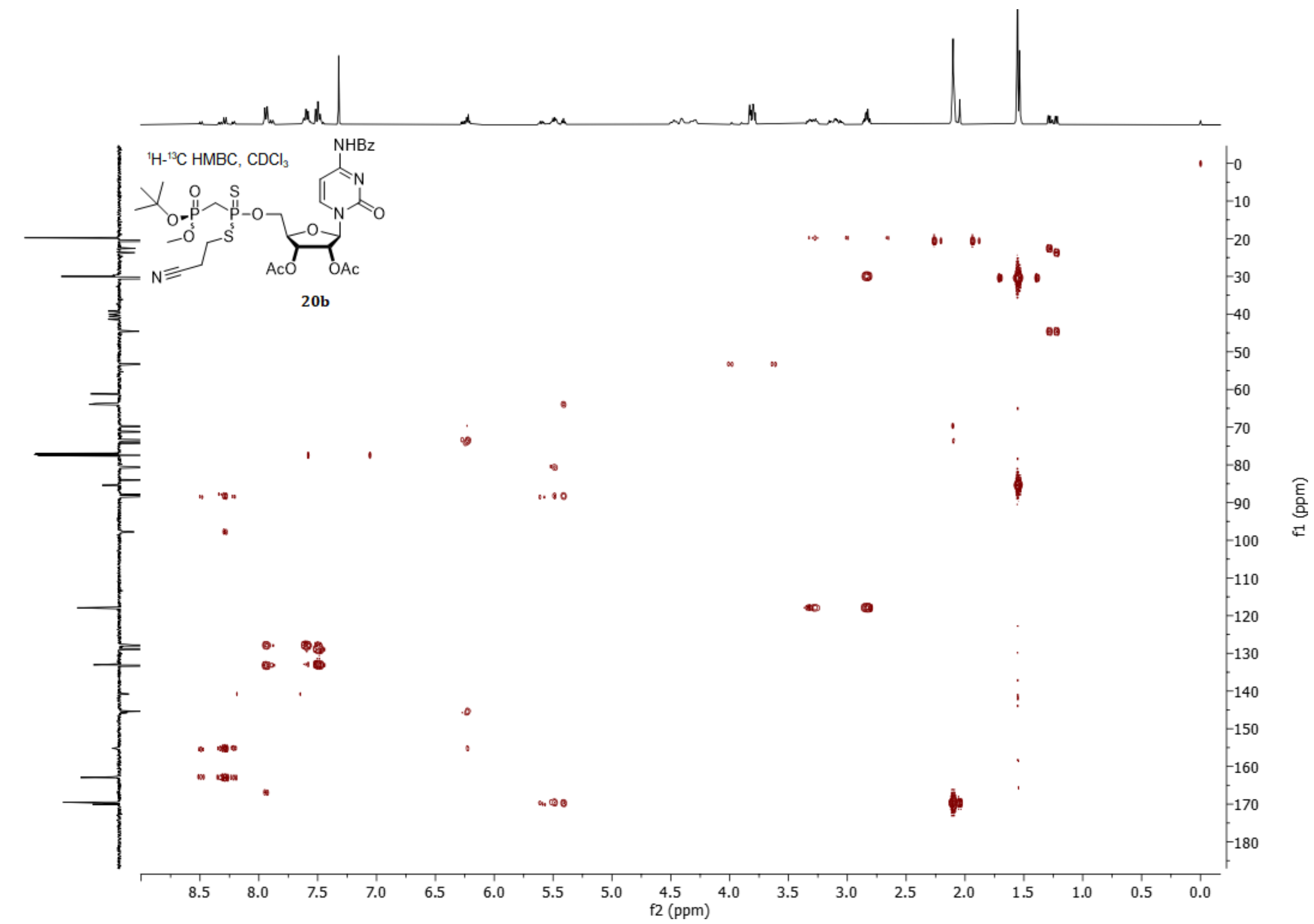

***sn*-3 me-CDP-αS-Gro tetraester 25a**

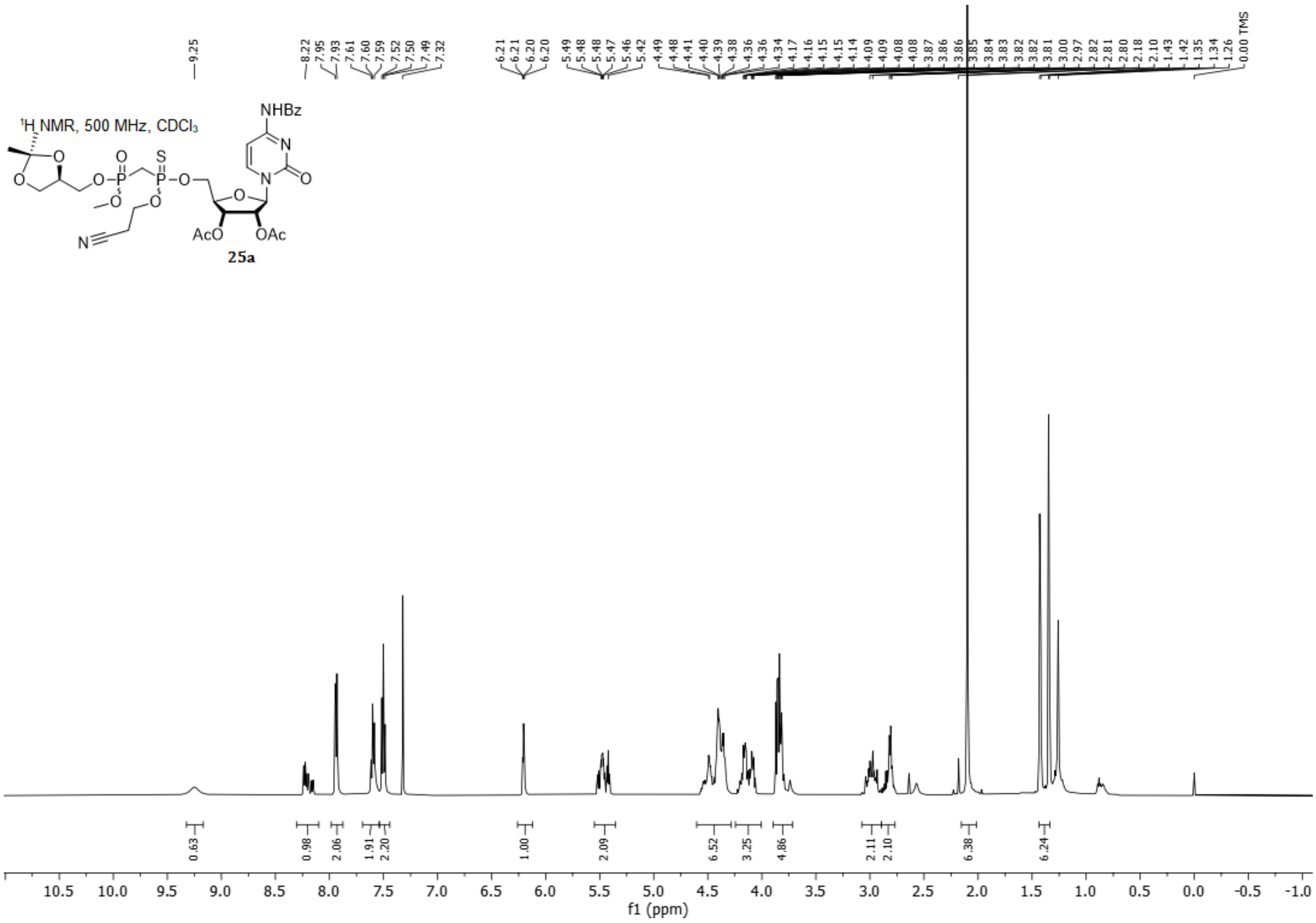

1H NMR, 500 MHz, CDCl3
NHBz
AcO
OAc
25a
f1 (ppm)

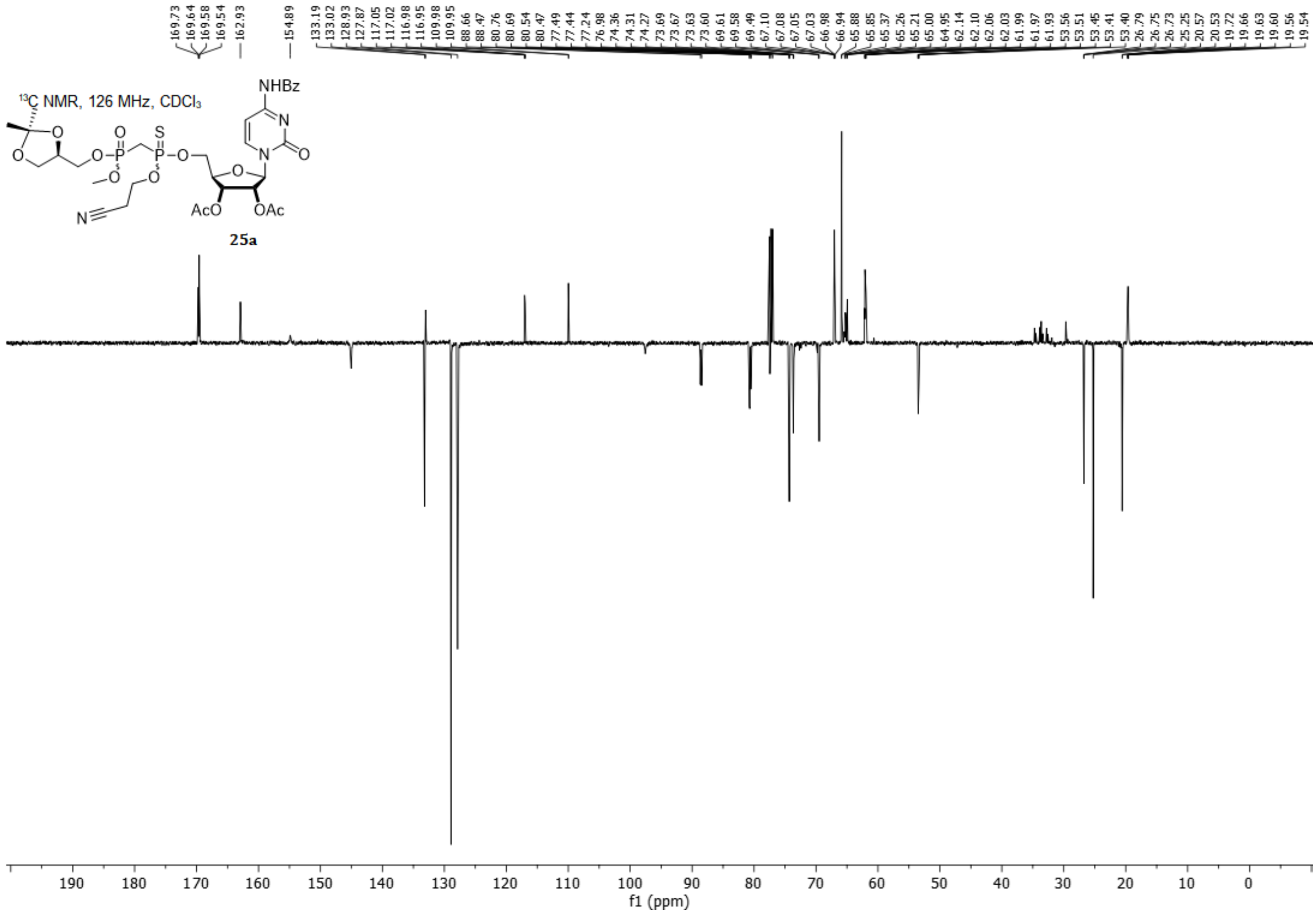

13C NMR, 126 MHz, CDCl3
NHBz
AcO
OAc
25a
f1 (ppm)

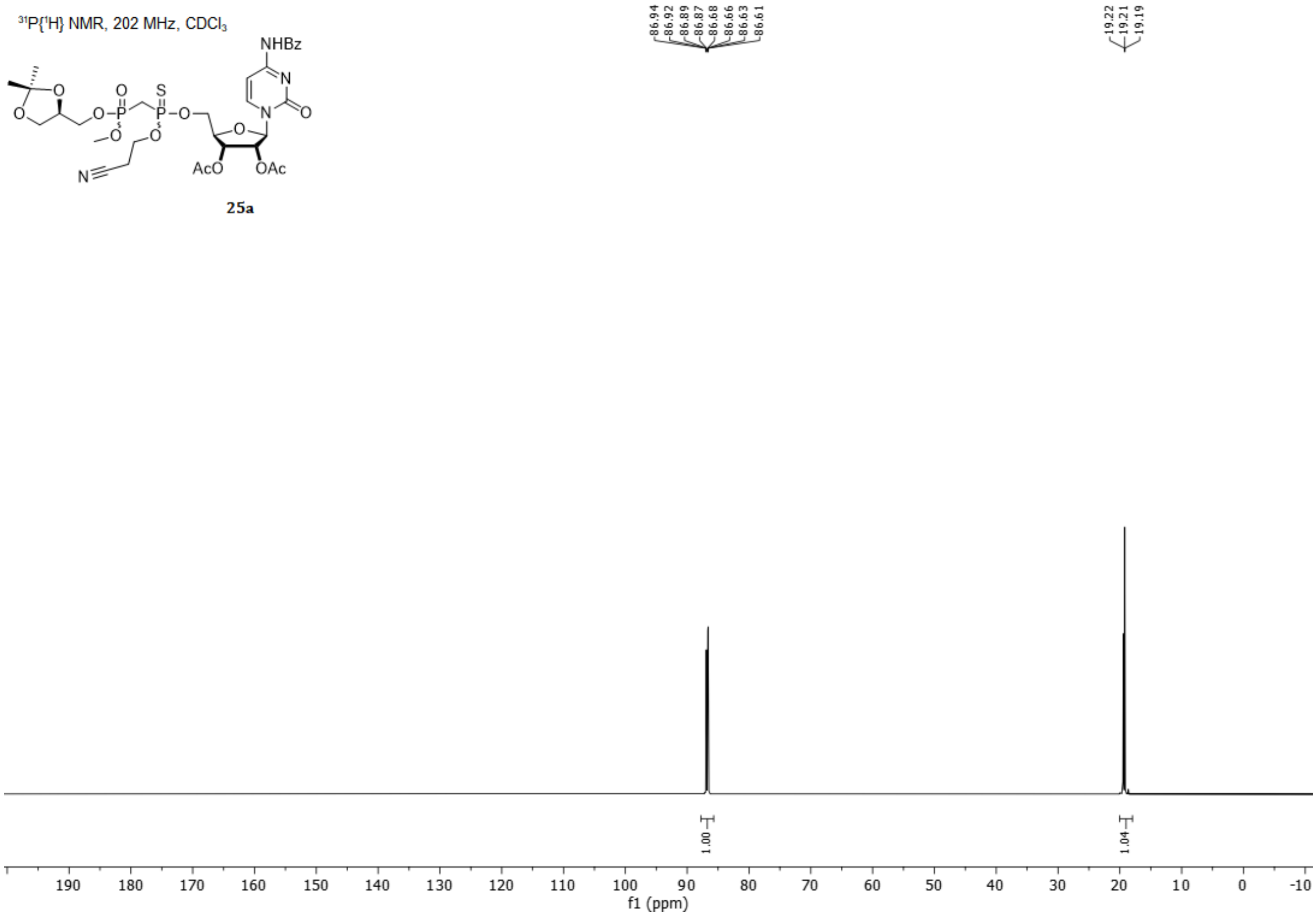

$^{31}P\{^{1}H\}$ NMR, 202 MHz, $CDCl_3$
NHBz
N
O
AcO
OAc
25a
86.94
86.92
86.89
86.87
86.68
86.66
86.63
86.61
19.22
19.21
19.19
1.00
1.04
190 180 170 160 150 140 130 120 110 100 90 80 70 60 50 40 30 20 10 0 -10
f1 (ppm)

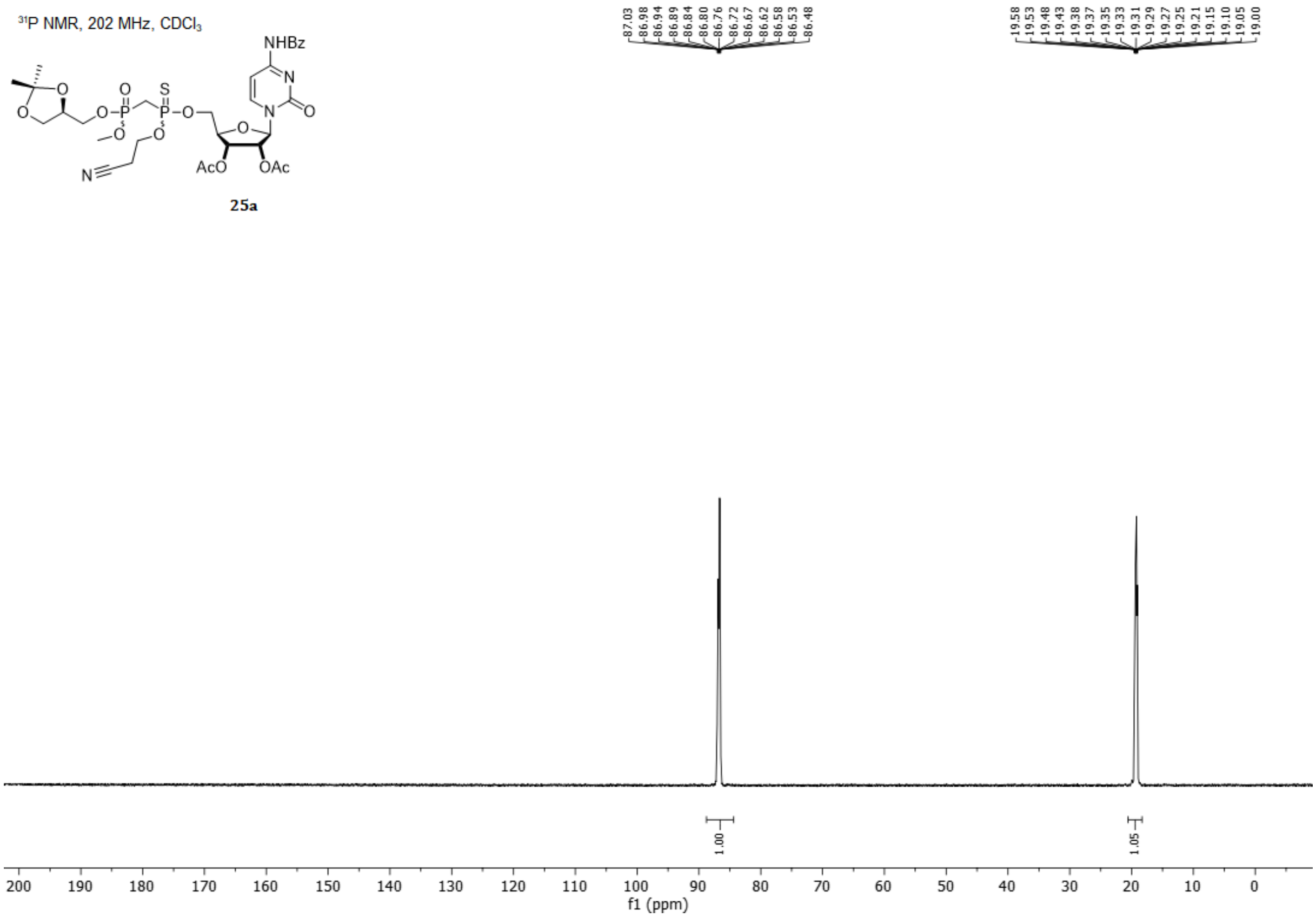

^{31}P NMR, 202 MHz, $CDCl_3$
NHBz
N
O
AcO
OAc
25a
87.03
86.98
86.94
86.89
86.84
86.80
86.76
86.72
86.67
86.62
86.58
86.53
86.48
19.58
19.53
19.48
19.43
19.38
19.37
19.35
19.33
19.31
19.29
19.27
19.25
19.21
19.15
19.10
19.05
19.00
1.00
1.05
200 190 180 170 160 150 140 130 120 110 100 90 80 70 60 50 40 30 20 10 0
f1 (ppm)

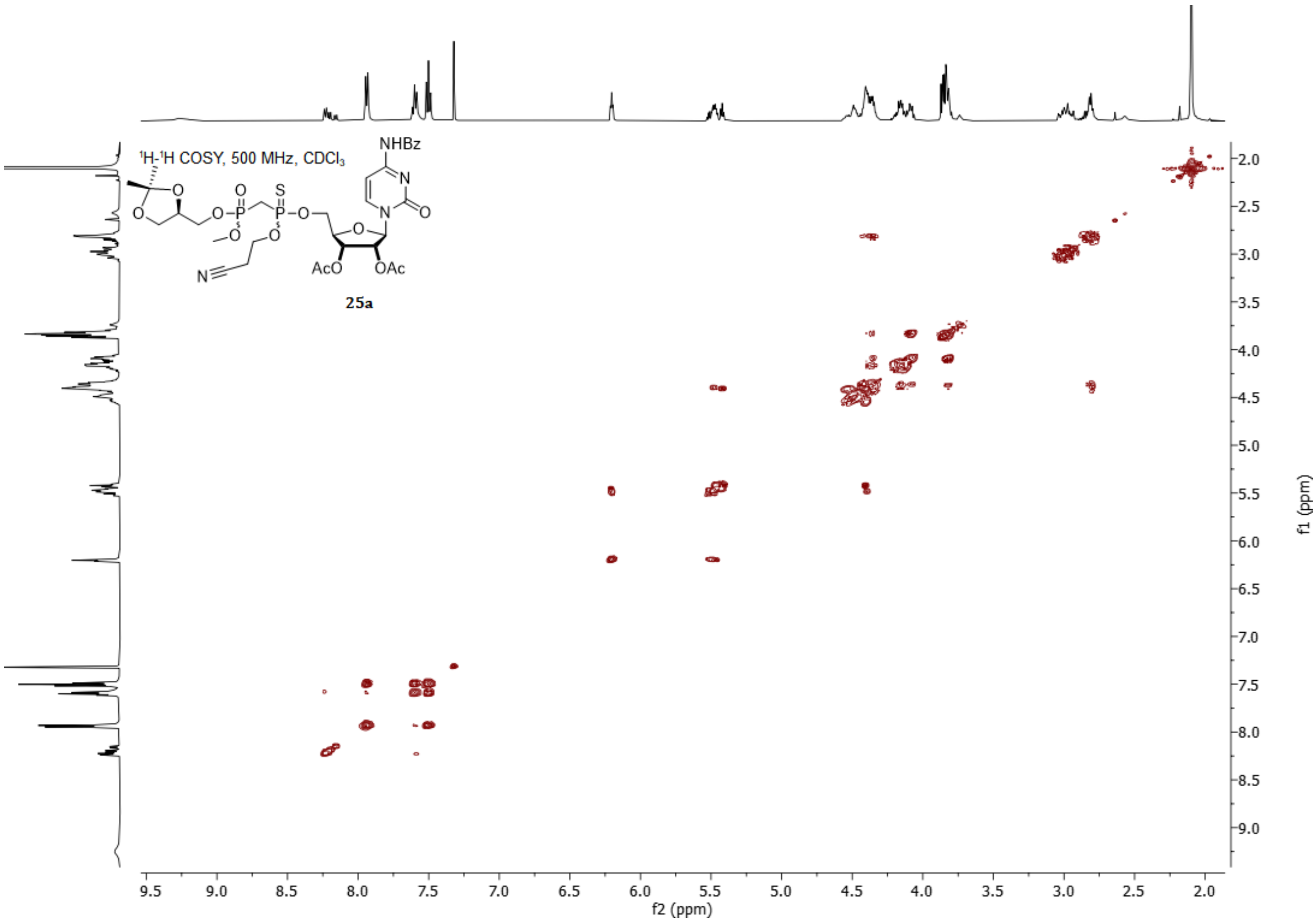

¹H-¹H COSY, 500 MHz, CDCl₃
NHBz
AcO
OAc
N
25a
f2 (ppm)
f1 (ppm)

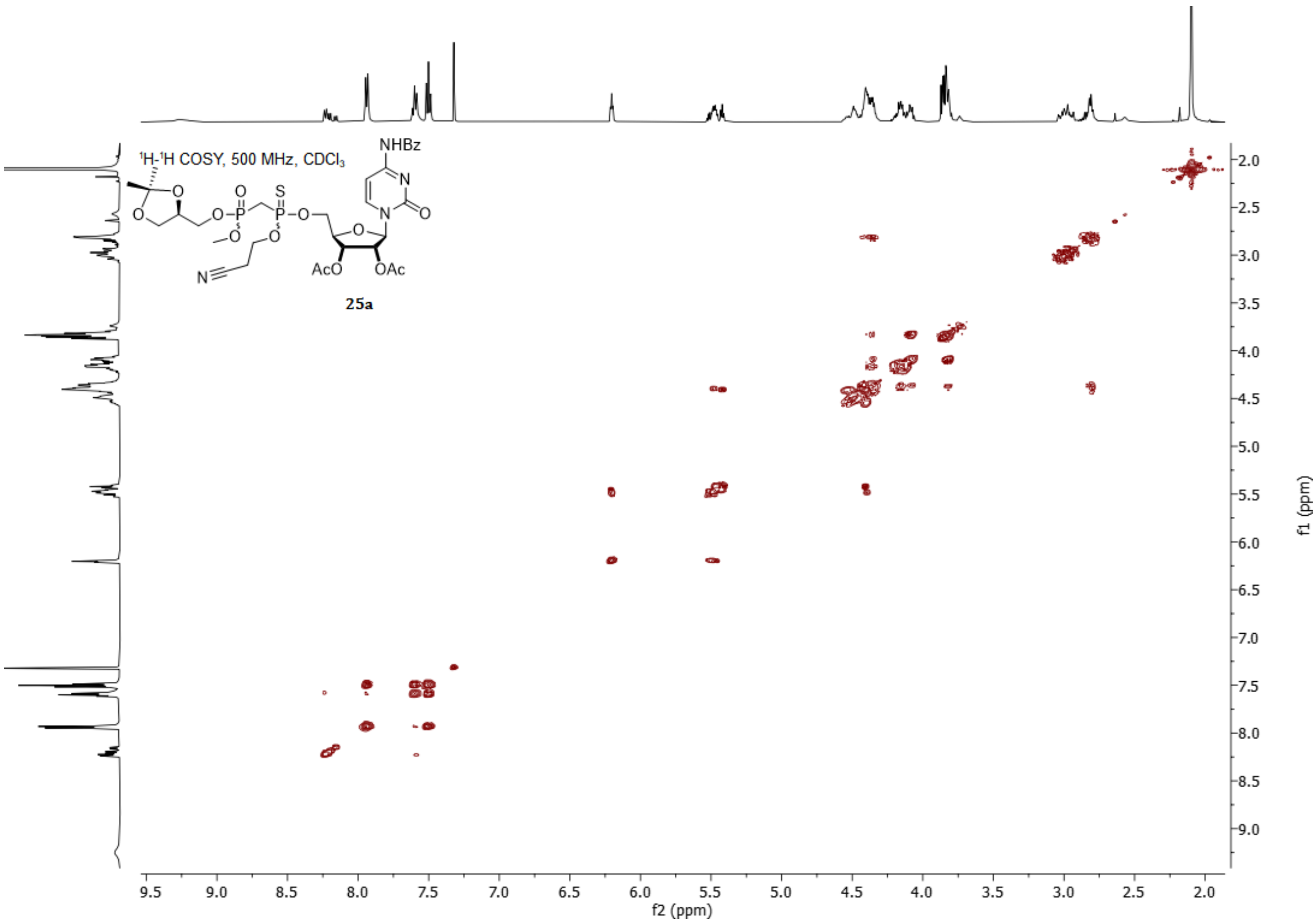

¹H-¹H COSY, 500 MHz, CDCl₃
NHBz
AcO
OAc
N
25a
f2 (ppm)
f1 (ppm)

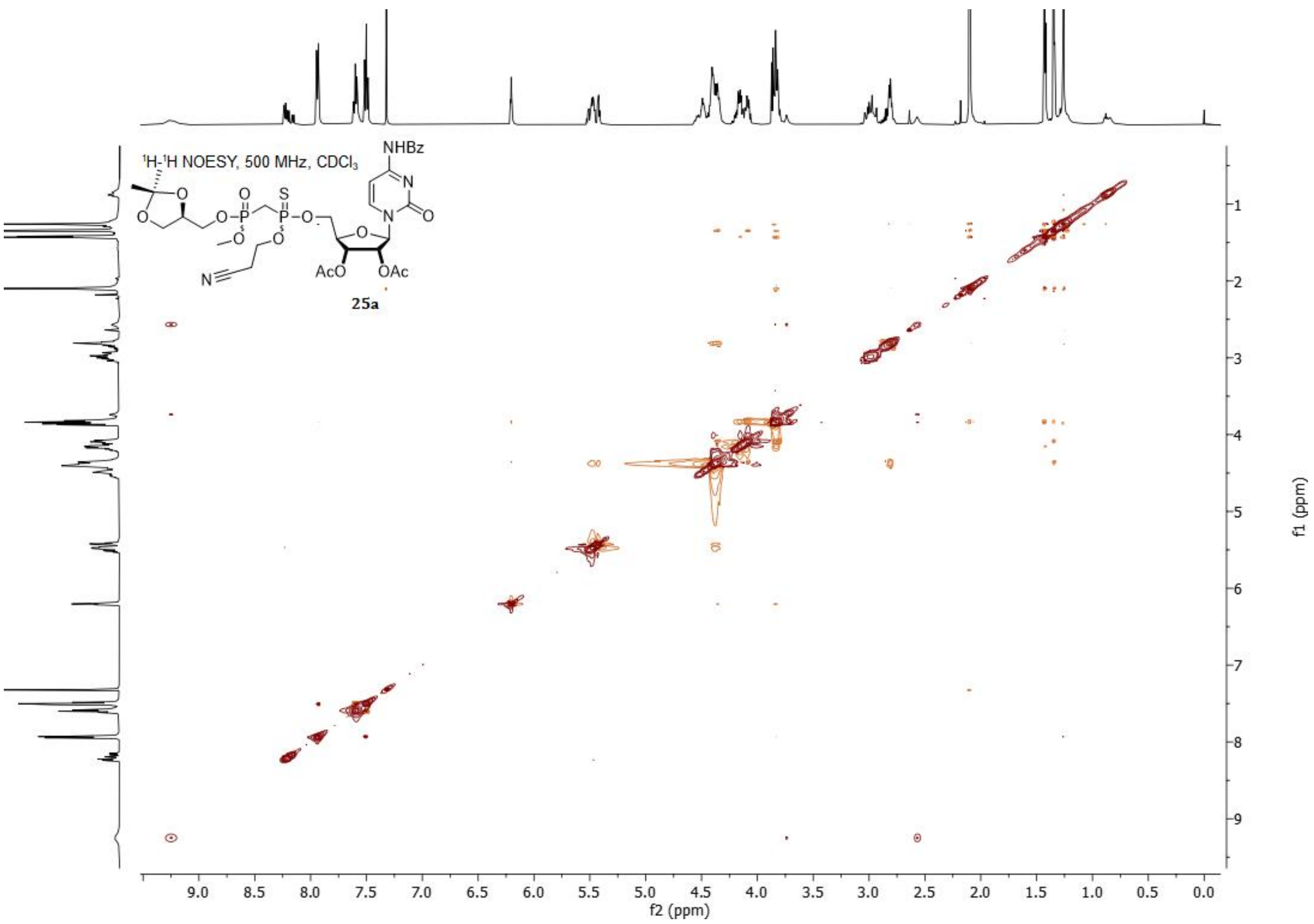

1H-1H NOESY, 500 MHz, CDCl3
NHBz
25a
f2 (ppm)
f1 (ppm)

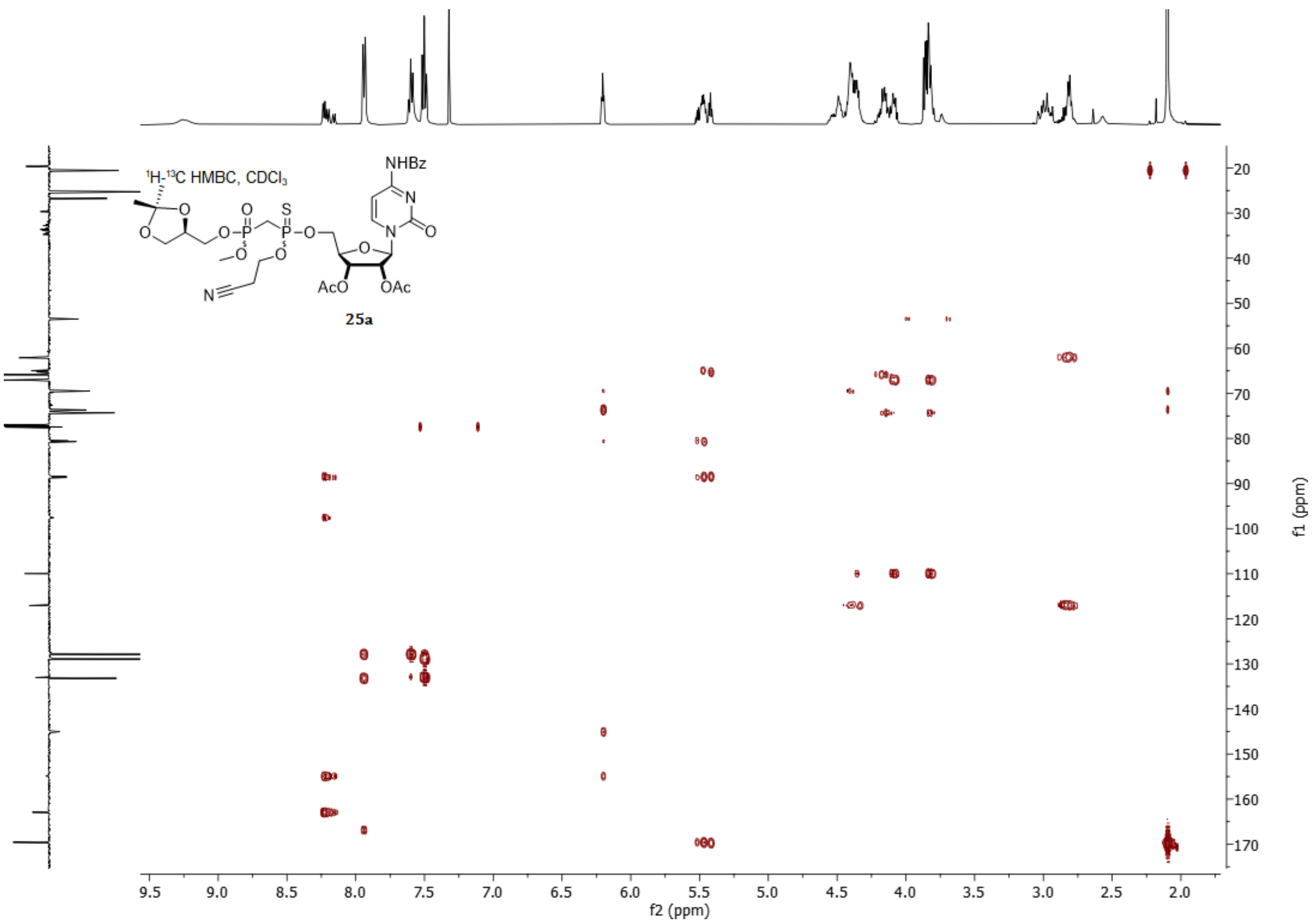

1H-13C HMBC, CDCl3
NHBz
25a
f2 (ppm)
f1 (ppm)

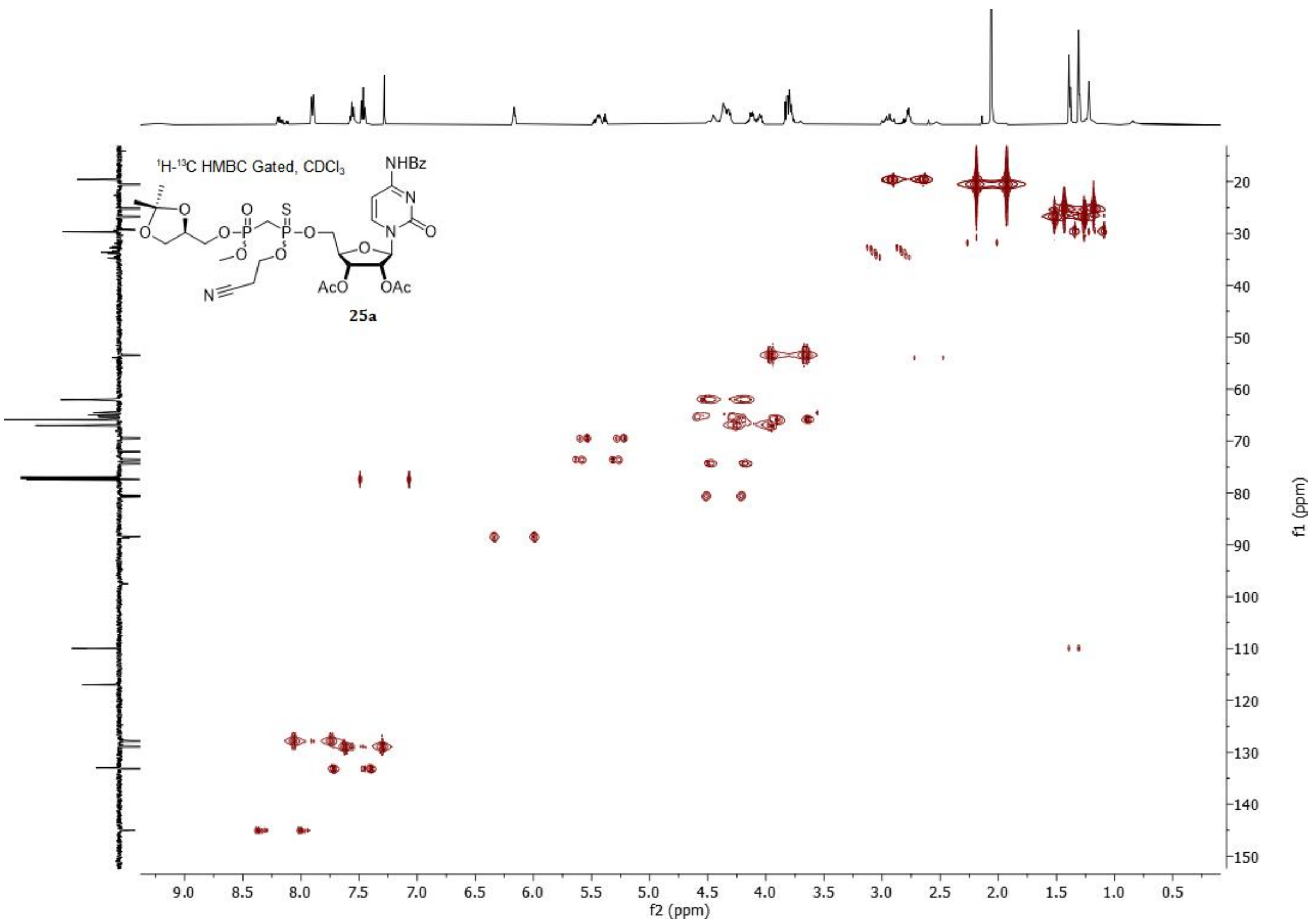

sn-3 me-CDP-αS$_2$-Gro tetraester 25b

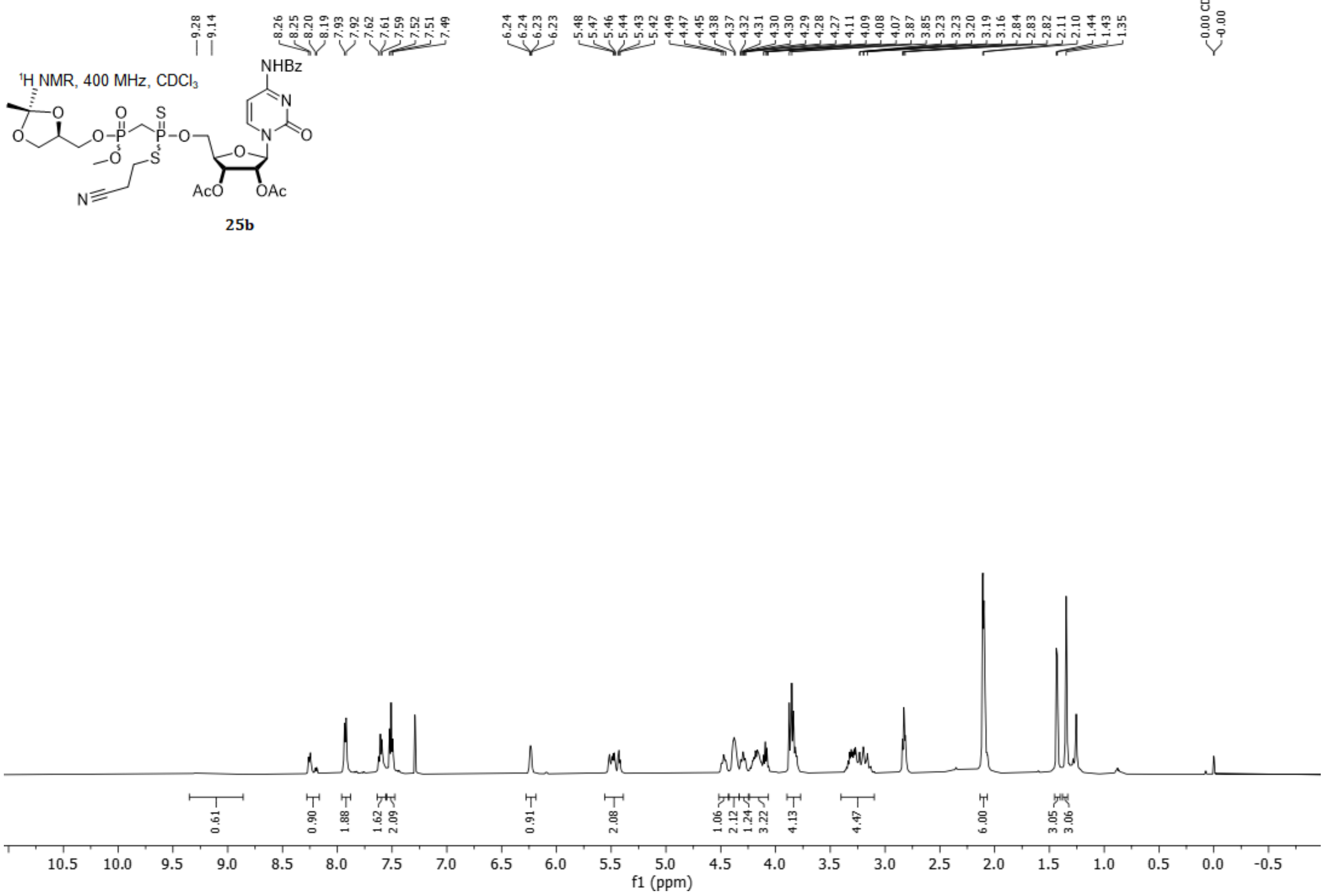

¹H NMR, 400 MHz, CDCl₃
NHBz
25b
9.28 9.14 8.26 8.25 8.20 8.19 7.93 7.92 7.62 7.61 7.59 7.52 7.51 7.49 6.24 6.24 6.23 6.23 5.48 5.47 5.46 5.44 5.43 5.42 4.49 4.47 4.45 4.38 4.37 4.32 4.31 4.30 4.30 4.29 4.28 4.27 4.11 4.09 4.08 4.07 3.87 3.85 3.23 3.23 3.20 3.19 3.16 2.84 2.83 2.82 2.11 2.10 1.44 1.43 1.35
0.00 CDCl3 -0.00
0.61 0.90 1.88 1.62 2.09 0.91 2.08 1.06 2.12 1.24 3.22 4.13 4.47 6.00 3.05 3.06
f1 (ppm)

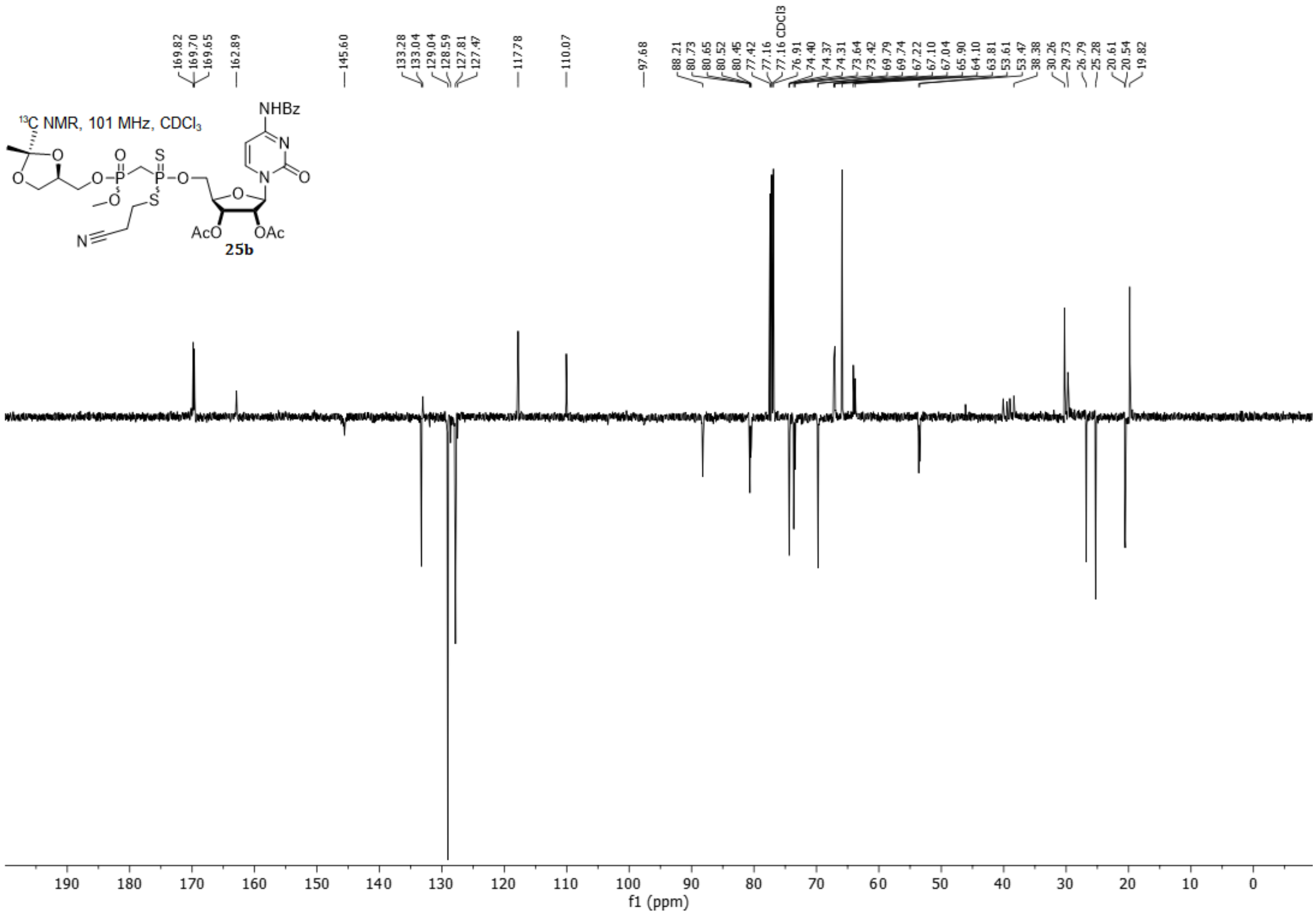

¹³C NMR, 101 MHz, CDCl₃
NHBz
25b
169.82 169.70 169.65 162.89 145.60 133.28 133.04 129.04 128.59 127.81 127.47 117.78 110.07 97.68 88.21 80.73 80.65 80.52 80.45 77.42 77.16 77.16 CDCl3 76.91 74.40 74.37 74.31 73.64 73.42 69.79 69.74 67.22 67.10 67.04 65.90 64.10 63.81 53.61 53.47 38.38 30.26 29.73 26.79 25.28 20.61 20.54 19.82
f1 (ppm)

$^{31}P\{^{1}H\}$ NMR, 162 MHz, $CDCl_3$

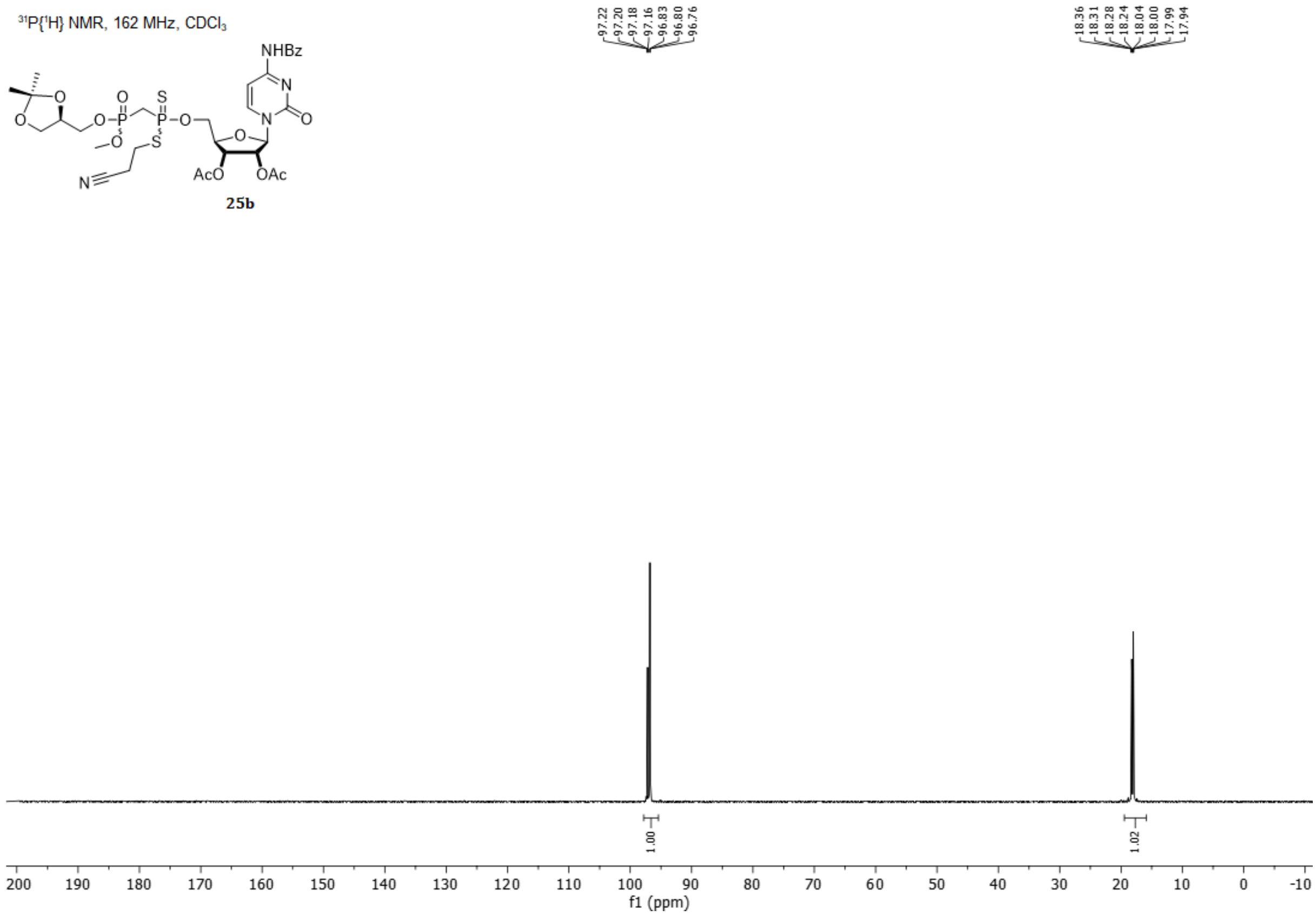

^{31}P NMR, 162 MHz, $CDCl_3$

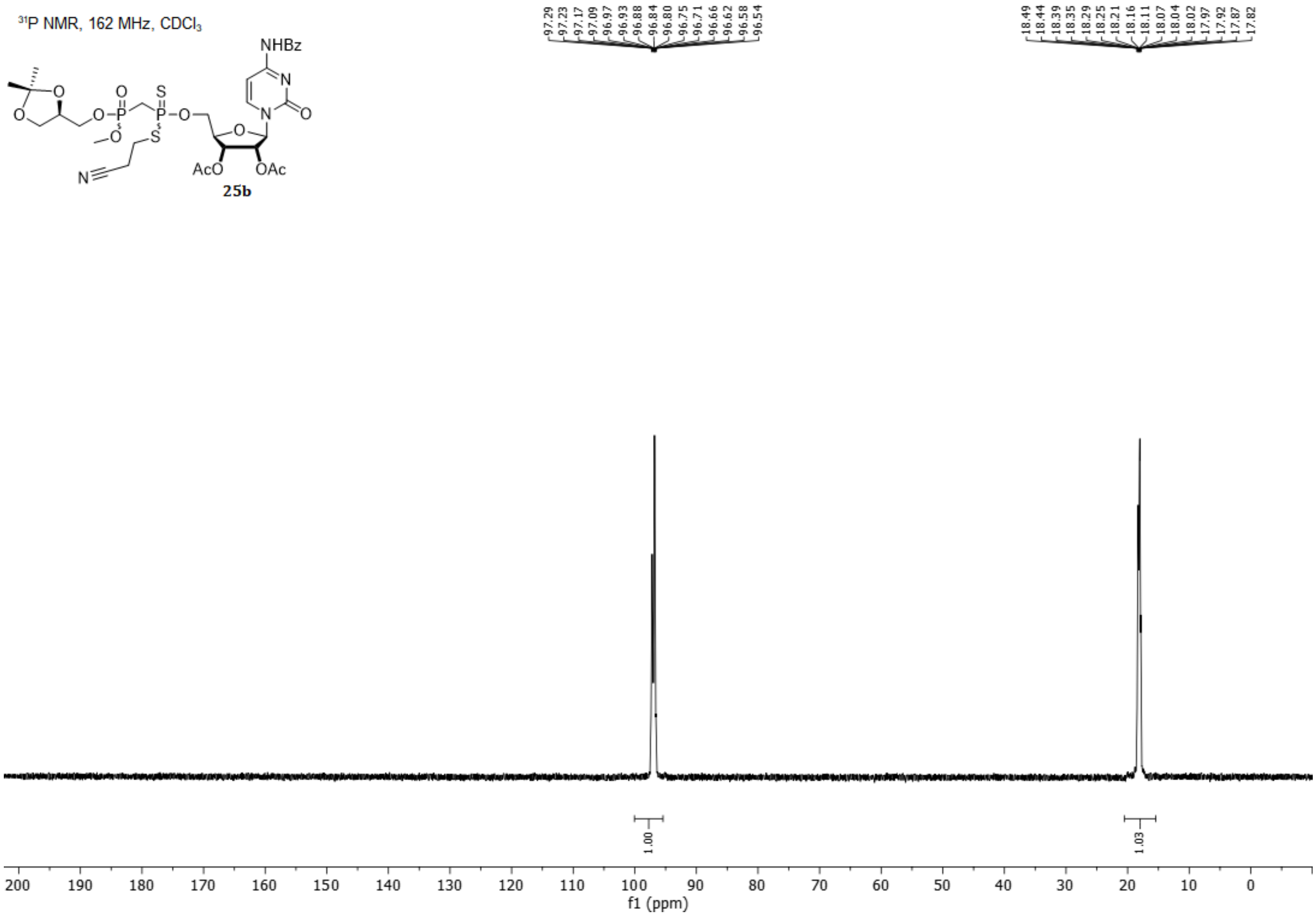

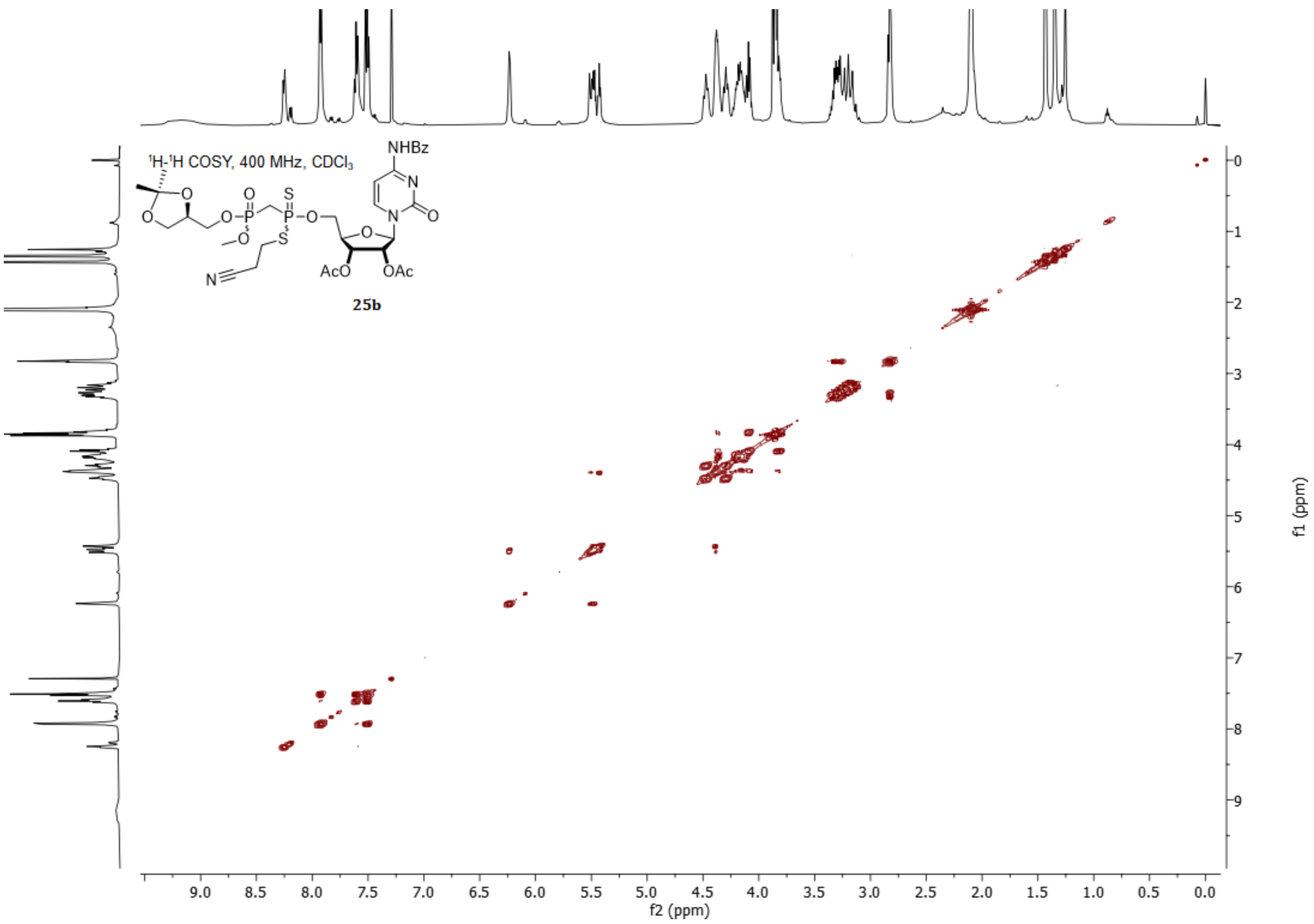
1H-1H COSY, 400 MHz, CDCl3
NHBz
N
O
S
P
AcO
OAc
N
25b
f2 (ppm)
f1 (ppm)

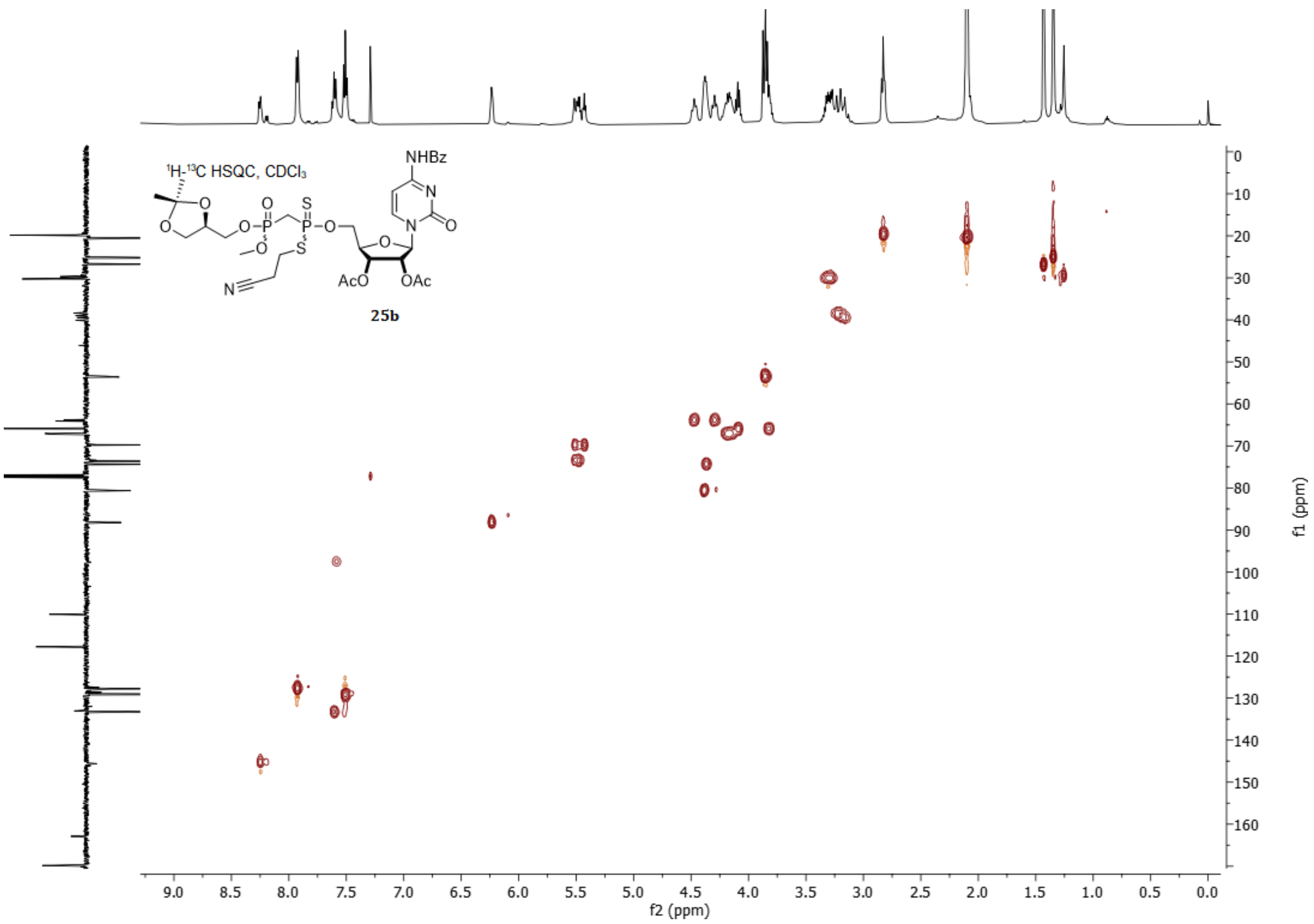
1H-13C HSQC, CDCl3
NHBz
N
O
S
P
AcO
OAc
N
25b
f2 (ppm)
f1 (ppm)

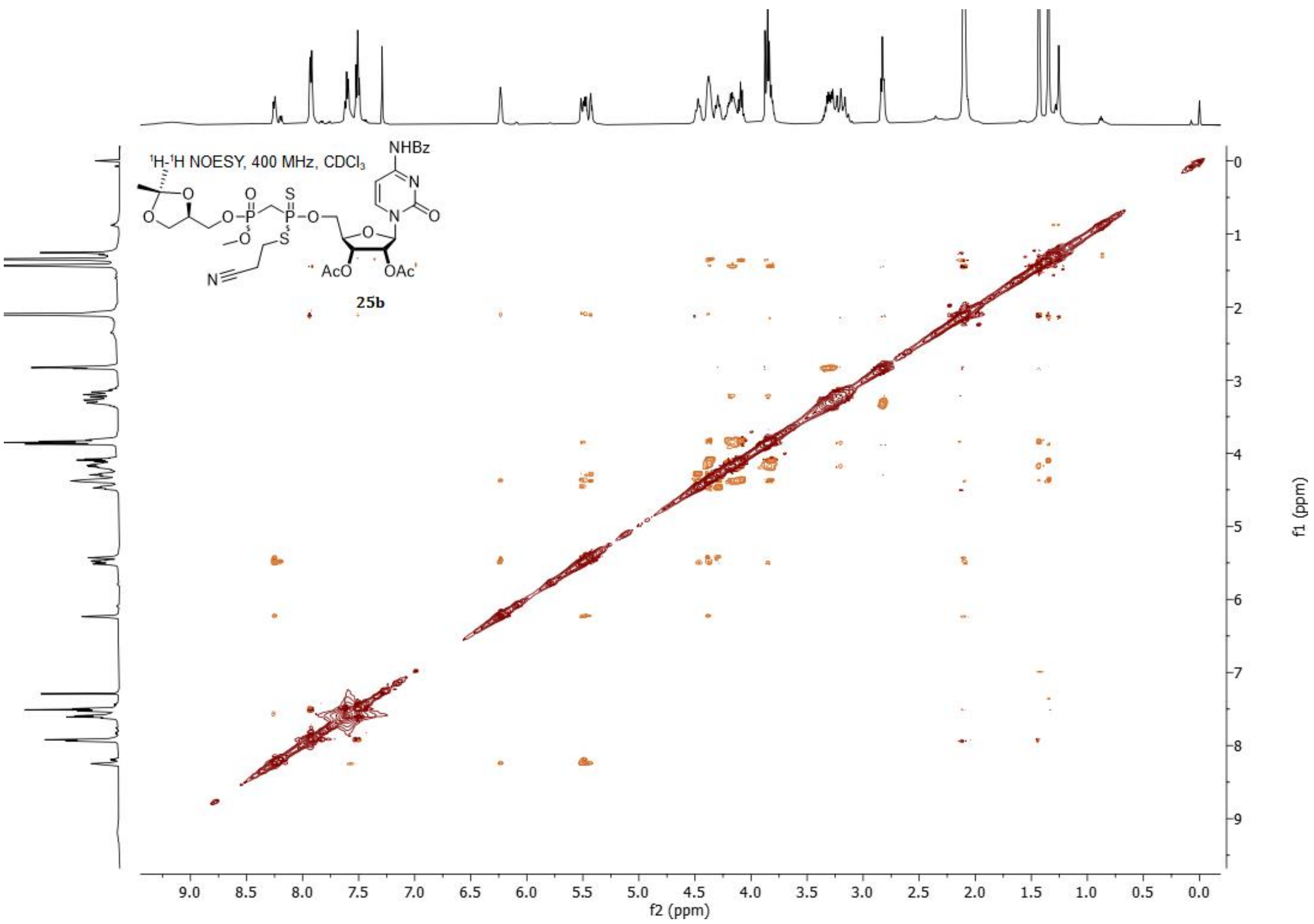
1H-1H NOESY, 400 MHz, CDCl3
NHBz
AcO
OAc
25b
f2 (ppm)
f1 (ppm)

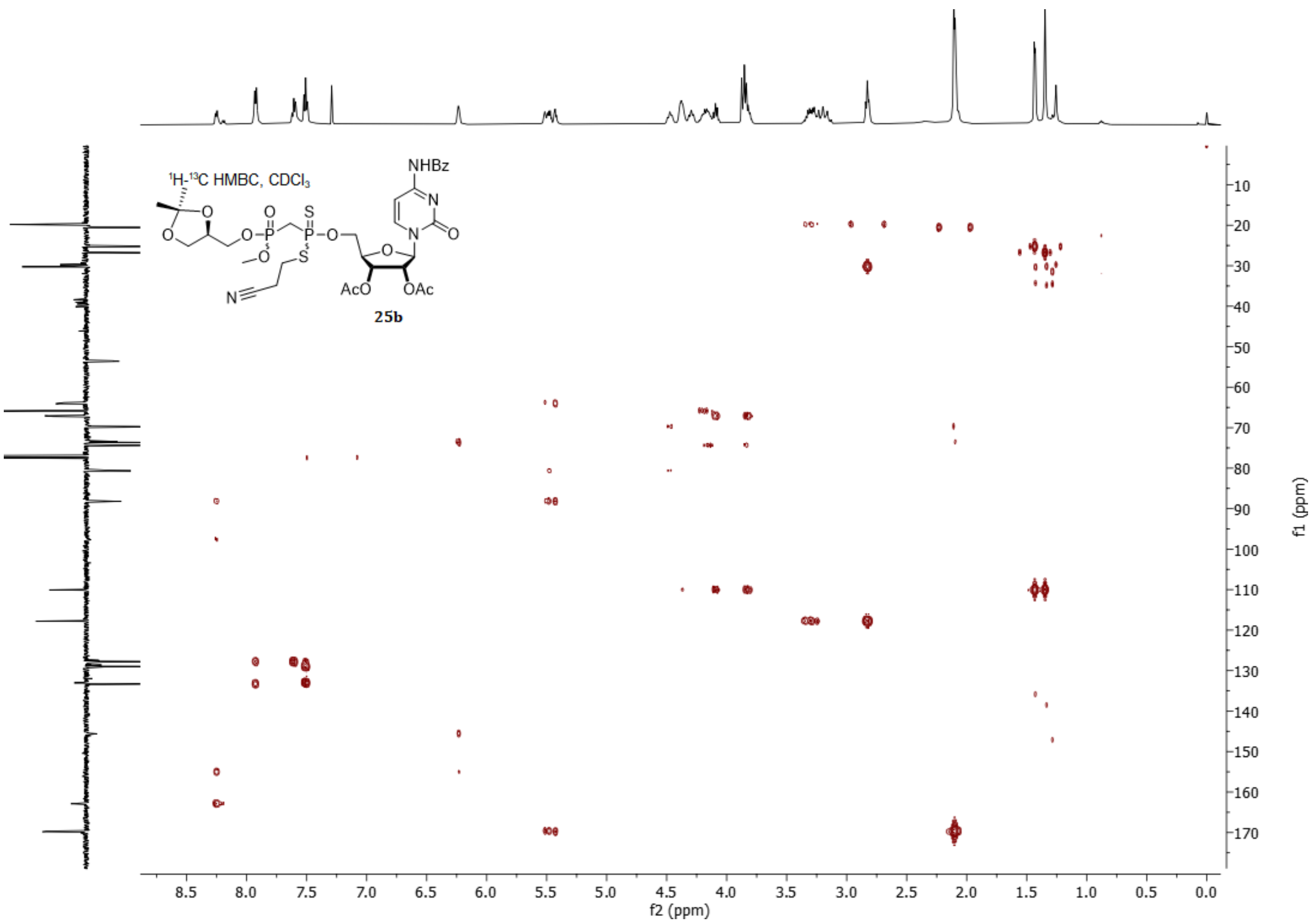
1H-13C HMBC, CDCl3
NHBz
AcO
OAc
25b
f2 (ppm)
f1 (ppm)

sn-1 me-CDP-αS-Gro tetraester 26a

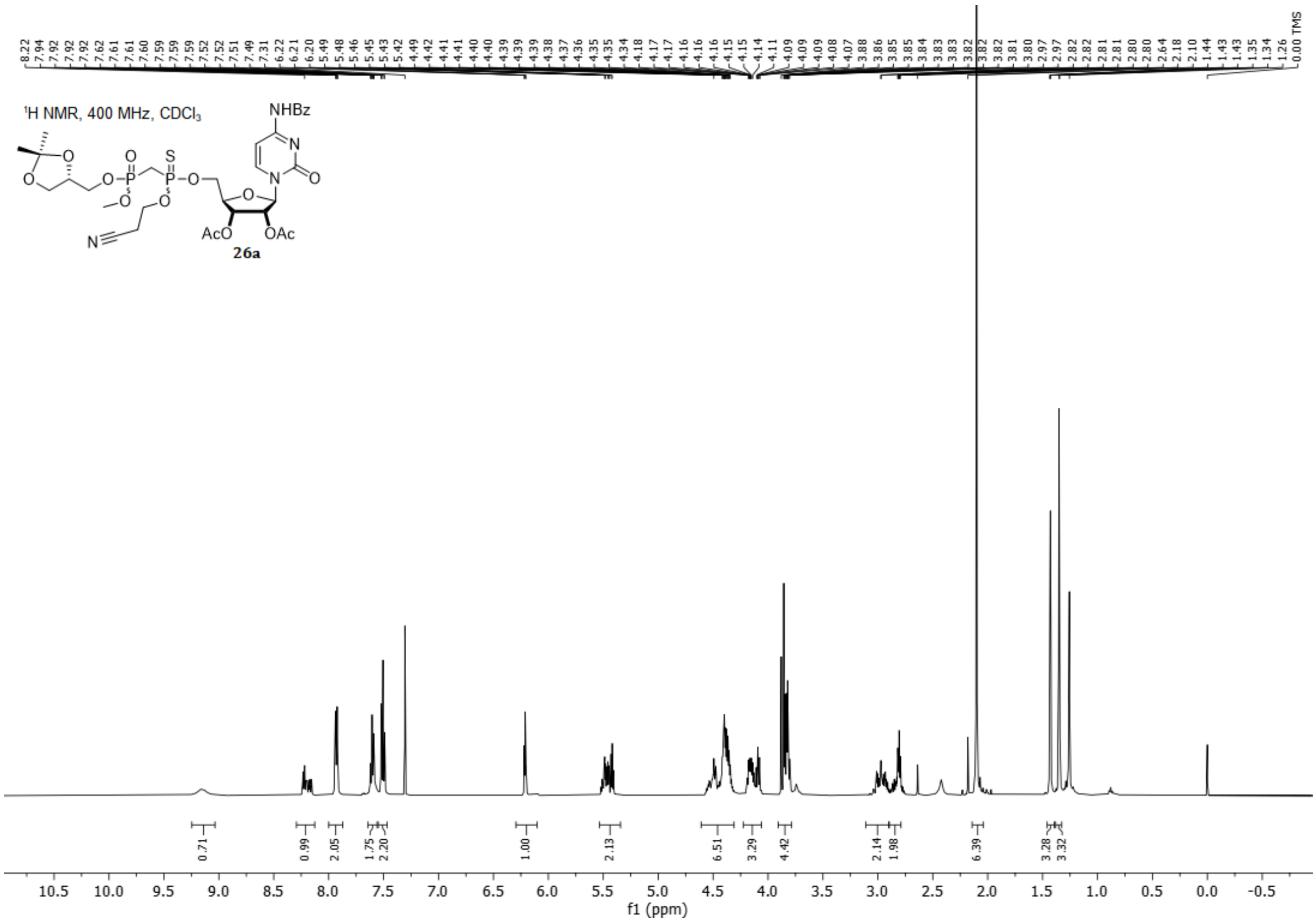

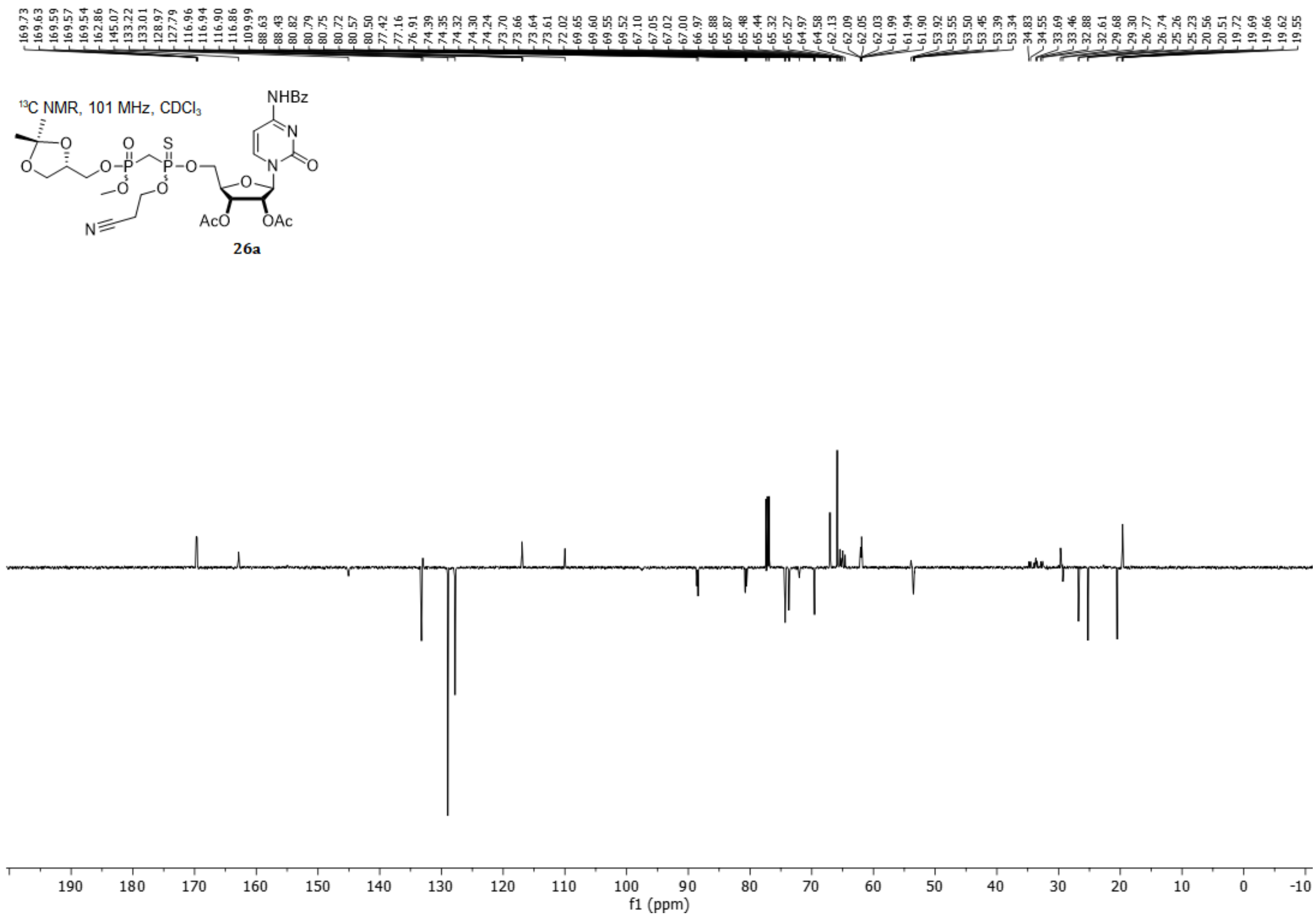

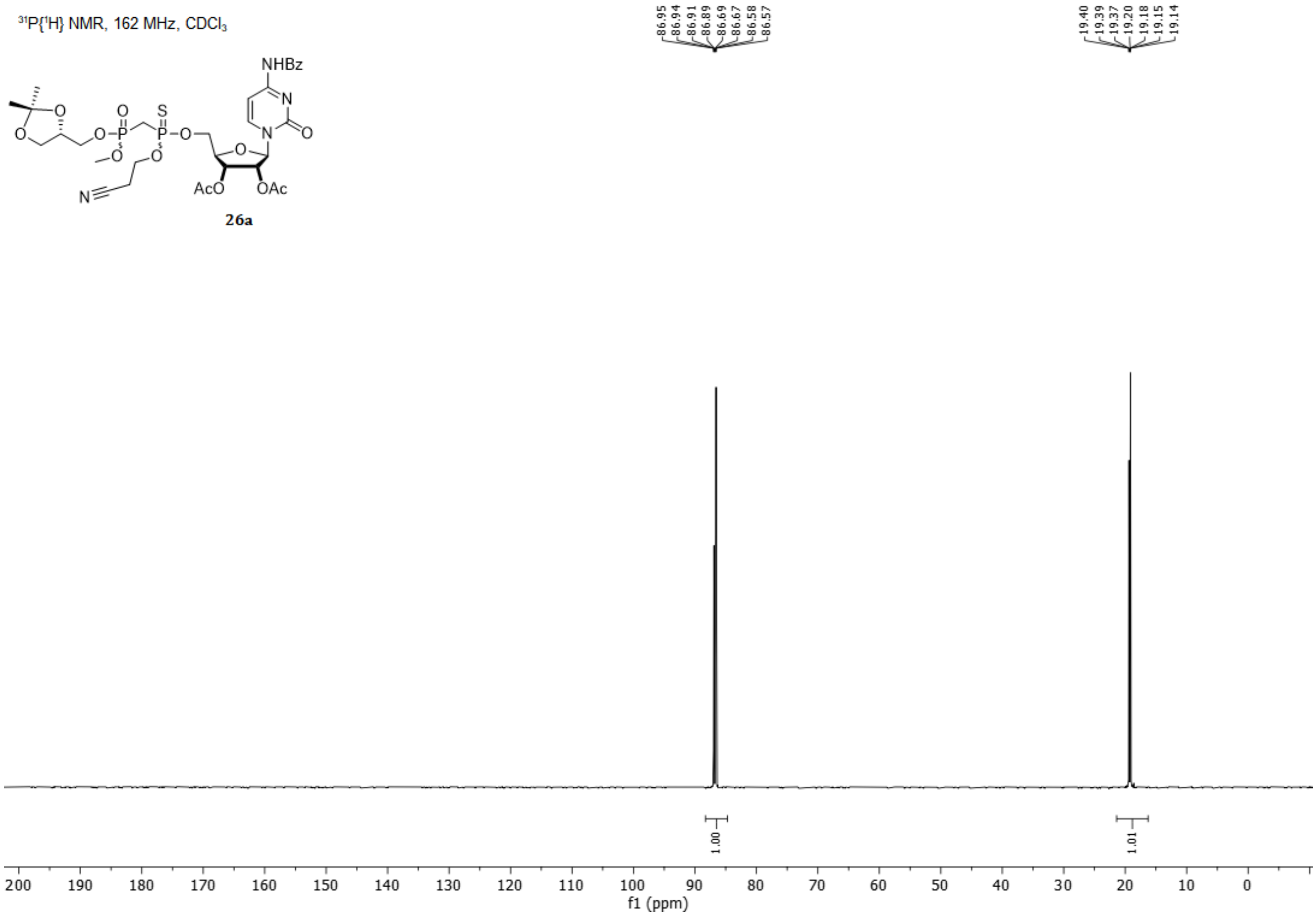

$^{31}P\{^1H\}$ NMR, 162 MHz, $CDCl_3$
NHBz
26a
86.95 86.94 86.91 86.89 86.69 86.67 86.58 86.57
19.40 19.39 19.37 19.20 19.18 19.15 19.14
1.00
1.01
f1 (ppm)

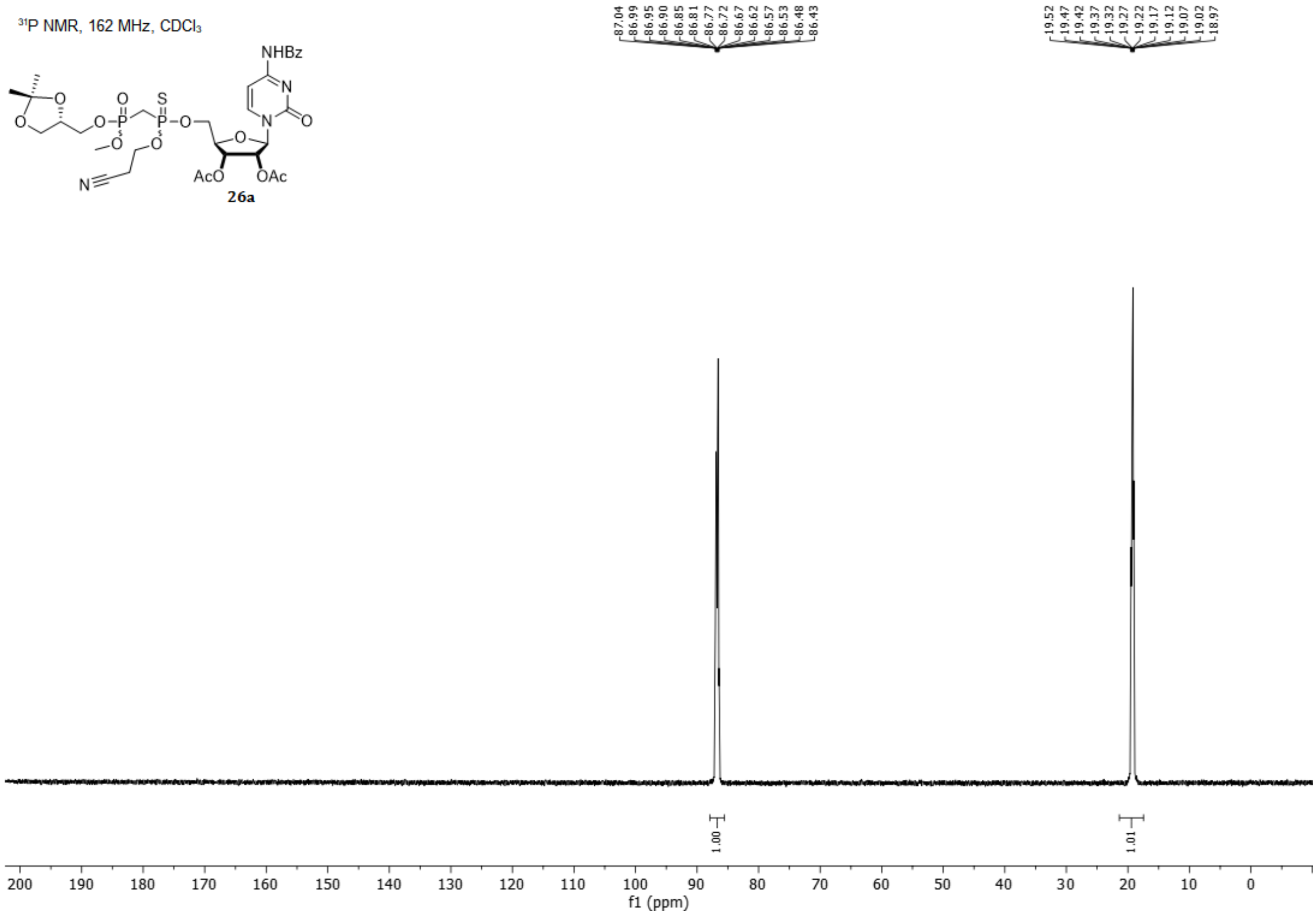

^{31}P NMR, 162 MHz, $CDCl_3$
NHBz
26a
87.04 86.99 86.95 86.90 86.85 86.81 86.77 86.72 86.67 86.62 86.57 86.53 86.48 86.43
19.52 19.47 19.42 19.37 19.32 19.27 19.22 19.17 19.12 19.07 19.02 18.97
1.00
1.01
f1 (ppm)

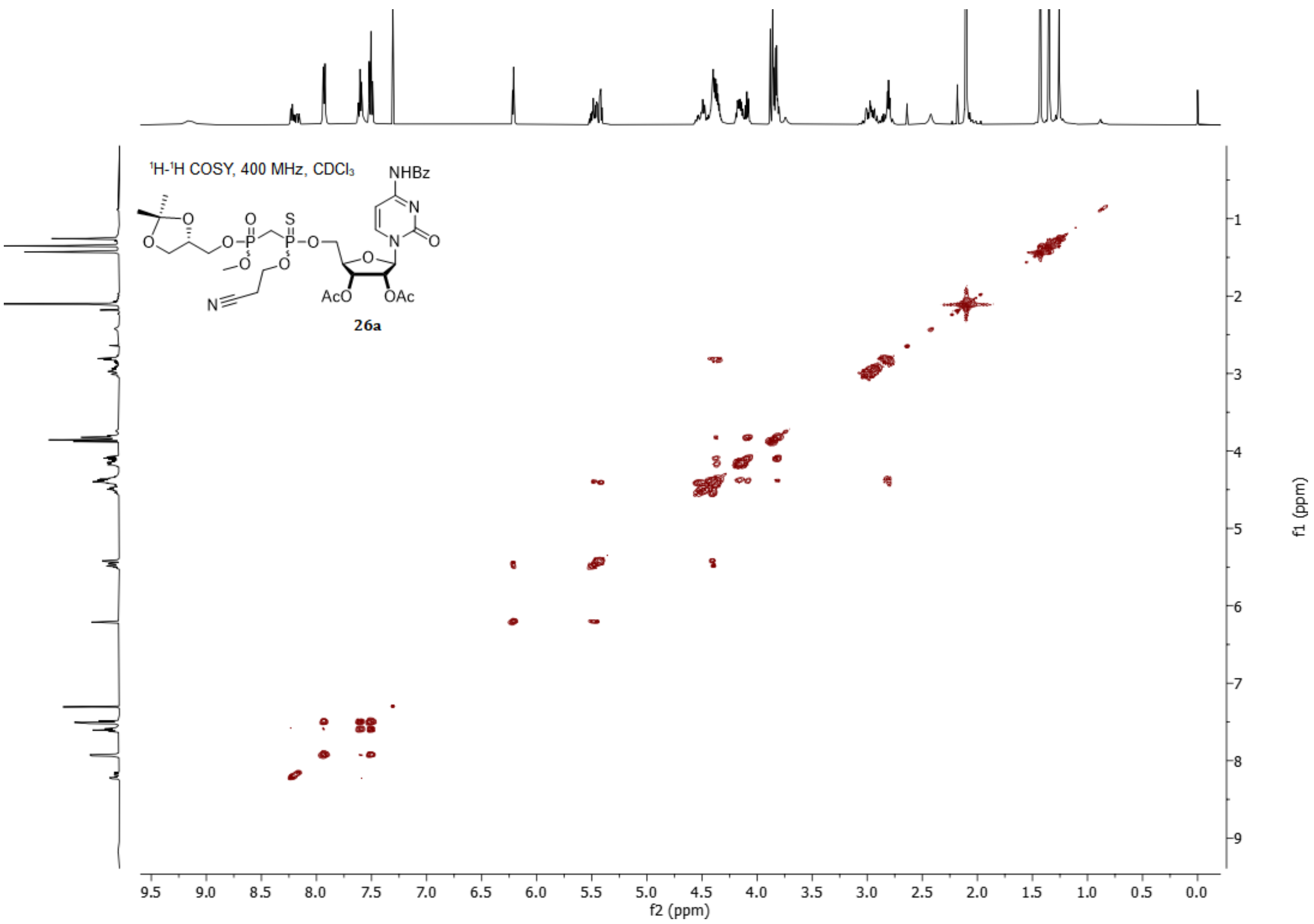

¹H-¹H COSY, 400 MHz, CDCl₃
NHBz
AcO
OAc
26a
f2 (ppm)
f1 (ppm)

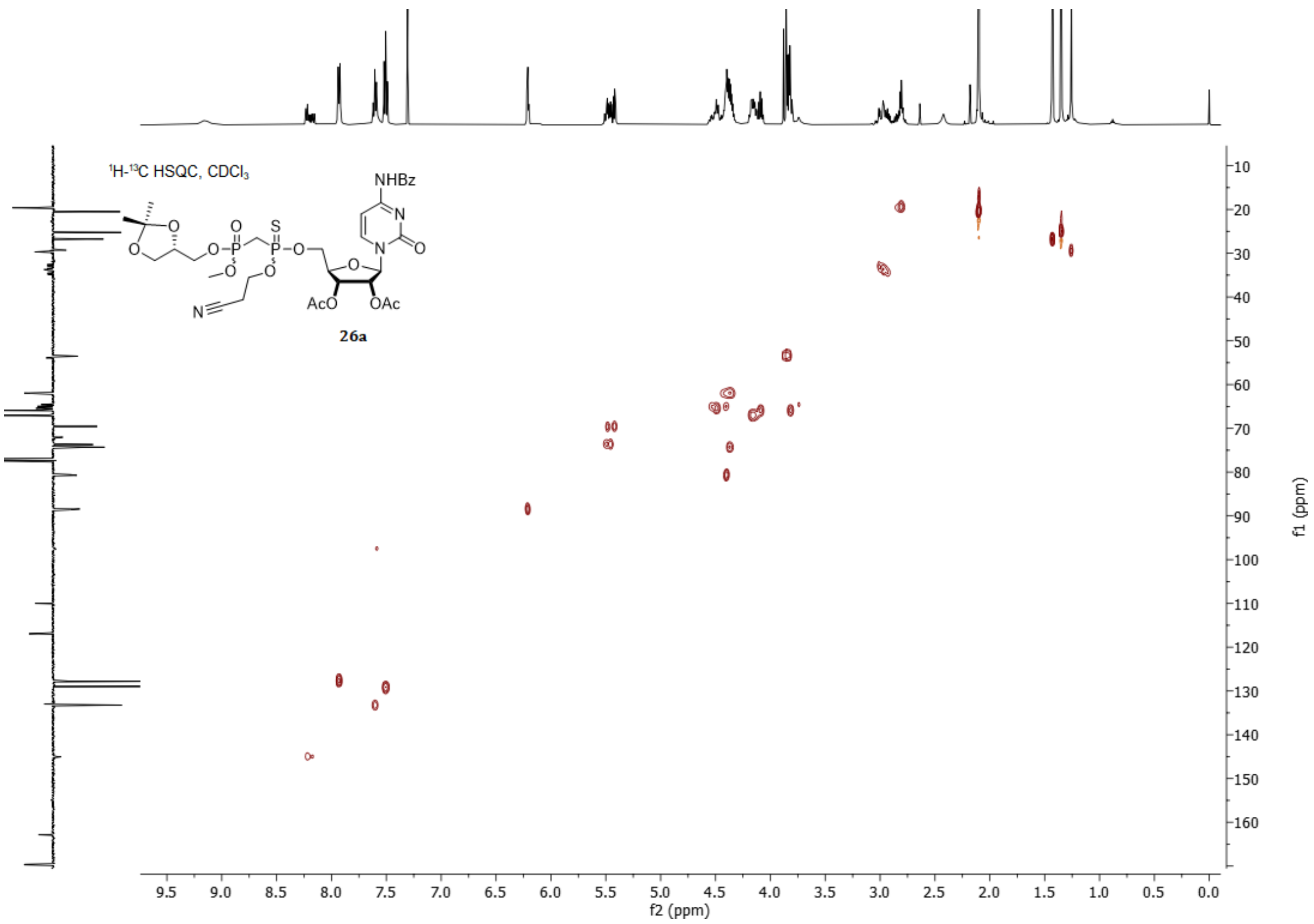

¹H-¹³C HSQC, CDCl₃
NHBz
AcO
OAc
26a
f2 (ppm)
f1 (ppm)

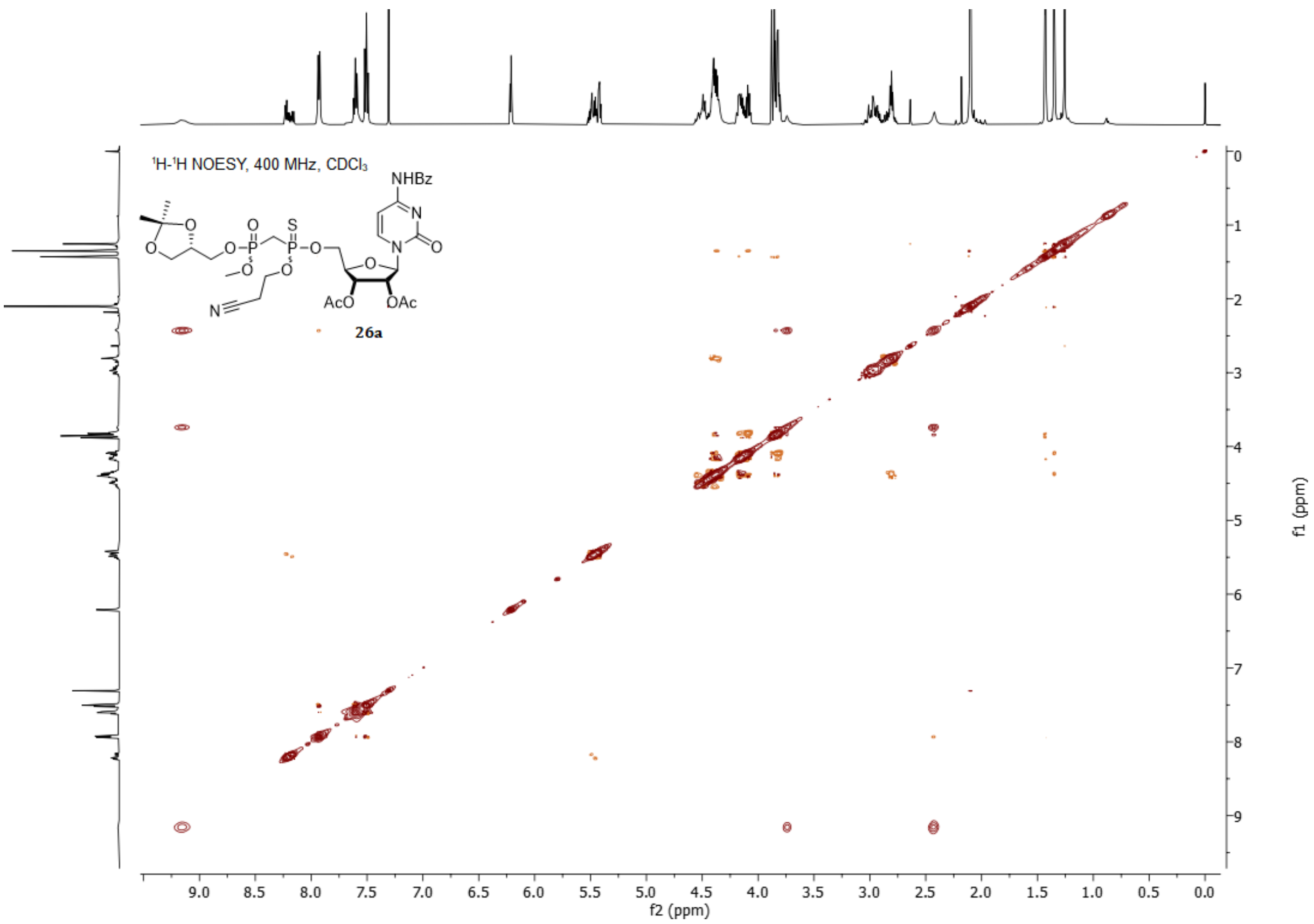

¹H-¹H NOESY, 400 MHz, CDCl₃
NHBz
N
O
S
AcO
OAc
26a
f2 (ppm)
f1 (ppm)

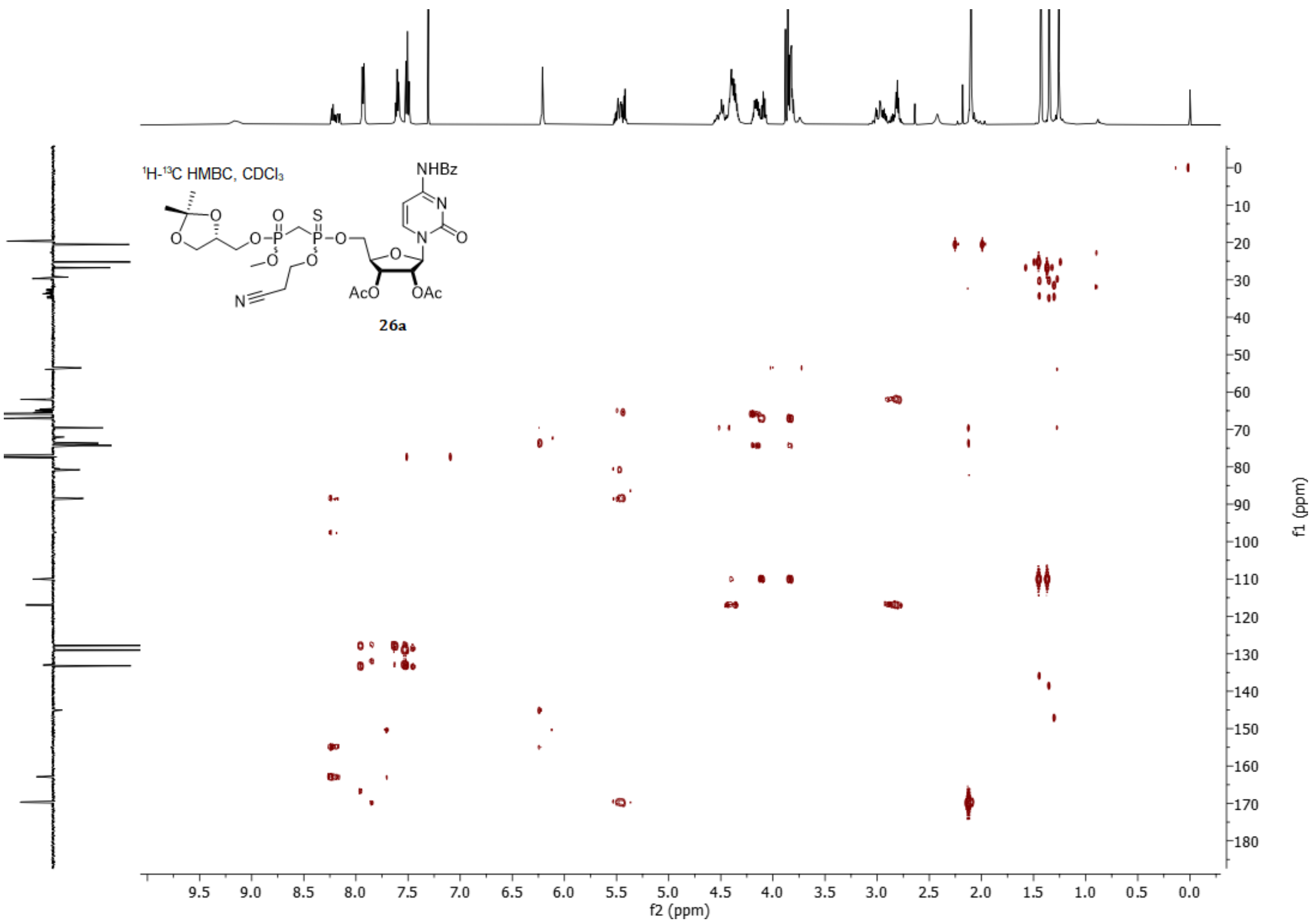

¹H-¹³C HMBC, CDCl₃
NHBz
N
O
S
AcO
OAc
26a
f2 (ppm)
f1 (ppm)

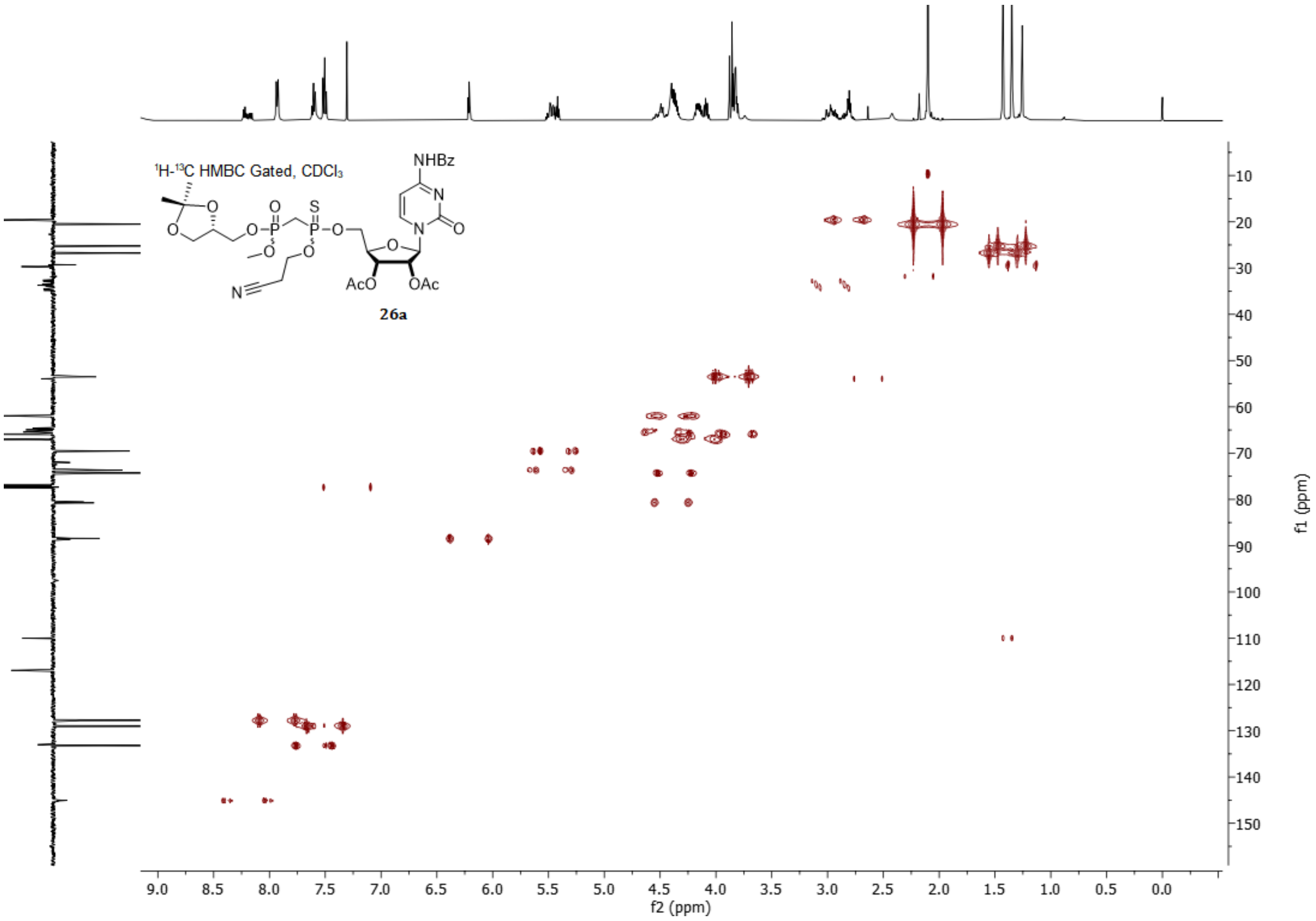

***sn*-1 me-CDP-αS$_2$-Gro tetraester 26b**

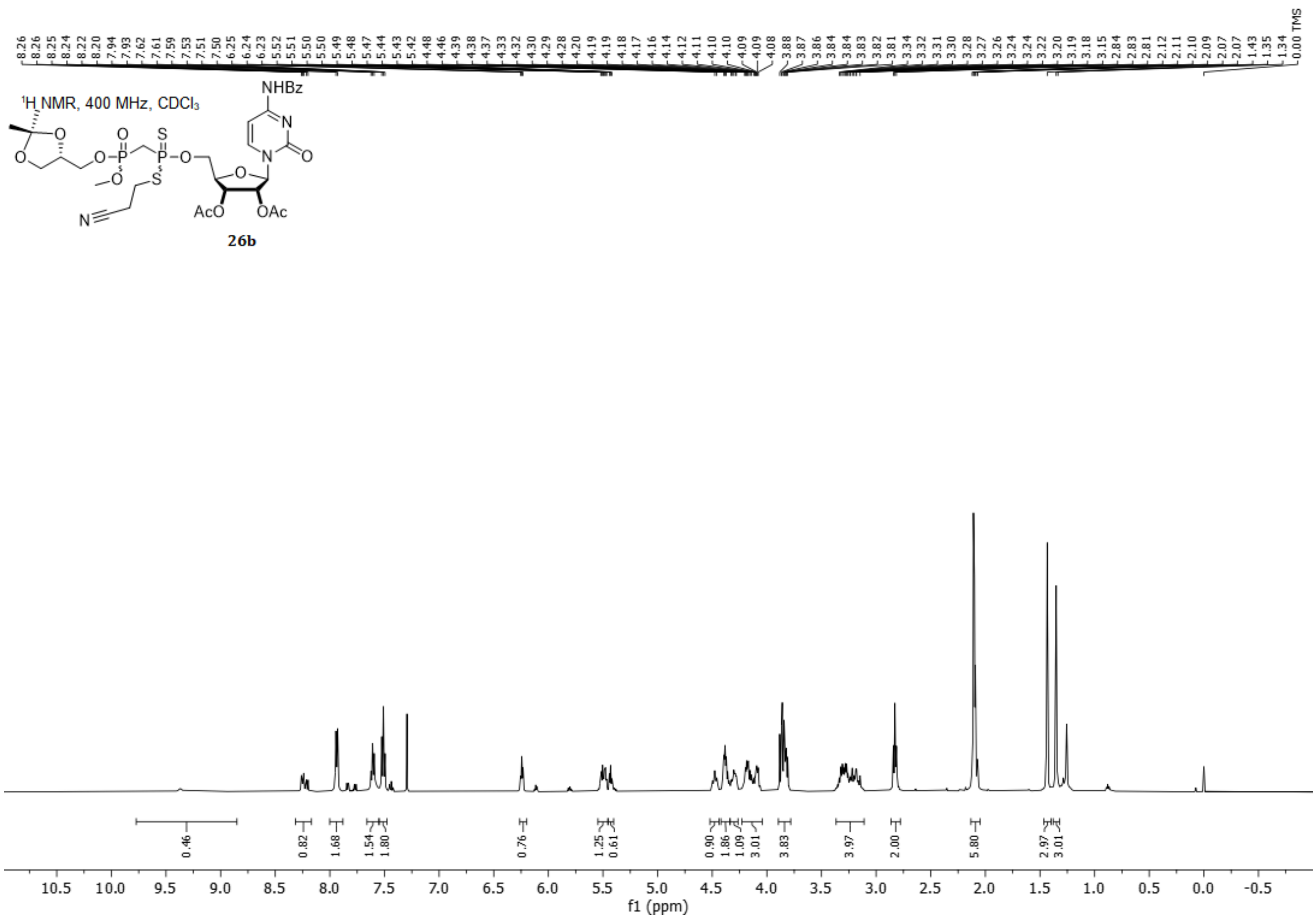

^{13}C NMR, 101 MHz, $CDCl_3$

26b

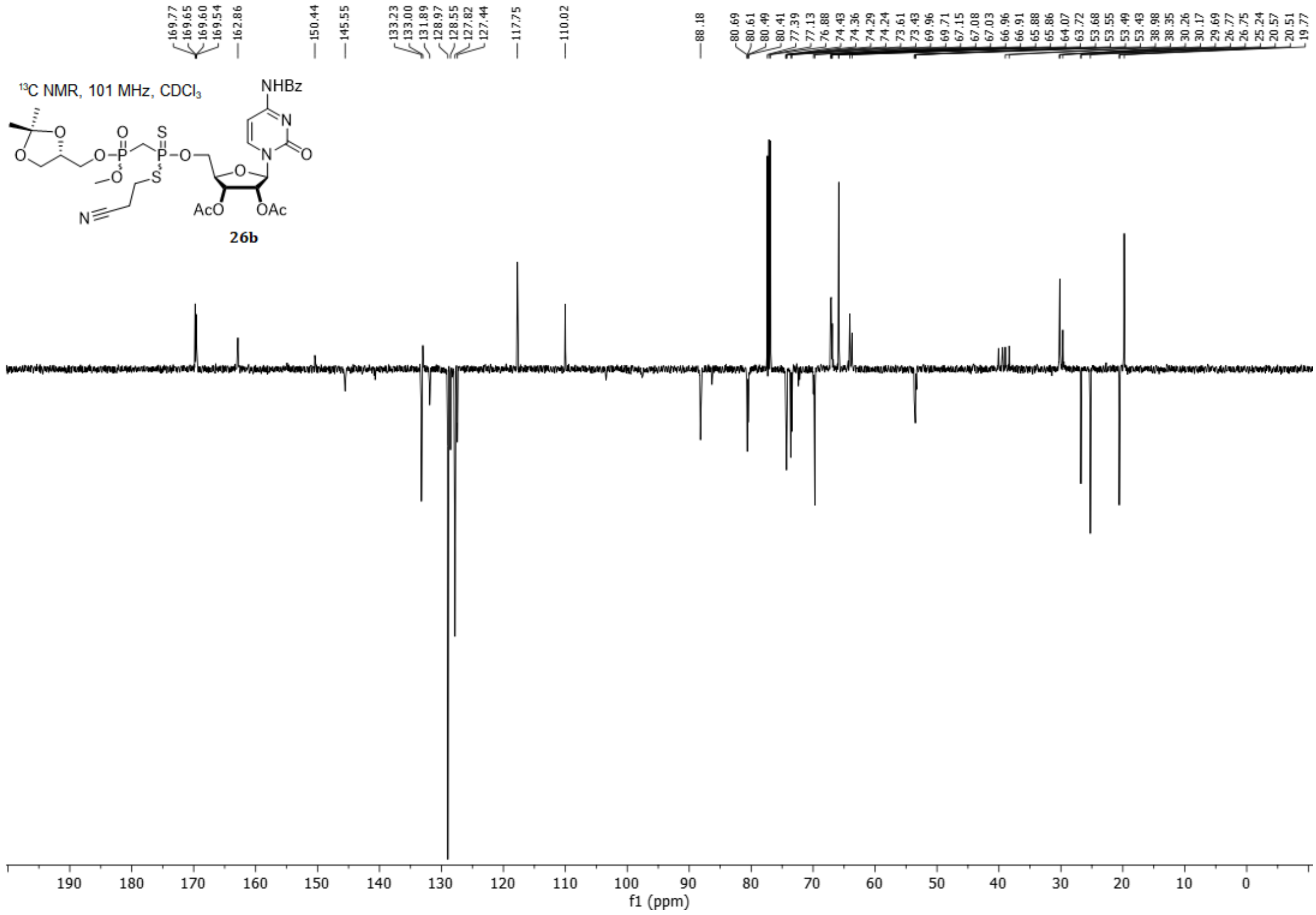

$^{31}P\{^1H\}$ NMR, 162 MHz, $CDCl_3$

26b

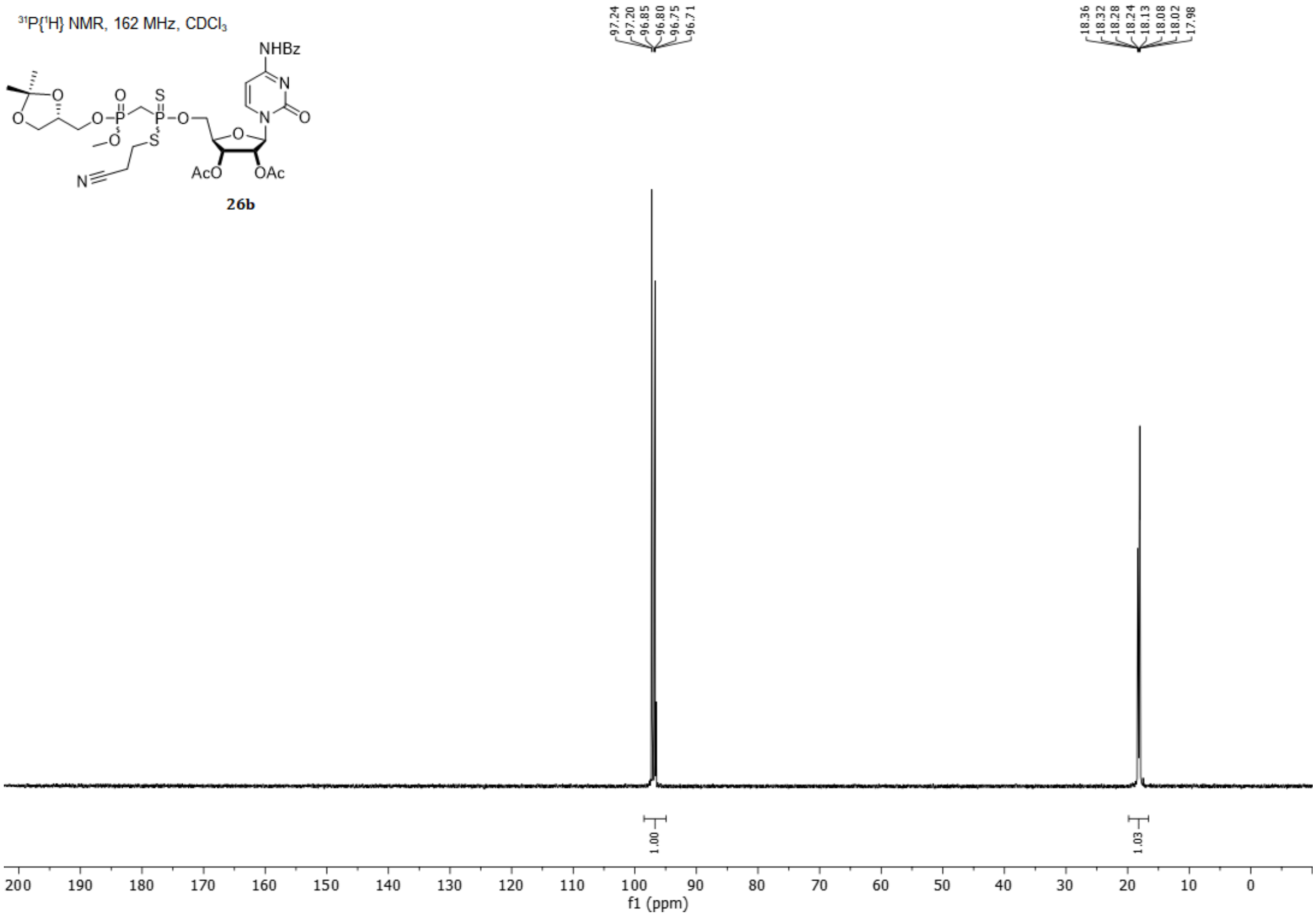

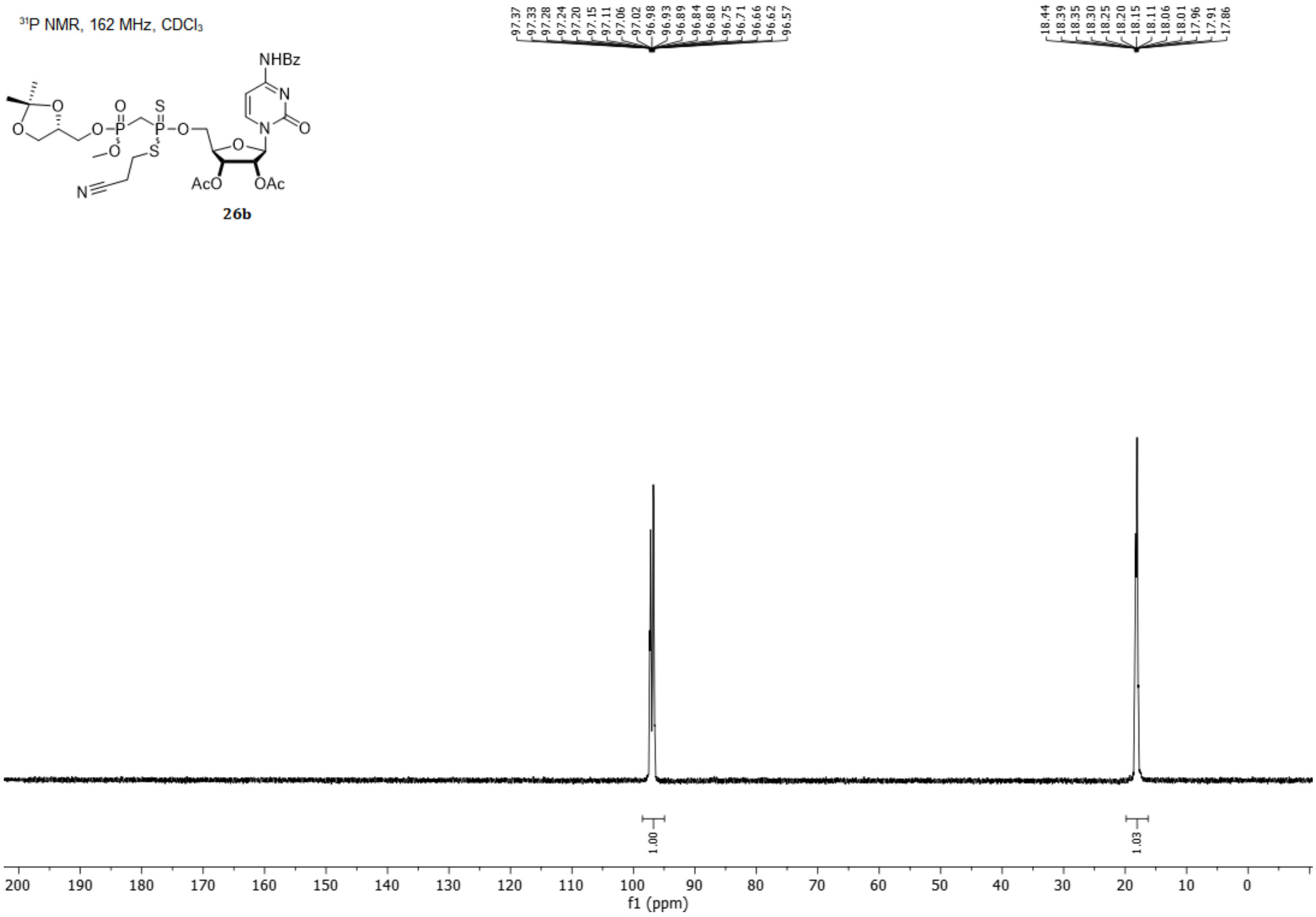

³¹P NMR, 162 MHz, CDCl₃
NHBz
O
N
O
O
P
S
P
O
O
N
O
O
S
N
AcO
OAc
26b
97.37
97.33
97.28
97.24
97.20
97.15
97.11
97.06
97.02
96.98
96.93
96.89
96.84
96.80
96.75
96.71
96.66
96.62
96.57
18.44
18.39
18.35
18.30
18.25
18.20
18.15
18.11
18.06
18.01
17.96
17.91
17.86
1.00
1.03
200 190 180 170 160 150 140 130 120 110 100 90 80 70 60 50 40 30 20 10 0
f1 (ppm)

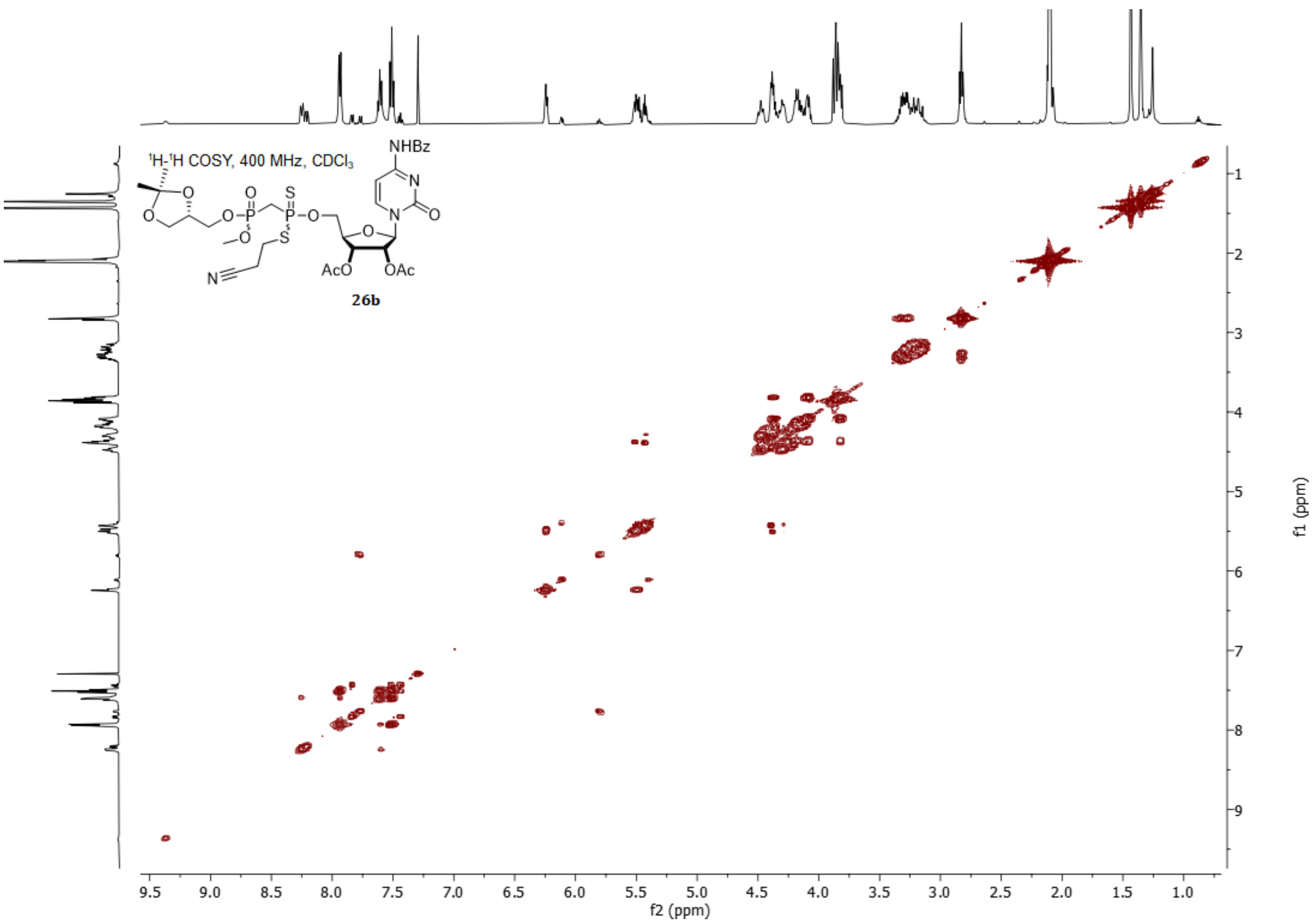

¹H-¹H COSY, 400 MHz, CDCl₃
NHBz
26b
AcO
OAc
9.5 9.0 8.5 8.0 7.5 7.0 6.5 6.0 5.5 5.0 4.5 4.0 3.5 3.0 2.5 2.0 1.5 1.0
f2 (ppm)
1 2 3 4 5 6 7 8 9
f1 (ppm)

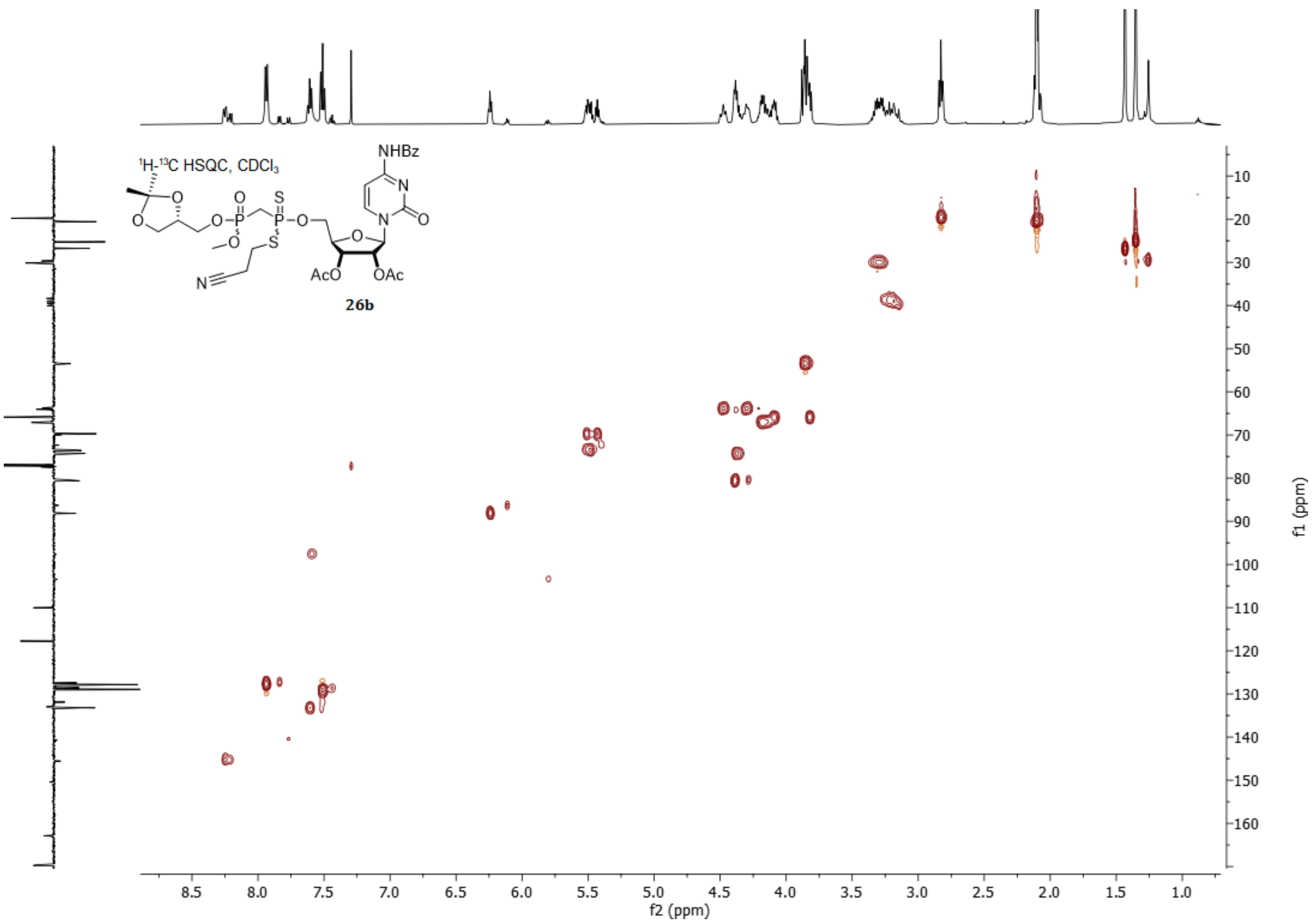

¹H-¹³C HSQC, CDCl₃
NHBz
26b
f2 (ppm)
f1 (ppm)

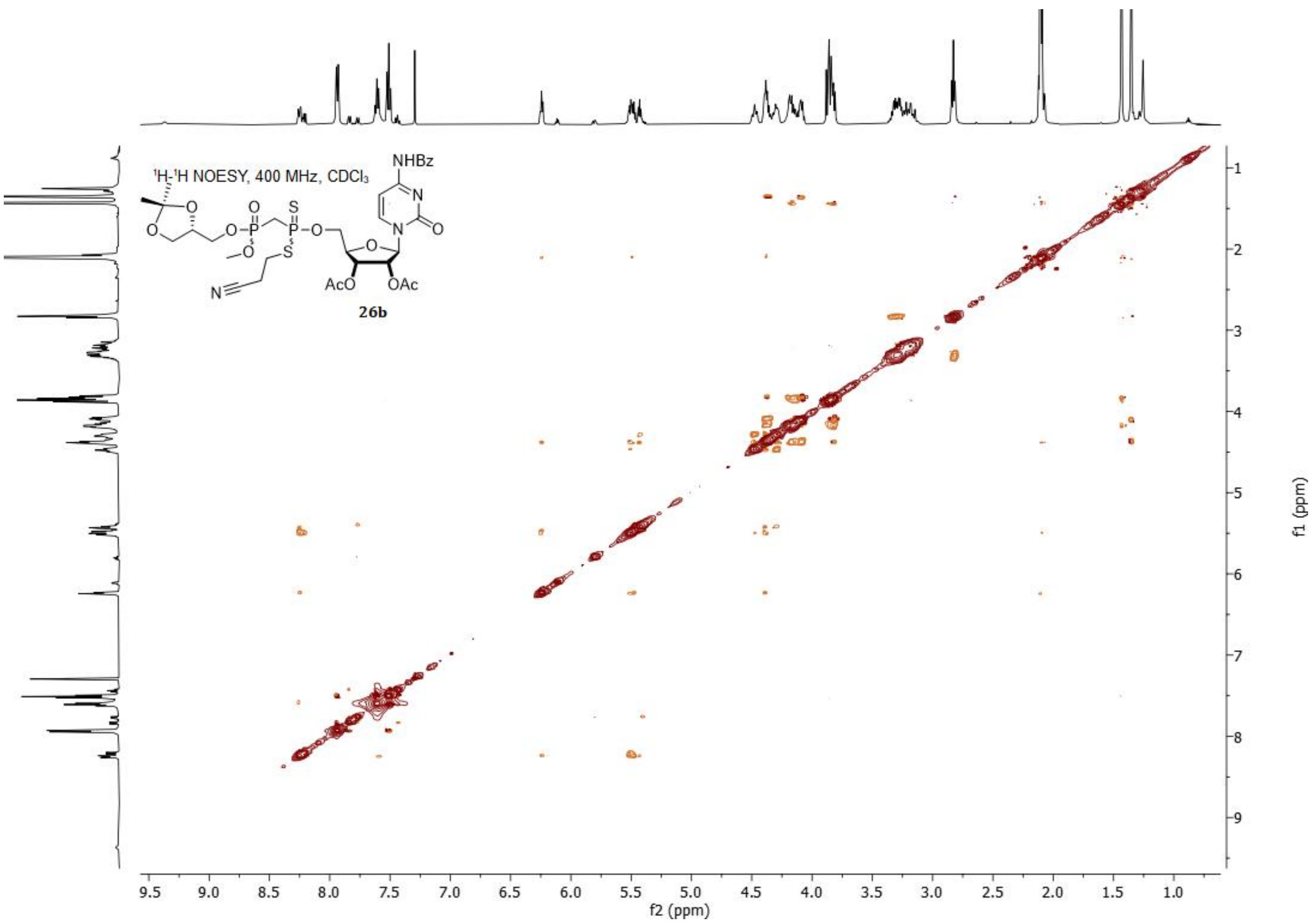

¹H-¹H NOESY, 400 MHz, CDCl₃
NHBz
26b
f2 (ppm)
f1 (ppm)

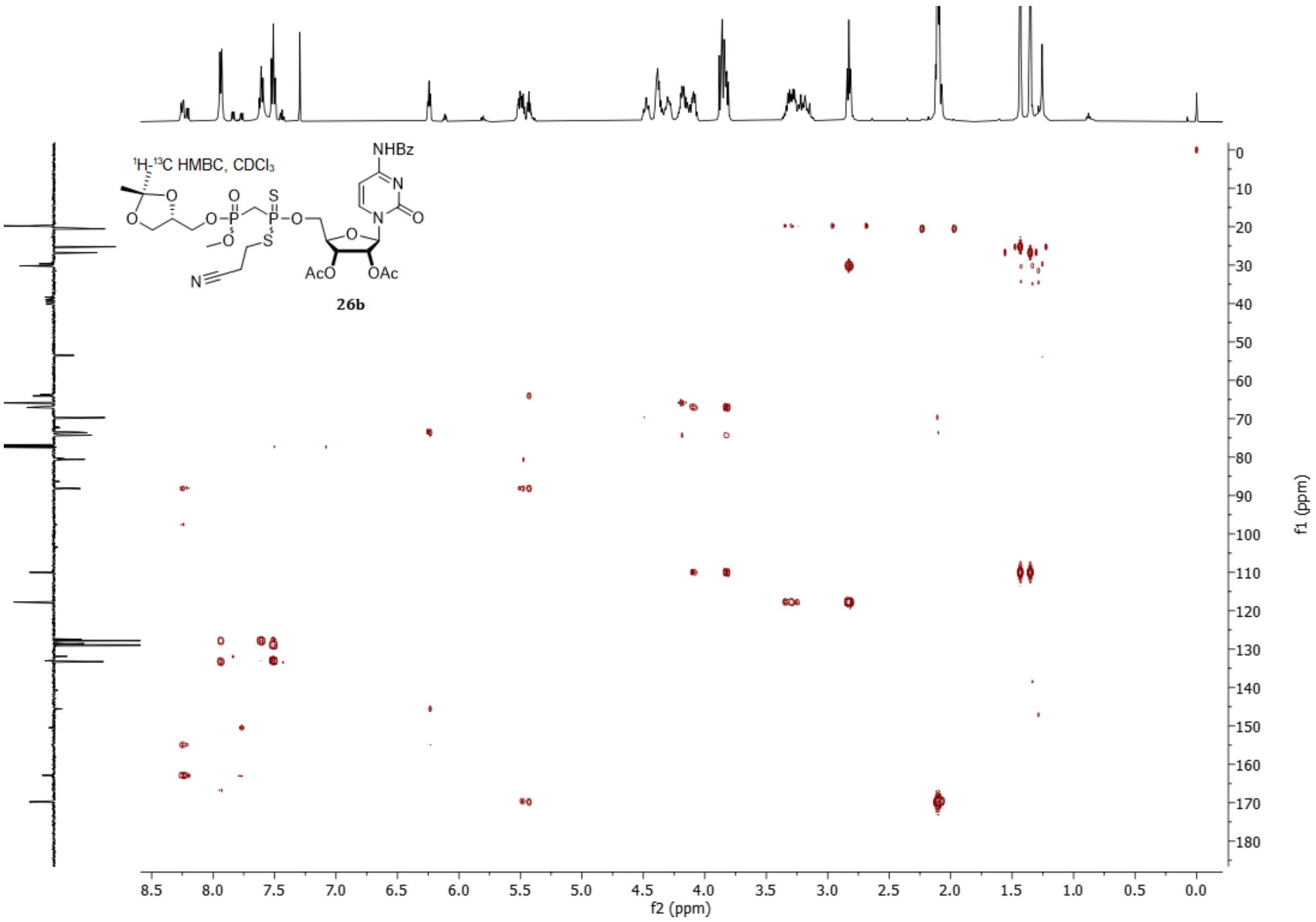

me-CDP-αS-ribitol tetraester 27a

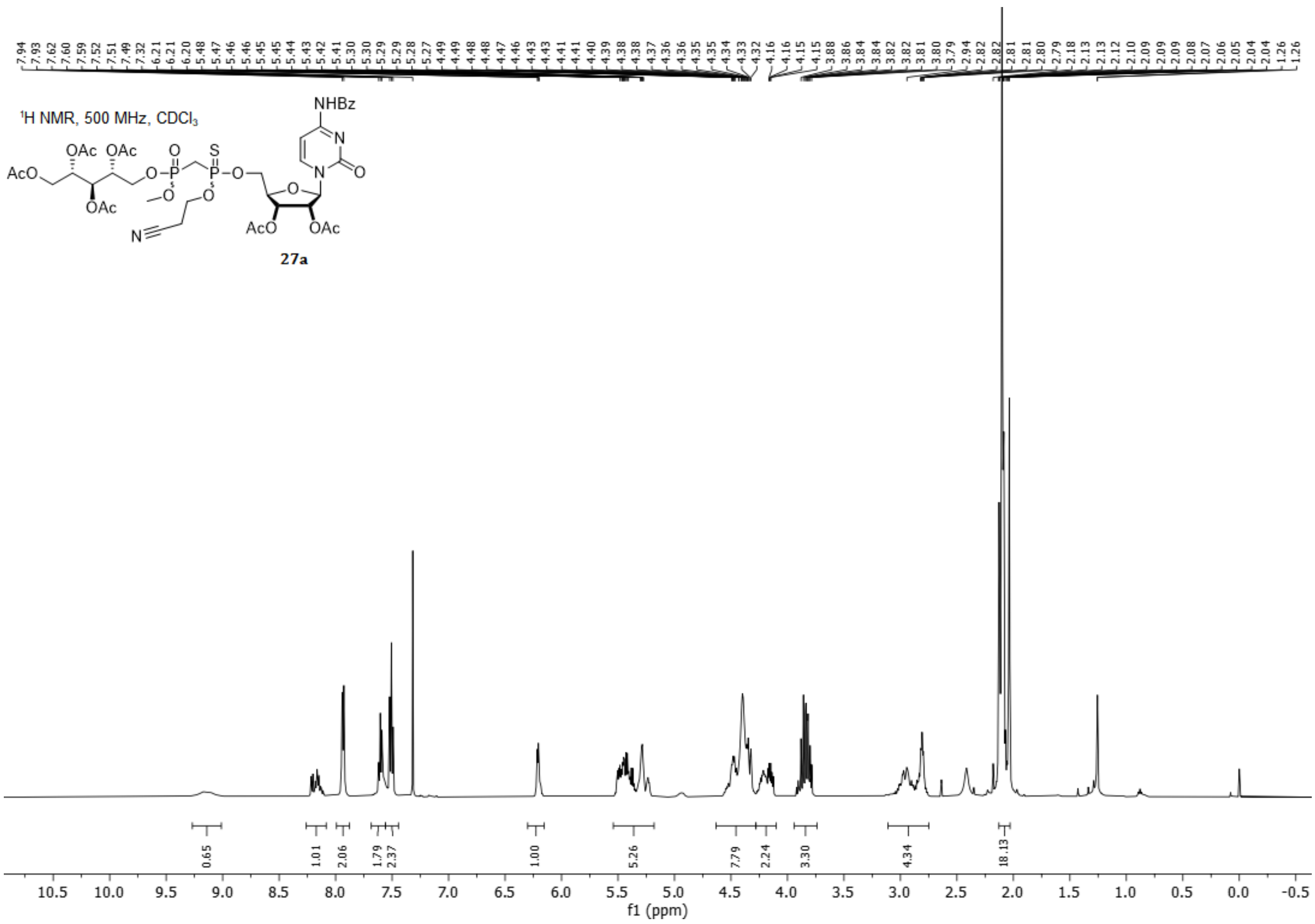

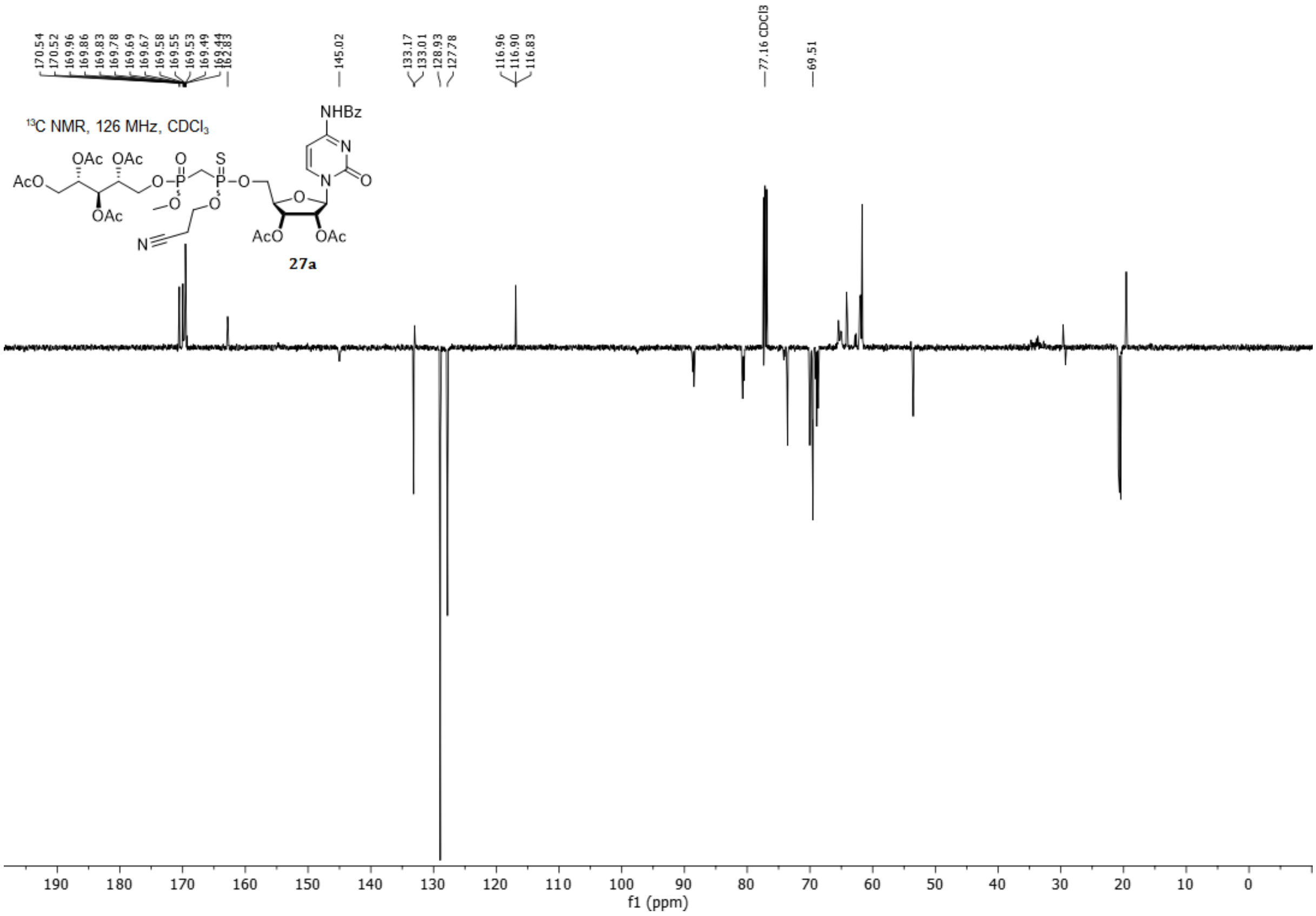

170.54
170.52
169.96
169.86
169.83
169.78
169.69
169.67
169.58
169.55
169.53
169.49
145.02
133.17
133.01
128.93
127.78
116.96
116.90
116.83
77.16 CDCl3
69.51
13C NMR, 126 MHz, CDCl3
NHBz
AcO
OAc
27a
f1 (ppm)

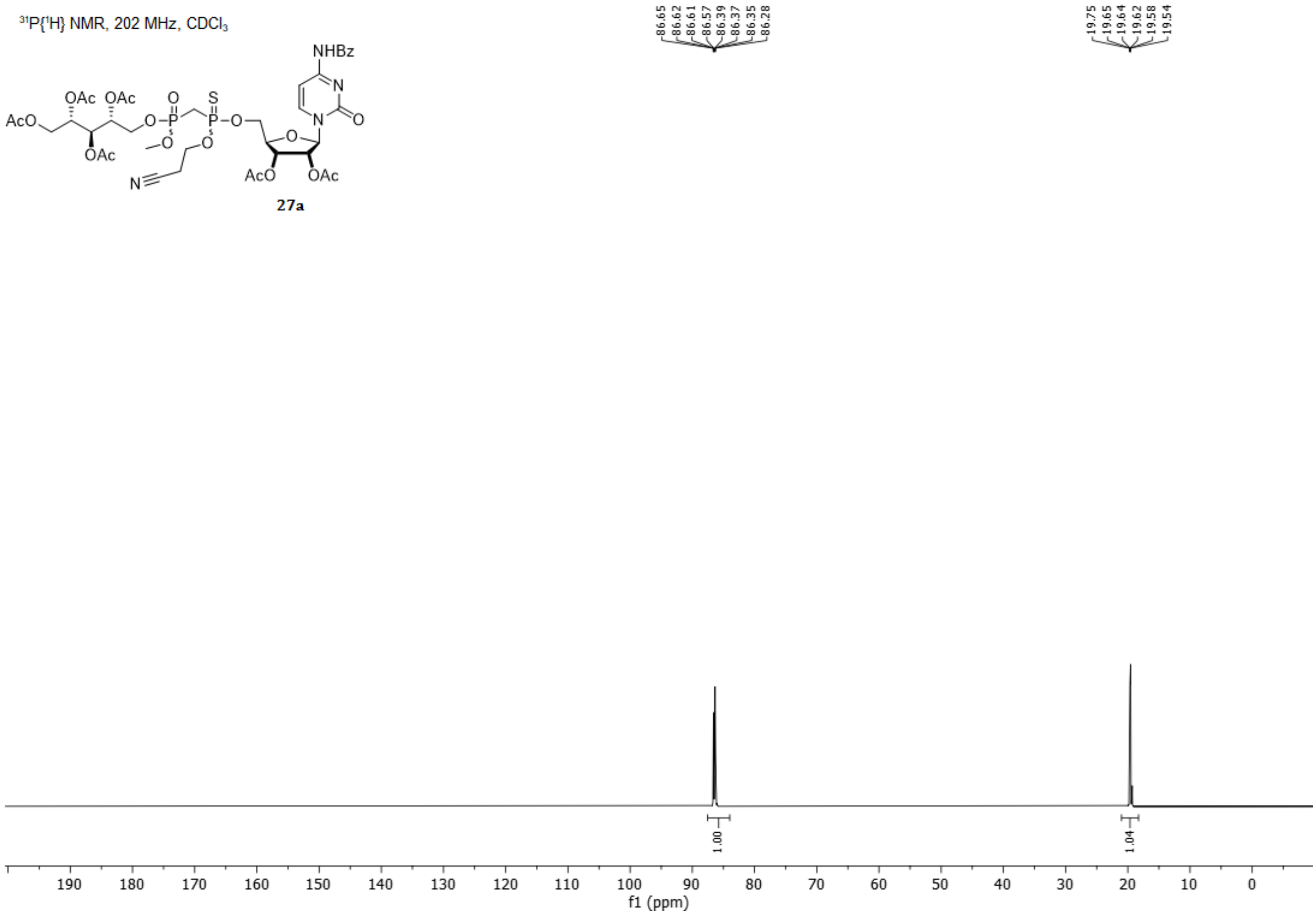

31P{1H} NMR, 202 MHz, CDCl3
86.65
86.62
86.61
86.57
86.39
86.37
86.35
86.28
19.75
19.65
19.64
19.62
19.58
19.54
NHBz
AcO
OAc
27a
1.00
1.04
f1 (ppm)

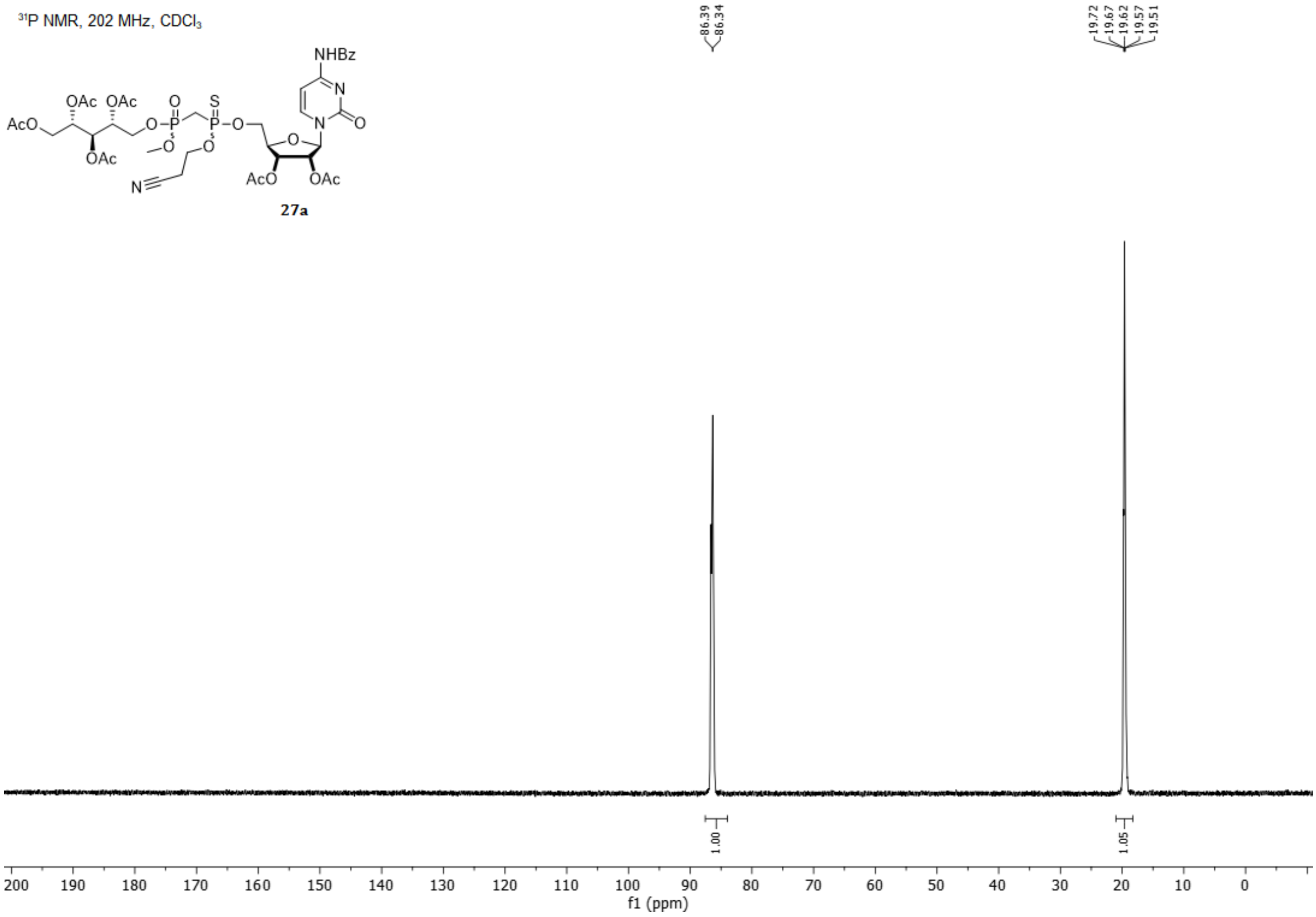

31P NMR, 202 MHz, CDCl3
27a
86.39
86.34
19.72
19.67
19.62
19.57
19.51
1.00
1.05
f1 (ppm)

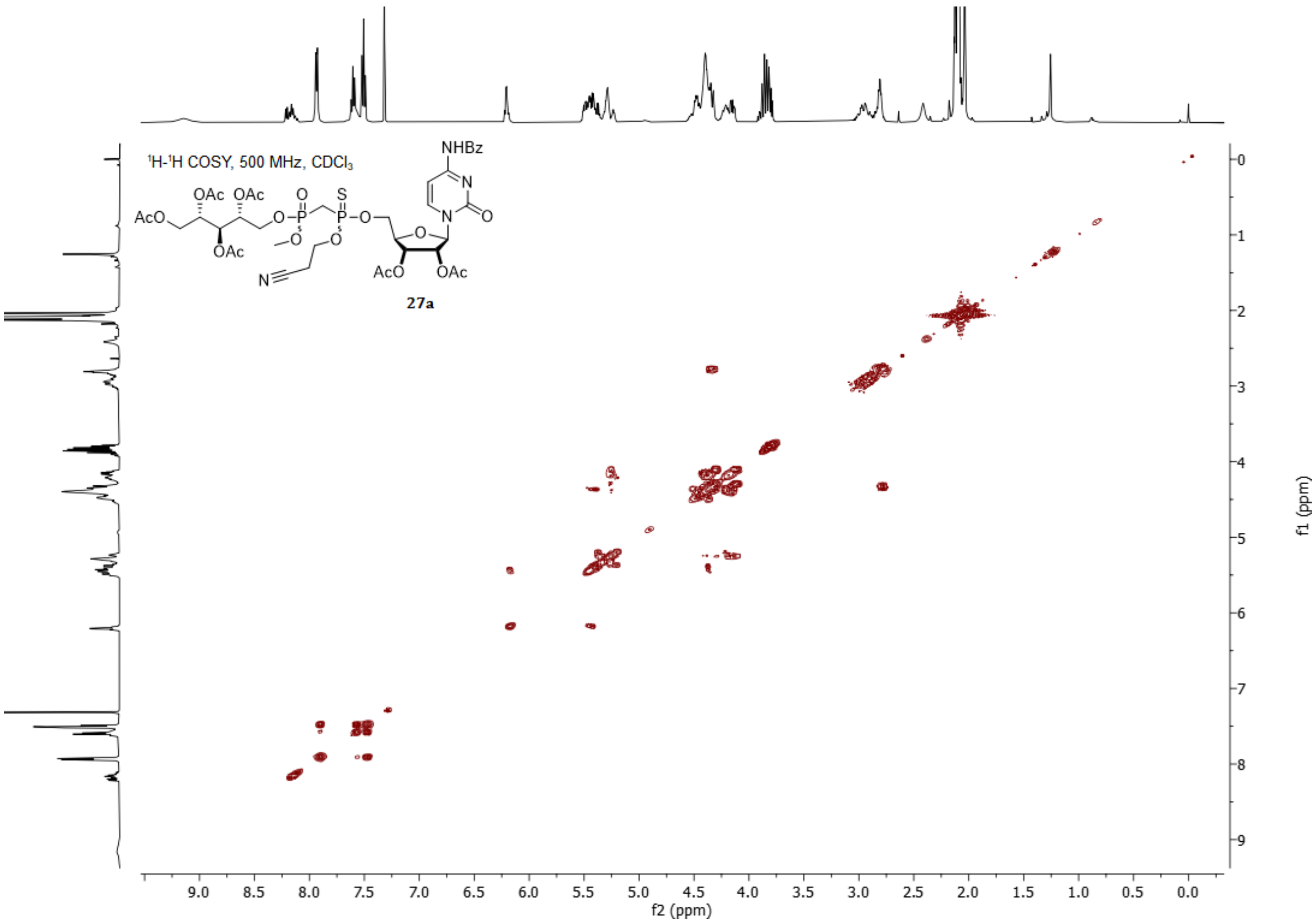

1H-1H COSY, 500 MHz, CDCl3
27a
f2 (ppm)
f1 (ppm)

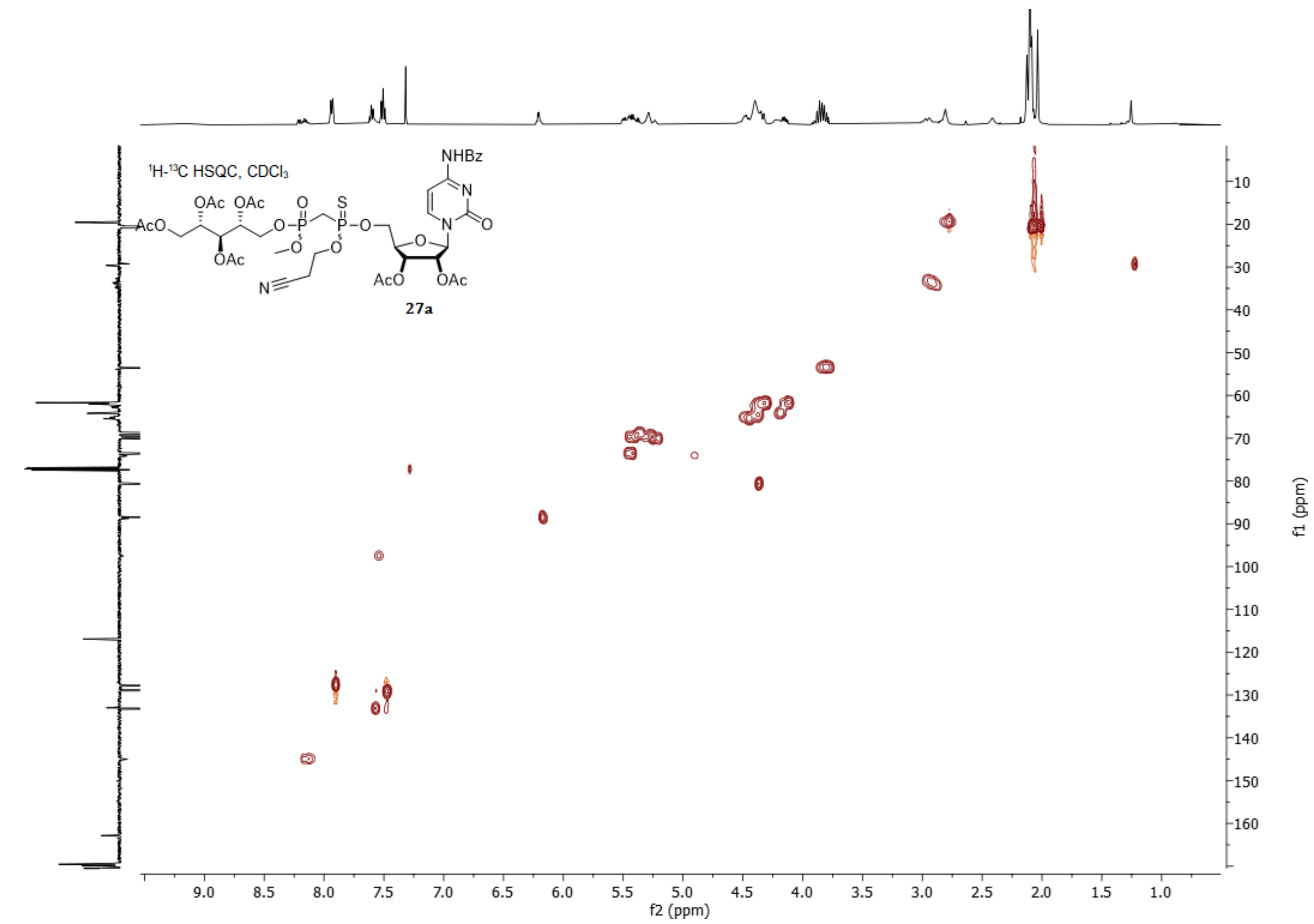
¹H-¹³C HSQC, CDCl₃
27a
f2 (ppm)
f1 (ppm)

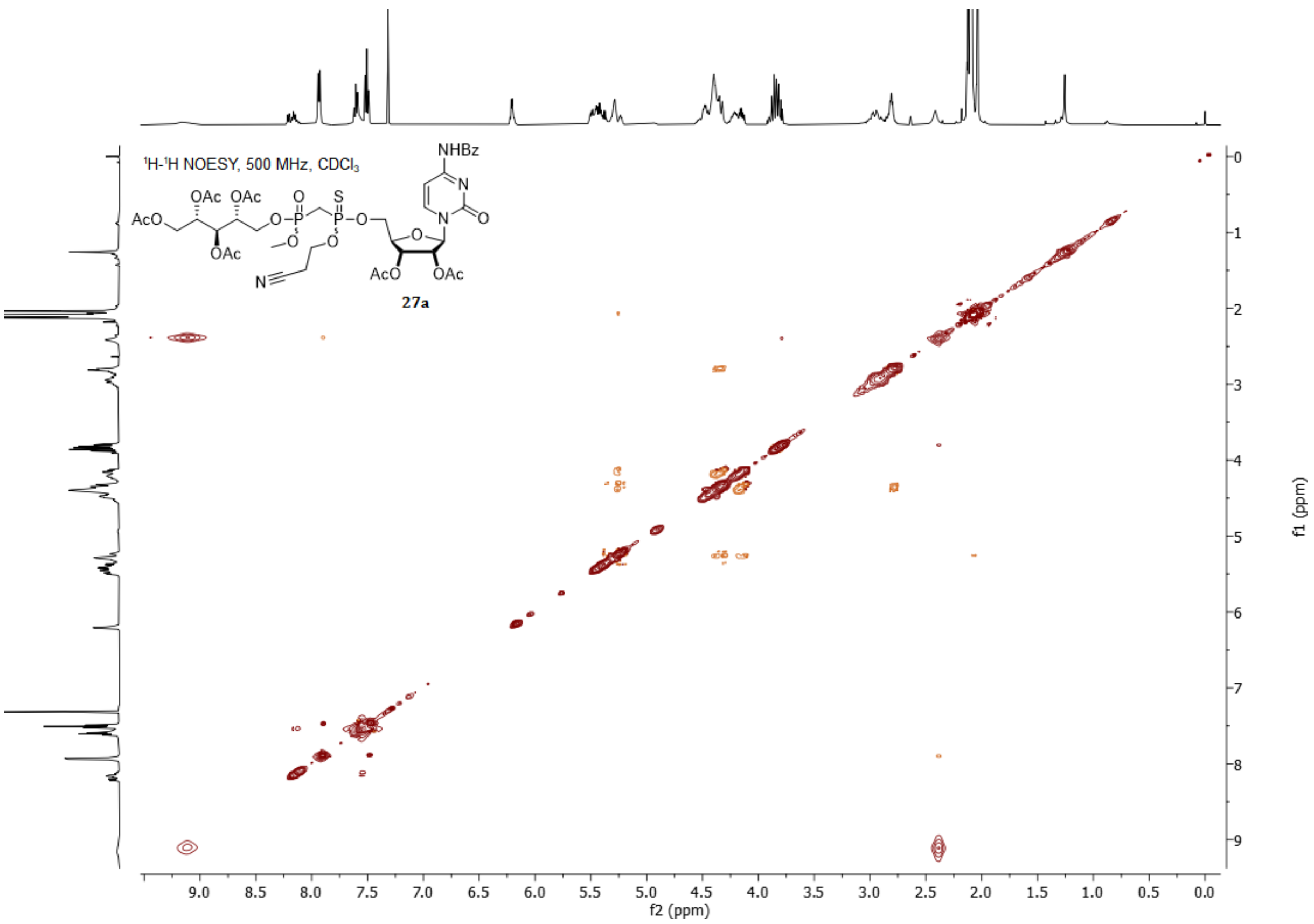
¹H-¹H NOESY, 500 MHz, CDCl₃
27a
f2 (ppm)
f1 (ppm)

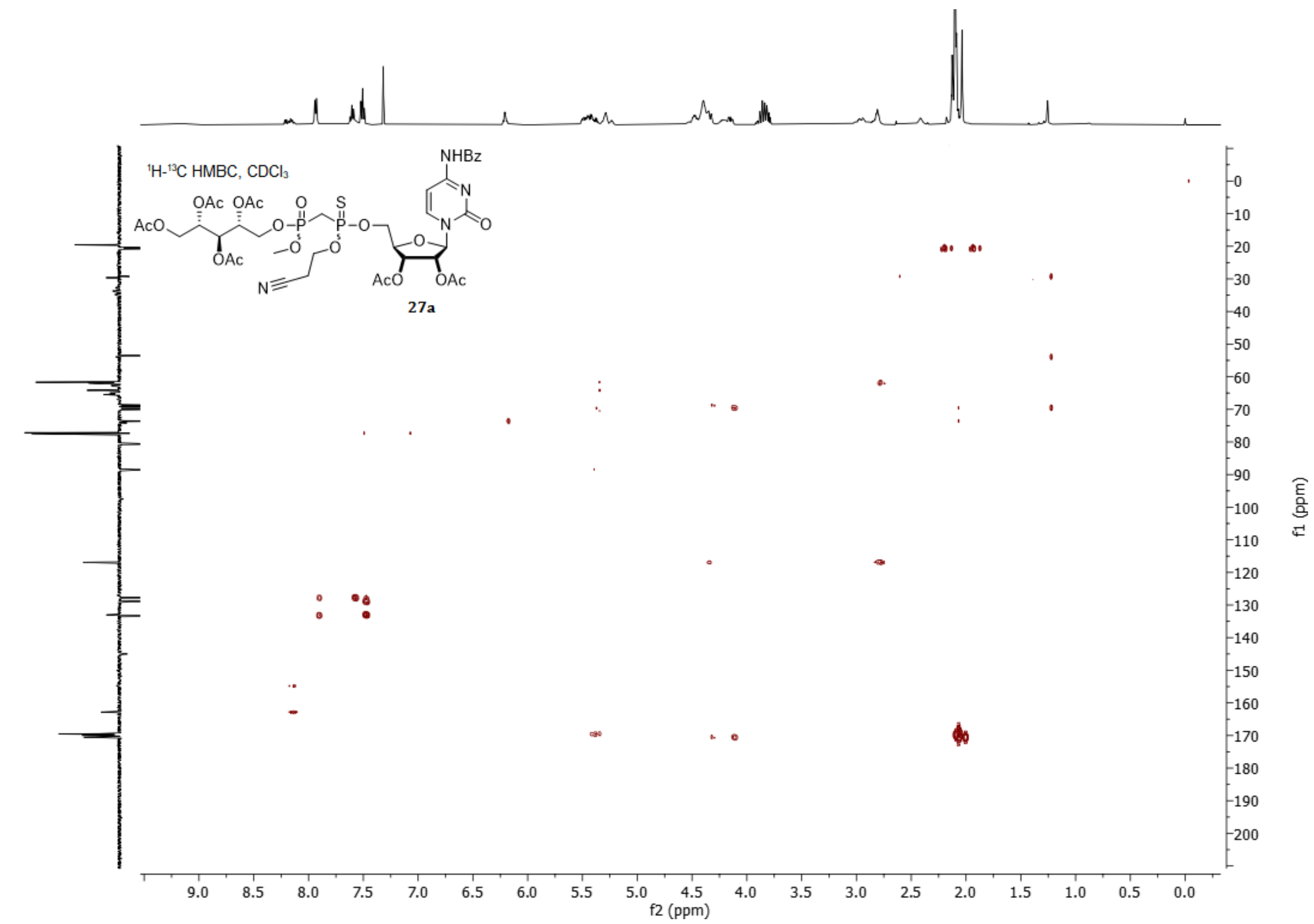

me-CDP-αS$_2$-ribitol tetraester 27b

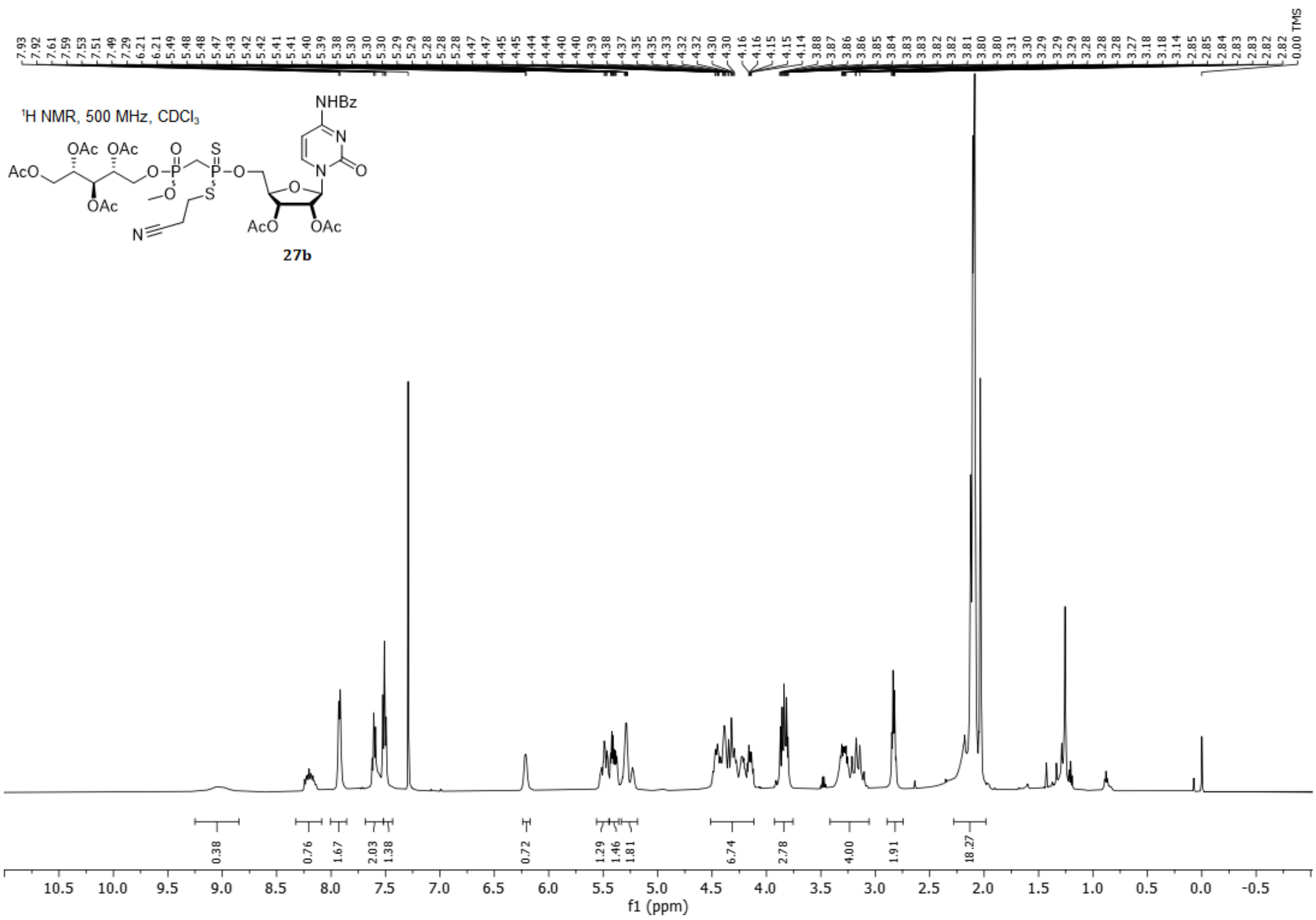

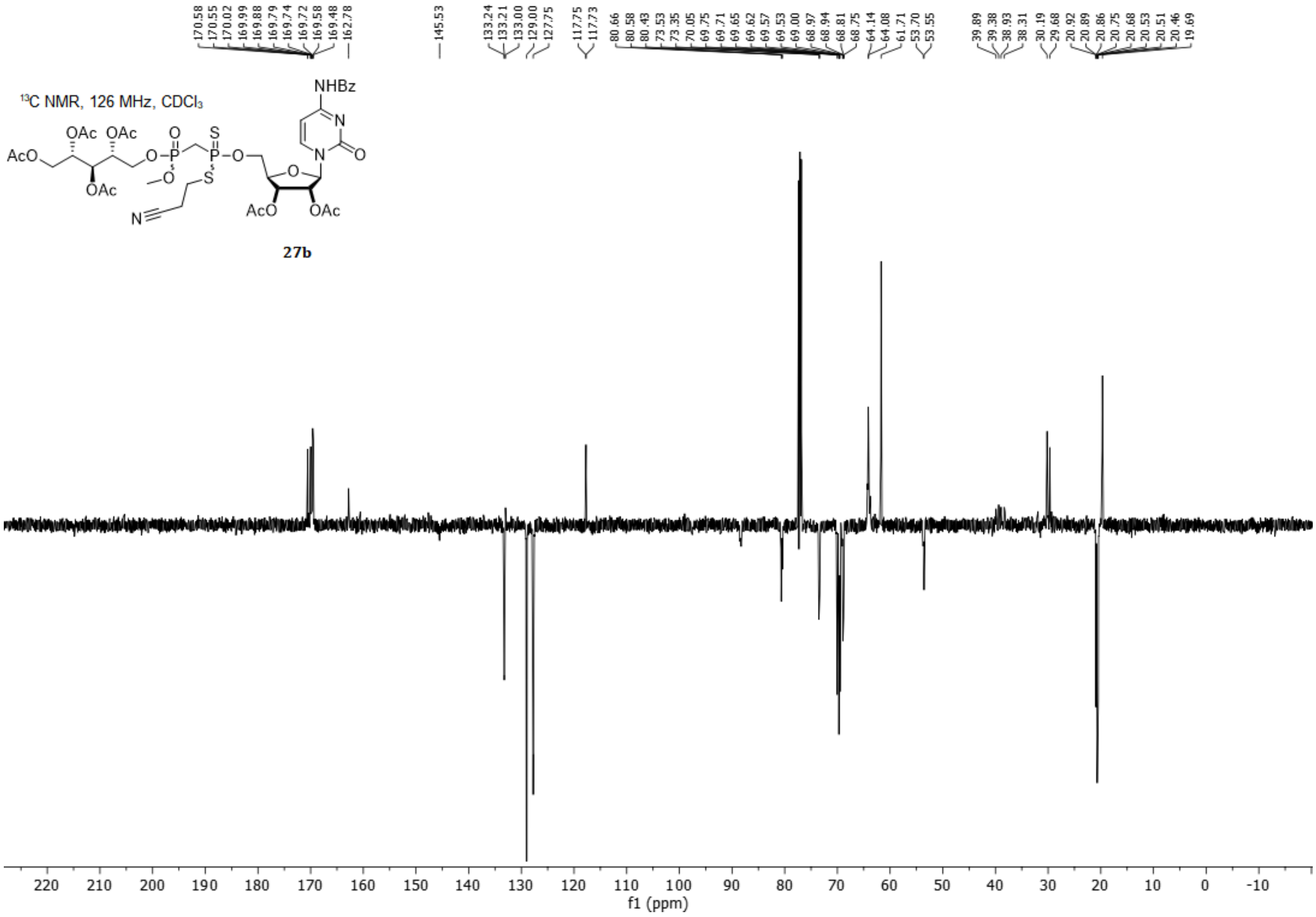

^{13}C NMR, 126 MHz, $CDCl_3$

27b

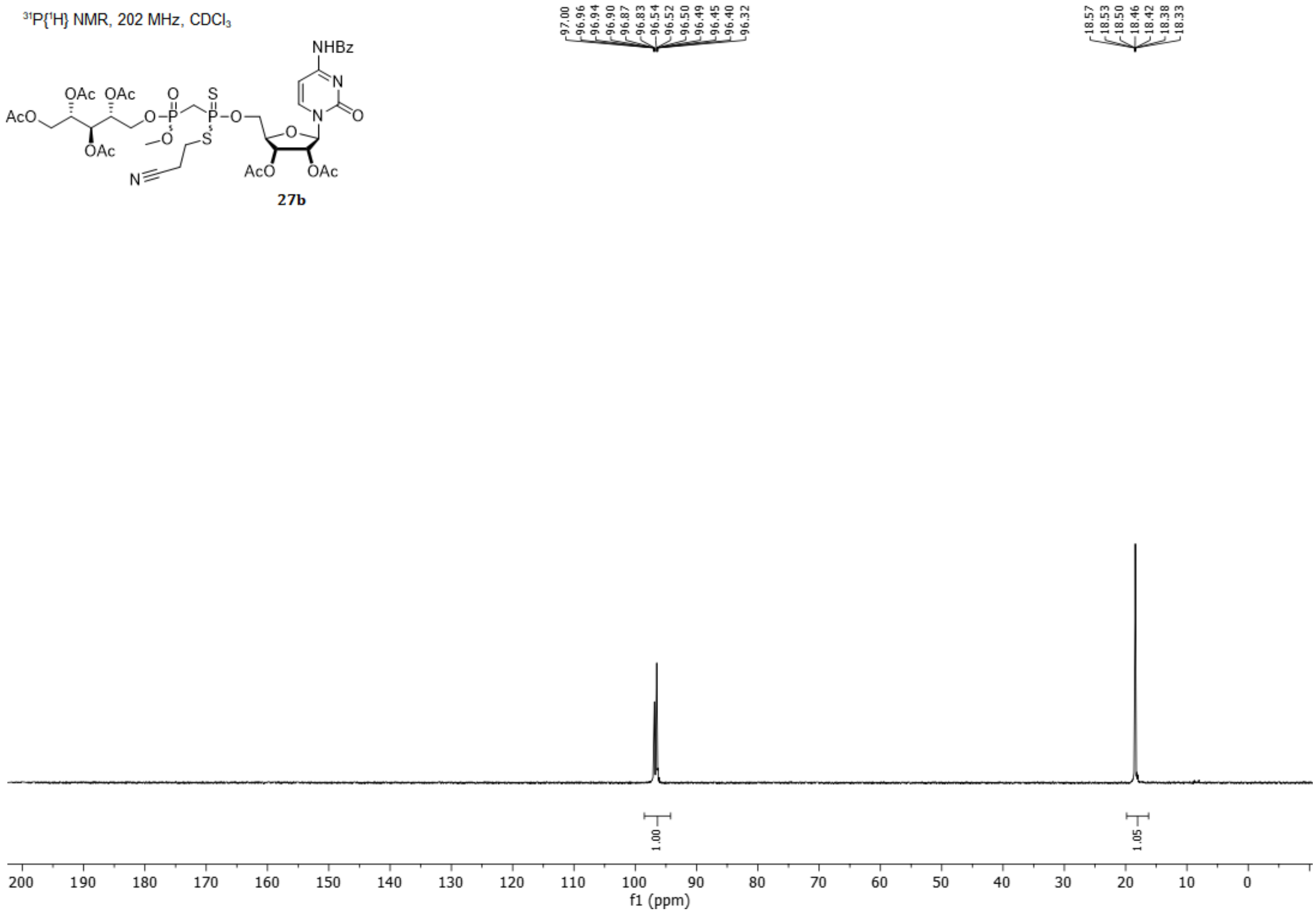

$^{31}P\{^1H\}$ NMR, 202 MHz, $CDCl_3$

27b

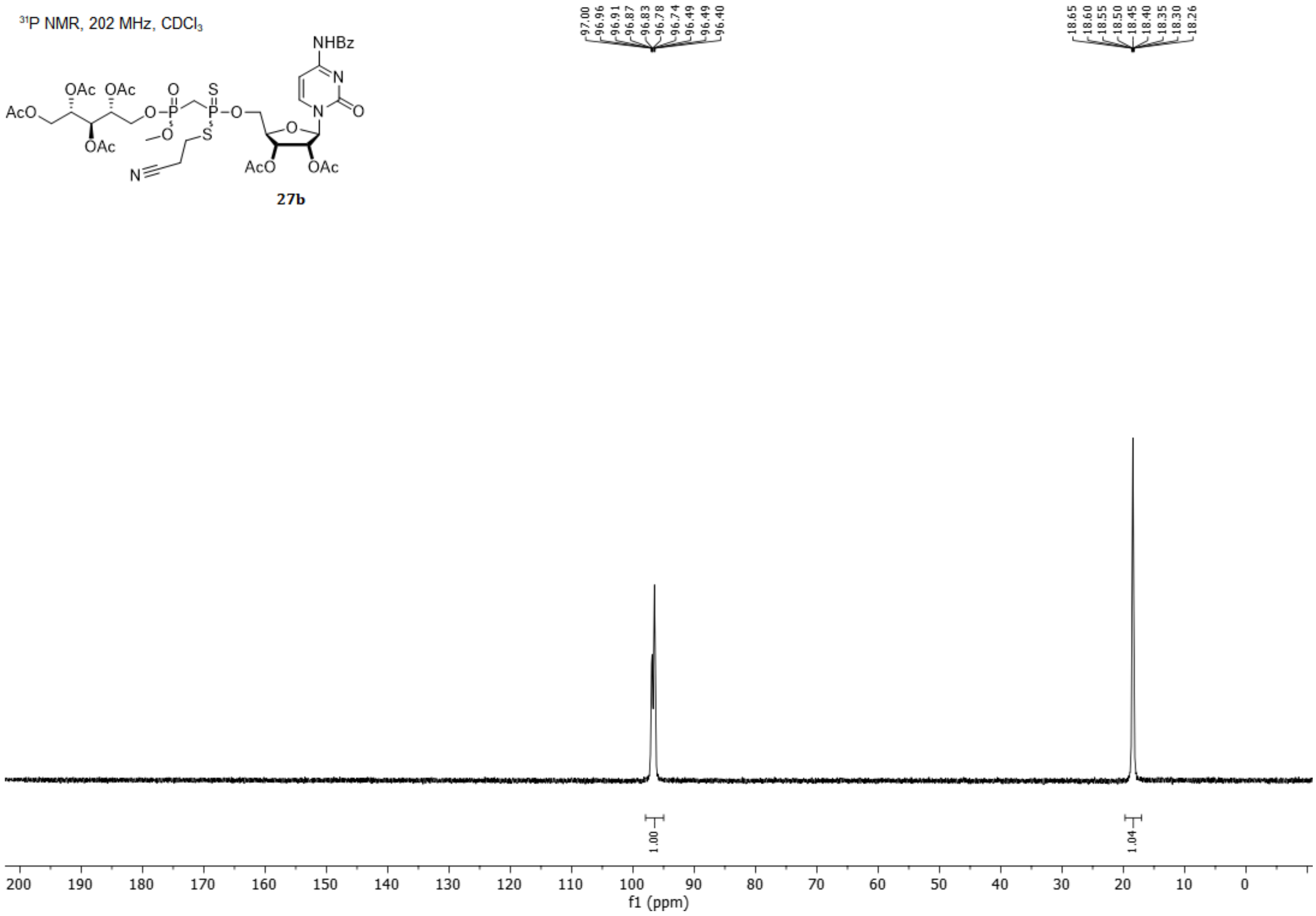
31P NMR, 202 MHz, CDCl3
NHBz
27b
97.00
96.96
96.91
96.87
96.83
96.78
96.74
96.49
96.49
96.40
18.65
18.60
18.55
18.50
18.45
18.40
18.35
18.30
18.26
1.00
1.04
f1 (ppm)

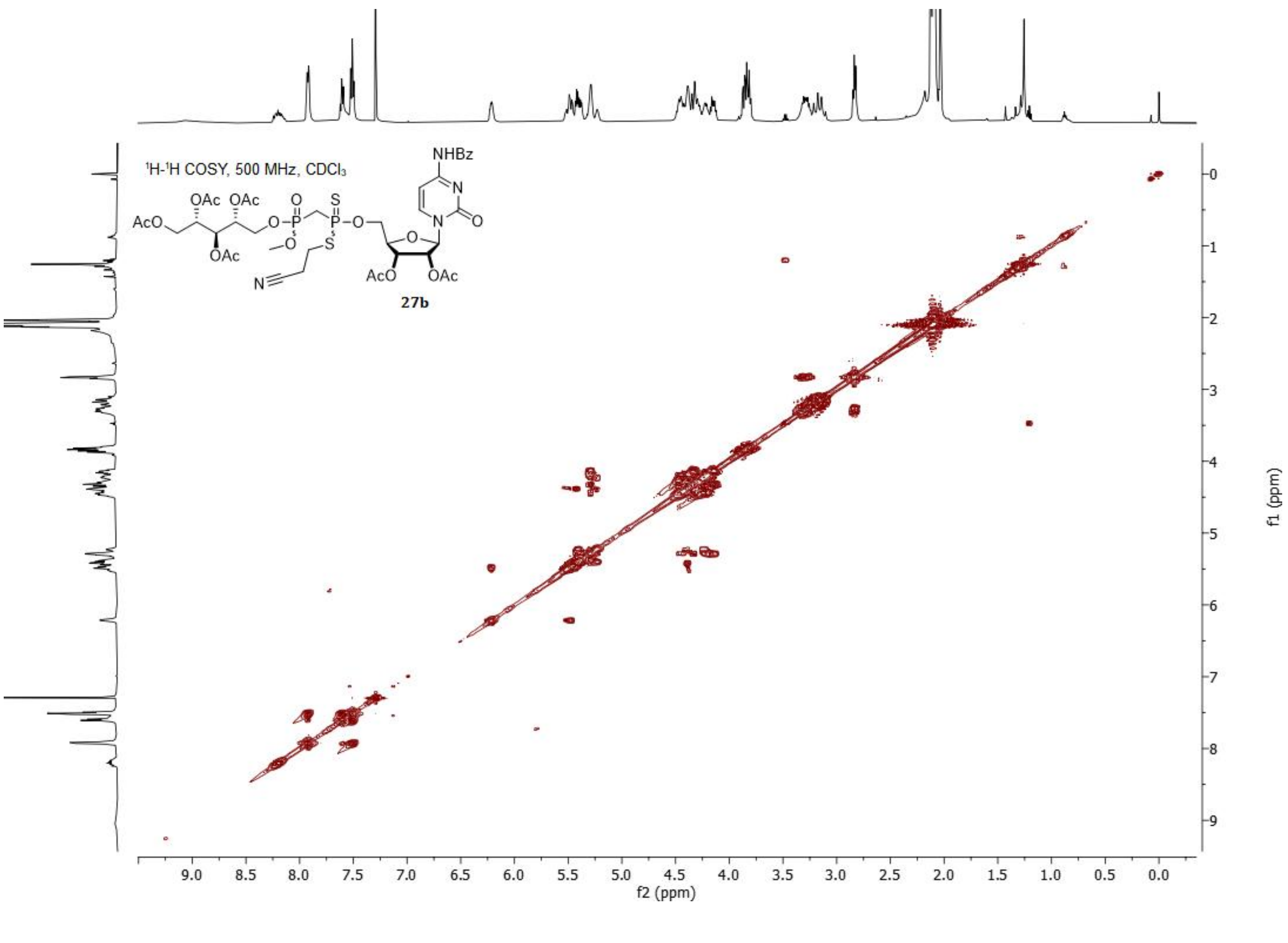
1H-1H COSY, 500 MHz, CDCl3
NHBz
27b
f2 (ppm)
f1 (ppm)

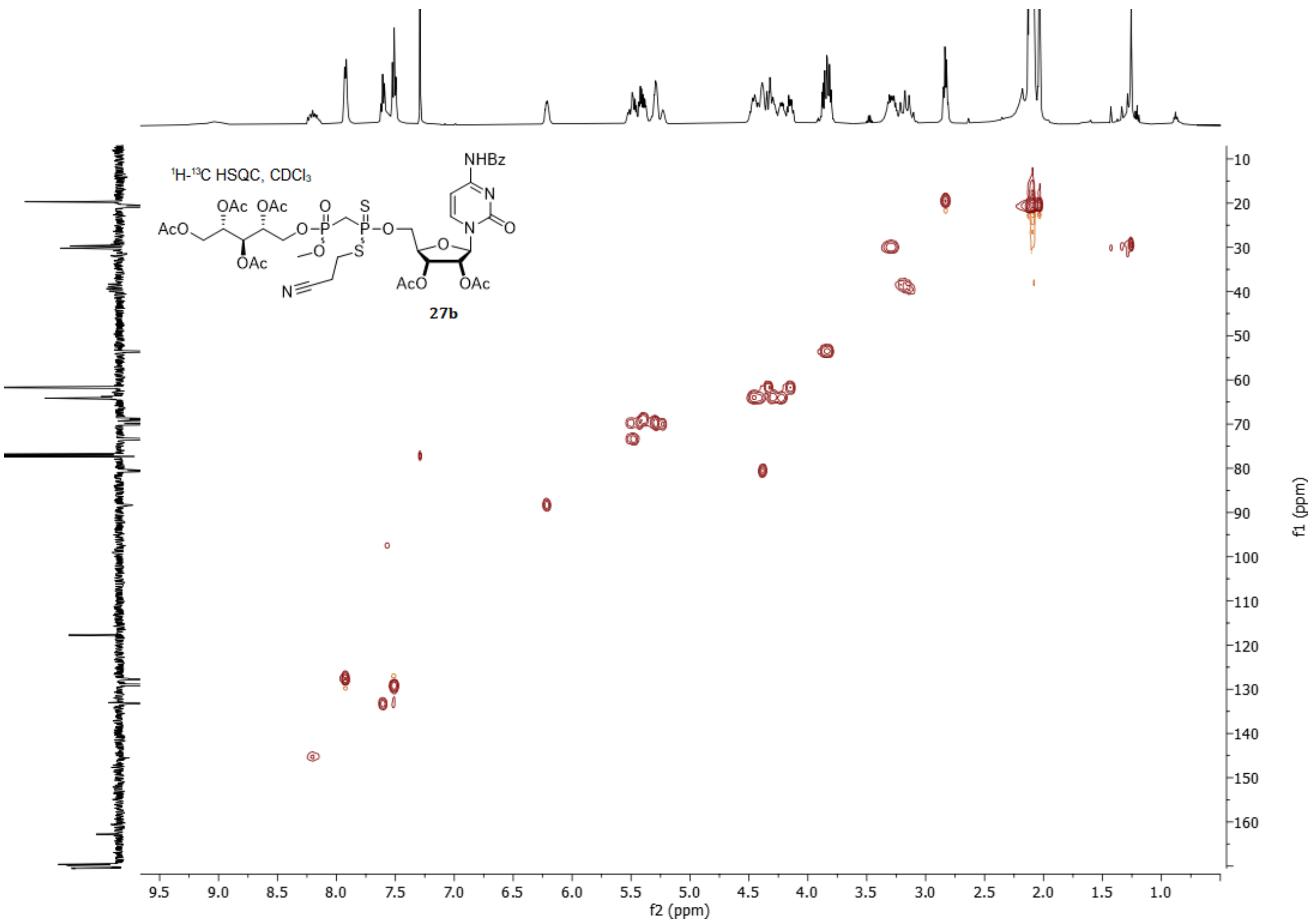
1H-13C HSQC, CDCl3
NHBz
OAc OAc
AcO
OAc
AcO
OAc
27b
f2 (ppm)
f1 (ppm)

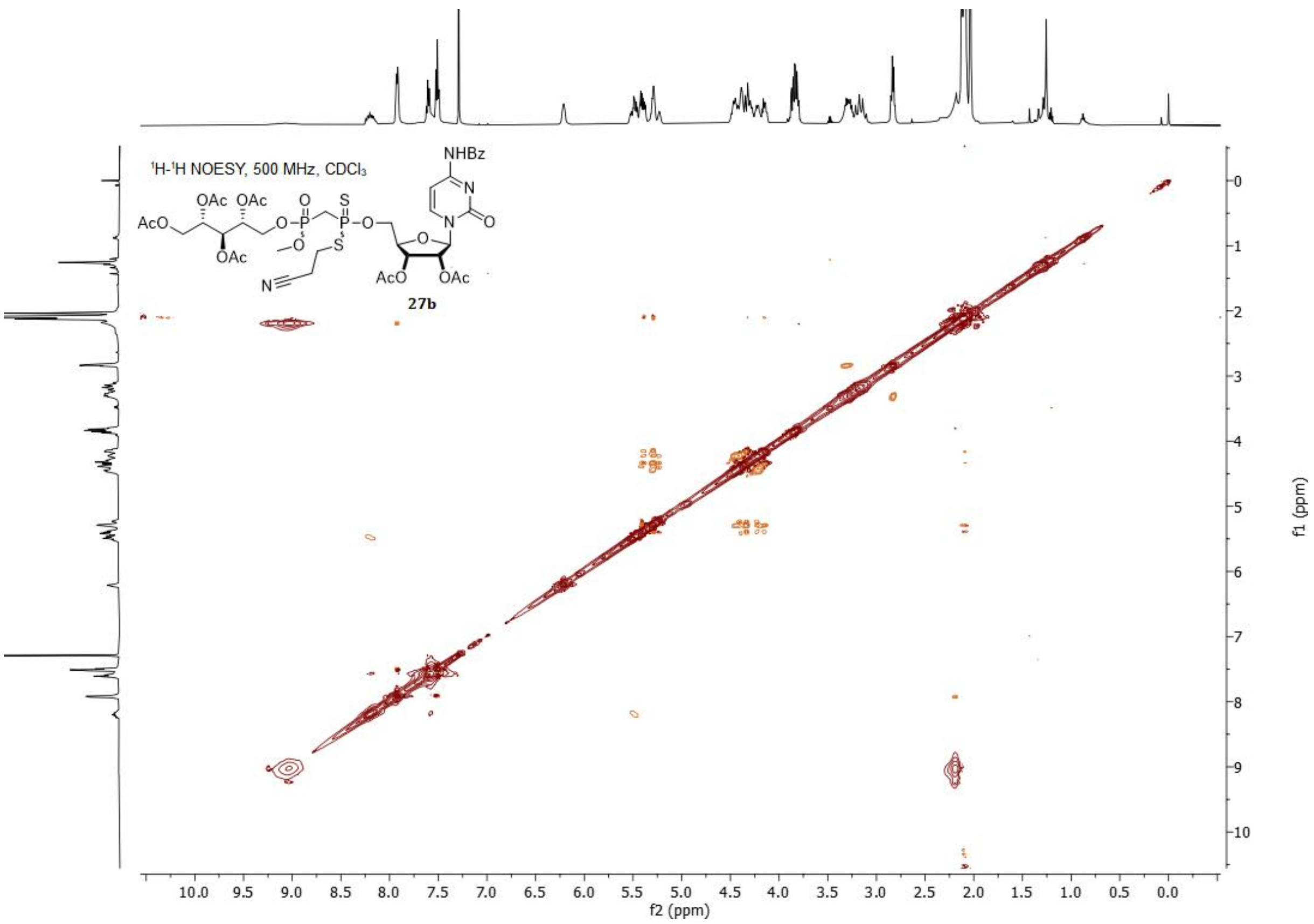
1H-1H NOESY, 500 MHz, CDCl3
NHBz
OAc OAc
AcO
OAc
AcO
OAc
27b
f2 (ppm)
f1 (ppm)

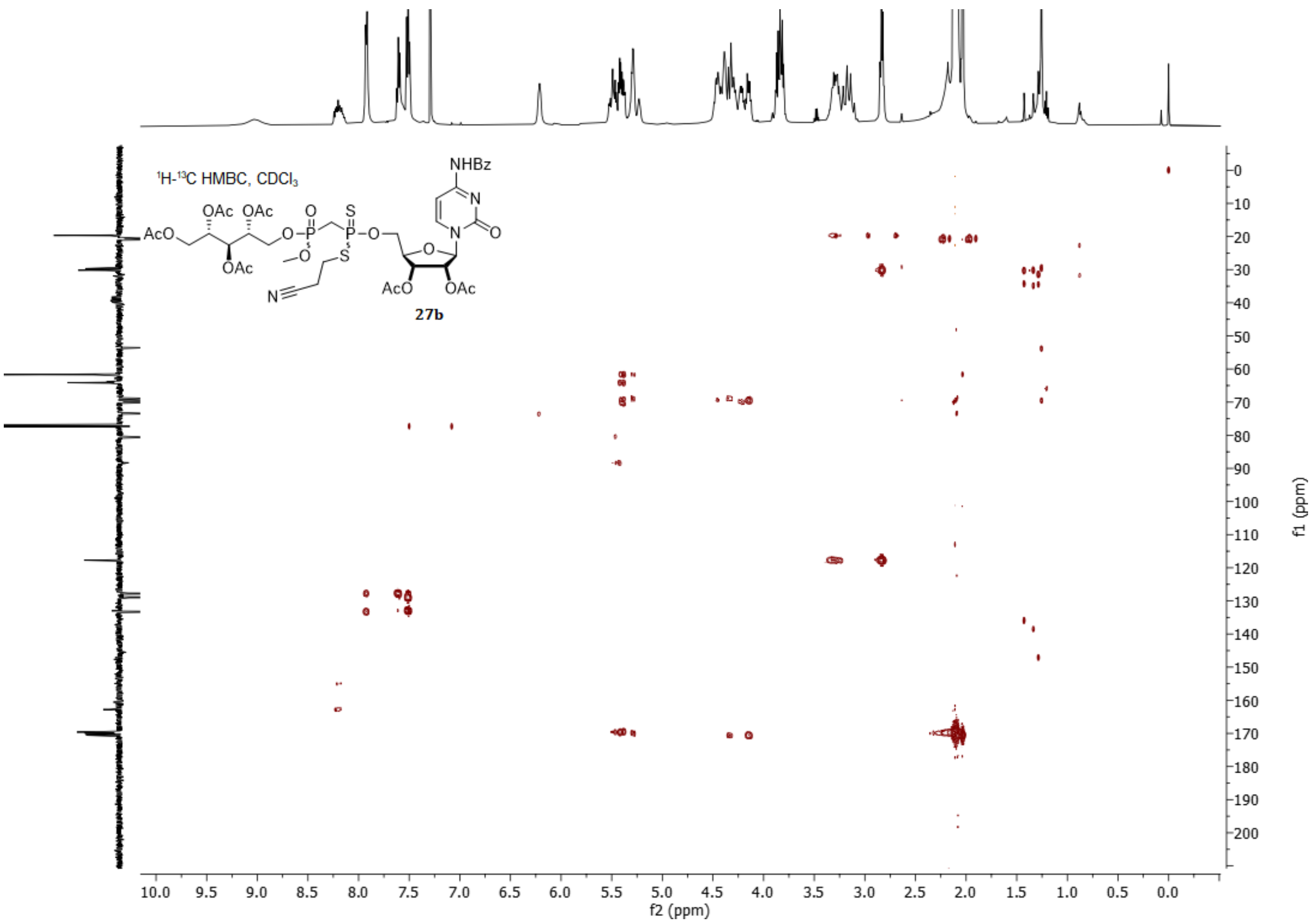

sn-3 me-CDP-αS-Gro 28a

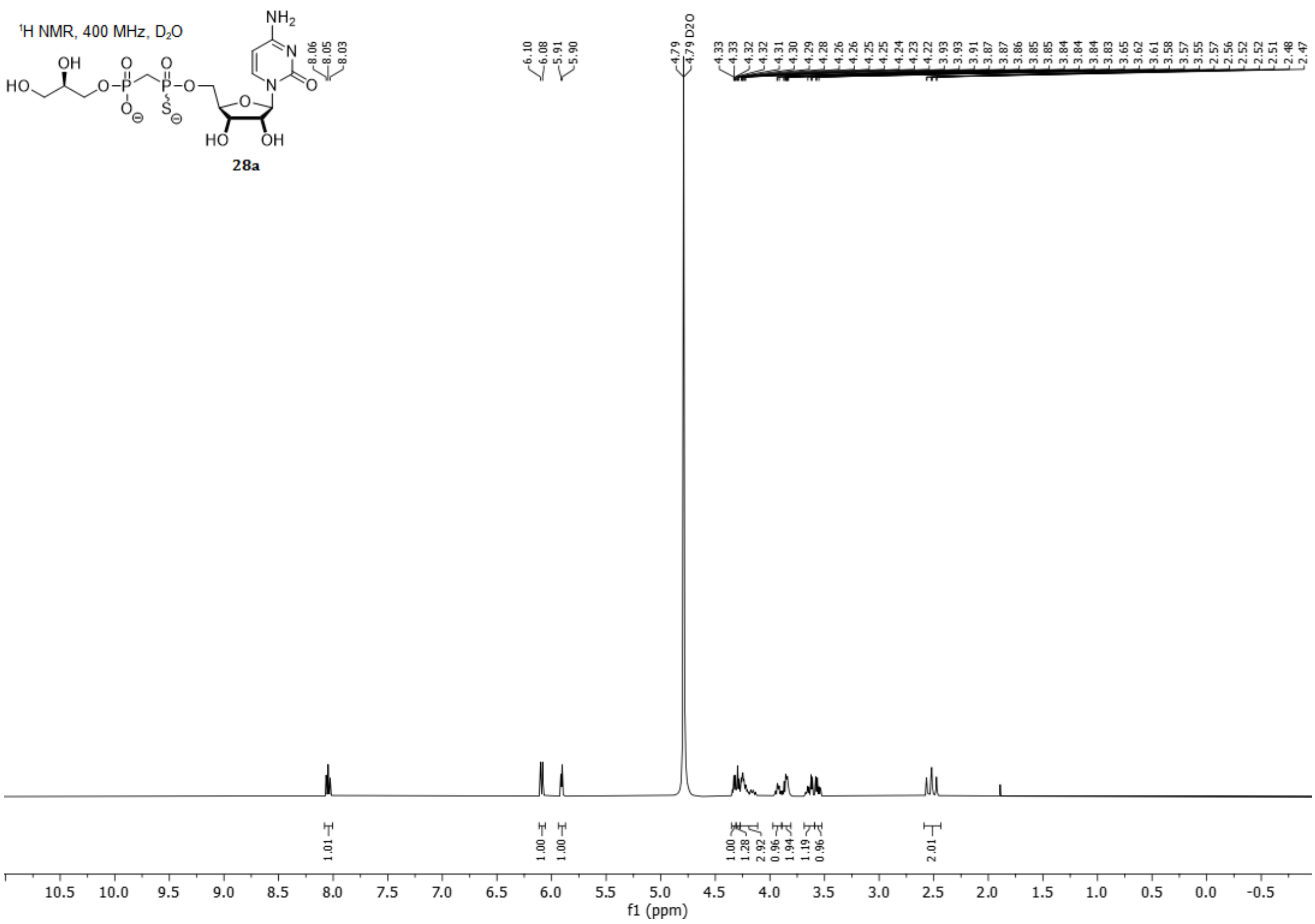

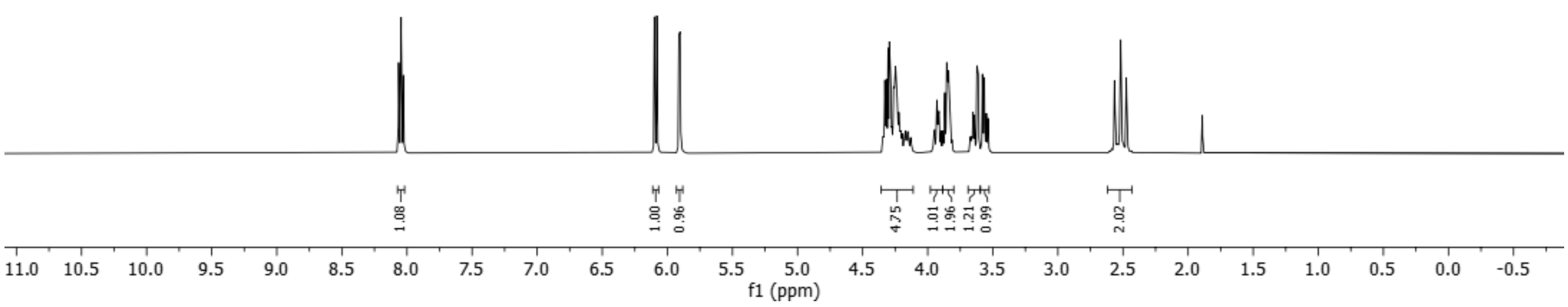

^{1}H Presat NMR, 400 MHz, D_2O

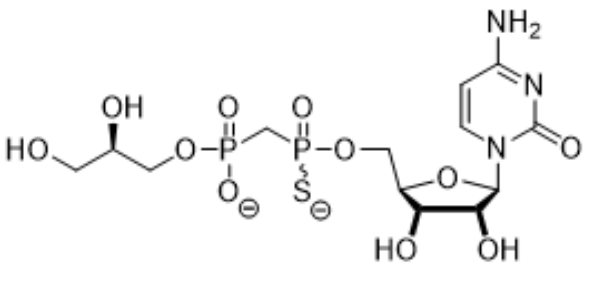

28a

^{13}C NMR, 101 MHz, D_2O

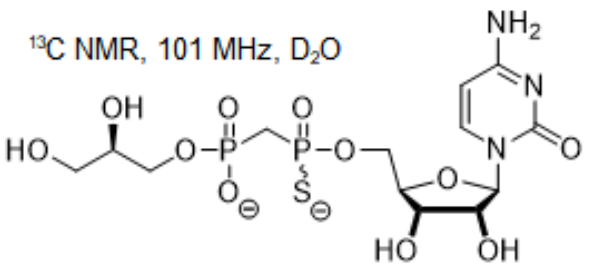

28a

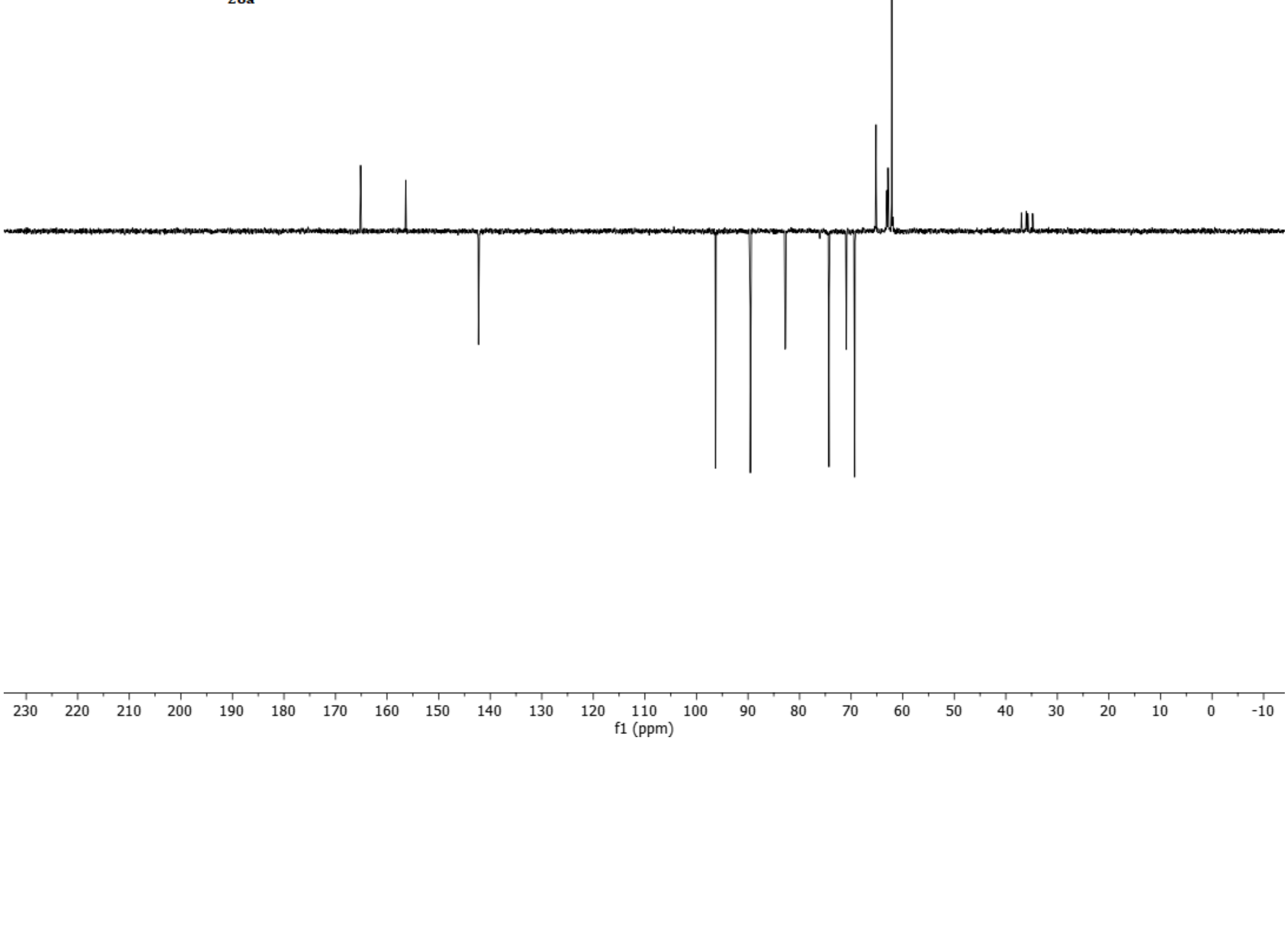

$^{31}P\{^{1}H\}$ NMR, 162 MHz, D_2O

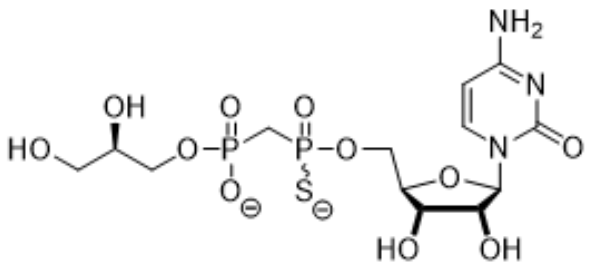

28a

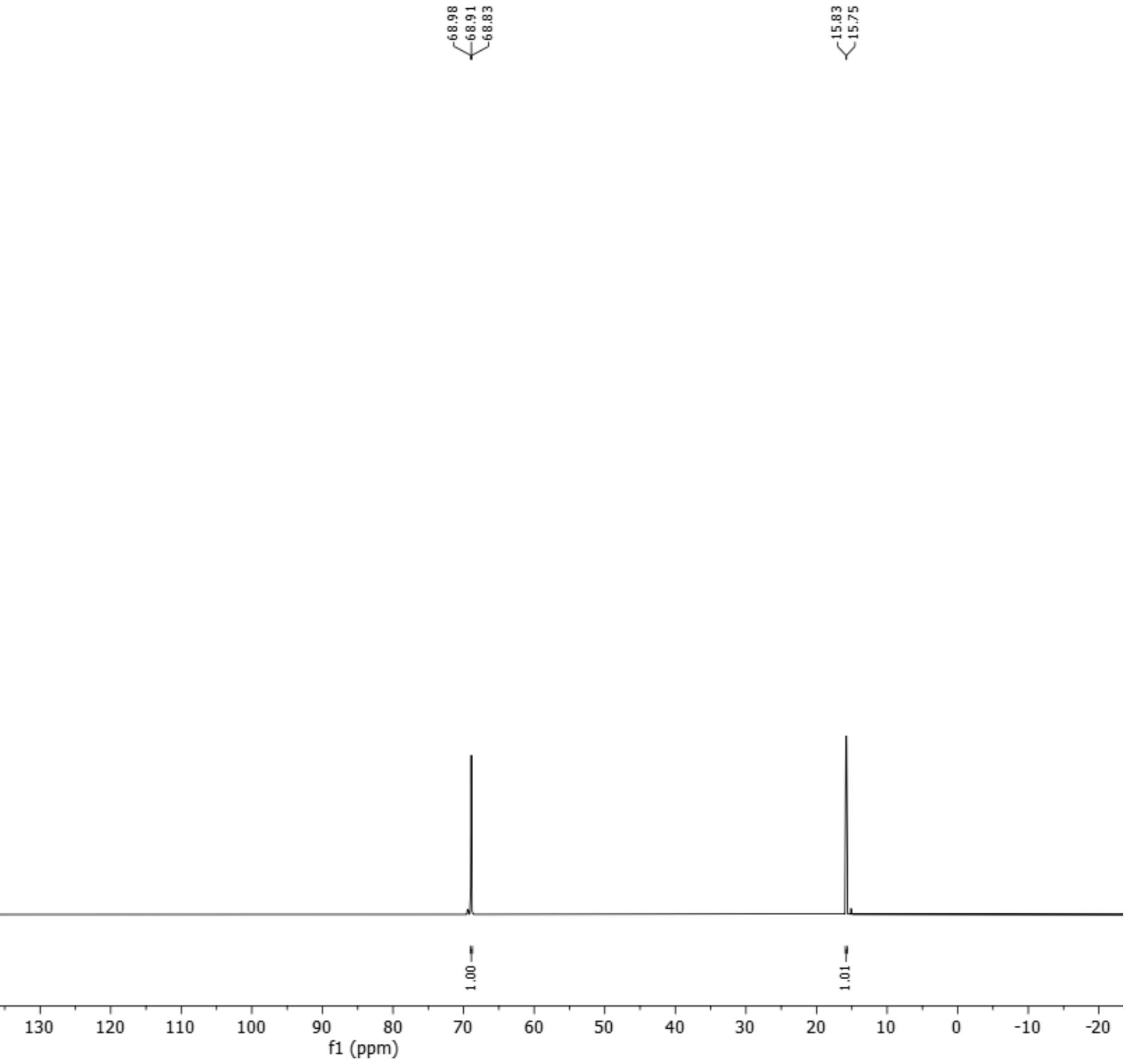

^{31}P NMR, 162 MHz, D_2O

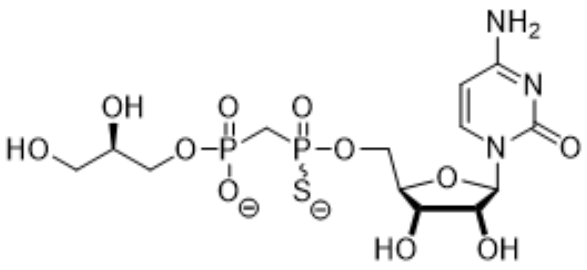

28a

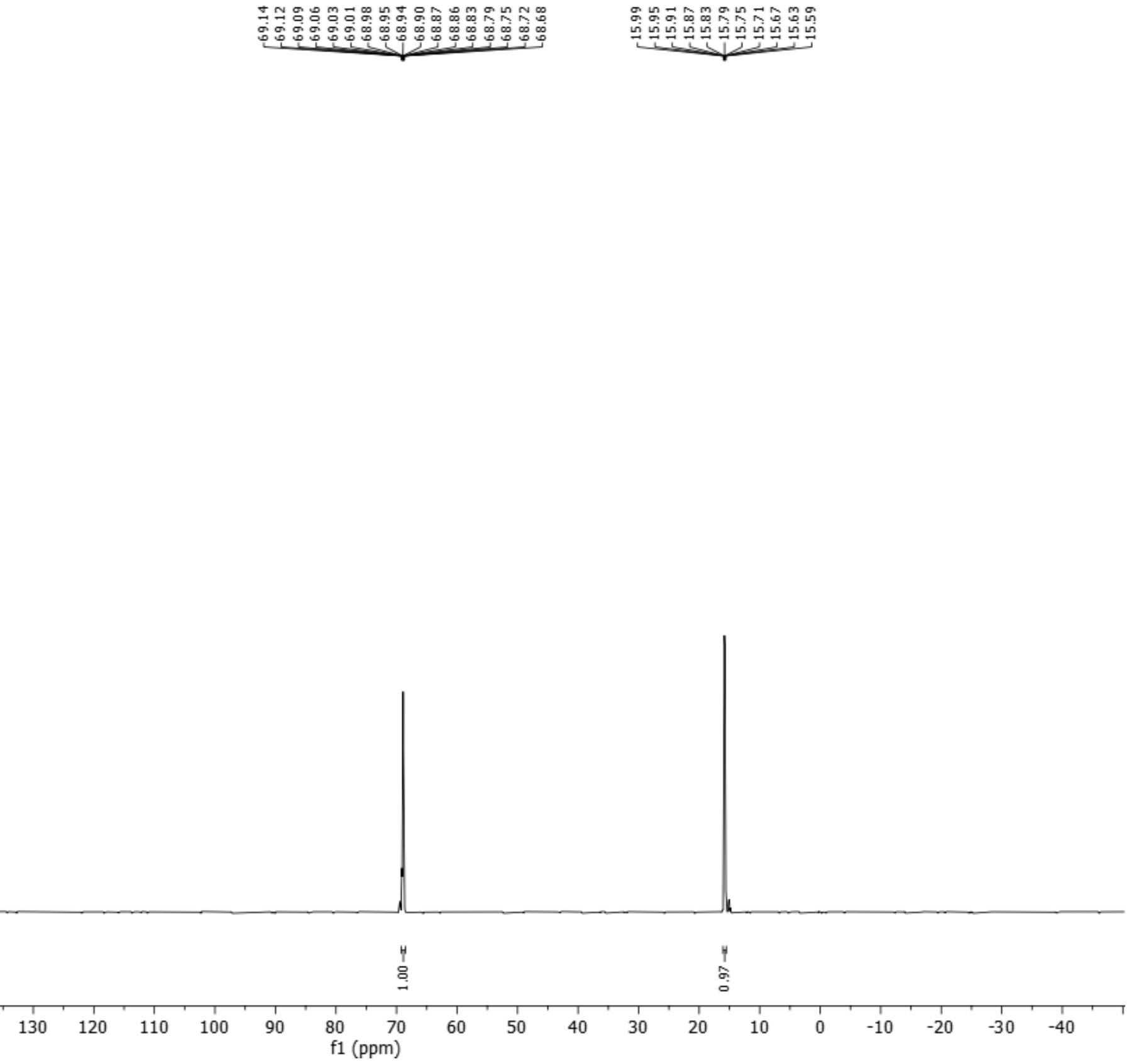

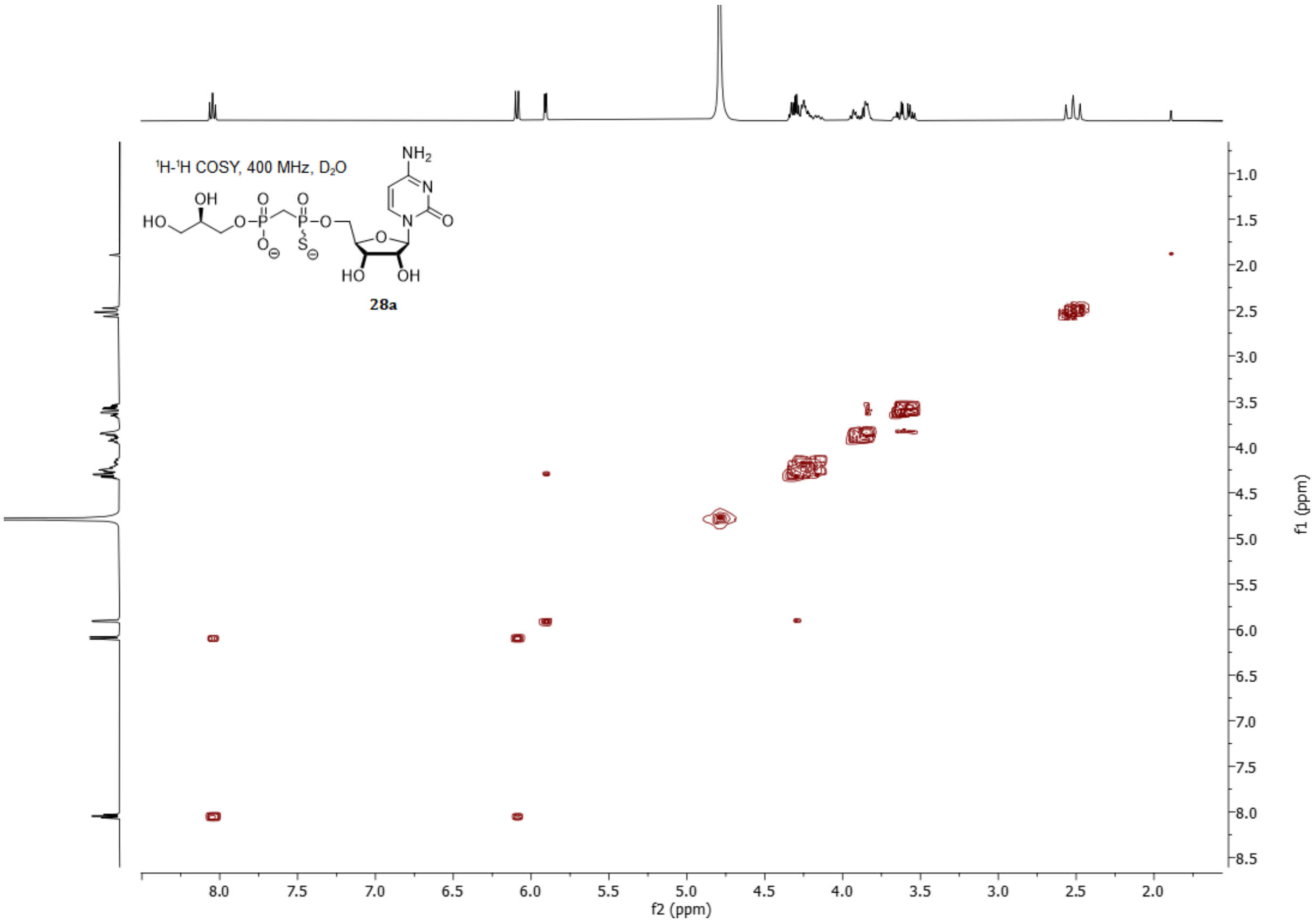

¹H-¹H COSY, 400 MHz, D₂O
NH2
N
OH
HO
O
P
O
P
O
O
N
O
O
S
OH
HO
28a
f2 (ppm)
f1 (ppm)

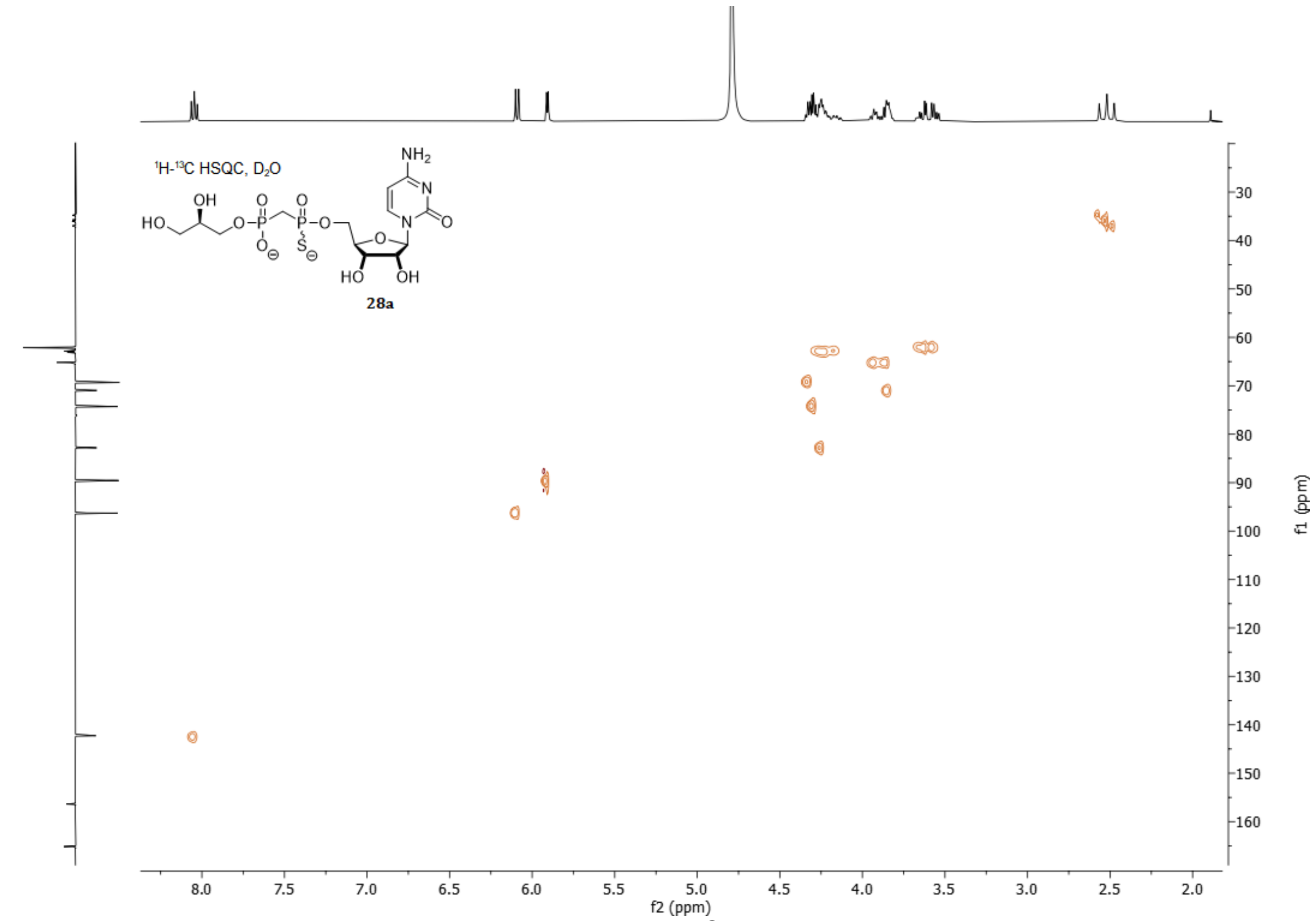

¹H-¹³C HSQC, D₂O
28a
f2 (ppm)
f1 (ppm)

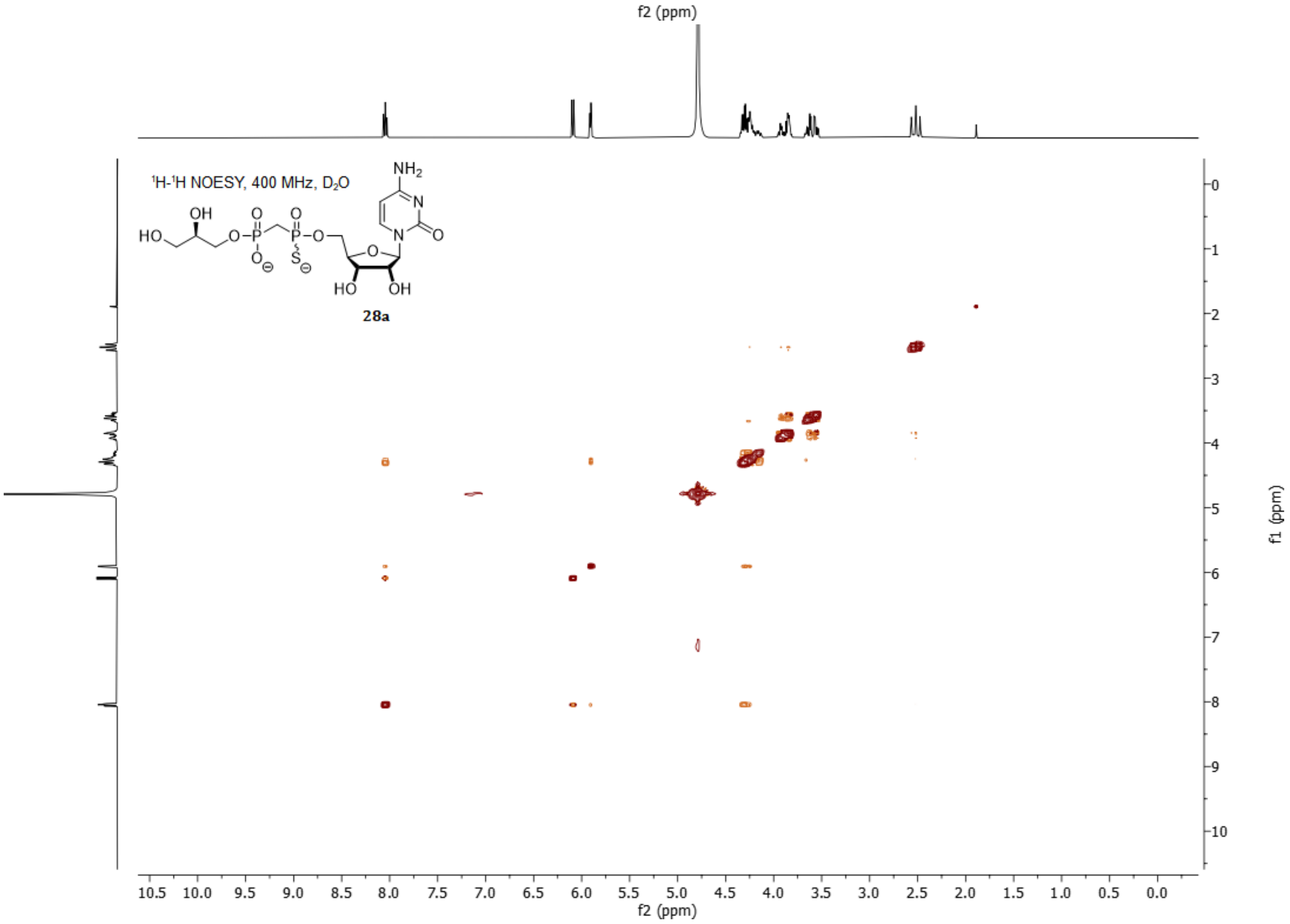

¹H-¹H NOESY, 400 MHz, D₂O
28a
f2 (ppm)
f1 (ppm)

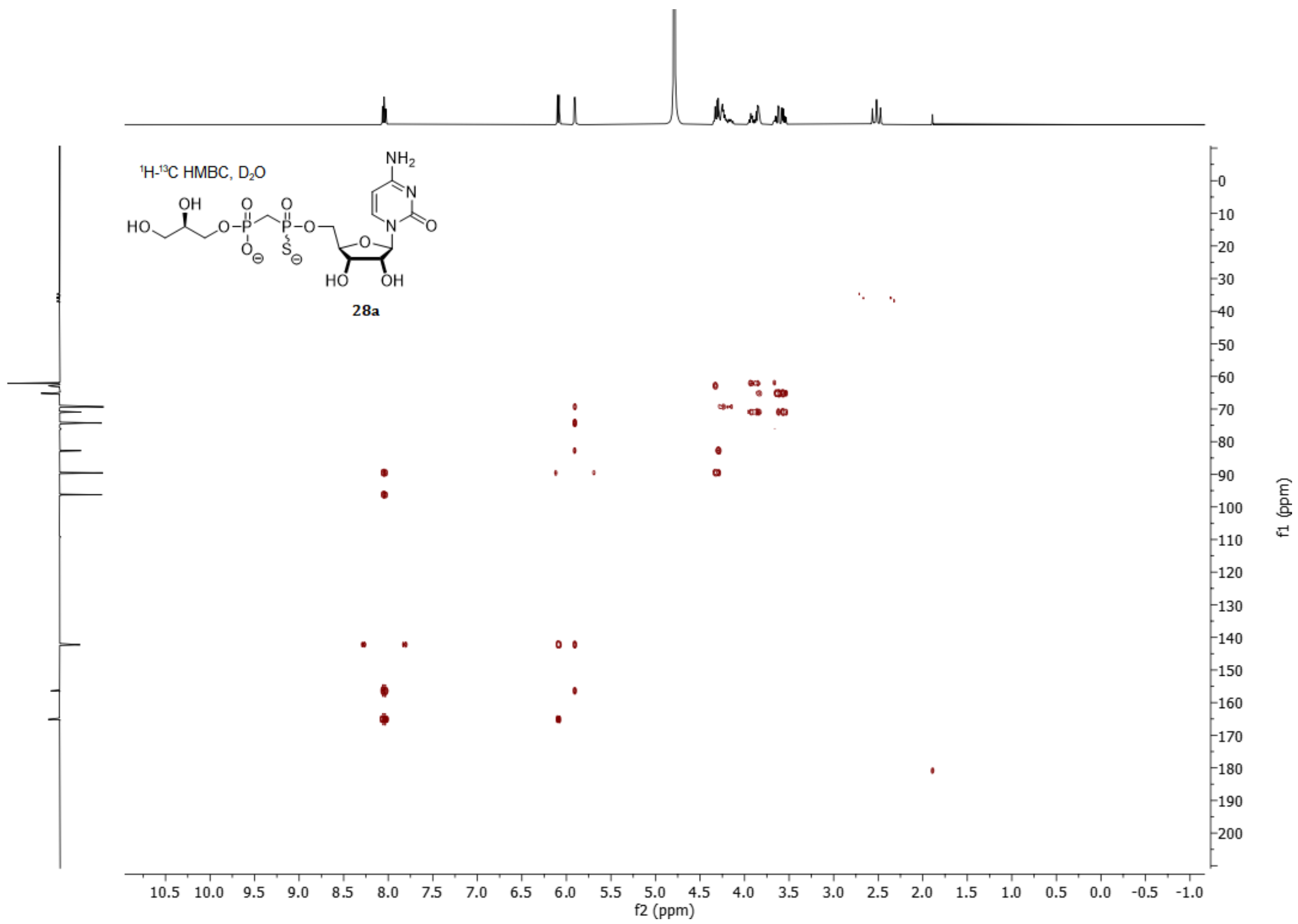
1H-13C HMBC, D2O
28a
f2 (ppm)
f1 (ppm)

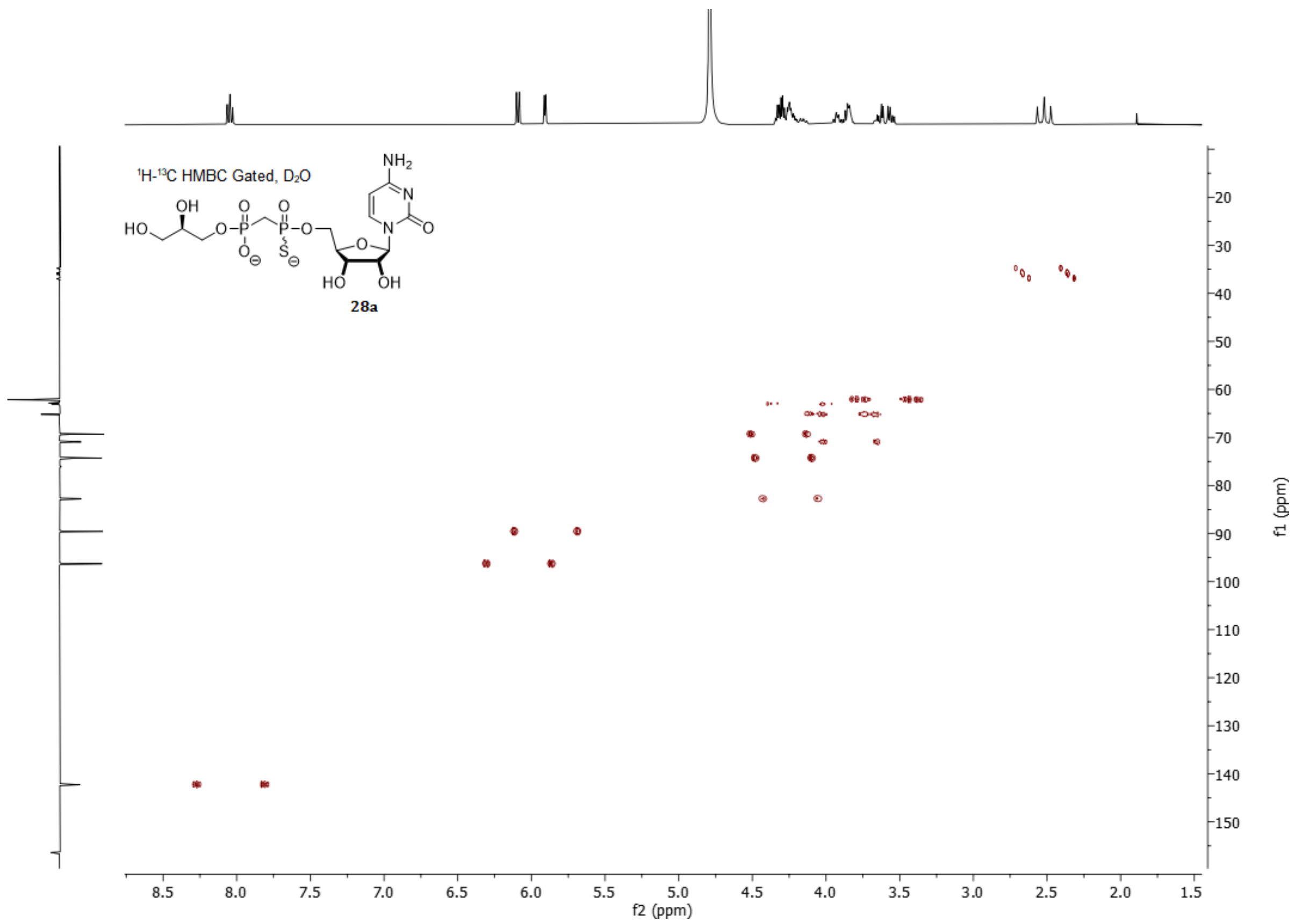
1H-13C HMBC Gated, D2O
28a
f2 (ppm)
f1 (ppm)

***sn*-3 me-CDP-αS$_2$ Gro 28**

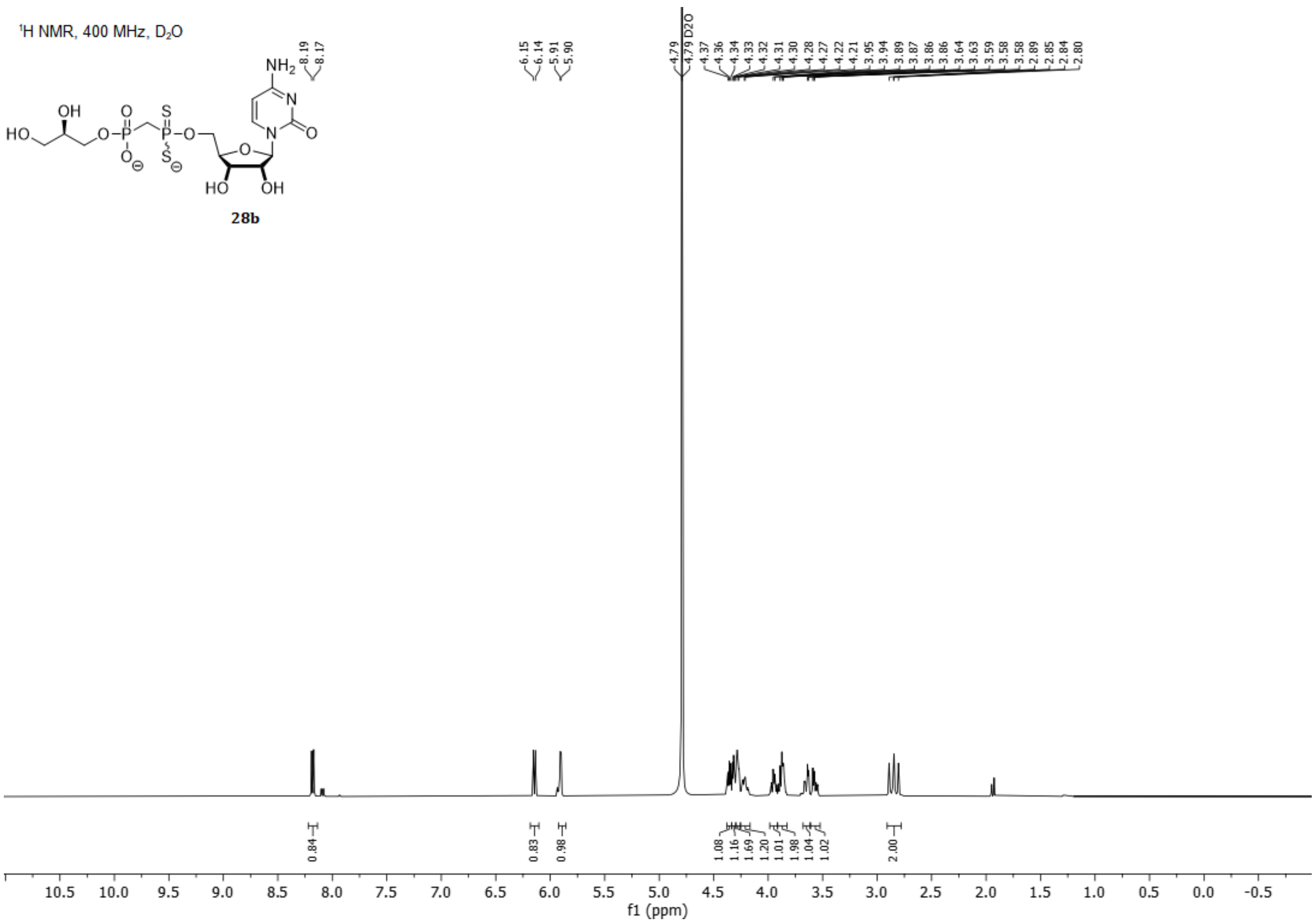

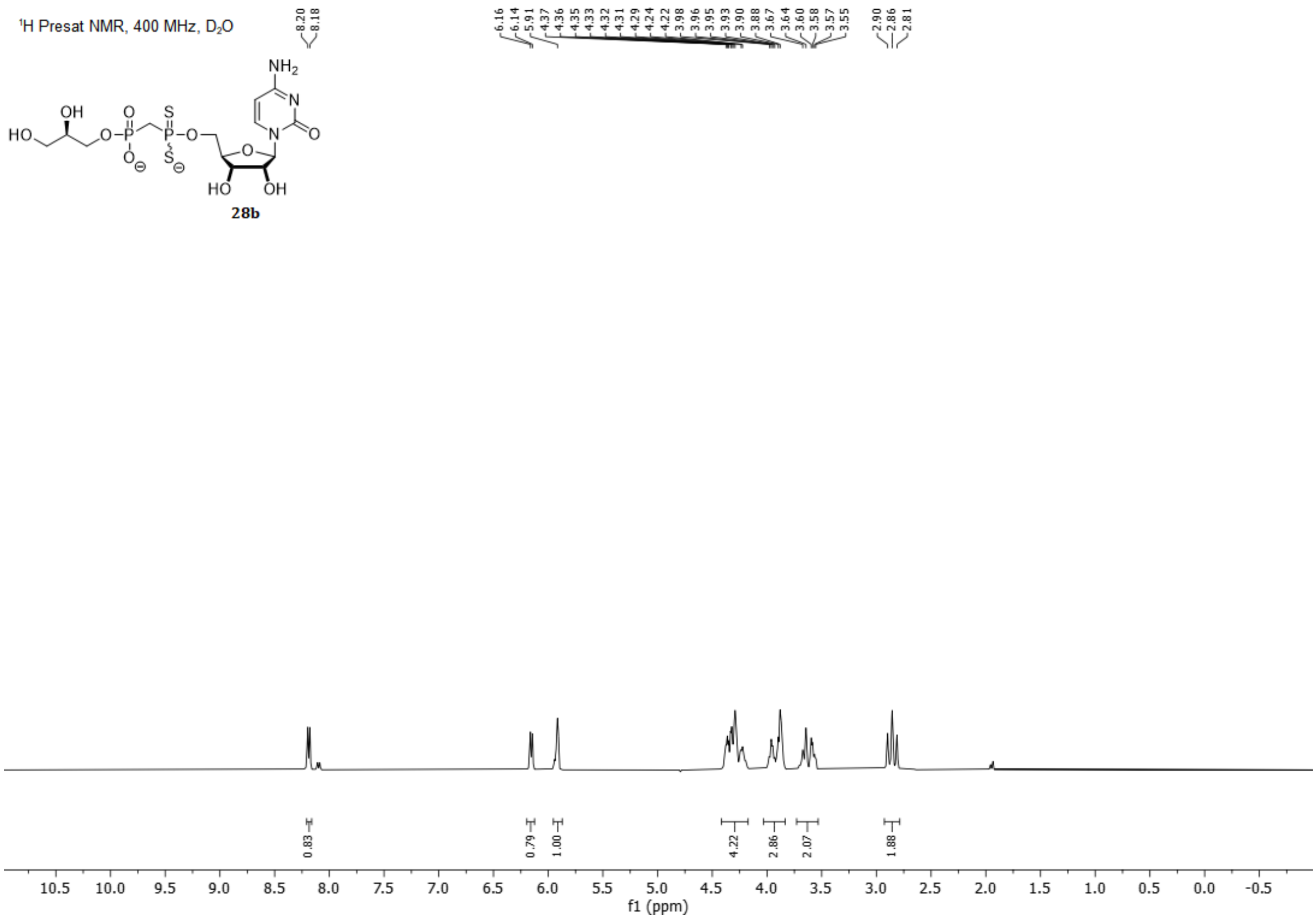

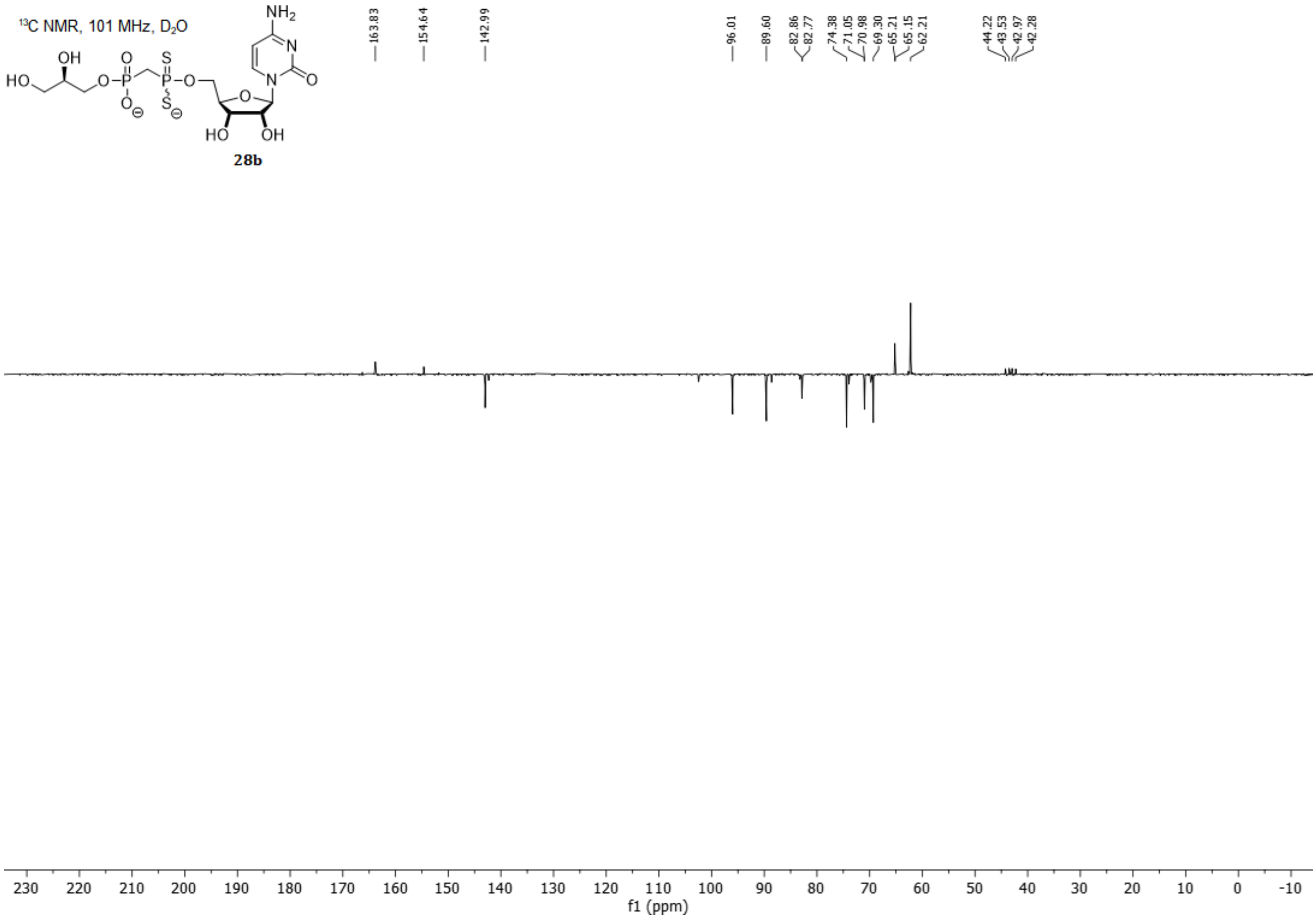

13C NMR, 101 MHz, D2O
28b
163.83
154.64
142.99
96.01
89.60
82.86
82.77
74.38
71.05
70.98
69.30
65.21
65.15
62.21
44.22
43.53
42.97
42.28
230 220 210 200 190 180 170 160 150 140 130 120 110 100 90 80 70 60 50 40 30 20 10 0 -10
f1 (ppm)

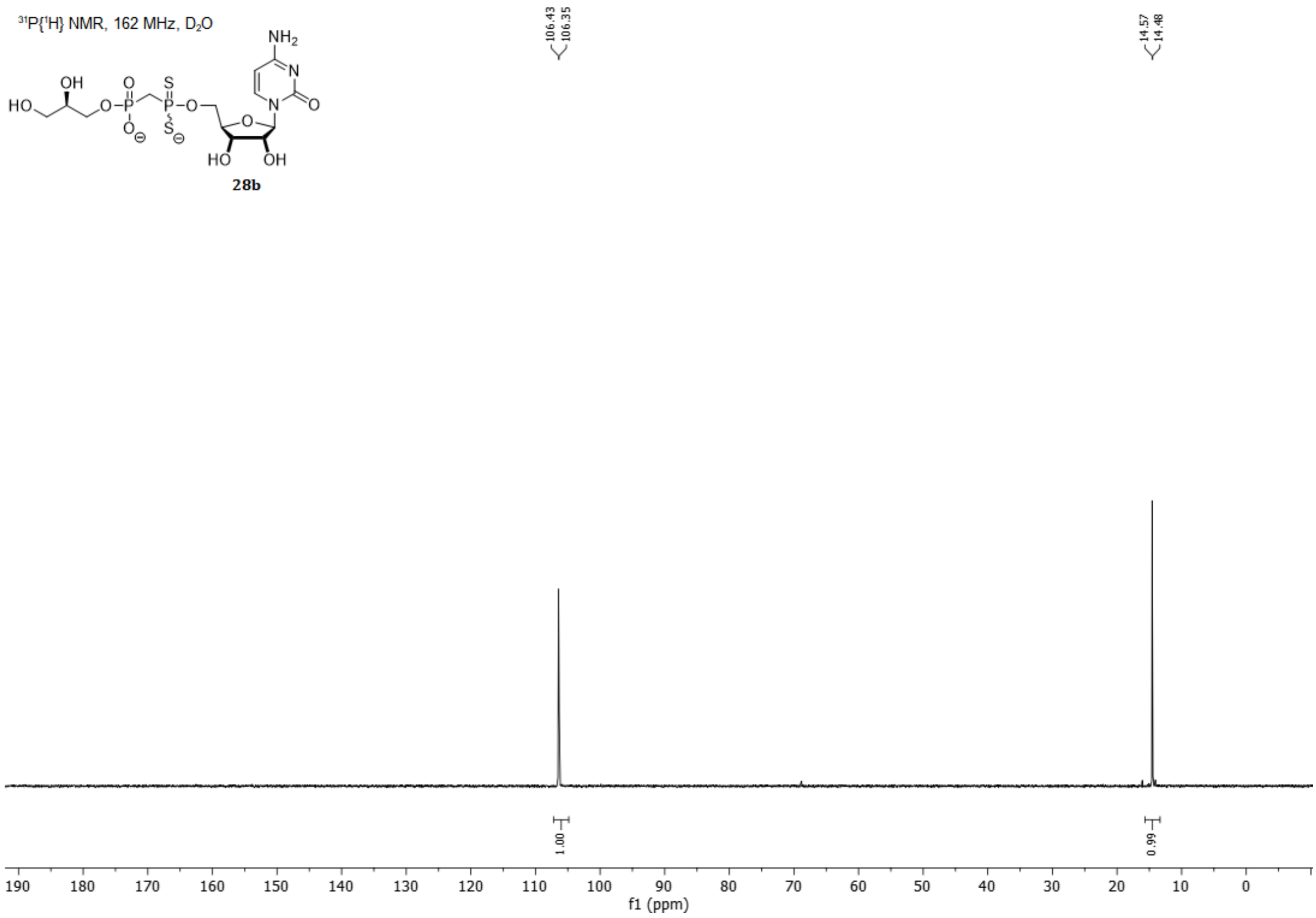

31P{1H} NMR, 162 MHz, D2O
28b
106.43
106.35
14.57
14.48
1.00
0.99
190 180 170 160 150 140 130 120 110 100 90 80 70 60 50 40 30 20 10 0
f1 (ppm)

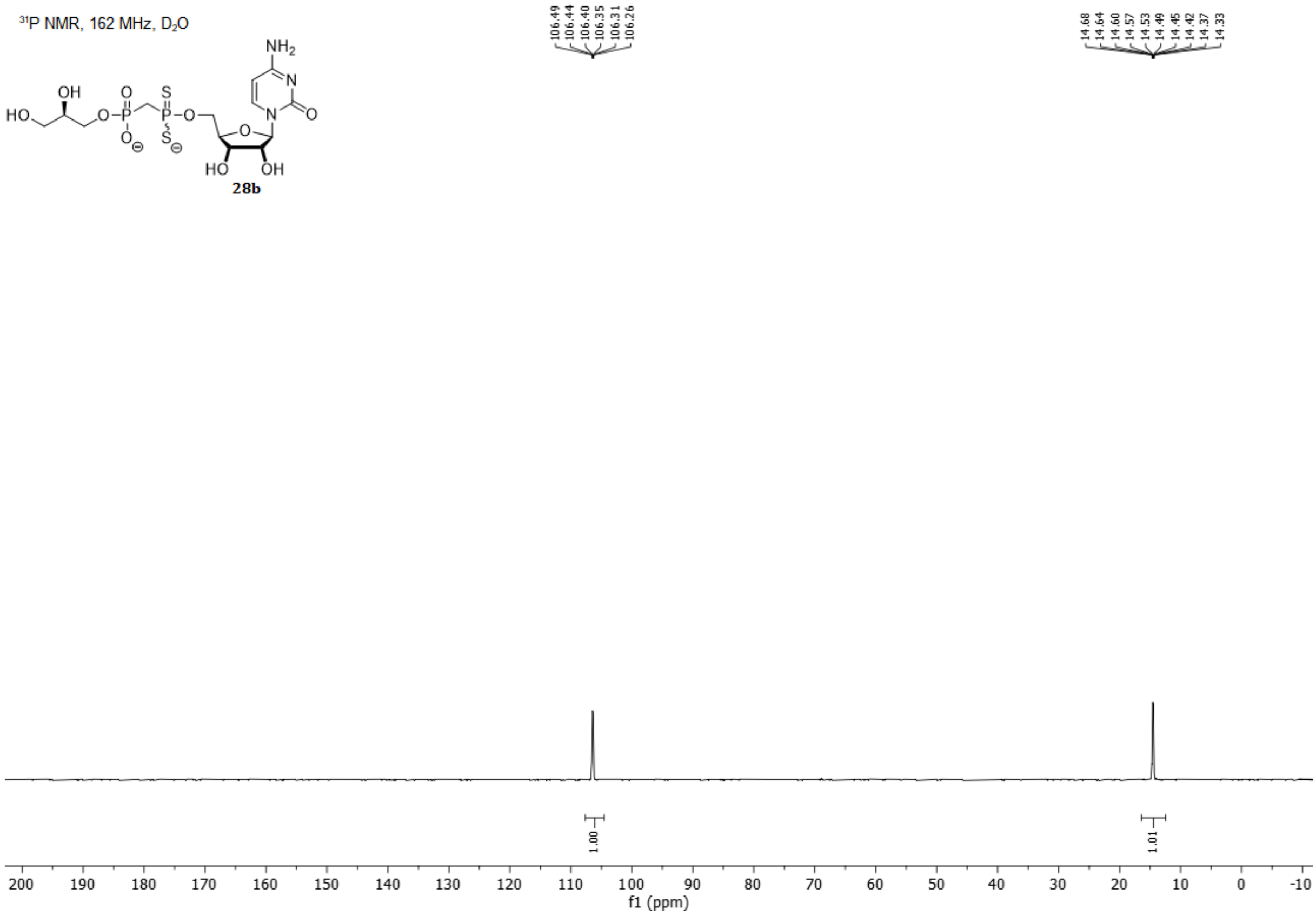

31P NMR, 162 MHz, D2O
28b
106.49
106.44
106.40
106.35
106.31
106.26
14.68
14.64
14.60
14.57
14.53
14.49
14.45
14.42
14.37
14.33
1.00
1.01
f1 (ppm)

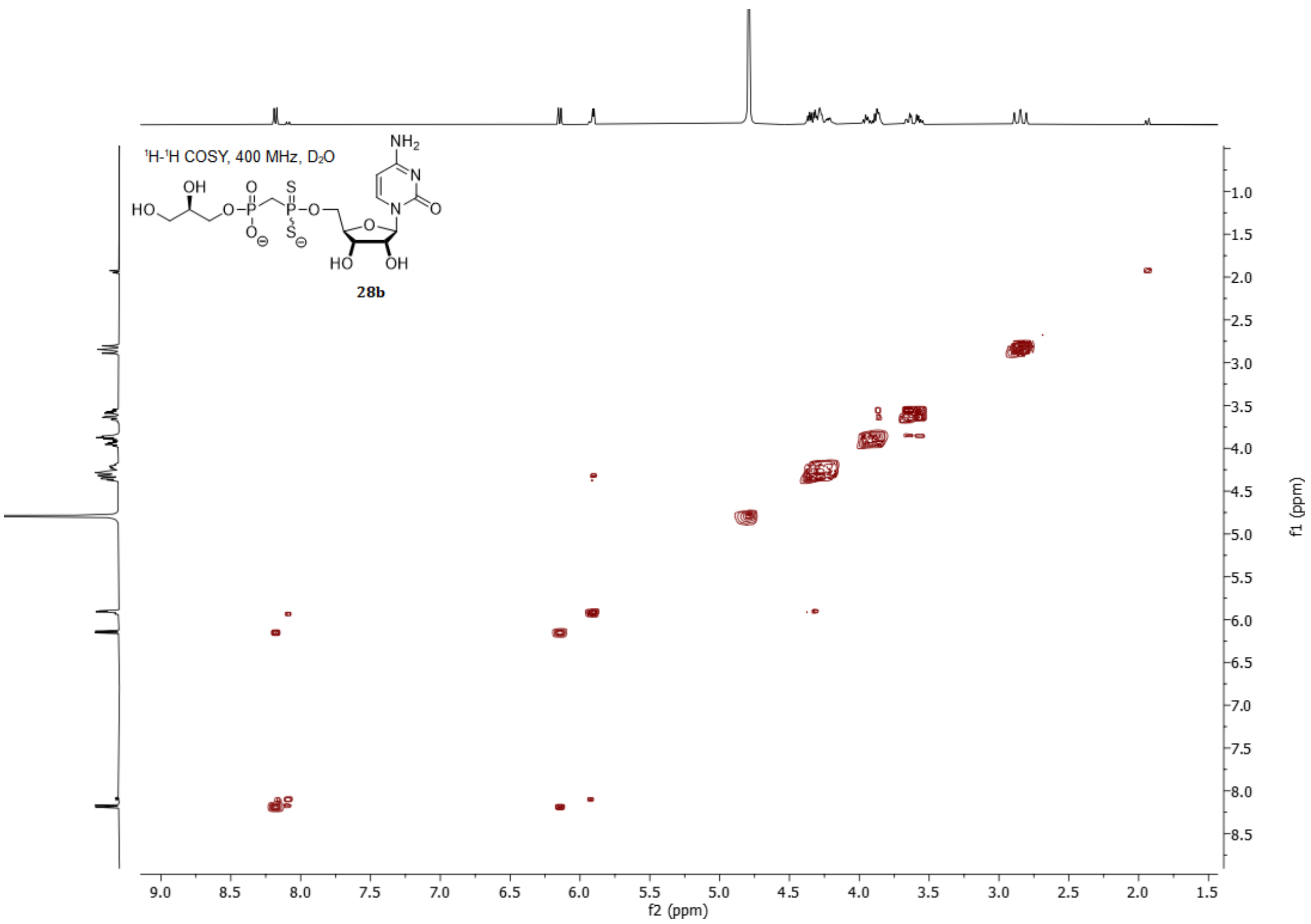

1H-1H COSY, 400 MHz, D2O
28b
f2 (ppm)
f1 (ppm)

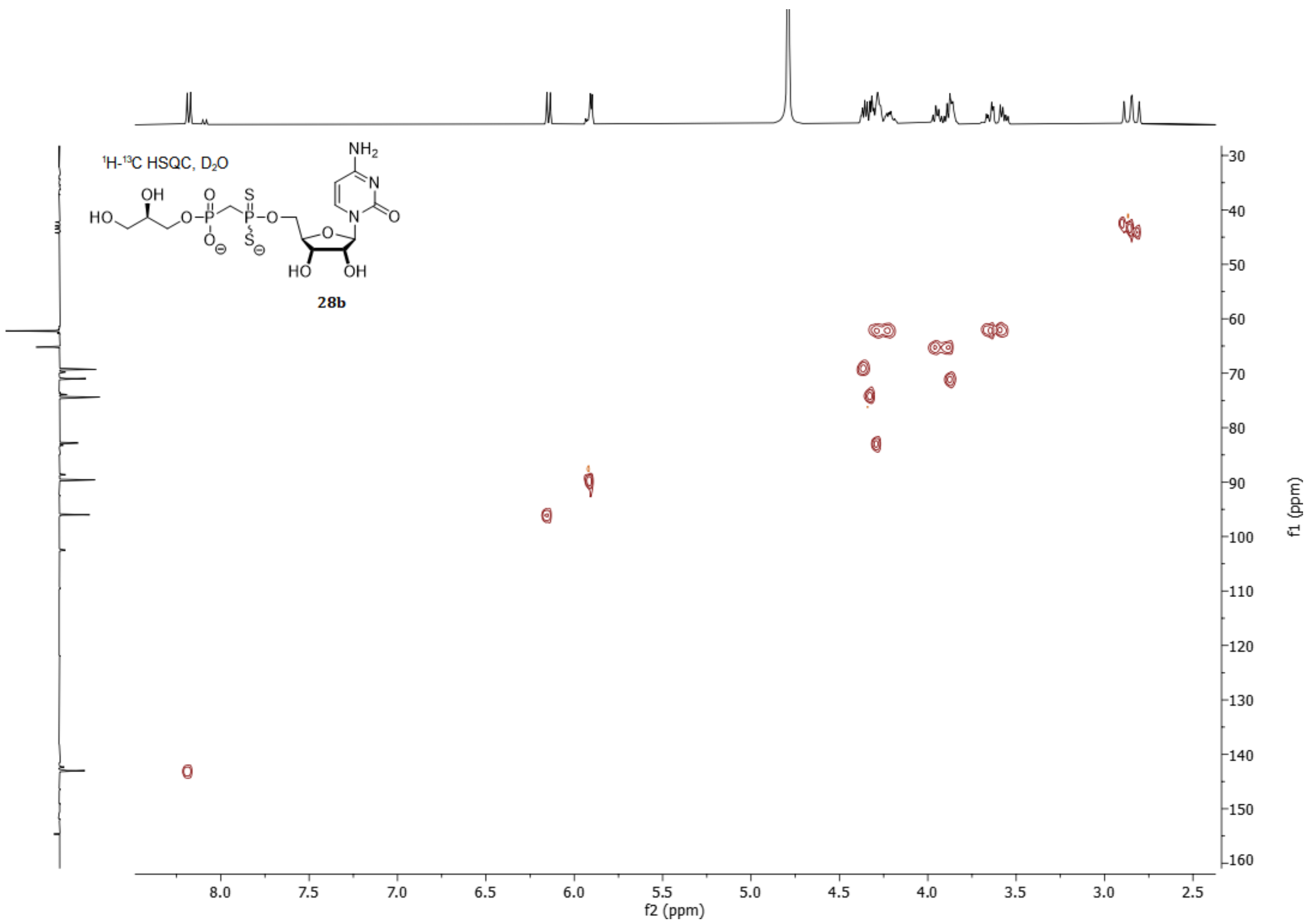
^{1}H-^{13}C HSQC, D$_2$O
NH$_2$
HO
OH
OH
HO
28b
f2 (ppm)
f1 (ppm)
8.0 7.5 7.0 6.5 6.0 5.5 5.0 4.5 4.0 3.5 3.0 2.5
30 40 50 60 70 80 90 100 110 120 130 140 150 160

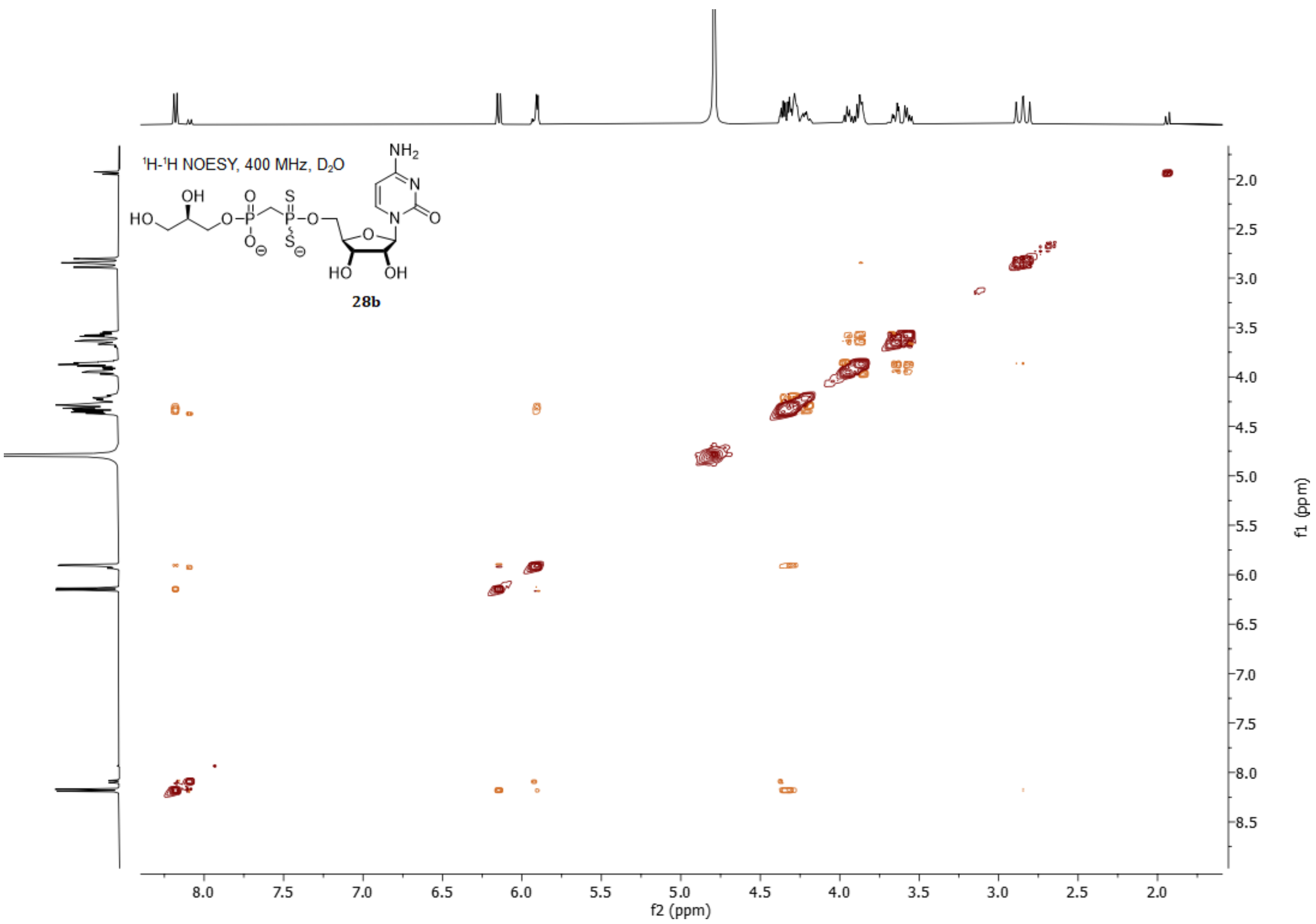
^{1}H-^{1}H NOESY, 400 MHz, D$_2$O
NH$_2$
HO
OH
OH
HO
28b
f2 (ppm)
f1 (ppm)
8.0 7.5 7.0 6.5 6.0 5.5 5.0 4.5 4.0 3.5 3.0 2.5 2.0
2.0 2.5 3.0 3.5 4.0 4.5 5.0 5.5 6.0 6.5 7.0 7.5 8.0 8.5

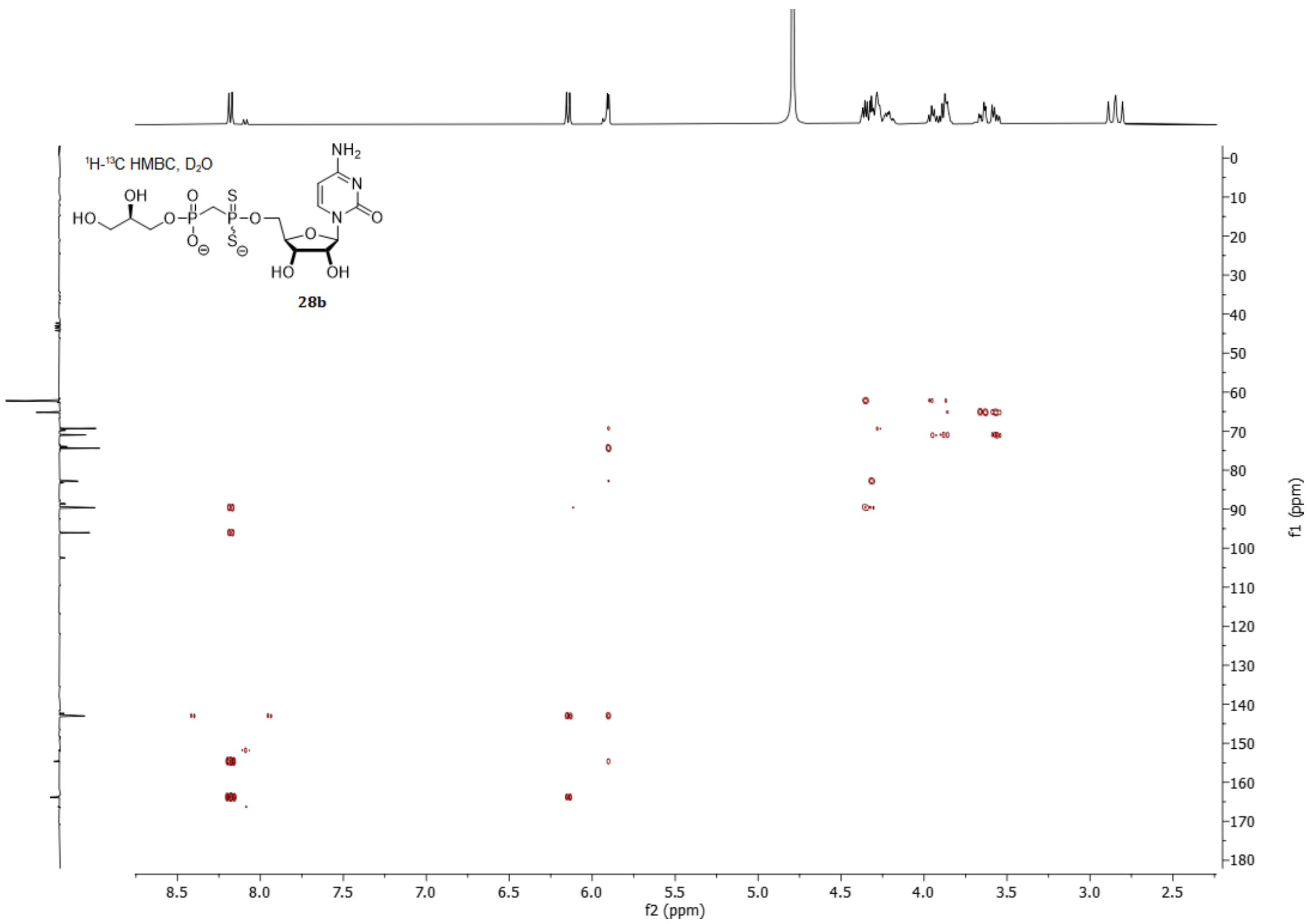

***sn*-1 me-CDP-αS-Gro 29a**

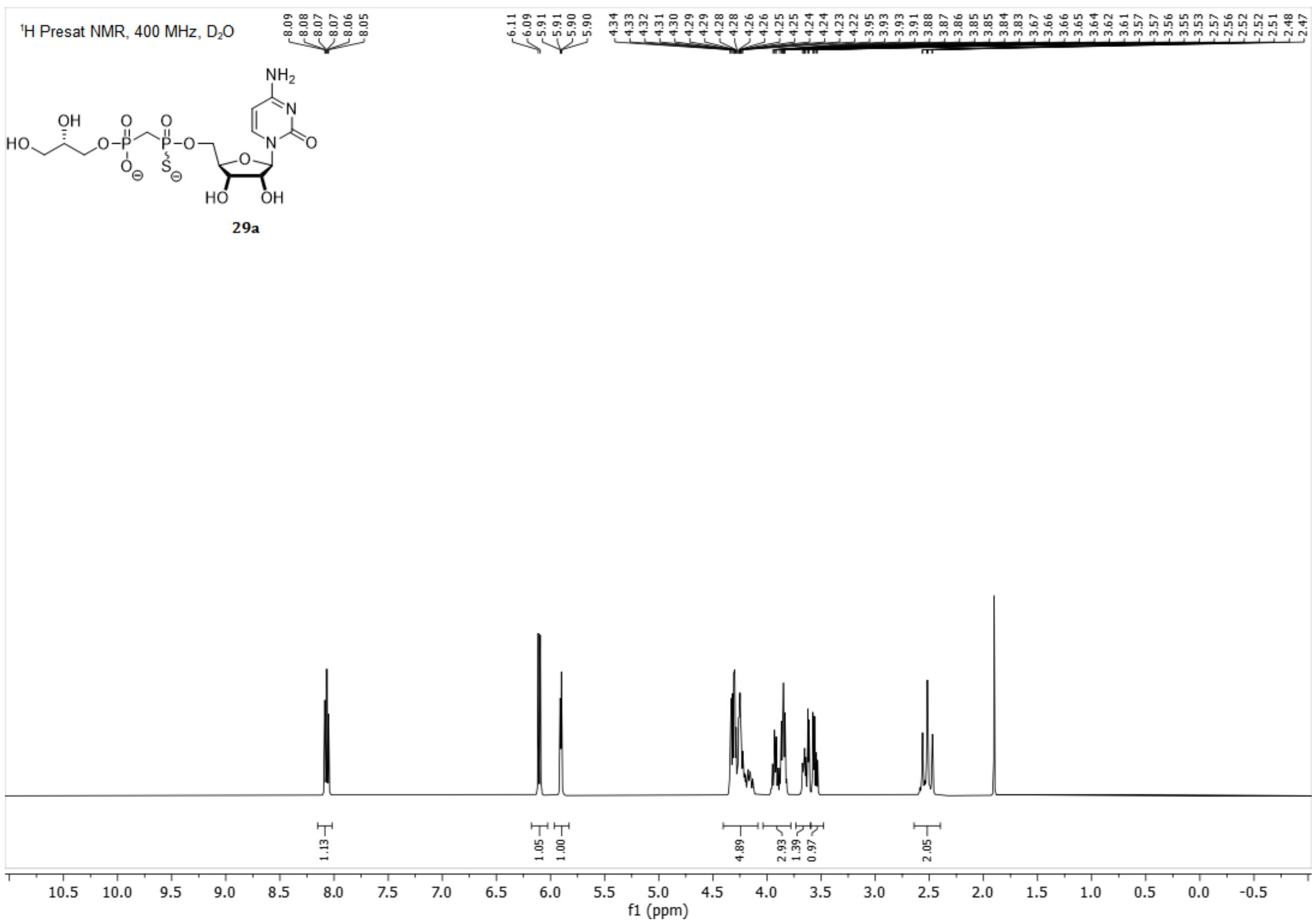

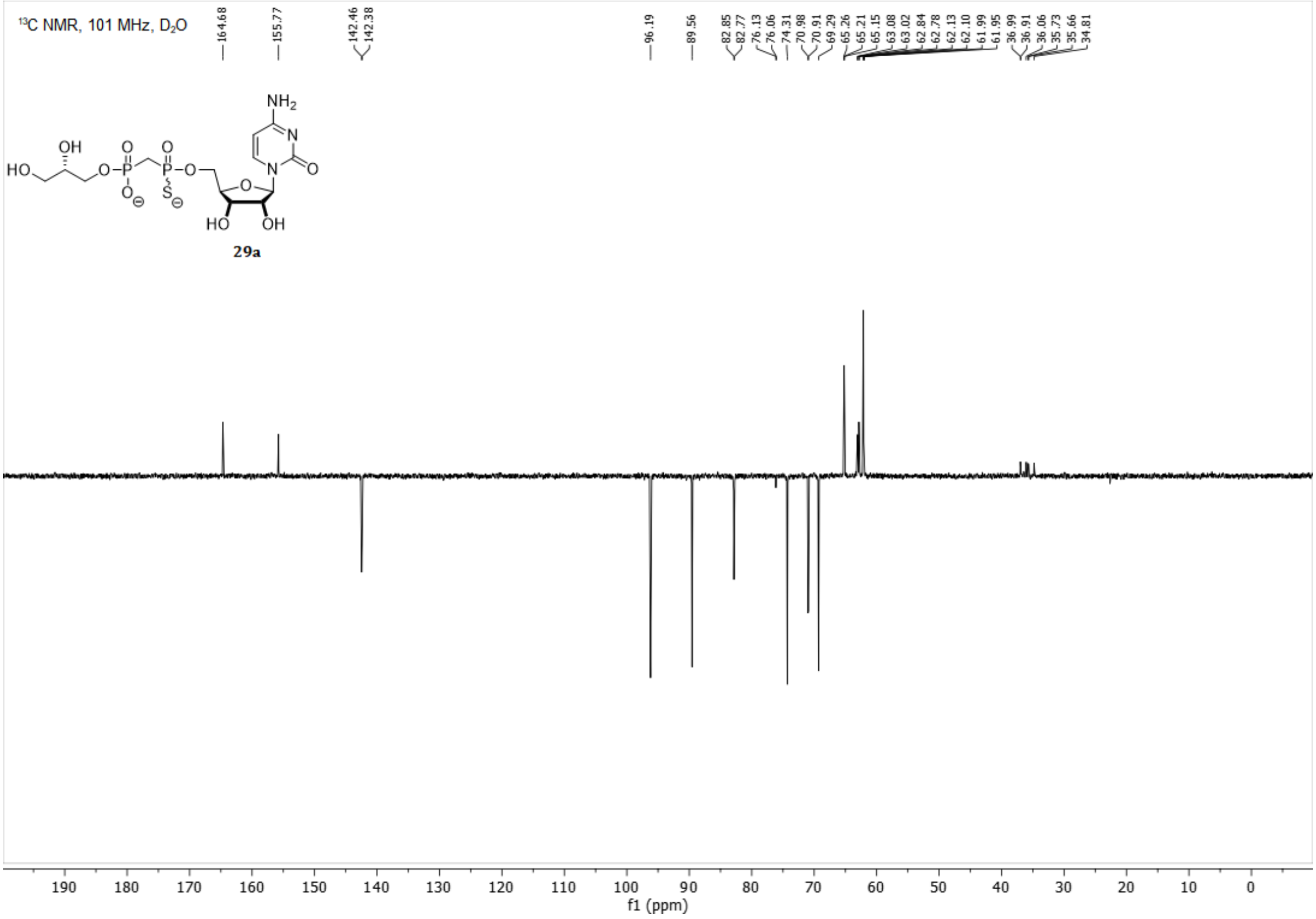

13C NMR, 101 MHz, D2O
164.68
155.77
142.46
142.38
96.19
89.56
82.85
82.77
76.13
76.06
74.31
70.98
70.91
69.29
65.26
65.21
65.15
63.08
63.02
62.84
62.78
62.13
62.10
61.99
61.95
36.99
36.91
36.06
35.73
35.66
34.81
NH2
OH
HO
O
N
S
HO
OH
29a
f1 (ppm)

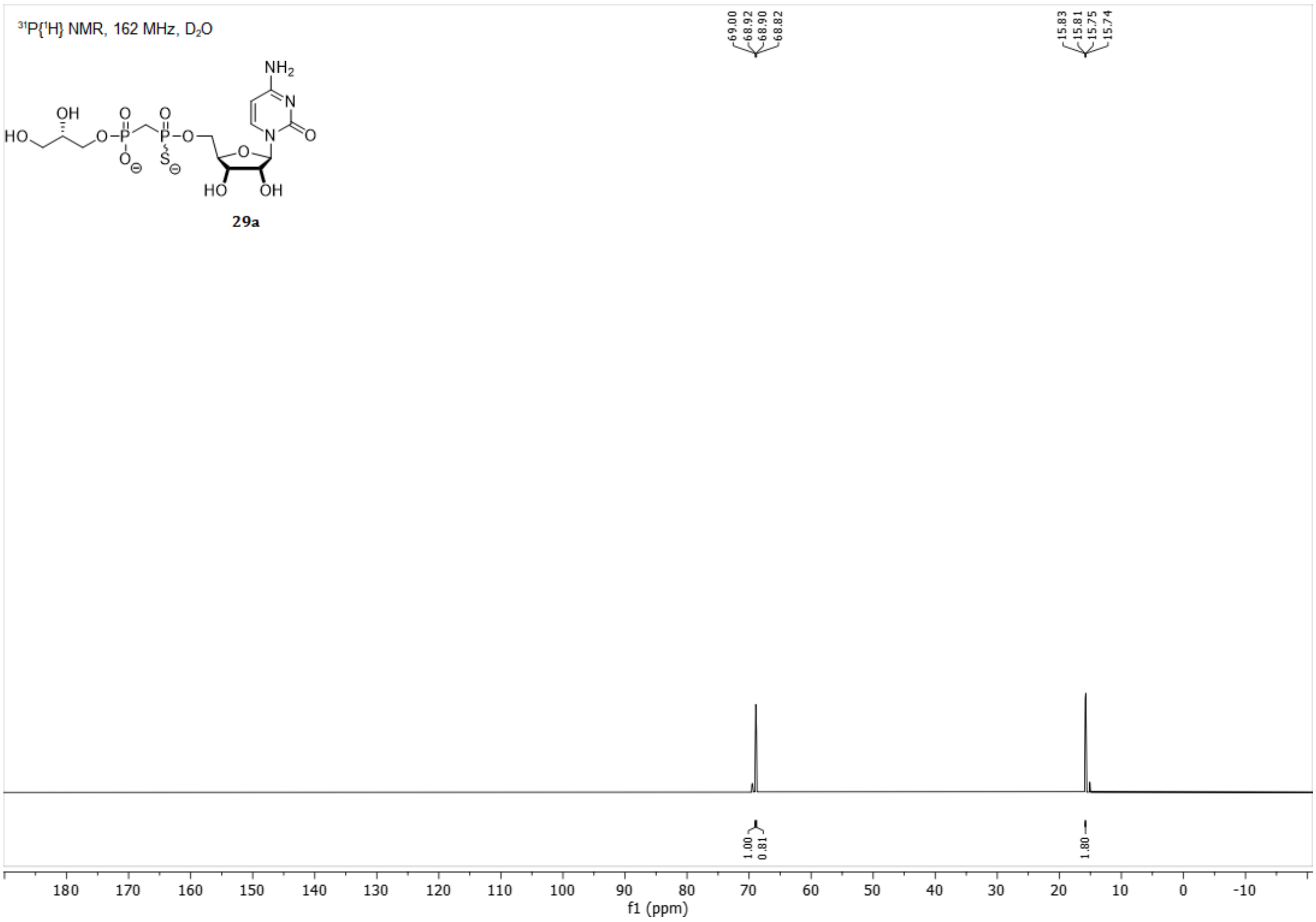

31P{1H} NMR, 162 MHz, D2O
69.00
68.92
68.90
68.82
15.83
15.81
15.75
15.74
NH2
OH
HO
O
N
S
HO
OH
29a
1.00
0.81
1.80
f1 (ppm)

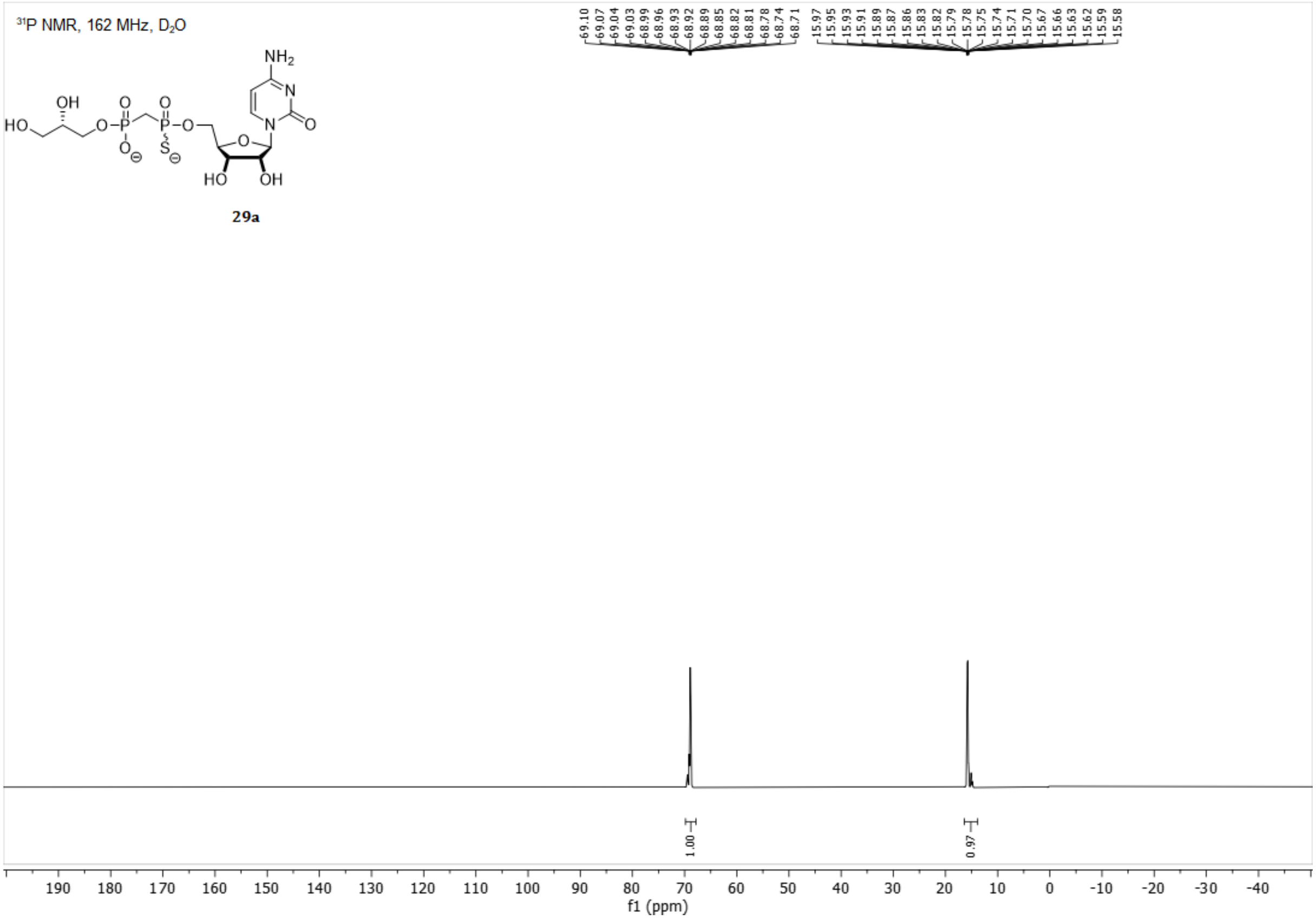

³¹P NMR, 162 MHz, D₂O
29a
f1 (ppm)

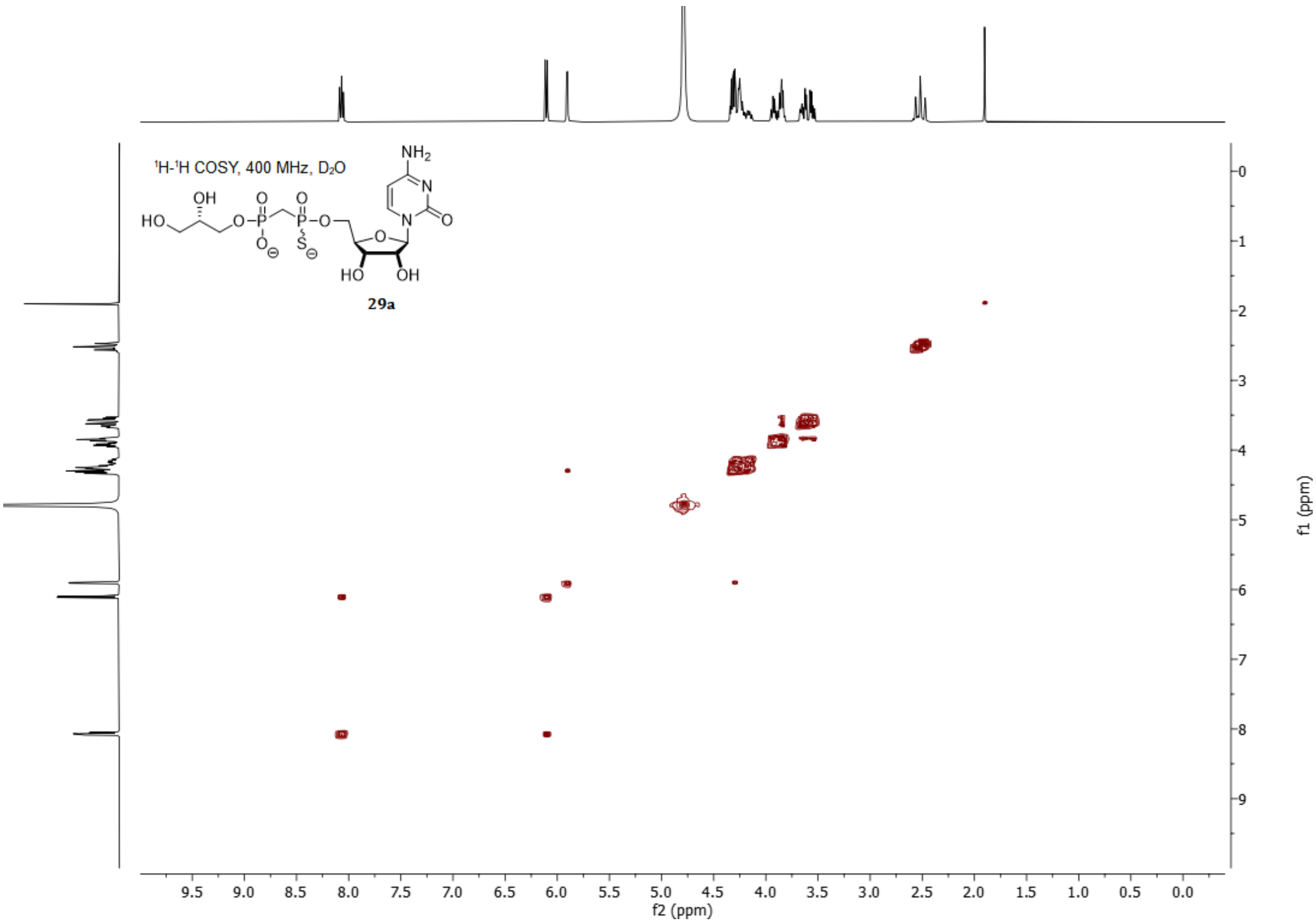

¹H-¹H COSY, 400 MHz, D₂O
29a
f2 (ppm)
f1 (ppm)

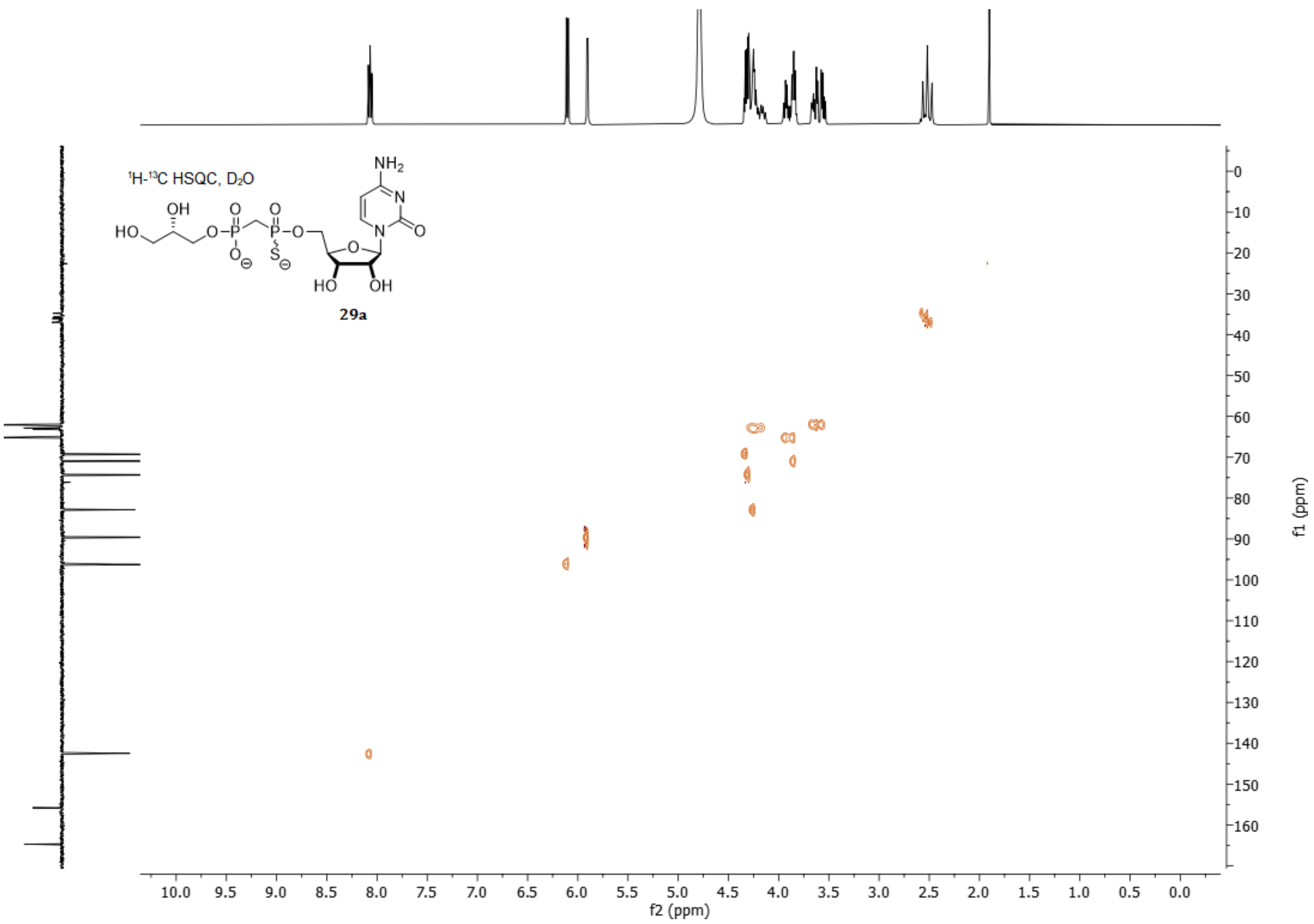

¹H-¹³C HSQC, D₂O
NH₂
OH
HO
O
P
O
S
N
OH
29a
f1 (ppm)
f2 (ppm)
10.0 9.5 9.0 8.5 8.0 7.5 7.0 6.5 6.0 5.5 5.0 4.5 4.0 3.5 3.0 2.5 2.0 1.5 1.0 0.5 0.0
0 10 20 30 40 50 60 70 80 90 100 110 120 130 140 150 160

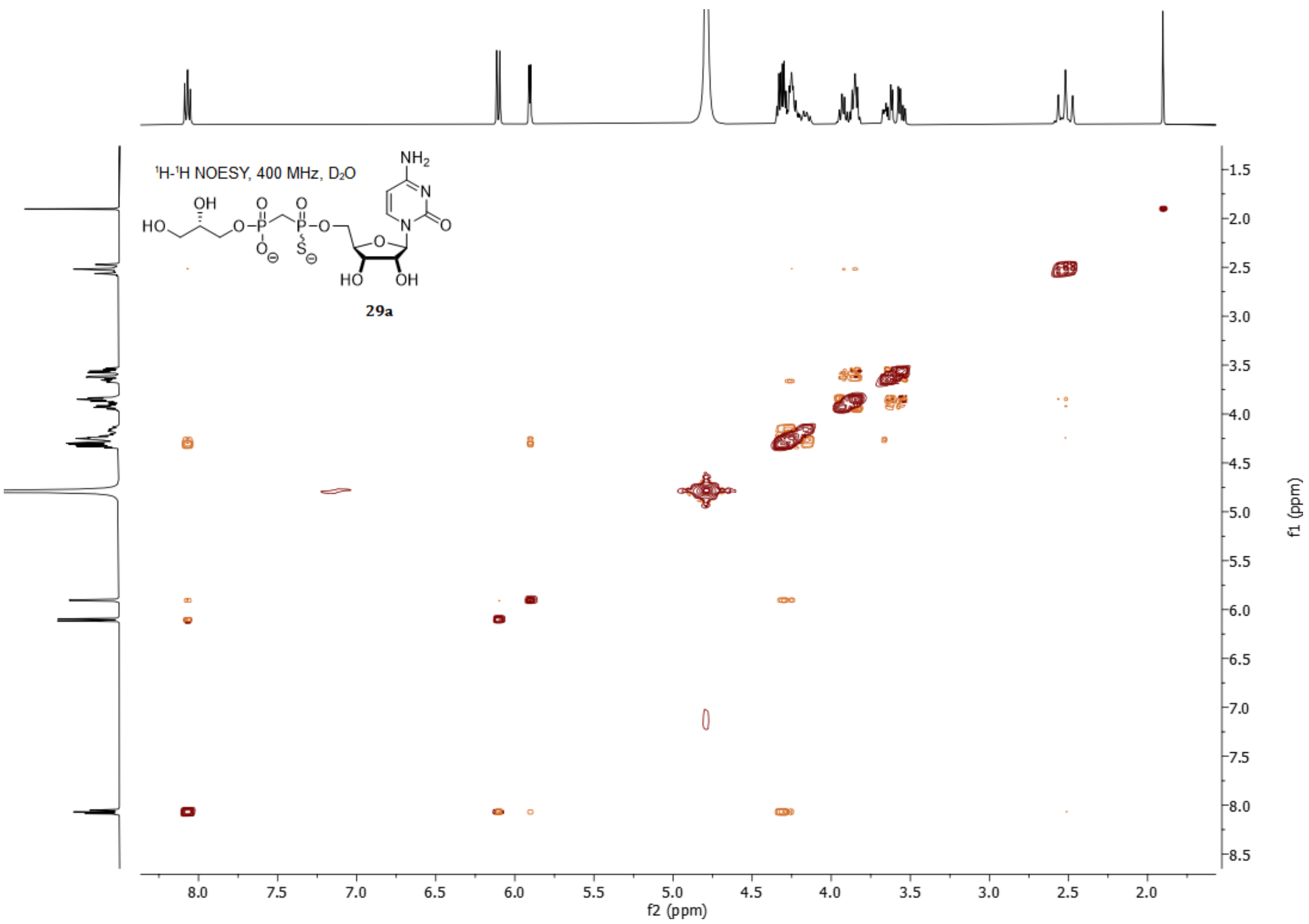

¹H-¹H NOESY, 400 MHz, D₂O
NH₂
OH
HO
O
P
O
S
N
OH
29a
f1 (ppm)
f2 (ppm)
8.0 7.5 7.0 6.5 6.0 5.5 5.0 4.5 4.0 3.5 3.0 2.5 2.0
1.5 2.0 2.5 3.0 3.5 4.0 4.5 5.0 5.5 6.0 6.5 7.0 7.5 8.0 8.5

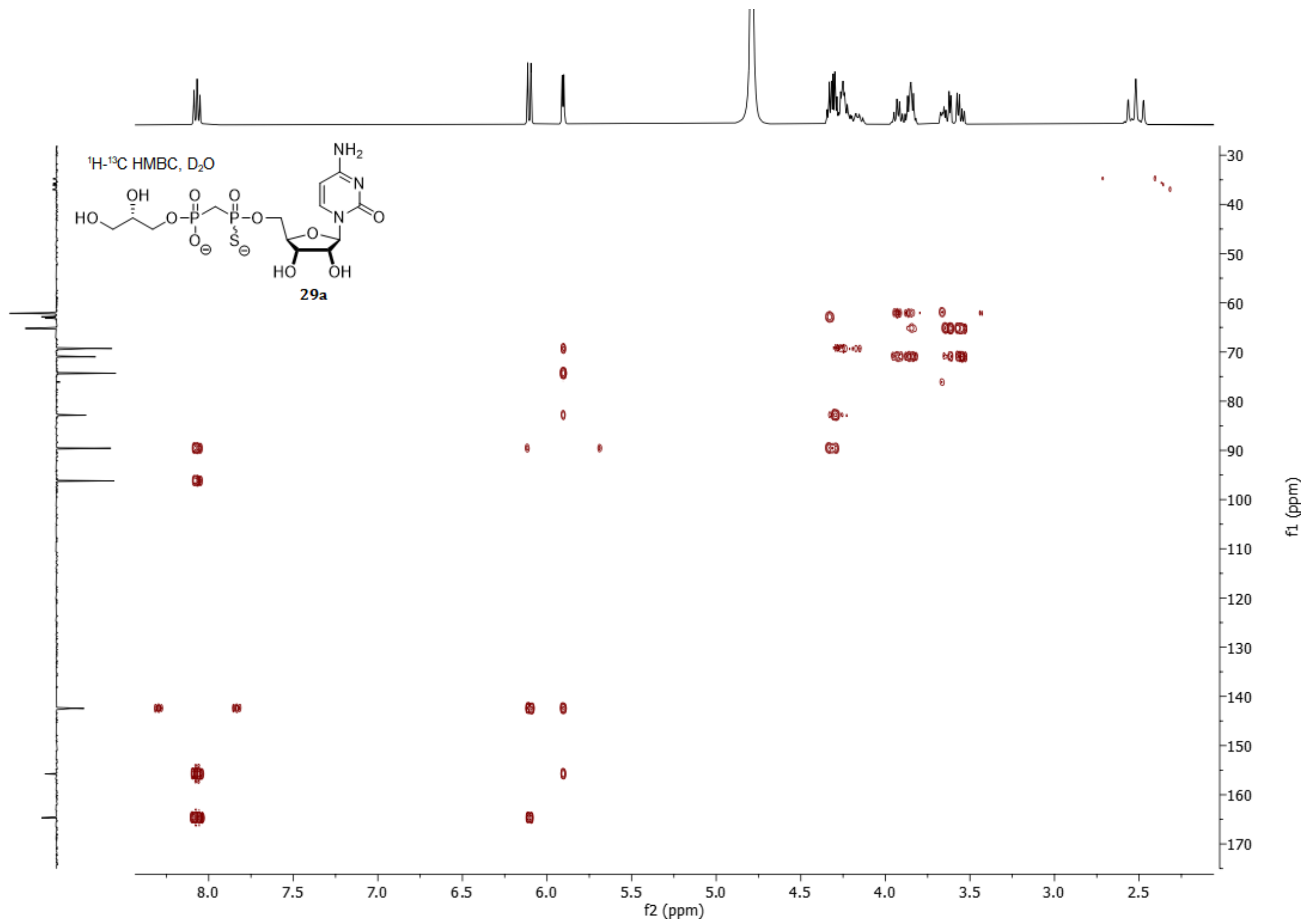

^{1}H-^{13}C HMBC, D$_2$O
29a
f2 (ppm)
f1 (ppm)

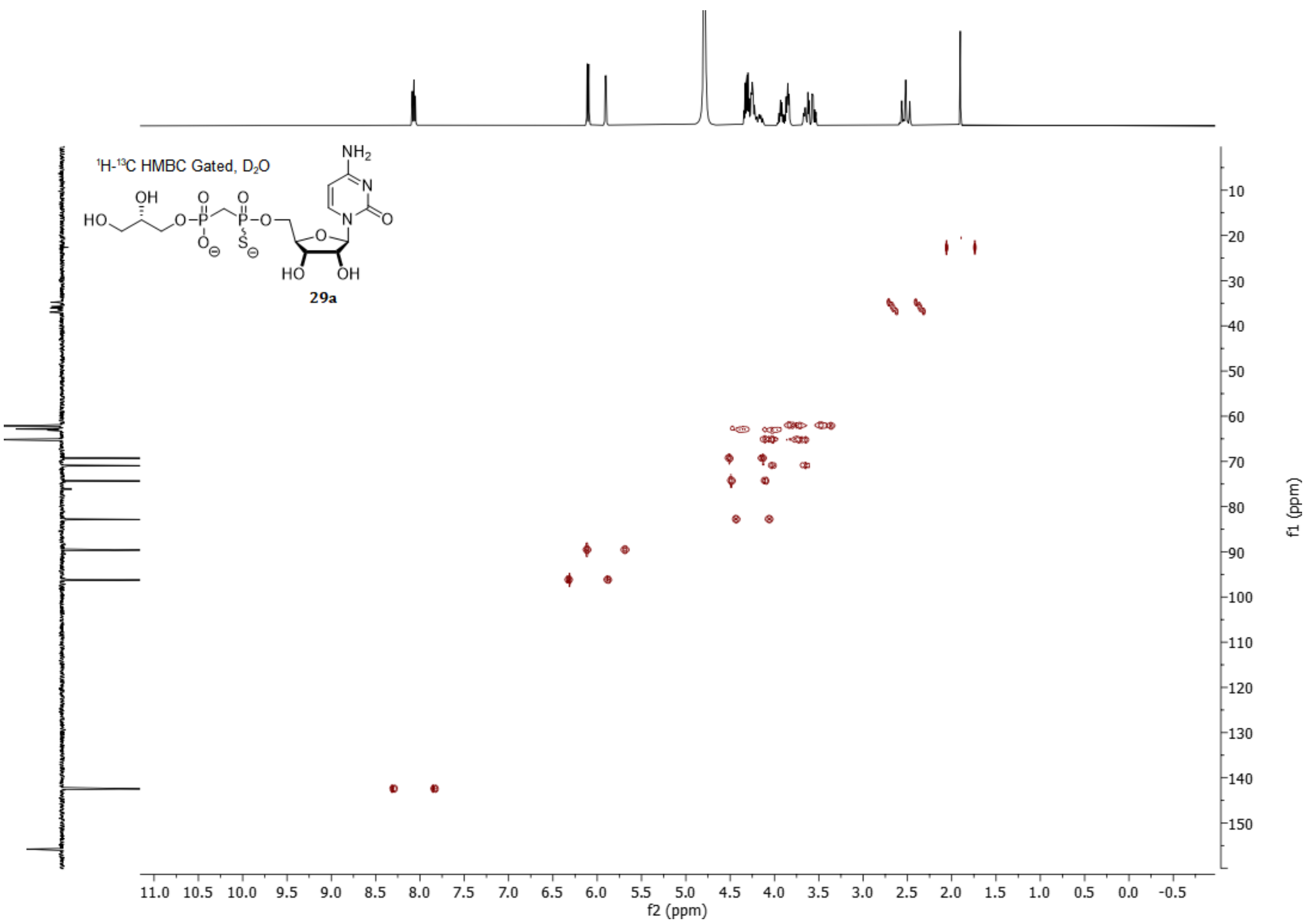

^{1}H-^{13}C HMBC Gated, D$_2$O
29a
f2 (ppm)
f1 (ppm)

***sn*-1 me-CDP-αS$_2$ Gro 29b**

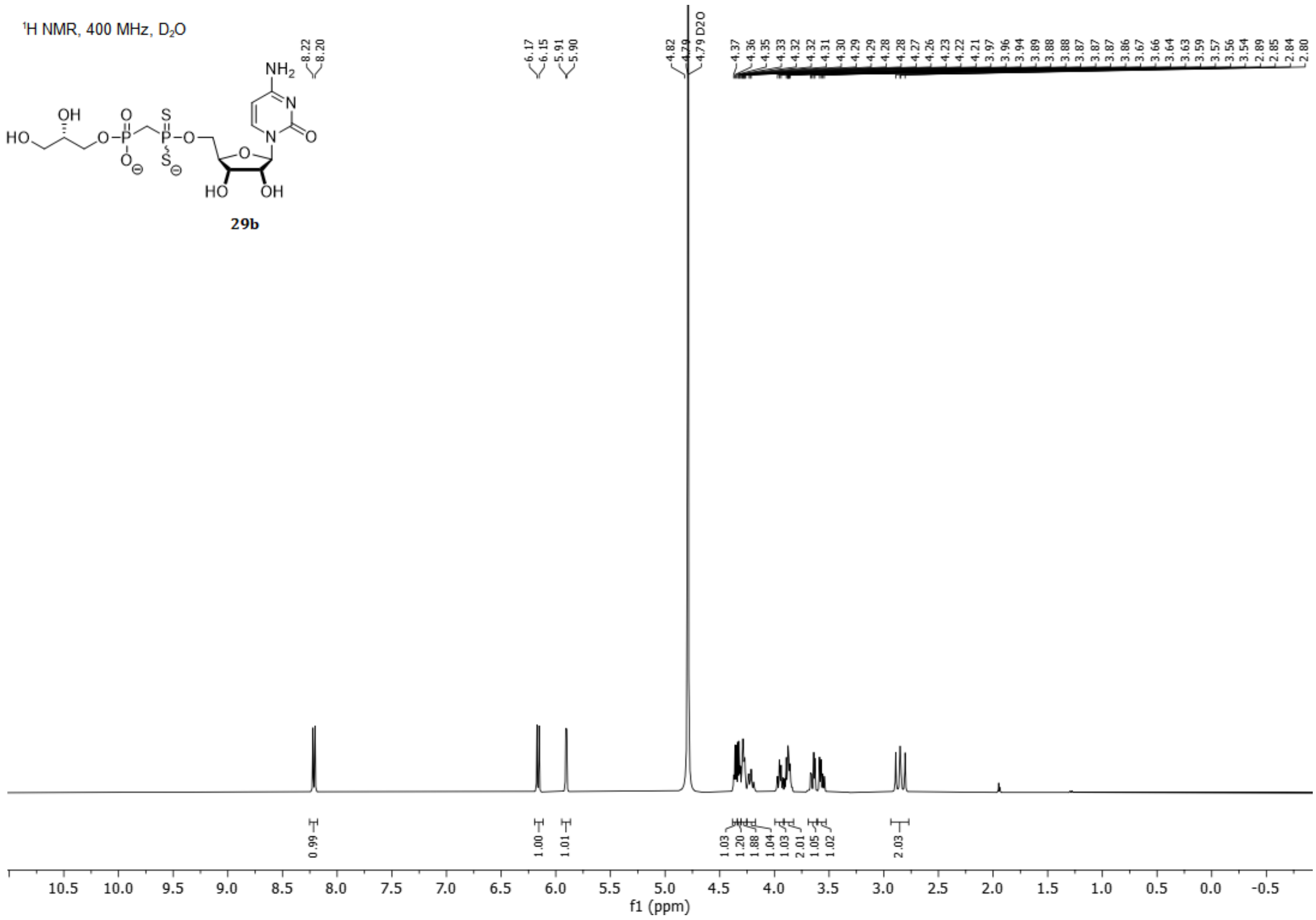

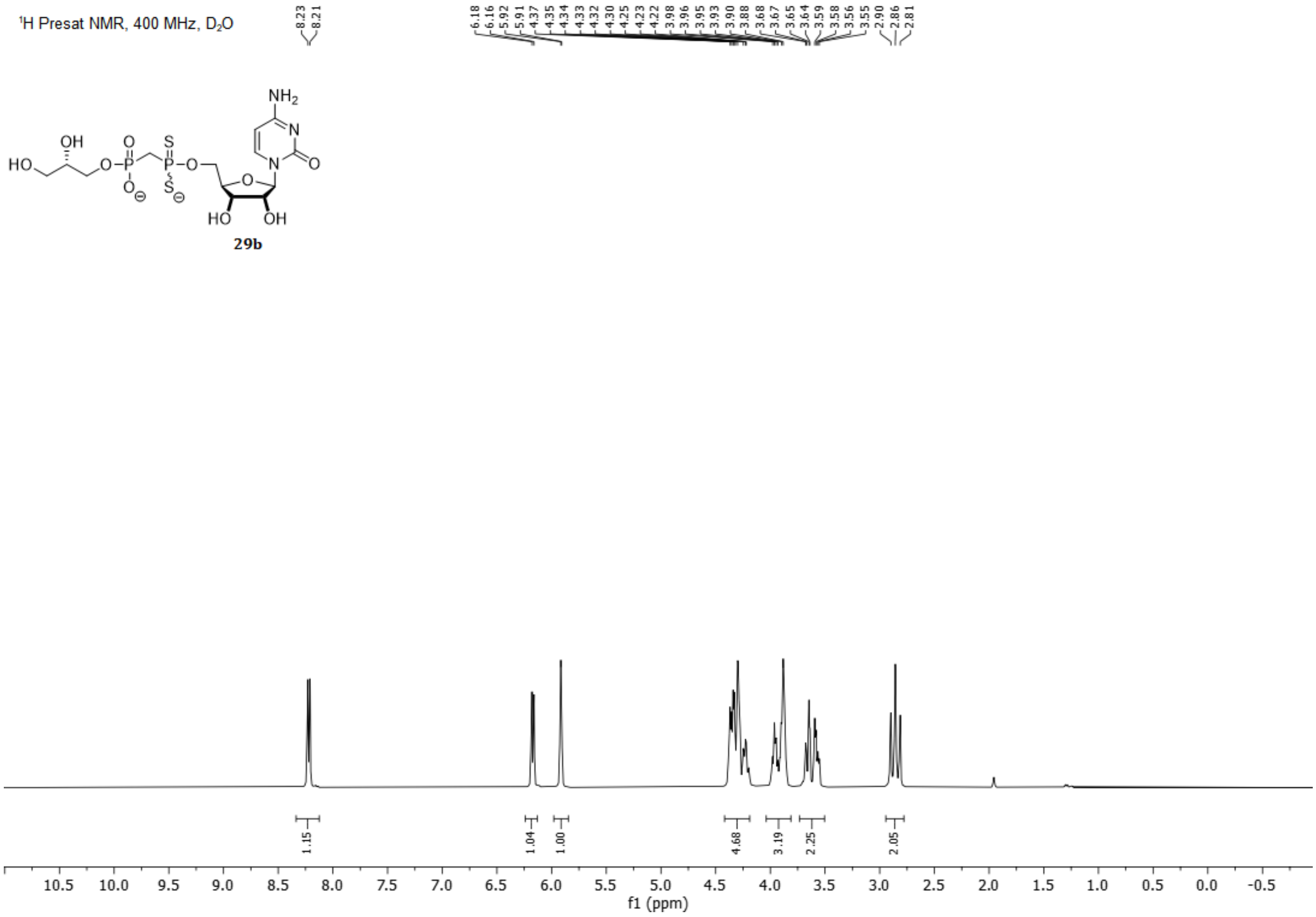

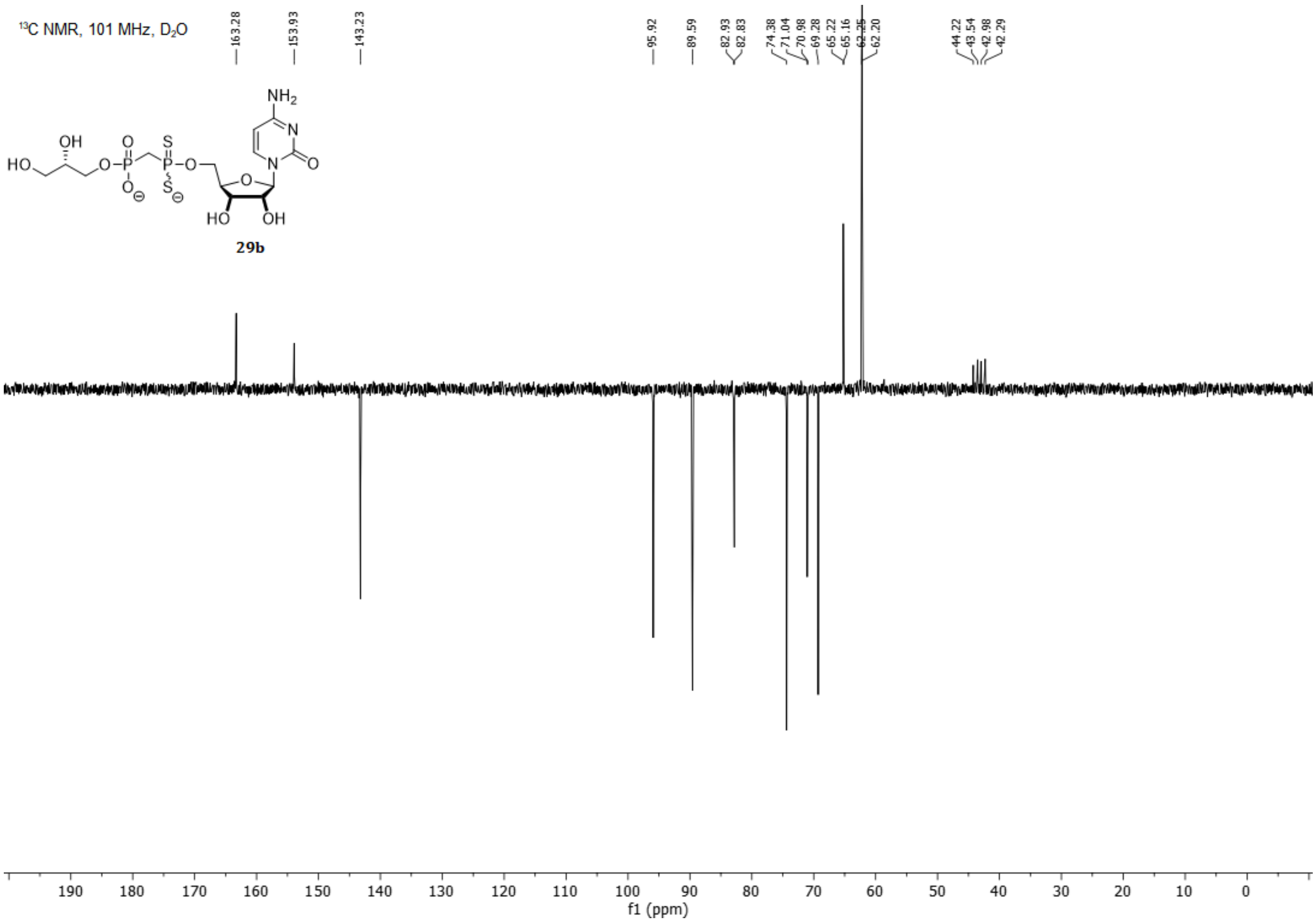

13C NMR, 101 MHz, D2O
163.28
153.93
143.23
95.92
89.59
82.93
82.83
74.38
71.04
70.98
69.28
65.22
65.16
62.35
62.20
44.22
43.54
42.98
42.29
NH2
N
O
OH
HO
O
P
S
HO
OH
29b
190
180
170
160
150
140
130
120
110
100
90
80
70
60
50
40
30
20
10
0
f1 (ppm)

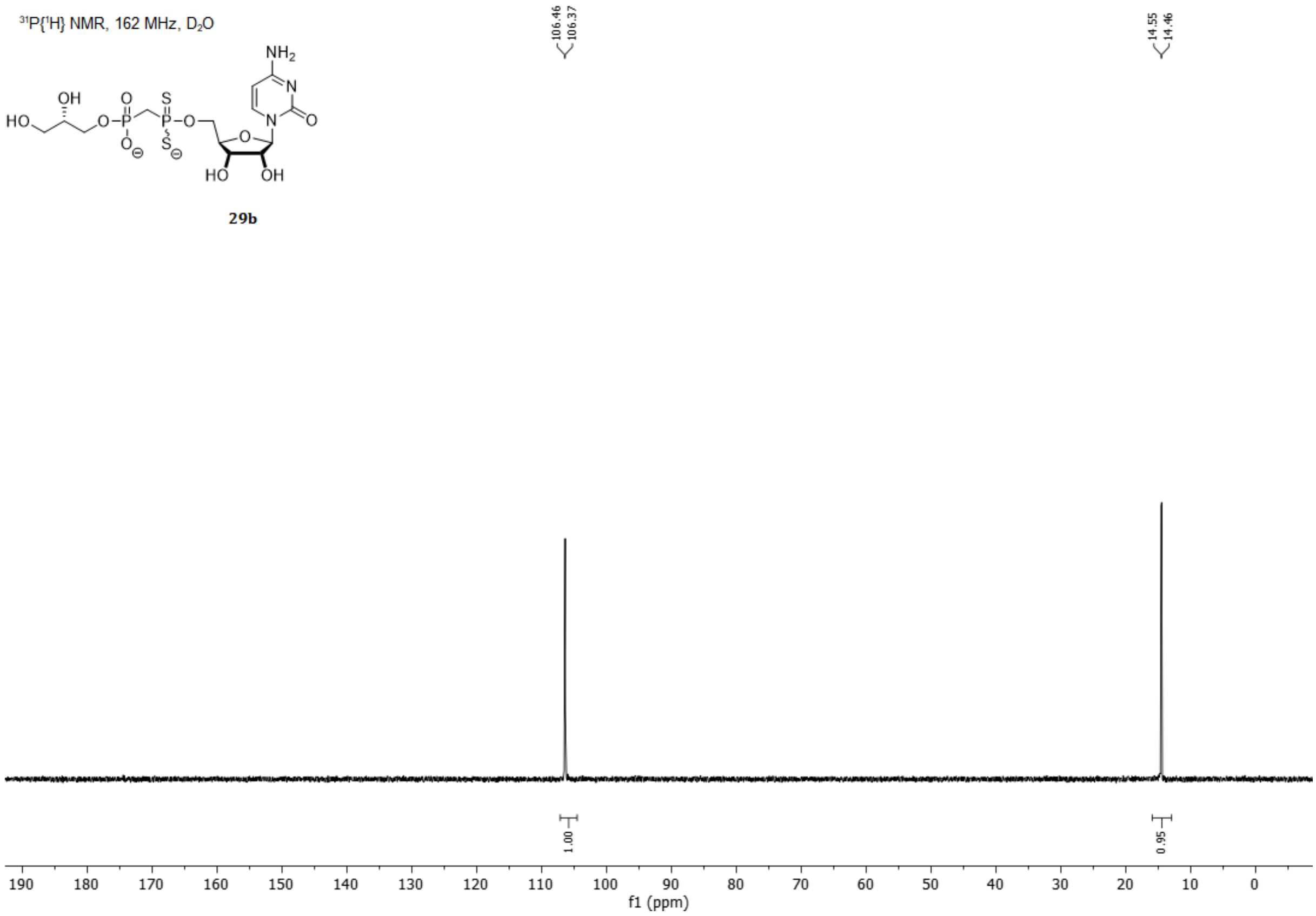

31P{1H} NMR, 162 MHz, D2O
106.46
106.37
14.55
14.46
NH2
N
O
OH
HO
O
P
S
HO
OH
29b
1.00
0.95
190
180
170
160
150
140
130
120
110
100
90
80
70
60
50
40
30
20
10
0
f1 (ppm)

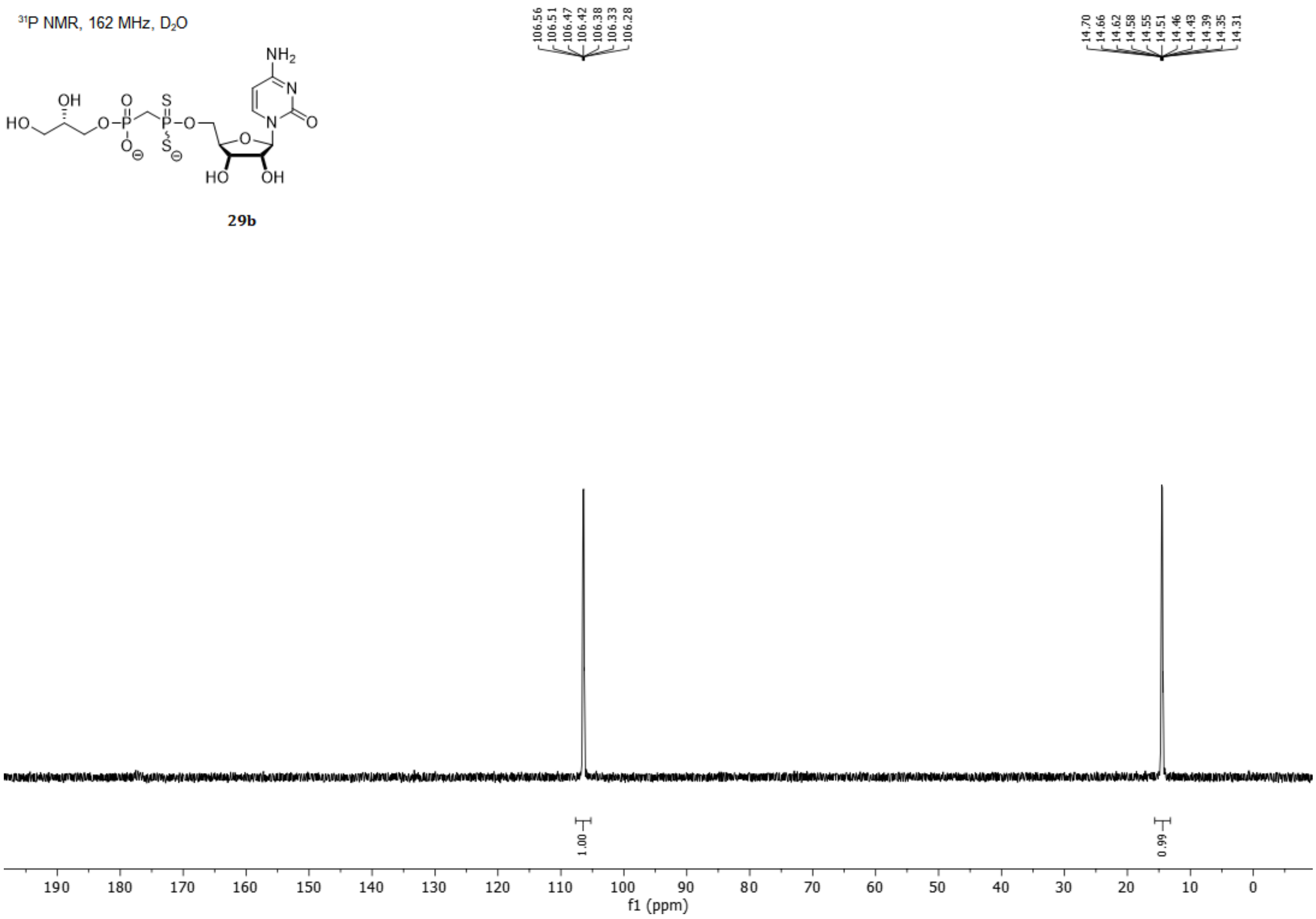

31P NMR, 162 MHz, D2O
29b
f1 (ppm)

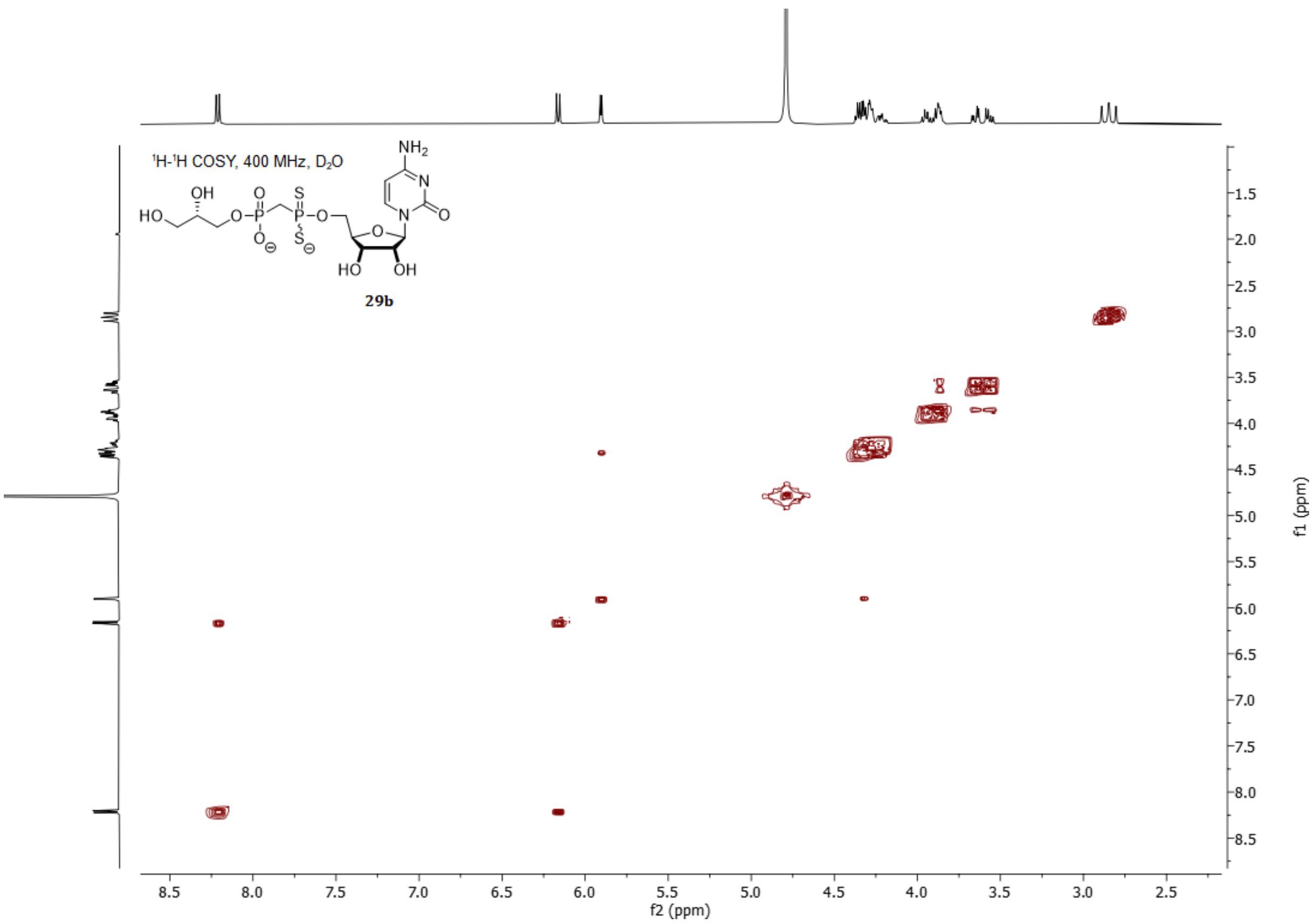

1H-1H COSY, 400 MHz, D2O
29b
f2 (ppm)
f1 (ppm)

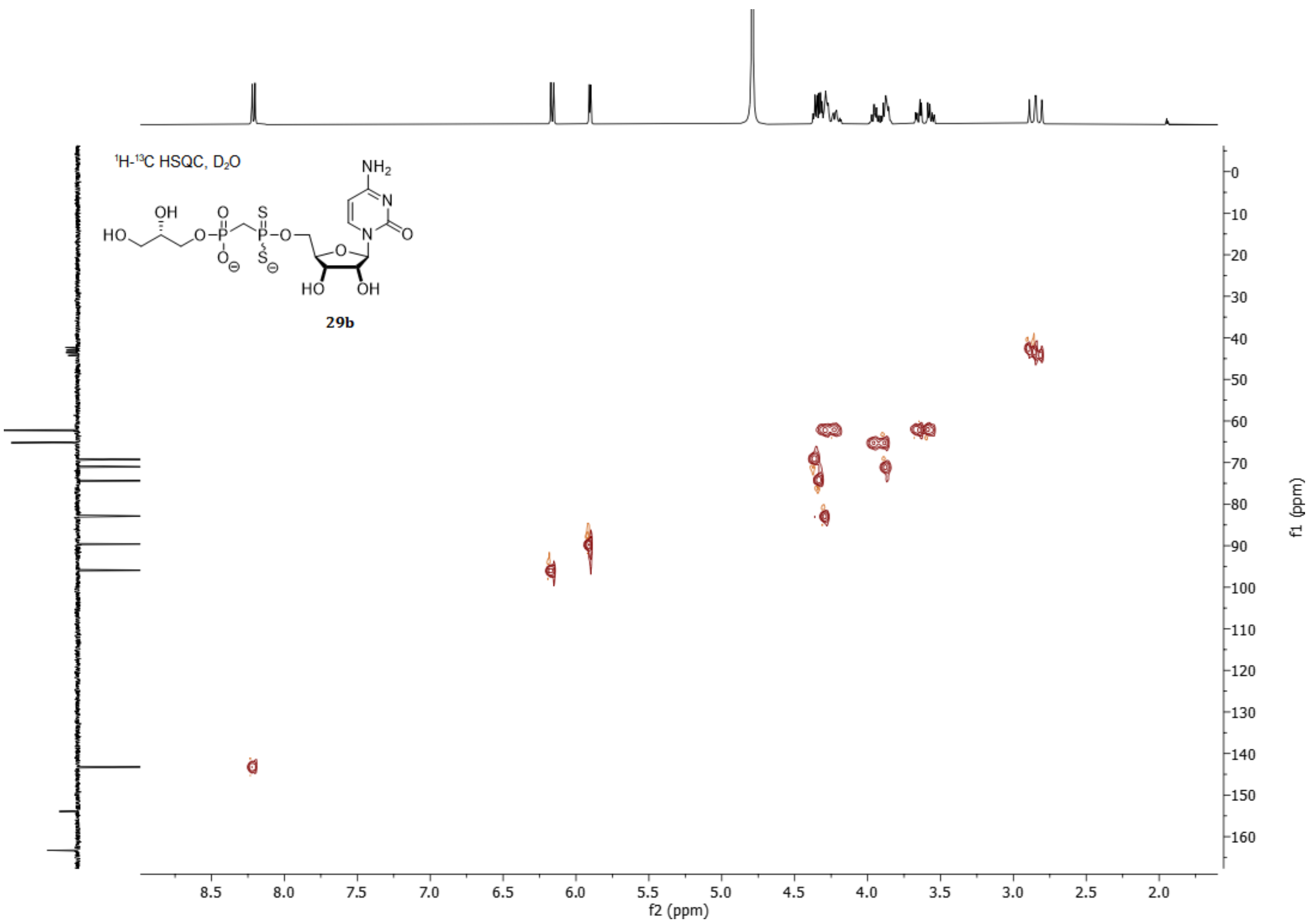

^1H-^{13}C HSQC, D_2O
29b
f2 (ppm)
f1 (ppm)

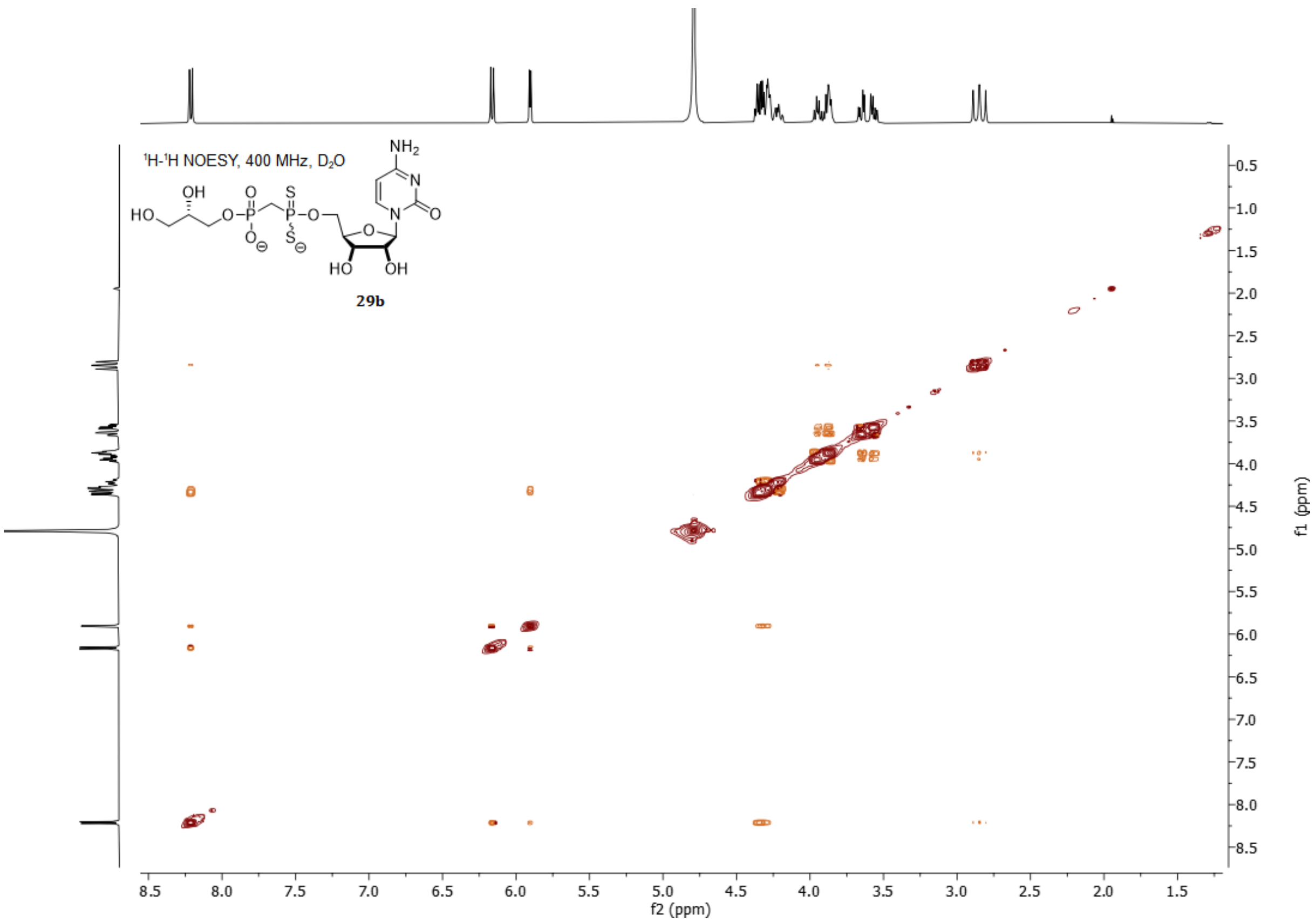

^1H-^1H NOESY, 400 MHz, D_2O
29b
f2 (ppm)
f1 (ppm)

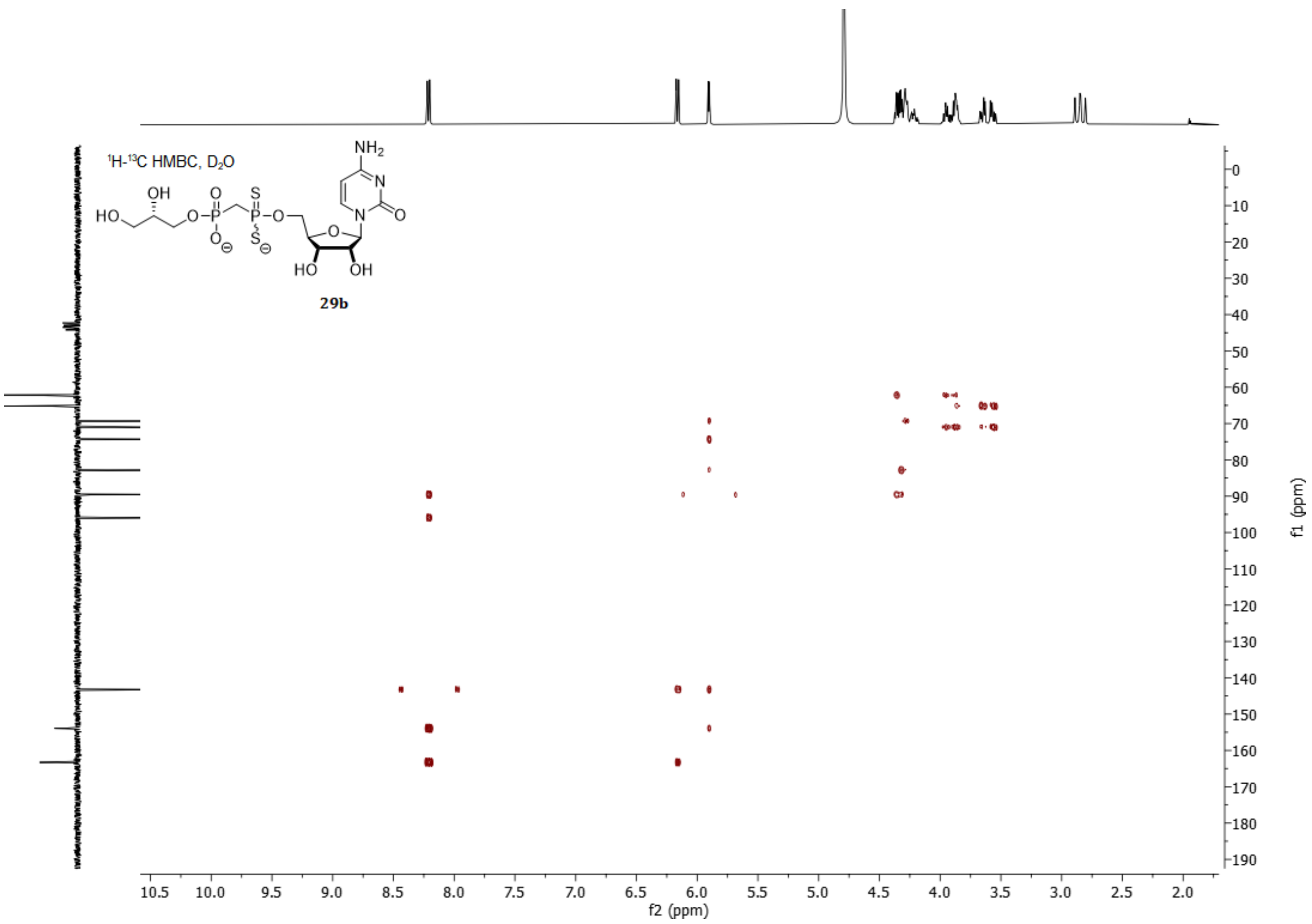

me-CDP-αS-ribitol 30a

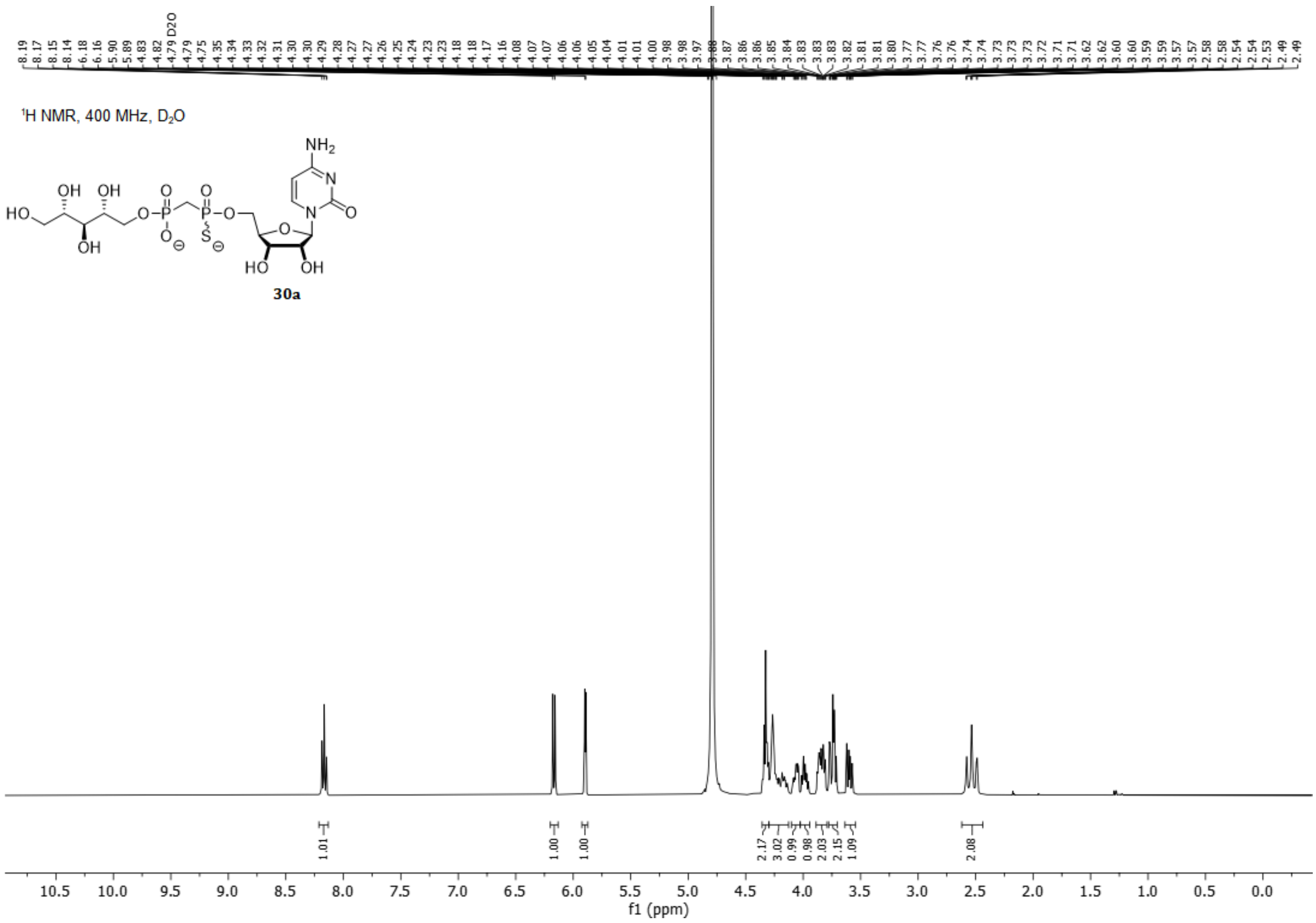

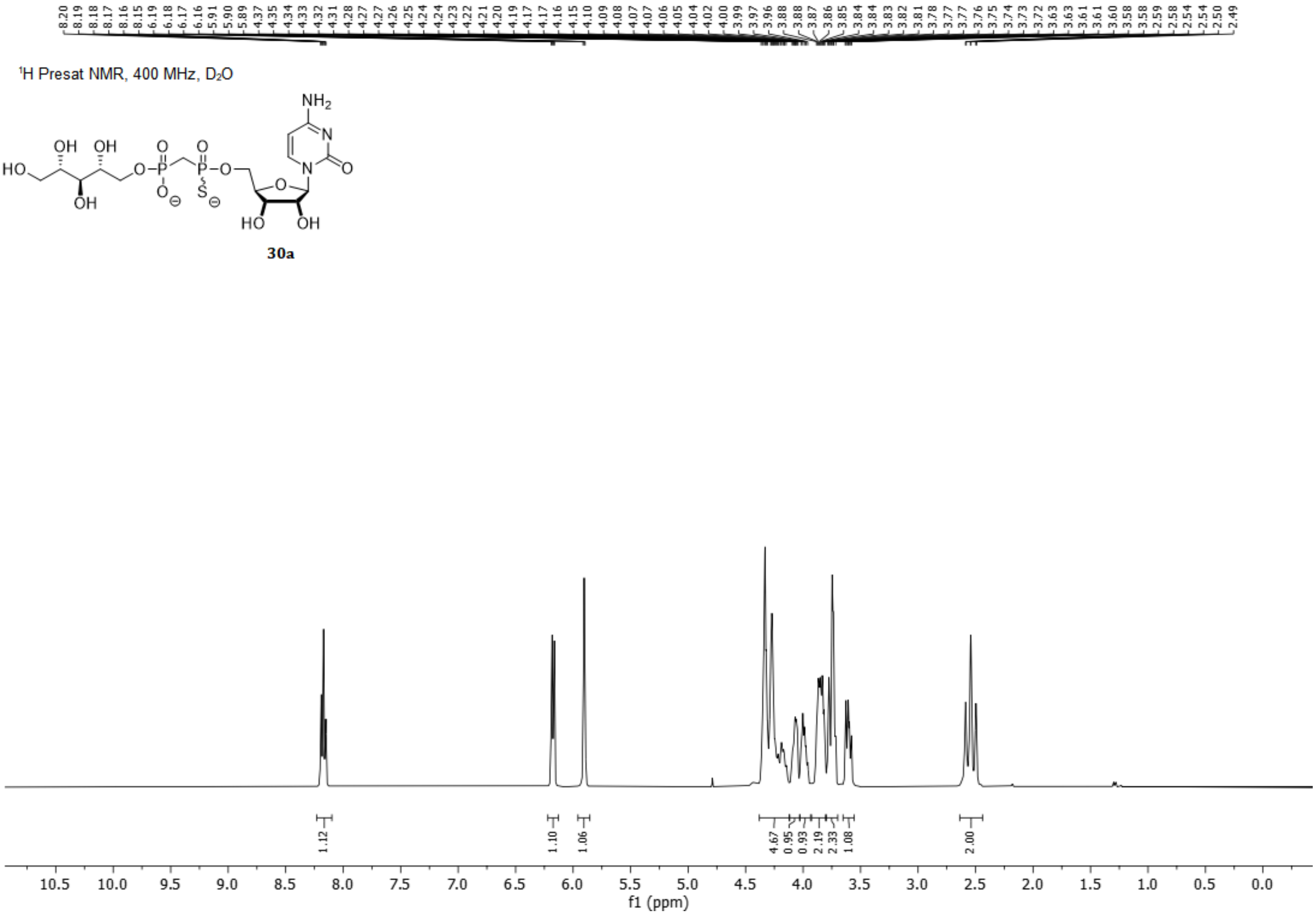
8.20 8.19 8.18 8.17 8.16 8.15 6.19 6.18 6.17 6.16 5.91 5.90 5.89 4.37 4.35 4.34 4.33 4.32 4.31 4.28 4.27 4.27 4.26 4.25 4.24 4.24 4.23 4.22 4.21 4.20 4.19 4.17 4.17 4.16 4.15 4.10 4.09 4.08 4.07 4.07 4.06 4.05 4.04 4.02 4.00 3.99 3.97 3.96 3.88 3.88 3.87 3.86 3.85 3.84 3.84 3.83 3.82 3.81 3.78 3.77 3.77 3.76 3.75 3.74 3.73 3.72 3.63 3.63 3.61 3.60 3.58 3.58 2.59 2.58 2.54 2.50 2.49
¹H Presat NMR, 400 MHz, D₂O
30a
1.12
1.10
1.06
4.67 0.95 0.93 2.19 2.33 1.08
2.00
10.5 10.0 9.5 9.0 8.5 8.0 7.5 7.0 6.5 6.0 5.5 5.0 4.5 4.0 3.5 3.0 2.5 2.0 1.5 1.0 0.5 0.0
f1 (ppm)

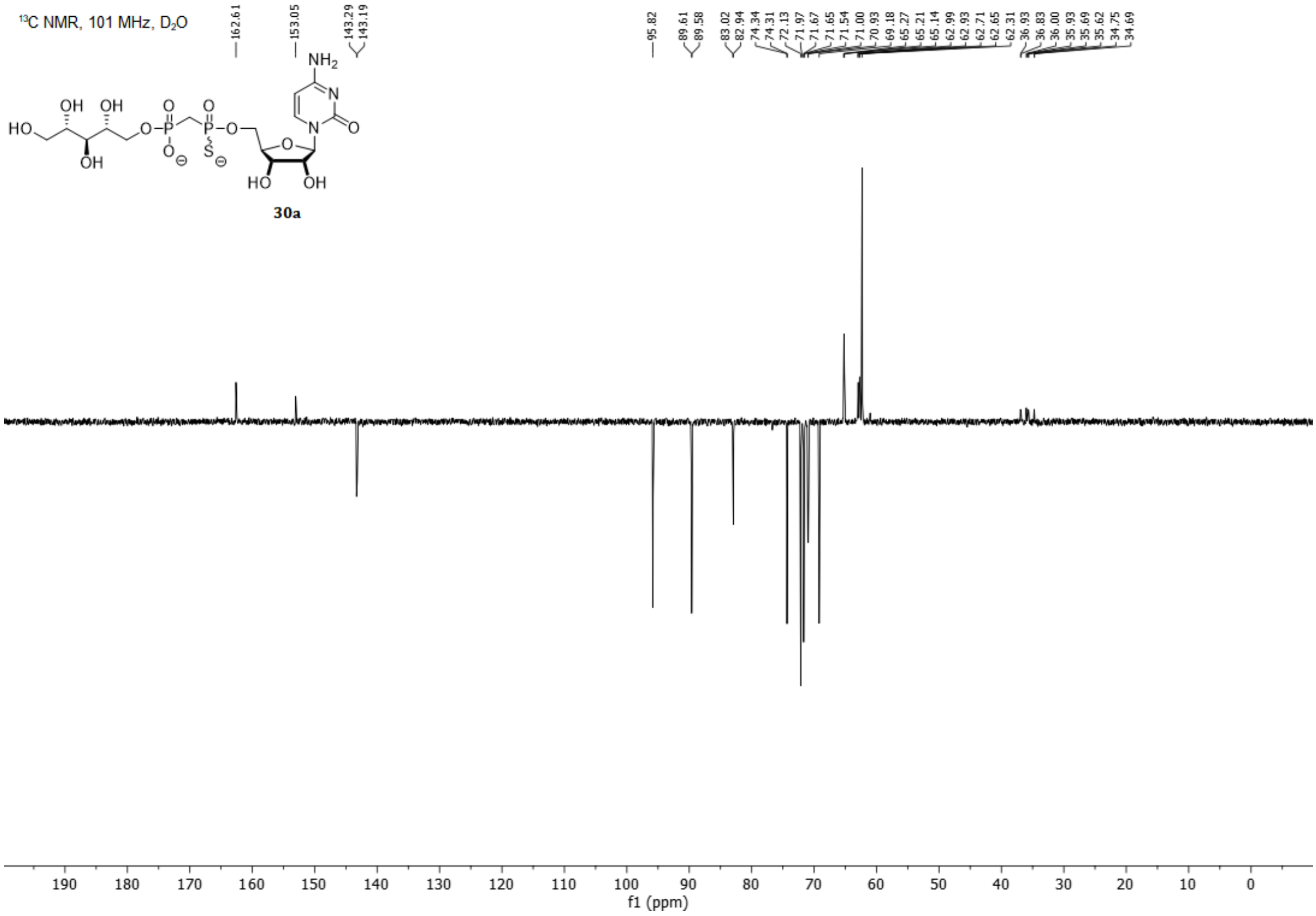
¹³C NMR, 101 MHz, D₂O
162.61
153.05
143.29 143.19
95.82
89.61 89.58
83.02 82.94
74.34 74.31 72.13 71.97 71.67 71.65 71.54 71.00 70.93 69.18 65.27 65.21 65.14 62.99 62.93 62.71 62.65 62.31
36.93 36.83 36.00 35.93 35.69 35.62 34.75 34.69
30a
190 180 170 160 150 140 130 120 110 100 90 80 70 60 50 40 30 20 10 0
f1 (ppm)

$^{31}P\{^1H\}$ NMR, 162 MHz, D_2O

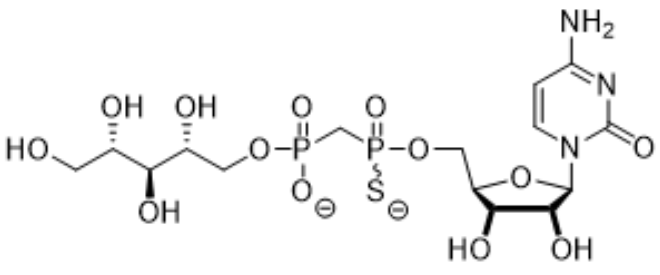

30a

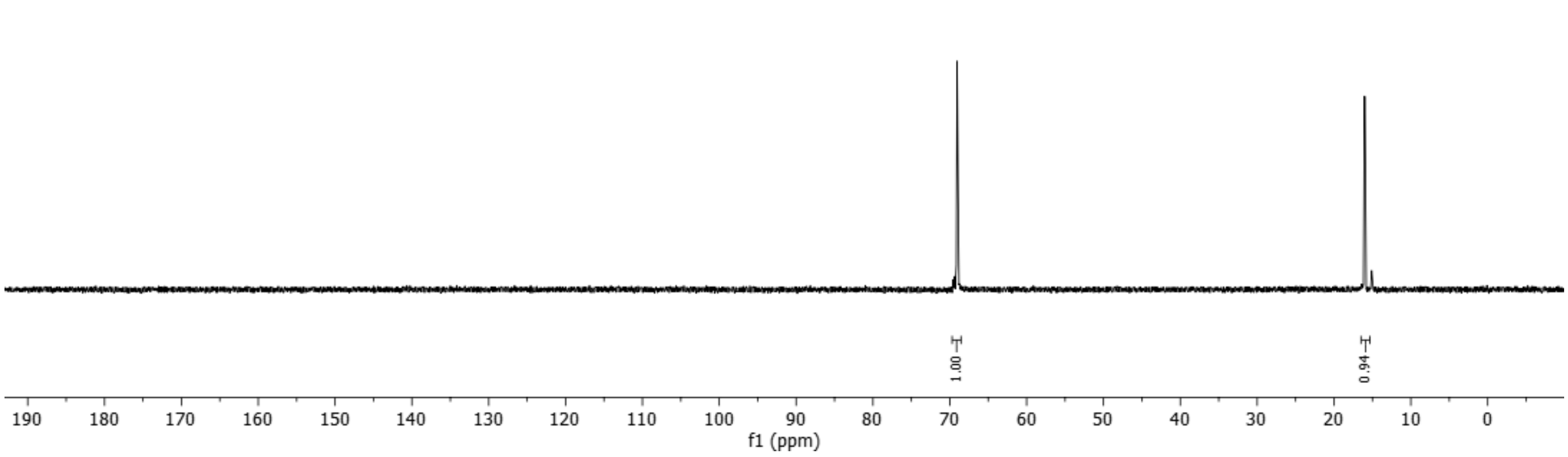

^{31}P NMR, 162 MHz, D_2O

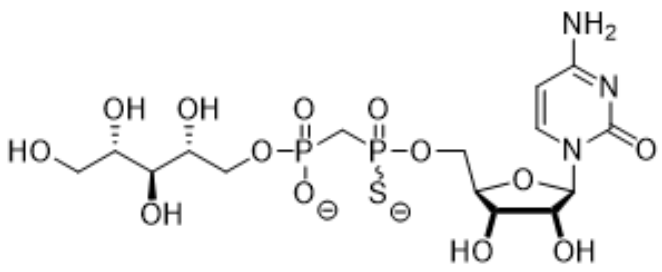

30a

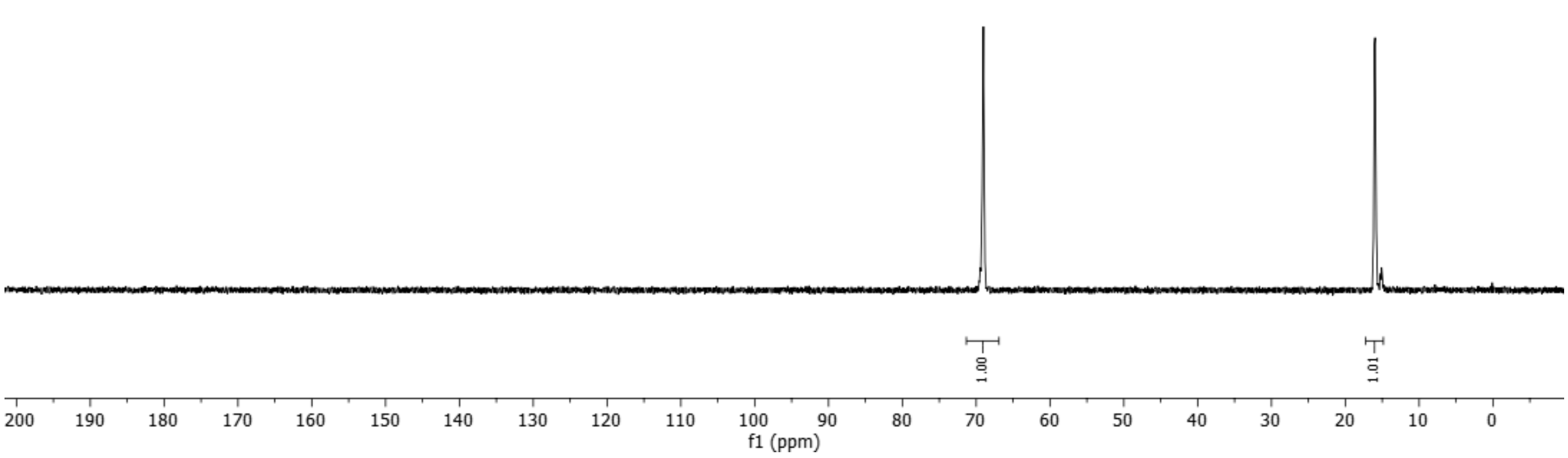

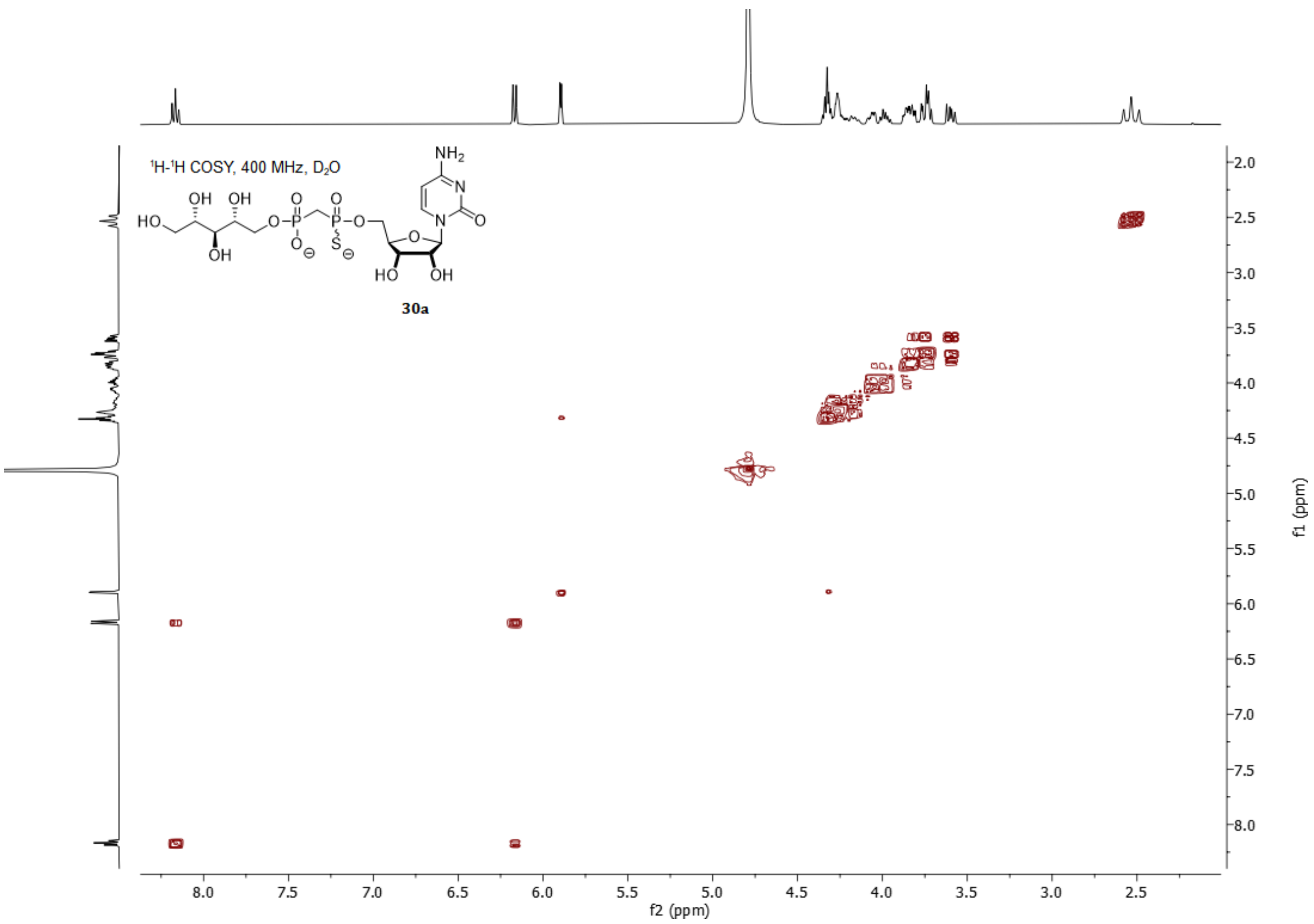
1H-1H COSY, 400 MHz, D2O
30a
f2 (ppm)
f1 (ppm)

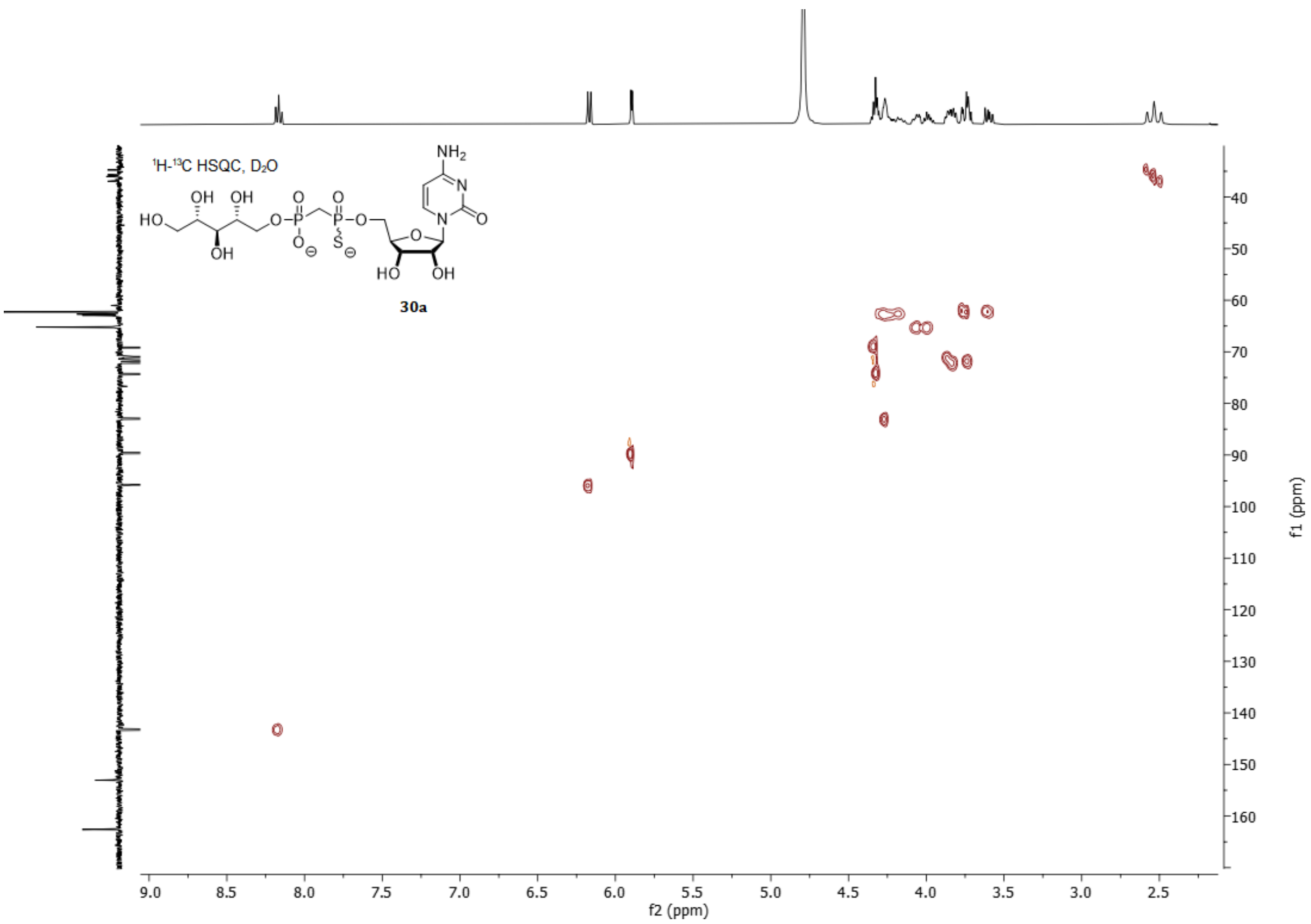
1H-13C HSQC, D2O
30a
f2 (ppm)
f1 (ppm)

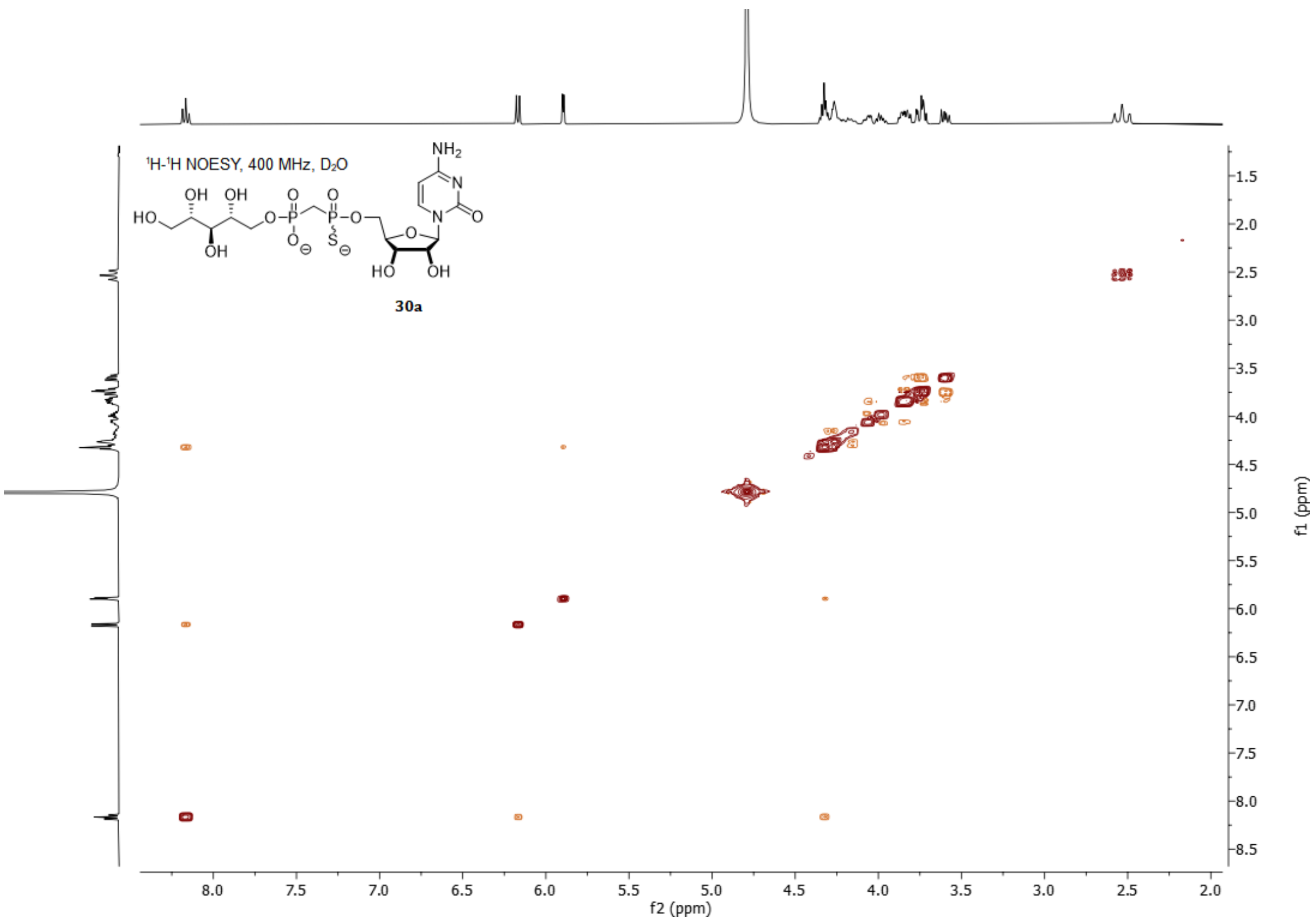

¹H-¹H NOESY, 400 MHz, D₂O
30a
f2 (ppm)
f1 (ppm)

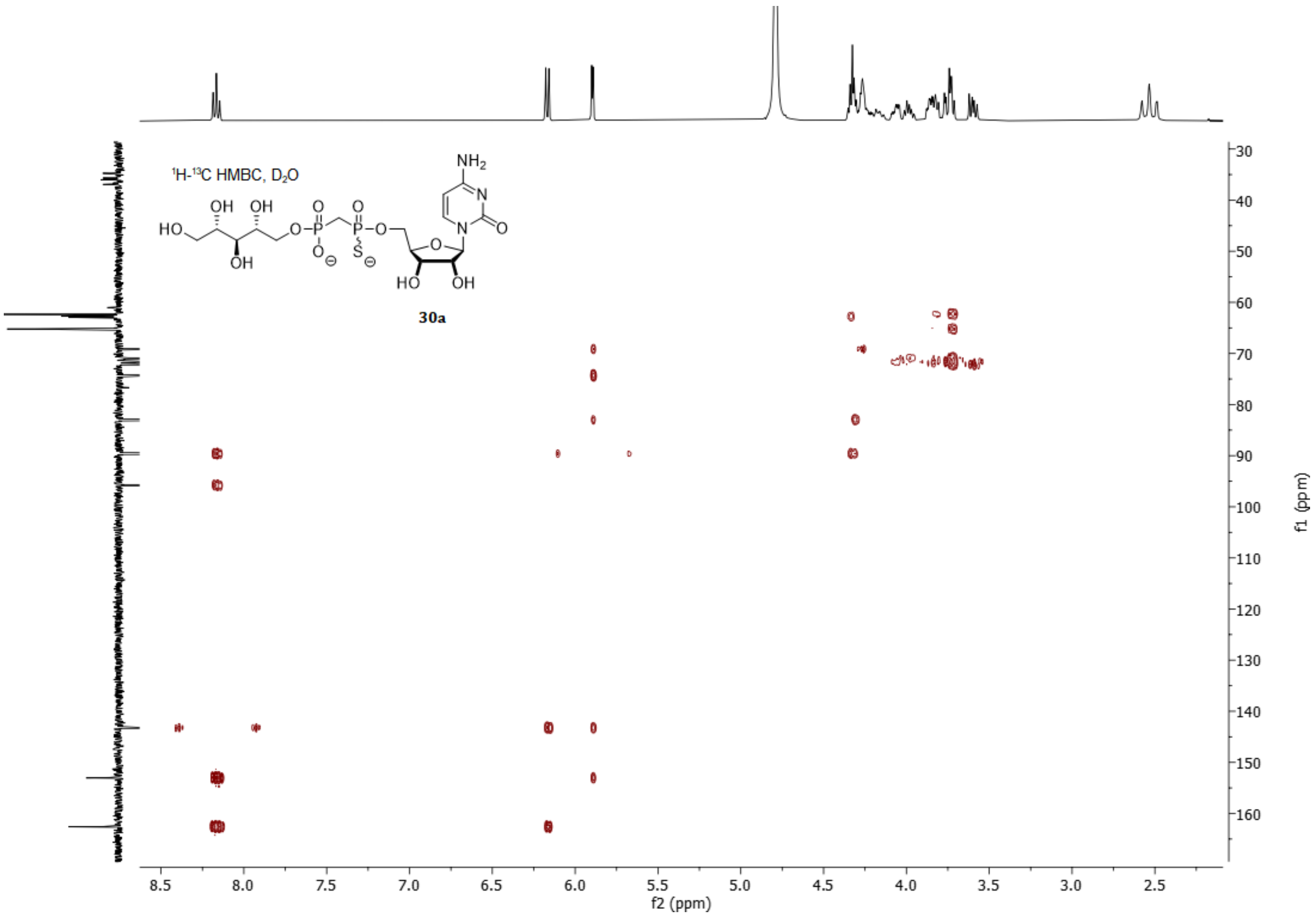

¹H-¹³C HMBC, D₂O
30a
f2 (ppm)
f1 (ppm)

me-CDP-αS₂ ribitol 30b

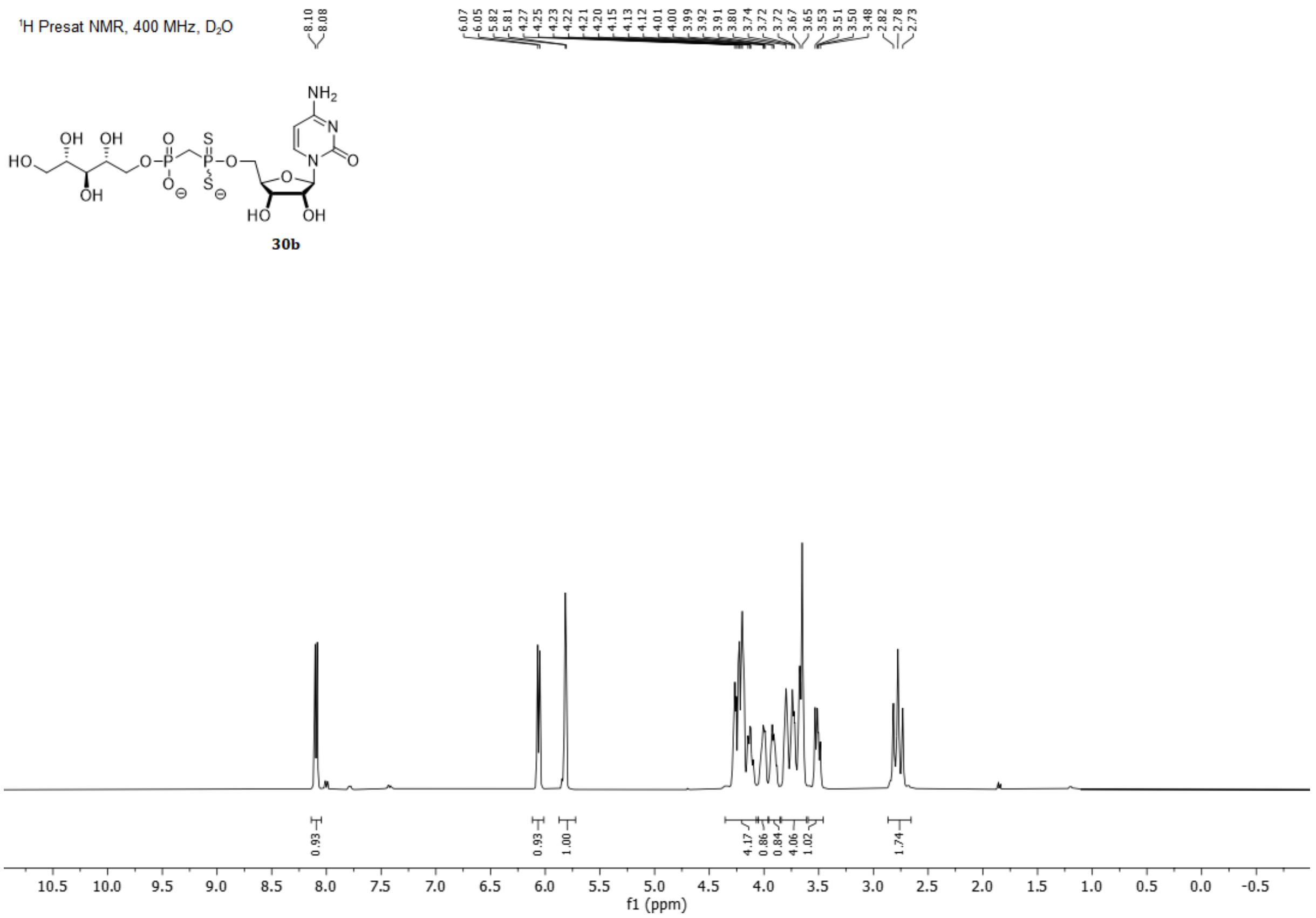

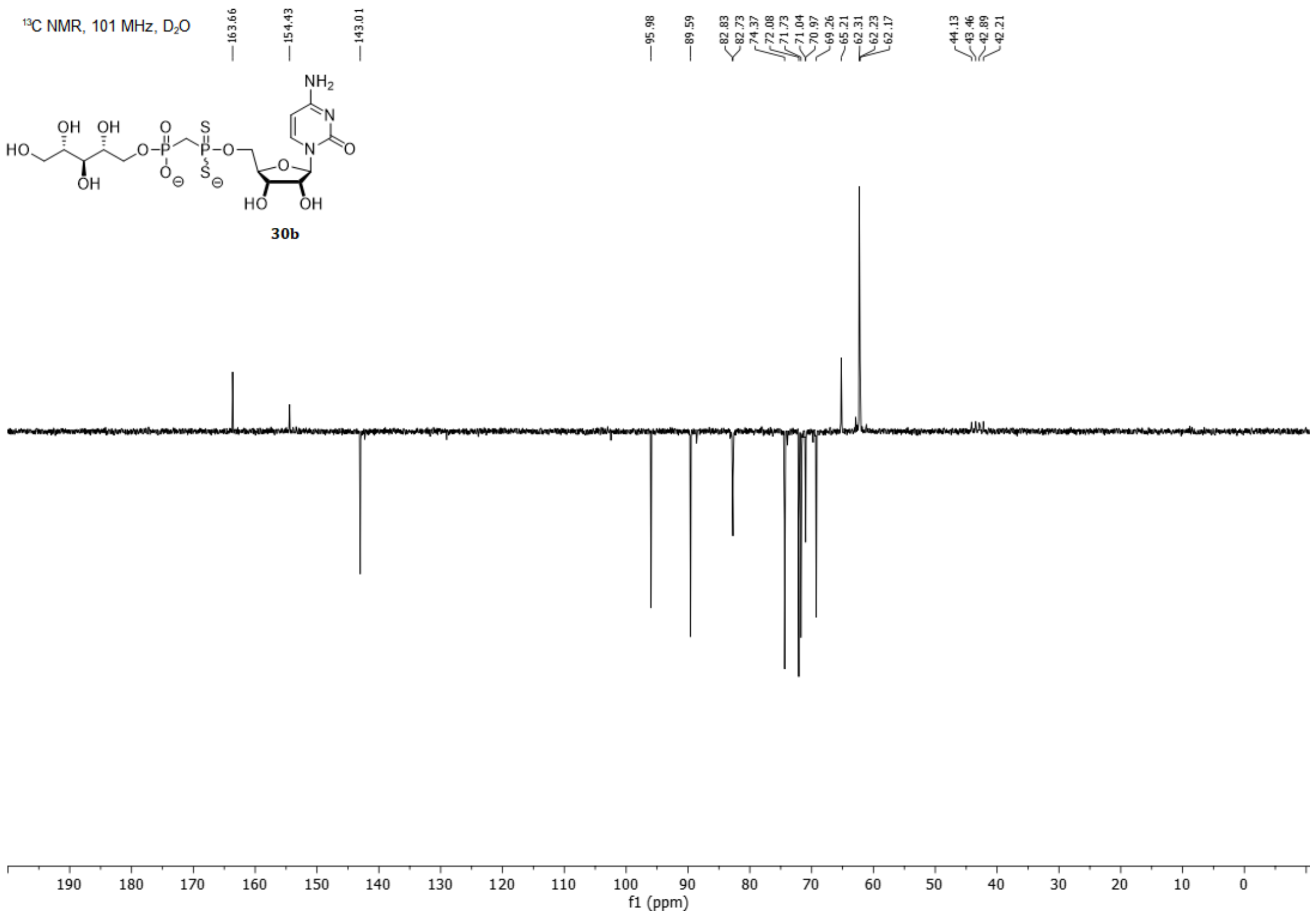

$^{31}P\{^1H\}$ NMR, 162 MHz, D_2O

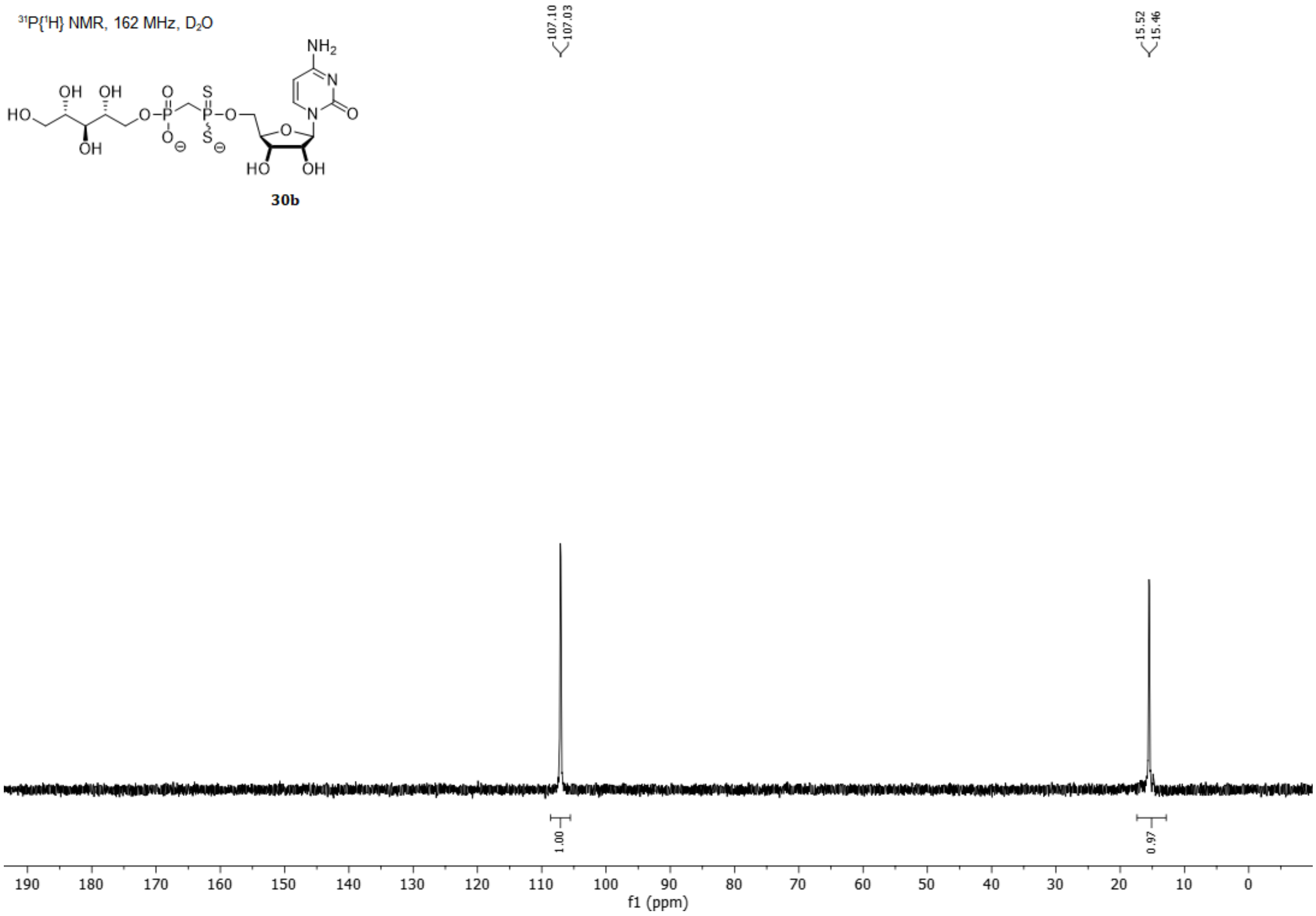

^{31}P NMR, 162 MHz, D_2O

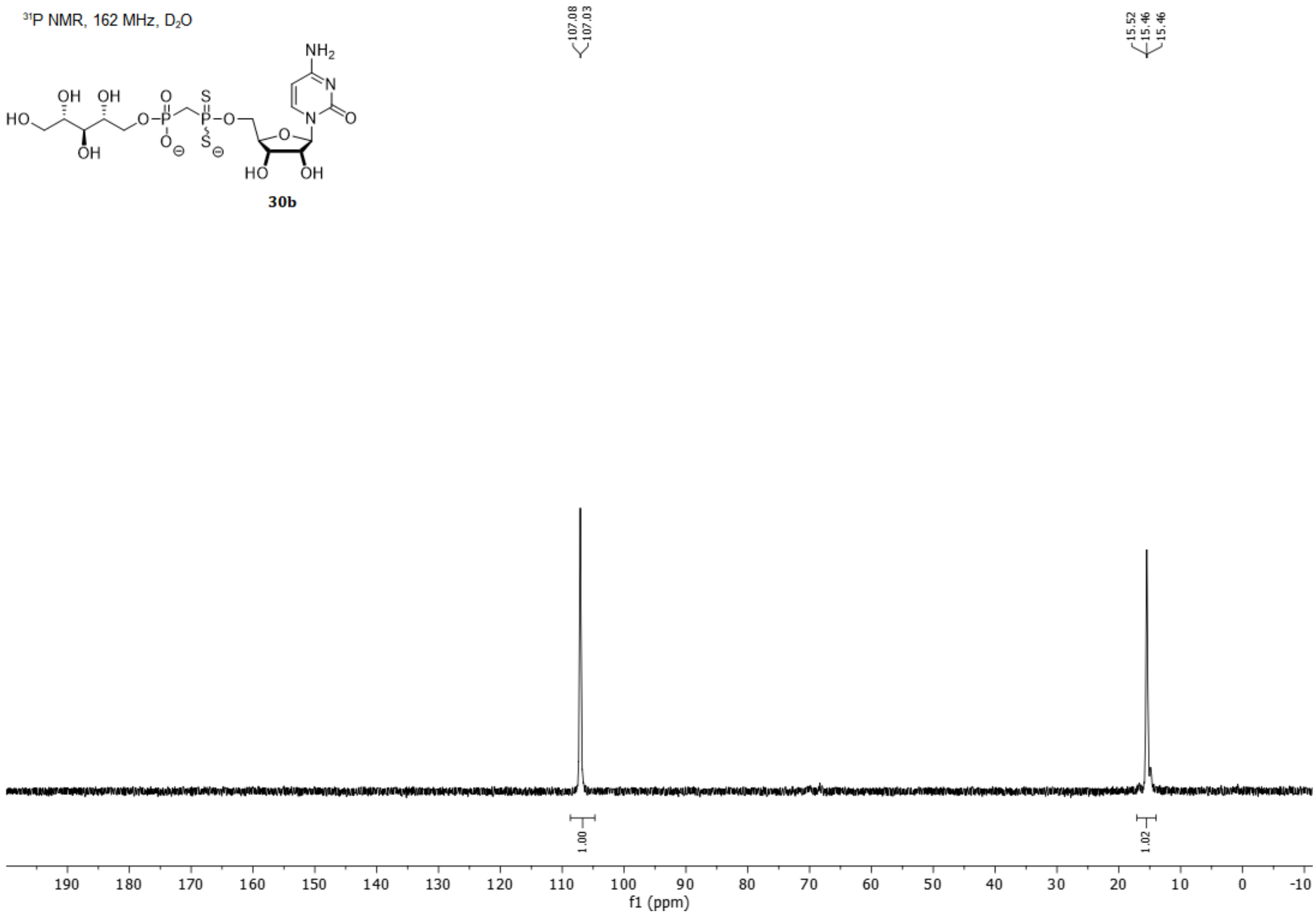

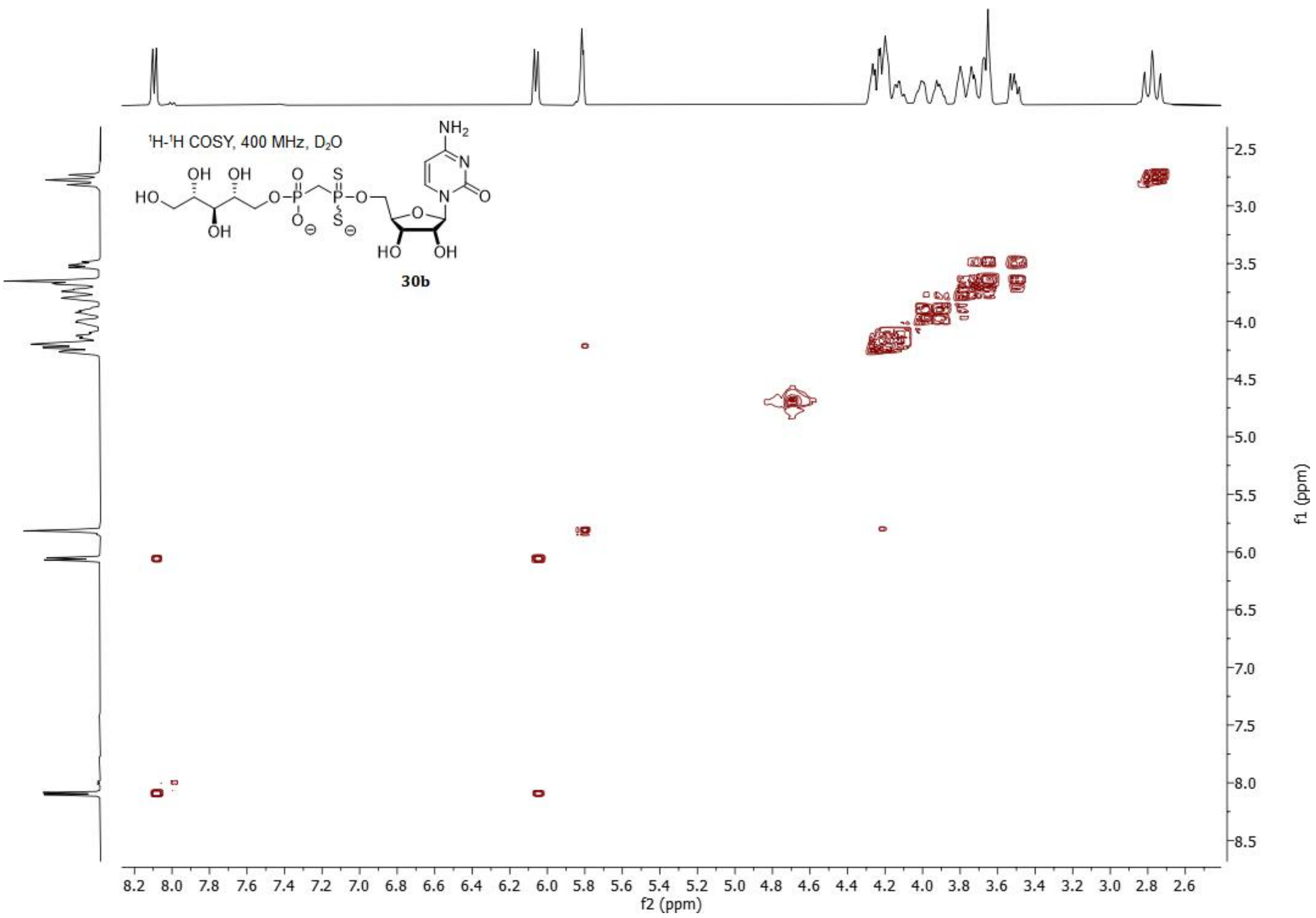
1H-1H COSY, 400 MHz, D2O
30b
f2 (ppm)
f1 (ppm)

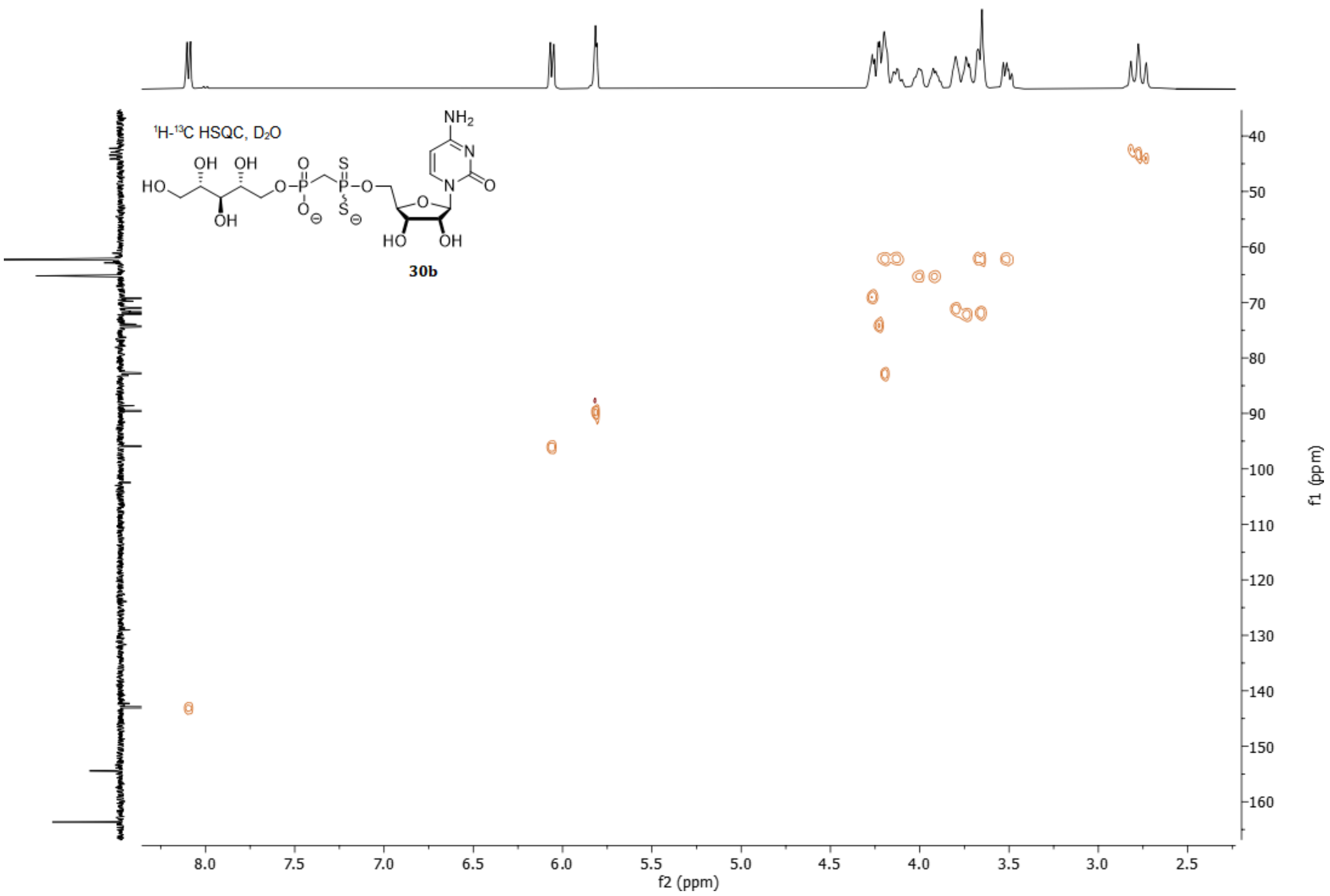
1H-13C HSQC, D2O
30b
f2 (ppm)
f1 (ppm)

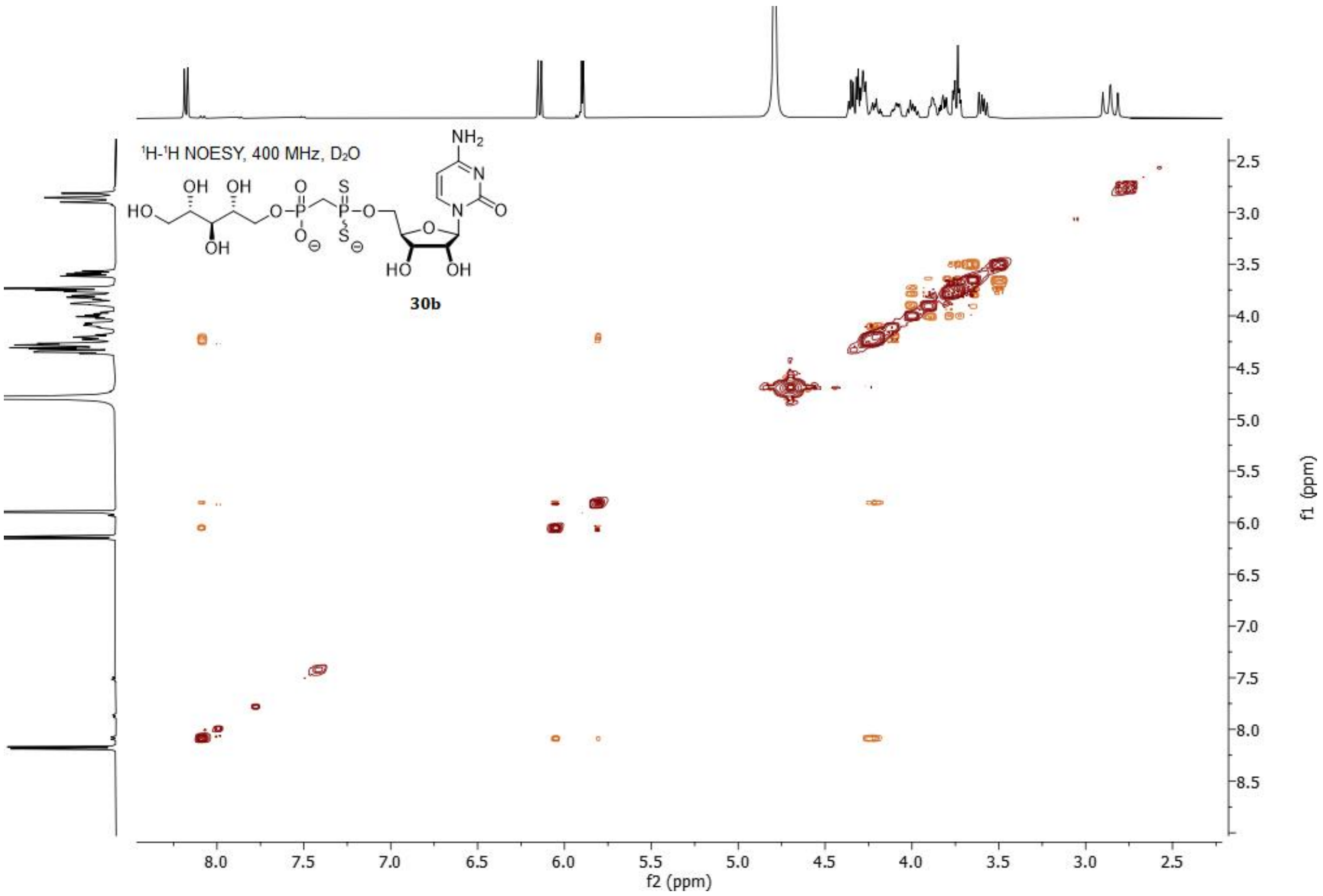

¹H-¹H NOESY, 400 MHz, D₂O
30b
f1 (ppm)
f2 (ppm)

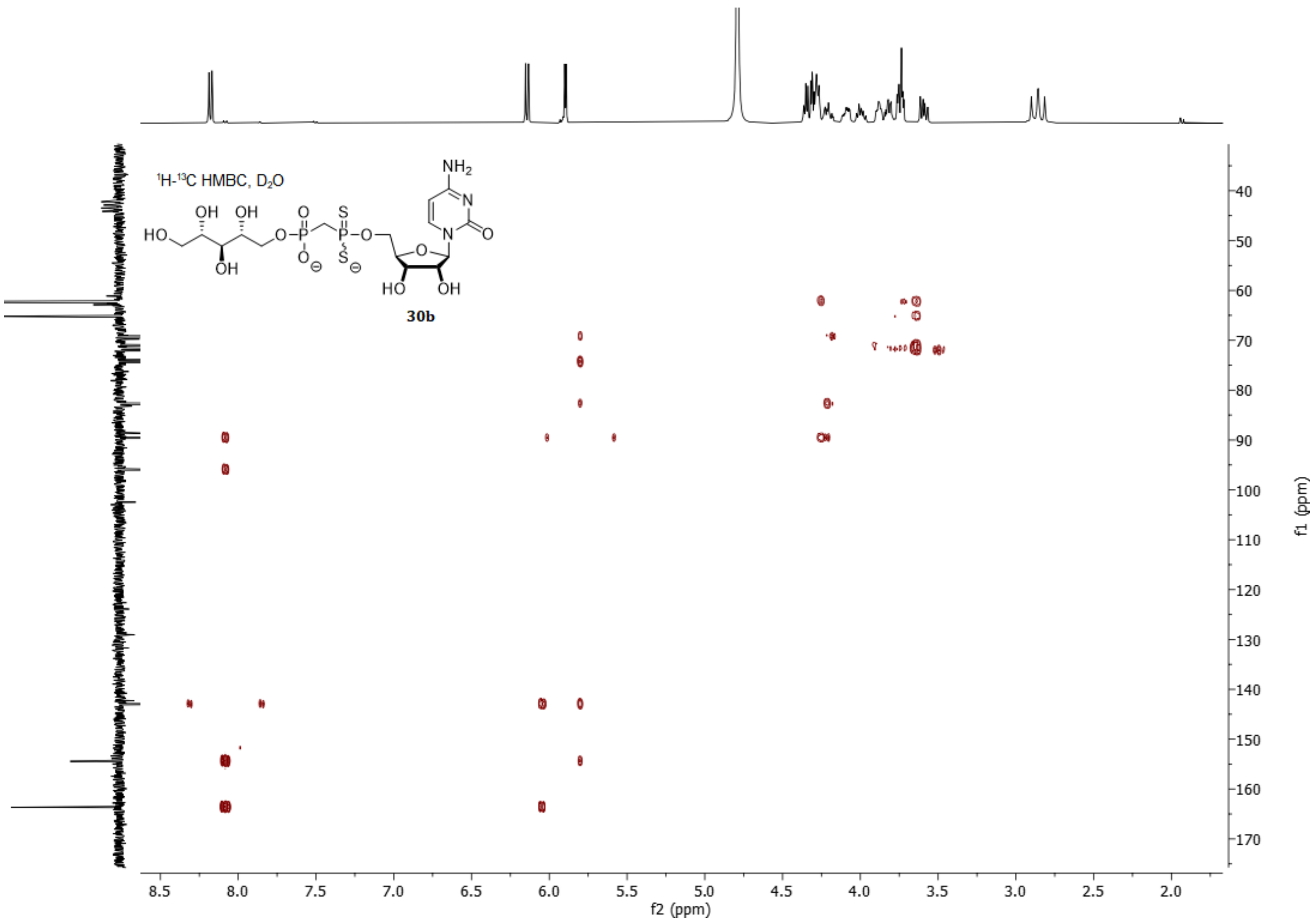

¹H-¹³C HMBC, D₂O
30b
f1 (ppm)
f2 (ppm)

NMR Spectra of Non-Symmetric Phosphonomethyl β-Phosphono(di)thioate Diesters and Precursors

***Tetra*-acetyl- D-ribitol phosphonomethyl phosphonothioate tetraester 31a**

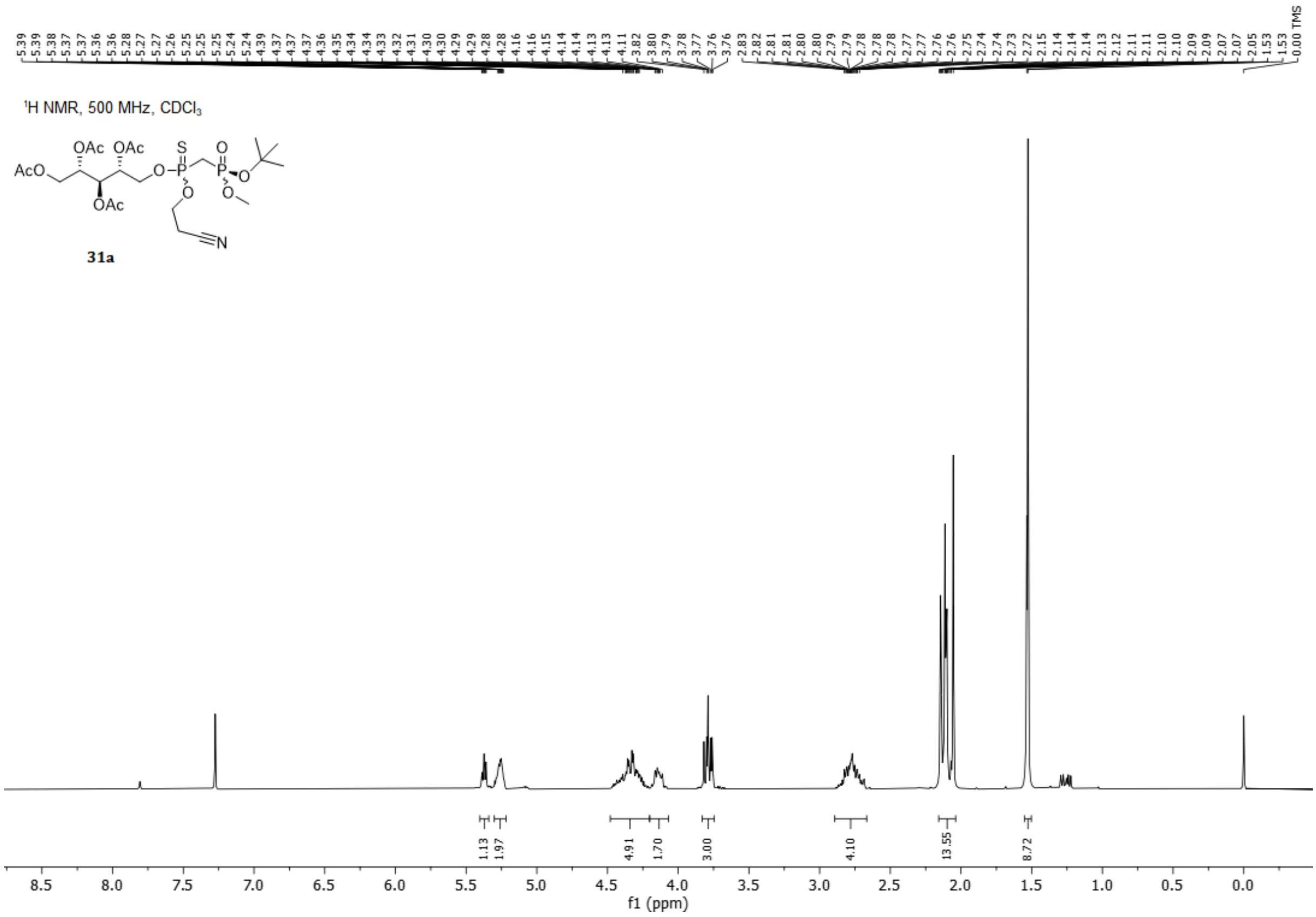

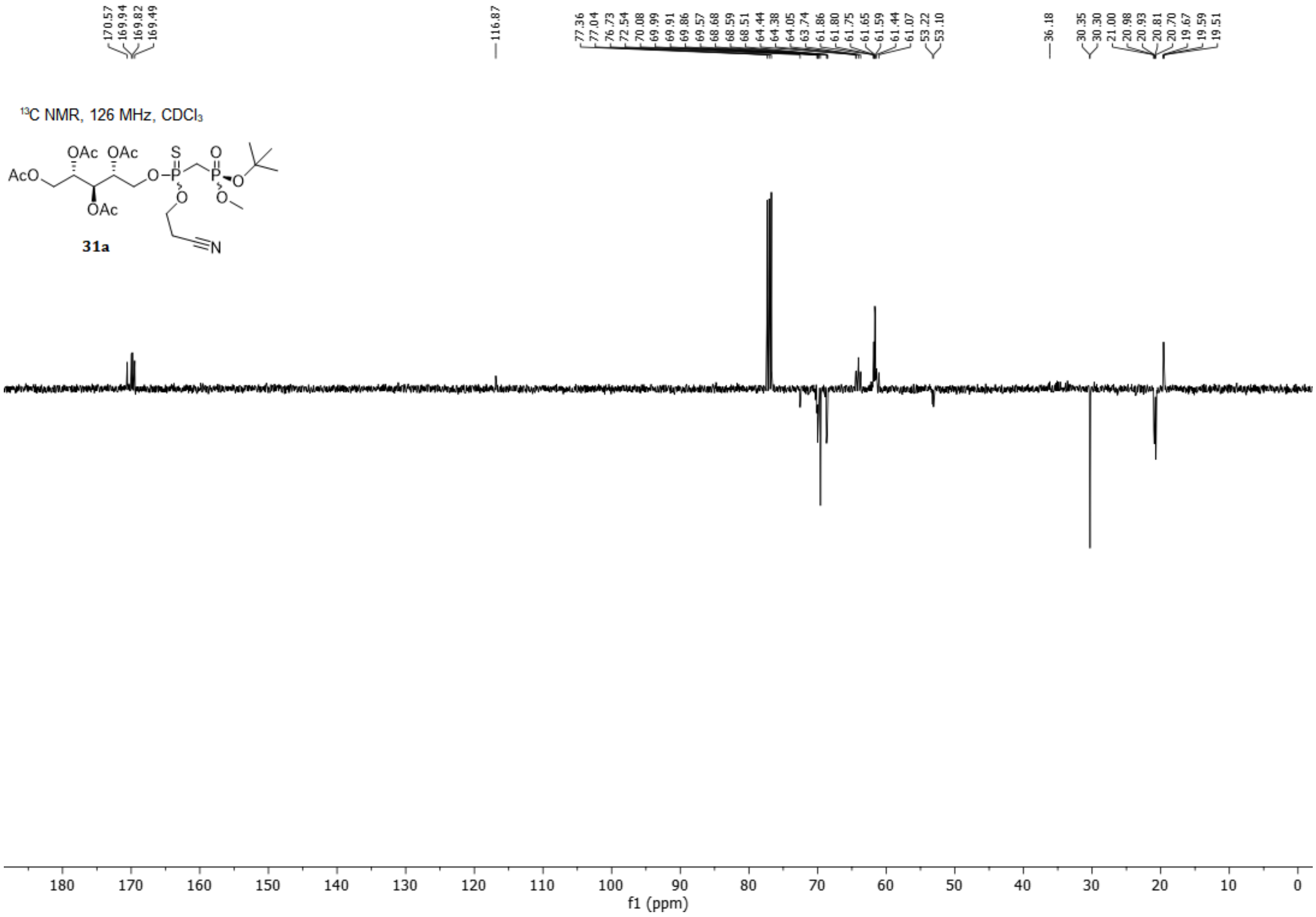

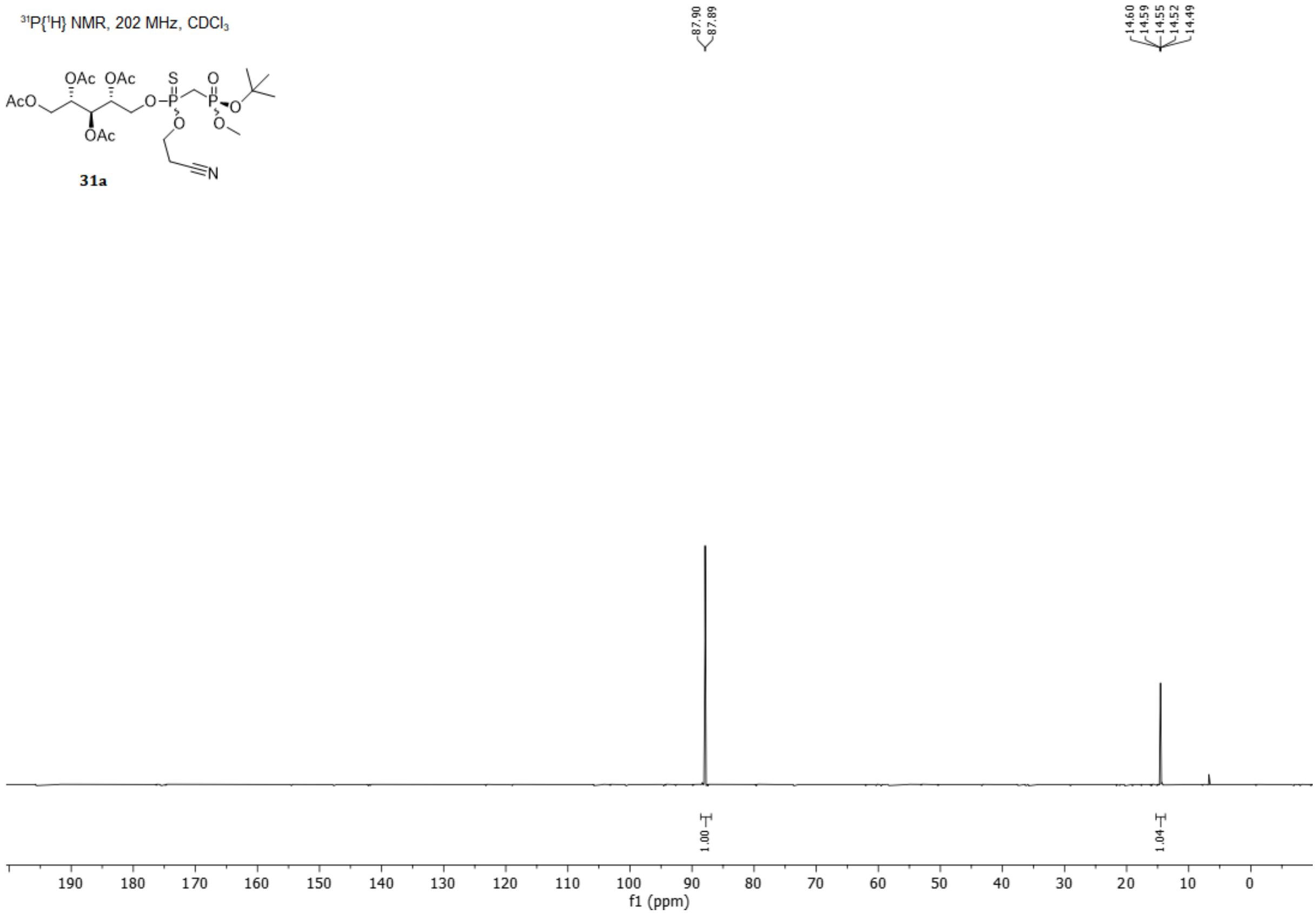
$^{31}P\{^1H\}$ NMR, 202 MHz, $CDCl_3$
31a
87.90
87.89
14.60
14.59
14.55
14.52
14.49
1.00
1.04
f1 (ppm)

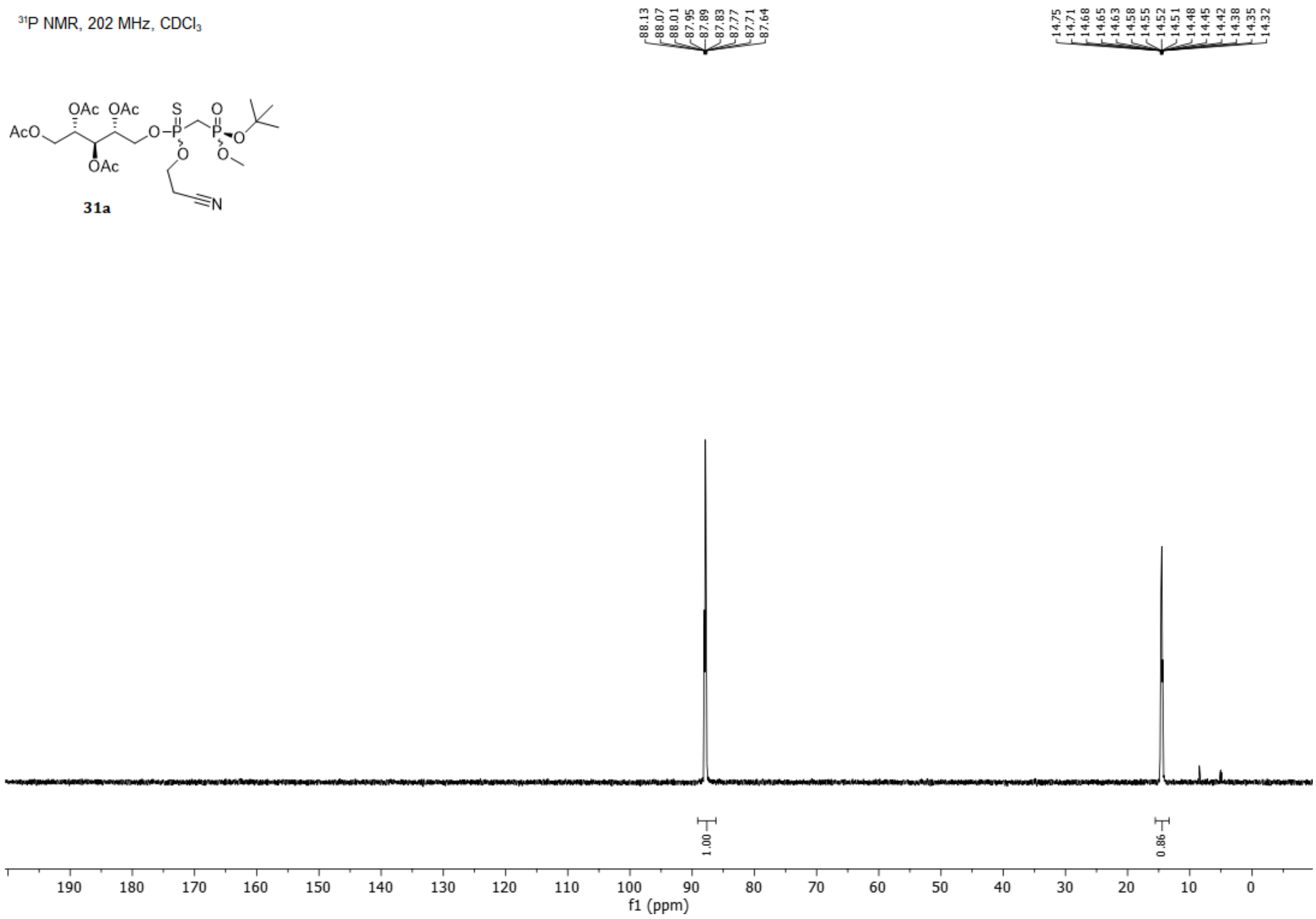
^{31}P NMR, 202 MHz, $CDCl_3$
31a
88.13
88.07
88.01
87.95
87.89
87.83
87.77
87.71
87.64
14.75
14.71
14.68
14.65
14.63
14.58
14.55
14.52
14.51
14.48
14.45
14.42
14.38
14.35
14.32
1.00
0.86
f1 (ppm)

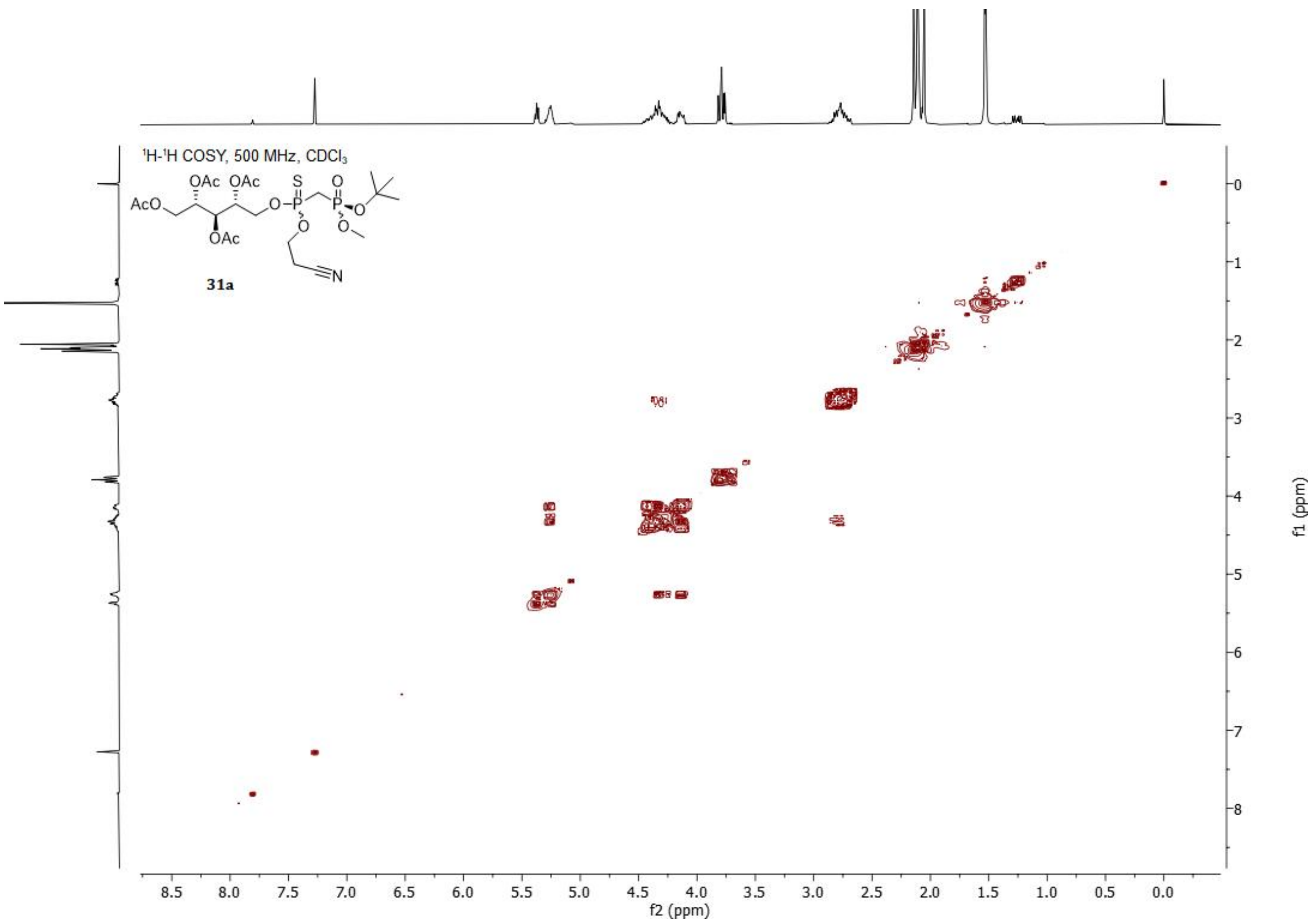

¹H-¹H COSY, 500 MHz, CDCl₃
AcO
OAc
OAc
OAc
O
P
S
O
P
O
O
O
N
f1 (ppm)
f2 (ppm)

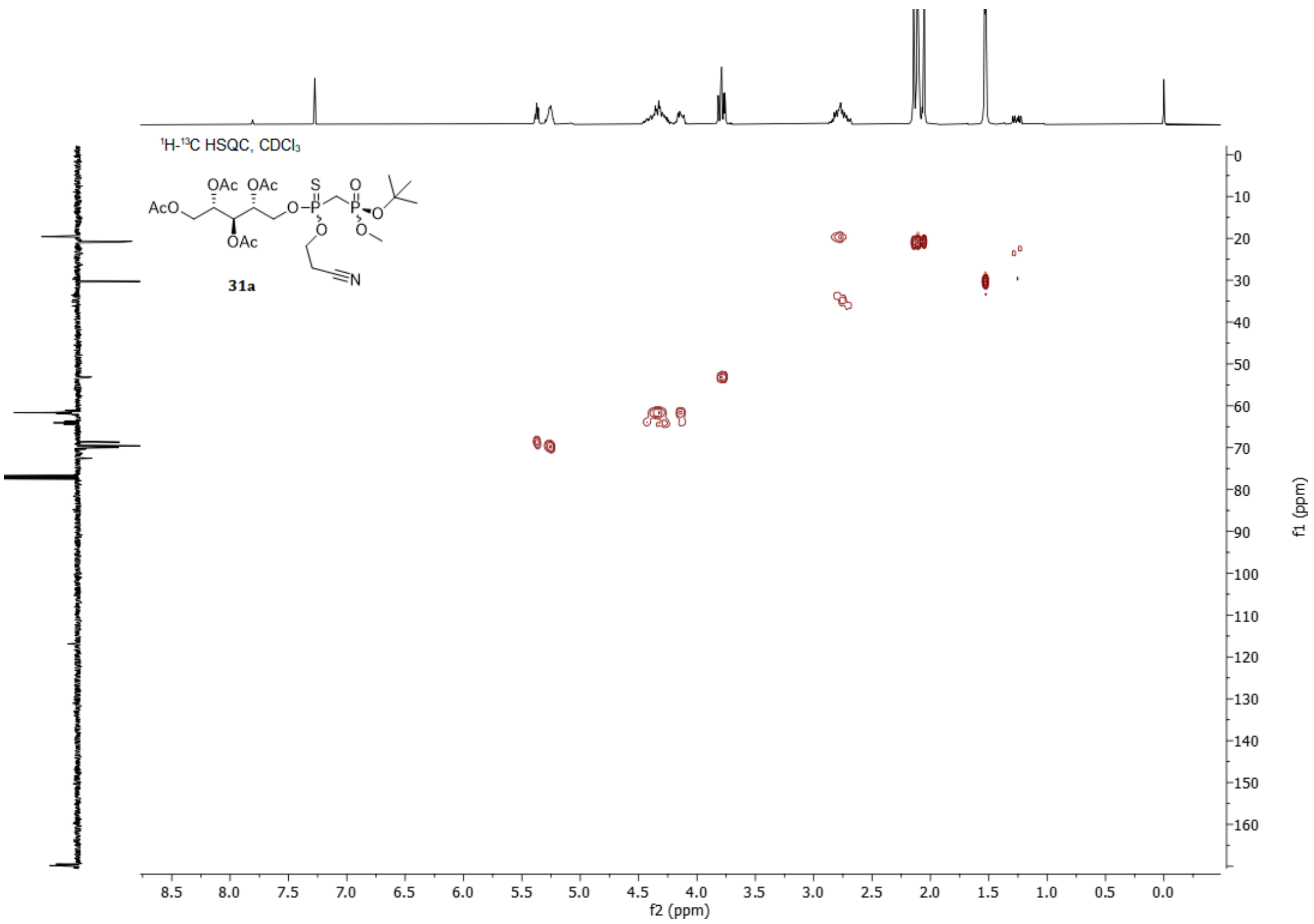

¹H-¹³C HSQC, CDCl₃
AcO
OAc
OAc
OAc
O
P
S
O
P
O
O
O
N
f1 (ppm)
f2 (ppm)

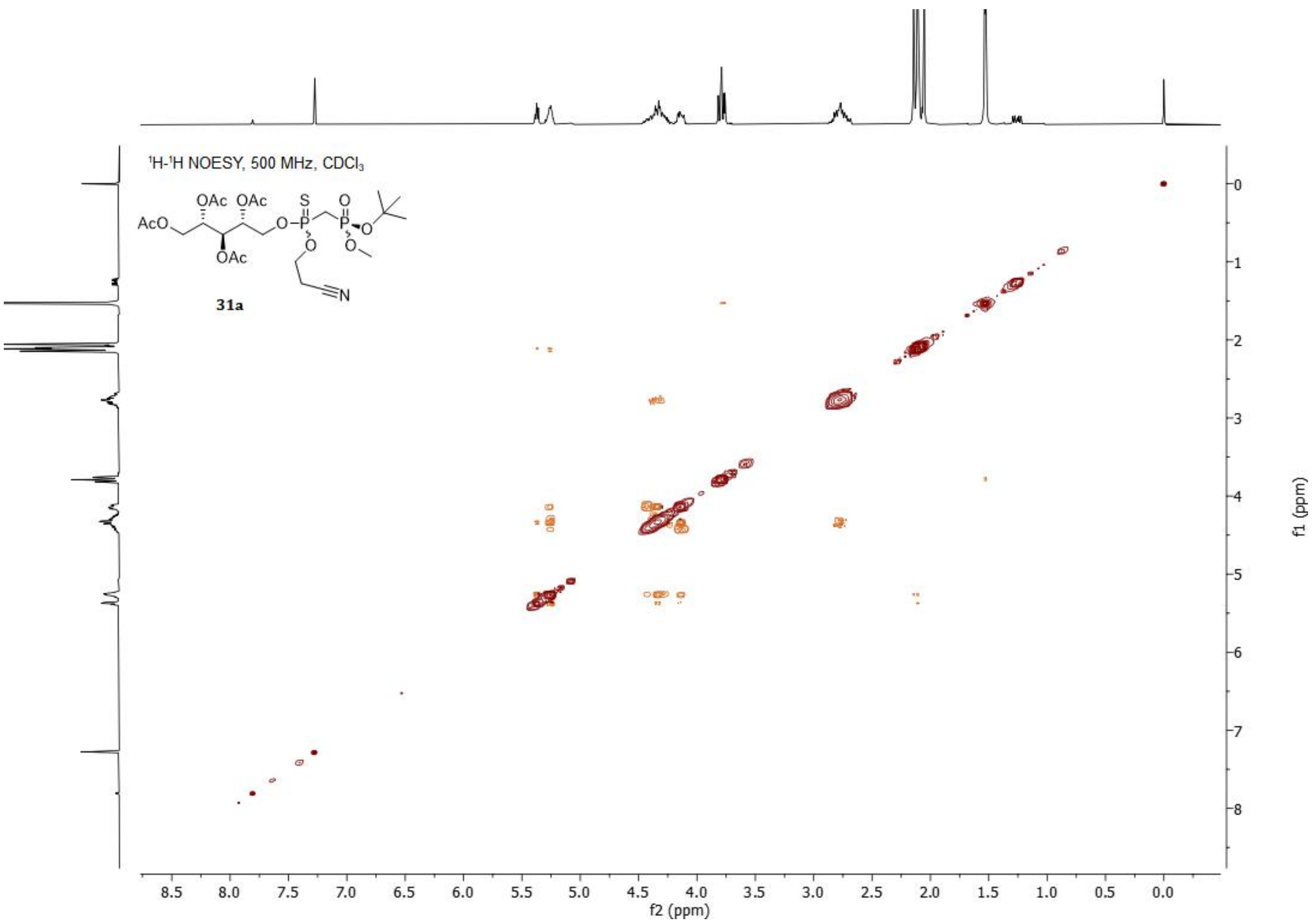
1H-1H NOESY, 500 MHz, CDCl3
AcO
OAc OAc
OAc
S
O
O
N
31a
f2 (ppm)
f1 (ppm)

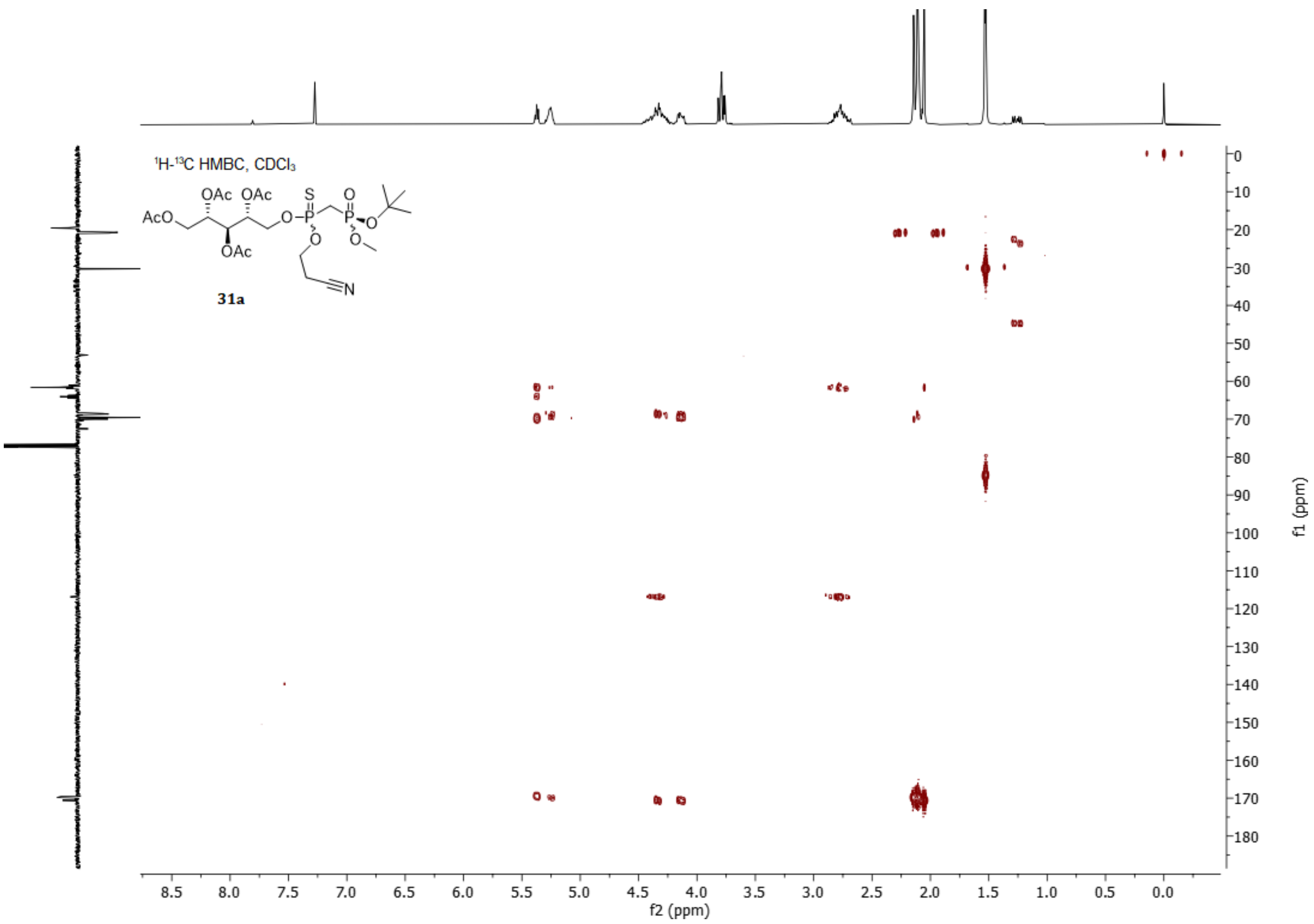
1H-13C HMBC, CDCl3
AcO
OAc OAc
OAc
S
O
O
N
31a
f2 (ppm)
f1 (ppm)

Tetra-acetyl- ᴅ-ribitol phosphonylmethyl phosphonodithioate tetraester 31b

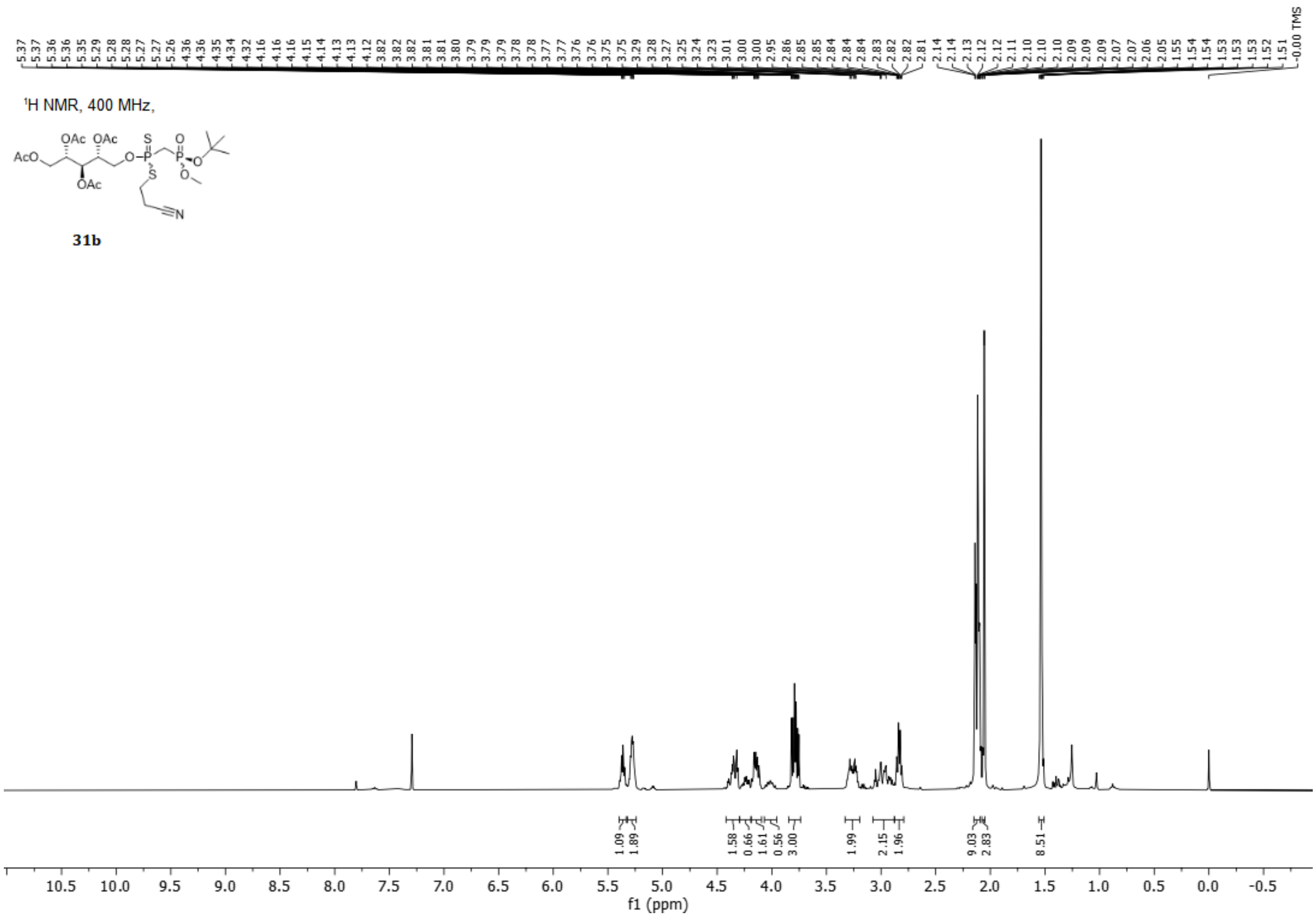

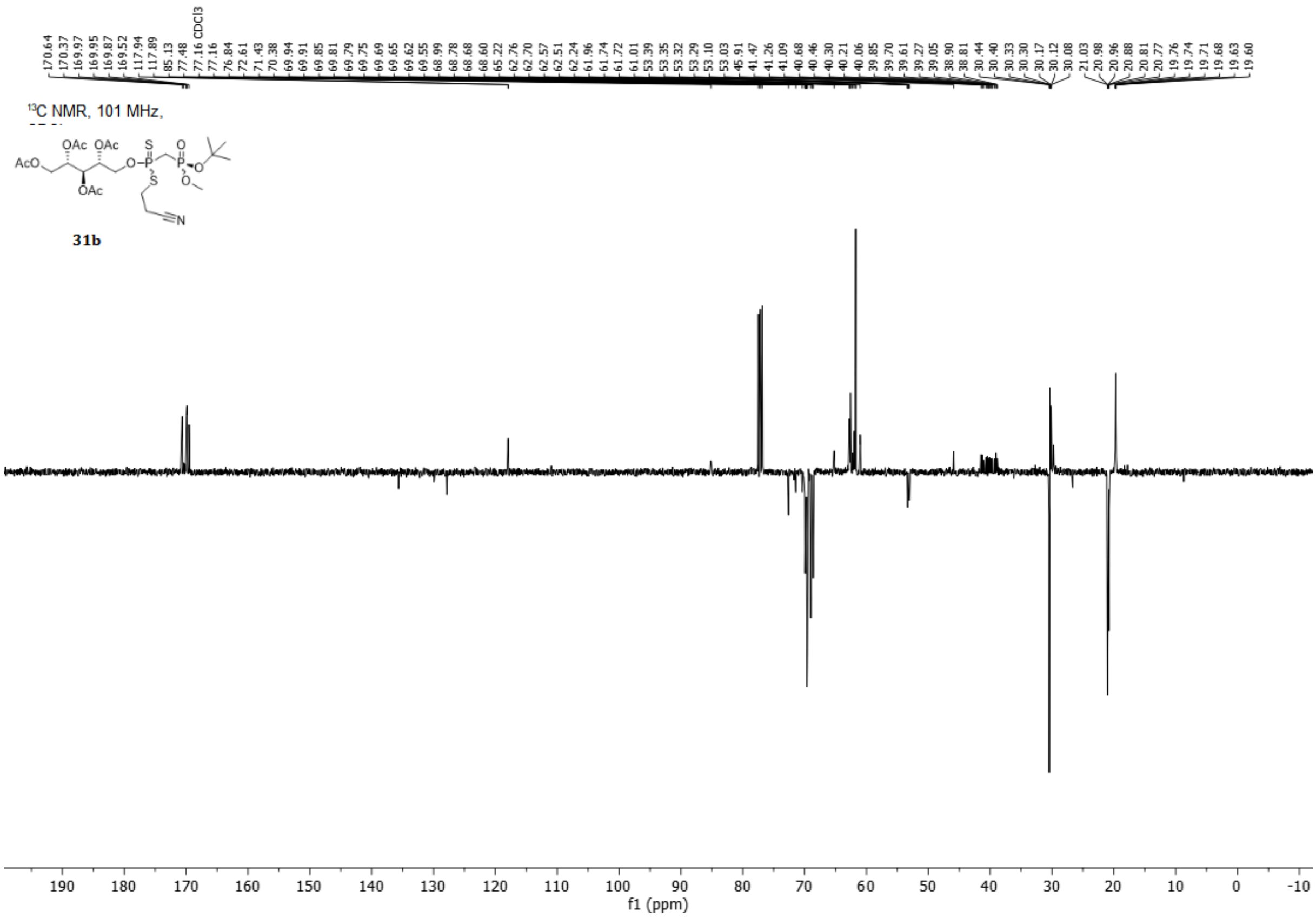

$^{31}P\{^{1}H\}$ NMR, 162 MHz,

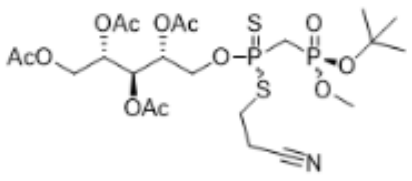

31b

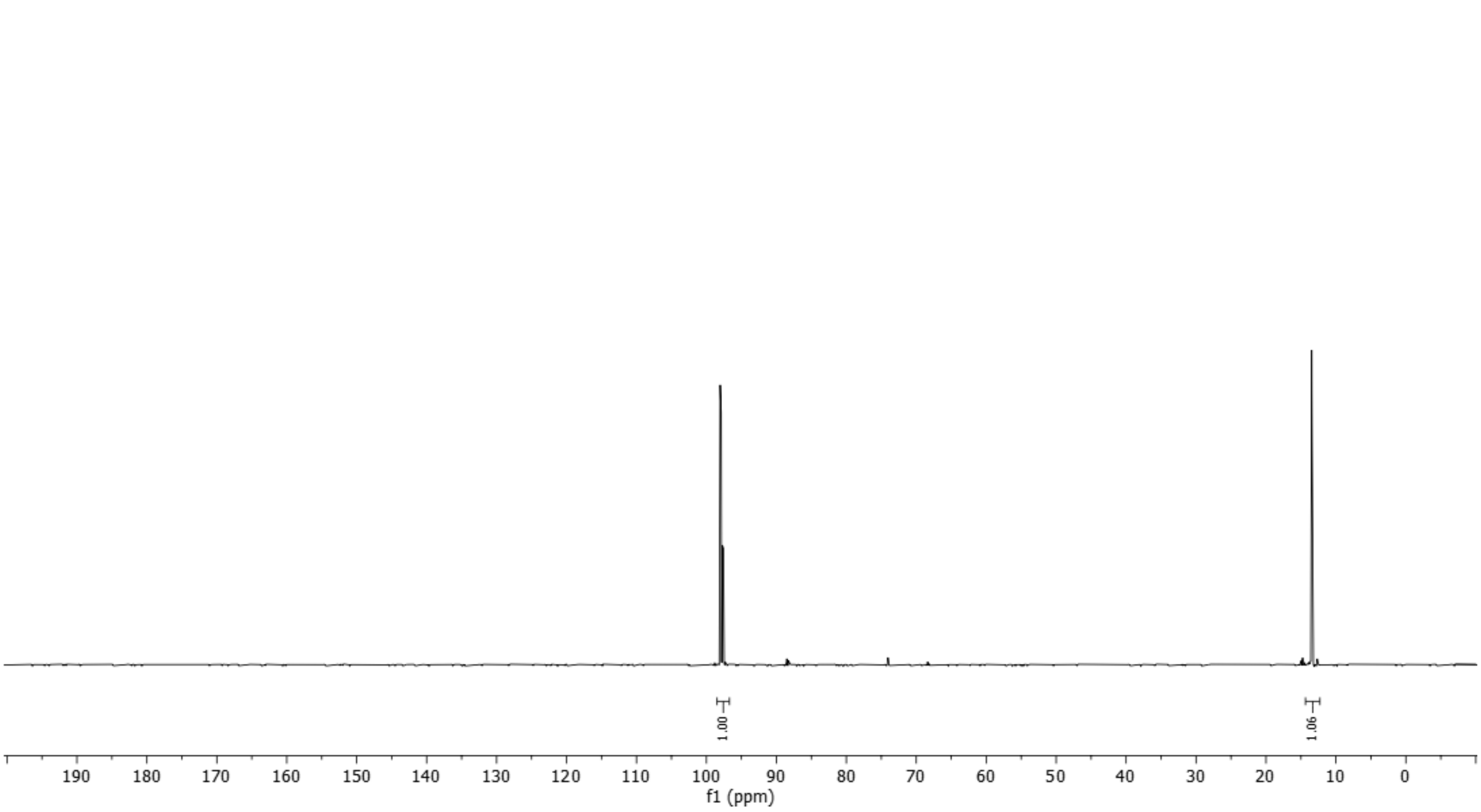

^{31}P NMR, 162 MHz,

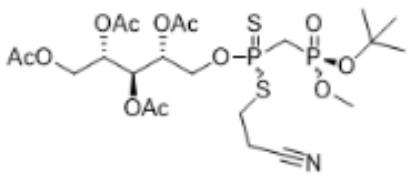

31b

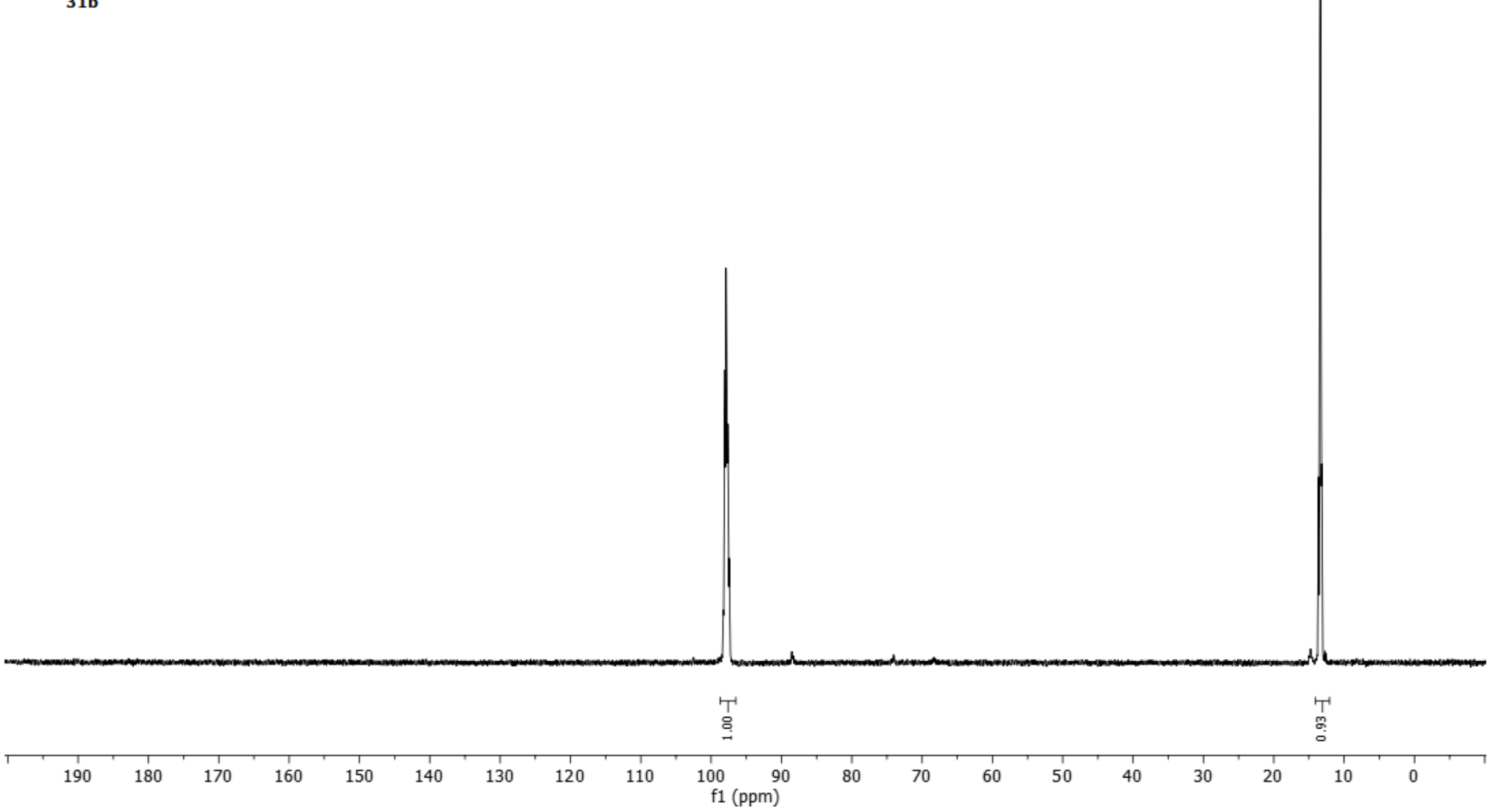

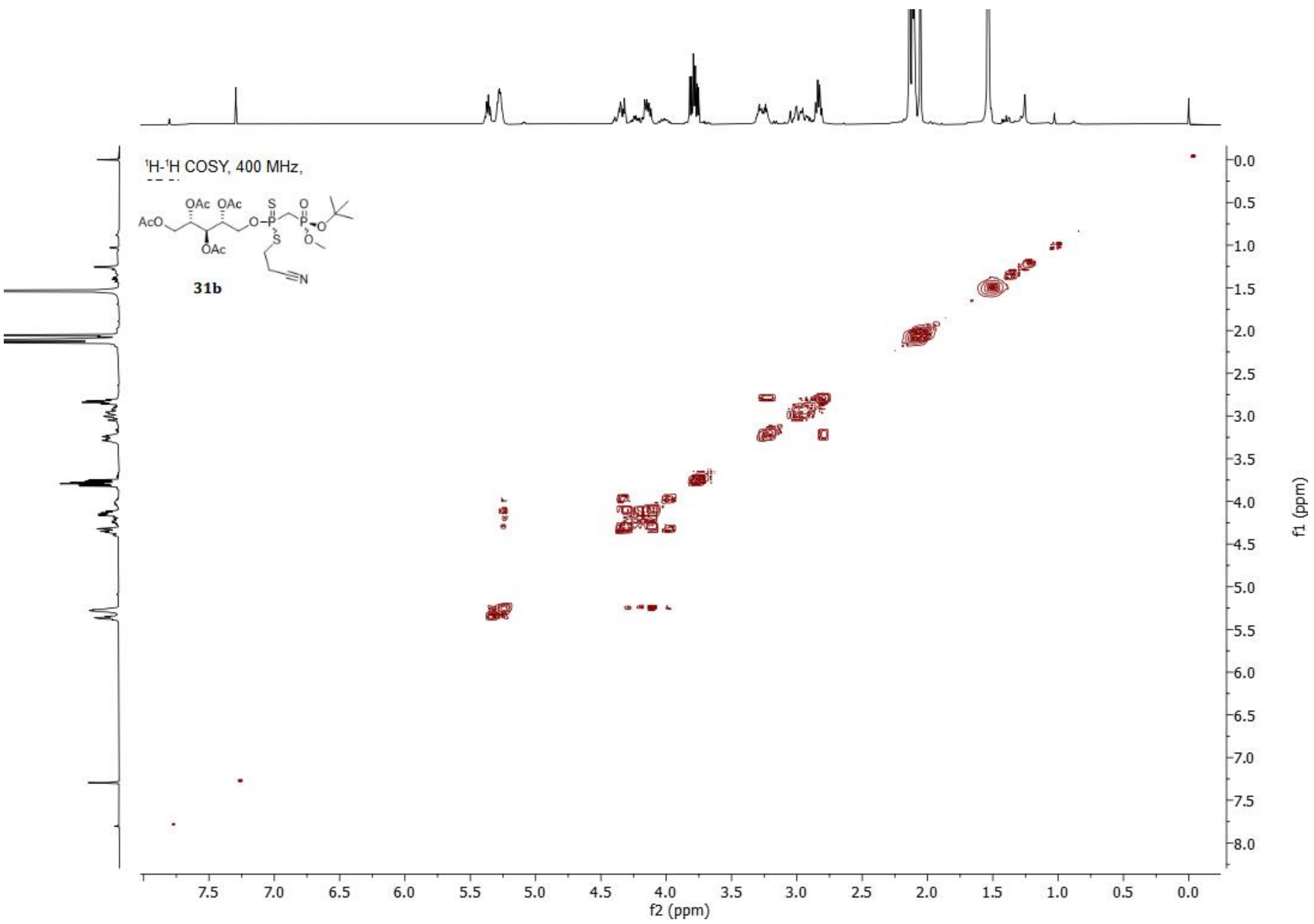

1H-1H COSY, 400 MHz,
31b
f2 (ppm)
f1 (ppm)

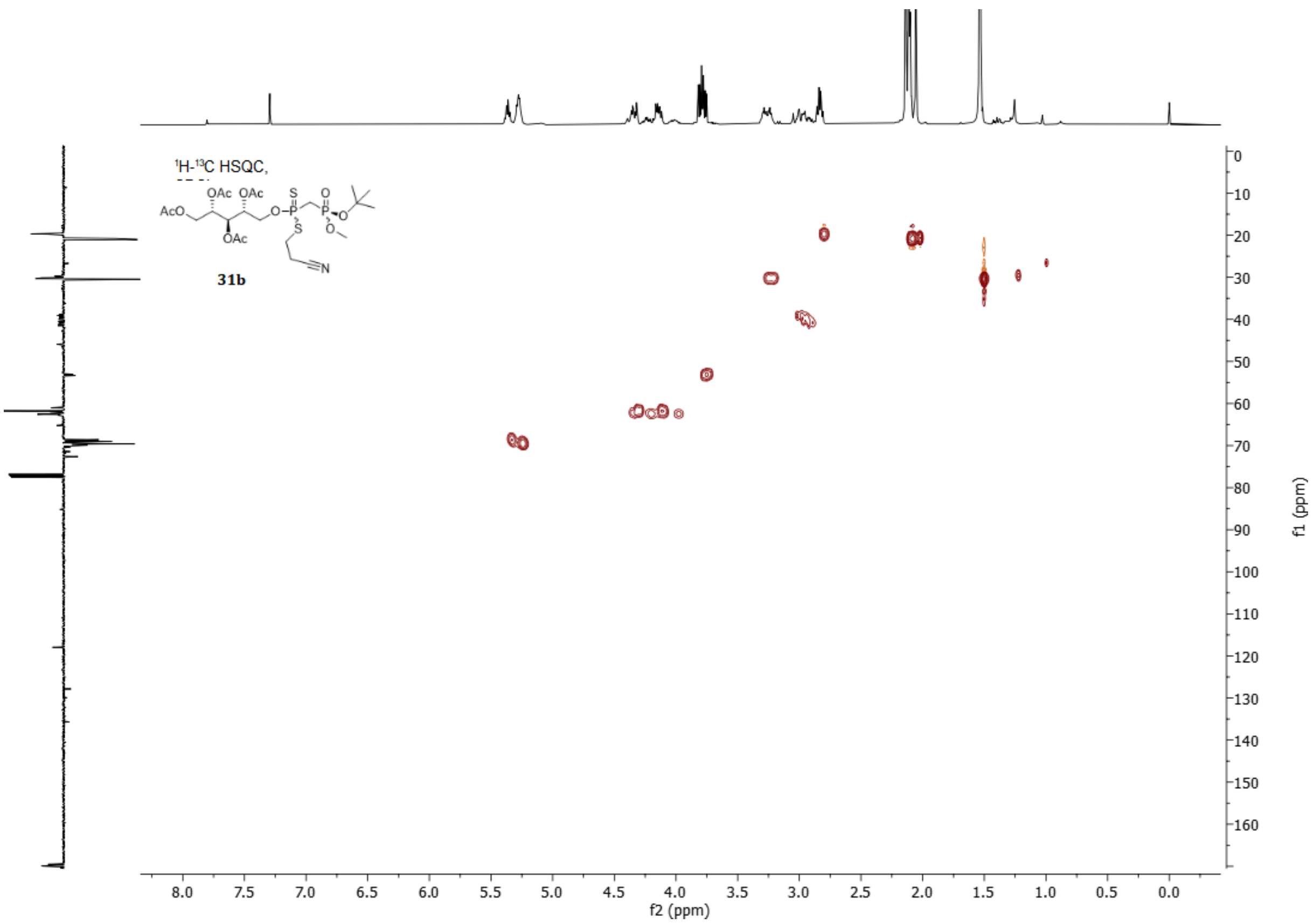

1H-13C HSQC,
31b
f2 (ppm)
f1 (ppm)

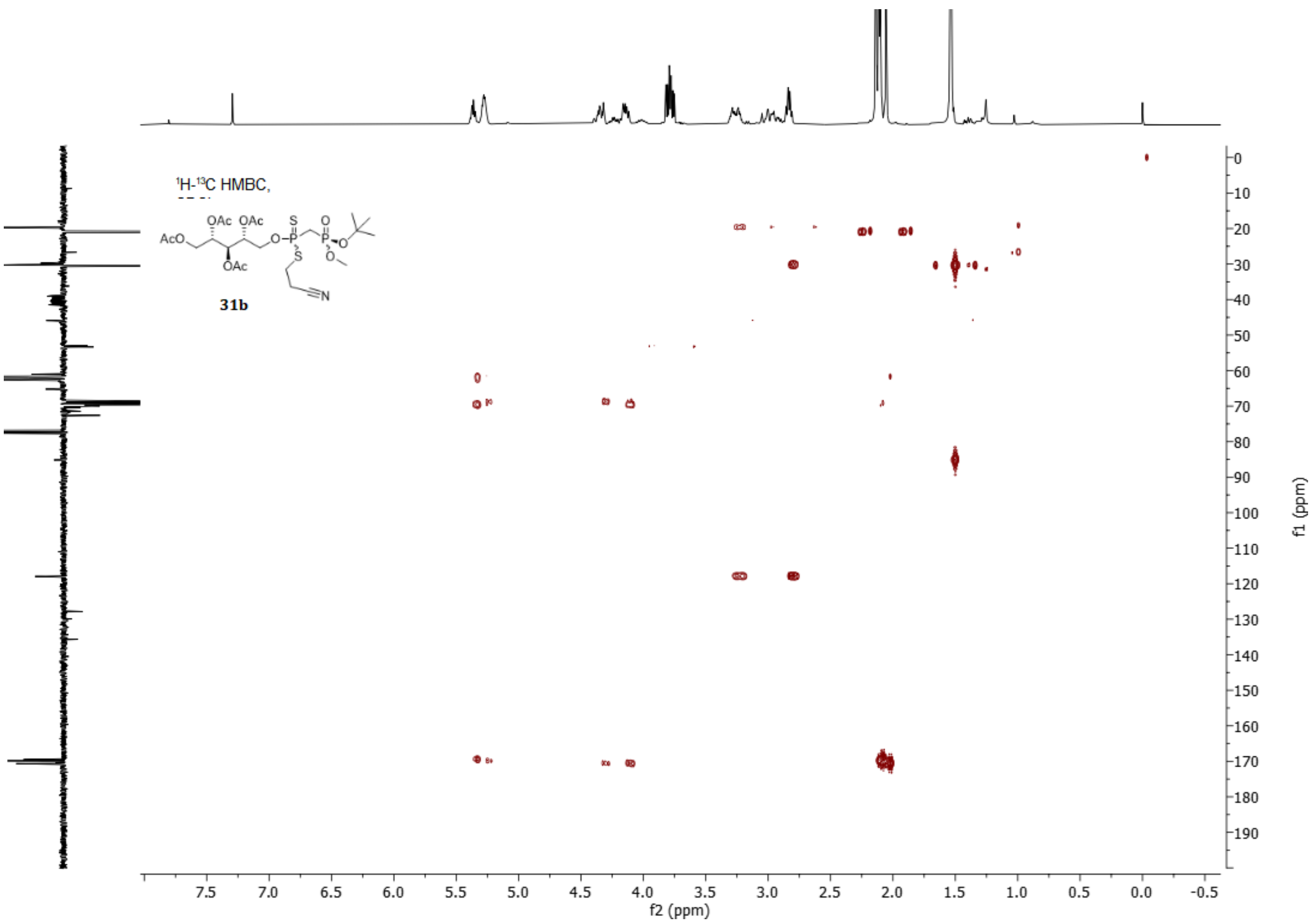

me-CDP-βS-ribitol tetraester 33a

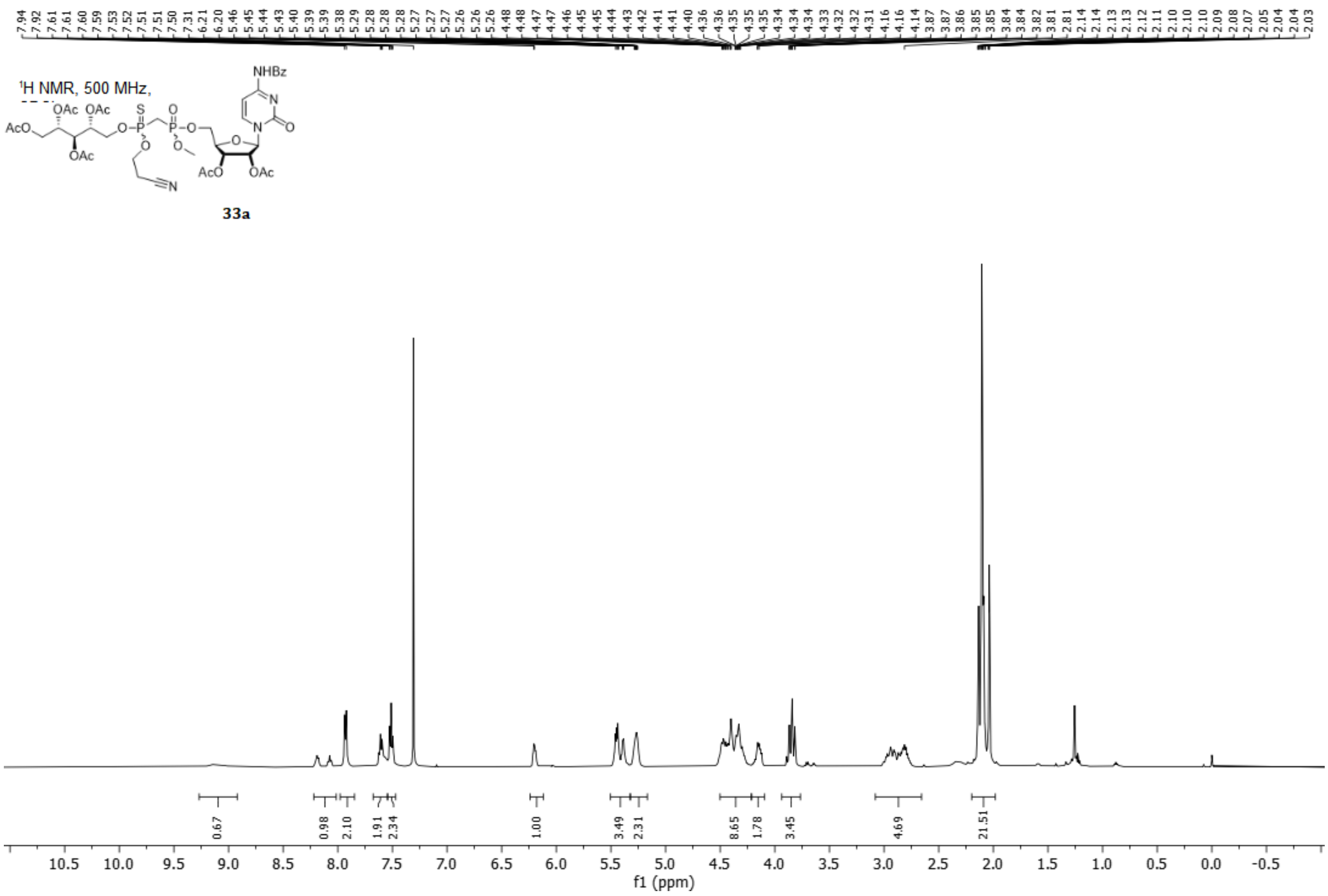

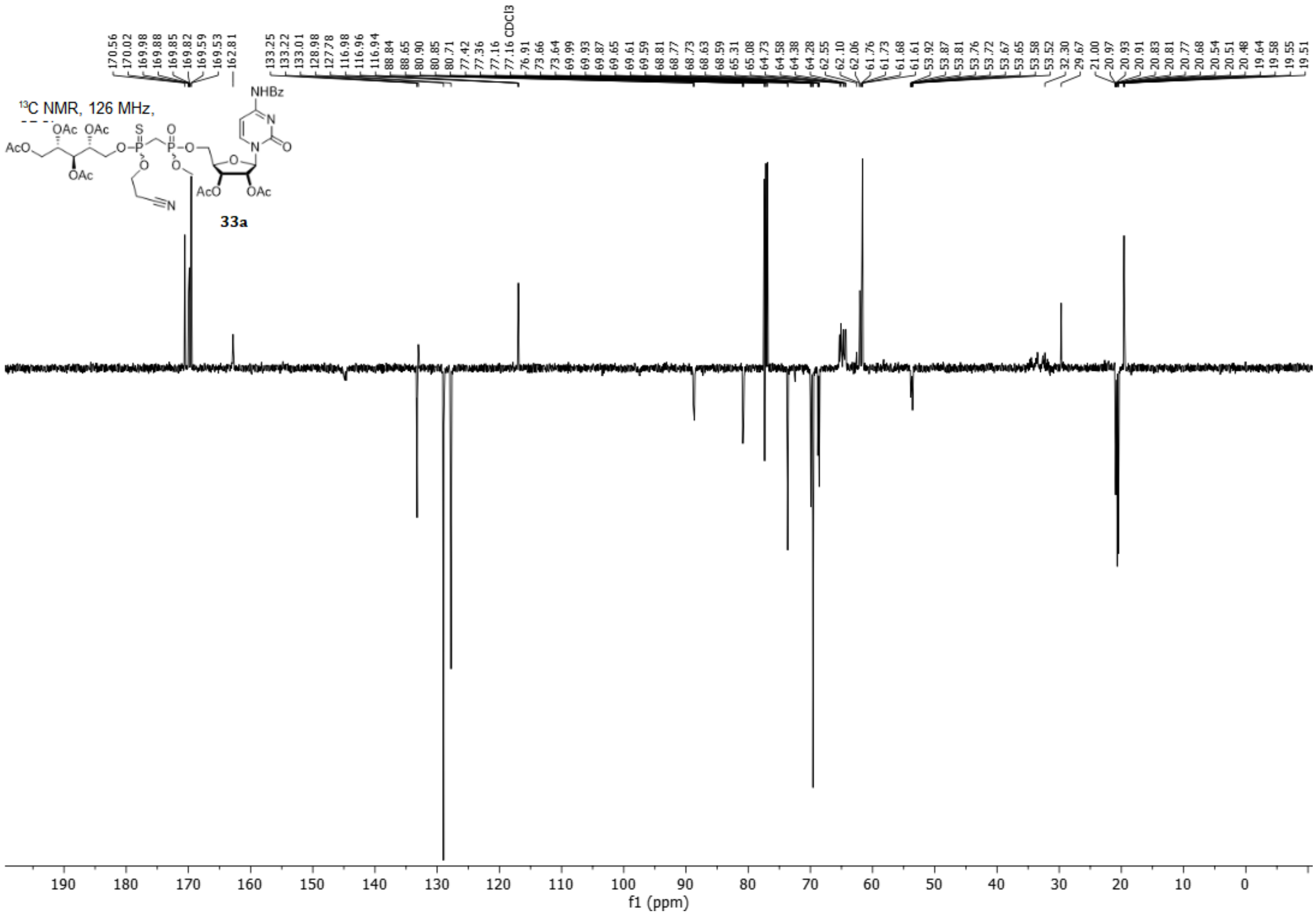

13C NMR, 126 MHz,
170.56 170.02 169.98 169.88 169.85 169.82 169.59 169.53 162.81
133.25 133.22 133.01 128.98 127.78 116.98 116.96 116.94 88.84 88.65 80.90 80.85 80.71 77.42 77.36 77.16 77.16 CDCl3 76.91 73.66 73.64 69.99 69.93 69.87 69.65 69.61 69.59 68.81 68.77 68.73 68.63 68.59 65.31 65.08 64.73 64.58 64.38 64.28 62.55 62.10 62.06 61.76 61.73 61.68 61.61 53.92 53.87 53.81 53.76 53.72 53.67 53.65 53.58 53.52 32.30 29.67 21.00 20.97 20.93 20.91 20.83 20.81 20.77 20.68 20.54 20.51 20.48 19.64 19.58 19.55 19.51
NHBz
OAc OAc
AcO
OAc
S
O
N
O
AcO
OAc
N
33a
f1 (ppm)

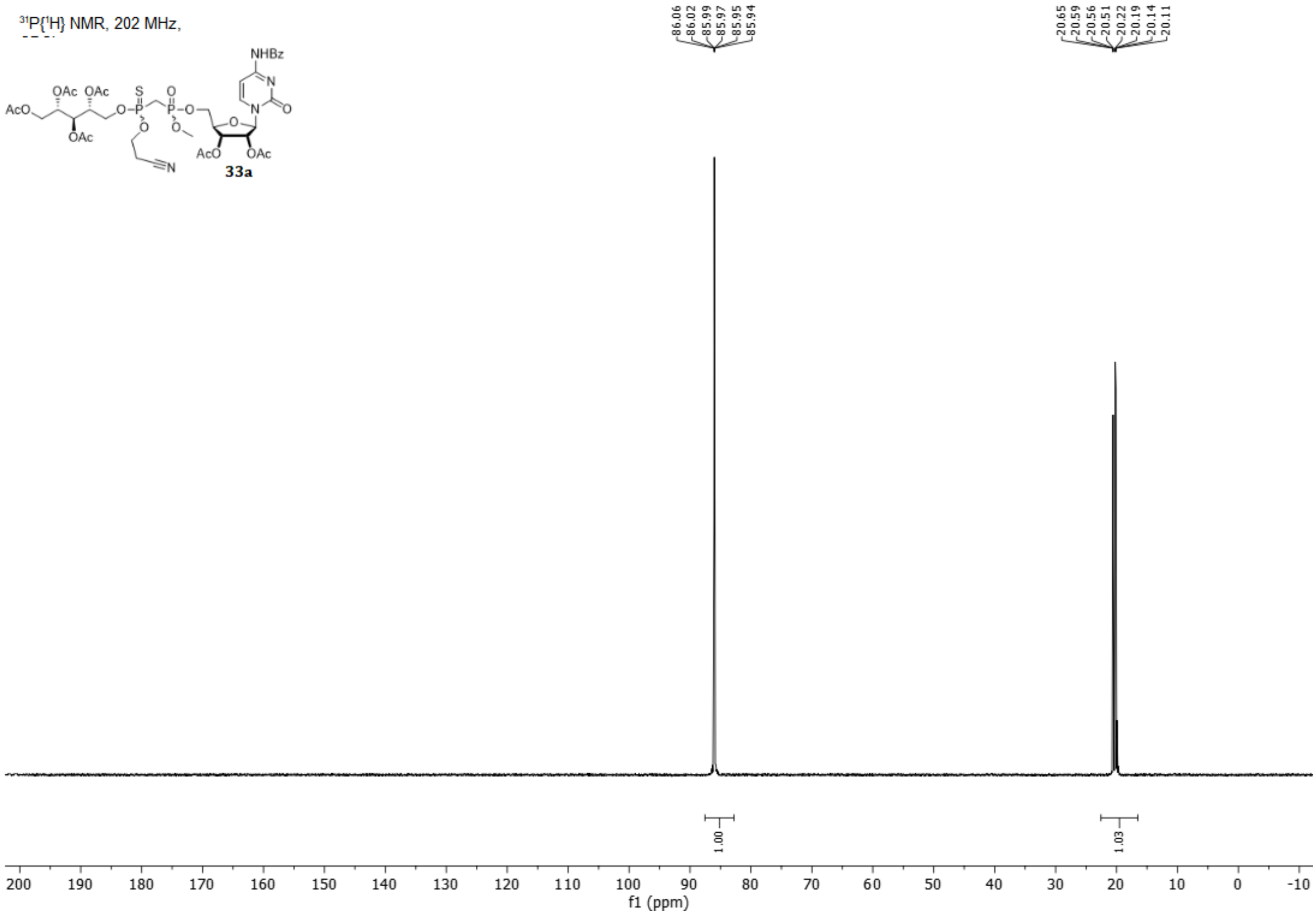

31P{1H} NMR, 202 MHz,
86.06 86.02 85.99 85.97 85.95 85.94
20.65 20.59 20.56 20.51 20.22 20.19 20.14 20.11
NHBz
OAc OAc
AcO
OAc
S
O
N
O
AcO
OAc
N
33a
1.00
1.03
f1 (ppm)

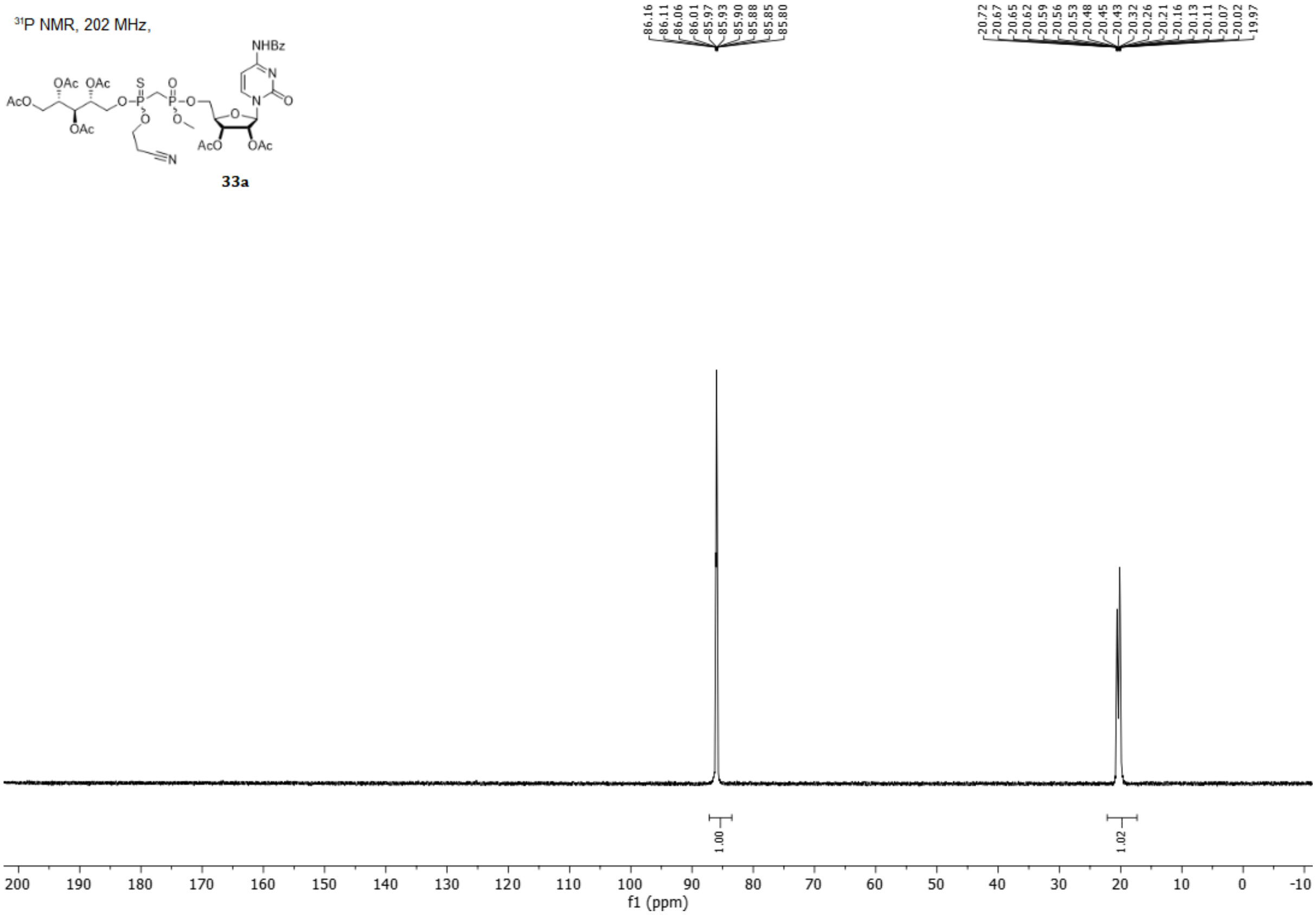

^{31}P NMR, 202 MHz,
NHBz
AcO
OAc
S
O
N
33a
86.16
86.11
86.06
86.01
85.97
85.93
85.90
85.88
85.85
85.80
20.72
20.67
20.65
20.62
20.59
20.56
20.53
20.48
20.45
20.43
20.32
20.26
20.21
20.16
20.13
20.11
20.07
20.02
19.97
1.00
1.02
f1 (ppm)

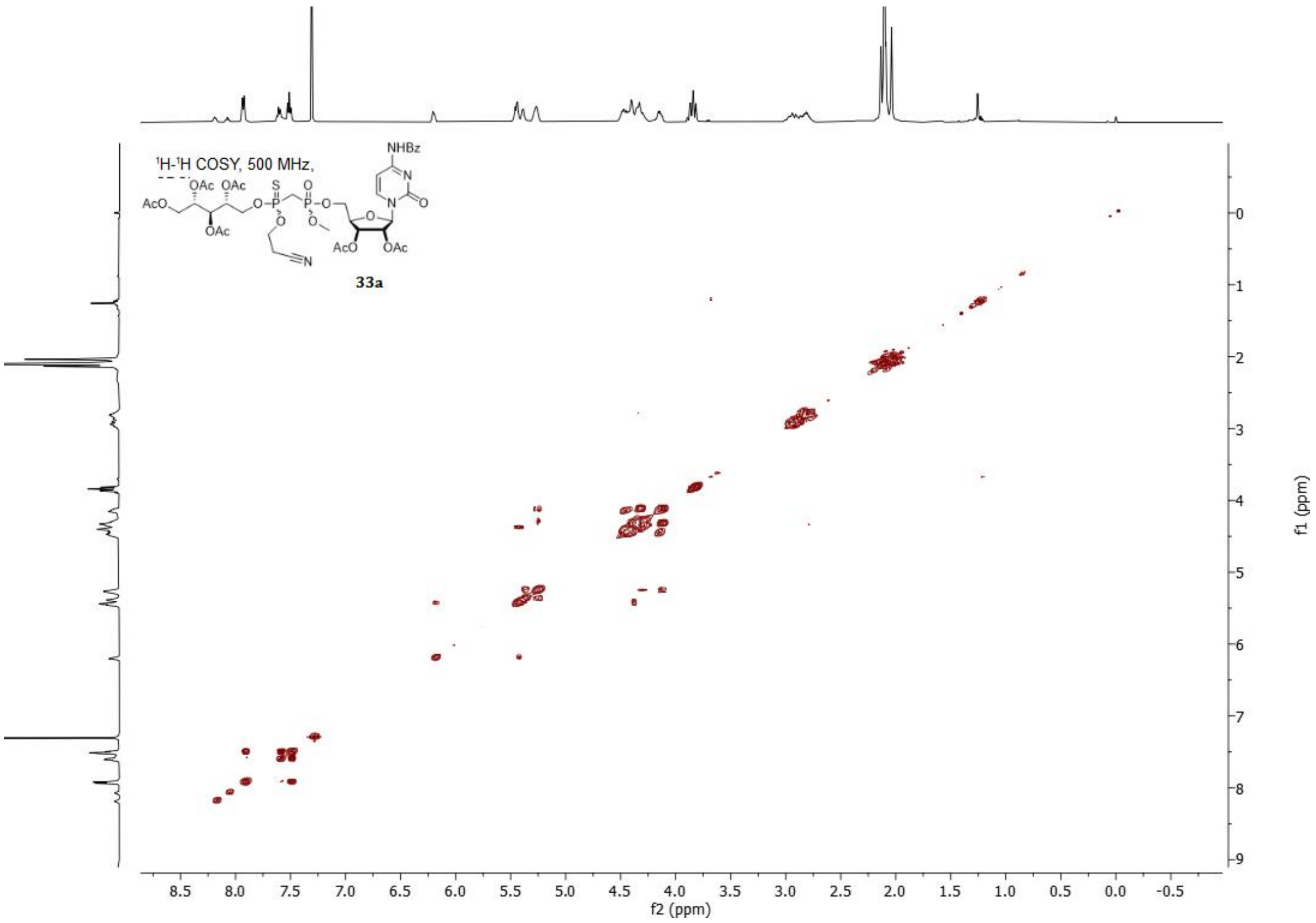

^{1}H-^{1}H COSY, 500 MHz,
NHBz
AcO
OAc
S
O
N
33a
f2 (ppm)
f1 (ppm)

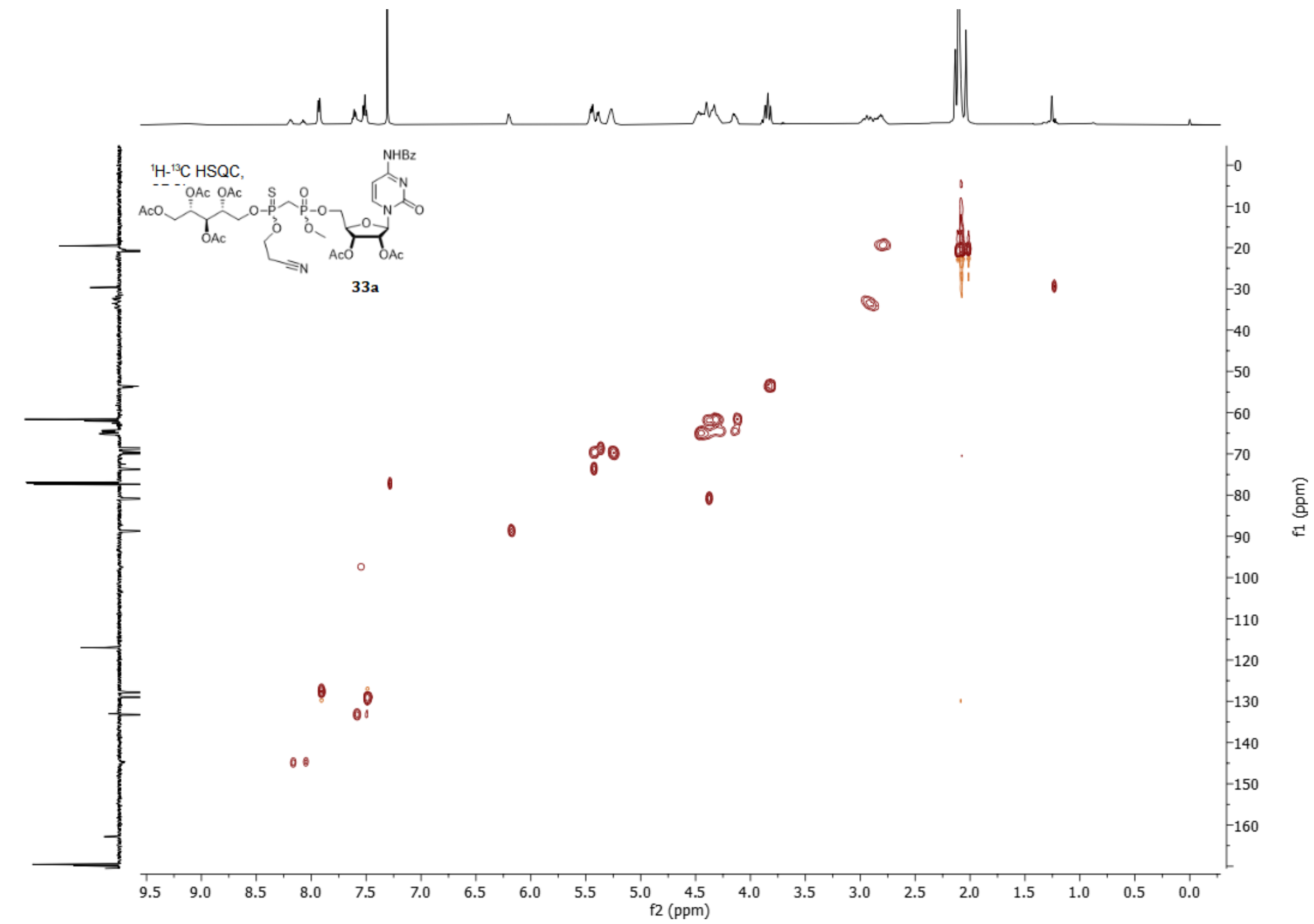

1H-13C HSQC,
NHBz
AcO OAc OAc OAc S O AcO OAc
33a
f2 (ppm)
f1 (ppm)

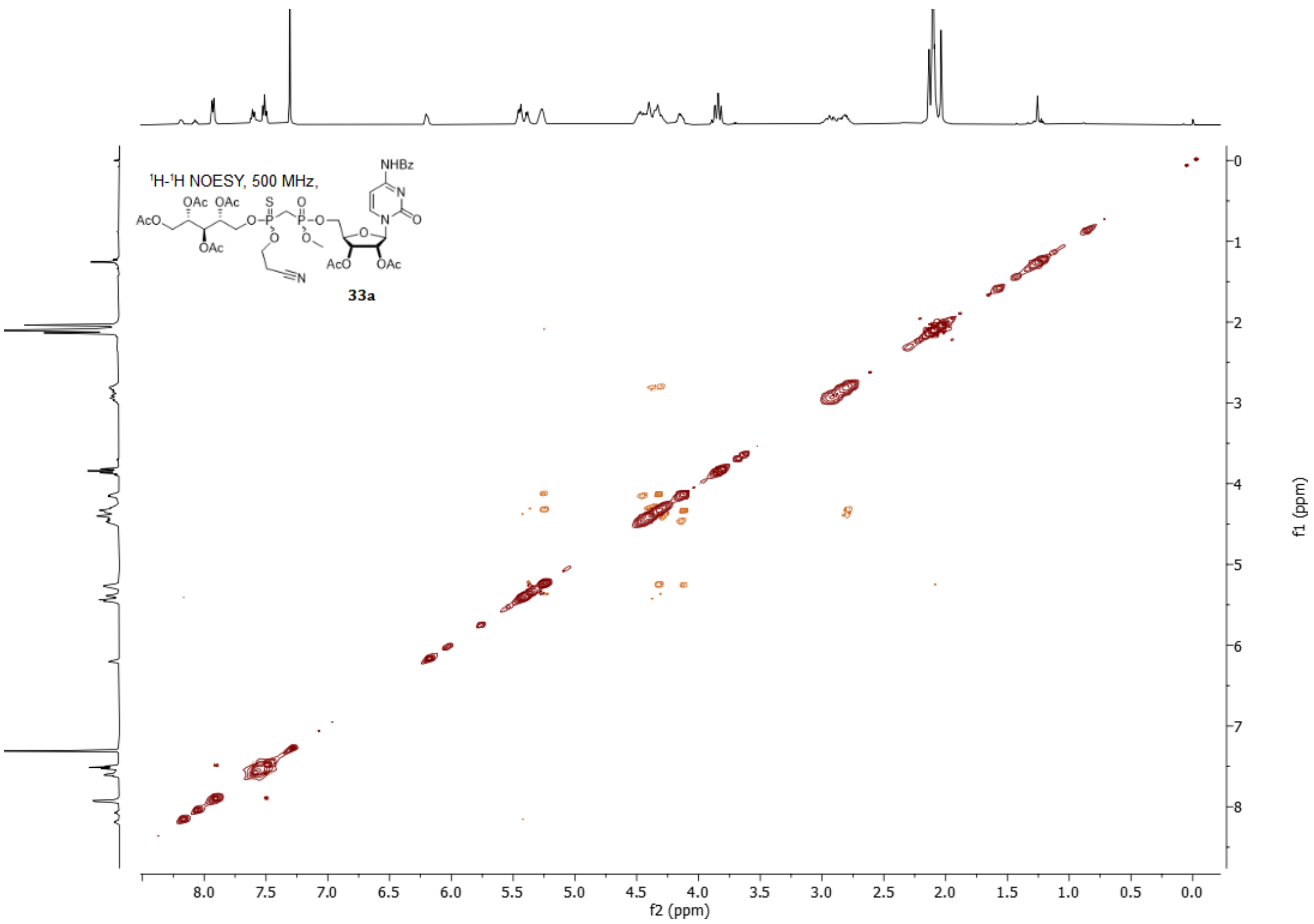

1H-1H NOESY, 500 MHz,
NHBz
33a
f2 (ppm)
f1 (ppm)

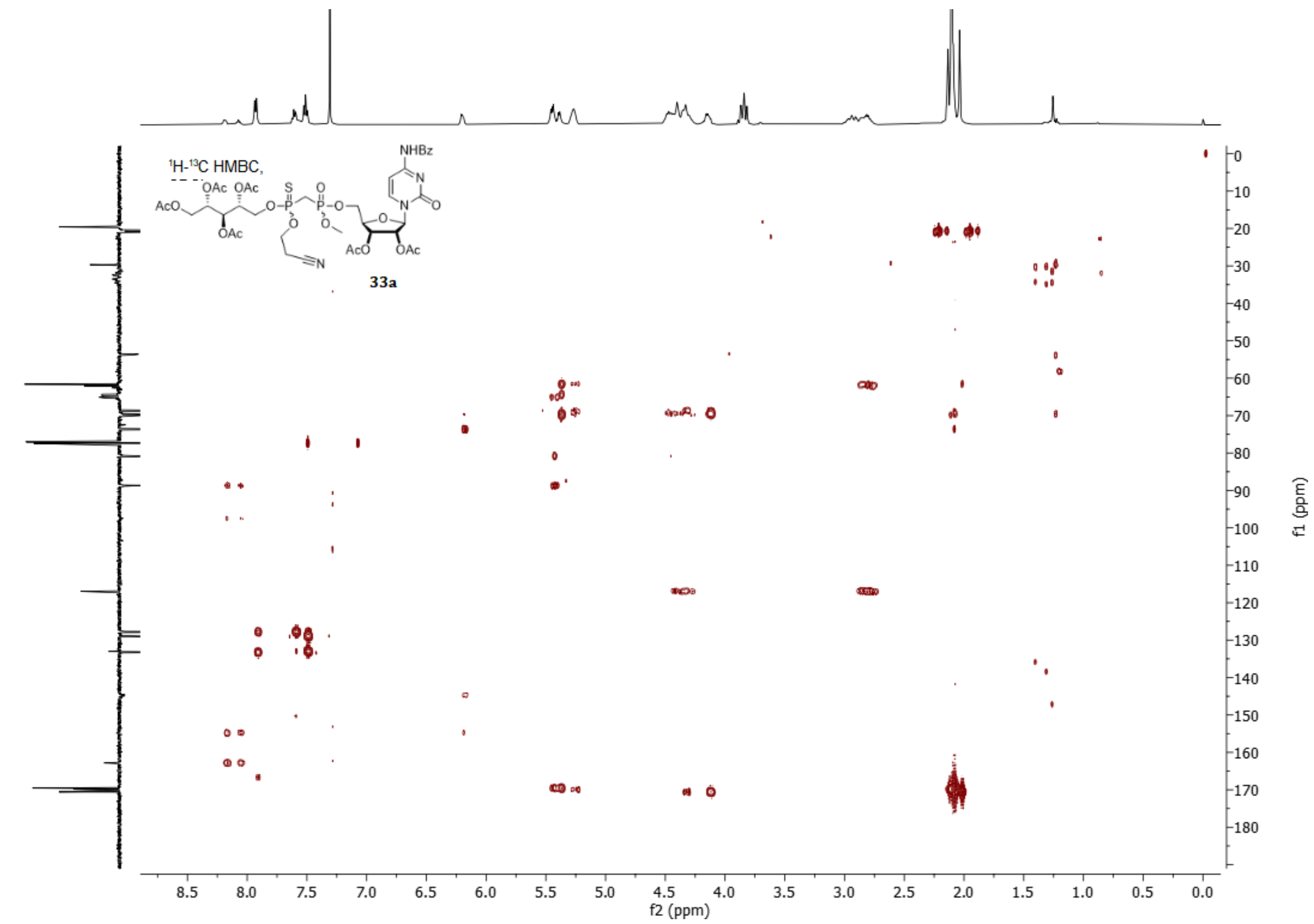

me-CDP-βS$_2$-ribitol tetraester 33b

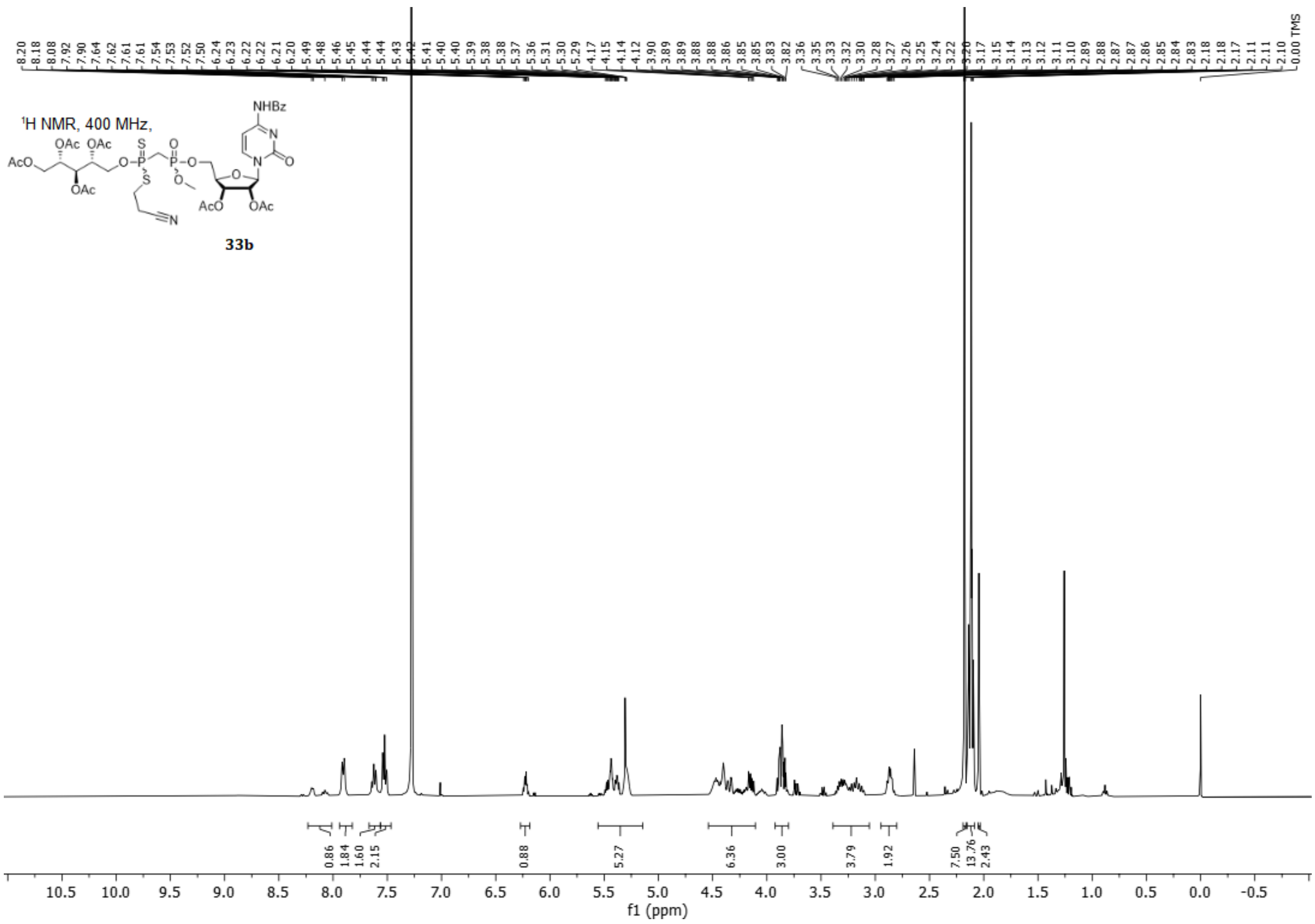

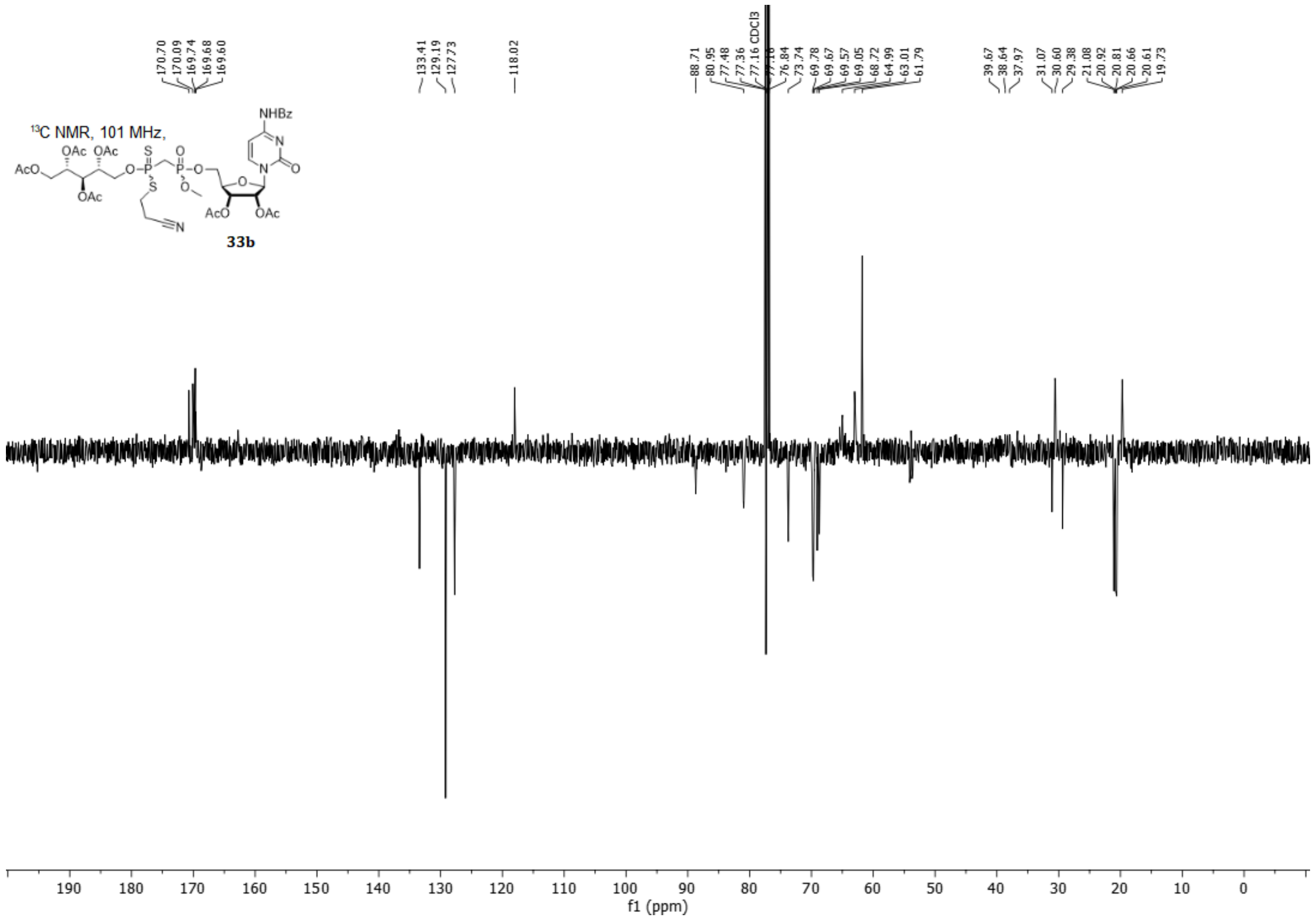

13C NMR, 101 MHz,
NHBz
33b
170.70 170.09 169.74 169.68 169.60
133.41 129.19 127.73
118.02
88.71 80.95 77.48 77.36 77.16 CDCl3 76.84 73.74 69.78 69.67 69.57 69.05 68.72 64.99 63.01 61.79
39.67 38.64 37.97 31.07 30.60 29.38 21.08 20.92 20.81 20.66 20.61 19.73
f1 (ppm)

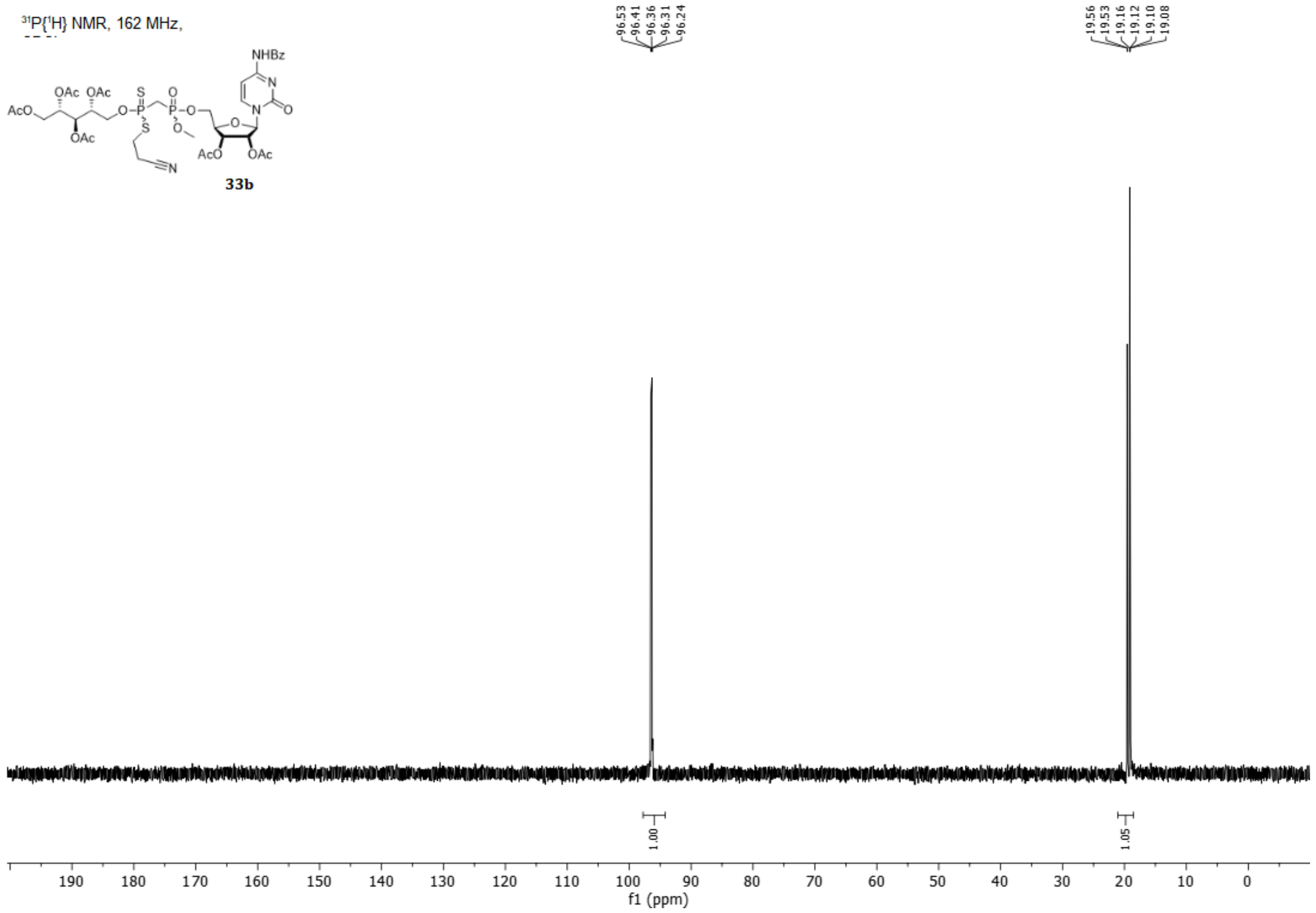

31P{1H} NMR, 162 MHz,
NHBz
33b
96.53 96.41 96.36 96.31 96.24
19.56 19.53 19.16 19.12 19.10 19.08
1.00
1.05
f1 (ppm)

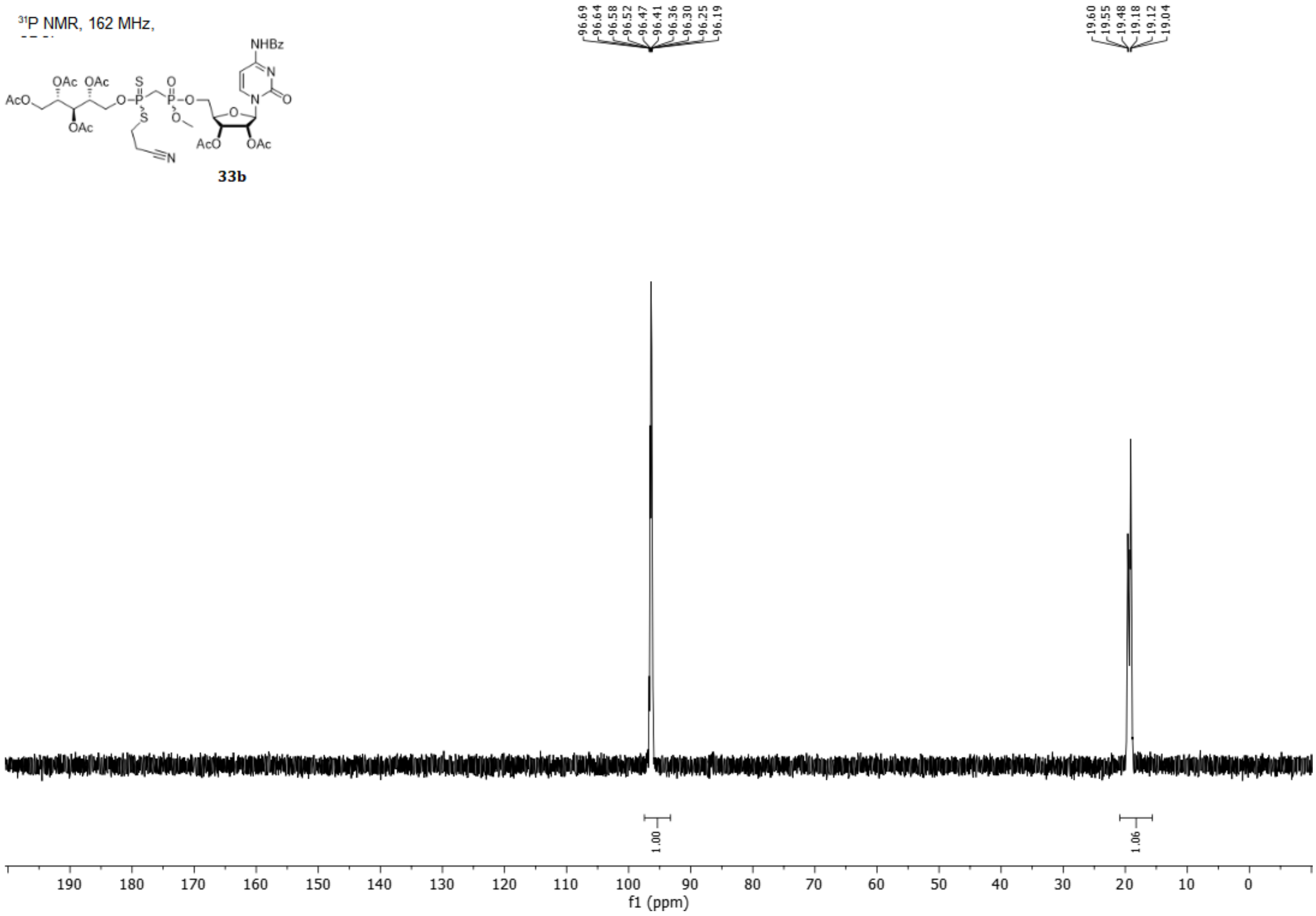

31P NMR, 162 MHz,
NHBz
OAc OAc
AcO
OAc
S
O
O-P
S
P-O
O
O
N
N
O
AcO
OAc
N
33b
96.69
96.64
96.58
96.52
96.47
96.41
96.36
96.30
96.25
96.19
19.60
19.55
19.48
19.18
19.12
19.04
1.00
1.06
190 180 170 160 150 140 130 120 110 100 90 80 70 60 50 40 30 20 10 0
f1 (ppm)

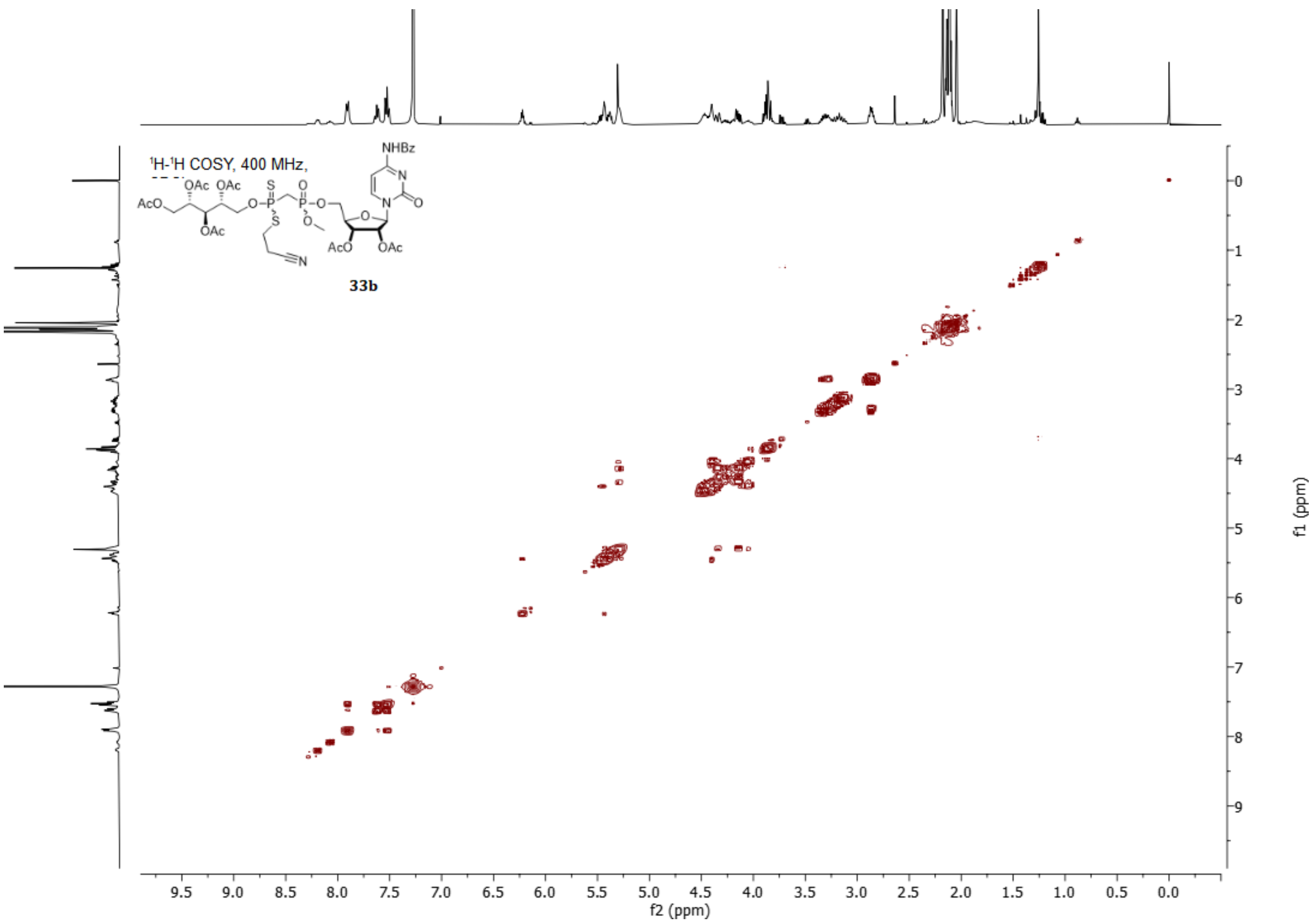

1H-1H COSY, 400 MHz,
NHBz
OAc OAc
AcO
OAc
S
O
O-P
S
P-O
O
O
N
N
O
AcO
OAc
N
33b
9.5 9.0 8.5 8.0 7.5 7.0 6.5 6.0 5.5 5.0 4.5 4.0 3.5 3.0 2.5 2.0 1.5 1.0 0.5 0.0
f2 (ppm)
0 1 2 3 4 5 6 7 8 9
f1 (ppm)

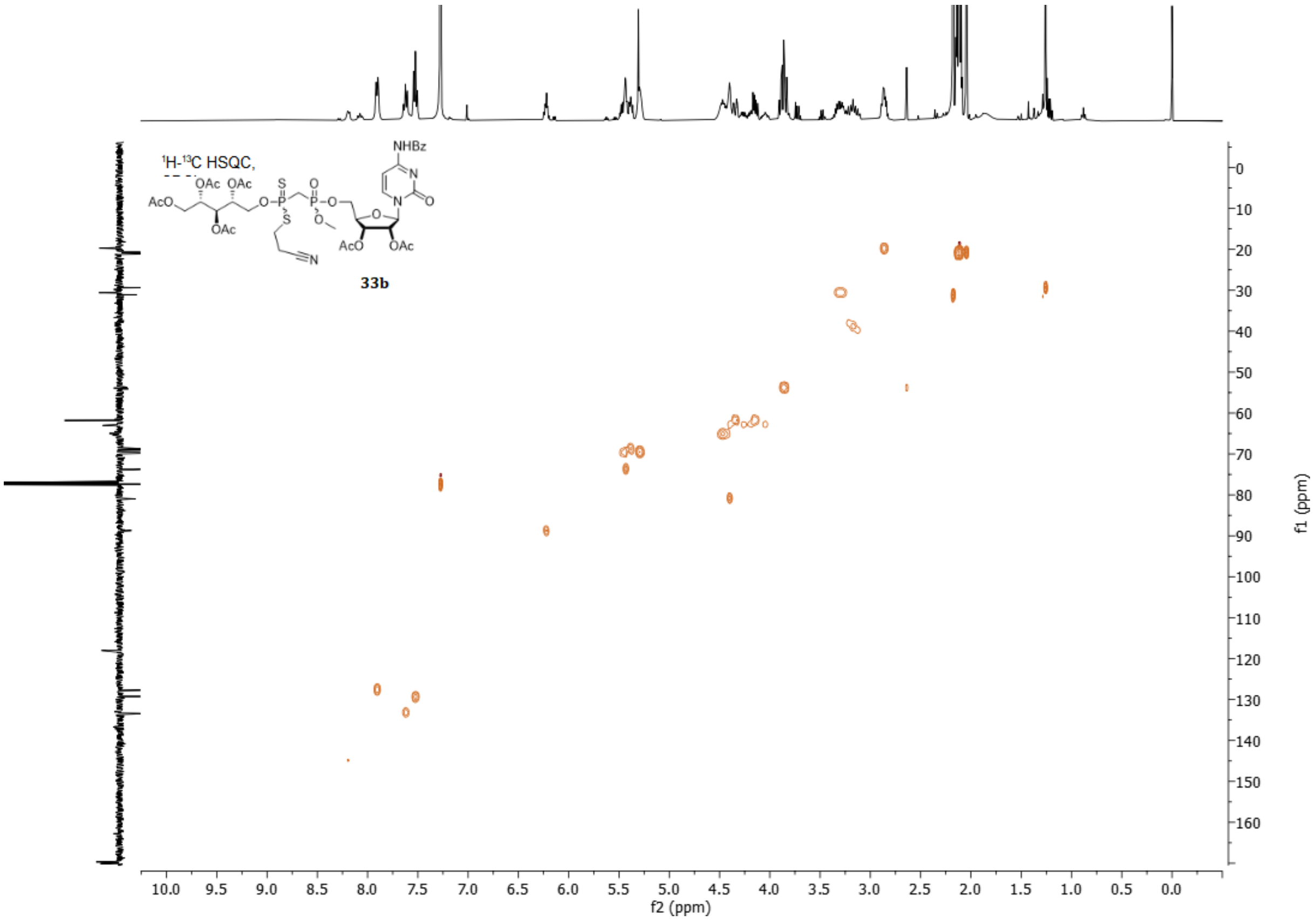

¹H-¹³C HSQC,
NHBz
AcO
OAc
33b
f2 (ppm)
f1 (ppm)

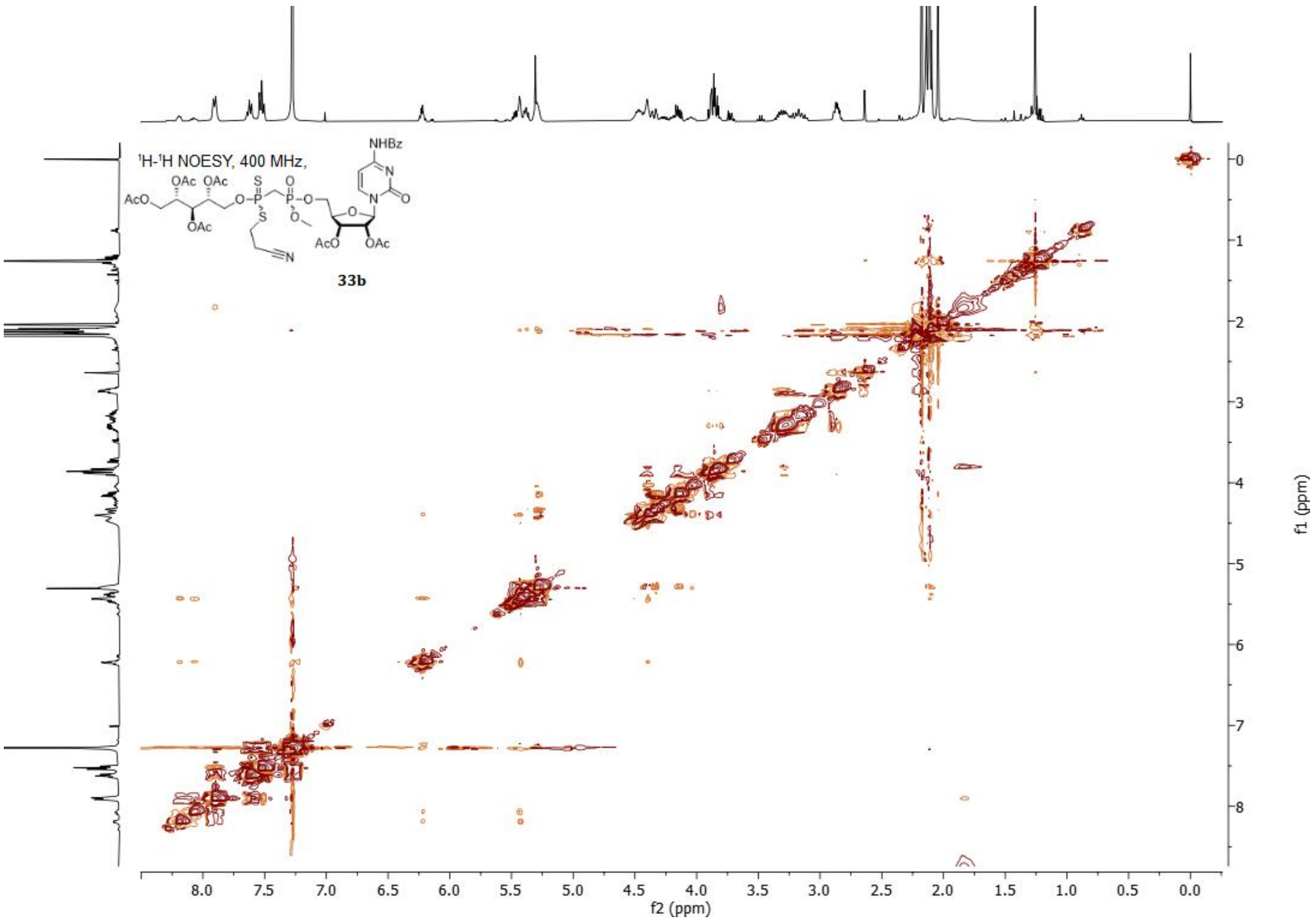

¹H-¹H NOESY, 400 MHz,
NHBz
AcO
OAc
33b
f2 (ppm)
f1 (ppm)

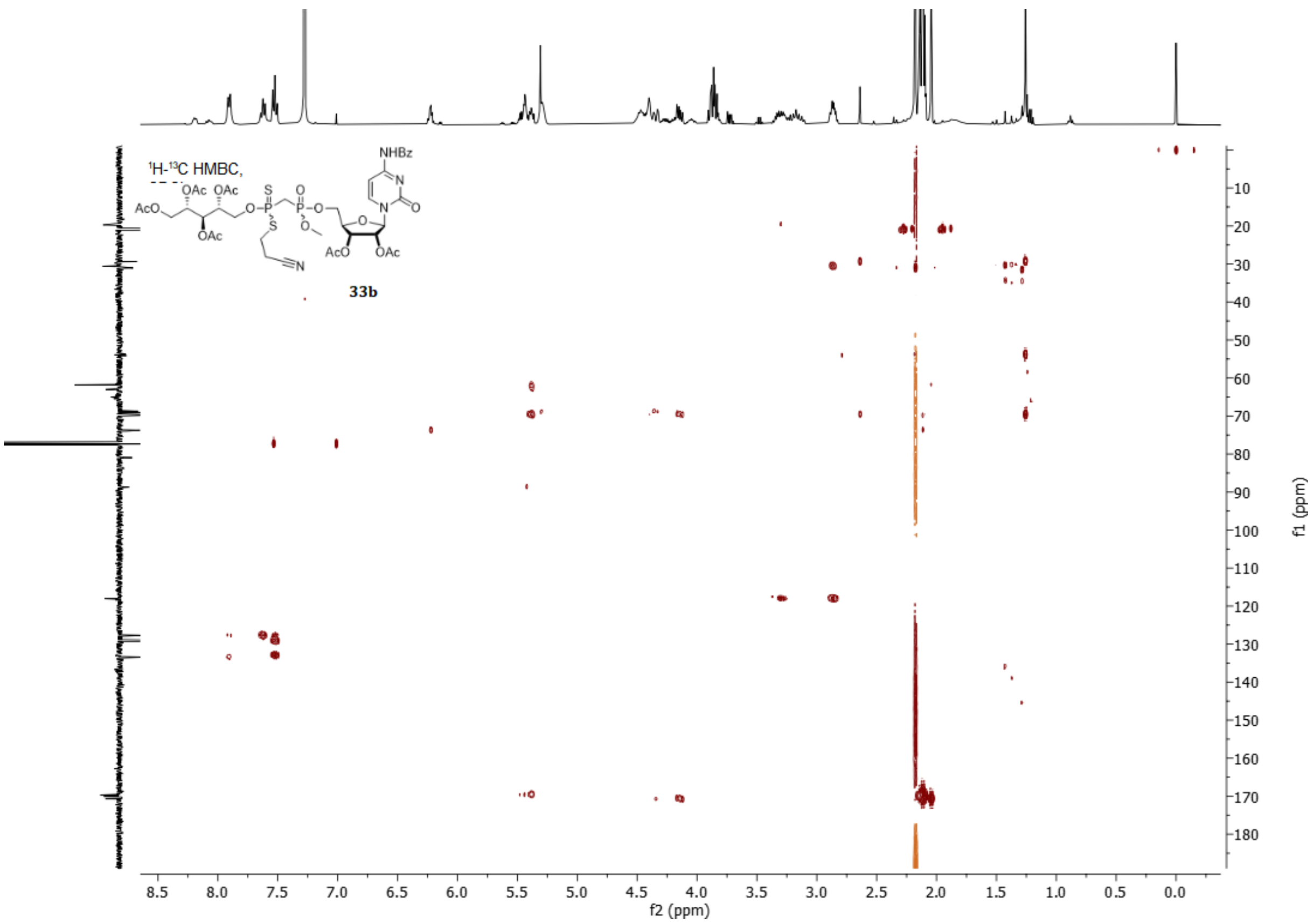

me-CDP-βS ribitol 34a

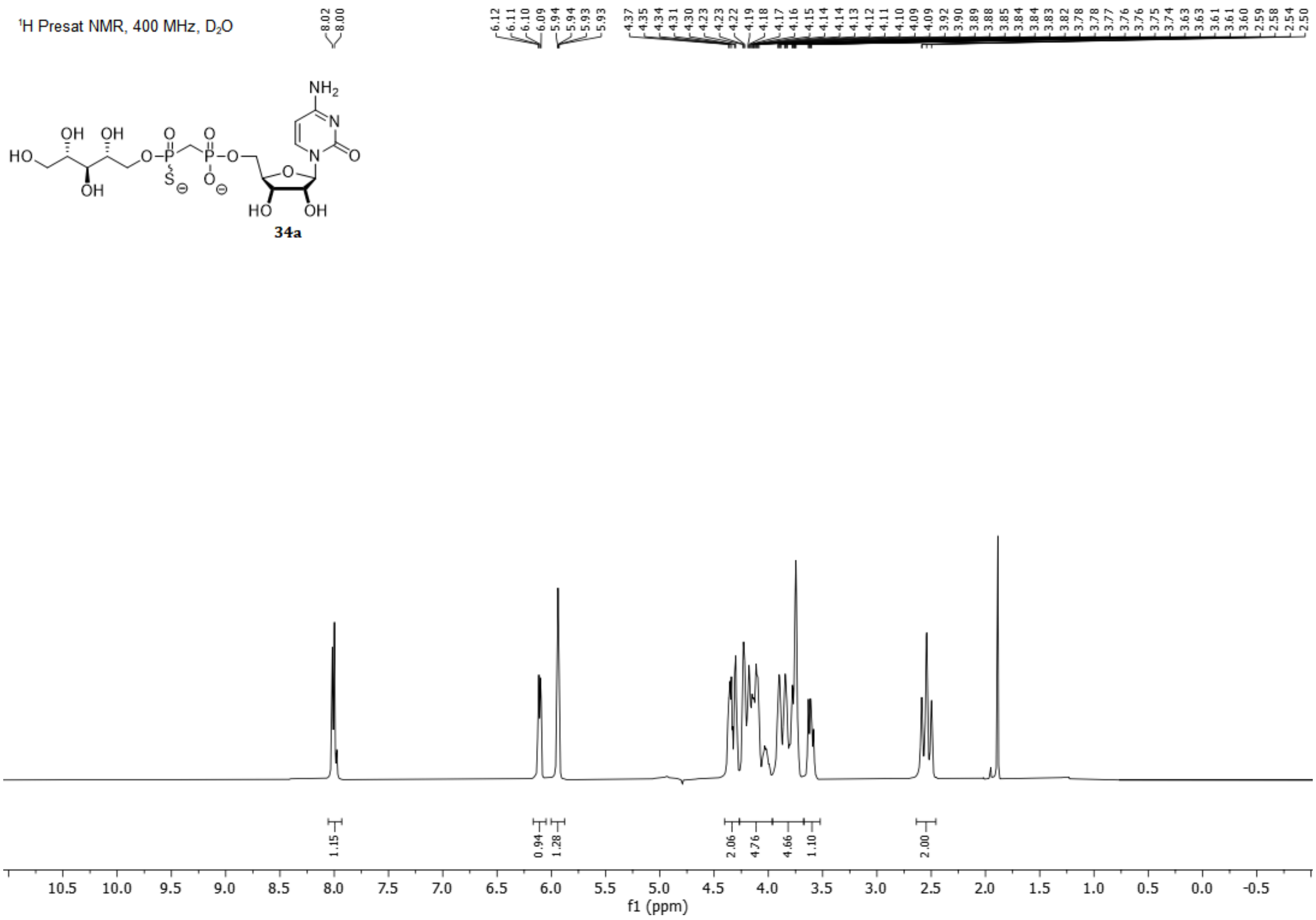

^{13}C NMR, 101 MHz, D_2O

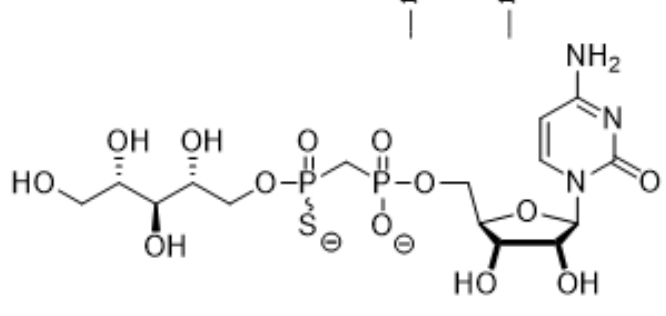

34a

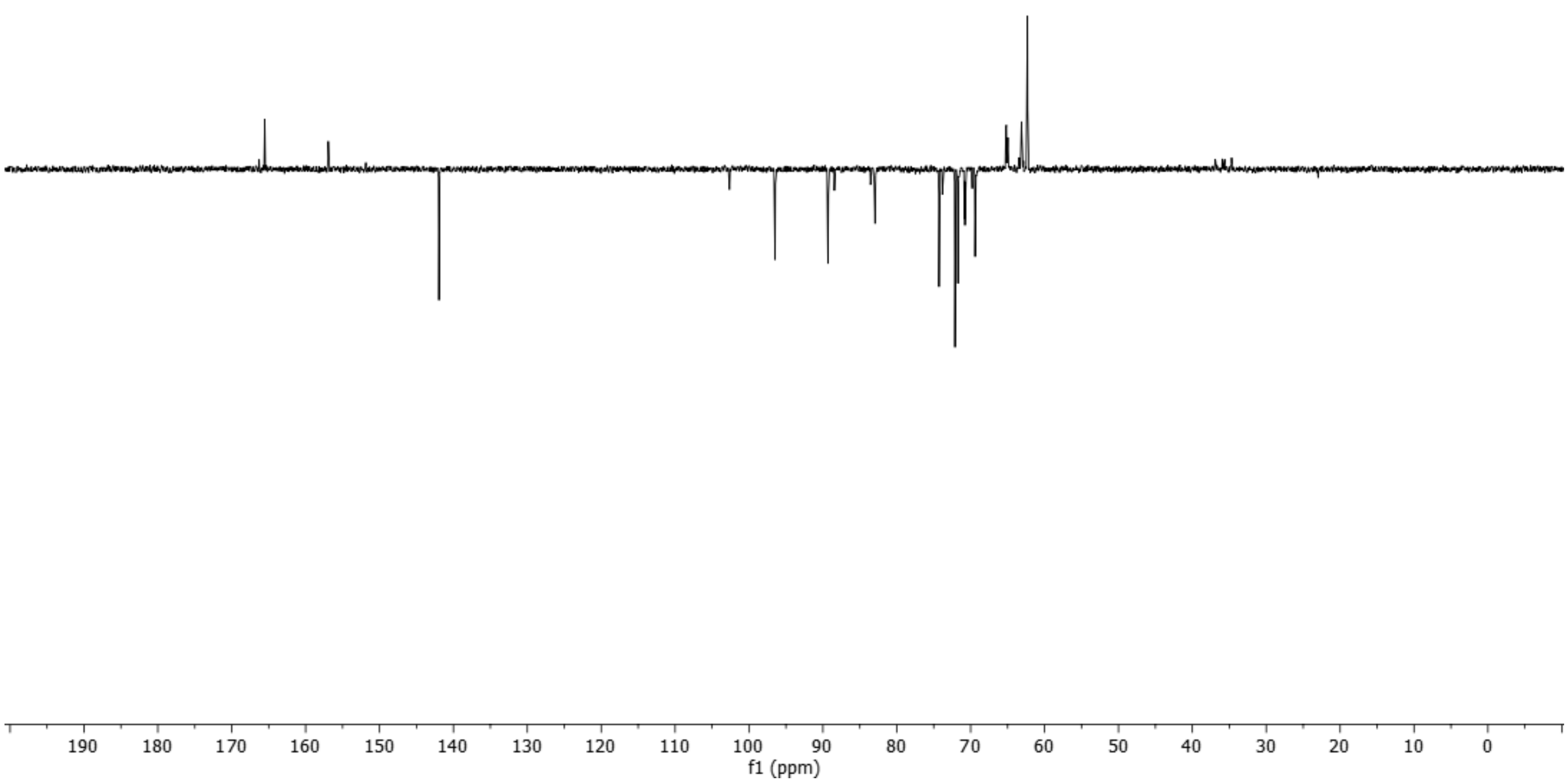

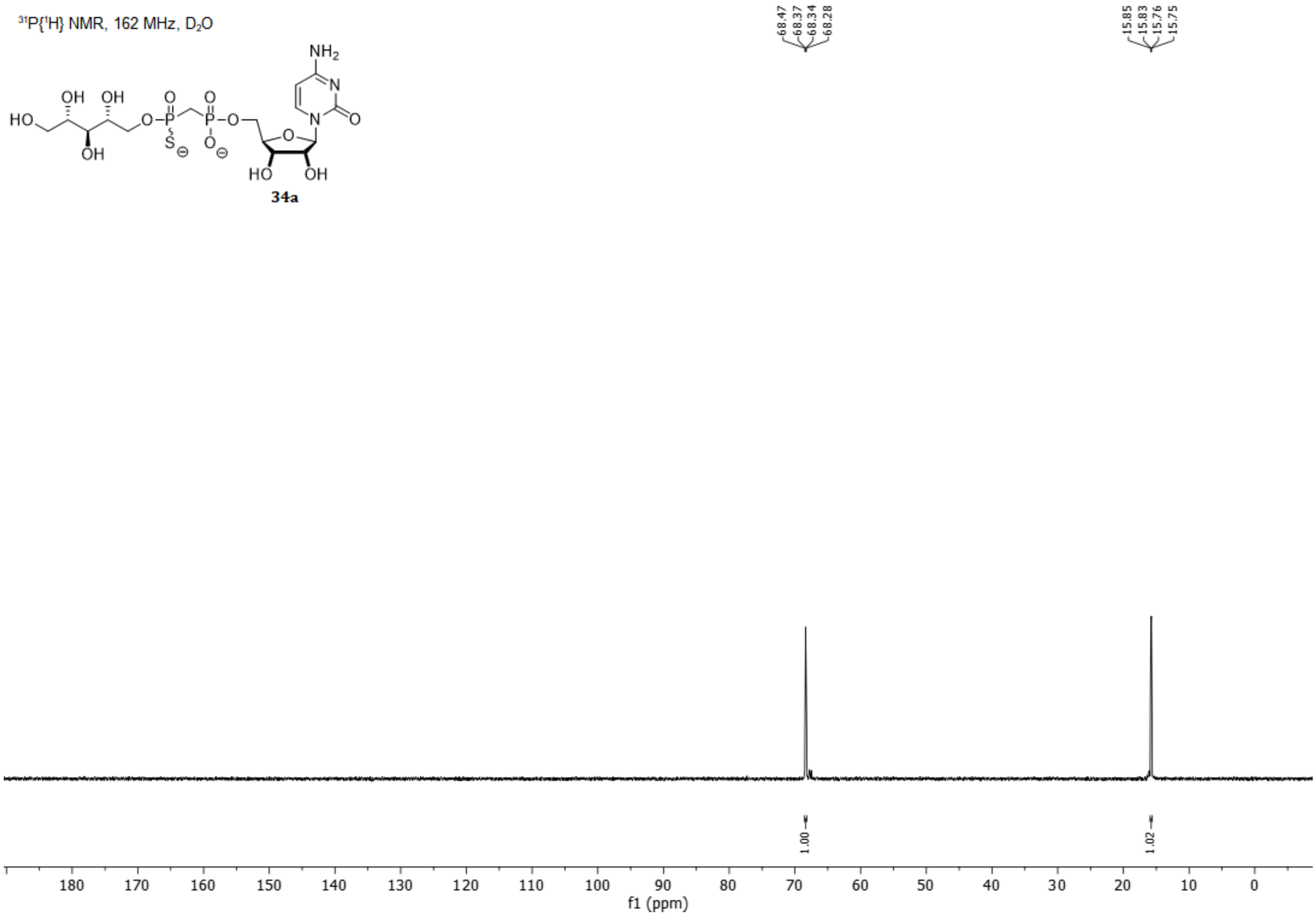

$^{31}P\{^1H\}$ NMR, 162 MHz, D_2O
34a
68.47
68.37
68.34
68.28
15.85
15.83
15.76
15.75
1.00
1.02
f1 (ppm)

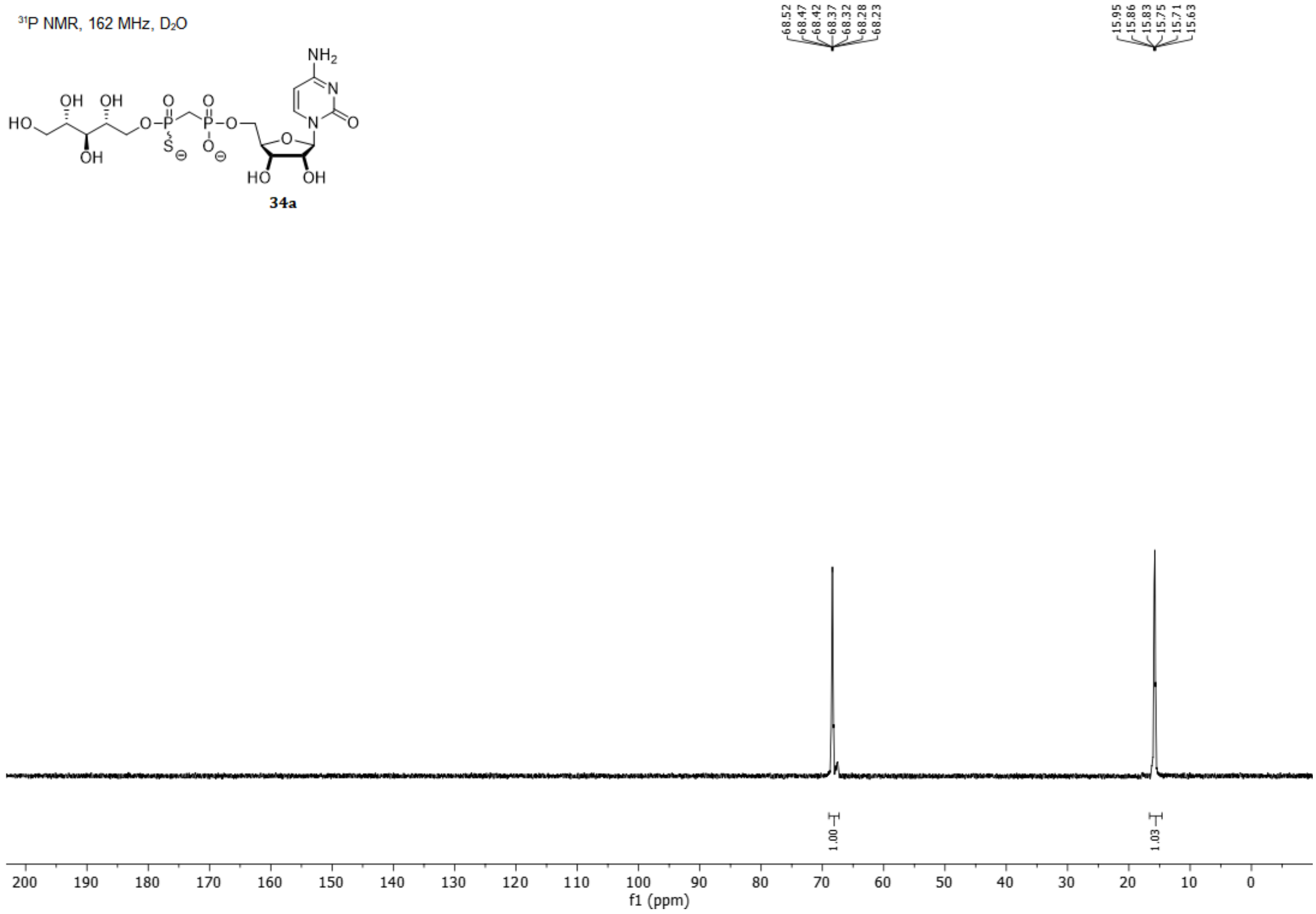

^{31}P NMR, 162 MHz, D_2O
34a
68.52
68.47
68.42
68.37
68.32
68.28
68.23
15.95
15.86
15.83
15.75
15.71
15.63
1.00
1.03
f1 (ppm)

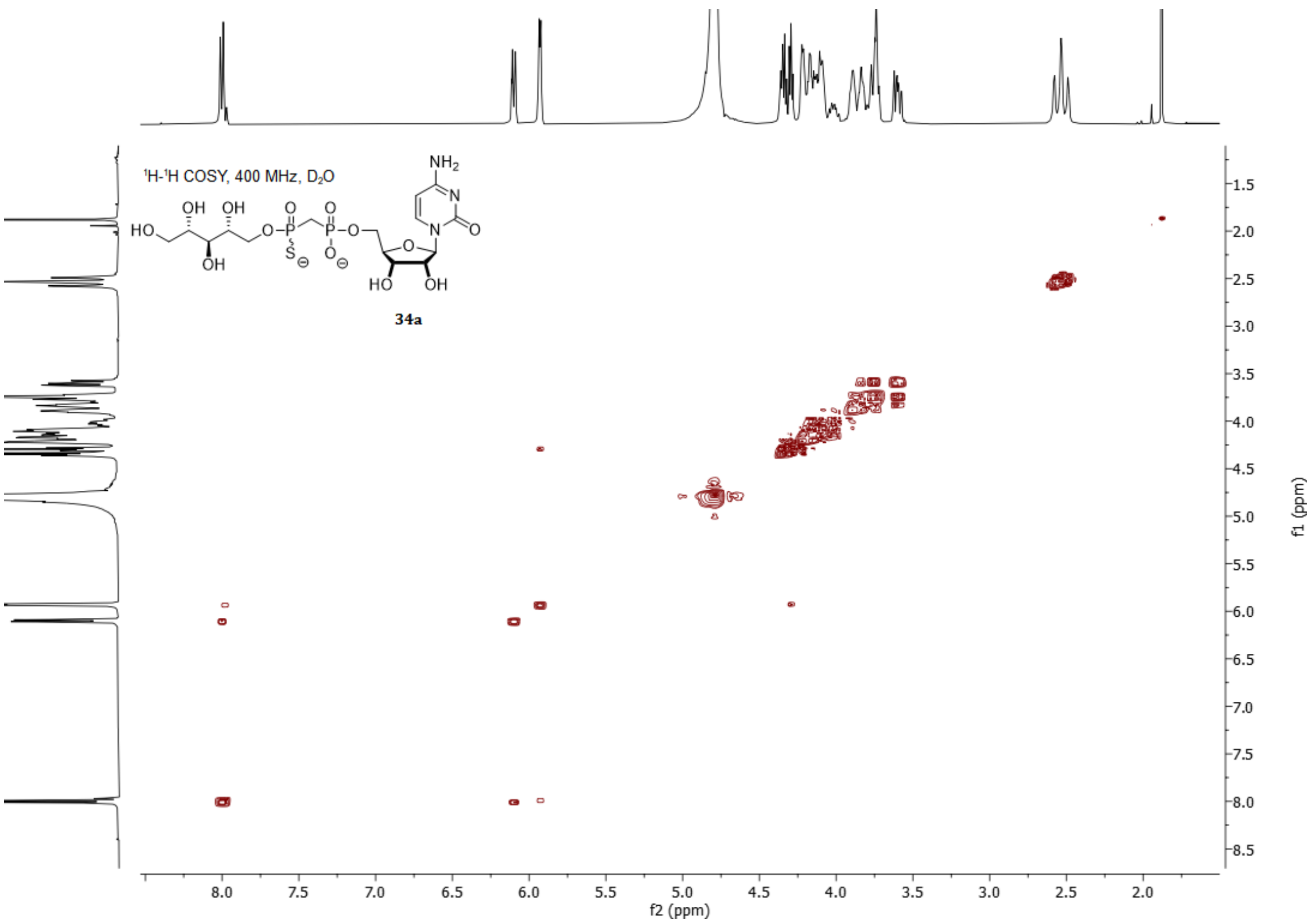
1H-1H COSY, 400 MHz, D2O
34a
f2 (ppm)
f1 (ppm)

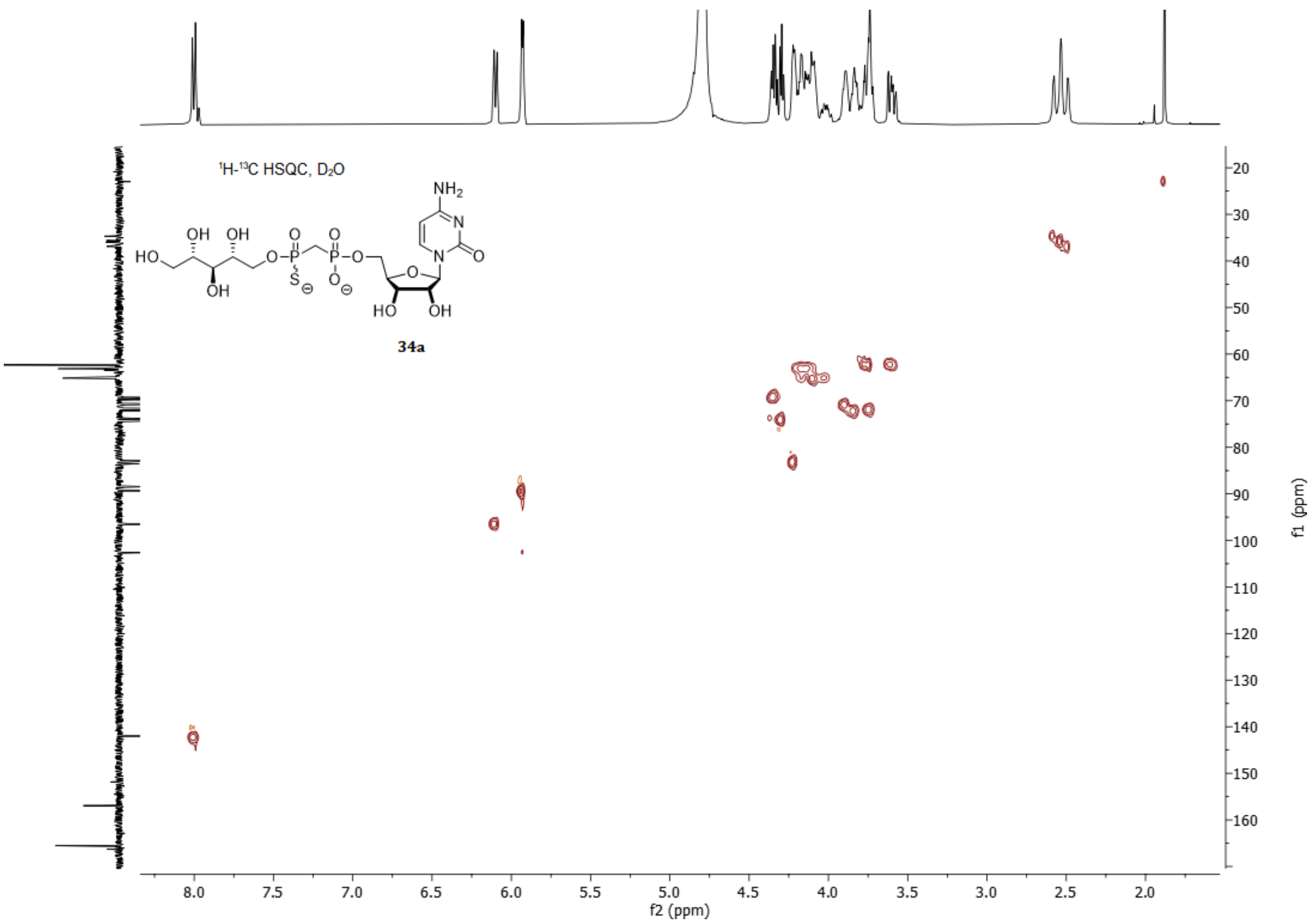
1H-13C HSQC, D2O
34a
f2 (ppm)
f1 (ppm)

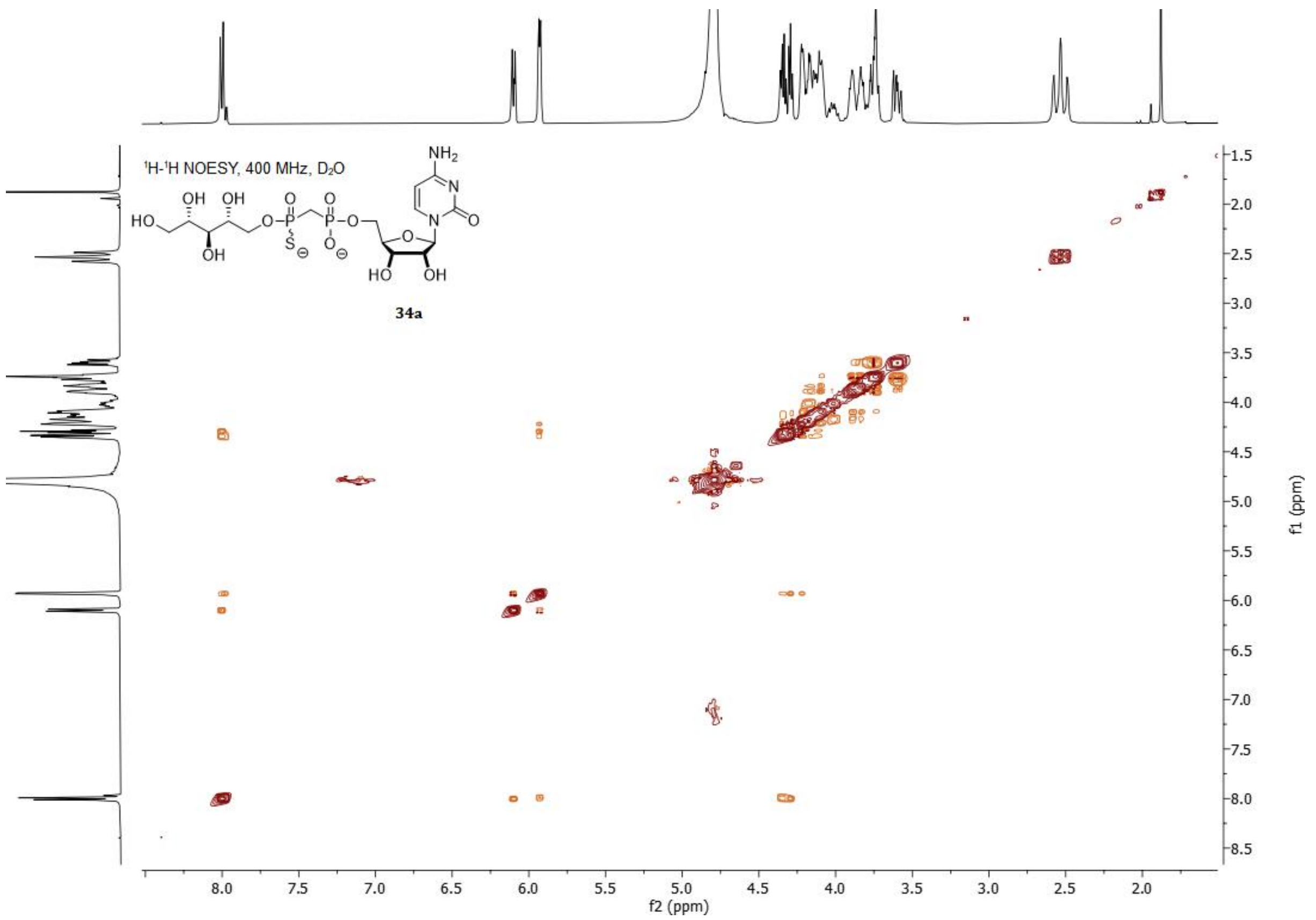

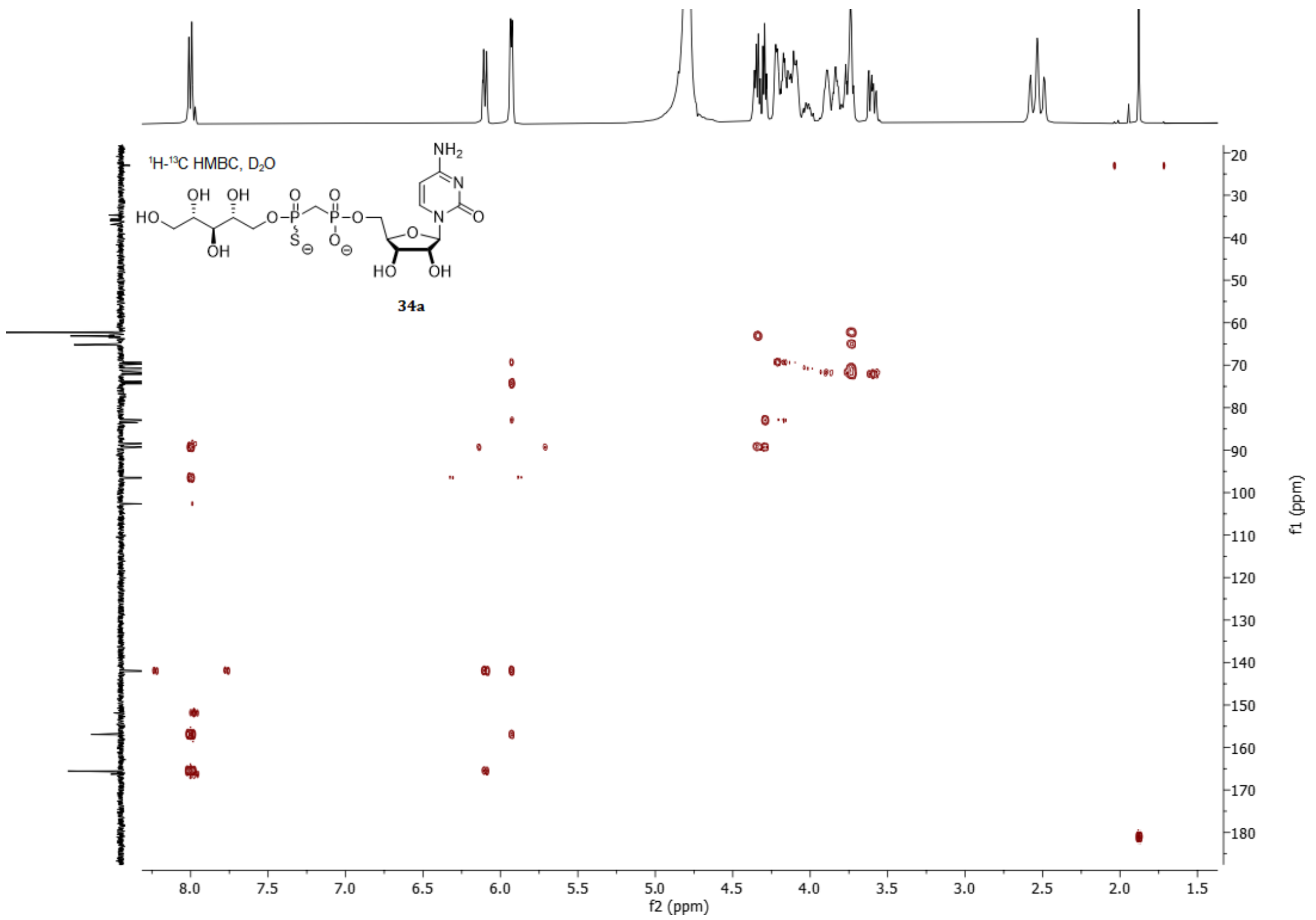

me-CDP-βS$_2$-ribitol 34b

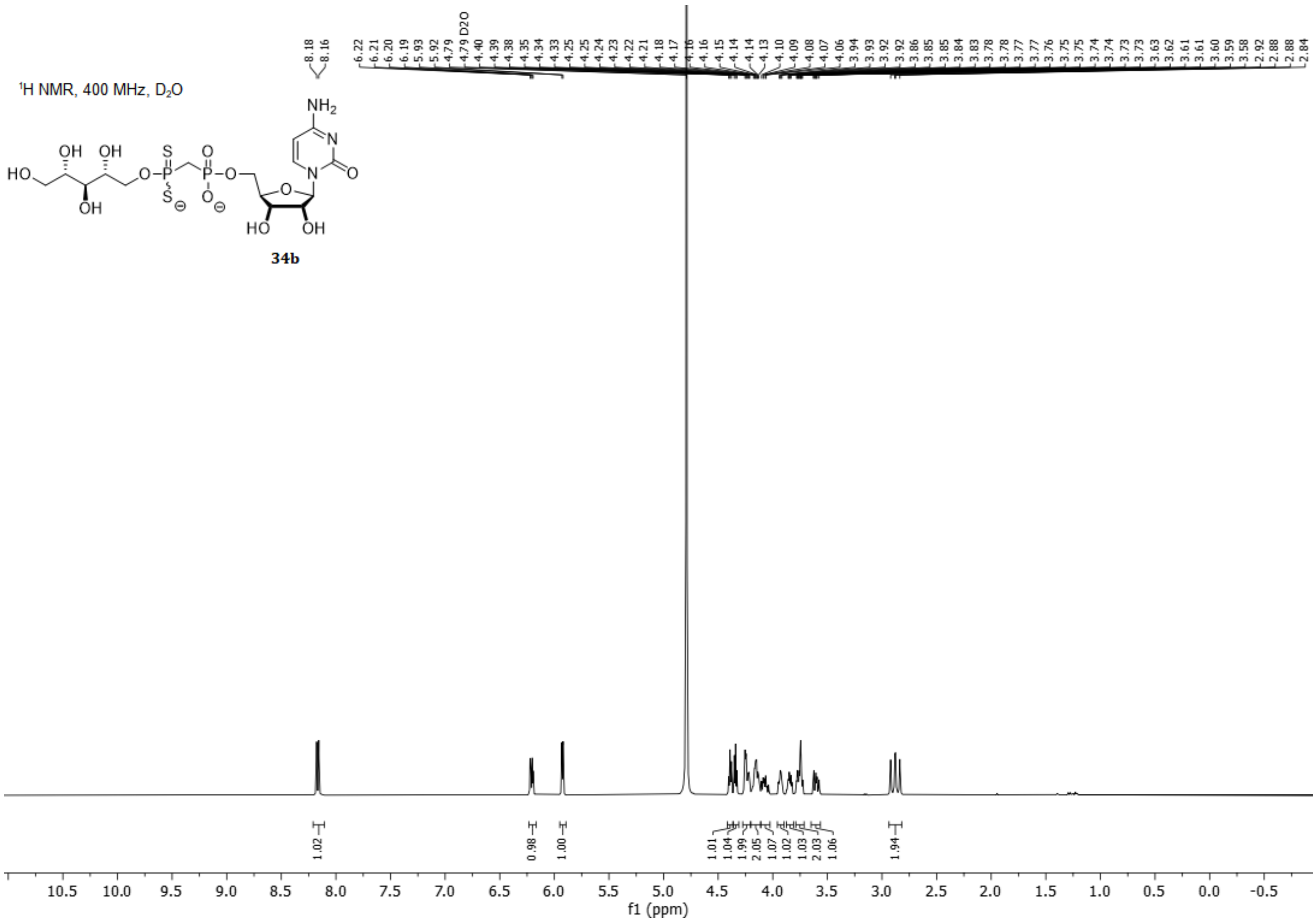
1H NMR, 400 MHz, D2O
34b
f1 (ppm)

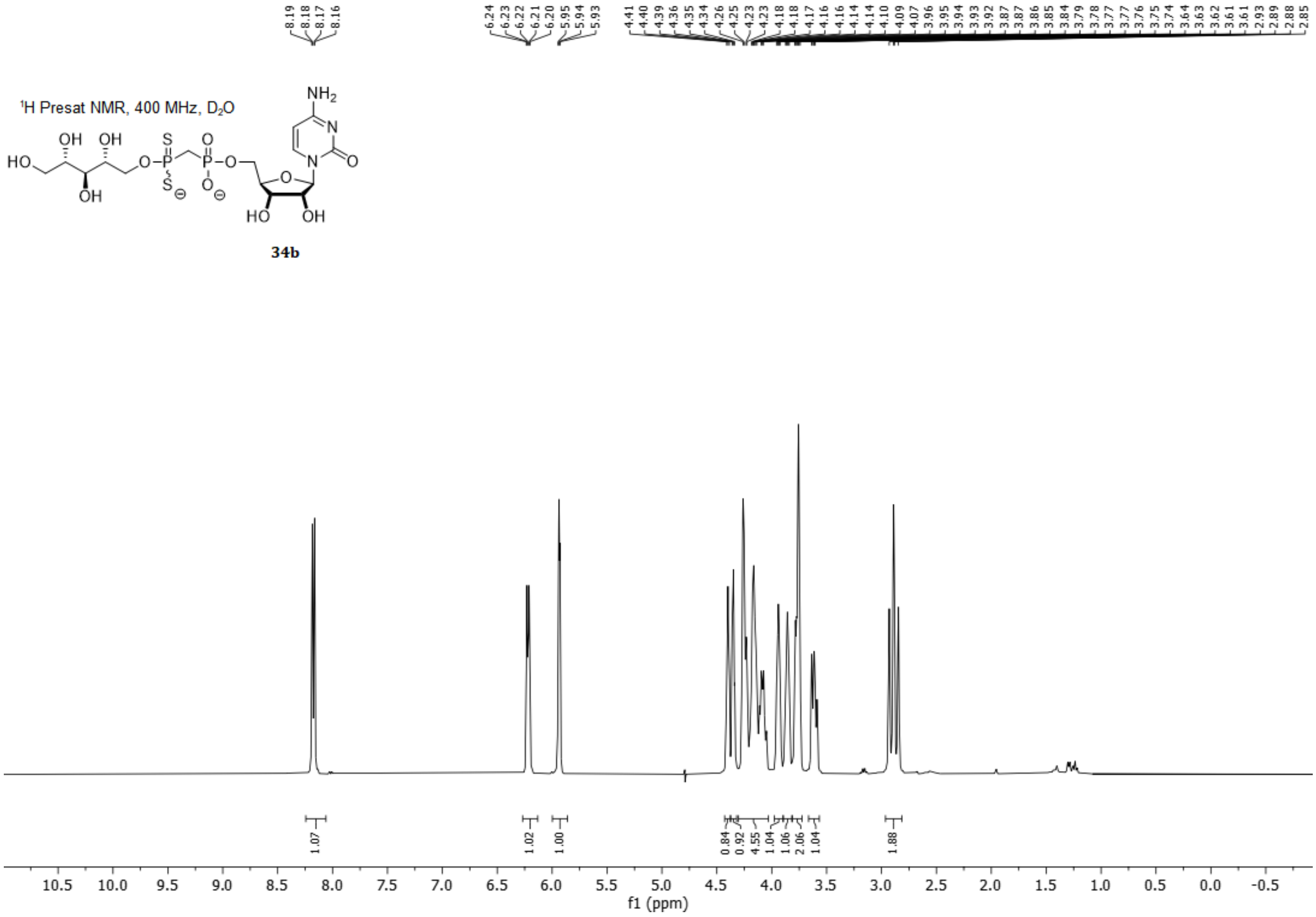
1H Presat NMR, 400 MHz, D2O
34b
f1 (ppm)

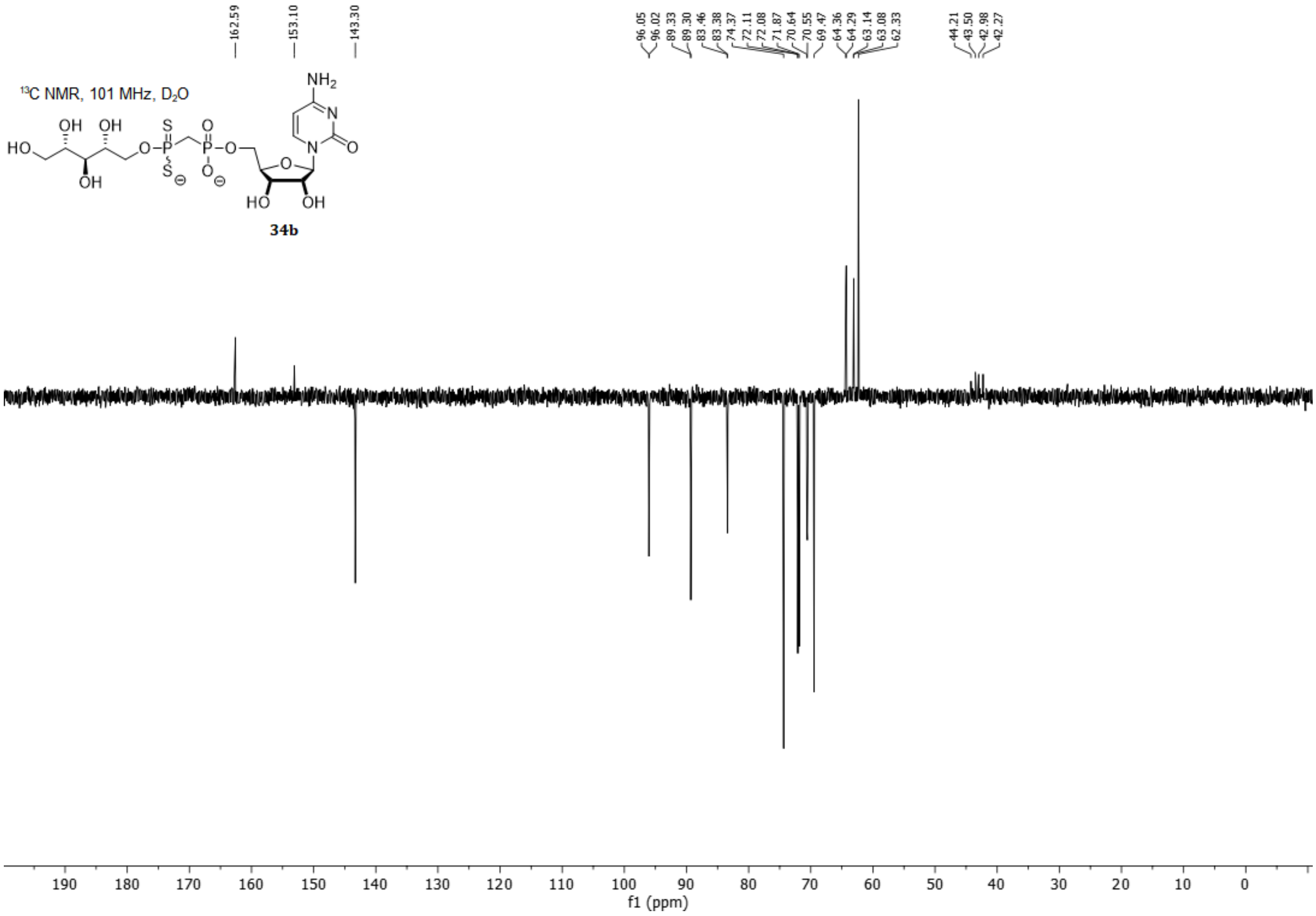

^{13}C NMR, 101 MHz, D_2O
NH2
HO OH OH O P S S O O O N N O HO OH OH
34b
162.59
153.10
143.30
96.05 96.02 89.33 89.30 83.46 83.38 74.37 72.11 72.08 71.87 70.64 70.55 69.47 64.36 64.29 63.14 63.08 62.33
44.21 43.50 42.98 42.27
190 180 170 160 150 140 130 120 110 100 90 80 70 60 50 40 30 20 10 0
f1 (ppm)

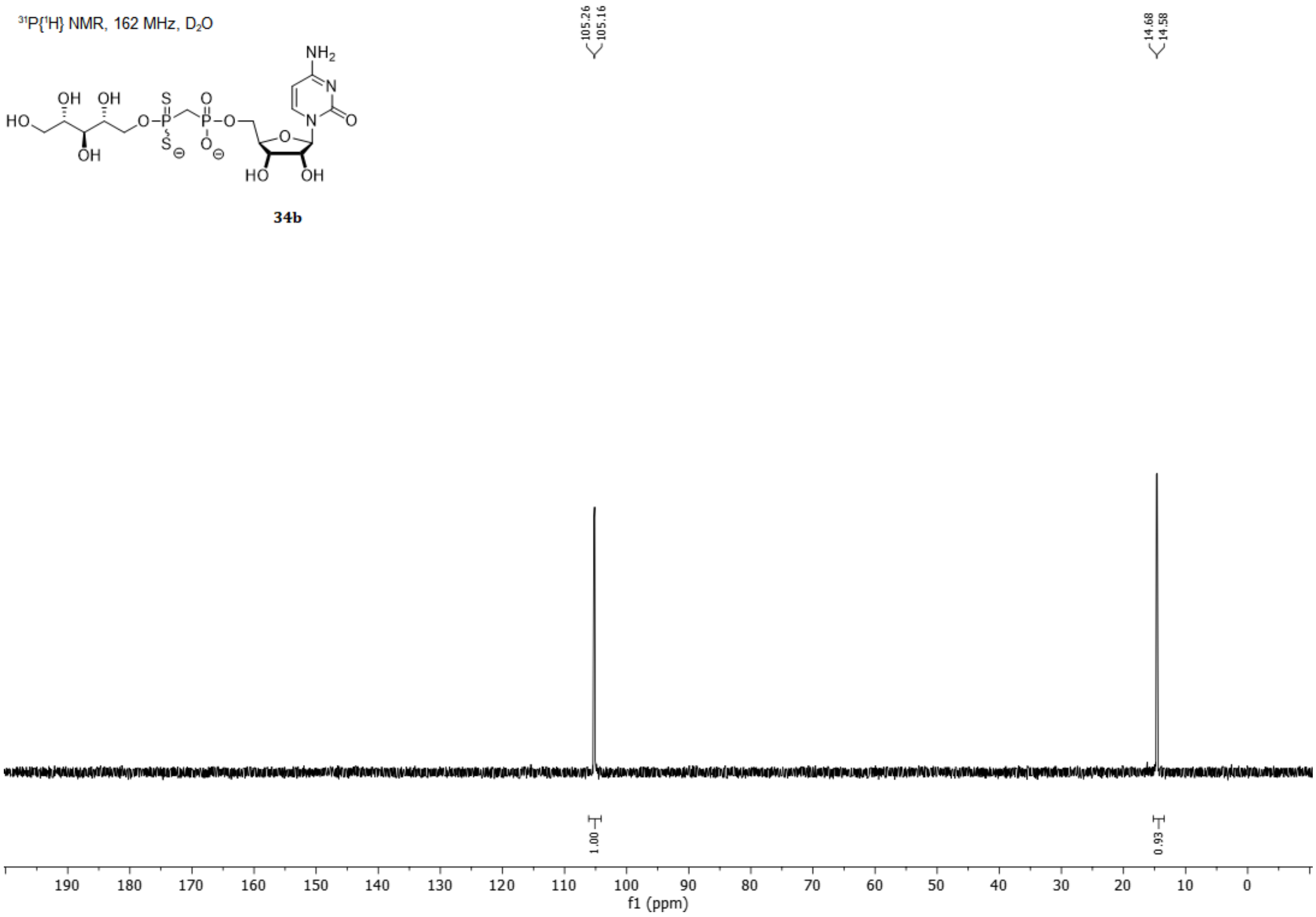

$^{31}P\{^1H\}$ NMR, 162 MHz, D_2O
NH2
HO OH OH O P S S O O O N N O HO OH OH
34b
105.26 105.16
14.68 14.58
1.00
0.93
190 180 170 160 150 140 130 120 110 100 90 80 70 60 50 40 30 20 10 0
f1 (ppm)

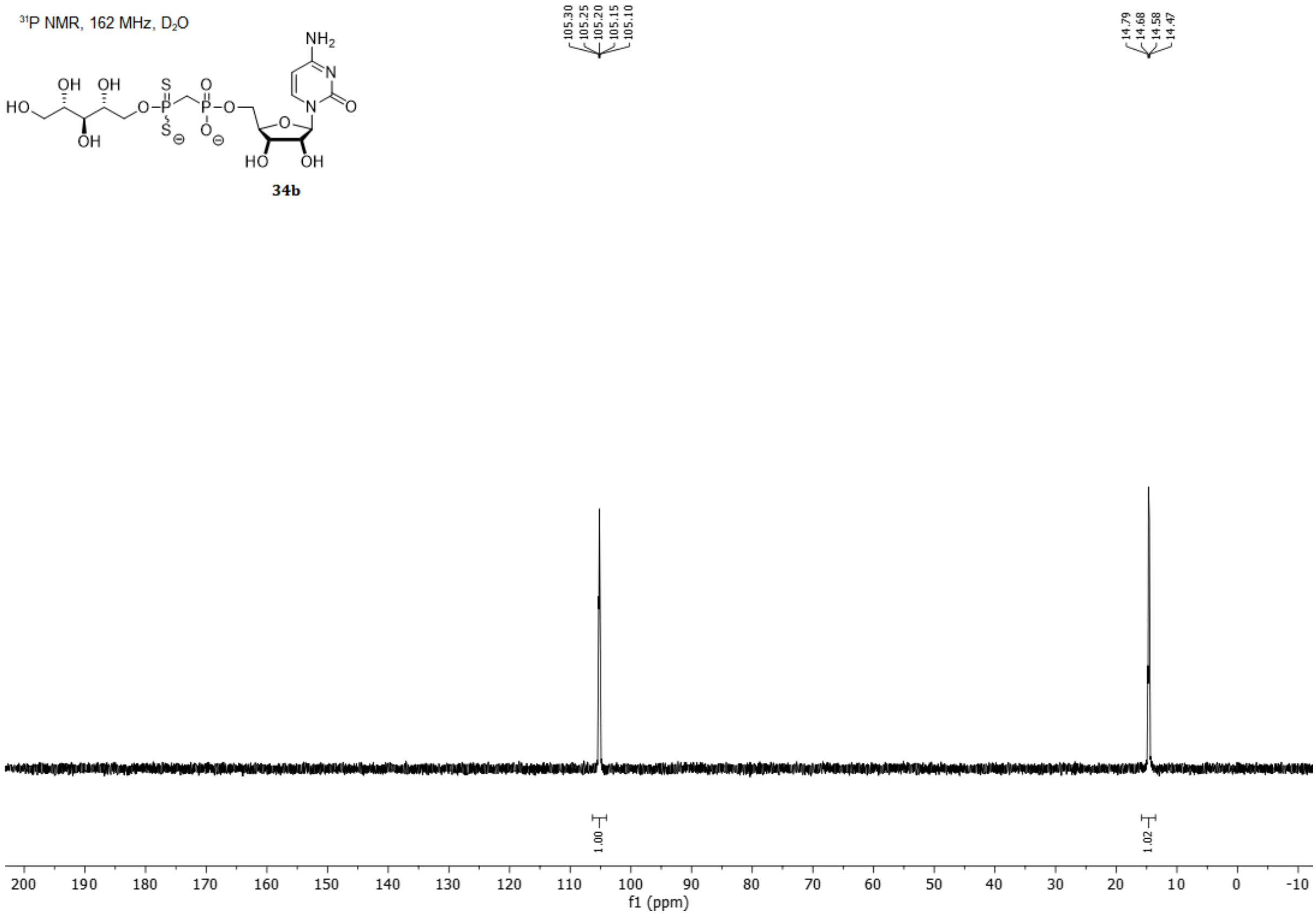

³¹P NMR, 162 MHz, D₂O
34b
105.30
105.25
105.20
105.15
105.10
14.79
14.68
14.58
14.47
1.00
1.02
f1 (ppm)

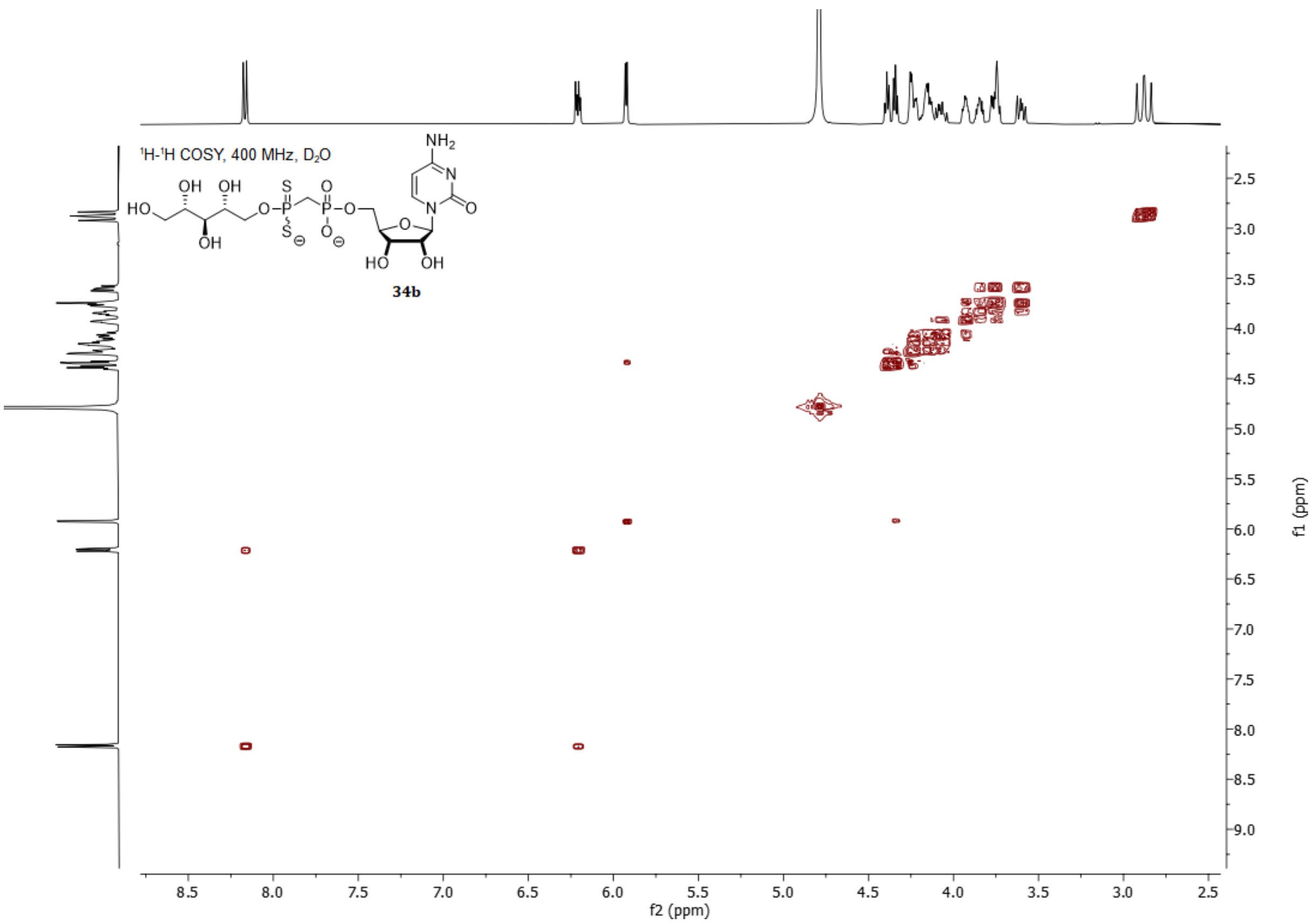

¹H-¹H COSY, 400 MHz, D₂O
34b
f2 (ppm)
f1 (ppm)

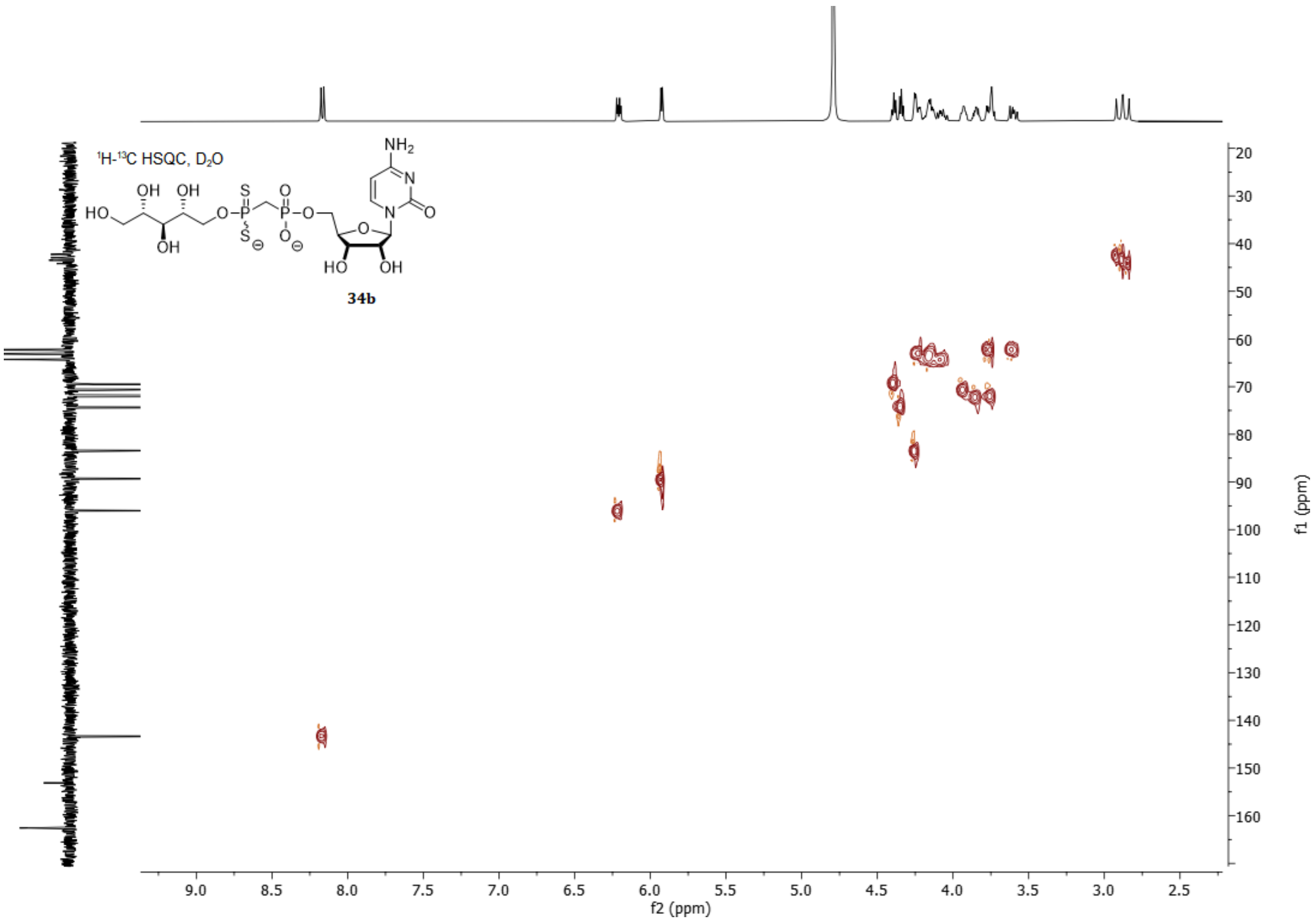
1H-13C HSQC, D2O
34b
f2 (ppm)
f1 (ppm)

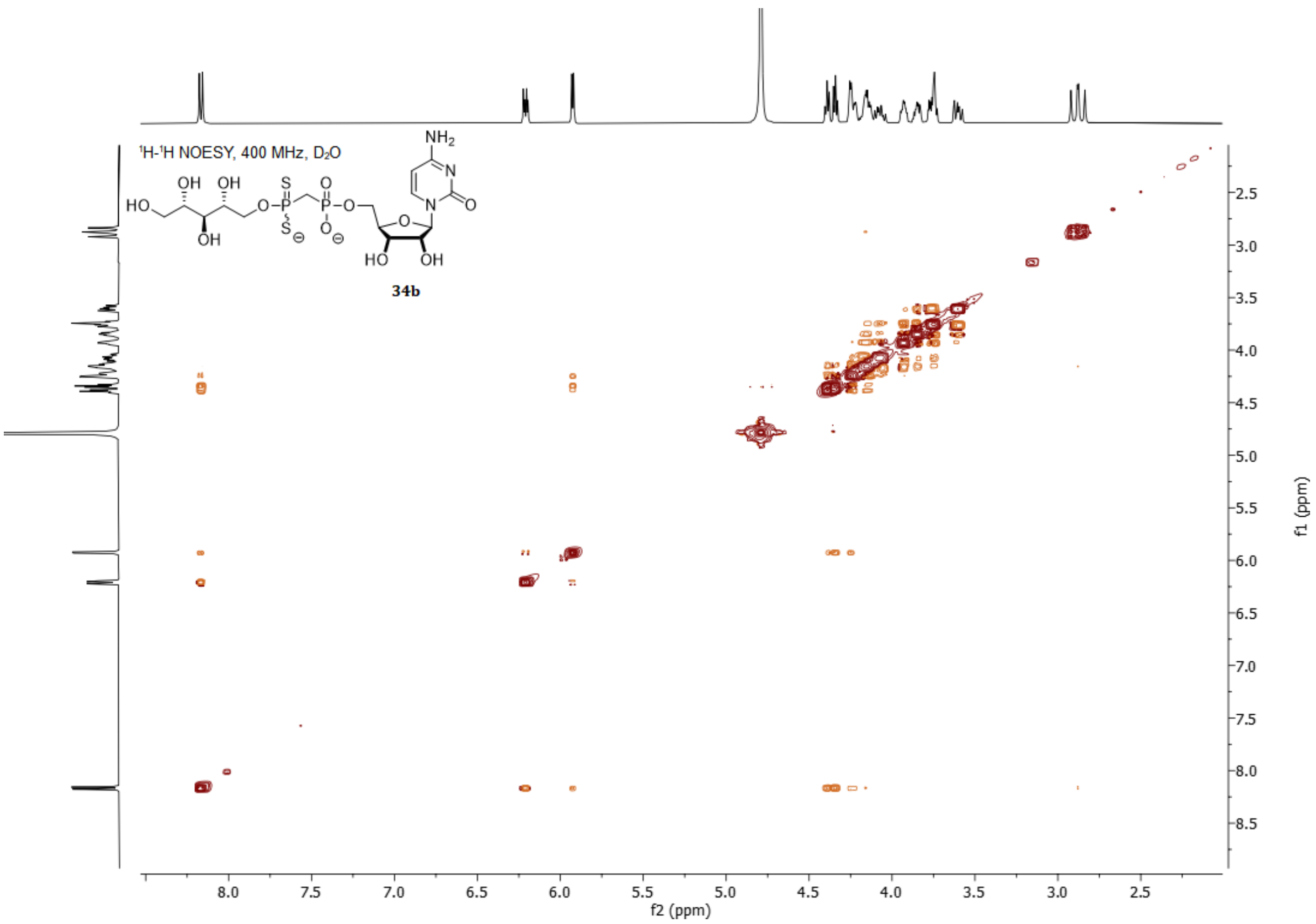
1H-1H NOESY, 400 MHz, D2O
34b
f2 (ppm)
f1 (ppm)

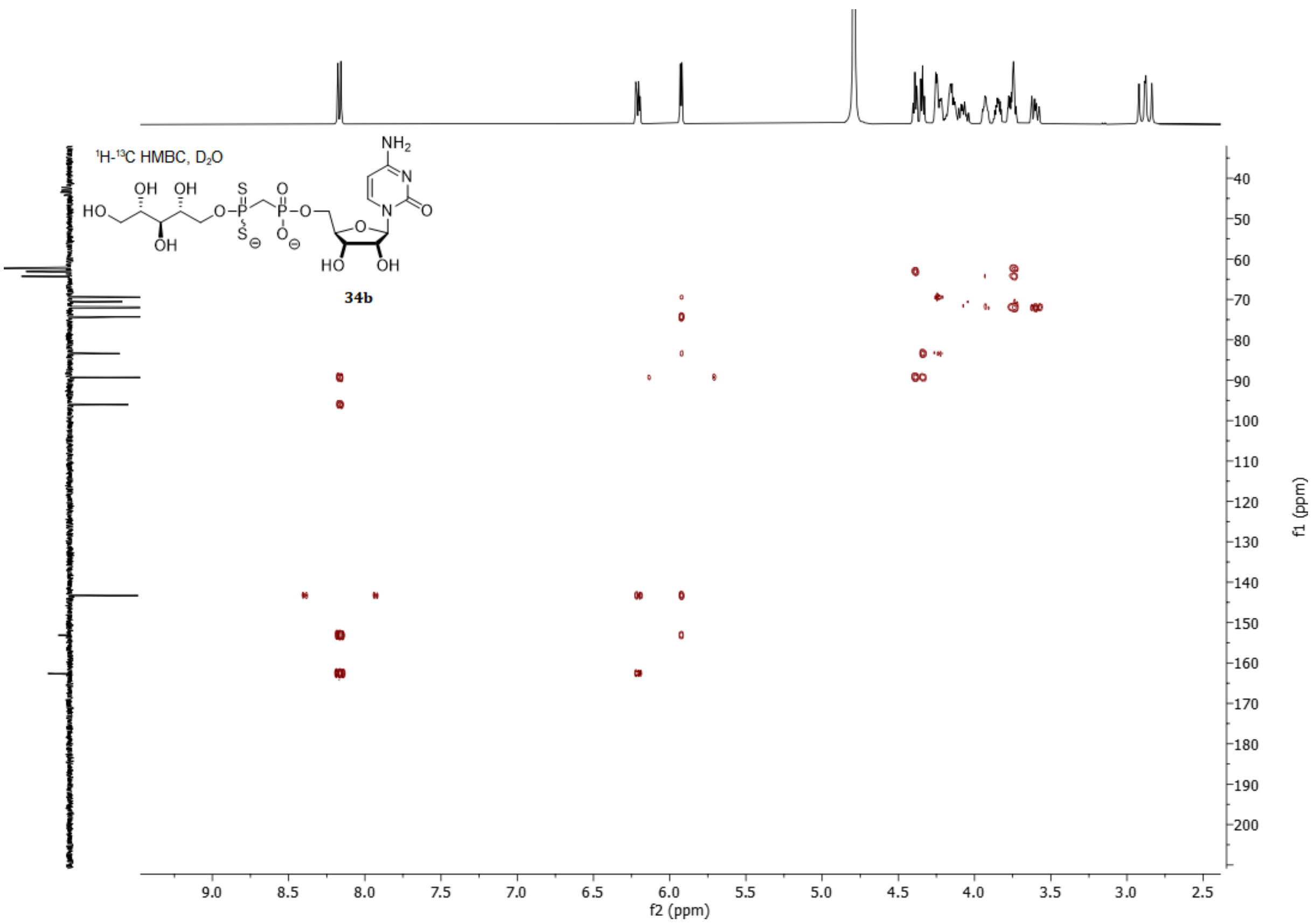

1H-13C HMBC, D2O
34b
f2 (ppm)
f1 (ppm)
